# Analysis of Soil Fungal and Bacterial Communities in Tianchi Volcano Crater, Northeast China

**DOI:** 10.3390/life11040280

**Published:** 2021-03-26

**Authors:** Xiao Wang, Lorenzo Pecoraro

**Affiliations:** School of Pharmaceutical Science and Technology, Tianjin University, 92 Weijin Road, Nankai District, Tianjin 300072, China; wang_xiao1996@163.com

**Keywords:** fungi, bacteria, microbial community, volcanic soil, high-altitude, extreme environment, Illumina sequencing, microbial network analysis

## Abstract

High-altitude volcanoes, typical examples of extreme environments, are considered of particular interest in biology as a possible source of novel and exclusive microorganisms. We analyzed the crater soil microbial diversity of Tianchi Volcano, northeast China, by combining molecular and morphological analyses of culturable microbes, and metabarcoding based on Illumina sequencing, in order to increase our understanding of high-altitude volcanic microbial community structure. One-hundred and seventeen fungal strains belonging to 51 species and 31 genera of Ascomycota, Basidiomycota and Mucoromycota were isolated. *Penicillium*, *Trichoderma*, *Cladosporium*, *Didymella*, *Alternaria* and *Fusarium* dominated the culturable fungal community. A considerable number of isolated microbes, including filamentous fungi, such as *Aureobasidium pullulans* and *Epicoccum nigrum*, yeasts (*Leucosporidium creatinivorum*), and bacteria (*Chryseobacterium lactis* and *Rhodococcus* spp.), typical of high-altitude, cold, and geothermal extreme environments, provided new insights in the ecological characterization of the investigated environment, and may represent a precious source for the isolation of new bioactive compounds. A total of 1254 fungal and 2988 bacterial operational taxonomic units were generated from metabarcoding. Data analyses suggested that the fungal community could be more sensitive to environmental and geographical change compared to the bacterial community, whose network was characterized by more complicated and closer associations.

## 1. Introduction

Microorganisms are considered an essential component of natural environments [1]. They are ubiquitous in nature based on their characteristics, such as small size, flexible capability to exploit nutrients and adaptability to unfavorable and extreme environmental conditions [1]. Microbial communities in volcanic environments are of particular interest for research on the emergence and evolution of life due to the unique extreme conditions that characterize these natural habitats [2]. Extremophilic microorganisms inhabiting volcanic environments can be a source of important biotechnological products like antibiotics, bio-fertilizers or bio-control agents [3,4,5]. New extremophiles have been isolated from different, very peculiar environments, which are often considered “empty” in the biological sense [6]. Novel microbial species have been recently described from barren alpine environments [7,8] and high-altitude volcanic soils [4,5].

Both morphological culture-based and molecular approaches are generally used to characterize environmental microbial communities. Culturable microorganisms provide more detailed information for their identification at species level, and for understanding their role in environmental processes, but only less than 5% of the microorganisms on Earth are culturable in lab condition, due to the selectivity of media and culture conditions [9,10]. To overcome this limitation, diverse methods have been developed to obtain genetic information on the presence of microorganisms living in natural environments. These methods allow the culture-independent analysis of the total microbial genomes called “metagenome” in a particular environment [2,11,12,13]. The development of molecular biological techniques (particularly high-throughput sequencing) represents a powerful tool to access a much larger proportion of microbial communities, compare to traditional culture-based methods, by means of environmental sample total DNA extraction [14].

Changbai Mountain Nature Reserve is located in the northeast of China. It is considered one of the most well-protected and conserved natural ecosystems in China [15]. Many new microbial species have been described in this area, which is particularly predisposed to harbor uncommon microorganisms due to its distinctive landforms and complex climatic conditions [16]. For instance, a novel bacterial strain *Bacillus methylotrophicus* was isolated from the soil near the roots of *Pinus koraiensis* plants at an altitude of 2749 m a.s.l. [4], while a novel thermophilic anaerobic bacterium *Fervidobacterium changbaicum* was collected from the mixture of water and mud from a hot spring, in Changbai Mountain [17]. A number of novel macro-mycete taxa were also described from the highly diverse fungal community of this Nature Reserve [18,19]. Tianchi Volcano is located at the highest point of Changbai Mountain ranges [20]. It is an active volcano with the highest potential eruption risk in China [21,22]. Water accumulates at the top of the volcanic cone and forms Tianchi lake, the highest volcanic lake in China, at an altitude of 2189 m a.s.l. The lake is surrounded by 16 peaks, the highest peak reaching the altitude of 2749.5 m a.s.l. [23,24]. The crater of Tianchi Volcano is the accumulation area of volcanic lavas and debris, which are mainly composed by Ti, Fe, Mn, Si, Al, Ca, Na, K and Mg oxides, trace elements, including high concentration of rare-earth elements, and heavy metals, such as Zn, Pb and Cu [23,25].

To date, a few studies on microbial diversity have been performed in Changbai Mountain Nature Reserve, mainly focusing on the structure and function of the soil microbial communities in vertical vegetation zones, with little attention to microbial communities in the high crater area of Tianchi Volcano. For instance, the fungal and bacterial diversity and community composition along an elevation gradient (2000–2500 m a.s.l.) on the northern alpine tundra belt of Changbai Mountain were analyzed using Illumina sequencing [26,27]. Other studies focused on specific soil microbial diversity associated with vegetation zones [28,29,30,31]. Zhao et al. characterized microbes in rhizosphere soils of Cowskin Azalea (*Rhododendron aureum*) using culture-dependent methods [32]. The diversity of culturable forest micro-fungi in different vegetational belts from 700 to 2600 m a.s.l. was described by Yang et al. [16]. In this study, we focused on the mountaintop area of Tianchi Volcano, which was expected to harbor a peculiar microbial community adapted to the particularly extreme environmental conditions characterizing the highest elevation montane zones, including colder temperatures, and higher exposure to wind and solar radiation. We performed a comprehensive analysis of the soil fungal and bacterial diversity colonizing the crater margin of Tianchi Volcano, using a combination of molecular and morphological analyses of culturable microbes, and metabarcoding based on Illumina sequencing, in order to shed light on the structure of the microbial community inhabiting this unexplored extreme environment and increase our understanding of high-altitude volcanic microorganism network.

## 2. Materials and Methods

### 2.1. Study Area and Sampling

The study plots were located along the crater margin of Tianchi Volcano, which is part of the Changbai Mountain Nature Reserve (41°41′49′′–42°25′18′′ N, 127°42′55′′–128°16′48′′ E) in Jilin Province, northeast China [32]. This Nature Reserve, at an altitude ranging from 500 m to 2691 m a.s.l., is characterized by a typical mountain climate, with low temperature and heavy precipitation, being the mean annual temperature 4.9–7.3 °C, and the mean annual precipitation over 800 mm [33]. The Changbai Mountain high altitude zone environment is constituted by a tundra belt, which is distributed between 1950 m and 2650 m a.s.l., and includes volcanic, glacial and periglacial landforms. The specific mean annual temperature (−4.8 °C) and precipitation (1154 mm) of this area are typical of tundra-periglacial climate. The tundra belt is covered with snow for about 6 months every year, from mid-October to mid-May [26]. The Tianchi Volcano crater area, around Tianchi lake, belongs to the tundra belt.

**Figure 1:**
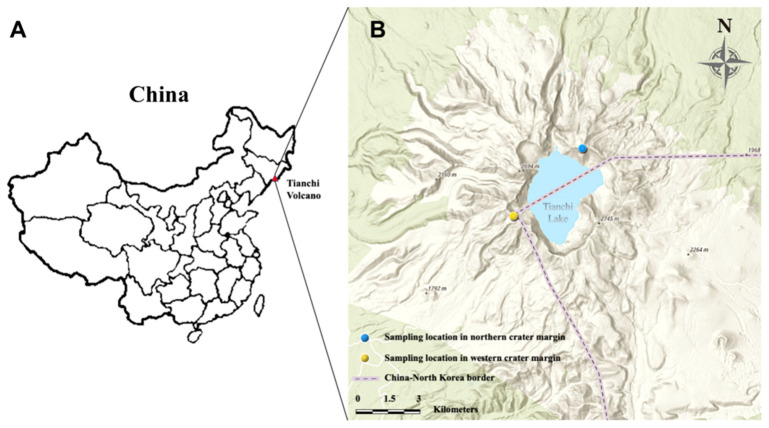
Location of Tianchi Volcano in China (**A**) and sampling area in northern and western margin of Tianchi crater (**B**). The map was generated from ArcGIS Online (https://maps.arcgis.com/index.html, accessed on 1 January 2021) [34].

During 26–27 October 2019, soil stone mixture samples from northern and western crater area of Tianchi Volcano were collected. Six plots of 20 m diam were randomly selected at the very top of the mountain, along the crater margin, on each of the two analyzed sides of the crater to collect a total of 12 soil stone mixture samples. All plots were completely devoid of vegetation. The number of plots was chosen based on the accessibility of the crater site and following the sampling restrictions in the protected area. Each sample was collected from the center of the plot at shallow depth (1–5 cm), after removing the surface layer (ca. 1 cm). Figure 1 shows the geographical distribution of sampling locations, while specific information of coordinates and altitudes are listed in Table 1. The samples were immediately stored on ice in insulated containers. After returning to the laboratory, each sample was divided into two subsamples, one stored at 4 °C for isolation of culturable microbes, the other one stored at −80 °C for metabarcoding analysis. The pH of soil samples was measured adding distilled water to ground material at a ratio of 1:2.5 (w/v).

### 2.2. Cultivable Microbe Isolation, Microscopy and Molecular Analyses

Microbes were isolated following a modified dilution plate method [35]. Soil stone mixture suspensions were prepared as follows: samples were first evenly grounded in sterilized mortars, then 10 g of each sample were suspended in 40 mL sterile water to obtain the diluted suspension. 1 mL of uniform diluted solution was taken and further diluted in 9 mL sterile water. All sample diluted suspensions were finally shaken at 220 rpm for 5 min. 100 μL of suspension from each of the two dilutions were plated on Potato Dextrose Agar (PDA) amended with 100 mg/L penicillin and streptomycin to reduce the growth of bacteria to a minimum level and favor the isolation of fungi. Petri dishes were sealed and incubated at room temperature (25 °C), in the darkness. After microbial colonies appeared, the different morphotypes were accurately selected for strain isolation, based on characters such as texture and pigmentation. Single colonies were picked and transferred to new Petri dishes containing the same medium to obtain pure cultures.

Fungal morphological traits (such as branched septate hyphae, pseudohyphae, conidiophores, conidia, poroconidia, arthroconidia and sporangiospores) were examined under Nikon ECLIPSE Ci microscope for identification of isolates following the taxonomic keys for different *taxa* described by Gilman [36], Alexopoulos and Mims [37].

The modified CTAB (cetyl trimethyl ammonium bromide) method was used to extract fungal DNA from mycelia of pure cultures [38]. A total of 500 mg of fungal mycelia was added to a 2.0 mL microcentrifuge tube with 0.2 g zirconia beads, then 100 μL of sterile lysis buffer (1 M Tris-HCl (pH 8.0) 10 mL, 0.5 M EDTA (pH 8.0) 4 mL, 5 M NaCl 28 mL, CTAB 2 g, add ddH_2_O to 100 mL) was added. The mixture was homogenized for 30 s, 60 Hz, 2 cycles using a high-throughput tissue grinder (SCIENTZ-48, SCIENTZ, Ningbo, China). 400 μL lysis buffer was added after homogenization, then the mixture was incubated at 60 °C for 30–60 min in a water basin by inverting the tubes at intervals of 15 min. The tubes were added with 500 μL of SEVAG (chloroform:isoamyl alcohol, 24:1), mixed evenly, then centrifuged at 12,000 rpm for 15 min. The supernatant was transferred to a new clean 2.0 mL tube, and an equal volume of SEVAG was added. The resulting solution was mixed evenly and centrifuged at 12,000 rpm for 15 min. The last step was repeated twice, then the supernatant (225–300 μL) was taken and transferred to a new clean 1.5 mL microcentrifuge tube, 600 μL isopropyl alcohol were added and the tubes inverted gently several times, thus making DNA “ropes” precipitate visible. The tubes were put at −20 °C for 1 h and then centrifuged at 12,000 rpm for 5 min. Supernatant was removed carefully from each tube and the pellets were washed with 400 μL of 70% ethanol, then centrifuged at 12,000 rpm for 3 min. After repeating the last step twice, the aqueous phase was discarded and the tubes placed in super clean bench or fume cupboard to dry. The DNA was redissolved in 50 μL of sterile ddH_2_O for further study. For bacteria, DNA was obtained by boiling method [39]. Bacterial samples were put into 100 μL sterile ddH_2_O in 1.5 mL microcentrifuge tubes and heated at 100 °C boiling water for 15 min, DNA was dissolved in the liquid phase. This step was followed by centrifugation (14,000 rpm/1 min) to recover supernatant, then the tubes containing supernatant were stored at −20 °C.

The internal transcribed spacer (ITS) region of fungal rDNA was amplified using primers ITS1 and ITS4, whereas the V3 to V4 hypervariable region of bacterial 16S rRNA was amplified using the primers U341F and U806R (Table 2). PCR amplifications were performed in 25 mL reaction mixtures that were prepared with 1 μL of genomic DNA as template DNA, 12.5 mL of 2× Vazyme Rapid Taq Mater Mix, 1 μL of each primer (10 μM), and double-distilled H_2_O to a total volume of 25 μL, using the following reaction conditions: Temperature of 95 °C for 3 min, 30 cycles at 95 °C for 15 s, 55 °C for 15 s, 72 °C for 15 s, and a final extension at 72 °C for 5 min. PCR products were Sanger sequenced at Tianjin Tsingke Biological Technology Co., Ltd. (Tianjin, China). Sequences were assembled using BioEdit [40]. All obtained sequences were BLASTn searched in NCBI and assigned to potential genera and species. The nomenclature followed Index Fungorum (indexfungorum.org) [41]. Sequences were deposited in GenBank under the accession numbers MW582315–MW582428 (fungi) and MW577449–MW577451 (bacteria).

All fungal colonies isolated in this study were inoculated, both on slanted medium in glass tubes and in strain preservation tubes containing double-sterilized ultra-pure water, and stored at 4 °C for long term preservation. Bacterial strains were preserved in 30% glycerol at –80 °C. All strains were deposited in the LP Culture Collection (personal culture collection held in the laboratory of Prof. Lorenzo Pecoraro), at the School of Pharmaceutical Science and Technology, Tianjin University, Tianjin, China.

### 2.3. Assessment of Microbial Community Using Illumina Sequencing

The total genomic DNA was extracted from 0.5 g of soil stone mixture for each sample by using a FastDNA^®^ Spin Kit for soil (MP Biomedicals, Solon, OH, USA) according to the manufacturer’s instructions. The products were checked in 1% agarose gel and quantified with NanoDrop 2000 UV-vis spectrophotometer (Thermo Scientific, Waltham, MA, USA). The fungal ITS2 rDNA region was amplified using the fungus-specific primer pair ITS3F (5′-GCATCGATGAAGAACGCAGC-3′)–ITS4R (5′-TCCTCCGCTTATTGATATGC-3′). The V3–V4 hypervariable region of the bacterial 16S rRNA gene was amplified using the primers 338F (5′-ACTCCTACGGGAGGCAGCAG-3′) and 806R (5′-GGACTACHVGGGTWTCTA AT-3′). PCR reactions were carried out in triplicate 20 μL mixture containing 4 μL of 5× TransStart FastPfu Buffer, 2 μL of dNTPs (2.5 mM), 0.8 μL of each primer (5 μM), 0.4 μL of TransStart FastPfu Polymerase, 0.2 μL of bovine serum albumin (Takara Bio Inc., Kusatsu, Shiga, Japan) and 10 ng of template DNA. The PCR reaction conditions were as follows: an initial denaturation of 95 °C for 3 min, followed by 35 cycles (for the amplification of fungal ITS region, for bacteria was 27 cycles) of denaturation at 95 °C for 30 s, annealing at 55 °C for 30 s and extension at 72 °C for 45 s, and a final extension at 72 °C for 10 min. PCR products were checked in 2% agarose gel and purified with the AxyPrep DNA Gel Extraction Kit (Axygen Biosciences, Union City, CA, USA) and quantified using QuantiFluor-ST Fluorometer (Promega, Madison, WI, USA). Amplicon libraries were generated using the NEXTFLEX^®^ Rapid DNA-Seq Kit (Bioo Scientific, Austin, TX, USA) following the manufacturer’s recommendations to add index codes. The library quality was assessed by using the QuantiFluor^TM^-ST Blue fluorescence quantitative system (Promega) according to the manual. Equimolar amounts of purified amplicons from different samples were pooled and paired-end sequenced (2 × 300 bases) on an Illumina MiSeq PE300 platform (Illumina Inc., San Diego, CA, USA) at Shanghai Majorbio Bio-Pharm Technology Co., Ltd. (Shanghai, China). The MiSeq raw sequencing fastq files were deposited in the Sequence Read Archive (SRA) at the National Center for Biotechnology Information (NCBI) as BioProject ID PRJNA700061.

### 2.4. Bioinformatics of Fungal and Bacterial Sequences

Raw sequences were quality-filtered by Fastp v0.19.6 [42] according to the following rules: (1) Filter the bases in the tail of the reads with quality score below 20; (2) set 50 bp sliding windows on the reads, cut off the back-end bases from the window if the average quality value in the window is lower than 20, and the reads below 50 bp after quality control were removed; (3) reads containing ambiguous nucleotide (N) were eliminated; (4) the barcode mismatches were not allowed and the maximum number of primer mismatches was 2. Paired-end reads were subsequently merged by FLASH v1.2.11 [43] with a minimum overlap of 10 bp and maximum allowable mismatch ratio 0.2. Quality-controlled sequences were clustered in operational taxonomic units (OTUs) at a 97% similarity threshold using UPARSE v7.1 [44]. Chimeric sequences were identified and removed using UCHIME [45]. The sequence with the highest abundance were selected as representative sequence for each OTU. Taxonomic assignments of the representative sequence of each OTU were based on RDP Classifier v2.2 [46] using the UNITE Database v8.0 (for fungal sequences) [47], and SILVA 16S rRNA Database (release 138, for bacterial sequences) [48,49]. Volcanic fungal and bacterial OTU table were rarefied to counts up to 43,002 and 36,117 reads per sample, respectively, (these were the lowest sequence depths obtained from all samples) and used for downstream analyses, including calculation of relative abundance of different taxonomic groups, α-diversity and β-diversity analyses, as well as network analyses.

### 2.5. Statistical Analysis

α-diversity indexes, including richness (number of OTUs), Shannon, Chao1, and Good’s Coverage were calculated in Mothur used to estimate the richness, diversity and coverage of microbial communities. Rarefaction curves [50] and Rank abundance curve [51] were drawn in R package for evaluation of sufficient sequence depth, as well as assessment of community richness and evenness. Between-groups Venn diagram was plotted using R to identify unique and common OTUs. Principal co-ordinate analysis (PCoA) and Analysis of Similarity (ANOSIM) with 999 permutations were performed for the assessment of dissimilarities between samples based on taxonomic Bray–Curtis [52], phylogenetic Weighted and Unweighted Unifrac distance matrices [53].

### 2.6. Network Analysis

The interactions between microbial taxa were analyzed through a network structure to assess the complexity of fungal and bacterial communities in Tianchi crater. Rarefied fungal and bacterial OTU tables were used for network analyses of crater soils. Only the OTUs in the top 100 abundance level were retained for the analysis to reduce the complexity of the data sets. Spearman’s rank correlation coefficients (r) between the OTUs with a magnitude ≥0.8 or ≤−0.8 and statistically significant (*p* < 0.05) were further included for network construction. Gephi (version 0.9.2) was used for the topological properties estimation, visualization and modular analysis of the network [54]. The nodes in the constructed network represent the OTUs indicating different *taxa*, whereas the edges correspond to significant positive or negative correlations between *taxa*.

## 3. Results

### 3.1. Diversity of Culturable Microbes

In total, 117 fungal strains were isolated from the 12 soil samples collected along the margin of Tianchi crater (Table S1). The isolated fungi belonged to 51 species in 31 genera, based on combined ITS sequencing and microscopic identification (Table 3). Among them, 92.16% (i.e., 47 species, 93 strains) belonged to 27 genera of Ascomycota, 5.88% (3 species, 6 strains) belonged to three genera of Basidiomycota, while Mucoromycota was represented by a single species, *Mucor hiemalis*, which yielded 18 strains (Figure S1, Table S2). The most common genera included *Penicillium* (9.80%), *Trichoderma* (9.80%), *Cladosporium* (7.84%), *Didymella* (7.84%), *Alternaria* (5.88%) and *Fusarium* (5.88%) (Figure S2, Table S2). The most common species was *Mucor hiemalis* (15.38%), followed by *Fusarium tricinctum* (9.40%) and *Cladosporium cladosporioides* (6.84%). Three resistant bacterial strains were also isolated in the media supplemented with antibiotics, and were identified as *Rhodococcus degradans*, *R. qingshengii* and *Chryseobacterium lactis* (Table S1).

### 3.2. Microbial Diversity by Illumina Sequencing

From metabarcoding analyses, a total of 4,371,622 raw reads with 772,186 effective sequences were obtained by fungal ITS2 gene sequencing, using the Illumina MiSeq PE300 platform. The number of sequences from the samples ranged from 43,002 to 70,583. After normalization of sequences by resampling 43,002 reads every sample, 1254 OTUs could be classified into the fungal kingdom (Table S3). From bacterial 16S V3–V4 region sequencing, 3,105,944 raw reads with 507,467 effective sequences were obtained. 2988 bacterial OTUs were clustered subsequently after the OTU table was rarefied to counts up to 36,117 reads per sample (the number of sequences ranged from 36,117 to 49,230) (Table S4).

**Figure 2:**
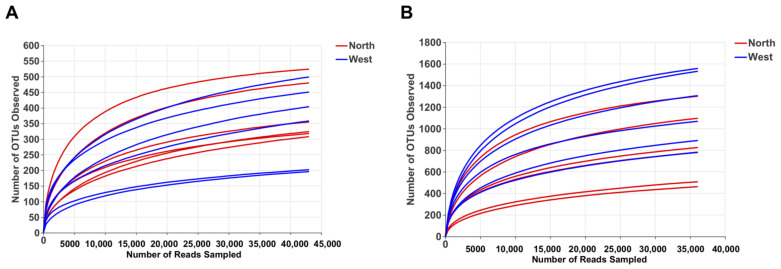
Rarefaction curve of fungal OTUs (**A**) and bacterial OTUs (**B**) for α-diversity analyses.

**Figure 3:**
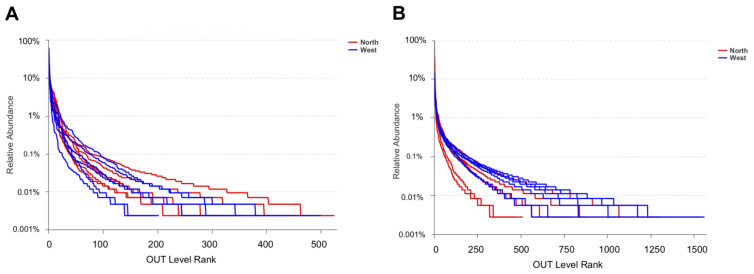
Rank abundance curve of fungal OTUs (**A**) and bacterial OTUs (**B**) for α-diversity analyses.

OTU richness, Shannon and Chao1 indexes were calculated for both fungal and bacterial communities (Table 4). Tianchi crater margin soil contained more diverse and richer bacterial than fungal communities as shown by the significantly higher α-diversity indexes measured according to Mean ± SD values (Table 4). Good’s coverage for all samples was > 99.0% (Table 4), which indicated the small probability that sequences were not detected in each sample. This result was consistent with the rarefaction curves related to the OTUs that showed all the curves approaching saturation plateau (Figure 2). The rank abundance curve representing richness and evenness of fungal and bacterial communities showed that the highest richness and evenness was found from northern soil samples, for fungi, while from western samples, for bacteria (Figure 3).

From the rarefied fungal OTUs table, 1077 out of the 1254 retrieved OTUs were identified into eight phyla, 38 classes, 112 orders, 250 families and 455 genera (Table S3), whereas 165 OTUs remained unidentified and 12 *Incertae sedis* at phylum level (14.11%). Among the 1077 identified fungal OTUs, 804 were assigned to Ascomycota, 213 to Basidiomycota, 32 Chytridiomycota and 16 Mortierellomycota. Other phyla, including Monoblepharomycota, Glomeromycota, Rozellomycota and Mucoromycota were recovered in small proportions (12 OTUs) (Table S3). For the bacterial community, except 52 OTUs that remained unidentified at phylum level (1.74%), 2936 OTUs were assigned to 32 phyla, 91 classes, 217 orders, 353 families and 654 genera (Table S4). The phyla Proteobacteria (557OTUs), Chloroflexi (375 OTUs), Actinobacteriota (334 OTUs), Bacteroidota (314 OTUs), Acidobacteriota (217 OTUs), Myxococcota (184 OTUs), Patescibacteria (158 OTUs), Bdellovibrionota (151 OTUs), Gemmatimonadota (112 OTUs) and Planctomycetota (106 OTUs) were the most dominant in the crater soil (Table S4).

**Figure 4:**
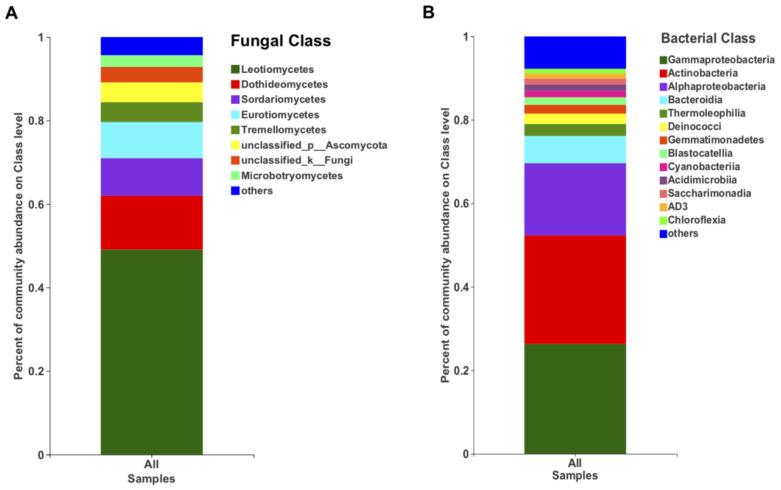
The relative abundance of fungi (**A**) and bacteria (**B**) at class level.

**Figure 5:**
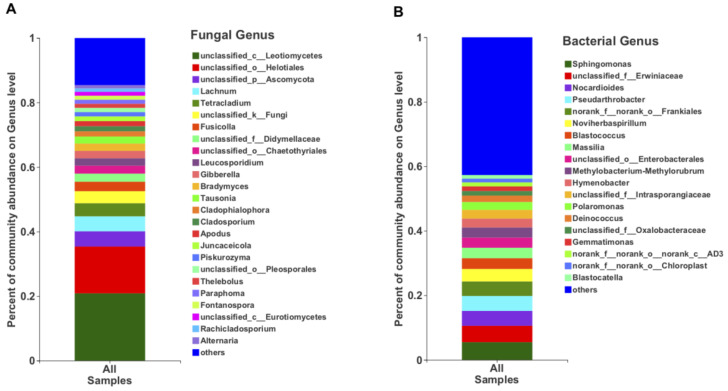
The relative abundance of fungi (**A**) and bacteria (**B**) at genus level.

In terms of the relative abundance, the class Leotiomycetes (49.13%), followed by Dothieomycetes (12.90%), dominated the Tianchi crater soil fungal community (Figure 4A), while for the bacterial community, Gammaproteobacteria (26.30%), Actinobacteria (26.04%) and Alphaproteobacteria (17.29%) were dominant (Figure 4B). At genus level, the relative abundance of *Leucosporidium* was found to be the highest (2.30%) among Basidiomycota, while the most abundant ascomycetes genera remained not clearly discriminated as belonging to the class Leotiomycetes (Figure 5A). The genera from other phyla were with very low relative abundances (<1%) and merged as “others” in the bar plot (Figure 5A). Among bacteria, the genus *Sphingomonas* (5.50%) was the most abundant in the analyzed crater soil (Figure 5B).

**Figure 6:**
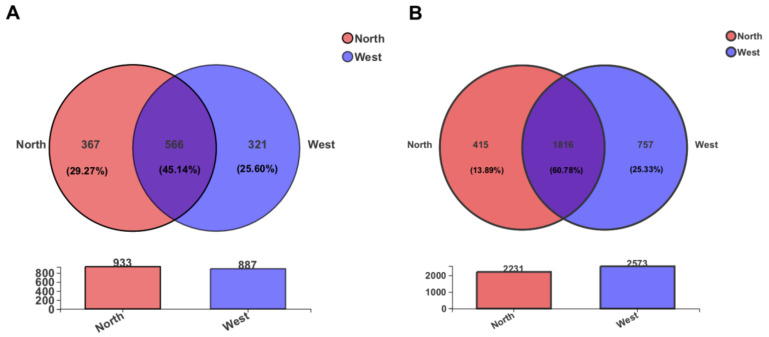
The Venn diagram displaying the distribution of shared and unique OTUs of the fungal community (**A**) and bacterial community (**B**) in two sides of the crater. The number of OTUs in each side is indicated in the respective circle.

From a comparison of OTUs diversity between northern and western crater soil samples, a total of 566 fungal and 1816 bacterial OTUs, accounting for 45.14% and 60.78% of the total OTUs, respectively, were found to be common to both sides of the crater (Figure 6). Some of the common OTUs exhibited a relatively high abundance among all OTUs. For instance, fungal OTU1055 (unclassified Leotiomycetes) and OTU1230 (unclassified Helotiales) were the most abundant OTUs detected, showing a relative abundance of 11.89% and 11.87%, respectively. Similarly, common bacterial OTU11 (unclassified Erwiniaceae), with relative abundance 5.07%, and OTU541 (unclassified *Pseudarthrobacter*, 4.62%) dominated the bacterial community.

**Figure 7:**
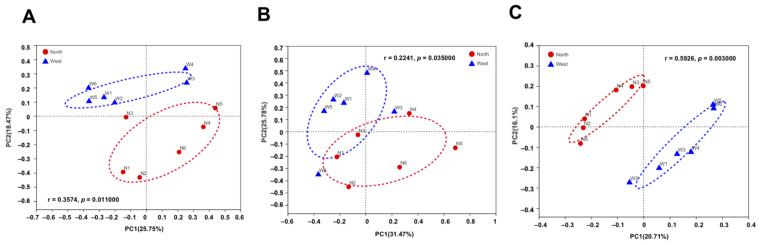
Principal co-ordinate analysis (PCoA) plots of fungal communities in northern and western crater soil based on Bray–Curtis distance (**A**), weighted Unifrac distance (**B**) and unweighted Unifrac distance (**C**). The r and *p*-values of analysis of similarity (ANOSIM) were shown respectively in each figure (*p* < 0.05).

**Figure 8:**
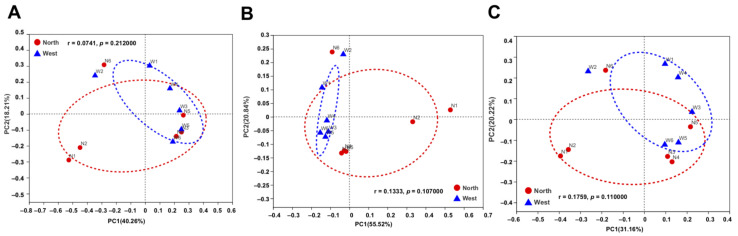
Principal co-ordinate analysis (PCoA) plots of bacterial communities in northern and western crater soil based on Bray–Curtis distance (**A**), weighted Unifrac distance (**B**) and unweighted Unifrac distance (**C**). The r and *p*-values of analysis of similarity (ANOSIM) were shown respectively in each figure (*p* > 0.05).

The variations of fungal and bacterial OTUs were analyzed by principal co-ordinate analysis (PCoA). Fungal communities in northern and western crater soil were clearly distinguished based on Bray–Curtis distance (ANOSIM *p* = 0.011) (Figure 7A), weighted Unifrac distance (ANOSIM *p* = 0.035) and unweighted Unifrac distance (ANOSIM *p* = 0.003) (Figure 7B,C). The separation of communities was more significant using unweighted Unifrac distance, which is more sensitive to *taxa* presence or absence regardless of their abundances compared with Bray–Curtis distance and weighted Unifrac distance. For bacterial communities, on the contrary, no clear separation was found based on the different dissimilarity matrices (Figure 8).

### 3.3. Network Complexity for Bacterial and Fungal Communities

**Figure 9:**
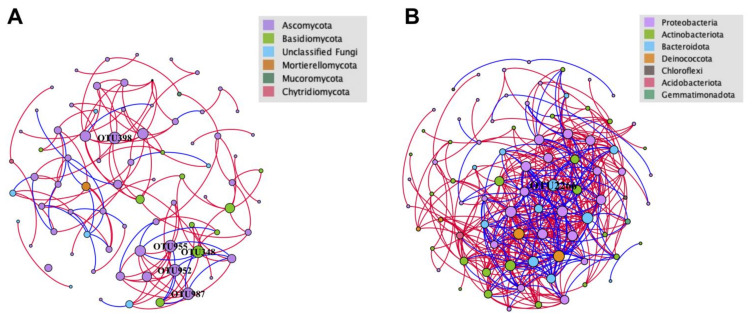
Soil OTUs network analysis in the Tianchi crater soil (Fruchterman Reingold layout). (**A**) Network of fungal community; (**B**) network of bacterial community. Each node represents an OTU indicating an individual species. Color codes for nodes belonging to different dominant phyla. The node size is proportional to the degree (degree = number of direct correlations to a node). Positive interactions are displayed as red edges and negative interactions are displayed as blue edges.

Separated co-occurrence network analyses were performed for both fungal and bacterial communities to explore and evaluate the complex interactions between the microbial *taxa* detected in the analyzed crater soils. All interactions comprised in the network were strongly correlated and statistically significant (r ≥ 0.8 or ≤ −0.8 and *p* < 0.05). In general, there were considerable differences in network topology and structure between fungal and bacterial communities (Figure 9, Table 5). The more compact and complicated bacterial network than the fungal network was reflected by the average path lengths (2.661 vs. 4.982) and average degrees (10.143 vs. 3.487) (Figure 9, Table 5). Contrasting correlations between OTUs within fungal and bacterial communities were observed. Seventy-seven nodes were included in the network of the fungal community, with 136 edges, 108 positive interactions and 28 negative interactions, while 84 nodes were included in the bacterial network. The network metrics of fungal community were significantly lower than those for bacterial community (426 edges, 302 positive and 124 negative interactions) (Figure 9, Table 5). Both networks were predominated by positive interactions (79.41% and 70.89%), indicating that the mutual effects dominate the microbial communities in the crater. For fungal community, *Leucosporidium* sp. (OTU348), *Paraphoma fimeti* (OTU398), *Alatospora* sp. (OTU952), unclassified Chaetothyriales (OTU955) and unclassified Eurotiomycetes (OTU987) were found to have the most abundant interactions with other nodes (degree: 9) (Figure 9A). *Pedobacter* sp. (OTU2266) was found to have the most abundant interactions in the network of bacterial community (degree = 26) (Figure 9B).

## 4. Discussion

In this study, we provided a comprehensive characterization of fungal and bacterial communities along the crater margin of Tianchi Volcano, in Changbai Mountain Nature Reserve, by combing molecular and morphological analyses of culturable microbes, and metabarcoding analyses based on Illumina sequencing. A few previous studies have been conducted on microbial diversity in relatively lower altitudes of Changbai Mountain northern slope, mostly using high-throughput sequencing methods alone [14,33,55]. To the best of our knowledge, this study represents the first microbial community analysis in different sides of Tianchi Volcano crater margin, using integrated culture-dependent and metabarcoding approaches.

Many microbial species isolated from this study have been frequently reported from high-altitude or cold environments, and have been extensively applied in biotechnological fields, showing particular research significance due to their peculiar ecology. For instance, *Aureobasidium* strains isolated from Tianchi northern crater sediments, highly matched with *A. pullulans* isolated from saxicolous lichens growing in the north side of Taibai Mountain in China, at the altitude of 2614 m a.s.l. [56], and *A. pullulans* found from the glacier surface snow of Tibetan Plateau in China (>99% similarity) by Shao [57]. Besides, *A. pullulans* have been previously isolated from high Italian glaciers [7], high Arctic glaciers [58] and Antarctica soils [59]. It has been well documented that *A. pullulans* is a psychrophilic fungus able to produce various useful bioproducts (enzymes, pullulan, single-cell proteins and siderophore) for waste treatments, chemical industry materials, food industries and biocontrol [59]. Our finding of *Candida tropicalis* in the western crater of Tianchi Volcano is in agreement with previous studies in which *Candida* is regarded as a common Antarctic yeast genus [8]. *Candida tropicalis* has been described capable of degrading high-concentration phenol [60], and tolerant to copper [61]. This species may play an important functional role in the studied crater area of Tianchi volcano characterized by high heavy-metal concentration. The ascomycete *Epicoccum nigrum*, isolated from both sides of the analyzed crater, has been described as a xerotolerant species in a study conducted in Annapurna Mountains of Nepal [62]. This fungal species has been found to produce antibacterial compounds [3] and described as a biocontrol agent [63]. Among the few bacterial isolates belonging to *Chryseobacterium* and *Rhodococcus* genera, *Chryseobacterium* strain showed high similarity with *C. lactis* (accession number = MT065804) isolated from Qinling high altitude area in China by Men X. in an unpublished study, while *Rhodococcus* strains highly matched with *R. degradans* (accession number: LR216744) isolated from sodic soil in Hungary by Krett G., and *R. qingshengii* (accession number: MT632489) isolated from oil well in Russia by Borzenkov et al., in unpublished studies. Species from *Rhodococcus* have been previously found to be psychrotolerant in a study conducted in Antarctica [64]. This considerable number of microbes isolated from Tianchi Volcano, which have previously shown preference for high-altitude, cold, and geothermal extreme environments, and have been described as psychrotolerant and metal-tolerant microorganisms, provide new insights in the characterization of the ecological features of the investigated environment, and may represent a precious source for the isolation of new bioactive compounds [65,66].

The use of molecular and high-sensitive metabarcoding methods allowed the detection and identification of both common and rare microbial *taxa*, thus providing detailed and accurate information on fungal and bacterial communities in the investigated high-altitude volcanic habitat. The high relative abundances of unclassified genera detected in this study may indicate the presence of a significant number of undiscovered and possibly endemic *taxa* in the analyzed communities. Recent studies have described the fungal diversity in apparently barren (plant-free) zones of high alpine environments, including the Himalayas (Nepal, 5146 and 5509 m a.s.l.) [67], Colorado (USA, 3660–3800 m a.s.l.) [68,69], Llullaillaco (6030 and 6330 m a.s.l.) [8] and Socompa (5235 m a.s.l.) volcanoes [70] in the Andes, along the Chilean-Argentinian border. A major group of basidiomycetes found from these barren high-altitude soils (mostly Colorado) was constituted by members of the Microbotryomycetes, including *Leucosporidium antarcticum*. Accordingly, our results revealed that *Leucosporidium* was the most abundant genus in Basidiomycota from high-throughput sequencing, and two sequences obtained from isolated strains showed 100% similarity with *L. creatinivorum* isolated from the soil of King George Island, in the sub-Antarctic region. *Leucosporidium creatinivorum* is known as a psychrotolerant yeast, which has been frequently isolated from cold extreme environments, such as glaciers in Argentina, Russia, Iceland and Italian Alps [71,72], and has been also reported from soil, marine sponges and lichens in Antarctica [73,74,75]. Members of *Leucosporidium* have been described as sources of cold-active enzymes [73,76,77] and anti-freeze proteins [78], and have been shown to possess phenol and phenol-related compounds degradation ability [79]. The high relative abundance of *Leucosporidium* in high-altitude volcanic soil stone mixtures of Tianchi volcano confirmed the peculiar preference of this genus for extreme cold environments. Up to now, to our knowledge, the existence of *Leucosporidium* in China was only detected by metagenomic analysis [80,81]. Our results represent the first isolation of *L. creatinivorum* in China. These isolated strains certainly deserve further attention as potential producers of metabolites for biotechnological application. Besides, in our study, *Leucosporidium* was also found to have the most complicated interactions in the fungal network, which may indicate a fundamental ecological role of this *taxon* in maintaining the stability of the fungal community in high elevation volcanic environments.

The bacterial community was dominated by Proteobacteria, result in agreement with previous studies where this taxon has been described as the most dominant soil bacterial phylotype in different continents across the globe [82]. Gammaproteobacteria was the most abundant class within Proteobacteria, followed by Alphaproteobacteria, which is consistent with previous findings from Deception Island Volcano in Antarctica [83]. *Sphingomonas*, the most abundant bacterial genus in Tianchi Volcano, has been previously reported from other high-altitude volcanic areas, including the active volcano Socompa in Argentina [84], and El Chichón volcano in Mexico [85]. Members of this genus play prominent functional roles in the remediation of contaminated environments. For instance, several *Sphingomonas* species have been extensively associated to the chelation of heavy metals, and degradation/bio-transformation of aromatic hydrocarbons [86]. *Sphingomonas* strains have been also found to produce highly beneficial phytohormones [86]. The dominant presence of *Sphingomonas* in high-altitude, metal-enriched, and carbon poor sediments of Tianchi crater, suggested an important contribution of this bacterial group to the functionality of the studied ecosystem, and confirmed the metal-tolerant ability of these microorganisms, which probably possess special nutritional strategies to adapt and survive in barren habitats, under extreme conditions. Further studies focusing on the metabolic activity and nutritional mode of *Sphingomonas* species may clarify the mechanisms allowing extremotolerant microbes to strive in environments which are unsuitable for the majority of life forms.

The significant clear variation of soil fungal communities in the two sides of the Tianchi crater suggested that geographical and environmental factors may have a stronger influence on the diversity of fungi than bacteria, which, instead, did not show any remarkable community difference between North and West sampling areas. Different community diversity patterns may be the result of mixed effects of multiple factors, such as solar radiation, temperature, and soil properties that may be more relevant to fungi than bacteria [87]. However, our analysis of samples pH showed highly homogeneous values, with average pH at 7.76, which could not provide useful information to explain the observed community diversity variation. Further studies are needed in order to analyze environmental factors affecting bacterial and fungal community diversity in high elevation volcanic extreme environments. The results of PCoA were consistent with co-occurrence network analyses. Compare to the fungal network, the bacterial network was composed by more complicated and closer associations, in terms of numbers of nodes, edges, average degree and average path length, thus indicating that the bacterial communities possess rapid responsiveness to perturbation [88], and much stronger resistance to environmental changes than fungal communities. However, these related environmental factors remain to be studied.

The detailed characterization of Tianchi Volcano microbial diversity provided in this study may constitute an important reference for future long-term monitoring, aiming at tracing the effects of global warming on this delicate environment. Indeed, previous studies have proved that increasing global average temperatures can drive successions of microbial communities, for instance by changing the quality and quantity of genes potentially available for horizontal gene transfer, and can lead to increasingly divergent succession, with possibly higher impact on fungi than bacteria [89,90]. Besides, the impact of climate changes is predicted to be very pronounced at high-elevations and in tundra ecosystems [91,92]. The area along the margin of the Tianchi crater represents a typical example of a high-altitude tundra ecosystem in China. It has been reported that the mean annual temperature in Changbai Mountain Nature Reserve increased at a rate of 0.392 °C/10 years from 1958 to 2015, which is significantly higher than the national warming rate (0.22 °C/10 years) [93]. Because global warming may particularly alter the biogeochemistry and ecology of cold soil ecosystems [94,95,96], the increasing warming registered in Changbai Mountain region deserves attention, and further studies are needed to verify the response to environmental changes of the particularly sensitive and fragile high-altitude tundra microbes unveiled from our study in Tianchi volcano.

## 5. Conclusions

Our results represent an unprecedent comprehensive microbial community analysis along the high-altitude crater margin of Tianchi Volcano, by combing culture-dependent and metabarcoding analyses. We observed that Tianchi Volcano hosted a combination of taxonomic groups characteristic of high-altitude, cold and geothermal environments, with a considerable number of isolated microbes being of particular research significance, due to their rarity and peculiar ecology. Our study suggested that the structure and diversity of fungal community was more sensitive to environmental and geographical changes compare to bacterial community in the analyzed area. Our findings may represent an important starting point for future studies to explore the precious metabolite resources of the isolated microbes, and elucidate the effect of different environmental factors on community structure and dynamics in high-altitude volcanic environments.

## Figures and Tables

**Table 1:** Details of sampling sites.

Sampling Sites	Latitude	Longitude	Altitude (m a.s.l)	Sample Type
Northern margin of Tianchi crater	42°1′33″ N	128°4′8″ E	2620	6 Soil stone mixture samples
Western margin of Tianchi crater	41°59′48″ N	128°1′44″ E	2460	6 Soil stone mixture samples

**Table 2:** Primers used for culturable microbe identification.

Microbes	Locus	Primer Name	Direction	Sequence	Target Region
Fungi	Internal transcribed spacer (ITS)	ITS1	Forward	5′-TCCGTAGGTGAACCTGCGG-3′	ITS
		ITS4	Reverse	5′-TCCTCCGCTTATTGATATGC-3′	
Bacteria	V3 to V4 hypervariable region	U341F	Forward	5′-ACTCCTACGGGAGGCAGCAG-3′	16S rRNA
		U806R	Reverse	5′-GGACTACHVGGGTWTCTAAT-3′	

**Table 3:** Taxonomic groups of fungi isolated from Tianchi Crater of Changbai Mountain.

Phylum	Class	Order	Genus (Number of Species)
**Ascomycota**	Dothideomycetes	Capnodiales	*Cladosporium* (4)
		Coniosporiales	*Coniosporium* (1)
		Dothideales	*Aureobasidium* (1)
		Pleosporales	*Alternaria* (3), *Curvularia* (1), *Didymella* (4), *Epicoccum* (1), *Juxtiphoma* (1), *Leptosphaeria* (1), *Paraphaeosphaeria* (1), *Paraphoma* (1), *Phaeosphaeria* (2), *Phoma* (2)
	Eurotiomycetes	Chaetothyriales	*Exophiala* (1)
		Eurotiales	*Byssochlamys* (1), *Penicillium* (5)
	Incertae sedis	Incertae sedis	*Tricellula* (1)
	Leotiomycetes	Helotiales	*Xenochalara* (1)
		Helotiales	*Helotiales* sp. (1)
	Saccharomycetes	Saccharomycetales	*Candida* (1)
	Sordariomycetes	Amphisphaeriales	*Microdochium* (1)
		Hypocreales	*Fusarium* (3), *Nectria* (1), *Tangerinosporium* (1), *Trichoderma* (5)
		Microascales	*Cephalotrichum* (1)
		Xylariales	*Xylaria* (1)
**Basidiomycota**	Microbotryomycetes	Leucosporidiales	*Leucosporidium* (1)
	Tremellomycetes	Cystofilobasidiales	*Tausonia* (1)
		Tremellales	*Naganishia* (1)
**Mucoromycota**	Mucoromycetes	Mucorales	*Mucor* (1)

**Table 4:** α-diversity indexes (OTU richness, Shannon, Chao1 and Coverage) data of fungal and bacterial communities from crater soil samples from North (N) and West (W) slopes. OTU = Operational Taxonomic Unit.

Sample	Fungal Community				Bacterial Community			
	OTU Numbers	Shannon	Chao	Coverage (%)	OTU Numbers	Shannon	Chao	Coverage (%)
Mean ± SD	368 ± 108	2.95 ± 0.74	446.14 ± 108	99.8 ± 0.1	1009 ± 365	4.72 ± 0.93	1240.98 ± 393.60	99.3 ± 0.2
N1	324	2.36	395.67	99.8	462	2.59	607.01	99.6
N2	480	3.45	526.36	99.8	508	3.38	707.94	99.5
N3	308	2.44	426.32	99.8	825	4.78	1061.35	99.4
N4	355	3.35	412.37	99.8	782	4.62	1049.14	99.4
N5	318	3.42	397.44	99.8	1096	4.84	1297.27	99.3
N6	524	4.04	555.52	99.9	1301	5.58	1481.06	99.3
W1	499	3.20	588.25	99.7	1558	5.75	1828.56	99.1
W2	404	3.01	496.64	99.7	1068	5.22	1326.74	99.3
W3	451	3.86	554.26	99.7	1306	5.44	1569.18	99.2
W4	358	2.34	477.28	99.7	1532	5.47	1840.95	99.0
W5	196	1.54	255.11	99.9	890	4.49	1101.68	99.3
W6	202	2.39	268.5	99.9	780	4.52	1020.9	99.4

**Table 5:** Network topological properties of fungal and bacterial communities in the crater soil of the Tianchi Volcano.

Network Metrics	Community	
	Fungi	Bacteria
Number of nodes	77	84
Total number of edges	136	426
Number and percentage of positive correlations	108 (79.41%)	302 (70.89%)
Number and percentage of negative correlations	28 (20.59%)	124 (29.11%)
Average degree	3.487	10.143
Network diameter	12	7
Modularity	0.709	0.341
Number of communities	14	10
Average clustering coefficient	0.521	0.565
Average path length	4.982	2.661

## Data Availability

The Fungal and bacterial DNA sequences amplified during this study are available in GenBank under accessions MW582315–MW582428 (fungi) and MW577449–MW577451 (bacteria), and in the Sequences Read Archive of NCBI as BioProject ID PRJNA700061.

## Supplementary Materials

The following are available online at https://www.mdpi.com/article/10.3390/life11040280/s1, Figure S1: Number of fungal genera, species and strains in different phyla obtained from Tianchi crater soil. Figure S2: Number of fungal species and strains from most abundant genera observed in Tianchi crater soil. Table S1: Fungal and bacterial diversity molecularly detected in Tianchi Volcano soil, from DNA extracted from isolated microbes. Table S2: An overview of fungal strains isolated from the crater soil samples. Table S3: Rarefied fungal OTUs table based on fungal ITS2 rDNA region Illumina sequencing. Table S4: Rarefied bacterial OTUs table based on V3–V4 hypervariable region of the bacterial 16S rRNA gene Illumina sequencing.
