# Supplementary material for: Analysis of Soil Fungal and Bacterial Communities in Tianchi Volcano Crater, Northeast China
Source: Life (Basel). 2021 Mar 26;11(4):280. doi: 10.3390/life11040280 (PMC8066613; doi:10.3390/life11040280)
Supplement: Supplementary file 1 [file life-11-00280-s001.pdf]

Supplementary information

Analysis of soil fungal and bacterial communities in Tianchi Volcano crater, northeast China

Xiao Wang[1] and Lorenzo Pecoraro[1]*

[1]School of Pharmaceutical Science and Technology, Tianjin University, 92 Weijin Road, Nankai District, 300072 Tianjin, China; wang_xiao1996@163.com (X.W.); lorenzo.pecoraro@tju.edu.cn (L.P.).

*Correspondence: Lorenzo Pecoraro (telephone: +86 18520824550, e-mail: lorenzo.pecoraro@tju.edu.cn) School of Pharmaceutical Science and Technology, Tianjin University, 92 Weijin Road, Nankai District, Tianjin 300072, China.

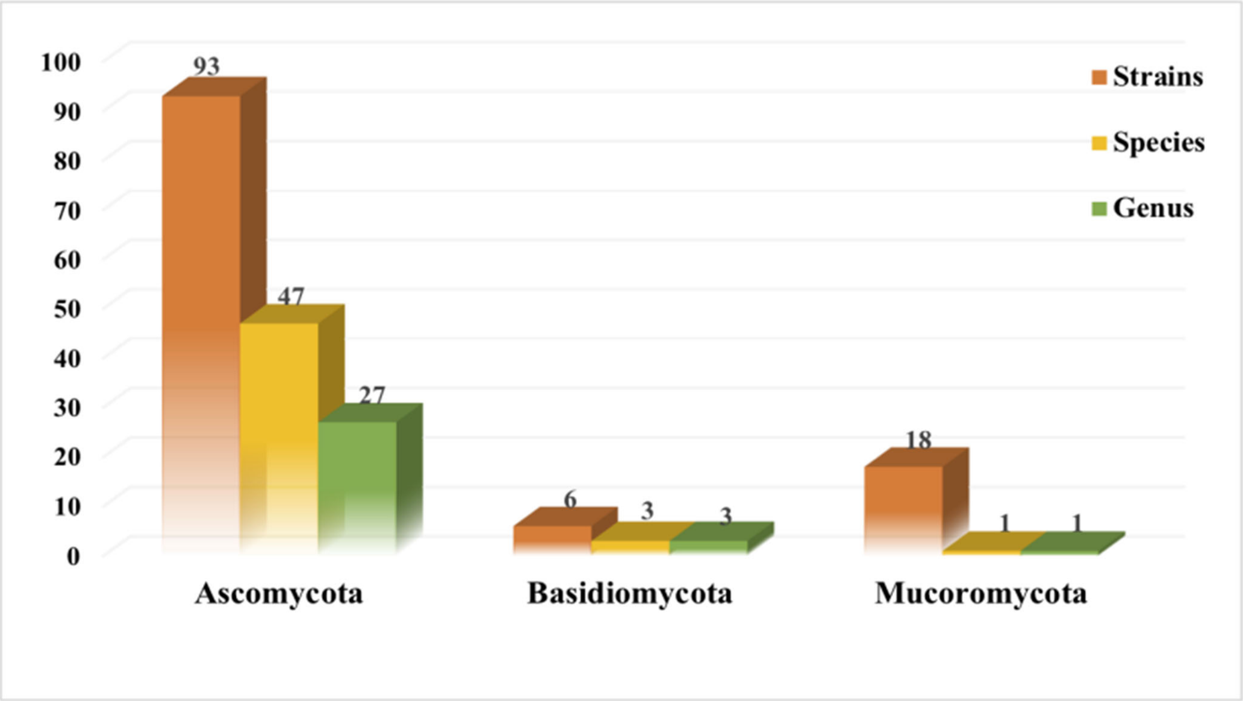

Figure S1. Number of fungal genera, species and strains in different phyla obtained from Tianchi crater soil.

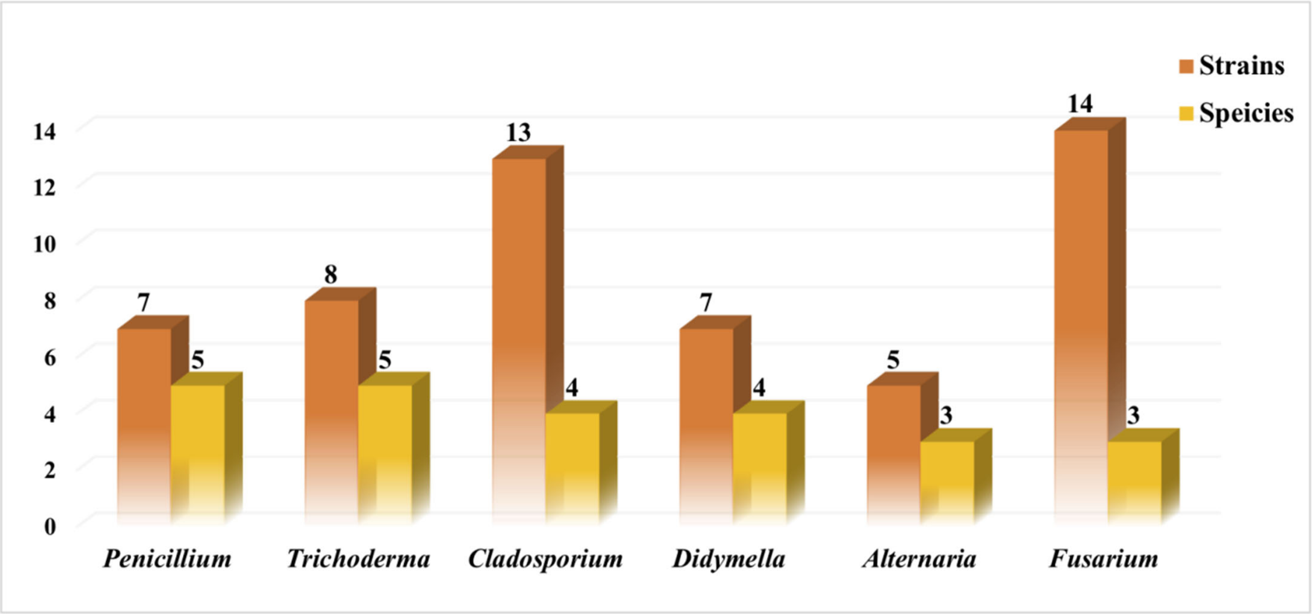

Figure S2. Number of fungal species and strains from most abundant genera observed in Tianchi crater soil.

Table S1. Fungal and bacterial diversity molecularly detected in Tianchi Volcano soil, from DNA extracted from isolated microbes.

Strain	GenBank code	Best BLAST matches	Accession code	Overlap length	% match
**Fungi**					
N-I-1	MW582315	*Aureobasidium pullulans*	KJ589574	950	99.6%
N-I-2	MW582316	*Cladosporium cladosporioides*	MK111524	896	99.8%
N-I-3	MW582317	*Cladosporium cladosporioides*	MK111524	902	100.0%
N-I-4	MW582318	*Cladosporium cladosporioides*	MF077224	935	99.6%
N-I-5	MW582319	Uncultured Ascomycota	KJ461327	924	99.8%
		Cladosporium tenuissimum	MT497425	922	100.0%
N-I-6	MW582320	*Didymella bellidis*	KX302079	915	99.8%
N-I-7	MW582321	*Trichoderma paraviridescens*	MN900599	966	100.0%
N-I-8	MW582322	*Xylaria primorskensis*	KT192367	632	91.4%
N-II-1	MW582323	Fungal sp.	KT375614	935	100.0%
		Alternaria viticola	JX418361	935	100.0%
N-II-2	MW582324	Uncultured fungus	KM877199	953	99.8%
		Aureobasidium pullulans	JN400825	953	99.8%
N-II-3	MW582325	Uncultured fungus	KM877199	961	99.3%
		Aureobasidium pullulans	JX188096	961	99.3%
N-II-4	MW582326	*Cladosporium cladosporioides*	MT609901	870	100.0%
N-II-5	MW582327	*Cladosporium tenuissimum*	MK881131	708	98.3%
N-II-6	MW582328	*Curvularia lunata*	MF380802	979	99.1%
N-II-7	MW582329	*Epicoccum nigrum*	MT355641	822	97.9%
N-II-8	MW582330	Fungal sp.	KJ650607	929	99.6%
		Fusarium tricinctum	MT180474	928	99.8%
N-II-9	MW582331	*Fusarium tricinctum*	MT180474	952	99.4%
N-II-10	MW582332	*Phaeosphaeria culmorum*	MK907699	710	100.0%
N-II-11	MW582333	*Phaeosphaeria vagans*	KF251193	944	99.8%
N-II-12	MW582334	*Trichoderma harzianum*	MT065756	1050	98.8%
N-II-13		*Trichoderma harzianum*	MH156141	137	100.0%
N-II-14	MW582335	*Trichoderma koningiopsis*	KU645324	1009	99.8%
N-III-1	MW582336	*Didymella glomerata*	GU724511	905	99.2%
N-III-2	MW582337	*Fusarium acuminatum*	MF687292	941	99.8%
N-III-3	MW582338	*Fusarium avenaceum*	KU057819	924	100.0%
N-III-4	MW582339	*Fusarium tricinctum*	MN833356	905	100.0%
N-III-5	MW582340	*Fusarium tricinctum*	MN833356	935	99.8%
N-III-6	MW582341	Fungal sp.	KJ650607	926	99.6%
		Fusarium tricinctum	HQ845047	926	99.6%
N-III-7	MW582342	*Fusarium tricinctum*	HQ845047	924	99.8%
N-III-8	MW582343	*Trichoderma koningiopsis*	KU645324	996	100.0%
N-III-9	MW582344	*Mucor hiemalis*	MT279287	1090	99.0%
N-III-10	MW582345	*Mucor hiemalis*	KT323200	1079	100.0%
N-III-11	MW582346	*Mucor hiemalis*	MH859263	1064	99.8%
N-IV-1	MW582347	*Alternaria alternata*	FJ717733	800	95.0%
N-IV-2	MW582348	*Alternaria alternata*	MK518403	970	99.6%
N-IV-3	MW582349	*Alternaria alternata*	MK285657	952	99.8%
N-IV-4	MW582350	*Alternaria citrimacularis*	JX418334	933	99.0%
N-IV-5	MW582351	*Cladosporium* sp.	MK111605	928	100.0%

Strain	GenBank code	Best BLAST matches	Accession code	Overlap length	% match
N-IV-6	MW582352	*Fusarium tricinctum*	MN833356	917	99.6%
N-IV-7	MW582353	*Fusarium tricinctum*	HQ845047	931	99.8%
N-IV-8		Ascomycota sp.	MN450645	529	100.0%
		Juxtiphoma eupyrena	MK907745	529	100.0%
N-IV-9	MW582354	*Paraphoma fimeti*	MH859843	1033	99.7%
N-IV-10	MW582355	*Leucosporidium creatinivorum*	JQ857019	889	100.0%
N-IV-11	MW582356	Fungal sp.	MT318485	811	100.0%
		Mucor hiemalis	MT093250	811	100.0%
N-V-1	MW582357	*Fusarium tricinctum*	MT180474	889	99.2%
N-V-2	MW582358	*Paraphoma fimeti*	MH859843	1018	100.0%
N-V-3	MW582359	*Leucosporidium creatinivorum*	JQ857019	961	100.0%
N-V-4	MW582360	*Mucor hiemalis*	MH859159	1079	99.8%
N-V-5	MW582361	*Mucor hiemalis*	MH859159	1081	99.7%
N-VI-1	MW582362	*Penicillium camemberti*	MT530220	826	100.0%
N-VI-2	MW582363	*Mucor hiemalis*	MT626048	811	90.6%
N-VI-3	MW582364	*Mucor hiemalis*	KU196760	1075	99.0%
N-VI-4	MW582365	*Mucor hiemalis*	MT514370	1057	99.7%
N-VI-5	MW582366	*Mucor hiemalis*	KT323202	809	91.6%
W-I-1	MW582367	*Cladosporium cladosporioides*	MG731215	922	99.6%
W-I-2	MW582368	*Penicillium expansum*	KC456327	1200	95.0%
W-I-3	MW582369	*Penicillium italicum*	MT582779	778	100.0%
W-I-4	MW582370	*Trichoderma atroviride*	HM047770	1011	100.0%
W-I-5	MW582371	*Trichoderma paraviridescens*	KY750537	1018	99.6%
W-I-6	MW582372	*Mucor hiemalis*	KY081701	950	99.8%
W-I-7	MW582373	*Mucor hiemalis*	MT514370	1068	99.5%
W-II-1	MW582374	Uncultured fungus	MT236408	907	99.6%
		Microdochium phragmitis	MH861162	907	99.6%
W-II-2	MW582375	*Mucor hiemalis*	KU196760	1081	100.0%
W-II-3	MW582376	*Mucor hiemalis*	KU196760	1077	99.7%
W-II-4	MW582377	*Mucor hiemalis*	KU196760	1085	99.5%
W-III-1	MW582378	*Cladosporium cladosporioides*	KR084328	918	99.6%
W-III-2	MW582379	*Cladosporium cladosporioides*	KR028334	929	99.0%
W-III-3	MW582380	*Curvularia lunata*	MH010914	1005	99.5%
W-III-4	MW582381	*Didymella glomerata*	MK907777	893	99.5%
W-III-5	MW582382	*Fusarium acuminatum*	KJ589601	986	99.0%
W-III-6		*Penicillium brevicompactum*	MT558924	477	98.2%
W-III-7	MW582383	*Penicillium brevicompactum*	MT558924	765	100.0%
W-III-8	MW582384	*Penicillium brevicompactum*	KY124643	957	99.1%
W-III-9	MW582385	*Mucor hiemalis*	KU196760	1081	99.8%
W-IV-1	MW582386	*Byssochlamys spectabilis*	MT609890	990	99.8%
W-IV-2	MW582387	*Candida tropicalis*	KT692959	957	99.4%
W-IV-3	MW582388	*Cephalotrichum nanum*	MH857949	1005	98.6%
W-IV-4	MW582389	*Didymella pomorum*	MK646044	896	99.8%
W-IV-5	MW582390	*Fusarium tricinctum*	MT180474	957	99.6%
W-IV-6	MW582391	Uncultured *Microdochium*	JF449471	885	98.4%
		Microdochium phragmitis	JF303866	881	98.8%
W-IV-7	MW582392	Hypocreales sp.	JN545830	900	99.0%

Strain	GenBank code	Best BLAST matches	Accession code	Overlap length	% match
		Nectria sp.	KU556523	893	99.6%
W-IV-8	MW582393	*Phoma herbarum*	LC514997	776	100.0%
W-IV-9	MW582394	*Phoma* sp.	JX045784	896	99.8%
W-IV-10	MW582395	*Mucor hiemalis*	KT323200	1079	100.0%
W-IV-11	MW582396	*Mucor hiemalis*	KU196760	1101	99.5%
W-V-1	MW582397	*Cladosporium herbarum*	MN486496	921	99.4%
W-V-2	MW582398	*Didymella glomerata*	GU724511	898	99.8%
W-V-3	MW582399	*Didymella* sp.	MN634502	885	98.6%
W-V-4	MW582400	*Juxtiphoma eupyrena*	MK907745	898	99.8%
W-V-5	MW582401	*Juxtiphoma eupyrena*	MK907745	878	99.2%
W-V-6	MW582402	*Leptosphaeria* sp.	KF657727	996	99.1%
W-V-7	MW582403	*Leptosphaeria* sp.	KF657727	987	100.0%
W-V-8	MW582404	*Paraphaeosphaeria sporulosa*	MH859903	1000	100.0%
W-V-9	MW582405	*Paraphaeosphaeria sporulosa*	MH859903	1007	100.0%
W-V-10	MW582406	*Paraphoma fimeti*	MG586984	1026	99.8%
W-V-11	MW582407	*Penicillium kongii*	MT558920	990	99.5%
W-V-12	MW582408	*Phoma herbarum*	LC085217	896	99.4%
W-V-13	MW582409	*Tangerinosporium thalitricola*	KU847243	985	98.9%
W-V-14	MW582410	Uncultured fungus	MF972066	941	99.8%
		Tricellula sp.	MH858506	898	98.8%
W-V-15	MW582411	Uncultured fungus	MF972066	741	100.0%
		Tricellula sp.	MH858506	713	98.8%
W-V-16	MW582412	*Trichoderma koningii*	HM037934	983	99.1%
W-V-17	MW582413	*Naganishia adeliensis*	JQ707891	1042	99.7%
W-V-18	MW582414	Uncultured fungus	MT236707	1042	100.0%
		Tausonia pullulans	KY105596	1042	100.0%
W-VI-1	MW582415	*Cladosporium cladosporioides*	MG731215	935	97.5%
W-VI-2	MW582416	*Cladosporium tenuissimum*	MT497425	918	100.0%
W-VI-3	MW582417	*Coniosporium* sp.	KT369825	573	84.3%
W-VI-4	MW582418	*Didymella glomerata*	MH864401	887	98.6%
W-VI-5	MW582419	*Epicoccum nigrum*	KX099630	911	99.8%
W-VI-6	MW582420	Uncultured fungus	FR682212	1138	99.1%
		Exophiala sp.	KC790479	989	95.8%
W-VI-7	MW582421	*Fusarium tricinctum*	MT180474	944	99.8%
W-VI-8	MW582422	Fungal sp.	HQ601953	800	96.7%
		Helotiales sp.	KM113762	798	96.7%
W-VI-9	MW582423	*Phoma herbarum*	DQ132841	904	99.4%
W-VI-10	MW582424	Uncultured fungus	MF972066	839	97.2%
		Tricellula sp.	MH858506	819	96.4%
W-VI-11	MW582425	Uncultured fungus	MF972066	780	100.0%
		Tricellula sp.	MH858506	752	98.8%
W-VI-12	MW582426	Uncultured fungus	MT236770	846	97.6%
		Xenochalara sp.	AY465471	819	98.3%
W-VI-13	MW582427	Uncultured fungus	HQ267074	1040	99.7%
		Tausonia pullulans	KY105596	1038	99.8%
W-VI-14	MW582428	Uncultured fungus	MT236707	1033	99.8%
		Tausonia pullulans	KY105596	1033	99.8%

Strain	GenBank code	Best BLAST matches	Accession code	Overlap length	% match
**Bacteria**					
W-VI-15	MW577449	*Chryseobacterium lactis*	MT065804	773	99.3%
W-VI-16	MW577450	Uncultured *Rhodococcus*	EU638729	743	99.0%
		Rhodococcus degradans	LR216744	737	98.8%
W-VI-17	MW577451	*Rhodococcus qingshengii*	MT632489	747	99.3%

BLAST search closest matches of fungal internal transcribed spacer and bacterial V3 - V4 hypervariable region DNA sequences amplified from Tianchi Volcano soil isolated strains. In strain codes, first letters (N and W) indicate the Northern and Western slopes, Roman numerals (I-VI) the soil samples, Arabic numerals the isolated fungal strains. Strain GenBank accession codes, accession codes for the closest GenBank matches, sequence identity, and overlap of each match are reported.

Table S2. An overview of fungal strains isolated from the crater soil samples.

Fungal Isolates	Class	Order	Strain Number		
			Total	North	West
**Ascomycota**					
Alternaria alternata	Dothideomycetes	Pleosporales	3	3	-
Alternaria citrimacularis	Dothideomycetes	Pleosporales	1	1	-
Alternaria viticola	Dothideomycetes	Pleosporales	1	1	-
Aureobasidium pullulans	Dothideomycetes	Dothideales	3	3	-
Byssochlamys spectabilis	Eurotiomycetes	Eurotiales	1	-	1
Candida tropicalis	Saccharomycetes	Saccharomycetales	1	-	1
Cephalotrichum nanum	Sordariomycetes	Microascales	1	-	1
Cladosporium cladosporioides	Dothideomycetes	Capnodiales	8	4	4
Cladosporium sp.	Dothideomycetes	Capnodiales	1	1	-
Cladosporium herbarum	Dothideomycetes	Capnodiales	1	-	1
Cladosporium tenuissimum	Dothideomycetes	Capnodiales	3	2	1
Coniosporium sp.	Dothideomycetes	Coniosporiales	1	-	1
Curvularia lunata	Dothideomycetes	Pleosporales	2	1	1
Didymella bellidis	Dothideomycetes	Pleosporales	1	1	-
Didymella glomerata	Dothideomycetes	Pleosporales	4	1	3
Didymella pomorum	Dothideomycetes	Pleosporales	1	-	1
Didymella sp.	Dothideomycetes	Pleosporales	1	-	1
Epicoccum nigrum	Dothideomycetes	Pleosporales	2	1	1
Exophiala sp.	Eurotiomycetes	Chaetothyriales	1	-	1
Fusarium acuminatum	Sordariomycetes	Hypocreales	2	1	1
Fusarium avenaceum	Sordariomycetes	Hypocreales	1	1	-
Fusarium tricinctum	Sordariomycetes	Hypocreales	11	9	2
Juxtiphoma eupyrena	Dothideomycetes	Pleosporales	3	1	2
Leptosphaeria sp.	Dothideomycetes	Pleosporales	2	-	2
Microdochium phragmitis	Sordariomycetes	Amphisphaeriales	2	-	2
Nectria sp.	Sordariomycetes	Hypocreales	1	-	1
Paraphaeosphaeria sporulosa	Dothideomycetes	Pleosporales	2	-	2
Paraphoma fimeti	Dothideomycetes	Pleosporales	3	2	1
Penicillium brevicompactum	Eurotiomycetes	Eurotiales	3	-	3
Penicillium camemberti	Eurotiomycetes	Eurotiales	1	1	-
Penicillium italicum	Eurotiomycetes	Eurotiales	1	-	1
Penicillium kongii	Eurotiomycetes	Eurotiales	1	-	1
Penicillium expansum	Eurotiomycetes	Eurotiales	1	-	1
Phaeosphaeria culmorum	Dothideomycetes	Pleosporales	1	1	-
Phaeosphaeria vagans	Dothideomycetes	Pleosporales	1	1	-
Phoma herbarum	Dothideomycetes	Pleosporales	3	-	3
Phoma sp.	Dothideomycetes	Pleosporales	1	-	1
Tangerinosporium thalitricola	Sordariomycetes	Hypocreales	1	-	1
Tricellula sp.	Incertae sedis	Incertae sedis	4	-	4
Trichoderma atroviride	Sordariomycetes	Hypocreales	1	-	1
Trichoderma harzianum	Sordariomycetes	Hypocreales	2	2	-
Trichoderma koningii	Sordariomycetes	Hypocreale	1	-	1

Trichoderma koningiopsis	Sordariomycetes	Hypocreales	2	2	-
Trichoderma paraviridescens	Sordariomycetes	Hypocreales	2	1	1
Xenochalara sp.	Leotiomycetes	Helotiales	1	-	1
Xylaria primorskensis	Sordariomycetes	Xylariales	1	1	-
Helotiales sp.	/	Helotiales	1	-	1
**Basidiomycota**					
Leucosporidium creatinivorum	Microbotryomycetes	Leucosporidiales	2	2	-
Naganishia adeliensis	Tremellomycetes	Tremellales	1	-	1
Tausonia pullulans	Tremellomycetes	Cystofilobasidiales	3	-	3
**Mucoromycota**					
Mucor hiemalis	Mucoromycetes	Mucorales	18	10	8

Table S3. Rarefied fungal OTUs table based on fungal ITS2 rDNA region Illumina Sequencing.

Domain	Kingdom	Phylum	Class	Order	Family	Genus	Species	OTU
d__Eukaryota	k__Fungi	p__Ascomycota	c__Dothideomycetes	o__Capnodiales	f__Teratosphaeriaceae	g__Devriesia	s__Devriesia_sp	OTU515
d__Eukaryota	k__Fungi	p__Ascomycota	c__Sordariomycetes	o__Xylariales	f__Xylariaceae	g__Daldinia	s__Daldinia_childiae	OTU428
d__Eukaryota	k__Fungi	p__Chytridiomycota	c__Rhizophydiomycetes	o__Rhizophydiales	f__unclassified_o__Rhizophydiales	g__unclassified_o__Rhizophydiales	s__Rhizophydiales_sp	OTU251
d__Eukaryota	k__Fungi	p__Ascomycota	c__Leotiomycetes	o__Helotiales	f__Helotiales_fam_Incertae_sedis	g__Coleophoma	s__Coleophoma_sp	OTU612
d__Eukaryota	k__Fungi	p__Ascomycota	c__Sordariomycetes	o__Coniochaetales	f__Coniochaetaceae	g__unclassified_f__Coniochaetaceae	s__unclassified_f__Coniochaetaceae	OTU767
d__Eukaryota	k__Fungi	p__Basidiomycota	c__Cystobasidiomycetes	o__Erythrobasidiales	f__Erythrobasidiales_fam_Incertae_sedis	g__Sakaguchia	s__unclassified_g__Sakaguchia	OTU247
d__Eukaryota	k__Fungi	p__Ascomycota	c__Leotiomycetes	o__Helotiales	f__unclassified_o__Helotiales	g__unclassified_o__Helotiales	s__unclassified_o__Helotiales	OTU936
d__Eukaryota	k__Fungi	p__Ascomycota	c__Eurotiomycetes	o__Chaetothyriales	f__Herpotrichiellaceae	g__Coniosporium	s__Coniosporium_sp	OTU464
d__Eukaryota	k__Fungi	p__Ascomycota	c__Leotiomycetes	o__Helotiales	f__Dermateaceae	g__Pezicula	s__Pezicula_ericae	OTU905
d__Eukaryota	k__Fungi	p__Ascomycota	c__Sordariomycetes	o__Coniochaetales	f__unclassified_o__Coniochaetales	g__unclassified_o__Coniochaetales	s__Coniochaetales_sp	OTU926
d__Eukaryota	k__Fungi	p__Basidiomycota	c__Agaricomycetes	o__Agaricales	f__Tricholomataceae	g__Macrocystidia	s__Macrocystidia_cucumis	OTU310
d__Eukaryota	k__Fungi	p__unclassified_k__Fungi	c__unclassified_k__Fungi	o__unclassified_k__Fungi	f__unclassified_k__Fungi	g__unclassified_k__Fungi	s__unclassified_k__Fungi	OTU463
d__Eukaryota	k__Fungi	p__unclassified_k__Fungi	c__unclassified_k__Fungi	o__unclassified_k__Fungi	f__unclassified_k__Fungi	g__unclassified_k__Fungi	s__unclassified_k__Fungi	OTU637
d__Eukaryota	k__Fungi	p__Ascomycota	c__Leotiomycetes	o__Helotiales	f__Leotiaceae	g__Pezoloma	s__unclassified_g__Pezoloma	OTU23
d__Eukaryota	k__Fungi	p__Ascomycota	c__unclassified_p__Ascomycota	o__unclassified_p__Ascomycota	f__unclassified_p__Ascomycota	g__unclassified_p__Ascomycota	s__unclassified_p__Ascomycota	OTU742
d__Eukaryota	k__Fungi	p__Basidiomycota	c__Microbotryomycetes	o__unclassified_c__Microbotryomycetes	f__unclassified_c__Microbotryomycetes	g__unclassified_c__Microbotryomycetes	s__unclassified_c__Microbotryomycetes	OTU185
d__Eukaryota	k__Fungi	p__Ascomycota	c__Pezizomycotina_cls_Incertae_sedis	o__Pezizomycotina_ord_Incertae_sedis	f__Pezizomycotina_fam_Incertae_sedis	g__Ciliophora	s__Ciliophora_sp	OTU1194
d__Eukaryota	k__Fungi	p__Fungi_phy_Incertae_sedis	c__Fungi_cls_Incertae_sedis	o__Fungi_ord_Incertae_sedis	f__Fungi_fam_Incertae_sedis	g__Desmococcus	s__Desmococcus_sp	OTU1092
d__Eukaryota	k__Fungi	p__unclassified_k__Fungi	c__unclassified_k__Fungi	o__unclassified_k__Fungi	f__unclassified_k__Fungi	g__unclassified_k__Fungi	s__unclassified_k__Fungi	OTU500
d__Eukaryota	k__Fungi	p__unclassified_k__Fungi	c__unclassified_k__Fungi	o__unclassified_k__Fungi	f__unclassified_k__Fungi	g__unclassified_k__Fungi	s__unclassified_k__Fungi	OTU265
d__Eukaryota	k__Fungi	p__Ascomycota	c__Eurotiomycetes	o__Sclerococcales	f__Dactylosporaceae	g__Dactylospora	s__Dactylospora_ahtii	OTU775
d__Eukaryota	k__Fungi	p__Ascomycota	c__unclassified_p__Ascomycota	o__unclassified_p__Ascomycota	f__unclassified_p__Ascomycota	g__unclassified_p__Ascomycota	s__unclassified_p__Ascomycota	OTU99
d__Eukaryota	k__Fungi	p__Ascomycota	c__unclassified_p__Ascomycota	o__unclassified_p__Ascomycota	f__unclassified_p__Ascomycota	g__unclassified_p__Ascomycota	s__unclassified_p__Ascomycota	OTU94
d__Eukaryota	k__Fungi	p__Monoblepharomycota	c__Sanchytriomycetes	o__Sanchytriales	f__Sanchytriaceae	g__unclassified_f__Sanchytriaceae	s__unclassified_f__Sanchytriaceae	OTU1052
d__Eukaryota	k__Fungi	p__Basidiomycota	c__Agaricomycetes	o__Agaricales	f__Lycoperdaceae	g__Bovista	s__Bovista_aestivalis	OTU584
d__Eukaryota	k__Fungi	p__Ascomycota	c__Orbiliomycetes	o__Orbiliales	f__Orbiliales_fam_Incertae_sedis	g__Lecophagus	s__Lecophagus_longisporus	OTU550
d__Eukaryota	k__Fungi	p__Mortierellomycota	c__Mortierellomycetes	o__Mortierellales	f__Mortierellaceae	g__Mortierella	s__Mortierella_hyalina	OTU540
d__Eukaryota	k__Fungi	p__unclassified_k__Fungi	c__unclassified_k__Fungi	o__unclassified_k__Fungi	f__unclassified_k__Fungi	g__unclassified_k__Fungi	s__unclassified_k__Fungi	OTU268
d__Eukaryota	k__Fungi	p__Basidiomycota	c__Microbotryomycetes	o__Leucosporidiales	f__Leucosporidiaceae	g__Leucosporidium	s__Leucosporidium_sp	OTU434
d__Eukaryota	k__Fungi	p__Basidiomycota	c__Agaricomycetes	o__Trechisporales	f__Hydnodontaceae	g__Trechispora	s__unclassified_g__Trechispora	OTU651

Domain	Kingdom	Phylum	Class	Order	Family	Genus	Species	OTU
d__Eukaryota	k__Fungi	p__Ascomycota	c__Orbiliomycetes	o__Orbiliales	f__Orbiliaceae	g__unclassified_f__Orbiliaceae	s__unclassified_f__Orbiliaceae	OTU645
d__Eukaryota	k__Fungi	p__Ascomycota	c__Eurotiomycetes	o__Verrucariales	f__Verrucariaceae	g__Verrucaria	s__Verrucaria_alpicola	OTU743
d__Eukaryota	k__Fungi	p__Ascomycota	c__Sordariomycetes	o__Hypocreales	f__Nectriaceae	g__unclassified_f__Nectriaceae	s__unclassified_f__Nectriaceae	OTU661
d__Eukaryota	k__Fungi	p__Ascomycota	c__Dothideomycetes	o__Dothideales	f__Dothideaceae	g__Celosporium	s__Celosporium_sp	OTU769
d__Eukaryota	k__Fungi	p__Ascomycota	c__Leotiomycetes	o__Helotiales	f__Helotiaceae	g__Articulospora	s__Articulospora_sp	OTU1036
d__Eukaryota	k__Fungi	p__unclassified_k__Fungi	c__unclassified_k__Fungi	o__unclassified_k__Fungi	f__unclassified_k__Fungi	g__unclassified_k__Fungi	s__unclassified_k__Fungi	OTU950
d__Eukaryota	k__Fungi	p__Ascomycota	c__Sordariomycetes	o__Sordariales	f__unclassified_o__Sordariales	g__unclassified_o__Sordariales	s__Sordariales_sp	OTU1188
d__Eukaryota	k__Fungi	p__Basidiomycota	c__Tremellomycetes	o__Cystofilobasidiales	f__Mrakiaceae	g__Itersonilia	s__Itersonilia_pastinacae	OTU622
d__Eukaryota	k__Fungi	p__Ascomycota	c__Dothideomycetes	o__Tubeufiales	f__Tubeufiaceae	g__Tubeufia	s__Tubeufia_cerea	OTU469
d__Eukaryota	k__Fungi	p__Ascomycota	c__Sordariomycetes	o__unclassified_c__Sordariomycetes	f__unclassified_c__Sordariomycetes	g__unclassified_c__Sordariomycetes	s__unclassified_c__Sordariomycetes	OTU577
d__Eukaryota	k__Fungi	p__Ascomycota	c__Dothideomycetes	o__Pleosporales	f__unclassified_o__Pleosporales	g__unclassified_o__Pleosporales	s__unclassified_o__Pleosporales	OTU762
d__Eukaryota	k__Fungi	p__Monoblepharomycota	c__unclassified_p__Monoblepharomycota	o__unclassified_p__Monoblepharomycota	f__unclassified_p__Monoblepharomycota	g__unclassified_p__Monoblepharomycota	s__unclassified_p__Monoblepharomycota	OTU262
d__Eukaryota	k__Fungi	p__Basidiomycota	c__Cystobasidiomycetes	o__Cystobasidiales	f__Cystobasidiaceae	g__Cystobasidium	s__Cystobasidium_slooffiae	OTU129
d__Eukaryota	k__Fungi	p__Ascomycota	c__Dothideomycetes	o__Pleosporales	f__unclassified_o__Pleosporales	g__unclassified_o__Pleosporales	s__unclassified_o__Pleosporales	OTU969
d__Eukaryota	k__Fungi	p__Ascomycota	c__Sordariomycetes	o__Glomerellales	f__Plectosphaerellaceae	g__Plectosphaerella	s__Plectosphaerella_cucumerina	OTU1124
d__Eukaryota	k__Fungi	p__Ascomycota	c__Leotiomycetes	o__Helotiales	f__unclassified_o__Helotiales	g__unclassified_o__Helotiales	s__unclassified_o__Helotiales	OTU394
d__Eukaryota	k__Fungi	p__Ascomycota	c__Dothideomycetes	o__Capnodiales	f__Cladosporiaceae	g__Rachicladosporium	s__Rachicladosporium_sp	OTU942
d__Eukaryota	k__Fungi	p__Ascomycota	c__Leotiomycetes	o__Thelebolales	f__Thelebolaceae	g__Thelebolus	s__Thelebolus_sp	OTU250
d__Eukaryota	k__Fungi	p__Basidiomycota	c__Agaricomycetes	o__Trechisporales	f__Hydnodontaceae	g__Trechispora	s__Trechispora_cohaerens	OTU83
d__Eukaryota	k__Fungi	p__Ascomycota	c__Dothideomycetes	o__Pleosporales	f__unclassified_o__Pleosporales	g__unclassified_o__Pleosporales	s__unclassified_o__Pleosporales	OTU768
d__Eukaryota	k__Fungi	p__Ascomycota	c__Dothideomycetes	o__Pleosporales	f__Torulaceae	g__Torula	s__Torula_ficus	OTU571
d__Eukaryota	k__Fungi	p__Ascomycota	c__Pezizomycotina_cls_Incertae_sedis	o__Pezizomycotina_ord_Incertae_sedis	f__Pezizomycotina_fam_Incertae_sedis	g__Ciliophora	s__Ciliophora_sp	OTU677
d__Eukaryota	k__Fungi	p__unclassified_k__Fungi	c__unclassified_k__Fungi	o__unclassified_k__Fungi	f__unclassified_k__Fungi	g__unclassified_k__Fungi	s__unclassified_k__Fungi	OTU659
d__Eukaryota	k__Fungi	p__Ascomycota	c__Dothideomycetes	o__Pleosporales	f__Leptosphaeriaceae	g__Plenodomus	s__unclassified_g__Plenodomus	OTU971
d__Eukaryota	k__Fungi	p__Ascomycota	c__unclassified_p__Ascomycota	o__unclassified_p__Ascomycota	f__unclassified_p__Ascomycota	g__unclassified_p__Ascomycota	s__unclassified_p__Ascomycota	OTU180
d__Eukaryota	k__Fungi	p__Basidiomycota	c__Agaricomycetes	o__Trechisporales	f__Hydnodontaceae	g__Trechispora	s__Trechispora_subsphaerospora	OTU629
d__Eukaryota	k__Fungi	p__unclassified_k__Fungi	c__unclassified_k__Fungi	o__unclassified_k__Fungi	f__unclassified_k__Fungi	g__unclassified_k__Fungi	s__unclassified_k__Fungi	OTU219
d__Eukaryota	k__Fungi	p__Basidiomycota	c__Agaricomycetes	o__Amylocorticiales	f__Amylocorticiaceae	g__Plicaturopsis	s__Plicaturopsis_crispa	OTU658
d__Eukaryota	k__Fungi	p__Ascomycota	c__unclassified_p__Ascomycota	o__unclassified_p__Ascomycota	f__unclassified_p__Ascomycota	g__unclassified_p__Ascomycota	s__unclassified_p__Ascomycota	OTU626
d__Eukaryota	k__Fungi	p__Ascomycota	c__Leotiomycetes	o__Erysiphales	f__Erysiphaceae	g__Podosphaera	s__Podosphaera_fusca	OTU25

Domain	Kingdom	Phylum	Class	Order	Family	Genus	Species	OTU
d__Eukaryota	k__Fungi	p__Ascomycota	c__Lecanoromycetes	o__Lecanorales	f__unclassified_o__Lecanorales	g__unclassified_o__Lecanorales	s__unclassified_o__Lecanorales	OTU466
d__Eukaryota	k__Fungi	p__Ascomycota	c__Dothideomycetes	o__Capnodiales	f__Teratosphaeriaceae	g__Elasticomyces	s__Elasticomyces_elasticus	OTU529
d__Eukaryota	k__Fungi	p__Ascomycota	c__Eurotiomycetes	o__Chaetothyriales	f__Herpotrichiellaceae	g__Cladophialophora	s__unclassified_g__Cladophialophora	OTU118
d__Eukaryota	k__Fungi	p__Ascomycota	c__Dothideomycetes	o__Capnodiales	f__Teratosphaeriaceae	g__Capnobotryella	s__Capnobotryella_renispora	OTU53
d__Eukaryota	k__Fungi	p__Chytridiomycota	c__unclassified_p__Chytridiomycota	o__unclassified_p__Chytridiomycota	f__unclassified_p__Chytridiomycota	g__unclassified_p__Chytridiomycota	s__unclassified_p__Chytridiomycota	OTU407
d__Eukaryota	k__Fungi	p__Ascomycota	c__Eurotiomycetes	o__Chaetothyriales	f__unclassified_o__Chaetothyriales	g__unclassified_o__Chaetothyriales	s__unclassified_o__Chaetothyriales	OTU401
d__Eukaryota	k__Fungi	p__Basidiomycota	c__Agaricomycetes	o__Trechisporales	f__Hydnodontaceae	g__Trechispora	s__Trechispora_invisitata	OTU68
d__Eukaryota	k__Fungi	p__Ascomycota	c__Sordariomycetes	o__Sordariales	f__Chaetomiaceae	g__Trichocladium	s__Trichocladium_asperum	OTU739
d__Eukaryota	k__Fungi	p__Ascomycota	c__unclassified_p__Ascomycota	o__unclassified_p__Ascomycota	f__unclassified_p__Ascomycota	g__unclassified_p__Ascomycota	s__unclassified_p__Ascomycota	OTU785
d__Eukaryota	k__Fungi	p__Chytridiomycota	c__unclassified_p__Chytridiomycota	o__unclassified_p__Chytridiomycota	f__unclassified_p__Chytridiomycota	g__unclassified_p__Chytridiomycota	s__unclassified_p__Chytridiomycota	OTU566
d__Eukaryota	k__Fungi	p__Ascomycota	c__Dothideomycetes	o__Pleosporales	f__Didymosphaeriaceae	g__Deniquelata	s__Deniquelata_sp	OTU152
d__Eukaryota	k__Fungi	p__Ascomycota	c__Dothideomycetes	o__Pleosporales	f__Massarinaceae	g__unclassified_f__Massarinaceae	s__unclassified_f__Massarinaceae	OTU292
d__Eukaryota	k__Fungi	p__Basidiomycota	c__Agaricomycetes	o__Agaricales	f__Inocybaceae	g__Inocybe	s__Inocybe_calamistrata	OTU703
d__Eukaryota	k__Fungi	p__Ascomycota	c__Leotiomycetes	o__Helotiales	f__unclassified_o__Helotiales	g__unclassified_o__Helotiales	s__unclassified_o__Helotiales	OTU644
d__Eukaryota	k__Fungi	p__Ascomycota	c__Sordariomycetes	o__Hypocreales	f__Hypocreales_fam_Incertae_sedis	g__Acremonium	s__Acremonium_furcatum	OTU69
d__Eukaryota	k__Fungi	p__unclassified_k__Fungi	c__unclassified_k__Fungi	o__unclassified_k__Fungi	f__unclassified_k__Fungi	g__unclassified_k__Fungi	s__unclassified_k__Fungi	OTU1005
d__Eukaryota	k__Fungi	p__Basidiomycota	c__Agaricomycetes	o__Hymenochaetales	f__Hymenochaetales_fam_Incertae_sedis	g__Trichaptum	s__Trichaptum_fuscoviolaceum	OTU98
d__Eukaryota	k__Fungi	p__Basidiomycota	c__Agaricomycetes	o__Agaricales	f__Psathyrellaceae	g__Psathyrella	s__unclassified_g__Psathyrella	OTU559
d__Eukaryota	k__Fungi	p__Ascomycota	c__Dothideomycetes	o__Pleosporales	f__Sporormiaceae	g__unclassified_f__Sporormiaceae	s__unclassified_f__Sporormiaceae	OTU254
d__Eukaryota	k__Fungi	p__Ascomycota	c__Leotiomycetes	o__unclassified_c__Leotiomycetes	f__unclassified_c__Leotiomycetes	g__unclassified_c__Leotiomycetes	s__unclassified_c__Leotiomycetes	OTU477
d__Eukaryota	k__Fungi	p__Ascomycota	c__unclassified_p__Ascomycota	o__unclassified_p__Ascomycota	f__unclassified_p__Ascomycota	g__unclassified_p__Ascomycota	s__unclassified_p__Ascomycota	OTU1259
d__Eukaryota	k__Fungi	p__Ascomycota	c__Leotiomycetes	o__Helotiales	f__Leotiaceae	g__Alatospora	s__Alatospora_sp	OTU952
d__Eukaryota	k__Fungi	p__Basidiomycota	c__Agaricomycetes	o__Agaricales	f__Tricholomataceae	g__unclassified_f__Tricholomataceae	s__unclassified_f__Tricholomataceae	OTU641
d__Eukaryota	k__Fungi	p__Basidiomycota	c__Agaricomycetes	o__Agaricales	f__Psathyrellaceae	g__Psathyrella	s__Psathyrella_microrhiza	OTU860
d__Eukaryota	k__Fungi	p__Ascomycota	c__Sordariomycetes	o__Sordariales	f__Lasiosphaeriaceae	g__Cercophora	s__unclassified_g__Cercophora	OTU755
d__Eukaryota	k__Fungi	p__Ascomycota	c__Eurotiomycetes	o__Chaetothyriales	f__Herpotrichiellaceae	g__unclassified_f__Herpotrichiellaceae	s__unclassified_f__Herpotrichiellaceae	OTU919
d__Eukaryota	k__Fungi	p__Ascomycota	c__Dothideomycetes	o__Capnodiales	f__Cladosporiaceae	g__Rachicladosporium	s__Rachicladosporium_sp	OTU229
d__Eukaryota	k__Fungi	p__Basidiomycota	c__Agaricomycetes	o__Hymenochaetales	f__Schizoporaceae	g__unclassified_f__Schizoporaceae	s__unclassified_f__Schizoporaceae	OTU492
d__Eukaryota	k__Fungi	p__Ascomycota	c__Leotiomycetes	o__Helotiales	f__unclassified_o__Helotiales	g__unclassified_o__Helotiales	s__unclassified_o__Helotiales	OTU733
d__Eukaryota	k__Fungi	p__Ascomycota	c__unclassified_p__Ascomycota	o__unclassified_p__Ascomycota	f__unclassified_p__Ascomycota	g__unclassified_p__Ascomycota	s__unclassified_p__Ascomycota	OTU725
d__Eukaryota	k__Fungi	p__Ascomycota	c__Sordariomycetes	o__Myrmecridiales	f__Myrmecridiaceae	g__Myrmecridium	s__Myrmecridium_schulzeri	OTU867

Domain	Kingdom	Phylum	Class	Order	Family	Genus	Species	OTU
d__Eukaryota	k__Fungi	p__Ascomycota	c__unclassified_p__Ascomycota	o__unclassified_p__Ascomycota	f__unclassified_p__Ascomycota	g__unclassified_p__Ascomycota	s__unclassified_p__Ascomycota	OTU657
d__Eukaryota	k__Fungi	p__Ascomycota	c__Saccharomycetes	o__Saccharomycetales	f__Dipodascaceae	g__Dipodascus	s__Dipodascus_armillariae	OTU51
d__Eukaryota	k__Fungi	p__Basidiomycota	c__Tremellomycetes	o__Filobasidiales	f__Filobasidiaceae	g__Filobasidium	s__unclassified_g__Filobasidium	OTU119
d__Eukaryota	k__Fungi	p__Ascomycota	c__Leotiomycetes	o__Helotiales	f__Helotiaceae	g__Tetracladium	s__Tetracladium_sp	OTU332
d__Eukaryota	k__Fungi	p__Ascomycota	c__unclassified_p__Ascomycota	o__unclassified_p__Ascomycota	f__unclassified_p__Ascomycota	g__unclassified_p__Ascomycota	s__unclassified_p__Ascomycota	OTU616
d__Eukaryota	k__Fungi	p__Ascomycota	c__Eurotiomycetes	o__Chaetothyriales	f__Herpotrichiellaceae	g__Capronia	s__Capronia_sp	OTU1207
d__Eukaryota	k__Fungi	p__Ascomycota	c__unclassified_p__Ascomycota	o__unclassified_p__Ascomycota	f__unclassified_p__Ascomycota	g__unclassified_p__Ascomycota	s__unclassified_p__Ascomycota	OTU101
d__Eukaryota	k__Fungi	p__Ascomycota	c__unclassified_p__Ascomycota	o__unclassified_p__Ascomycota	f__unclassified_p__Ascomycota	g__unclassified_p__Ascomycota	s__unclassified_p__Ascomycota	OTU100
d__Eukaryota	k__Fungi	p__Ascomycota	c__Lecanoromycetes	o__Lecanorales	f__Lecanoraceae	g__Lecanora	s__Lecanora_polytropa	OTU20
d__Eukaryota	k__Fungi	p__Ascomycota	c__unclassified_p__Ascomycota	o__unclassified_p__Ascomycota	f__unclassified_p__Ascomycota	g__unclassified_p__Ascomycota	s__unclassified_p__Ascomycota	OTU720
d__Eukaryota	k__Fungi	p__Ascomycota	c__unclassified_p__Ascomycota	o__unclassified_p__Ascomycota	f__unclassified_p__Ascomycota	g__unclassified_p__Ascomycota	s__unclassified_p__Ascomycota	OTU107
d__Eukaryota	k__Fungi	p__Ascomycota	c__unclassified_p__Ascomycota	o__unclassified_p__Ascomycota	f__unclassified_p__Ascomycota	g__unclassified_p__Ascomycota	s__unclassified_p__Ascomycota	OTU106
d__Eukaryota	k__Fungi	p__Basidiomycota	c__Agaricostilbomycetes	o__unclassified_c__Agaricostilbomycetes	f__unclassified_c__Agaricostilbomycetes	g__unclassified_c__Agaricostilbomycetes	s__Agaricostilbomycetes_sp	OTU201
d__Eukaryota	k__Fungi	p__Ascomycota	c__Lecanoromycetes	o__Peltigerales	f__Pannariaceae	g__Pectenia	s__Pectenia_sp	OTU561
d__Eukaryota	k__Fungi	p__Ascomycota	c__Dothideomycetes	o__Pleosporales	f__Cucurbitariaceae	g__Pyrenochaetopsis	s__Pyrenochaetopsis_sp	OTU170
d__Eukaryota	k__Fungi	p__Basidiomycota	c__unclassified_p__Basidiomycota	o__unclassified_p__Basidiomycota	f__unclassified_p__Basidiomycota	g__unclassified_p__Basidiomycota	s__unclassified_p__Basidiomycota	OTU139
d__Eukaryota	k__Fungi	p__unclassified_k__Fungi	c__unclassified_k__Fungi	o__unclassified_k__Fungi	f__unclassified_k__Fungi	g__unclassified_k__Fungi	s__unclassified_k__Fungi	OTU649
d__Eukaryota	k__Fungi	p__unclassified_k__Fungi	c__unclassified_k__Fungi	o__unclassified_k__Fungi	f__unclassified_k__Fungi	g__unclassified_k__Fungi	s__unclassified_k__Fungi	OTU648
d__Eukaryota	k__Fungi	p__Basidiomycota	c__Agaricomycetes	o__Trechisporales	f__Hydnodontaceae	g__Trechispora	s__Trechispora_sp	OTU351
d__Eukaryota	k__Fungi	p__unclassified_k__Fungi	c__unclassified_k__Fungi	o__unclassified_k__Fungi	f__unclassified_k__Fungi	g__unclassified_k__Fungi	s__unclassified_k__Fungi	OTU86
d__Eukaryota	k__Fungi	p__Ascomycota	c__Eurotiomycetes	o__Chaetothyriales	f__unclassified_o__Chaetothyriales	g__unclassified_o__Chaetothyriales	s__Chaetothyriales_sp	OTU751
d__Eukaryota	k__Fungi	p__Basidiomycota	c__Tremellomycetes	o__Filobasidiales	f__Filobasidiaceae	g__Heterocephalacria	s__Heterocephalacria_sp	OTU973
d__Eukaryota	k__Fungi	p__Ascomycota	c__Eurotiomycetes	o__Verrucariales	f__Verrucariaceae	g__Verrucaria	s__Verrucaria_alpicola	OTU802
d__Eukaryota	k__Fungi	p__Ascomycota	c__Sordariomycetes	o__Boliniales	f__Boliniaceae	g__Pseudovalsaria	s__Pseudovalsaria_sp	OTU642
d__Eukaryota	k__Fungi	p__Ascomycota	c__Dothideomycetes	o__Pleosporales	f__Cucurbitariaceae	g__Pyrenochaeta	s__Pyrenochaeta_cava	OTU516
d__Eukaryota	k__Fungi	p__Ascomycota	c__Sordariomycetes	o__Diaporthales	f__Schizoparmaceae	g__Coniella	s__Coniella_fragariae	OTU487
d__Eukaryota	k__Fungi	p__Ascomycota	c__Dothideomycetes	o__Capnodiales	f__unclassified_o__Capnodiales	g__unclassified_o__Capnodiales	s__unclassified_o__Capnodiales	OTU198
d__Eukaryota	k__Fungi	p__Basidiomycota	c__Tremellomycetes	o__Tremellales	f__Phaeotremellaceae	g__Phaeotremella	s__unclassified_g__Phaeotremella	OTU1183
d__Eukaryota	k__Fungi	p__Ascomycota	c__Dothideomycetes	o__Pleosporales	f__Didymosphaeriaceae	g__Paraphaeosphaeria	s__Paraphaeosphaeria_sporulosa	OTU1165
d__Eukaryota	k__Fungi	p__unclassified_k__Fungi	c__unclassified_k__Fungi	o__unclassified_k__Fungi	f__unclassified_k__Fungi	g__unclassified_k__Fungi	s__unclassified_k__Fungi	OTU1245

Domain	Kingdom	Phylum	Class	Order	Family	Genus	Species	OTU
d__Eukaryota	k__Fungi	p__unclassified_k__Fungi	c__unclassified_k__Fungi	o__unclassified_k__Fungi	f__unclassified_k__Fungi	g__unclassified_k__Fungi	s__unclassified_k__Fungi	OTU1247
d__Eukaryota	k__Fungi	p__Ascomycota	c__Dothideomycetes	o__Pleosporales	f__Phaeosphaeriaceae	g__Setomelanomma	s__Setomelanomma_holmii	OTU902
d__Eukaryota	k__Fungi	p__Ascomycota	c__Dothideomycetes	o__Capnodiales	f__Teratosphaeriaceae	g__Devriesia	s__Devriesia_pseudoamericana	OTU320
d__Eukaryota	k__Fungi	p__Mortierellomycota	c__Mortierellomycetes	o__Mortierellales	f__Mortierellaceae	g__Mortierella	s__Mortierella_alpina	OTU594
d__Eukaryota	k__Fungi	p__Ascomycota	c__Leotiomycetes	o__Helotiales	f__unclassified_o__Helotiales	g__unclassified_o__Helotiales	s__unclassified_o__Helotiales	OTU13
d__Eukaryota	k__Fungi	p__Ascomycota	c__unclassified_p__Ascomycota	o__unclassified_p__Ascomycota	f__unclassified_p__Ascomycota	g__unclassified_p__Ascomycota	s__unclassified_p__Ascomycota	OTU73
d__Eukaryota	k__Fungi	p__Ascomycota	c__Dothideomycetes	o__Pleosporales	f__Lophiostomataceae	g__Lophiostoma	s__Lophiostoma_sp	OTU1076
d__Eukaryota	k__Fungi	p__Ascomycota	c__Eurotiomycetes	o__Verrucariales	f__Verrucariaceae	g__unclassified_f__Verrucariaceae	s__unclassified_f__Verrucariaceae	OTU853
d__Eukaryota	k__Fungi	p__Basidiomycota	c__Tremellomycetes	o__Cystofilobasidiales	f__Cystofilobasidiaceae	g__Cystofilobasidium	s__Cystofilobasidium_infirmominiatum	OTU405
d__Eukaryota	k__Fungi	p__Ascomycota	c__unclassified_p__Ascomycota	o__unclassified_p__Ascomycota	f__unclassified_p__Ascomycota	g__unclassified_p__Ascomycota	s__unclassified_p__Ascomycota	OTU1046
d__Eukaryota	k__Fungi	p__Basidiomycota	c__Agaricomycetes	o__Agaricales	f__Psathyrellaceae	g__Coprinellus	s__Coprinellus_micaceus	OTU293
d__Eukaryota	k__Fungi	p__Ascomycota	c__Leotiomycetes	o__Helotiales	f__Hyaloscyphaceae	g__Hyaloscypha	s__unclassified_g__Hyaloscypha	OTU839
d__Eukaryota	k__Fungi	p__Ascomycota	c__Dothideomycetes	o__Pleosporales	f__Pleosporaceae	g__Alternaria	s__Alternaria_terricola	OTU749
d__Eukaryota	k__Fungi	p__Ascomycota	c__Dothideomycetes	o__Pleosporales	f__Sporormiaceae	g__Sporormiella	s__Sporormiella_sp	OTU716
d__Eukaryota	k__Fungi	p__Ascomycota	c__Dothideomycetes	o__Dothideales	f__Dothideaceae	g__unclassified_f__Dothideaceae	s__unclassified_f__Dothideaceae	OTU307
d__Eukaryota	k__Fungi	p__Ascomycota	c__Dothideomycetes	o__Pleosporales	f__unclassified_o__Pleosporales	g__unclassified_o__Pleosporales	s__unclassified_o__Pleosporales	OTU301
d__Eukaryota	k__Fungi	p__Ascomycota	c__Dothideomycetes	o__Pleosporales	f__Sporormiaceae	g__Preussia	s__Preussia_lignicola	OTU899
d__Eukaryota	k__Fungi	p__Basidiomycota	c__Tremellomycetes	o__Tremellales	f__Tremellaceae	g__unclassified_f__Tremellaceae	s__unclassified_f__Tremellaceae	OTU122
d__Eukaryota	k__Fungi	p__Ascomycota	c__unclassified_p__Ascomycota	o__unclassified_p__Ascomycota	f__unclassified_p__Ascomycota	g__unclassified_p__Ascomycota	s__unclassified_p__Ascomycota	OTU217
d__Eukaryota	k__Fungi	p__Ascomycota	c__unclassified_p__Ascomycota	o__unclassified_p__Ascomycota	f__unclassified_p__Ascomycota	g__unclassified_p__Ascomycota	s__unclassified_p__Ascomycota	OTU216
d__Eukaryota	k__Fungi	p__Ascomycota	c__Leotiomycetes	o__Helotiales	f__Helotiales_fam_Incertae_sedis	g__unclassified_f__Helotiales_fam_Incertae_sedis	s__unclassified_f__Helotiales_fam_Incertae_sedis	OTU932
d__Eukaryota	k__Fungi	p__Ascomycota	c__Dothideomycetes	o__Pleosporales	f__Lophiostomataceae	g__Biappendiculispora	s__Biappendiculispora_sp	OTU663
d__Eukaryota	k__Fungi	p__Ascomycota	c__Dothideomycetes	o__Capnodiales	f__Capnodiales_fam_Incertae_sedis	g__Arthrocatena	s__Arthrocatena_sp	OTU55
d__Eukaryota	k__Fungi	p__Ascomycota	c__Dothideomycetes	o__Capnodiales	f__Teratosphaeriaceae	g__Bryochiton	s__Bryochiton_sp	OTU945
d__Eukaryota	k__Fungi	p__Ascomycota	c__Eurotiomycetes	o__Chaetothyriales	f__Trichomeriaceae	g__unclassified_f__Trichomeriaceae	s__unclassified_f__Trichomeriaceae	OTU997
d__Eukaryota	k__Fungi	p__Basidiomycota	c__Agaricomycetes	o__Thelephorales	f__Thelephoraceae	g__Tomentella	s__Tomentella_ellisii	OTU568
d__Eukaryota	k__Fungi	p__Ascomycota	c__Eurotiomycetes	o__Verrucariales	f__Verrucariaceae	g__unclassified_f__Verrucariaceae	s__unclassified_f__Verrucariaceae	OTU470
d__Eukaryota	k__Fungi	p__Ascomycota	c__Dothideomycetes	o__Pleosporales	f__Periconiaceae	g__Periconia	s__unclassified_g__Periconia	OTU726
d__Eukaryota	k__Fungi	p__Ascomycota	c__Sordariomycetes	o__Hypocreales	f__Nectriaceae	g__Gibberella	s__Gibberella_tricincta	OTU621
d__Eukaryota	k__Fungi	p__unclassified_k__Fungi	c__unclassified_k__Fungi	o__unclassified_k__Fungi	f__unclassified_k__Fungi	g__unclassified_k__Fungi	s__unclassified_k__Fungi	OTU383

Domain	Kingdom	Phylum	Class	Order	Family	Genus	Species	OTU
d__Eukaryota	k__Fungi	p__Basidiomycota	c__Agaricomycetes	o__Polyporales	f__Meripilaceae	g__unclassified_f__Meripilaceae	s__unclassified_f__Meripilaceae	OTU884
d__Eukaryota	k__Fungi	p__Ascomycota	c__Pezizomycetes	o__Pezizales	f__Helvellaceae	g__Helvella	s__unclassified_g__Helvella	OTU1048
d__Eukaryota	k__Fungi	p__Basidiomycota	c__Agaricomycetes	o__Agaricales	f__Crepidotaceae	g__Crepidotus	s__Crepidotus_mollis	OTU309
d__Eukaryota	k__Fungi	p__Basidiomycota	c__Cystobasidiomycetes	o__Cystobasidiomycetes_ord_Incertae_sedis	f__Symmetrosporaceae	g__Symmetrospora	s__Symmetrospora_sp	OTU194
d__Eukaryota	k__Fungi	p__unclassified_k__Fungi	c__unclassified_k__Fungi	o__unclassified_k__Fungi	f__unclassified_k__Fungi	g__unclassified_k__Fungi	s__unclassified_k__Fungi	OTU1014
d__Eukaryota	k__Fungi	p__unclassified_k__Fungi	c__unclassified_k__Fungi	o__unclassified_k__Fungi	f__unclassified_k__Fungi	g__unclassified_k__Fungi	s__unclassified_k__Fungi	OTU1017
d__Eukaryota	k__Fungi	p__Ascomycota	c__Sordariomycetes	o__Hypocreales	f__Nectriaceae	g__Neocosmospora	s__Neocosmospora_perseae	OTU465
d__Eukaryota	k__Fungi	p__Ascomycota	c__Leotiomycetes	o__Helotiales	f__Helotiales_fam_Incertae_sedis	g__Xenopolyscytalum	s__Xenopolyscytalum_sp	OTU845
d__Eukaryota	k__Fungi	p__unclassified_k__Fungi	c__unclassified_k__Fungi	o__unclassified_k__Fungi	f__unclassified_k__Fungi	g__unclassified_k__Fungi	s__unclassified_k__Fungi	OTU1056
d__Eukaryota	k__Fungi	p__Ascomycota	c__Eurotiomycetes	o__Chaetothyriales	f__Trichomeriaceae	g__Knufia	s__Knufia_cryptophialidica	OTU335
d__Eukaryota	k__Fungi	p__unclassified_k__Fungi	c__unclassified_k__Fungi	o__unclassified_k__Fungi	f__unclassified_k__Fungi	g__unclassified_k__Fungi	s__unclassified_k__Fungi	OTU195
d__Eukaryota	k__Fungi	p__unclassified_k__Fungi	c__unclassified_k__Fungi	o__unclassified_k__Fungi	f__unclassified_k__Fungi	g__unclassified_k__Fungi	s__unclassified_k__Fungi	OTU197
d__Eukaryota	k__Fungi	p__Ascomycota	c__Sordariomycetes	o__Hypocreales	f__Hypocreaceae	g__Trichoderma	s__unclassified_g__Trichoderma	OTU164
d__Eukaryota	k__Fungi	p__Mortierellomycota	c__Mortierellomycetes	o__Mortierellales	f__Mortierellaceae	g__Mortierella	s__Mortierella_alpina	OTU114
d__Eukaryota	k__Fungi	p__Basidiomycota	c__unclassified_p__Basidiomycota	o__unclassified_p__Basidiomycota	f__unclassified_p__Basidiomycota	g__unclassified_p__Basidiomycota	s__unclassified_p__Basidiomycota	OTU710
d__Eukaryota	k__Fungi	p__Ascomycota	c__unclassified_p__Ascomycota	o__unclassified_p__Ascomycota	f__unclassified_p__Ascomycota	g__unclassified_p__Ascomycota	s__unclassified_p__Ascomycota	OTU46
d__Eukaryota	k__Fungi	p__Basidiomycota	c__Tremellomycetes	o__Tremellales	f__Bulleribasidiaceae	g__Dioszegia	s__unclassified_g__Dioszegia	OTU1147
d__Eukaryota	k__Fungi	p__Ascomycota	c__unclassified_p__Ascomycota	o__unclassified_p__Ascomycota	f__unclassified_p__Ascomycota	g__unclassified_p__Ascomycota	s__unclassified_p__Ascomycota	OTU44
d__Eukaryota	k__Fungi	p__Ascomycota	c__Arthoniomycetes	o__Lichenostigmatales	f__Phaeococcomycetaceae	g__Phaeococcomyces	s__Phaeococcomyces_sp	OTU65
d__Eukaryota	k__Fungi	p__Chytridiomycota	c__unclassified_p__Chytridiomycota	o__unclassified_p__Chytridiomycota	f__unclassified_p__Chytridiomycota	g__unclassified_p__Chytridiomycota	s__Chytridiomycota_sp	OTU673
d__Eukaryota	k__Fungi	p__Ascomycota	c__Dothideomycetes	o__Pleosporales	f__Leptosphaeriaceae	g__Subplenodomus	s__Subplenodomus_iridicola	OTU103
d__Eukaryota	k__Fungi	p__Basidiomycota	c__Agaricomycetes	o__Phallales	f__Phallaceae	g__Phallus	s__Phallus_sp	OTU513
d__Eukaryota	k__Fungi	p__Ascomycota	c__Dothideomycetes	o__Pleosporales	f__unclassified_o__Pleosporales	g__unclassified_o__Pleosporales	s__unclassified_o__Pleosporales	OTU337
d__Eukaryota	k__Fungi	p__Basidiomycota	c__Agaricomycetes	o__Agaricales	f__Bolbitiaceae	g__Conocybe	s__Conocybe_vexans	OTU761
d__Eukaryota	k__Fungi	p__unclassified_k__Fungi	c__unclassified_k__Fungi	o__unclassified_k__Fungi	f__unclassified_k__Fungi	g__unclassified_k__Fungi	s__unclassified_k__Fungi	OTU421
d__Eukaryota	k__Fungi	p__Ascomycota	c__Dothideomycetes	o__Pleosporales	f__Phaeosphaeriaceae	g__Ophiobolopsis	s__Ophiobolopsis_italica	OTU182
d__Eukaryota	k__Fungi	p__Basidiomycota	c__Agaricomycetes	o__Trechisporales	f__unclassified_o__Trechisporales	g__unclassified_o__Trechisporales	s__unclassified_o__Trechisporales	OTU486
d__Eukaryota	k__Fungi	p__Ascomycota	c__Dothideomycetes	o__Pleosporales	f__unclassified_o__Pleosporales	g__unclassified_o__Pleosporales	s__Pleosporales_sp	OTU782
d__Eukaryota	k__Fungi	p__Ascomycota	c__Dothideomycetes	o__Pleosporales	f__Didymosphaeriaceae	g__Tremateia	s__Tremateia_sp	OTU288
d__Eukaryota	k__Fungi	p__Basidiomycota	c__Agaricomycetes	o__Agaricales	f__Bolbitiaceae	g__Conocybe	s__Conocybe_hausknechtii	OTU631

Domain	Kingdom	Phylum	Class	Order	Family	Genus	Species	OTU
d__Eukaryota	k__Fungi	p__Ascomycota	c__Orbiliomycetes	o__Orbiliales	f__Orbiliaceae	g__Dactylaria	s__Dactylaria_dimorphospora	OTU1131
d__Eukaryota	k__Fungi	p__Chytridiomycota	c__unclassified_p__Chytridiomycota	o__unclassified_p__Chytridiomycota	f__unclassified_p__Chytridiomycota	g__unclassified_p__Chytridiomycota	s__unclassified_p__Chytridiomycota	OTU355
d__Eukaryota	k__Fungi	p__unclassified_k__Fungi	c__unclassified_k__Fungi	o__unclassified_k__Fungi	f__unclassified_k__Fungi	g__unclassified_k__Fungi	s__unclassified_k__Fungi	OTU1182
d__Eukaryota	k__Fungi	p__Basidiomycota	c__Tremellomycetes	o__Cystofilobasidiales	f__Mrakiaceae	g__Mrakia	s__Mrakia_blollopis	OTU1204
d__Eukaryota	k__Fungi	p__Ascomycota	c__Pezizomycetes	o__Pezizales	f__Pyronemataceae	g__Tricharina	s__Tricharina_gilva	OTU1196
d__Eukaryota	k__Fungi	p__Ascomycota	c__Dothideomycetes	o__Pleosporales	f__Pleomassariaceae	g__Tumularia	s__Tumularia_aquatica	OTU130
d__Eukaryota	k__Fungi	p__Ascomycota	c__Leotiomycetes	o__Helotiales	f__Sclerotiniaceae	g__Sclerotinia	s__Sclerotinia_nivalis	OTU675
d__Eukaryota	k__Fungi	p__Ascomycota	c__Dothideomycetes	o__Botryosphaeriales	f__Aplosporellaceae	g__Aplosporella	s__Aplosporella_longipes	OTU722
d__Eukaryota	k__Fungi	p__Ascomycota	c__Lecanoromycetes	o__Acarosporales	f__Acarosporaceae	g__Acarospora	s__Acarospora_rugulosa	OTU281
d__Eukaryota	k__Fungi	p__Basidiomycota	c__Agaricomycetes	o__Polyporales	f__Meruliaceae	g__Phlebia	s__Phlebia_rufa	OTU617
d__Eukaryota	k__Fungi	p__Ascomycota	c__Leotiomycetes	o__Helotiales	f__unclassified_o__Helotiales	g__unclassified_o__Helotiales	s__unclassified_o__Helotiales	OTU787
d__Eukaryota	k__Fungi	p__Ascomycota	c__Saccharomycetes	o__Saccharomycetales	f__Lipomycetaceae	g__Lipomyces	s__Lipomyces_starkeyi	OTU830
d__Eukaryota	k__Fungi	p__Fungi_phy_Incertae_sedis	c__Fungi_cls_Incertae_sedis	o__Fungi_ord_Incertae_sedis	f__Fungi_fam_Incertae_sedis	g__Desmococcus	s__Desmococcus_sp	OTU736
d__Eukaryota	k__Fungi	p__Mucoromycota	c__Mucoromycetes	o__Mucorales	f__Mucoraceae	g__Mucor	s__Mucor_hiemalis	OTU552
d__Eukaryota	k__Fungi	p__Ascomycota	c__Lecanoromycetes	o__Caliciales	f__Caliciaceae	g__Buellia	s__unclassified_g__Buellia	OTU924
d__Eukaryota	k__Fungi	p__Ascomycota	c__Dothideomycetes	o__Pleosporales	f__Sporormiaceae	g__Sporormiella	s__Sporormiella_minima	OTU126
d__Eukaryota	k__Fungi	p__Ascomycota	c__Leotiomycetes	o__Helotiales	f__Helotiaceae	g__Heyderia	s__Heyderia_abietis	OTU989
d__Eukaryota	k__Fungi	p__Ascomycota	c__unclassified_p__Ascomycota	o__unclassified_p__Ascomycota	f__unclassified_p__Ascomycota	g__unclassified_p__Ascomycota	s__unclassified_p__Ascomycota	OTU660
d__Eukaryota	k__Fungi	p__Ascomycota	c__Leotiomycetes	o__Helotiales	f__Hyaloscyphaceae	g__Cistella	s__unclassified_g__Cistella	OTU788
d__Eukaryota	k__Fungi	p__Ascomycota	c__Dothideomycetes	o__Pleosporales	f__Trematosphaeriaceae	g__Trematosphaeria	s__Trematosphaeria_hydrela	OTU815
d__Eukaryota	k__Fungi	p__Ascomycota	c__Lecanoromycetes	o__Lecanorales	f__Lecanoraceae	g__Lecanora	s__Lecanora_layana	OTU400
d__Eukaryota	k__Fungi	p__Ascomycota	c__unclassified_p__Ascomycota	o__unclassified_p__Ascomycota	f__unclassified_p__Ascomycota	g__unclassified_p__Ascomycota	s__unclassified_p__Ascomycota	OTU585
d__Eukaryota	k__Fungi	p__Ascomycota	c__Leotiomycetes	o__Helotiales	f__Helotiales_fam_Incertae_sedis	g__Rhexocercosporidium	s__Rhexocercosporidium_panacis	OTU801
d__Eukaryota	k__Fungi	p__Ascomycota	c__Leotiomycetes	o__Helotiales	f__Helotiaceae	g__Discinella	s__Discinella_boudieri	OTU951
d__Eukaryota	k__Fungi	p__Ascomycota	c__Eurotiomycetes	o__Onygenales	f__Arthrodermataceae	g__Arthroderma	s__Arthroderma_sp	OTU995
d__Eukaryota	k__Fungi	p__Ascomycota	c__Sordariomycetes	o__Xylariales	f__Xylariales_fam_Incertae_sedis	g__Hansfordia	s__Hansfordia_pulvinata	OTU480
d__Eukaryota	k__Fungi	p__Ascomycota	c__Leotiomycetes	o__unclassified_c__Leotiomycetes	f__unclassified_c__Leotiomycetes	g__unclassified_c__Leotiomycetes	s__unclassified_c__Leotiomycetes	OTU954
d__Eukaryota	k__Fungi	p__unclassified_k__Fungi	c__unclassified_k__Fungi	o__unclassified_k__Fungi	f__unclassified_k__Fungi	g__unclassified_k__Fungi	s__unclassified_k__Fungi	OTU551
d__Eukaryota	k__Fungi	p__Ascomycota	c__Dothideomycetes	o__Capnodiales	f__unclassified_o__Capnodiales	g__unclassified_o__Capnodiales	s__unclassified_o__Capnodiales	OTU165
d__Eukaryota	k__Fungi	p__Ascomycota	c__unclassified_p__Ascomycota	o__unclassified_p__Ascomycota	f__unclassified_p__Ascomycota	g__unclassified_p__Ascomycota	s__unclassified_p__Ascomycota	OTU748
d__Eukaryota	k__Fungi	p__Ascomycota	c__Dothideomycetes	o__Capnodiales	f__Teratosphaeriaceae	g__Lapidomyces	s__Lapidomyces_sp	OTU190

Domain	Kingdom	Phylum	Class	Order	Family	Genus	Species	OTU
d__Eukaryota	k__Fungi	p__Ascomycota	c__Pezizomycotina_cls_Incertae_sedis	o__Pezizomycotina_ord_Incertae_sedis	f__Pezizomycotina_fam_Incertae_sedis	g__Ciliophora	s__Ciliophora_sp	OTU436
d__Eukaryota	k__Fungi	p__Ascomycota	c__Sordariomycetes	o__Myrmecridiales	f__Myrmecridiaceae	g__Myrmecridium	s__unclassified_g__Myrmecridium	OTU1166
d__Eukaryota	k__Fungi	p__Basidiomycota	c__unclassified_p__Basidiomycota	o__unclassified_p__Basidiomycota	f__unclassified_p__Basidiomycota	g__unclassified_p__Basidiomycota	s__unclassified_p__Basidiomycota	OTU940
d__Eukaryota	k__Fungi	p__Ascomycota	c__Dothideomycetes	o__Dothideales	f__Aureobasidiaceae	g__Aureobasidium	s__unclassified_g__Aureobasidium	OTU1184
d__Eukaryota	k__Fungi	p__Ascomycota	c__Leotiomycetes	o__Helotiales	f__Helotiales_fam_Incertae_sedis	g__Cadophora	s__unclassified_g__Cadophora	OTU66
d__Eukaryota	k__Fungi	p__Ascomycota	c__Saccharomycetes	o__Saccharomycetales	f__Saccharomycetaceae	g__Saccharomyces	s__Saccharomyces_sp	OTU323
d__Eukaryota	k__Fungi	p__Ascomycota	c__Dothideomycetes	o__Capnodiales	f__Mycosphaerellaceae	g__Acrodontium	s__Acrodontium_sp	OTU314
d__Eukaryota	k__Fungi	p__Ascomycota	c__Dothideomycetes	o__Pleosporales	f__unclassified_o__Pleosporales	g__unclassified_o__Pleosporales	s__unclassified_o__Pleosporales	OTU1123
d__Eukaryota	k__Fungi	p__Ascomycota	c__Sordariomycetes	o__unclassified_c__Sordariomycetes	f__unclassified_c__Sordariomycetes	g__unclassified_c__Sordariomycetes	s__unclassified_c__Sordariomycetes	OTU1159
d__Eukaryota	k__Fungi	p__Ascomycota	c__Leotiomycetes	o__unclassified_c__Leotiomycetes	f__unclassified_c__Leotiomycetes	g__unclassified_c__Leotiomycetes	s__unclassified_c__Leotiomycetes	OTU636
d__Eukaryota	k__Fungi	p__Ascomycota	c__Lecanoromycetes	o__Pertusariales	f__Megasporaceae	g__Aspicilia	s__Aspicilia_subradians	OTU329
d__Eukaryota	k__Fungi	p__Ascomycota	c__Leotiomycetes	o__Helotiales	f__Helotiales_fam_Incertae_sedis	g__Eleutheromyces	s__unclassified_g__Eleutheromyces	OTU576
d__Eukaryota	k__Fungi	p__Basidiomycota	c__Tremellomycetes	o__Filobasidiales	f__Piskurozymaceae	g__Solicoccozyma	s__Solicoccozyma_terricola	OTU432
d__Eukaryota	k__Fungi	p__Basidiomycota	c__Agaricomycetes	o__Agaricales	f__Tricholomataceae	g__Clitocybula	s__Clitocybula_lacerata	OTU528
d__Eukaryota	k__Fungi	p__Basidiomycota	c__unclassified_p__Basidiomycota	o__unclassified_p__Basidiomycota	f__unclassified_p__Basidiomycota	g__unclassified_p__Basidiomycota	s__unclassified_p__Basidiomycota	OTU39
d__Eukaryota	k__Fungi	p__Ascomycota	c__Sordariomycetes	o__Sordariales	f__Chaetomiaceae	g__Achaetomium	s__unclassified_g__Achaetomium	OTU532
d__Eukaryota	k__Fungi	p__Ascomycota	c__Lecanoromycetes	o__Lecanorales	f__unclassified_o__Lecanorales	g__unclassified_o__Lecanorales	s__unclassified_o__Lecanorales	OTU196
d__Eukaryota	k__Fungi	p__Ascomycota	c__Leotiomycetes	o__Helotiales	f__unclassified_o__Helotiales	g__unclassified_o__Helotiales	s__unclassified_o__Helotiales	OTU728
d__Eukaryota	k__Fungi	p__Ascomycota	c__Orbiliomycetes	o__Orbiliales	f__Orbiliaceae	g__Dactylaria	s__Dactylaria_fragilis	OTU946
d__Eukaryota	k__Fungi	p__Ascomycota	c__Eurotiomycetes	o__Chaetothyriales	f__Herpotrichiellaceae	g__Coniosporium	s__Coniosporium_sp	OTU849
d__Eukaryota	k__Fungi	p__Ascomycota	c__Leotiomycetes	o__Helotiales	f__Helotiaceae	g__Ascocoryne	s__unclassified_g__Ascocoryne	OTU738
d__Eukaryota	k__Fungi	p__Basidiomycota	c__Agaricomycetes	o__Russulales	f__Auriscalpiaceae	g__Lentinellus	s__Lentinellus_sp	OTU505
d__Eukaryota	k__Fungi	p__Ascomycota	c__Dothideomycetes	o__Pleosporales	f__Didymosphaeriaceae	g__unclassified_f__Didymosphaeriaceae	s__unclassified_f__Didymosphaeriaceae	OTU1078
d__Eukaryota	k__Fungi	p__Basidiomycota	c__Agaricomycetes	o__Agaricales	f__Strophariaceae	g__Pholiota	s__Pholiota_lubrica	OTU303
d__Eukaryota	k__Fungi	p__Basidiomycota	c__Tremellomycetes	o__Tremellales	f__Bulleribasidiaceae	g__Vishniacozyma	s__Vishniacozyma_tephrensis	OTU1027
d__Eukaryota	k__Fungi	p__Ascomycota	c__Leotiomycetes	o__Helotiales	f__unclassified_o__Helotiales	g__unclassified_o__Helotiales	s__unclassified_o__Helotiales	OTU334
d__Eukaryota	k__Fungi	p__Ascomycota	c__Dothideomycetes	o__Pleosporales	f__unclassified_o__Pleosporales	g__unclassified_o__Pleosporales	s__unclassified_o__Pleosporales	OTU766
d__Eukaryota	k__Fungi	p__unclassified_k__Fungi	c__unclassified_k__Fungi	o__unclassified_k__Fungi	f__unclassified_k__Fungi	g__unclassified_k__Fungi	s__unclassified_k__Fungi	OTU57
d__Eukaryota	k__Fungi	p__Ascomycota	c__unclassified_p__Ascomycota	o__unclassified_p__Ascomycota	f__unclassified_p__Ascomycota	g__unclassified_p__Ascomycota	s__unclassified_p__Ascomycota	OTU84
d__Eukaryota	k__Fungi	p__Basidiomycota	c__Agaricomycetes	o__Russulales	f__Bondarzewiaceae	g__Heterobasidion	s__Heterobasidion_sp	OTU851
d__Eukaryota	k__Fungi	p__Ascomycota	c__Dothideomycetes	o__Pleosporales	f__Phaeosphaeriaceae	g__Neosulcatispora	s__Neosulcatispora_strelitziae	OTU47

Domain	Kingdom	Phylum	Class	Order	Family	Genus	Species	OTU
d__Eukaryota	k__Fungi	p__Basidiomycota	c__Agaricomycetes	o__Agaricales	f__Agaricaceae	g__Agaricus	s__Agaricus_sinoplacomyces	OTU490
d__Eukaryota	k__Fungi	p__Ascomycota	c__Leotiomycetes	o__Helotiales	f__Vibrisseaceae	g__Phialocephala	s__Phialocephala_bamuru	OTU906
d__Eukaryota	k__Fungi	p__Ascomycota	c__Dothideomycetes	o__Pleosporales	f__Pleosporaceae	g__Bipolaris	s__Bipolaris_zeicola	OTU43
d__Eukaryota	k__Fungi	p__Ascomycota	c__Sordariomycetes	o__Sordariales	f__Lasiosphaeriaceae	g__Fimetariella	s__Fimetariella_rabenhorstii	OTU141
d__Eukaryota	k__Fungi	p__Ascomycota	c__unclassified_p__Ascomycota	o__unclassified_p__Ascomycota	f__unclassified_p__Ascomycota	g__unclassified_p__Ascomycota	s__unclassified_p__Ascomycota	OTU1142
d__Eukaryota	k__Fungi	p__Ascomycota	c__Dothideomycetes	o__Pleosporales	f__Leptosphaeriaceae	g__Leptosphaeria	s__Leptosphaeria_cichorium	OTU256
d__Eukaryota	k__Fungi	p__Ascomycota	c__Lecanoromycetes	o__Lecanorales	f__Parmeliaceae	g__Punctelia	s__Punctelia_borreri	OTU999
d__Eukaryota	k__Fungi	p__Ascomycota	c__Lecanoromycetes	o__Lecanorales	f__unclassified_o__Lecanorales	g__unclassified_o__Lecanorales	s__unclassified_o__Lecanorales	OTU581
d__Eukaryota	k__Fungi	p__Ascomycota	c__Dothideomycetes	o__Pleosporales	f__Cucurbitariaceae	g__Pyrenochaetopsis	s__Pyrenochaetopsis_leptospora	OTU962
d__Eukaryota	k__Fungi	p__Basidiomycota	c__Agaricomycetes	o__Polyporales	f__Meruliaceae	g__Phanerochaete	s__Phanerochaete_sordida	OTU600
d__Eukaryota	k__Fungi	p__unclassified_k__Fungi	c__unclassified_k__Fungi	o__unclassified_k__Fungi	f__unclassified_k__Fungi	g__unclassified_k__Fungi	s__unclassified_k__Fungi	OTU148
d__Eukaryota	k__Fungi	p__Ascomycota	c__Leotiomycetes	o__Helotiales	f__Hyaloscyphaceae	g__Calycina	s__Calycina_sp	OTU980
d__Eukaryota	k__Fungi	p__Ascomycota	c__Arthoniomycetes	o__Lichenostigmatales	f__unclassified_o__Lichenostigmatales	g__unclassified_o__Lichenostigmatales	s__Lichenostigmatales_sp	OTU188
d__Eukaryota	k__Fungi	p__Ascomycota	c__Sordariomycetes	o__Hypocreales	f__Nectriaceae	g__Fusarium	s__Fusarium_proliferatum	OTU125
d__Eukaryota	k__Fungi	p__Ascomycota	c__Eurotiomycetes	o__Chaetothyriales	f__Chaetothyriaceae	g__Chaetothyrium	s__Chaetothyrium_sp	OTU692
d__Eukaryota	k__Fungi	p__unclassified_k__Fungi	c__unclassified_k__Fungi	o__unclassified_k__Fungi	f__unclassified_k__Fungi	g__unclassified_k__Fungi	s__unclassified_k__Fungi	OTU374
d__Eukaryota	k__Fungi	p__Ascomycota	c__Leotiomycetes	o__Helotiales	f__Helotiaceae	g__Meliniomyces	s__Meliniomyces_bicolor	OTU871
d__Eukaryota	k__Fungi	p__Ascomycota	c__Dothideomycetes	o__Pleosporales	f__Cucurbitariaceae	g__Pyrenochaeta	s__Pyrenochaeta_sp	OTU1067
d__Eukaryota	k__Fungi	p__Basidiomycota	c__Agaricomycetes	o__Cantharellales	f__Cantharellales_fam_Incertae_sedis	g__Sistotrema	s__Sistotrema_sp	OTU179
d__Eukaryota	k__Fungi	p__Ascomycota	c__Leotiomycetes	o__Helotiales	f__Sclerotiniaceae	g__unclassified_f__Sclerotiniaceae	s__unclassified_f__Sclerotiniaceae	OTU760
d__Eukaryota	k__Fungi	p__Ascomycota	c__Dothideomycetes	o__Capnodiales	f__Teratosphaeriaceae	g__Capnobotryella	s__Capnobotryella_renispora	OTU322
d__Eukaryota	k__Fungi	p__Ascomycota	c__Eurotiomycetes	o__Onygenales	f__Arthrodermataceae	g__Arthroderma	s__Arthroderma_sp	OTU1025
d__Eukaryota	k__Fungi	p__Ascomycota	c__Leotiomycetes	o__Helotiales	f__unclassified_o__Helotiales	g__unclassified_o__Helotiales	s__unclassified_o__Helotiales	OTU113
d__Eukaryota	k__Fungi	p__Basidiomycota	c__Agaricomycetes	o__Trechisporales	f__Hydnodontaceae	g__Trechispora	s__Trechispora_cohaerens	OTU873
d__Eukaryota	k__Fungi	p__Basidiomycota	c__Cystobasidiomycetes	o__Erythrobasidiales	f__Erythrobasidiaceae	g__Erythrobasidium	s__Erythrobasidium_hasegawianum	OTU1232
d__Eukaryota	k__Fungi	p__Basidiomycota	c__Tremellomycetes	o__Tremellales	f__Bulleraceae	g__Genolevuria	s__Genolevuria_sp	OTU977
d__Eukaryota	k__Fungi	p__Ascomycota	c__Dothideomycetes	o__Pleosporales	f__Didymellaceae	g__unclassified_f__Didymellaceae	s__unclassified_f__Didymellaceae	OTU315
d__Eukaryota	k__Fungi	p__Ascomycota	c__Dothideomycetes	o__Pleosporales	f__Melanommataceae	g__Melanomma	s__Melanomma_pulvis-pyrius	OTU171
d__Eukaryota	k__Fungi	p__Ascomycota	c__Eurotiomycetes	o__Eurotiales	f__Aspergillaceae	g__Penicillium	s__Penicillium_brevicompactum	OTU1043
d__Eukaryota	k__Fungi	p__Mortierellomycota	c__Mortierellomycetes	o__Mortierellales	f__Mortierellaceae	g__Mortierella	s__Mortierella_minutissima	OTU707
d__Eukaryota	k__Fungi	p__Mucoromycota	c__Mucoromycetes	o__Mucorales	f__Mucoraceae	g__Mucor	s__Mucor_corticola	OTU264

Domain	Kingdom	Phylum	Class	Order	Family	Genus	Species	OTU
d__Eukaryota	k__Fungi	p__Basidiomycota	c__Tremellomycetes	o__Filobasidiales	f__Piskurozymaceae	g__Piskurozyma	s__Piskurozyma_sp	OTU11
d__Eukaryota	k__Fungi	p__Ascomycota	c__Lecanoromycetes	o__Caliciales	f__Physciaceae	g__Physcia	s__Physcia_tribacia	OTU159
d__Eukaryota	k__Fungi	p__Ascomycota	c__Sordariomycetes	o__Hypocreales	f__Cordycipitaceae	g__Isaria	s__Isaria_cateniannulata	OTU193
d__Eukaryota	k__Fungi	p__Basidiomycota	c__Agaricomycetes	o__Trechisporales	f__Hydnodontaceae	g__Trechispora	s__Trechispora_cohaerens	OTU679
d__Eukaryota	k__Fungi	p__Chytridiomycota	c__Spizellomycetes	o__Spizellomycetales	f__unclassified_o__Spizellomycetales	g__unclassified_o__Spizellomycetales	s__Spizellomycetales_sp	OTU413
d__Eukaryota	k__Fungi	p__Basidiomycota	c__Agaricomycetes	o__Polyporales	f__Ganodermataceae	g__Perenniporia	s__Perenniporia_maackiae	OTU850
d__Eukaryota	k__Fungi	p__Basidiomycota	c__Tremellomycetes	o__Tremellales	f__Bulleribasidiaceae	g__Dioszegia	s__unclassified_g__Dioszegia	OTU291
d__Eukaryota	k__Fungi	p__Basidiomycota	c__Microbotryomycetes	o__Sporidiobolales	f__Sporidiobolaceae	g__Sporobolomyces	s__Sporobolomyces_sp	OTU343
d__Eukaryota	k__Fungi	p__Ascomycota	c__unclassified_p__Ascomycota	o__unclassified_p__Ascomycota	f__unclassified_p__Ascomycota	g__unclassified_p__Ascomycota	s__unclassified_p__Ascomycota	OTU270
d__Eukaryota	k__Fungi	p__Ascomycota	c__Sordariomycetes	o__Xylariales	f__Diatrypaceae	g__Diatrype	s__Diatrype_stigma	OTU189
d__Eukaryota	k__Fungi	p__Chytridiomycota	c__Rhizophydiomycetes	o__Rhizophydiales	f__Rhizophydiaceae	g__Rhizophydium	s__Rhizophydium_sp	OTU620
d__Eukaryota	k__Fungi	p__Ascomycota	c__unclassified_p__Ascomycota	o__unclassified_p__Ascomycota	f__unclassified_p__Ascomycota	g__unclassified_p__Ascomycota	s__unclassified_p__Ascomycota	OTU449
d__Eukaryota	k__Fungi	p__Basidiomycota	c__Tremellomycetes	o__Cystofilobasidiales	f__Mrakiaceae	g__Itersonilia	s__Itersonilia_pannonica	OTU1138
d__Eukaryota	k__Fungi	p__Ascomycota	c__Dothideomycetes	o__Pleosporales	f__unclassified_o__Pleosporales	g__unclassified_o__Pleosporales	s__unclassified_o__Pleosporales	OTU497
d__Eukaryota	k__Fungi	p__Chytridiomycota	c__unclassified_p__Chytridiomycota	o__unclassified_p__Chytridiomycota	f__unclassified_p__Chytridiomycota	g__unclassified_p__Chytridiomycota	s__unclassified_p__Chytridiomycota	OTU9
d__Eukaryota	k__Fungi	p__Ascomycota	c__Sordariomycetes	o__Pleurotheciales	f__Pleurotheciaceae	g__Sterigmatobotrys	s__Sterigmatobotrys_sp	OTU1085
d__Eukaryota	k__Fungi	p__unclassified_k__Fungi	c__unclassified_k__Fungi	o__unclassified_k__Fungi	f__unclassified_k__Fungi	g__unclassified_k__Fungi	s__unclassified_k__Fungi	OTU476
d__Eukaryota	k__Fungi	p__Ascomycota	c__Lecanoromycetes	o__Rhizocarpales	f__Rhizocarpaceae	g__Rhizocarpon	s__unclassified_g__Rhizocarpon	OTU92
d__Eukaryota	k__Fungi	p__Ascomycota	c__Dothideomycetes	o__Pleosporales	f__Leptosphaeriaceae	g__Leptosphaeria	s__Leptosphaeria_sclerotioides	OTU844
d__Eukaryota	k__Fungi	p__Basidiomycota	c__Microbotryomycetes	o__Kriegeriales	f__Camptobasidiaceae	g__Glaciozyma	s__Glaciozyma_martinii	OTU395
d__Eukaryota	k__Fungi	p__Ascomycota	c__Sordariomycetes	o__Hypocreales	f__Nectriaceae	g__Fusarium	s__Fusarium_incarnatum	OTU325
d__Eukaryota	k__Fungi	p__Ascomycota	c__unclassified_p__Ascomycota	o__unclassified_p__Ascomycota	f__unclassified_p__Ascomycota	g__unclassified_p__Ascomycota	s__unclassified_p__Ascomycota	OTU1260
d__Eukaryota	k__Fungi	p__Ascomycota	c__Eurotiomycetes	o__unclassified_c__Eurotiomycetes	f__unclassified_c__Eurotiomycetes	g__unclassified_c__Eurotiomycetes	s__unclassified_c__Eurotiomycetes	OTU145
d__Eukaryota	k__Fungi	p__unclassified_k__Fungi	c__unclassified_k__Fungi	o__unclassified_k__Fungi	f__unclassified_k__Fungi	g__unclassified_k__Fungi	s__unclassified_k__Fungi	OTU1115
d__Eukaryota	k__Fungi	p__Ascomycota	c__unclassified_p__Ascomycota	o__unclassified_p__Ascomycota	f__unclassified_p__Ascomycota	g__unclassified_p__Ascomycota	s__unclassified_p__Ascomycota	OTU882
d__Eukaryota	k__Fungi	p__Ascomycota	c__Dothideomycetes	o__Pleosporales	f__Coniothyriaceae	g__Coniothyrium	s__Coniothyrium_telephii	OTU1061
d__Eukaryota	k__Fungi	p__Ascomycota	c__Pezizomycetes	o__Pezizales	f__Sarcosomataceae	g__Strumella	s__Strumella_coryneoidea	OTU750
d__Eukaryota	k__Fungi	p__Ascomycota	c__Sordariomycetes	o__Xylariales	f__unclassified_o__Xylariales	g__unclassified_o__Xylariales	s__unclassified_o__Xylariales	OTU426
d__Eukaryota	k__Fungi	p__Ascomycota	c__Lecanoromycetes	o__Lecanorales	f__Lecanoraceae	g__Scoliciosporum	s__Scoliciosporum_umbrinum	OTU238
d__Eukaryota	k__Fungi	p__Ascomycota	c__Dothideomycetes	o__Capnodiales	f__unclassified_o__Capnodiales	g__unclassified_o__Capnodiales	s__unclassified_o__Capnodiales	OTU690
d__Eukaryota	k__Fungi	p__Ascomycota	c__Eurotiomycetes	o__Verrucariales	f__unclassified_o__Verrucariales	g__unclassified_o__Verrucariales	s__Verrucariales_sp	OTU875

Domain	Kingdom	Phylum	Class	Order	Family	Genus	Species	OTU
d__Eukaryota	k__Fungi	p__unclassified_k__Fungi	c__unclassified_k__Fungi	o__unclassified_k__Fungi	f__unclassified_k__Fungi	g__unclassified_k__Fungi	s__unclassified_k__Fungi	OTU1257
d__Eukaryota	k__Fungi	p__Monoblepharomycota	c__Sanchytriomycetes	o__Sanchytriales	f__Sanchytriaceae	g__Sanchytrium	s__Sanchytrium_sp	OTU1037
d__Eukaryota	k__Fungi	p__Basidiomycota	c__Agaricomycetes	o__Polyporales	f__Meruliaceae	g__Phlebia	s__Phlebia_livida	OTU700
d__Eukaryota	k__Fungi	p__Basidiomycota	c__Microbotryomycetes	o__Microbotryomycetes_ord_Incertae_sedis	f__Chrysozymaceae	g__Sampaiozyma	s__Sampaiozyma_sp	OTU440
d__Eukaryota	k__Fungi	p__unclassified_k__Fungi	c__unclassified_k__Fungi	o__unclassified_k__Fungi	f__unclassified_k__Fungi	g__unclassified_k__Fungi	s__unclassified_k__Fungi	OTU1152
d__Eukaryota	k__Fungi	p__Ascomycota	c__Dothideomycetes	o__Pleosporales	f__Phaeosphaeriaceae	g__Paraphoma	s__Paraphoma_fimeti	OTU398
d__Eukaryota	k__Fungi	p__Chytridiomycota	c__Spizellomycetes	o__Spizellomycetales	f__unclassified_o__Spizellomycetales	g__unclassified_o__Spizellomycetales	s__Spizellomycetales_sp	OTU858
d__Eukaryota	k__Fungi	p__Chytridiomycota	c__unclassified_p__Chytridiomycota	o__unclassified_p__Chytridiomycota	f__unclassified_p__Chytridiomycota	g__unclassified_p__Chytridiomycota	s__unclassified_p__Chytridiomycota	OTU162
d__Eukaryota	k__Fungi	p__Chytridiomycota	c__unclassified_p__Chytridiomycota	o__unclassified_p__Chytridiomycota	f__unclassified_p__Chytridiomycota	g__unclassified_p__Chytridiomycota	s__Chytridiomycota_sp	OTU209
d__Eukaryota	k__Fungi	p__Basidiomycota	c__Tremellomycetes	o__Tremellales	f__Bulleribasidiaceae	g__Hannaella	s__Hannaella_zeae	OTU305
d__Eukaryota	k__Fungi	p__Basidiomycota	c__Dacrymycetes	o__Dacrymycetales	f__Dacrymycetaceae	g__Dacrymyces	s__unclassified_g__Dacrymyces	OTU580
d__Eukaryota	k__Fungi	p__Ascomycota	c__Leotiomycetes	o__Phacidiales	f__Bulgariaceae	g__Bulgaria	s__Bulgaria_inquinans	OTU494
d__Eukaryota	k__Fungi	p__Ascomycota	c__unclassified_p__Ascomycota	o__unclassified_p__Ascomycota	f__unclassified_p__Ascomycota	g__unclassified_p__Ascomycota	s__unclassified_p__Ascomycota	OTU630
d__Eukaryota	k__Fungi	p__Ascomycota	c__Leotiomycetes	o__Helotiales	f__Helotiales_fam_Incertae_sedis	g__Coleophoma	s__Coleophoma_sp	OTU1087
d__Eukaryota	k__Fungi	p__Mortierellomycota	c__Mortierellomycetes	o__Mortierellales	f__Mortierellaceae	g__Mortierella	s__Mortierella_parvispora	OTU541
d__Eukaryota	k__Fungi	p__Ascomycota	c__Dothideomycetes	o__Pleosporales	f__unclassified_o__Pleosporales	g__unclassified_o__Pleosporales	s__Pleosporales_sp	OTU133
d__Eukaryota	k__Fungi	p__Basidiomycota	c__Agaricomycetes	o__Sebacinales	f__Serendipitaceae	g__Serendipita	s__Serendipita_sp	OTU777
d__Eukaryota	k__Fungi	p__Basidiomycota	c__Agaricomycetes	o__Hymenochaetales	f__Schizoporaceae	g__Xylodon	s__Xylodon_asperus	OTU473
d__Eukaryota	k__Fungi	p__Basidiomycota	c__Agaricomycetes	o__Polyporales	f__Polyporaceae	g__Polyporus	s__Polyporus_lipsiensis	OTU831
d__Eukaryota	k__Fungi	p__unclassified_k__Fungi	c__unclassified_k__Fungi	o__unclassified_k__Fungi	f__unclassified_k__Fungi	g__unclassified_k__Fungi	s__unclassified_k__Fungi	OTU604
d__Eukaryota	k__Fungi	p__Ascomycota	c__Leotiomycetes	o__Helotiales	f__Helotiales_fam_Incertae_sedis	g__Leohumicola	s__Leohumicola_sp	OTU983
d__Eukaryota	k__Fungi	p__Ascomycota	c__Dothideomycetes	o__Dothideales	f__Dothioraceae	g__Perusta	s__Perusta_sp	OTU32
d__Eukaryota	k__Fungi	p__Basidiomycota	c__Microbotryomycetes	o__Kriegeriales	f__Camptobasidiaceae	g__Glaciozyma	s__Glaciozyma_antarctica	OTU1064
d__Eukaryota	k__Fungi	p__Ascomycota	c__Dothideomycetes	o__Pleosporales	f__Sporormiaceae	g__Sporormiella	s__Sporormiella_megalospora	OTU467
d__Eukaryota	k__Fungi	p__Ascomycota	c__unclassified_p__Ascomycota	o__unclassified_p__Ascomycota	f__unclassified_p__Ascomycota	g__unclassified_p__Ascomycota	s__unclassified_p__Ascomycota	OTU14
d__Eukaryota	k__Fungi	p__Basidiomycota	c__Agaricomycetes	o__Agaricales	f__unclassified_o__Agaricales	g__unclassified_o__Agaricales	s__unclassified_o__Agaricales	OTU573
d__Eukaryota	k__Fungi	p__Ascomycota	c__Sordariomycetes	o__Sordariales	f__Sordariales_fam_Incertae_sedis	g__Ramophialophora	s__Ramophialophora_sp	OTU1019
d__Eukaryota	k__Fungi	p__Ascomycota	c__unclassified_p__Ascomycota	o__unclassified_p__Ascomycota	f__unclassified_p__Ascomycota	g__unclassified_p__Ascomycota	s__unclassified_p__Ascomycota	OTU17
d__Eukaryota	k__Fungi	p__unclassified_k__Fungi	c__unclassified_k__Fungi	o__unclassified_k__Fungi	f__unclassified_k__Fungi	g__unclassified_k__Fungi	s__unclassified_k__Fungi	OTU582
d__Eukaryota	k__Fungi	p__Ascomycota	c__Arthoniomycetes	o__Lichenostigmatales	f__unclassified_o__Lichenostigmatales	g__unclassified_o__Lichenostigmatales	s__Lichenostigmatales_sp	OTU74

Domain	Kingdom	Phylum	Class	Order	Family	Genus	Species	OTU
d__Eukaryota	k__Fungi	p__Ascomycota	c__Leotiomycetes	o__unclassified_c__Leotiomycetes	f__unclassified_c__Leotiomycetes	g__unclassified_c__Leotiomycetes	s__unclassified_c__Leotiomycetes	OTU531
d__Eukaryota	k__Fungi	p__unclassified_k__Fungi	c__unclassified_k__Fungi	o__unclassified_k__Fungi	f__unclassified_k__Fungi	g__unclassified_k__Fungi	s__unclassified_k__Fungi	OTU166
d__Eukaryota	k__Fungi	p__Ascomycota	c__Sordariomycetes	o__Hypocreales	f__Tilachlidiaceae	g__unclassified_f__Tilachlidiaceae	s__unclassified_f__Tilachlidiaceae	OTU783
d__Eukaryota	k__Fungi	p__Ascomycota	c__Eurotiomycetes	o__Chaetothyriales	f__Chaetothyriaceae	g__unclassified_f__Chaetothyriaceae	s__unclassified_f__Chaetothyriaceae	OTU1075
d__Eukaryota	k__Fungi	p__Ascomycota	c__Dothideomycetes	o__Pleosporales	f__unclassified_o__Pleosporales	g__unclassified_o__Pleosporales	s__Pleosporales_sp	OTU506
d__Eukaryota	k__Fungi	p__Ascomycota	c__Saccharomycetes	o__Saccharomycetales	f__Saccharomycetales_fam_Incertae_sedis	g__Candida	s__Candida_intermedia	OTU38
d__Eukaryota	k__Fungi	p__Basidiomycota	c__Agaricomycetes	o__Trechisporales	f__Hydnodontaceae	g__Trechispora	s__Trechispora_sp	OTU67
d__Eukaryota	k__Fungi	p__Ascomycota	c__Sordariomycetes	o__Sordariales	f__Chaetomiaceae	g__Dichotomopilus	s__unclassified_g__Dichotomopilus	OTU520
d__Eukaryota	k__Fungi	p__Ascomycota	c__Eurotiomycetes	o__Chaetothyriales	f__unclassified_o__Chaetothyriales	g__unclassified_o__Chaetothyriales	s__unclassified_o__Chaetothyriales	OTU177
d__Eukaryota	k__Fungi	p__Basidiomycota	c__Agaricomycetes	o__Polyporales	f__Meruliaceae	g__Bjerkandera	s__Bjerkandera_adusta	OTU655
d__Eukaryota	k__Fungi	p__Basidiomycota	c__Agaricomycetes	o__Agaricales	f__Strophariaceae	g__Hypholoma	s__Hypholoma_capnoides	OTU798
d__Eukaryota	k__Fungi	p__Ascomycota	c__Dothideomycetes	o__Pleosporales	f__unclassified_o__Pleosporales	g__unclassified_o__Pleosporales	s__unclassified_o__Pleosporales	OTU437
d__Eukaryota	k__Fungi	p__Ascomycota	c__Leotiomycetes	o__unclassified_c__Leotiomycetes	f__unclassified_c__Leotiomycetes	g__unclassified_c__Leotiomycetes	s__unclassified_c__Leotiomycetes	OTU967
d__Eukaryota	k__Fungi	p__Ascomycota	c__Sordariomycetes	o__Diaporthales	f__Valsaceae	g__Cytospora	s__Cytospora_sacculus	OTU382
d__Eukaryota	k__Fungi	p__unclassified_k__Fungi	c__unclassified_k__Fungi	o__unclassified_k__Fungi	f__unclassified_k__Fungi	g__unclassified_k__Fungi	s__unclassified_k__Fungi	OTU455
d__Eukaryota	k__Fungi	p__unclassified_k__Fungi	c__unclassified_k__Fungi	o__unclassified_k__Fungi	f__unclassified_k__Fungi	g__unclassified_k__Fungi	s__unclassified_k__Fungi	OTU450
d__Eukaryota	k__Fungi	p__unclassified_k__Fungi	c__unclassified_k__Fungi	o__unclassified_k__Fungi	f__unclassified_k__Fungi	g__unclassified_k__Fungi	s__unclassified_k__Fungi	OTU451
d__Eukaryota	k__Fungi	p__unclassified_k__Fungi	c__unclassified_k__Fungi	o__unclassified_k__Fungi	f__unclassified_k__Fungi	g__unclassified_k__Fungi	s__unclassified_k__Fungi	OTU452
d__Eukaryota	k__Fungi	p__Ascomycota	c__Eurotiomycetes	o__Eurotiales	f__Aspergillaceae	g__Aspergillus	s__unclassified_g__Aspergillus	OTU40
d__Eukaryota	k__Fungi	p__Ascomycota	c__unclassified_p__Ascomycota	o__unclassified_p__Ascomycota	f__unclassified_p__Ascomycota	g__unclassified_p__Ascomycota	s__unclassified_p__Ascomycota	OTU178
d__Eukaryota	k__Fungi	p__Ascomycota	c__Leotiomycetes	o__Helotiales	f__Helotiaceae	g__Tetracladium	s__Tetracladium_sp	OTU457
d__Eukaryota	k__Fungi	p__unclassified_k__Fungi	c__unclassified_k__Fungi	o__unclassified_k__Fungi	f__unclassified_k__Fungi	g__unclassified_k__Fungi	s__unclassified_k__Fungi	OTU298
d__Eukaryota	k__Fungi	p__Ascomycota	c__Dothideomycetes	o__Pleosporales	f__Periconiaceae	g__Periconia	s__Periconia_pseudobyssoides	OTU192
d__Eukaryota	k__Fungi	p__Basidiomycota	c__Agaricomycetes	o__Polyporales	f__Ganodermataceae	g__Ganoderma	s__unclassified_g__Ganoderma	OTU481
d__Eukaryota	k__Fungi	p__Ascomycota	c__Dothideomycetes	o__Pleosporales	f__Phaeosphaeriaceae	g__Neostagonospora	s__unclassified_g__Neostagonospora	OTU96
d__Eukaryota	k__Fungi	p__Ascomycota	c__Dothideomycetes	o__Pleosporales	f__Pleosporaceae	g__Stemphylium	s__Stemphylium_lycopersici	OTU163
d__Eukaryota	k__Fungi	p__Ascomycota	c__Dothideomycetes	o__Capnodiales	f__Mycosphaerellaceae	g__Periconiella	s__Periconiella_sp	OTU474
d__Eukaryota	k__Fungi	p__Basidiomycota	c__unclassified_p__Basidiomycota	o__unclassified_p__Basidiomycota	f__unclassified_p__Basidiomycota	g__unclassified_p__Basidiomycota	s__unclassified_p__Basidiomycota	OTU1139
d__Eukaryota	k__Fungi	p__Ascomycota	c__Sordariomycetes	o__Hypocreales	f__Nectriaceae	g__Neocosmospora	s__Neocosmospora_rubicola	OTU370
d__Eukaryota	k__Fungi	p__unclassified_k__Fungi	c__unclassified_k__Fungi	o__unclassified_k__Fungi	f__unclassified_k__Fungi	g__unclassified_k__Fungi	s__unclassified_k__Fungi	OTU808

Domain	Kingdom	Phylum	Class	Order	Family	Genus	Species	OTU
d__Eukaryota	k__Fungi	p__Ascomycota	c__GS37	o__unclassified_c__GS37	f__unclassified_c__GS37	g__unclassified_c__GS37	s__unclassified_c__GS37	OTU810
d__Eukaryota	k__Fungi	p__Ascomycota	c__Leotiomycetes	o__Helotiales	f__Helotiaceae	g__Heterosphaeria	s__Heterosphaeria_patella	OTU249
d__Eukaryota	k__Fungi	p__Ascomycota	c__Sordariomycetes	o__Trichosphaeriales	f__Trichosphaeriaceae	g__Nigrospora	s__Nigrospora_oryzae	OTU670
d__Eukaryota	k__Fungi	p__Ascomycota	c__Sordariomycetes	o__unclassified_c__Sordariomycetes	f__unclassified_c__Sordariomycetes	g__unclassified_c__Sordariomycetes	s__unclassified_c__Sordariomycetes	OTU353
d__Eukaryota	k__Fungi	p__unclassified_k__Fungi	c__unclassified_k__Fungi	o__unclassified_k__Fungi	f__unclassified_k__Fungi	g__unclassified_k__Fungi	s__unclassified_k__Fungi	OTU807
d__Eukaryota	k__Fungi	p__Ascomycota	c__Eurotiomycetes	o__Chaetothyriales	f__Chaetothyriaceae	g__unclassified_f__Chaetothyriaceae	s__unclassified_f__Chaetothyriaceae	OTU501
d__Eukaryota	k__Fungi	p__Ascomycota	c__Lecanoromycetes	o__Caliciales	f__Physciaceae	g__Phaeophyscia	s__Phaeophyscia_rubropulchra	OTU169
d__Eukaryota	k__Fungi	p__Ascomycota	c__Dothideomycetes	o__Pleosporales	f__Lophiostomataceae	g__Lophiostoma	s__Lophiostoma_macrostomum	OTU255
d__Eukaryota	k__Fungi	p__Ascomycota	c__Pezizomycotina_cls_Incertae_sedis	o__Pezizomycotina_ord_Incertae_sedis	f__Pezizomycotina_fam_Incertae_sedis	g__Ciliophora	s__Ciliophora_sp	OTU721
d__Eukaryota	k__Fungi	p__Ascomycota	c__Sordariomycetes	o__Xylariales	f__Hyponectriaceae	g__Monographella	s__Monographella_nivalis	OTU1150
d__Eukaryota	k__Fungi	p__Ascomycota	c__Orbiliomycetes	o__Orbiliales	f__Orbiliaceae	g__Retiarius	s__Retiarius_sp	OTU191
d__Eukaryota	k__Fungi	p__Ascomycota	c__Eurotiomycetes	o__Eurotiales	f__Aspergillaceae	g__Penicillium	s__Penicillium_oxalicum	OTU362
d__Eukaryota	k__Fungi	p__Ascomycota	c__Lecanoromycetes	o__Trapeliales	f__Trapeliaceae	g__Trapelia	s__Trapelia_glebulosa	OTU963
d__Eukaryota	k__Fungi	p__Basidiomycota	c__Tremellomycetes	o__Trichosporonales	f__Trichosporonaceae	g__Apiotrichum	s__Apiotrichum_dulcitum	OTU1060
d__Eukaryota	k__Fungi	p__Basidiomycota	c__Agaricomycetes	o__Cantharellales	f__Cantharellales_fam_Incertae_sedis	g__Sistotrema	s__unclassified_g__Sistotrema	OTU6
d__Eukaryota	k__Fungi	p__Basidiomycota	c__Agaricomycetes	o__Polyporales	f__Coriolaceae	g__Trametes	s__unclassified_g__Trametes	OTU218
d__Eukaryota	k__Fungi	p__Ascomycota	c__Pezizomycotina_cls_Incertae_sedis	o__Pezizomycotina_ord_Incertae_sedis	f__Pezizomycotina_fam_Incertae_sedis	g__Ciliophora	s__Ciliophora_sp	OTU484
d__Eukaryota	k__Fungi	p__Ascomycota	c__Eurotiomycetes	o__Chaetothyriales	f__Herpotrichiellaceae	g__Cladophialophora	s__unclassified_g__Cladophialophora	OTU21
d__Eukaryota	k__Fungi	p__Ascomycota	c__Dothideomycetes	o__Pleosporales	f__Pleosporaceae	g__Curvularia	s__Curvularia_nodulosa	OTU605
d__Eukaryota	k__Fungi	p__Ascomycota	c__unclassified_p__Ascomycota	o__unclassified_p__Ascomycota	f__unclassified_p__Ascomycota	g__unclassified_p__Ascomycota	s__unclassified_p__Ascomycota	OTU7
d__Eukaryota	k__Fungi	p__Ascomycota	c__Dothideomycetes	o__Pleosporales	f__Pleosporales_fam_Incertae_sedis	g__Pseudorobillarda	s__Pseudorobillarda_phragmitis	OTU1149
d__Eukaryota	k__Fungi	p__Ascomycota	c__Leotiomycetes	o__Helotiales	f__unclassified_o__Helotiales	g__unclassified_o__Helotiales	s__unclassified_o__Helotiales	OTU996
d__Eukaryota	k__Fungi	p__Ascomycota	c__Sordariomycetes	o__Xylariales	f__Xylariaceae	g__Hypoxylon	s__unclassified_g__Hypoxylon	OTU857
d__Eukaryota	k__Fungi	p__Ascomycota	c__Leotiomycetes	o__Helotiales	f__unclassified_o__Helotiales	g__unclassified_o__Helotiales	s__unclassified_o__Helotiales	OTU1230
d__Eukaryota	k__Fungi	p__Ascomycota	c__Sordariomycetes	o__Microascales	f__Microascaceae	g__Cephalotrichum	s__Cephalotrichum_nanum	OTU404
d__Eukaryota	k__Fungi	p__Basidiomycota	c__Tremellomycetes	o__Tremellales	f__Phaeotremellaceae	g__Phaeotremella	s__unclassified_g__Phaeotremella	OTU366
d__Eukaryota	k__Fungi	p__Ascomycota	c__Dothideomycetes	o__Pleosporales	f__Massarinaceae	g__unclassified_f__Massarinaceae	s__Massarinaceae_sp	OTU33
d__Eukaryota	k__Fungi	p__unclassified_k__Fungi	c__unclassified_k__Fungi	o__unclassified_k__Fungi	f__unclassified_k__Fungi	g__unclassified_k__Fungi	s__unclassified_k__Fungi	OTU112
d__Eukaryota	k__Fungi	p__unclassified_k__Fungi	c__unclassified_k__Fungi	o__unclassified_k__Fungi	f__unclassified_k__Fungi	g__unclassified_k__Fungi	s__unclassified_k__Fungi	OTU330
d__Eukaryota	k__Fungi	p__Ascomycota	c__Leotiomycetes	o__Helotiales	f__Hyaloscyphaceae	g__Cistella	s__unclassified_g__Cistella	OTU187
d__Eukaryota	k__Fungi	p__Ascomycota	c__Dothideomycetes	o__Capnodiales	f__Teratosphaeriaceae	g__unclassified_f__Teratosphaeriaceae	s__unclassified_f__Teratosphaeriaceae	OTU816

Domain	Kingdom	Phylum	Class	Order	Family	Genus	Species	OTU
d__Eukaryota	k__Fungi	p__Basidiomycota	c__Agaricomycetes	o__Atheliales	f__Atheliaceae	g__Piloderma	s__Piloderma_byssinum	OTU682
d__Eukaryota	k__Fungi	p__Ascomycota	c__Sordariomycetes	o__Xylariales	f__unclassified_o__Xylariales	g__unclassified_o__Xylariales	s__unclassified_o__Xylariales	OTU1176
d__Eukaryota	k__Fungi	p__Ascomycota	c__Sordariomycetes	o__Coniochaetales	f__Coniochaetaceae	g__unclassified_f__Coniochaetaceae	s__unclassified_f__Coniochaetaceae	OTU349
d__Eukaryota	k__Fungi	p__Fungi_phy_Incertae_sedis	c__Fungi_cls_Incertae_sedis	o__Fungi_ord_Incertae_sedis	f__Fungi_fam_Incertae_sedis	g__Desmococcus	s__Desmococcus_sp	OTU233
d__Eukaryota	k__Fungi	p__Ascomycota	c__Leotiomycetes	o__Helotiales	f__Hyaloscyphaceae	g__Clathrosphaerina	s__Clathrosphaerina_zalewskii	OTU878
d__Eukaryota	k__Fungi	p__Chytridiomycota	c__Chytridiomycetes	o__Chytridiales	f__unclassified_o__Chytridiales	g__unclassified_o__Chytridiales	s__Chytridiales_sp	OTU1234
d__Eukaryota	k__Fungi	p__Ascomycota	c__Eurotiomycetes	o__Eurotiales	f__Aspergillaceae	g__Aspergillus	s__Aspergillus_penicillioides	OTU160
d__Eukaryota	k__Fungi	p__Basidiomycota	c__Tremellomycetes	o__Tremellales	f__Bulleribasidiaceae	g__Vishniacozyma	s__Vishniacozyma_foliicola	OTU228
d__Eukaryota	k__Fungi	p__Ascomycota	c__Pezizomycotina_cls_Incertae_sedis	o__Pezizomycotina_ord_Incertae_sedis	f__Pezizomycotina_fam_Incertae_sedis	g__Ciliophora	s__Ciliophora_sp	OTU795
d__Eukaryota	k__Fungi	p__Ascomycota	c__Sordariomycetes	o__Xylariales	f__Xylariaceae	g__Xylaria	s__Xylaria_primorskensis	OTU786
d__Eukaryota	k__Fungi	p__Ascomycota	c__Pezizomycotina_cls_Incertae_sedis	o__Pezizomycotina_ord_Incertae_sedis	f__Pezizomycotina_fam_Incertae_sedis	g__Ciliophora	s__Ciliophora_sp	OTU793
d__Eukaryota	k__Fungi	p__Ascomycota	c__Lecanoromycetes	o__unclassified_c__Lecanoromycetes	f__unclassified_c__Lecanoromycetes	g__unclassified_c__Lecanoromycetes	s__unclassified_c__Lecanoromycetes	OTU784
d__Eukaryota	k__Fungi	p__Ascomycota	c__Pezizomycetes	o__unclassified_c__Pezizomycetes	f__unclassified_c__Pezizomycetes	g__unclassified_c__Pezizomycetes	s__Pezizomycetes_sp	OTU656
d__Eukaryota	k__Fungi	p__Ascomycota	c__Dothideomycetes	o__Venturiales	f__unclassified_o__Venturiales	g__unclassified_o__Venturiales	s__unclassified_o__Venturiales	OTU61
d__Eukaryota	k__Fungi	p__Basidiomycota	c__Tremellomycetes	o__Tremellales	f__Phaeotremellaceae	g__Phaeotremella	s__unclassified_g__Phaeotremella	OTU1200
d__Eukaryota	k__Fungi	p__Ascomycota	c__unclassified_p__Ascomycota	o__unclassified_p__Ascomycota	f__unclassified_p__Ascomycota	g__unclassified_p__Ascomycota	s__unclassified_p__Ascomycota	OTU1219
d__Eukaryota	k__Fungi	p__Ascomycota	c__unclassified_p__Ascomycota	o__unclassified_p__Ascomycota	f__unclassified_p__Ascomycota	g__unclassified_p__Ascomycota	s__unclassified_p__Ascomycota	OTU478
d__Eukaryota	k__Fungi	p__Ascomycota	c__Dothideomycetes	o__Capnodiales	f__Cladosporiaceae	g__Rachicladosporium	s__unclassified_g__Rachicladosporium	OTU153
d__Eukaryota	k__Fungi	p__Ascomycota	c__Sordariomycetes	o__Hypocreales	f__Nectriaceae	g__unclassified_f__Nectriaceae	s__unclassified_f__Nectriaceae	OTU441
d__Eukaryota	k__Fungi	p__Ascomycota	c__Dothideomycetes	o__Venturiales	f__Sympoventuriaceae	g__Ochroconis	s__unclassified_g__Ochroconis	OTU1148
d__Eukaryota	k__Fungi	p__Ascomycota	c__unclassified_p__Ascomycota	o__unclassified_p__Ascomycota	f__unclassified_p__Ascomycota	g__unclassified_p__Ascomycota	s__unclassified_p__Ascomycota	OTU852
d__Eukaryota	k__Fungi	p__unclassified_k__Fungi	c__unclassified_k__Fungi	o__unclassified_k__Fungi	f__unclassified_k__Fungi	g__unclassified_k__Fungi	s__unclassified_k__Fungi	OTU1243
d__Eukaryota	k__Fungi	p__Ascomycota	c__Dothideomycetes	o__Venturiales	f__Venturiaceae	g__Venturia	s__unclassified_g__Venturia	OTU941
d__Eukaryota	k__Fungi	p__unclassified_k__Fungi	c__unclassified_k__Fungi	o__unclassified_k__Fungi	f__unclassified_k__Fungi	g__unclassified_k__Fungi	s__unclassified_k__Fungi	OTU381
d__Eukaryota	k__Fungi	p__Ascomycota	c__Leotiomycetes	o__Helotiales	f__Helotiales_fam_Incertae_sedis	g__Coleophoma	s__Coleophoma_sp	OTU756
d__Eukaryota	k__Fungi	p__Basidiomycota	c__Tremellomycetes	o__Tremellales	f__Bulleribasidiaceae	g__Hannaella	s__Hannaella_luteola	OTU287
d__Eukaryota	k__Fungi	p__Ascomycota	c__Sordariomycetes	o__Hypocreales	f__Nectriaceae	g__Fusicolla	s__Fusicolla_aquaeductuum	OTU365
d__Eukaryota	k__Fungi	p__Ascomycota	c__Sordariomycetes	o__Hypocreales	f__Nectriaceae	g__Gibberella	s__Gibberella_tricincta	OTU239
d__Eukaryota	k__Fungi	p__Ascomycota	c__Pezizomycetes	o__Pezizales	f__Sarcosomataceae	g__Urnula	s__Urnula_craterium	OTU778
d__Eukaryota	k__Fungi	p__Ascomycota	c__Eurotiomycetes	o__Chaetothyriales	f__Herpotrichiellaceae	g__Cladophialophora	s__Cladophialophora_minutissima	OTU460
d__Eukaryota	k__Fungi	p__Mortierellomycota	c__Mortierellomycetes	o__Mortierellales	f__Mortierellaceae	g__Mortierella	s__Mortierella_sarnyensis	OTU587

Domain	Kingdom	Phylum	Class	Order	Family	Genus	Species	OTU
d__Eukaryota	k__Fungi	p__Ascomycota	c__Sordariomycetes	o__Pleurotheciales	f__Pleurotheciaceae	g__Pleurotheciella	s__Pleurotheciella_sp	OTU1110
d__Eukaryota	k__Fungi	p__Ascomycota	c__Leotiomycetes	o__Helotiales	f__Helotiaceae	g__Tetracladium	s__Tetracladium_sp	OTU635
d__Eukaryota	k__Fungi	p__unclassified_k__Fungi	c__unclassified_k__Fungi	o__unclassified_k__Fungi	f__unclassified_k__Fungi	g__unclassified_k__Fungi	s__unclassified_k__Fungi	OTU599
d__Eukaryota	k__Fungi	p__Ascomycota	c__Orbiliomycetes	o__Orbiliales	f__Orbiliales_fam_Incertae_sedis	g__Lecophagus	s__unclassified_g__Lecophagus	OTU1185
d__Eukaryota	k__Fungi	p__Basidiomycota	c__Tremellomycetes	o__Tremellales	f__Bulleribasidiaceae	g__Vishniacozyma	s__Vishniacozyma_victoriae	OTU403
d__Eukaryota	k__Fungi	p__Basidiomycota	c__Agaricomycetes	o__Trechisporales	f__Hydnodontaceae	g__Trechispora	s__Trechispora_sp	OTU363
d__Eukaryota	k__Fungi	p__Ascomycota	c__Dothideomycetes	o__Capnodiales	f__unclassified_o__Capnodiales	g__unclassified_o__Capnodiales	s__unclassified_o__Capnodiales	OTU740
d__Eukaryota	k__Fungi	p__Ascomycota	c__Taphrinomycetes	o__Taphrinales	f__Taphrinaceae	g__Taphrina	s__unclassified_g__Taphrina	OTU985
d__Eukaryota	k__Fungi	p__Basidiomycota	c__Agaricomycetes	o__Thelephorales	f__Thelephoraceae	g__unclassified_f__Thelephoraceae	s__unclassified_f__Thelephoraceae	OTU483
d__Eukaryota	k__Fungi	p__Basidiomycota	c__Agaricomycetes	o__Trechisporales	f__unclassified_o__Trechisporales	g__unclassified_o__Trechisporales	s__Trechisporales_sp	OTU706
d__Eukaryota	k__Fungi	p__Ascomycota	c__Sordariomycetes	o__Hypocreales	f__Nectriaceae	g__Fusarium	s__Fusarium_ciliatum	OTU360
d__Eukaryota	k__Fungi	p__Basidiomycota	c__Agaricomycetes	o__Boletales	f__Suillaceae	g__Suillus	s__Suillus_luteus	OTU161
d__Eukaryota	k__Fungi	p__Ascomycota	c__unclassified_p__Ascomycota	o__unclassified_p__Ascomycota	f__unclassified_p__Ascomycota	g__unclassified_p__Ascomycota	s__unclassified_p__Ascomycota	OTU371
d__Eukaryota	k__Fungi	p__Ascomycota	c__Sordariomycetes	o__Hypocreales	f__Bionectriaceae	g__Gliomastix	s__Gliomastix_cerealis	OTU1105
d__Eukaryota	k__Fungi	p__Ascomycota	c__Dothideomycetes	o__Pleosporales	f__Phaeosphaeriaceae	g__Phaeosphaeria	s__Phaeosphaeria_juncophila	OTU318
d__Eukaryota	k__Fungi	p__Ascomycota	c__Dothideomycetes	o__Pleosporales	f__Phaeosphaeriaceae	g__Juncaceicola	s__Juncaceicola_sp	OTU887
d__Eukaryota	k__Fungi	p__Ascomycota	c__Sordariomycetes	o__Hypocreales	f__Ophiocordycipitaceae	g__Tolypocladium	s__unclassified_g__Tolypocladium	OTU763
d__Eukaryota	k__Fungi	p__Ascomycota	c__Sordariomycetes	o__Xylariales	f__Microdochiaceae	g__Microdochium	s__Microdochium_phragmitis	OTU1178
d__Eukaryota	k__Fungi	p__Ascomycota	c__Sordariomycetes	o__Xylariales	f__Apiosporaceae	g__Apiospora	s__Apiospora_montagnei	OTU589
d__Eukaryota	k__Fungi	p__Ascomycota	c__Sordariomycetes	o__Hypocreales	f__Hypocreales_fam_Incertae_sedis	g__Ustilaginoidea	s__Ustilaginoidea_sp	OTU339
d__Eukaryota	k__Fungi	p__Ascomycota	c__Eurotiomycetes	o__Chaetothyriales	f__unclassified_o__Chaetothyriales	g__unclassified_o__Chaetothyriales	s__Chaetothyriales_sp	OTU955
d__Eukaryota	k__Fungi	p__Fungi_phy_Incertae_sedis	c__Fungi_cls_Incertae_sedis	o__Fungi_ord_Incertae_sedis	f__Fungi_fam_Incertae_sedis	g__Desmococcus	s__Desmococcus_sp	OTU128
d__Eukaryota	k__Fungi	p__Ascomycota	c__Pezizomycotina_cls_Incertae_sedis	o__Pezizomycotina_ord_Incertae_sedis	f__Pezizomycotina_fam_Incertae_sedis	g__Ciliophora	s__Ciliophora_sp	OTU536
d__Eukaryota	k__Fungi	p__Ascomycota	c__Pezizomycotina_cls_Incertae_sedis	o__Pezizomycotina_ord_Incertae_sedis	f__Pezizomycotina_fam_Incertae_sedis	g__Ciliophora	s__Ciliophora_sp	OTU537
d__Eukaryota	k__Fungi	p__Ascomycota	c__Sordariomycetes	o__Hypocreales	f__Hypocreaceae	g__Trichoderma	s__Trichoderma_paraviridescens	OTU524
d__Eukaryota	k__Fungi	p__Ascomycota	c__unclassified_p__Ascomycota	o__unclassified_p__Ascomycota	f__unclassified_p__Ascomycota	g__unclassified_p__Ascomycota	s__unclassified_p__Ascomycota	OTU609
d__Eukaryota	k__Fungi	p__Basidiomycota	c__Tremellomycetes	o__Tremellales	f__Phaeotremellaceae	g__Gelidatrema	s__Gelidatrema_sp	OTU230
d__Eukaryota	k__Fungi	p__Ascomycota	c__Eurotiomycetes	o__Chaetothyriales	f__Trichomeriaceae	g__Knufia	s__Knufia_sp	OTU306
d__Eukaryota	k__Fungi	p__Mortierellomycota	c__Mortierellomycetes	o__Mortierellales	f__Mortierellaceae	g__Mortierella	s__Mortierella_sclerotiella	OTU603
d__Eukaryota	k__Fungi	p__Ascomycota	c__Eurotiomycetes	o__Eurotiales	f__Aspergillaceae	g__Penicillium	s__unclassified_g__Penicillium	OTU672
d__Eukaryota	k__Fungi	p__Ascomycota	c__Leotiomycetes	o__Helotiales	f__Hyaloscyphaceae	g__Cistella	s__Cistella_sp	OTU396

Domain	Kingdom	Phylum	Class	Order	Family	Genus	Species	OTU
d__Eukaryota	k__Fungi	p__Basidiomycota	c__unclassified_p__Basidiomycota	o__unclassified_p__Basidiomycota	f__unclassified_p__Basidiomycota	g__unclassified_p__Basidiomycota	s__unclassified_p__Basidiomycota	OTU939
d__Eukaryota	k__Fungi	p__Ascomycota	c__Lecanoromycetes	o__Acarosporales	f__Acarosporaceae	g__unclassified_f__Acarosporaceae	s__unclassified_f__Acarosporaceae	OTU674
d__Eukaryota	k__Fungi	p__Ascomycota	c__Leotiomycetes	o__Helotiales	f__Helotiales_fam_Incertae_sedis	g__Coleophoma	s__Coleophoma_sp	OTU701
d__Eukaryota	k__Fungi	p__Ascomycota	c__Leotiomycetes	o__Helotiales	f__unclassified_o__Helotiales	g__unclassified_o__Helotiales	s__unclassified_o__Helotiales	OTU840
d__Eukaryota	k__Fungi	p__Ascomycota	c__Leotiomycetes	o__Helotiales	f__Leotiaceae	g__Neobulgaria	s__Neobulgaria_sp	OTU495
d__Eukaryota	k__Fungi	p__Ascomycota	c__Dothideomycetes	o__Pleosporales	f__Dictyosporiaceae	g__Pseudocoleophoma	s__Pseudocoleophoma_calamagrostidis	OTU757
d__Eukaryota	k__Fungi	p__Ascomycota	c__Eurotiomycetes	o__Chaetothyriales	f__Trichomeriaceae	g__Bradymyces	s__Bradymyces_alpinus	OTU429
d__Eukaryota	k__Fungi	p__Ascomycota	c__unclassified_p__Ascomycota	o__unclassified_p__Ascomycota	f__unclassified_p__Ascomycota	g__unclassified_p__Ascomycota	s__unclassified_p__Ascomycota	OTU286
d__Eukaryota	k__Fungi	p__Ascomycota	c__Sordariomycetes	o__Sordariales	f__Sordariales_fam_Incertae_sedis	g__Ramophialophora	s__Ramophialophora_humicola	OTU640
d__Eukaryota	k__Fungi	p__Ascomycota	c__Sordariomycetes	o__Microascales	f__unclassified_o__Microascales	g__unclassified_o__Microascales	s__unclassified_o__Microascales	OTU866
d__Eukaryota	k__Fungi	p__Ascomycota	c__Eurotiomycetes	o__Chaetothyriales	f__unclassified_o__Chaetothyriales	g__unclassified_o__Chaetothyriales	s__Chaetothyriales_sp	OTU841
d__Eukaryota	k__Fungi	p__unclassified_k__Fungi	c__unclassified_k__Fungi	o__unclassified_k__Fungi	f__unclassified_k__Fungi	g__unclassified_k__Fungi	s__unclassified_k__Fungi	OTU704
d__Eukaryota	k__Fungi	p__Basidiomycota	c__Agaricomycetes	o__Boletales	f__Serpulaceae	g__Serpula	s__Serpula_himantioides	OTU543
d__Eukaryota	k__Fungi	p__Fungi_phy_Incertae_sedis	c__Fungi_cls_Incertae_sedis	o__Fungi_ord_Incertae_sedis	f__Fungi_fam_Incertae_sedis	g__Desmococcus	s__Desmococcus_sp	OTU1213
d__Eukaryota	k__Fungi	p__Basidiomycota	c__Agaricomycetes	o__Trechisporales	f__Hydnodontaceae	g__Trechispora	s__unclassified_g__Trechispora	OTU564
d__Eukaryota	k__Fungi	p__Ascomycota	c__unclassified_p__Ascomycota	o__unclassified_p__Ascomycota	f__unclassified_p__Ascomycota	g__unclassified_p__Ascomycota	s__unclassified_p__Ascomycota	OTU127
d__Eukaryota	k__Fungi	p__Ascomycota	c__Eurotiomycetes	o__Eurotiales	f__Trichocomaceae	g__Talaromyces	s__Talaromyces_aurantiacus	OTU579
d__Eukaryota	k__Fungi	p__Basidiomycota	c__Tremellomycetes	o__Tremellales	f__Trimorphomycetaceae	g__Saitozyma	s__Saitozyma_flava	OTU377
d__Eukaryota	k__Fungi	p__Ascomycota	c__Dothideomycetes	o__Pleosporales	f__Leptosphaeriaceae	g__unclassified_f__Leptosphaeriaceae	s__unclassified_f__Leptosphaeriaceae	OTU205
d__Eukaryota	k__Fungi	p__Ascomycota	c__Dothideomycetes	o__Pleosporales	f__Didymellaceae	g__unclassified_f__Didymellaceae	s__unclassified_f__Didymellaceae	OTU274
d__Eukaryota	k__Fungi	p__Basidiomycota	c__Agaricomycetes	o__Russulales	f__Russulales_fam_Incertae_sedis	g__Aleurocystidiellum	s__Aleurocystidiellum_subcruentatum	OTU654
d__Eukaryota	k__Fungi	p__Ascomycota	c__Sordariomycetes	o__Hypocreales	f__Nectriaceae	g__Neonectria	s__Neonectria_candida	OTU1171
d__Eukaryota	k__Fungi	p__Ascomycota	c__Sordariomycetes	o__Hypocreales	f__Nectriaceae	g__Lasionectria	s__Lasionectria_sp	OTU1206
d__Eukaryota	k__Fungi	p__unclassified_k__Fungi	c__unclassified_k__Fungi	o__unclassified_k__Fungi	f__unclassified_k__Fungi	g__unclassified_k__Fungi	s__unclassified_k__Fungi	OTU1261
d__Eukaryota	k__Fungi	p__Ascomycota	c__Sordariomycetes	o__Sordariales	f__Sordariales_fam_Incertae_sedis	g__Ramophialophora	s__Ramophialophora_sp	OTU1077
d__Eukaryota	k__Fungi	p__Ascomycota	c__Eurotiomycetes	o__Eurotiales	f__Aspergillaceae	g__Penicillium	s__unclassified_g__Penicillium	OTU1039
d__Eukaryota	k__Fungi	p__unclassified_k__Fungi	c__unclassified_k__Fungi	o__unclassified_k__Fungi	f__unclassified_k__Fungi	g__unclassified_k__Fungi	s__unclassified_k__Fungi	OTU1264
d__Eukaryota	k__Fungi	p__Ascomycota	c__Dothideomycetes	o__Pleosporales	f__Pleosporaceae	g__Setosphaeria	s__Setosphaeria_sp	OTU1254
d__Eukaryota	k__Fungi	p__unclassified_k__Fungi	c__unclassified_k__Fungi	o__unclassified_k__Fungi	f__unclassified_k__Fungi	g__unclassified_k__Fungi	s__unclassified_k__Fungi	OTU1101
d__Eukaryota	k__Fungi	p__Ascomycota	c__Dothideomycetes	o__Pleosporales	f__Amniculicolaceae	g__Murispora	s__Murispora_cicognanii	OTU1141
d__Eukaryota	k__Fungi	p__Ascomycota	c__Leotiomycetes	o__Helotiales	f__Helotiaceae	g__Meliniomyces	s__Meliniomyces_bicolor	OTU937

Domain	Kingdom	Phylum	Class	Order	Family	Genus	Species	OTU
d__Eukaryota	k__Fungi	p__Ascomycota	c__Eurotiomycetes	o__Chaetothyriales	f__Trichomeriaceae	g__Knufia	s__Knufia_sp	OTU1233
d__Eukaryota	k__Fungi	p__Basidiomycota	c__Tremellomycetes	o__Filobasidiales	f__Piskurozymaceae	g__Piskurozyma	s__Piskurozyma_sp	OTU388
d__Eukaryota	k__Fungi	p__unclassified_k__Fungi	c__unclassified_k__Fungi	o__unclassified_k__Fungi	f__unclassified_k__Fungi	g__unclassified_k__Fungi	s__unclassified_k__Fungi	OTU1063
d__Eukaryota	k__Fungi	p__Ascomycota	c__unclassified_p__Ascomycota	o__unclassified_p__Ascomycota	f__unclassified_p__Ascomycota	g__unclassified_p__Ascomycota	s__unclassified_p__Ascomycota	OTU54
d__Eukaryota	k__Fungi	p__Basidiomycota	c__Agaricomycetes	o__Cantharellales	f__Ceratobasidiaceae	g__unclassified_f__Ceratobasidiaceae	s__Ceratobasidiaceae_sp	OTU485
d__Eukaryota	k__Fungi	p__unclassified_k__Fungi	c__unclassified_k__Fungi	o__unclassified_k__Fungi	f__unclassified_k__Fungi	g__unclassified_k__Fungi	s__unclassified_k__Fungi	OTU1066
d__Eukaryota	k__Fungi	p__Ascomycota	c__unclassified_p__Ascomycota	o__unclassified_p__Ascomycota	f__unclassified_p__Ascomycota	g__unclassified_p__Ascomycota	s__unclassified_p__Ascomycota	OTU237
d__Eukaryota	k__Fungi	p__Ascomycota	c__unclassified_p__Ascomycota	o__unclassified_p__Ascomycota	f__unclassified_p__Ascomycota	g__unclassified_p__Ascomycota	s__unclassified_p__Ascomycota	OTU236
d__Eukaryota	k__Fungi	p__Ascomycota	c__unclassified_p__Ascomycota	o__unclassified_p__Ascomycota	f__unclassified_p__Ascomycota	g__unclassified_p__Ascomycota	s__unclassified_p__Ascomycota	OTU235
d__Eukaryota	k__Fungi	p__Ascomycota	c__Pezizomycetes	o__Pezizales	f__Pezizaceae	g__Peziza	s__Peziza_howsei	OTU1210
d__Eukaryota	k__Fungi	p__Ascomycota	c__Leotiomycetes	o__Helotiales	f__unclassified_o__Helotiales	g__unclassified_o__Helotiales	s__unclassified_o__Helotiales	OTU30
d__Eukaryota	k__Fungi	p__Ascomycota	c__Pezizomycetes	o__Pezizales	f__Pezizaceae	g__Oedocephalum	s__unclassified_g__Oedocephalum	OTU1015
d__Eukaryota	k__Fungi	p__unclassified_k__Fungi	c__unclassified_k__Fungi	o__unclassified_k__Fungi	f__unclassified_k__Fungi	g__unclassified_k__Fungi	s__unclassified_k__Fungi	OTU410
d__Eukaryota	k__Fungi	p__Ascomycota	c__Leotiomycetes	o__Helotiales	f__unclassified_o__Helotiales	g__unclassified_o__Helotiales	s__unclassified_o__Helotiales	OTU85
d__Eukaryota	k__Fungi	p__Rozellomycota	c__unclassified_p__Rozellomycota	o__unclassified_p__Rozellomycota	f__unclassified_p__Rozellomycota	g__unclassified_p__Rozellomycota	s__Rozellomycota_sp	OTU1094
d__Eukaryota	k__Fungi	p__unclassified_k__Fungi	c__unclassified_k__Fungi	o__unclassified_k__Fungi	f__unclassified_k__Fungi	g__unclassified_k__Fungi	s__unclassified_k__Fungi	OTU416
d__Eukaryota	k__Fungi	p__Mortierellomycota	c__unclassified_p__Mortierellomycota	o__unclassified_p__Mortierellomycota	f__unclassified_p__Mortierellomycota	g__unclassified_p__Mortierellomycota	s__Mortierellomycota_sp	OTU1072
d__Eukaryota	k__Fungi	p__unclassified_k__Fungi	c__unclassified_k__Fungi	o__unclassified_k__Fungi	f__unclassified_k__Fungi	g__unclassified_k__Fungi	s__unclassified_k__Fungi	OTU1255
d__Eukaryota	k__Fungi	p__unclassified_k__Fungi	c__unclassified_k__Fungi	o__unclassified_k__Fungi	f__unclassified_k__Fungi	g__unclassified_k__Fungi	s__unclassified_k__Fungi	OTU419
d__Eukaryota	k__Fungi	p__Mortierellomycota	c__Mortierellomycetes	o__Mortierellales	f__Mortierellaceae	g__Mortierella	s__Mortierella_elongata	OTU653
d__Eukaryota	k__Fungi	p__Mortierellomycota	c__unclassified_p__Mortierellomycota	o__unclassified_p__Mortierellomycota	f__unclassified_p__Mortierellomycota	g__unclassified_p__Mortierellomycota	s__Mortierellomycota_sp	OTU1079
d__Eukaryota	k__Fungi	p__Ascomycota	c__Dothideomycetes	o__Pleosporales	f__Didymosphaeriaceae	g__Kalmusia	s__Kalmusia_sp	OTU694
d__Eukaryota	k__Fungi	p__Basidiomycota	c__Agaricomycetes	o__Boletales	f__Suillaceae	g__Suillus	s__Suillus_grevillei	OTU633
d__Eukaryota	k__Fungi	p__Basidiomycota	c__Microbotryomycetes	o__Leucosporidiales	f__Leucosporidiaceae	g__Leucosporidium	s__unclassified_g__Leucosporidium	OTU348
d__Eukaryota	k__Fungi	p__Ascomycota	c__Eurotiomycetes	o__Chaetothyriales	f__Herpotrichiellaceae	g__Coniosporium	s__Coniosporium_sp	OTU1240
d__Eukaryota	k__Fungi	p__Ascomycota	c__Dothideomycetes	o__Capnodiales	f__Mycosphaerellaceae	g__unclassified_f__Mycosphaerellaceae	s__unclassified_f__Mycosphaerellaceae	OTU915
d__Eukaryota	k__Fungi	p__Monoblepharomycota	c__Sanchytriomycetes	o__Sanchytriales	f__Sanchytriaceae	g__Sanchytrium	s__Sanchytrium_sp	OTU1249
d__Eukaryota	k__Fungi	p__Ascomycota	c__Dothideomycetes	o__Dothideales	f__Dothioraceae	g__Sydowia	s__Sydowia_sp	OTU56
d__Eukaryota	k__Fungi	p__Ascomycota	c__Dothideomycetes	o__Mytilinidiales	f__unclassified_o__Mytilinidiales	g__unclassified_o__Mytilinidiales	s__Mytilinidiales_sp	OTU384
d__Eukaryota	k__Fungi	p__Ascomycota	c__Sordariomycetes	o__Magnaporthales	f__Magnaporthaceae	g__Slopeiomyces	s__Slopeiomyces_cylindrosporus	OTU1160
d__Eukaryota	k__Fungi	p__Ascomycota	c__Leotiomycetes	o__Helotiales	f__unclassified_o__Helotiales	g__unclassified_o__Helotiales	s__unclassified_o__Helotiales	OTU965

**Domain**	**Kingdom**	**Phylum**	**Class**	**Order**	**Family**	**Genus**	**Species**	**OTU**
d__Eukaryota	k__Fungi	p__Ascomycota	c__Sordariomycetes	o__Hypocreales	f__unclassified_o__Hypocreales	g__unclassified_o__Hypocreales	s__unclassified_o__Hypocreales	OTU862
d__Eukaryota	k__Fungi	p__Ascomycota	c__Lecanoromycetes	o__Lecanorales	f__Micareaceae	g__Micarea	s__Micarea_lithinella	OTU1
d__Eukaryota	k__Fungi	p__Ascomycota	c__Sordariomycetes	o__Hypocreales	f__unclassified_o__Hypocreales	g__unclassified_o__Hypocreales	s__unclassified_o__Hypocreales	OTU861
d__Eukaryota	k__Fungi	p__Ascomycota	c__Leotiomycetes	o__Thelebolales	f__Pseudeurotiaceae	g__unclassified_f__Pseudeurotiaceae	s__unclassified_f__Pseudeurotiaceae	OTU931
d__Eukaryota	k__Fungi	p__Ascomycota	c__Leotiomycetes	o__Helotiales	f__Helotiaceae	g__unclassified_f__Helotiaceae	s__unclassified_f__Helotiaceae	OTU872
d__Eukaryota	k__Fungi	p__Ascomycota	c__Leotiomycetes	o__Helotiales	f__unclassified_o__Helotiales	g__unclassified_o__Helotiales	s__unclassified_o__Helotiales	OTU814
d__Eukaryota	k__Fungi	p__Basidiomycota	c__Agaricomycetes	o__Trechisporales	f__Hydnodontaceae	g__Trechispora	s__Trechispora_hymenocystis	OTU797
d__Eukaryota	k__Fungi	p__Ascomycota	c__Dothideomycetes	o__Capnodiales	f__Cladosporiaceae	g__Rachicladosporium	s__Rachicladosporium_sp	OTU199
d__Eukaryota	k__Fungi	p__Basidiomycota	c__Tremellomycetes	o__Cystofilobasidiales	f__Mrakiaceae	g__Mrakia	s__Mrakia_aquatica	OTU167
d__Eukaryota	k__Fungi	p__Ascomycota	c__Dothideomycetes	o__Pleosporales	f__unclassified_o__Pleosporales	g__unclassified_o__Pleosporales	s__unclassified_o__Pleosporales	OTU758
d__Eukaryota	k__Fungi	p__Ascomycota	c__Leotiomycetes	o__Helotiales	f__Hyaloscyphaceae	g__Hyalopeziza	s__Hyalopeziza_sp	OTU412
d__Eukaryota	k__Fungi	p__Basidiomycota	c__Agaricostilbomycetes	o__Agaricostilbales	f__Kondoaceae	g__unclassified_f__Kondoaceae	s__unclassified_f__Kondoaceae	OTU326
d__Eukaryota	k__Fungi	p__Ascomycota	c__Dothideomycetes	o__Capnodiales	f__Teratosphaeriaceae	g__Capnobotryella	s__Capnobotryella_sp	OTU312
d__Eukaryota	k__Fungi	p__unclassified_k__Fungi	c__unclassified_k__Fungi	o__unclassified_k__Fungi	f__unclassified_k__Fungi	g__unclassified_k__Fungi	s__unclassified_k__Fungi	OTU202
d__Eukaryota	k__Fungi	p__Ascomycota	c__Sordariomycetes	o__Hypocreales	f__Hypocreaceae	g__Trichoderma	s__Trichoderma_afroharzianum	OTU1181
d__Eukaryota	k__Fungi	p__unclassified_k__Fungi	c__unclassified_k__Fungi	o__unclassified_k__Fungi	f__unclassified_k__Fungi	g__unclassified_k__Fungi	s__unclassified_k__Fungi	OTU208
d__Eukaryota	k__Fungi	p__Ascomycota	c__Geoglossomycetes	o__Geoglossales	f__Geoglossaceae	g__Sarcoleotia	s__Sarcoleotia_globosa	OTU886
d__Eukaryota	k__Fungi	p__Ascomycota	c__Leotiomycetes	o__Helotiales	f__Hyaloscyphaceae	g__Lachnum	s__Lachnum_sp	OTU1170
d__Eukaryota	k__Fungi	p__Ascomycota	c__Lecanoromycetes	o__Lecanorales	f__Lecanoraceae	g__Lecanora	s__Lecanora_formosa	OTU77
d__Eukaryota	k__Fungi	p__Ascomycota	c__Eurotiomycetes	o__Eurotiales	f__Trichocomaceae	g__Talaromyces	s__unclassified_g__Talaromyces	OTU60
d__Eukaryota	k__Fungi	p__Ascomycota	c__Dothideomycetes	o__Pleosporales	f__Phaeosphaeriaceae	g__Ophiosphaerella	s__Ophiosphaerella_sp	OTU811
d__Eukaryota	k__Fungi	p__Ascomycota	c__Dothideomycetes	o__Pleosporales	f__Coniothyriaceae	g__Coniothyrium	s__unclassified_g__Coniothyrium	OTU269
d__Eukaryota	k__Fungi	p__Ascomycota	c__Sordariomycetes	o__Hypocreales	f__Nectriaceae	g__Paracremonium	s__Paracremonium_sp	OTU415
d__Eukaryota	k__Fungi	p__Basidiomycota	c__Agaricomycetes	o__unclassified_c__Agaricomycetes	f__unclassified_c__Agaricomycetes	g__unclassified_c__Agaricomycetes	s__unclassified_c__Agaricomycetes	OTU765
d__Eukaryota	k__Fungi	p__Basidiomycota	c__Tremellomycetes	o__Tremellales	f__Bulleribasidiaceae	g__Hannaella	s__Hannaella_oryzae	OTU331
d__Eukaryota	k__Fungi	p__Ascomycota	c__Sordariomycetes	o__unclassified_c__Sordariomycetes	f__unclassified_c__Sordariomycetes	g__unclassified_c__Sordariomycetes	s__unclassified_c__Sordariomycetes	OTU922
d__Eukaryota	k__Fungi	p__Ascomycota	c__Dothideomycetes	o__Pleosporales	f__Melanommataceae	g__Herpotrichia	s__Herpotrichia_sp	OTU1120
d__Eukaryota	k__Fungi	p__Ascomycota	c__Dothideomycetes	o__Venturiales	f__Venturiaceae	g__unclassified_f__Venturiaceae	s__unclassified_f__Venturiaceae	OTU898
d__Eukaryota	k__Fungi	p__Ascomycota	c__Dothideomycetes	o__Pleosporales	f__Phaeosphaeriaceae	g__Paraphoma	s__Paraphoma_fimeti	OTU1109
d__Eukaryota	k__Fungi	p__Ascomycota	c__Eurotiomycetes	o__Chaetothyriales	f__unclassified_o__Chaetothyriales	g__unclassified_o__Chaetothyriales	s__unclassified_o__Chaetothyriales	OTU732
d__Eukaryota	k__Fungi	p__Ascomycota	c__Dothideomycetes	o__Pleosporales	f__Didymosphaeriaceae	g__unclassified_f__Didymosphaeriaceae	s__unclassified_f__Didymosphaeriaceae	OTU1146

Domain	Kingdom	Phylum	Class	Order	Family	Genus	Species	OTU
d__Eukaryota	k__Fungi	p__Basidiomycota	c__Agaricomycetes	o__Russulales	f__Peniophoraceae	g__Peniophora	s__Peniophora_limitata	OTU154
d__Eukaryota	k__Fungi	p__Ascomycota	c__unclassified_p__Ascomycota	o__unclassified_p__Ascomycota	f__unclassified_p__Ascomycota	g__unclassified_p__Ascomycota	s__unclassified_p__Ascomycota	OTU1154
d__Eukaryota	k__Fungi	p__Basidiomycota	c__Tremellomycetes	o__Tremellales	f__unclassified_o__Tremellales	g__unclassified_o__Tremellales	s__unclassified_o__Tremellales	OTU392
d__Eukaryota	k__Fungi	p__Basidiomycota	c__Agaricomycetes	o__Agaricales	f__Strophariaceae	g__Hypholoma	s__Hypholoma_fasciculare	OTU556
d__Eukaryota	k__Fungi	p__Basidiomycota	c__Tremellomycetes	o__Tremellales	f__Bulleribasidiaceae	g__Vishniacozyma	s__Vishniacozyma_psychrotolerans	OTU448
d__Eukaryota	k__Fungi	p__Ascomycota	c__unclassified_p__Ascomycota	o__unclassified_p__Ascomycota	f__unclassified_p__Ascomycota	g__unclassified_p__Ascomycota	s__unclassified_p__Ascomycota	OTU959
d__Eukaryota	k__Fungi	p__Glomeromycota	c__Glomeromycetes	o__Diversisporales	f__unclassified_o__Diversisporales	g__unclassified_o__Diversisporales	s__Diversisporales_sp	OTU1121
d__Eukaryota	k__Fungi	p__Ascomycota	c__Leotiomycetes	o__unclassified_c__Leotiomycetes	f__unclassified_c__Leotiomycetes	g__unclassified_c__Leotiomycetes	s__unclassified_c__Leotiomycetes	OTU144
d__Eukaryota	k__Fungi	p__Ascomycota	c__Sordariomycetes	o__Coniochaetales	f__Coniochaetaceae	g__Coniochaeta	s__Coniochaeta_sp	OTU241
d__Eukaryota	k__Fungi	p__Ascomycota	c__Eurotiomycetes	o__Chaetothyriales	f__unclassified_o__Chaetothyriales	g__unclassified_o__Chaetothyriales	s__Chaetothyriales_sp	OTU992
d__Eukaryota	k__Fungi	p__Ascomycota	c__Eurotiomycetes	o__Chaetothyriales	f__Trichomeriaceae	g__Bradymyces	s__unclassified_g__Bradymyces	OTU279
d__Eukaryota	k__Fungi	p__Ascomycota	c__Dothideomycetes	o__Capnodiales	f__Teratosphaeriaceae	g__Constantinomyces	s__Constantinomyces_minimus	OTU240
d__Eukaryota	k__Fungi	p__Ascomycota	c__Eurotiomycetes	o__Chaetothyriales	f__unclassified_o__Chaetothyriales	g__unclassified_o__Chaetothyriales	s__Chaetothyriales_sp	OTU991
d__Eukaryota	k__Fungi	p__Basidiomycota	c__Tremellomycetes	o__Tremellales	f__Bulleribasidiaceae	g__Dioszegia	s__Dioszegia_fristingensis	OTU266
d__Eukaryota	k__Fungi	p__Ascomycota	c__Pezizomycotina_cls_Incertae_sedis	o__Pezizomycotina_ord_Incertae_sedis	f__Pezizomycotina_fam_Incertae_sedis	g__Ciliophora	s__Ciliophora_sp	OTU614
d__Eukaryota	k__Fungi	p__Ascomycota	c__Sordariomycetes	o__Xylariales	f__Xylariaceae	g__Xylaria	s__Xylaria_sibirica	OTU606
d__Eukaryota	k__Fungi	p__unclassified_k__Fungi	c__unclassified_k__Fungi	o__unclassified_k__Fungi	f__unclassified_k__Fungi	g__unclassified_k__Fungi	s__unclassified_k__Fungi	OTU453
d__Eukaryota	k__Fungi	p__Ascomycota	c__Leotiomycetes	o__Helotiales	f__Helotiaceae	g__Tricladium	s__Tricladium_angulatum	OTU459
d__Eukaryota	k__Fungi	p__Basidiomycota	c__Agaricomycetes	o__Sebacinales	f__unclassified_o__Sebacinales	g__unclassified_o__Sebacinales	s__unclassified_o__Sebacinales	OTU316
d__Eukaryota	k__Fungi	p__Ascomycota	c__Dothideomycetes	o__Pleosporales	f__Sporormiaceae	g__Sporormiella	s__Sporormiella_leporina	OTU488
d__Eukaryota	k__Fungi	p__Ascomycota	c__Pezizomycotina_cls_Incertae_sedis	o__Pezizomycotina_ord_Incertae_sedis	f__Pezizomycotina_fam_Incertae_sedis	g__Ciliophora	s__Ciliophora_sp	OTU583
d__Eukaryota	k__Fungi	p__Ascomycota	c__Dothideomycetes	o__Pleosporales	f__Periconiaceae	g__Periconia	s__unclassified_g__Periconia	OTU299
d__Eukaryota	k__Fungi	p__Ascomycota	c__Leotiomycetes	o__Helotiales	f__unclassified_o__Helotiales	g__unclassified_o__Helotiales	s__unclassified_o__Helotiales	OTU865
d__Eukaryota	k__Fungi	p__Basidiomycota	c__Agaricomycetes	o__Russulales	f__Hericiaceae	g__Scytinostromella	s__Scytinostromella_heterogenea	OTU80
d__Eukaryota	k__Fungi	p__Basidiomycota	c__Microbotryomycetes	o__Sporidiobolales	f__Sporidiobolaceae	g__Rhodosporidiobolus	s__Rhodosporidiobolus_colostri	OTU1189
d__Eukaryota	k__Fungi	p__Basidiomycota	c__Agaricomycetes	o__Polyporales	f__Fomitopsidaceae	g__Piptoporus	s__Piptoporus_betulinus	OTU575
d__Eukaryota	k__Fungi	p__unclassified_k__Fungi	c__unclassified_k__Fungi	o__unclassified_k__Fungi	f__unclassified_k__Fungi	g__unclassified_k__Fungi	s__unclassified_k__Fungi	OTU1117
d__Eukaryota	k__Fungi	p__unclassified_k__Fungi	c__unclassified_k__Fungi	o__unclassified_k__Fungi	f__unclassified_k__Fungi	g__unclassified_k__Fungi	s__unclassified_k__Fungi	OTU511
d__Eukaryota	k__Fungi	p__Ascomycota	c__Sordariomycetes	o__Sordariales	f__Lasiosphaeriaceae	g__Schizothecium	s__unclassified_g__Schizothecium	OTU1153
d__Eukaryota	k__Fungi	p__Ascomycota	c__Leotiomycetes	o__Helotiales	f__Helotiales_fam_Incertae_sedis	g__Cadophora	s__Cadophora_fastigiata	OTU45
d__Eukaryota	k__Fungi	p__unclassified_k__Fungi	c__unclassified_k__Fungi	o__unclassified_k__Fungi	f__unclassified_k__Fungi	g__unclassified_k__Fungi	s__unclassified_k__Fungi	OTU1116

Domain	Kingdom	Phylum	Class	Order	Family	Genus	Species	OTU
d__Eukaryota	k__Fungi	p__Ascomycota	c__Dothideomycetes	o__Pleosporales	f__Pleosporales_fam_Incertae_sedis	g__Latorua	s__Latorua_caligans	OTU181
d__Eukaryota	k__Fungi	p__Basidiomycota	c__Agaricostilbomycetes	o__Agaricostilbales	f__unclassified_o__Agaricostilbales	g__unclassified_o__Agaricostilbales	s__unclassified_o__Agaricostilbales	OTU173
d__Eukaryota	k__Fungi	p__Ascomycota	c__Dothideomycetes	o__Pleosporales	f__unclassified_o__Pleosporales	g__unclassified_o__Pleosporales	s__Pleosporales_sp	OTU754
d__Eukaryota	k__Fungi	p__unclassified_k__Fungi	c__unclassified_k__Fungi	o__unclassified_k__Fungi	f__unclassified_k__Fungi	g__unclassified_k__Fungi	s__unclassified_k__Fungi	OTU134
d__Eukaryota	k__Fungi	p__Ascomycota	c__Lecanoromycetes	o__unclassified_c__Lecanoromycetes	f__unclassified_c__Lecanoromycetes	g__unclassified_c__Lecanoromycetes	s__unclassified_c__Lecanoromycetes	OTU820
d__Eukaryota	k__Fungi	p__Ascomycota	c__Dothideomycetes	o__Capnodiales	f__unclassified_o__Capnodiales	g__unclassified_o__Capnodiales	s__unclassified_o__Capnodiales	OTU146
d__Eukaryota	k__Fungi	p__unclassified_k__Fungi	c__unclassified_k__Fungi	o__unclassified_k__Fungi	f__unclassified_k__Fungi	g__unclassified_k__Fungi	s__unclassified_k__Fungi	OTU638
d__Eukaryota	k__Fungi	p__unclassified_k__Fungi	c__unclassified_k__Fungi	o__unclassified_k__Fungi	f__unclassified_k__Fungi	g__unclassified_k__Fungi	s__unclassified_k__Fungi	OTU639
d__Eukaryota	k__Fungi	p__Ascomycota	c__Leotiomycetes	o__Helotiales	f__unclassified_o__Helotiales	g__unclassified_o__Helotiales	s__unclassified_o__Helotiales	OTU105
d__Eukaryota	k__Fungi	p__Ascomycota	c__Dothideomycetes	o__Pleosporales	f__unclassified_o__Pleosporales	g__unclassified_o__Pleosporales	s__unclassified_o__Pleosporales	OTU890
d__Eukaryota	k__Fungi	p__Ascomycota	c__Leotiomycetes	o__Helotiales	f__Hyaloscyphaceae	g__Phialea	s__Phialea_sp	OTU832
d__Eukaryota	k__Fungi	p__Basidiomycota	c__Agaricomycetes	o__Agaricales	f__Inocybaceae	g__Inocybe	s__Inocybe_mixtilis	OTU58
d__Eukaryota	k__Fungi	p__Chytridiomycota	c__unclassified_p__Chytridiomycota	o__unclassified_p__Chytridiomycota	f__unclassified_p__Chytridiomycota	g__unclassified_p__Chytridiomycota	s__unclassified_p__Chytridiomycota	OTU498
d__Eukaryota	k__Fungi	p__Ascomycota	c__unclassified_p__Ascomycota	o__unclassified_p__Ascomycota	f__unclassified_p__Ascomycota	g__unclassified_p__Ascomycota	s__unclassified_p__Ascomycota	OTU1013
d__Eukaryota	k__Fungi	p__Ascomycota	c__Dothideomycetes	o__Dothideales	f__Aureobasidiaceae	g__Aureobasidium	s__Aureobasidium_subglaciale	OTU526
d__Eukaryota	k__Fungi	p__Ascomycota	c__Eurotiomycetes	o__Eurotiales	f__Aspergillaceae	g__Penicillium	s__unclassified_g__Penicillium	OTU345
d__Eukaryota	k__Fungi	p__Basidiomycota	c__Agaricomycetes	o__Boletales	f__Suillaceae	g__Suillus	s__Suillus_viscidus	OTU499
d__Eukaryota	k__Fungi	p__Glomeromycota	c__Archaeosporomycetes	o__Archaeosporales	f__unclassified_o__Archaeosporales	g__unclassified_o__Archaeosporales	s__Archaeosporales_sp	OTU1042
d__Eukaryota	k__Fungi	p__Ascomycota	c__Sordariomycetes	o__Hypocreales	f__Stachybotryaceae	g__unclassified_f__Stachybotryaceae	s__unclassified_f__Stachybotryaceae	OTU745
d__Eukaryota	k__Fungi	p__Ascomycota	c__Dothideomycetes	o__Pleosporales	f__Phaeosphaeriaceae	g__Phaeosphaeria	s__unclassified_g__Phaeosphaeria	OTU696
d__Eukaryota	k__Fungi	p__Ascomycota	c__unclassified_p__Ascomycota	o__unclassified_p__Ascomycota	f__unclassified_p__Ascomycota	g__unclassified_p__Ascomycota	s__unclassified_p__Ascomycota	OTU731
d__Eukaryota	k__Fungi	p__Chytridiomycota	c__Spizellomycetes	o__Spizellomycetales	f__Powellomycetaceae	g__Powellomyces	s__Powellomyces_sp	OTU435
d__Eukaryota	k__Fungi	p__Ascomycota	c__unclassified_p__Ascomycota	o__unclassified_p__Ascomycota	f__unclassified_p__Ascomycota	g__unclassified_p__Ascomycota	s__unclassified_p__Ascomycota	OTU735
d__Eukaryota	k__Fungi	p__Ascomycota	c__Leotiomycetes	o__unclassified_c__Leotiomycetes	f__unclassified_c__Leotiomycetes	g__unclassified_c__Leotiomycetes	s__unclassified_c__Leotiomycetes	OTU1058
d__Eukaryota	k__Fungi	p__unclassified_k__Fungi	c__unclassified_k__Fungi	o__unclassified_k__Fungi	f__unclassified_k__Fungi	g__unclassified_k__Fungi	s__unclassified_k__Fungi	OTU8
d__Eukaryota	k__Fungi	p__Ascomycota	c__Leotiomycetes	o__Helotiales	f__Helotiales_fam_Incertae_sedis	g__Cadophora	s__Cadophora_novi-eboraci	OTU259
d__Eukaryota	k__Fungi	p__Basidiomycota	c__Agaricomycetes	o__Hymenochaetales	f__Schizoporaceae	g__Hyphodontia	s__Hyphodontia_flavipora	OTU590
d__Eukaryota	k__Fungi	p__Ascomycota	c__Dothideomycetes	o__Capnodiales	f__Capnodiales_fam_Incertae_sedis	g__Arthrocatena	s__Arthrocatena_sp	OTU545
d__Eukaryota	k__Fungi	p__Ascomycota	c__unclassified_p__Ascomycota	o__unclassified_p__Ascomycota	f__unclassified_p__Ascomycota	g__unclassified_p__Ascomycota	s__unclassified_p__Ascomycota	OTU904
d__Eukaryota	k__Fungi	p__Chytridiomycota	c__unclassified_p__Chytridiomycota	o__unclassified_p__Chytridiomycota	f__unclassified_p__Chytridiomycota	g__unclassified_p__Chytridiomycota	s__unclassified_p__Chytridiomycota	OTU50
d__Eukaryota	k__Fungi	p__Basidiomycota	c__Agaricostilbomycetes	o__Agaricostilbales	f__Kondoaceae	g__unclassified_f__Kondoaceae	s__unclassified_f__Kondoaceae	OTU290

Domain	Kingdom	Phylum	Class	Order	Family	Genus	Species	OTU
d__Eukaryota	k__Fungi	p__Ascomycota	c__Lecanoromycetes	o__Acarosporales	f__Acarosporaceae	g__unclassified_f__Acarosporaceae	s__unclassified_f__Acarosporaceae	OTU135
d__Eukaryota	k__Fungi	p__Ascomycota	c__Leotiomycetes	o__unclassified_c__Leotiomycetes	f__unclassified_c__Leotiomycetes	g__unclassified_c__Leotiomycetes	s__unclassified_c__Leotiomycetes	OTU1055
d__Eukaryota	k__Fungi	p__Basidiomycota	c__Agaricomycetes	o__Geastrales	f__Geastraceae	g__Geastrum	s__unclassified_g__Geastrum	OTU356
d__Eukaryota	k__Fungi	p__Chytridiomycota	c__Rhizophydiomycetes	o__Rhizophydiales	f__Rhizophydiaceae	g__Rhizophydium	s__Rhizophydium_sp	OTU910
d__Eukaryota	k__Fungi	p__Ascomycota	c__Dothideomycetes	o__Pleosporales	f__Sporormiaceae	g__Preussia	s__unclassified_g__Preussia	OTU1222
d__Eukaryota	k__Fungi	p__unclassified_k__Fungi	c__unclassified_k__Fungi	o__unclassified_k__Fungi	f__unclassified_k__Fungi	g__unclassified_k__Fungi	s__unclassified_k__Fungi	OTU151
d__Eukaryota	k__Fungi	p__unclassified_k__Fungi	c__unclassified_k__Fungi	o__unclassified_k__Fungi	f__unclassified_k__Fungi	g__unclassified_k__Fungi	s__unclassified_k__Fungi	OTU150
d__Eukaryota	k__Fungi	p__Ascomycota	c__Dothideomycetes	o__Pleosporales	f__unclassified_o__Pleosporales	g__unclassified_o__Pleosporales	s__Pleosporales_sp	OTU347
d__Eukaryota	k__Fungi	p__Ascomycota	c__Eurotiomycetes	o__Chaetothyriales	f__Herpotrichiellaceae	g__Cladophialophora	s__Cladophialophora_sp	OTU687
d__Eukaryota	k__Fungi	p__Ascomycota	c__Leotiomycetes	o__Helotiales	f__Hyaloscyphaceae	g__unclassified_f__Hyaloscyphaceae	s__unclassified_f__Hyaloscyphaceae	OTU572
d__Eukaryota	k__Fungi	p__Basidiomycota	c__Tremellomycetes	o__Tremellales	f__Bulleribasidiaceae	g__Hannaella	s__Hannaella_coprosmae	OTU368
d__Eukaryota	k__Fungi	p__Ascomycota	c__Eurotiomycetes	o__Chaetothyriales	f__unclassified_o__Chaetothyriales	g__unclassified_o__Chaetothyriales	s__unclassified_o__Chaetothyriales	OTU411
d__Eukaryota	k__Fungi	p__Basidiomycota	c__Microbotryomycetes	o__Microbotryomycetes_ord_Incertae_sedis	f__Chrysozymaceae	g__Sampaiozyma	s__Sampaiozyma_sp	OTU285
d__Eukaryota	k__Fungi	p__Ascomycota	c__Leotiomycetes	o__Helotiales	f__Helotiaceae	g__unclassified_f__Helotiaceae	s__unclassified_f__Helotiaceae	OTU215
d__Eukaryota	k__Fungi	p__Ascomycota	c__Sordariomycetes	o__Sordariales	f__Chaetomiaceae	g__Chaetomium	s__unclassified_g__Chaetomium	OTU373
d__Eukaryota	k__Fungi	p__Ascomycota	c__Dothideomycetes	o__Pleosporales	f__Sporormiaceae	g__Sporormiella	s__Sporormiella_pulchella	OTU578
d__Eukaryota	k__Fungi	p__Ascomycota	c__unclassified_p__Ascomycota	o__unclassified_p__Ascomycota	f__unclassified_p__Ascomycota	g__unclassified_p__Ascomycota	s__unclassified_p__Ascomycota	OTU1065
d__Eukaryota	k__Fungi	p__Ascomycota	c__Dothideomycetes	o__Pleosporales	f__Pleosporaceae	g__Exserohilum	s__Exserohilum_rostratum	OTU183
d__Eukaryota	k__Fungi	p__Ascomycota	c__Eurotiomycetes	o__Chaetothyriales	f__Herpotrichiellaceae	g__Exophiala	s__Exophiala_xenobiotica	OTU1071
d__Eukaryota	k__Fungi	p__unclassified_k__Fungi	c__unclassified_k__Fungi	o__unclassified_k__Fungi	f__unclassified_k__Fungi	g__unclassified_k__Fungi	s__unclassified_k__Fungi	OTU666
d__Eukaryota	k__Fungi	p__Ascomycota	c__Sordariomycetes	o__Pleurotheciales	f__Pleurotheciaceae	g__Pleurotheciella	s__Pleurotheciella_sp	OTU1111
d__Eukaryota	k__Fungi	p__unclassified_k__Fungi	c__unclassified_k__Fungi	o__unclassified_k__Fungi	f__unclassified_k__Fungi	g__unclassified_k__Fungi	s__unclassified_k__Fungi	OTU664
d__Eukaryota	k__Fungi	p__Ascomycota	c__Dothideomycetes	o__Pleosporales	f__Phaeosphaeriaceae	g__Chaetosphaeronema	s__Chaetosphaeronema_sp	OTU779
d__Eukaryota	k__Fungi	p__Ascomycota	c__Pezizomycotina_cls_Incertae_sedis	o__Pezizomycotina_ord_Incertae_sedis	f__Pezizomycotina_fam_Incertae_sedis	g__Ciliophora	s__Ciliophora_sp	OTU974
d__Eukaryota	k__Fungi	p__Ascomycota	c__Leotiomycetes	o__Helotiales	f__Helotiaceae	g__Helotium	s__Helotium_sp	OTU257
d__Eukaryota	k__Fungi	p__Basidiomycota	c__Agaricomycetes	o__Trechisporales	f__Hydnodontaceae	g__Trechispora	s__unclassified_g__Trechispora	OTU527
d__Eukaryota	k__Fungi	p__Ascomycota	c__Dothideomycetes	o__Pleosporales	f__Lentitheciaceae	g__unclassified_f__Lentitheciaceae	s__unclassified_f__Lentitheciaceae	OTU1010
d__Eukaryota	k__Fungi	p__Ascomycota	c__Lichinomycetes	o__Lichinales	f__Lichinaceae	g__Phylliscum	s__Phylliscum_sp	OTU502
d__Eukaryota	k__Fungi	p__Basidiomycota	c__Tremellomycetes	o__Tremellales	f__Phaeotremellaceae	g__Phaeotremella	s__unclassified_g__Phaeotremella	OTU1122
d__Eukaryota	k__Fungi	p__unclassified_k__Fungi	c__unclassified_k__Fungi	o__unclassified_k__Fungi	f__unclassified_k__Fungi	g__unclassified_k__Fungi	s__unclassified_k__Fungi	OTU1223

Domain	Kingdom	Phylum	Class	Order	Family	Genus	Species	OTU
d__Eukaryota	k__Fungi	p__Ascomycota	c__Leotiomycetes	o__Helotiales	f__Hyaloscyphaceae	g__Gyoerffyella	s__Gyoerffyella_gemellipara	OTU909
d__Eukaryota	k__Fungi	p__unclassified_k__Fungi	c__unclassified_k__Fungi	o__unclassified_k__Fungi	f__unclassified_k__Fungi	g__unclassified_k__Fungi	s__unclassified_k__Fungi	OTU1221
d__Eukaryota	k__Fungi	p__unclassified_k__Fungi	c__unclassified_k__Fungi	o__unclassified_k__Fungi	f__unclassified_k__Fungi	g__unclassified_k__Fungi	s__unclassified_k__Fungi	OTU1229
d__Eukaryota	k__Fungi	p__Basidiomycota	c__Agaricomycetes	o__Agaricales	f__Psathyrellaceae	g__Coprinopsis	s__unclassified_g__Coprinopsis	OTU717
d__Eukaryota	k__Fungi	p__Ascomycota	c__Eurotiomycetes	o__Chaetothyriales	f__unclassified_o__Chaetothyriales	g__unclassified_o__Chaetothyriales	s__unclassified_o__Chaetothyriales	OTU562
d__Eukaryota	k__Fungi	p__Ascomycota	c__Eurotiomycetes	o__Chaetothyriales	f__unclassified_o__Chaetothyriales	g__unclassified_o__Chaetothyriales	s__unclassified_o__Chaetothyriales	OTU408
d__Eukaryota	k__Fungi	p__Basidiomycota	c__Agaricomycetes	o__unclassified_c__Agaricomycetes	f__unclassified_c__Agaricomycetes	g__unclassified_c__Agaricomycetes	s__unclassified_c__Agaricomycetes	OTU521
d__Eukaryota	k__Fungi	p__unclassified_k__Fungi	c__unclassified_k__Fungi	o__unclassified_k__Fungi	f__unclassified_k__Fungi	g__unclassified_k__Fungi	s__unclassified_k__Fungi	OTU698
d__Eukaryota	k__Fungi	p__Basidiomycota	c__Agaricomycetes	o__Gloeophyllales	f__Gloeophyllaceae	g__Gloeophyllum	s__Gloeophyllum_sepiarium	OTU12
d__Eukaryota	k__Fungi	p__Ascomycota	c__Leotiomycetes	o__Helotiales	f__Hyaloscyphaceae	g__Phialea	s__Phialea_sp	OTU994
d__Eukaryota	k__Fungi	p__Ascomycota	c__Leotiomycetes	o__Helotiales	f__Hyaloscyphaceae	g__Lachnum	s__Lachnum_sp	OTU1030
d__Eukaryota	k__Fungi	p__Ascomycota	c__Leotiomycetes	o__Helotiales	f__Vibrisseaceae	g__Phialocephala	s__Phialocephala_dimorphospora	OTU869
d__Eukaryota	k__Fungi	p__Ascomycota	c__Leotiomycetes	o__Helotiales	f__Helotiaceae	g__Crocicreas	s__Crocicreas_sp	OTU296
d__Eukaryota	k__Fungi	p__Ascomycota	c__Leotiomycetes	o__Helotiales	f__Helotiales_fam_Incertae_sedis	g__unclassified_f__Helotiales_fam_Incertae_sedis	s__unclassified_f__Helotiales_fam_Incertae_sedis	OTU956
d__Eukaryota	k__Fungi	p__Ascomycota	c__Eurotiomycetes	o__Chaetothyriales	f__unclassified_o__Chaetothyriales	g__unclassified_o__Chaetothyriales	s__Chaetothyriales_sp	OTU771
d__Eukaryota	k__Fungi	p__Ascomycota	c__Sordariomycetes	o__Hypocreales	f__Hypocreaceae	g__Trichoderma	s__Trichoderma_delicatulum	OTU933
d__Eukaryota	k__Fungi	p__Basidiomycota	c__Microbotryomycetes	o__Leucosporidiales	f__Leucosporidiaceae	g__Leucosporidium	s__Leucosporidium_golubevii	OTU289
d__Eukaryota	k__Fungi	p__Ascomycota	c__Sordariomycetes	o__Coniochaetales	f__unclassified_o__Coniochaetales	g__unclassified_o__Coniochaetales	s__Coniochaetales_sp	OTU1173
d__Eukaryota	k__Fungi	p__Ascomycota	c__Eurotiomycetes	o__Chaetothyriales	f__unclassified_o__Chaetothyriales	g__unclassified_o__Chaetothyriales	s__Chaetothyriales_sp	OTU702
d__Eukaryota	k__Fungi	p__Ascomycota	c__unclassified_p__Ascomycota	o__unclassified_p__Ascomycota	f__unclassified_p__Ascomycota	g__unclassified_p__Ascomycota	s__unclassified_p__Ascomycota	OTU781
d__Eukaryota	k__Fungi	p__Ascomycota	c__Eurotiomycetes	o__Chaetothyriales	f__unclassified_o__Chaetothyriales	g__unclassified_o__Chaetothyriales	s__unclassified_o__Chaetothyriales	OTU1227
d__Eukaryota	k__Fungi	p__unclassified_k__Fungi	c__unclassified_k__Fungi	o__unclassified_k__Fungi	f__unclassified_k__Fungi	g__unclassified_k__Fungi	s__unclassified_k__Fungi	OTU283
d__Eukaryota	k__Fungi	p__Ascomycota	c__unclassified_p__Ascomycota	o__unclassified_p__Ascomycota	f__unclassified_p__Ascomycota	g__unclassified_p__Ascomycota	s__unclassified_p__Ascomycota	OTU719
d__Eukaryota	k__Fungi	p__Basidiomycota	c__Agaricomycetes	o__Russulales	f__Stereaceae	g__Conferticium	s__Conferticium_ochraceum	OTU610
d__Eukaryota	k__Fungi	p__Ascomycota	c__Sordariomycetes	o__Sordariales	f__Chaetomiaceae	g__unclassified_f__Chaetomiaceae	s__unclassified_f__Chaetomiaceae	OTU547
d__Eukaryota	k__Fungi	p__Ascomycota	c__Eurotiomycetes	o__Chaetothyriales	f__Herpotrichiellaceae	g__Cladophialophora	s__unclassified_g__Cladophialophora	OTU1107
d__Eukaryota	k__Fungi	p__Ascomycota	c__Eurotiomycetes	o__Eurotiales	f__Aspergillaceae	g__Aspergillus	s__Aspergillus_tubingensis	OTU357
d__Eukaryota	k__Fungi	p__Ascomycota	c__Dothideomycetes	o__Pleosporales	f__Lentitheciaceae	g__unclassified_f__Lentitheciaceae	s__unclassified_f__Lentitheciaceae	OTU764
d__Eukaryota	k__Fungi	p__Basidiomycota	c__Agaricomycetes	o__Agaricales	f__unclassified_o__Agaricales	g__unclassified_o__Agaricales	s__unclassified_o__Agaricales	OTU586
d__Eukaryota	k__Fungi	p__Ascomycota	c__unclassified_p__Ascomycota	o__unclassified_p__Ascomycota	f__unclassified_p__Ascomycota	g__unclassified_p__Ascomycota	s__unclassified_p__Ascomycota	OTU868

Domain	Kingdom	Phylum	Class	Order	Family	Genus	Species	OTU
d__Eukaryota	k__Fungi	p__unclassified_k__Fungi	c__unclassified_k__Fungi	o__unclassified_k__Fungi	f__unclassified_k__Fungi	g__unclassified_k__Fungi	s__unclassified_k__Fungi	OTU1242
d__Eukaryota	k__Fungi	p__unclassified_k__Fungi	c__unclassified_k__Fungi	o__unclassified_k__Fungi	f__unclassified_k__Fungi	g__unclassified_k__Fungi	s__unclassified_k__Fungi	OTU1136
d__Eukaryota	k__Fungi	p__Ascomycota	c__Eurotiomycetes	o__Chaetothyriales	f__unclassified_o__Chaetothyriales	g__unclassified_o__Chaetothyriales	s__unclassified_o__Chaetothyriales	OTU968
d__Eukaryota	k__Fungi	p__unclassified_k__Fungi	c__unclassified_k__Fungi	o__unclassified_k__Fungi	f__unclassified_k__Fungi	g__unclassified_k__Fungi	s__unclassified_k__Fungi	OTU1134
d__Eukaryota	k__Fungi	p__Ascomycota	c__unclassified_p__Ascomycota	o__unclassified_p__Ascomycota	f__unclassified_p__Ascomycota	g__unclassified_p__Ascomycota	s__unclassified_p__Ascomycota	OTU1168
d__Eukaryota	k__Fungi	p__Ascomycota	c__Leotiomycetes	o__Helotiales	f__unclassified_o__Helotiales	g__unclassified_o__Helotiales	s__unclassified_o__Helotiales	OTU918
d__Eukaryota	k__Fungi	p__Basidiomycota	c__unclassified_p__Basidiomycota	o__unclassified_p__Basidiomycota	f__unclassified_p__Basidiomycota	g__unclassified_p__Basidiomycota	s__unclassified_p__Basidiomycota	OTU1016
d__Eukaryota	k__Fungi	p__Ascomycota	c__Leotiomycetes	o__Helotiales	f__Dermateaceae	g__unclassified_f__Dermateaceae	s__unclassified_f__Dermateaceae	OTU1190
d__Eukaryota	k__Fungi	p__Ascomycota	c__Taphrinomycetes	o__Taphrinales	f__Taphrinaceae	g__unclassified_f__Taphrinaceae	s__unclassified_f__Taphrinaceae	OTU16
d__Eukaryota	k__Fungi	p__Ascomycota	c__Leotiomycetes	o__Helotiales	f__Helotiaceae	g__Crocicreas	s__Crocicreas_sp	OTU892
d__Eukaryota	k__Fungi	p__Ascomycota	c__Dothideomycetes	o__Dothideales	f__Dothioraceae	g__Pseudoseptoria	s__Pseudoseptoria_obscura	OTU534
d__Eukaryota	k__Fungi	p__Ascomycota	c__GS37	o__GS37	f__unclassified_o__GS37	g__unclassified_o__GS37	s__GS37_sp_g__unclassified	OTU1054
d__Eukaryota	k__Fungi	p__Chytridiomycota	c__unclassified_p__Chytridiomycota	o__unclassified_p__Chytridiomycota	f__unclassified_p__Chytridiomycota	g__unclassified_p__Chytridiomycota	s__unclassified_p__Chytridiomycota	OTU908
d__Eukaryota	k__Fungi	p__Ascomycota	c__Dothideomycetes	o__Pleosporales	f__Cucurbitariaceae	g__Neocucurbitaria	s__Neocucurbitaria_unguis-hominis	OTU207
d__Eukaryota	k__Fungi	p__Ascomycota	c__Leotiomycetes	o__Helotiales	f__Sclerotiniaceae	g__Botrytis	s__unclassified_g__Botrytis	OTU978
d__Eukaryota	k__Fungi	p__unclassified_k__Fungi	c__unclassified_k__Fungi	o__unclassified_k__Fungi	f__unclassified_k__Fungi	g__unclassified_k__Fungi	s__unclassified_k__Fungi	OTU567
d__Eukaryota	k__Fungi	p__unclassified_k__Fungi	c__unclassified_k__Fungi	o__unclassified_k__Fungi	f__unclassified_k__Fungi	g__unclassified_k__Fungi	s__unclassified_k__Fungi	OTU560
d__Eukaryota	k__Fungi	p__unclassified_k__Fungi	c__unclassified_k__Fungi	o__unclassified_k__Fungi	f__unclassified_k__Fungi	g__unclassified_k__Fungi	s__unclassified_k__Fungi	OTU248
d__Eukaryota	k__Fungi	p__Ascomycota	c__Arthoniomycetes	o__Lichenostigmatales	f__Phaeococcomycetaceae	g__Phaeococcomyces	s__Phaeococcomyces_mexicanus	OTU313
d__Eukaryota	k__Fungi	p__Ascomycota	c__Dothideomycetes	o__Botryosphaeriales	f__unclassified_o__Botryosphaeriales	g__unclassified_o__Botryosphaeriales	s__Botryosphaeriales_sp	OTU116
d__Eukaryota	k__Fungi	p__Ascomycota	c__Dothideomycetes	o__Pleosporales	f__Didymellaceae	g__unclassified_f__Didymellaceae	s__unclassified_f__Didymellaceae	OTU981
d__Eukaryota	k__Fungi	p__Chytridiomycota	c__Spizellomycetes	o__Spizellomycetales	f__Powellomycetaceae	g__unclassified_f__Powellomycetaceae	s__Powellomycetaceae_sp	OTU746
d__Eukaryota	k__Fungi	p__Ascomycota	c__Sordariomycetes	o__Sordariales	f__unclassified_o__Sordariales	g__unclassified_o__Sordariales	s__unclassified_o__Sordariales	OTU252
d__Eukaryota	k__Fungi	p__Basidiomycota	c__Agaricomycetes	o__Amylocorticiales	f__Amylocorticiaceae	g__Anomoloma	s__Anomoloma_flavissimum	OTU124
d__Eukaryota	k__Fungi	p__unclassified_k__Fungi	c__unclassified_k__Fungi	o__unclassified_k__Fungi	f__unclassified_k__Fungi	g__unclassified_k__Fungi	s__unclassified_k__Fungi	OTU1265
d__Eukaryota	k__Fungi	p__Ascomycota	c__Dothideomycetes	o__Capnodiales	f__Teratosphaeriaceae	g__unclassified_f__Teratosphaeriaceae	s__unclassified_f__Teratosphaeriaceae	OTU986
d__Eukaryota	k__Fungi	p__Basidiomycota	c__Agaricomycetes	o__Polyporales	f__Fomitopsidaceae	g__Postia	s__Postia_caesia	OTU553
d__Eukaryota	k__Fungi	p__Ascomycota	c__Pezizomycotina_cls_Incertae_sedis	o__Pezizomycotina_ord_Incertae_sedis	f__Pezizomycotina_fam_Incertae_sedis	g__Ciliophora	s__Ciliophora_sp	OTU393
d__Eukaryota	k__Fungi	p__Ascomycota	c__Leotiomycetes	o__Helotiales	f__Leotiaceae	g__Alatospora	s__Alatospora_sp	OTU1041
d__Eukaryota	k__Fungi	p__Basidiomycota	c__Tremellomycetes	o__Cystofilobasidiales	f__Mrakiaceae	g__Tausonia	s__Tausonia_pullulans	OTU340
d__Eukaryota	k__Fungi	p__Ascomycota	c__Dothideomycetes	o__Pleosporales	f__unclassified_o__Pleosporales	g__unclassified_o__Pleosporales	s__unclassified_o__Pleosporales	OTU1033

Domain	Kingdom	Phylum	Class	Order	Family	Genus	Species	OTU
d__Eukaryota	k__Fungi	p__unclassified_k__Fungi	c__unclassified_k__Fungi	o__unclassified_k__Fungi	f__unclassified_k__Fungi	g__unclassified_k__Fungi	s__unclassified_k__Fungi	OTU855
d__Eukaryota	k__Fungi	p__unclassified_k__Fungi	c__unclassified_k__Fungi	o__unclassified_k__Fungi	f__unclassified_k__Fungi	g__unclassified_k__Fungi	s__unclassified_k__Fungi	OTU1084
d__Eukaryota	k__Fungi	p__unclassified_k__Fungi	c__unclassified_k__Fungi	o__unclassified_k__Fungi	f__unclassified_k__Fungi	g__unclassified_k__Fungi	s__unclassified_k__Fungi	OTU1089
d__Eukaryota	k__Fungi	p__Ascomycota	c__Sordariomycetes	o__Microascales	f__unclassified_o__Microascales	g__unclassified_o__Microascales	s__Microascales_sp	OTU89
d__Eukaryota	k__Fungi	p__Basidiomycota	c__Dacrymycetes	o__Dacrymycetales	f__Dacrymycetaceae	g__Dacrymyces	s__Dacrymyces_sp	OTU1021
d__Eukaryota	k__Fungi	p__Ascomycota	c__Dothideomycetes	o__Pleosporales	f__Pleosporaceae	g__Curvularia	s__Curvularia_intermedia	OTU123
d__Eukaryota	k__Fungi	p__Ascomycota	c__unclassified_p__Ascomycota	o__unclassified_p__Ascomycota	f__unclassified_p__Ascomycota	g__unclassified_p__Ascomycota	s__unclassified_p__Ascomycota	OTU558
d__Eukaryota	k__Fungi	p__Ascomycota	c__Leotiomycetes	o__Helotiales	f__Helotiaceae	g__Crocicreas	s__unclassified_g__Crocicreas	OTU1179
d__Eukaryota	k__Fungi	p__Rozellomycota	c__unclassified_p__Rozellomycota	o__unclassified_p__Rozellomycota	f__unclassified_p__Rozellomycota	g__unclassified_p__Rozellomycota	s__Rozellomycota_sp	OTU695
d__Eukaryota	k__Fungi	p__Ascomycota	c__Pezizomycotina_cls_Incertae_sedis	o__Pezizomycotina_ord_Incertae_sedis	f__Pezizomycotina_fam_Incertae_sedis	g__Ciliophora	s__Ciliophora_sp	OTU1104
d__Eukaryota	k__Fungi	p__Basidiomycota	c__Agaricomycetes	o__Agaricales	f__Entolomataceae	g__Entoloma	s__Entoloma_conferendum	OTU491
d__Eukaryota	k__Fungi	p__Ascomycota	c__Arthoniomycetes	o__Lichenostigmatales	f__unclassified_o__Lichenostigmatales	g__unclassified_o__Lichenostigmatales	s__Lichenostigmatales_sp	OTU143
d__Eukaryota	k__Fungi	p__Ascomycota	c__Taphrinomycetes	o__Taphrinales	f__Protomycetaceae	g__Protomyces	s__Protomyces_inouyei	OTU35
d__Eukaryota	k__Fungi	p__Ascomycota	c__Eurotiomycetes	o__Verrucariales	f__Verrucariaceae	g__unclassified_f__Verrucariaceae	s__Verrucariaceae_sp	OTU1241
d__Eukaryota	k__Fungi	p__Ascomycota	c__Eurotiomycetes	o__Chaetothyriales	f__Herpotrichiellaceae	g__Coniosporium	s__Coniosporium_sp	OTU244
d__Eukaryota	k__Fungi	p__Basidiomycota	c__Agaricomycetes	o__Russulales	f__Stereaceae	g__Stereum	s__Stereum_ostrea	OTU668
d__Eukaryota	k__Fungi	p__Ascomycota	c__Lecanoromycetes	o__Lecanorales	f__Stereocaulaceae	g__Stereocaulon	s__unclassified_g__Stereocaulon	OTU623
d__Eukaryota	k__Fungi	p__Ascomycota	c__Dothideomycetes	o__Capnodiales	f__unclassified_o__Capnodiales	g__unclassified_o__Capnodiales	s__unclassified_o__Capnodiales	OTU29
d__Eukaryota	k__Fungi	p__Ascomycota	c__unclassified_p__Ascomycota	o__unclassified_p__Ascomycota	f__unclassified_p__Ascomycota	g__unclassified_p__Ascomycota	s__unclassified_p__Ascomycota	OTU482
d__Eukaryota	k__Fungi	p__Ascomycota	c__Dothideomycetes	o__Pleosporales	f__Periconiaceae	g__Periconia	s__unclassified_g__Periconia	OTU525
d__Eukaryota	k__Fungi	p__Basidiomycota	c__Agaricomycetes	o__Agaricales	f__Strophariaceae	g__Pholiota	s__Pholiota_lenta	OTU2
d__Eukaryota	k__Fungi	p__Chytridiomycota	c__unclassified_p__Chytridiomycota	o__unclassified_p__Chytridiomycota	f__unclassified_p__Chytridiomycota	g__unclassified_p__Chytridiomycota	s__unclassified_p__Chytridiomycota	OTU607
d__Eukaryota	k__Fungi	p__Basidiomycota	c__Tremellomycetes	o__Tremellales	f__Bulleribasidiaceae	g__Dioszegia	s__unclassified_g__Dioszegia	OTU1198
d__Eukaryota	k__Fungi	p__Ascomycota	c__unclassified_p__Ascomycota	o__unclassified_p__Ascomycota	f__unclassified_p__Ascomycota	g__unclassified_p__Ascomycota	s__unclassified_p__Ascomycota	OTU489
d__Eukaryota	k__Fungi	p__Ascomycota	c__Eurotiomycetes	o__Chaetothyriales	f__Trichomeriaceae	g__Knufia	s__Knufia_peltigerae	OTU1047
d__Eukaryota	k__Fungi	p__Ascomycota	c__Dothideomycetes	o__Pleosporales	f__Phaeosphaeriaceae	g__Juncaceicola	s__unclassified_g__Juncaceicola	OTU916
d__Eukaryota	k__Fungi	p__Ascomycota	c__Eurotiomycetes	o__Chaetothyriales	f__Chaetothyriaceae	g__unclassified_f__Chaetothyriaceae	s__unclassified_f__Chaetothyriaceae	OTU425
d__Eukaryota	k__Fungi	p__Ascomycota	c__Dothideomycetes	o__Pleosporales	f__unclassified_o__Pleosporales	g__unclassified_o__Pleosporales	s__unclassified_o__Pleosporales	OTU1137
d__Eukaryota	k__Fungi	p__Ascomycota	c__Leotiomycetes	o__Helotiales	f__unclassified_o__Helotiales	g__unclassified_o__Helotiales	s__unclassified_o__Helotiales	OTU826
d__Eukaryota	k__Fungi	p__Basidiomycota	c__unclassified_p__Basidiomycota	o__unclassified_p__Basidiomycota	f__unclassified_p__Basidiomycota	g__unclassified_p__Basidiomycota	s__unclassified_p__Basidiomycota	OTU1018
d__Eukaryota	k__Fungi	p__Ascomycota	c__Leotiomycetes	o__Helotiales	f__unclassified_o__Helotiales	g__unclassified_o__Helotiales	s__unclassified_o__Helotiales	OTU823

Domain	Kingdom	Phylum	Class	Order	Family	Genus	Species	OTU
d__Eukaryota	k__Fungi	p__Ascomycota	c__Sordariomycetes	o__Hypocreales	f__unclassified_o__Hypocreales	g__unclassified_o__Hypocreales	s__unclassified_o__Hypocreales	OTU608
d__Eukaryota	k__Fungi	p__unclassified_k__Fungi	c__unclassified_k__Fungi	o__unclassified_k__Fungi	f__unclassified_k__Fungi	g__unclassified_k__Fungi	s__unclassified_k__Fungi	OTU308
d__Eukaryota	k__Fungi	p__Ascomycota	c__Leotiomycetes	o__unclassified_c__Leotiomycetes	f__unclassified_c__Leotiomycetes	g__unclassified_c__Leotiomycetes	s__unclassified_c__Leotiomycetes	OTU1248
d__Eukaryota	k__Fungi	p__Ascomycota	c__Leotiomycetes	o__Helotiales	f__Helotiaceae	g__Tetracladium	s__Tetracladium_sp	OTU430
d__Eukaryota	k__Fungi	p__Ascomycota	c__Leotiomycetes	o__Helotiales	f__Helotiaceae	g__unclassified_f__Helotiaceae	s__unclassified_f__Helotiaceae	OTU1126
d__Eukaryota	k__Fungi	p__Ascomycota	c__Lecanoromycetes	o__Caliciales	f__Physciaceae	g__unclassified_f__Physciaceae	s__unclassified_f__Physciaceae	OTU115
d__Eukaryota	k__Fungi	p__Ascomycota	c__Eurotiomycetes	o__Chaetothyriales	f__unclassified_o__Chaetothyriales	g__unclassified_o__Chaetothyriales	s__unclassified_o__Chaetothyriales	OTU458
d__Eukaryota	k__Fungi	p__Ascomycota	c__Saccharomycetes	o__Saccharomycetales	f__Saccharomycetales_fam_Incertae_sedis	g__Candida	s__Candida_sake	OTU1038
d__Eukaryota	k__Fungi	p__unclassified_k__Fungi	c__unclassified_k__Fungi	o__unclassified_k__Fungi	f__unclassified_k__Fungi	g__unclassified_k__Fungi	s__unclassified_k__Fungi	OTU304
d__Eukaryota	k__Fungi	p__Ascomycota	c__Dothideomycetes	o__Capnodiales	f__unclassified_o__Capnodiales	g__unclassified_o__Capnodiales	s__unclassified_o__Capnodiales	OTU109
d__Eukaryota	k__Fungi	p__Ascomycota	c__Leotiomycetes	o__Helotiales	f__Vibrisseaceae	g__Phialocephala	s__Phialocephala_fortinii	OTU734
d__Eukaryota	k__Fungi	p__Ascomycota	c__Leotiomycetes	o__Helotiales	f__Dermateaceae	g__Calloria	s__Calloria_urticae	OTU423
d__Eukaryota	k__Fungi	p__Ascomycota	c__Dothideomycetes	o__Pleosporales	f__Montagnulaceae	g__unclassified_f__Montagnulaceae	s__Montagnulaceae_sp	OTU346
d__Eukaryota	k__Fungi	p__Ascomycota	c__Leotiomycetes	o__Helotiales	f__unclassified_o__Helotiales	g__unclassified_o__Helotiales	s__unclassified_o__Helotiales	OTU960
d__Eukaryota	k__Fungi	p__unclassified_k__Fungi	c__unclassified_k__Fungi	o__unclassified_k__Fungi	f__unclassified_k__Fungi	g__unclassified_k__Fungi	s__unclassified_k__Fungi	OTU37
d__Eukaryota	k__Fungi	p__unclassified_k__Fungi	c__unclassified_k__Fungi	o__unclassified_k__Fungi	f__unclassified_k__Fungi	g__unclassified_k__Fungi	s__unclassified_k__Fungi	OTU36
d__Eukaryota	k__Fungi	p__unclassified_k__Fungi	c__unclassified_k__Fungi	o__unclassified_k__Fungi	f__unclassified_k__Fungi	g__unclassified_k__Fungi	s__unclassified_k__Fungi	OTU34
d__Eukaryota	k__Fungi	p__Ascomycota	c__Sordariomycetes	o__Hypocreales	f__Cordycipitaceae	g__Cordyceps	s__Cordyceps_militaris	OTU597
d__Eukaryota	k__Fungi	p__Ascomycota	c__Dothideomycetes	o__Pleosporales	f__Pleosporaceae	g__Curvularia	s__Curvularia_spicifera	OTU214
d__Eukaryota	k__Fungi	p__Ascomycota	c__Sordariomycetes	o__Togniniales	f__Togniniaceae	g__Phaeoacremonium	s__Phaeoacremonium_scolyti	OTU667
d__Eukaryota	k__Fungi	p__Basidiomycota	c__Agaricomycetes	o__Agaricales	f__Lycoperdaceae	g__Lycoperdon	s__Lycoperdon_perlatum	OTU554
d__Eukaryota	k__Fungi	p__Basidiomycota	c__Agaricomycetes	o__unclassified_c__Agaricomycetes	f__unclassified_c__Agaricomycetes	g__unclassified_c__Agaricomycetes	s__unclassified_c__Agaricomycetes	OTU825
d__Eukaryota	k__Fungi	p__Ascomycota	c__Dothideomycetes	o__Myriangiales	f__Myriangiales_fam_Incertae_sedis	g__Endosporium	s__Endosporium_sp	OTU280
d__Eukaryota	k__Fungi	p__Ascomycota	c__Sordariomycetes	o__Hypocreales	f__Nectriaceae	g__unclassified_f__Nectriaceae	s__unclassified_f__Nectriaceae	OTU1080
d__Eukaryota	k__Fungi	p__Ascomycota	c__Dothideomycetes	o__Pleosporales	f__unclassified_o__Pleosporales	g__unclassified_o__Pleosporales	s__unclassified_o__Pleosporales	OTU136
d__Eukaryota	k__Fungi	p__Ascomycota	c__Sordariomycetes	o__Sordariales	f__Lasiosphaeriaceae	g__Podospora	s__unclassified_g__Podospora	OTU724
d__Eukaryota	k__Fungi	p__Ascomycota	c__Eurotiomycetes	o__Verrucariales	f__unclassified_o__Verrucariales	g__unclassified_o__Verrucariales	s__Verrucariales_sp	OTU881
d__Eukaryota	k__Fungi	p__unclassified_k__Fungi	c__unclassified_k__Fungi	o__unclassified_k__Fungi	f__unclassified_k__Fungi	g__unclassified_k__Fungi	s__unclassified_k__Fungi	OTU70
d__Eukaryota	k__Fungi	p__Basidiomycota	c__Tremellomycetes	o__Filobasidiales	f__Piskurozymaceae	g__Solicoccozyma	s__Solicoccozyma_aeria	OTU422
d__Eukaryota	k__Fungi	p__Basidiomycota	c__Agaricomycetes	o__Boletales	f__Suillaceae	g__Suillus	s__Suillus_americanus	OTU224

Domain	Kingdom	Phylum	Class	Order	Family	Genus	Species	OTU
d__Eukaryota	k__Fungi	p__Ascomycota	c__Leotiomycetes	o__Helotiales	f__unclassified_o__Helotiales	g__unclassified_o__Helotiales	s__unclassified_o__Helotiales	OTU570
d__Eukaryota	k__Fungi	p__Ascomycota	c__Dothideomycetes	o__Pleosporales	f__Pleosporaceae	g__Alternaria	s__Alternaria_solani	OTU282
d__Eukaryota	k__Fungi	p__unclassified_k__Fungi	c__unclassified_k__Fungi	o__unclassified_k__Fungi	f__unclassified_k__Fungi	g__unclassified_k__Fungi	s__unclassified_k__Fungi	OTU1237
d__Eukaryota	k__Fungi	p__Ascomycota	c__Leotiomycetes	o__Helotiales	f__unclassified_o__Helotiales	g__unclassified_o__Helotiales	s__unclassified_o__Helotiales	OTU1218
d__Eukaryota	k__Fungi	p__Basidiomycota	c__Tremellomycetes	o__Tremellales	f__Phaeotremellaceae	g__Phaeotremella	s__Phaeotremella_sp	OTU140
d__Eukaryota	k__Fungi	p__Ascomycota	c__Leotiomycetes	o__unclassified_c__Leotiomycetes	f__unclassified_c__Leotiomycetes	g__unclassified_c__Leotiomycetes	s__unclassified_c__Leotiomycetes	OTU433
d__Eukaryota	k__Fungi	p__Ascomycota	c__Leotiomycetes	o__Helotiales	f__Hyaloscyphaceae	g__unclassified_f__Hyaloscyphaceae	s__unclassified_f__Hyaloscyphaceae	OTU723
d__Eukaryota	k__Fungi	p__Ascomycota	c__Leotiomycetes	o__Helotiales	f__unclassified_o__Helotiales	g__unclassified_o__Helotiales	s__unclassified_o__Helotiales	OTU1216
d__Eukaryota	k__Fungi	p__Basidiomycota	c__Cystobasidiomycetes	o__Cystobasidiales	f__Cystobasidiaceae	g__Cystobasidium	s__Cystobasidium_lysinophilum	OTU443
d__Eukaryota	k__Fungi	p__Ascomycota	c__unclassified_p__Ascomycota	o__unclassified_p__Ascomycota	f__unclassified_p__Ascomycota	g__unclassified_p__Ascomycota	s__unclassified_p__Ascomycota	OTU24
d__Eukaryota	k__Fungi	p__Monoblepharomycota	c__Hyaloraphidiomycetes	o__Hyaloraphidiales	f__Hyaloraphidiales_fam_Incertae_sedis	g__Hyaloraphidium	s__Hyaloraphidium_curvatum	OTU242
d__Eukaryota	k__Fungi	p__Basidiomycota	c__Agaricomycetes	o__Agaricales	f__Lycoperdaceae	g__unclassified_f__Lycoperdaceae	s__unclassified_f__Lycoperdaceae	OTU174
d__Eukaryota	k__Fungi	p__Ascomycota	c__Pezizomycotina_cls_Incertae_sedis	o__Pezizomycotina_ord_Incertae_sedis	f__Pezizomycotina_fam_Incertae_sedis	g__Ciliophora	s__Ciliophora_sp	OTU1199
d__Eukaryota	k__Fungi	p__Basidiomycota	c__Agaricomycetes	o__Polyporales	f__Ganodermataceae	g__Perenniporia	s__Perenniporia_subacida	OTU627
d__Eukaryota	k__Fungi	p__Ascomycota	c__Dothideomycetes	o__Pleosporales	f__Lentitheciaceae	g__Keissleriella	s__Keissleriella_culmifida	OTU1195
d__Eukaryota	k__Fungi	p__Ascomycota	c__Dothideomycetes	o__Capnodiales	f__unclassified_o__Capnodiales	g__unclassified_o__Capnodiales	s__unclassified_o__Capnodiales	OTU137
d__Eukaryota	k__Fungi	p__Ascomycota	c__Leotiomycetes	o__Helotiales	f__Helotiales_fam_Incertae_sedis	g__Varicosporium	s__Varicosporium_elodeae	OTU496
d__Eukaryota	k__Fungi	p__Ascomycota	c__unclassified_p__Ascomycota	o__unclassified_p__Ascomycota	f__unclassified_p__Ascomycota	g__unclassified_p__Ascomycota	s__unclassified_p__Ascomycota	OTU472
d__Eukaryota	k__Fungi	p__Ascomycota	c__Dothideomycetes	o__Pleosporales	f__Phaeosphaeriaceae	g__unclassified_f__Phaeosphaeriaceae	s__unclassified_f__Phaeosphaeriaceae	OTU984
d__Eukaryota	k__Fungi	p__Basidiomycota	c__Agaricomycetes	o__Trechisporales	f__Hydnodontaceae	g__Trechispora	s__unclassified_g__Trechispora	OTU539
d__Eukaryota	k__Fungi	p__unclassified_k__Fungi	c__unclassified_k__Fungi	o__unclassified_k__Fungi	f__unclassified_k__Fungi	g__unclassified_k__Fungi	s__unclassified_k__Fungi	OTU684
d__Eukaryota	k__Fungi	p__Ascomycota	c__Dothideomycetes	o__Capnodiales	f__Teratosphaeriaceae	g__unclassified_f__Teratosphaeriaceae	s__unclassified_f__Teratosphaeriaceae	OTU563
d__Eukaryota	k__Fungi	p__Chytridiomycota	c__unclassified_p__Chytridiomycota	o__unclassified_p__Chytridiomycota	f__unclassified_p__Chytridiomycota	g__unclassified_p__Chytridiomycota	s__unclassified_p__Chytridiomycota	OTU206
d__Eukaryota	k__Fungi	p__Ascomycota	c__Xylonomycetes	o__GS34	f__unclassified_o__GS34	g__unclassified_o__GS34	s__GS34_sp	OTU819
d__Eukaryota	k__Fungi	p__Ascomycota	c__Saccharomycetes	o__Saccharomycetales	f__Dipodascaceae	g__unclassified_f__Dipodascaceae	s__unclassified_f__Dipodascaceae	OTU10
d__Eukaryota	k__Fungi	p__unclassified_k__Fungi	c__unclassified_k__Fungi	o__unclassified_k__Fungi	f__unclassified_k__Fungi	g__unclassified_k__Fungi	s__unclassified_k__Fungi	OTU628
d__Eukaryota	k__Fungi	p__Ascomycota	c__Eurotiomycetes	o__Verrucariales	f__unclassified_o__Verrucariales	g__unclassified_o__Verrucariales	s__Verrucariales_sp	OTU794
d__Eukaryota	k__Fungi	p__Ascomycota	c__Leotiomycetes	o__Helotiales	f__Hyaloscyphaceae	g__Clathrosphaerina	s__Clathrosphaerina_zalewskii	OTU957
d__Eukaryota	k__Fungi	p__unclassified_k__Fungi	c__unclassified_k__Fungi	o__unclassified_k__Fungi	f__unclassified_k__Fungi	g__unclassified_k__Fungi	s__unclassified_k__Fungi	OTU1108
d__Eukaryota	k__Fungi	p__Ascomycota	c__Dothideomycetes	o__Pleosporales	f__Pleosporaceae	g__Alternaria	s__Alternaria_tenuissima	OTU835
d__Eukaryota	k__Fungi	p__Ascomycota	c__unclassified_p__Ascomycota	o__unclassified_p__Ascomycota	f__unclassified_p__Ascomycota	g__unclassified_p__Ascomycota	s__unclassified_p__Ascomycota	OTU930

Domain	Kingdom	Phylum	Class	Order	Family	Genus	Species	OTU
d__Eukaryota	k__Fungi	p__Ascomycota	c__Leotiomycetes	o__Helotiales	f__unclassified_o__Helotiales	g__unclassified_o__Helotiales	s__unclassified_o__Helotiales	OTU961
d__Eukaryota	k__Fungi	p__Ascomycota	c__Leotiomycetes	o__Phacidiales	f__unclassified_o__Phacidiales	g__unclassified_o__Phacidiales	s__unclassified_o__Phacidiales	OTU1177
d__Eukaryota	k__Fungi	p__Ascomycota	c__Eurotiomycetes	o__Chaetothyriales	f__unclassified_o__Chaetothyriales	g__unclassified_o__Chaetothyriales	s__unclassified_o__Chaetothyriales	OTU517
d__Eukaryota	k__Fungi	p__Ascomycota	c__Dothideomycetes	o__Capnodiales	f__Cladosporiaceae	g__Cladosporium	s__Cladosporium_pseudocladosporioides	OTU227
d__Eukaryota	k__Fungi	p__Ascomycota	c__unclassified_p__Ascomycota	o__unclassified_p__Ascomycota	f__unclassified_p__Ascomycota	g__unclassified_p__Ascomycota	s__unclassified_p__Ascomycota	OTU833
d__Eukaryota	k__Fungi	p__Basidiomycota	c__Tremellomycetes	o__Filobasidiales	f__Filobasidiaceae	g__Naganishia	s__Naganishia_antarctica	OTU71
d__Eukaryota	k__Fungi	p__Ascomycota	c__Leotiomycetes	o__Helotiales	f__Helotiaceae	g__Fontanospora	s__Fontanospora_sp	OTU317
d__Eukaryota	k__Fungi	p__Ascomycota	c__Sordariomycetes	o__Sordariales	f__Chaetomiaceae	g__Melanocarpus	s__unclassified_g__Melanocarpus	OTU376
d__Eukaryota	k__Fungi	p__Ascomycota	c__Leotiomycetes	o__Helotiales	f__Helotiaceae	g__Helotium	s__Helotium_sp	OTU676
d__Eukaryota	k__Fungi	p__Ascomycota	c__Leotiomycetes	o__Helotiales	f__Dermateaceae	g__Mollisia	s__Mollisia_sp	OTU632
d__Eukaryota	k__Fungi	p__Ascomycota	c__Lecanoromycetes	o__Lecanorales	f__Lecanoraceae	g__Lecanora	s__unclassified_g__Lecanora	OTU789
d__Eukaryota	k__Fungi	p__Ascomycota	c__Eurotiomycetes	o__Mycocaliciales	f__Mycocaliciaceae	g__Mycocalicium	s__Mycocalicium_victoriae	OTU569
d__Eukaryota	k__Fungi	p__unclassified_k__Fungi	c__unclassified_k__Fungi	o__unclassified_k__Fungi	f__unclassified_k__Fungi	g__unclassified_k__Fungi	s__unclassified_k__Fungi	OTU120
d__Eukaryota	k__Fungi	p__unclassified_k__Fungi	c__unclassified_k__Fungi	o__unclassified_k__Fungi	f__unclassified_k__Fungi	g__unclassified_k__Fungi	s__unclassified_k__Fungi	OTU121
d__Eukaryota	k__Fungi	p__Fungi_phy_Incertae_sedis	c__Fungi_cls_Incertae_sedis	o__Fungi_ord_Incertae_sedis	f__Fungi_fam_Incertae_sedis	g__Desmococcus	s__Desmococcus_sp	OTU221
d__Eukaryota	k__Fungi	p__unclassified_k__Fungi	c__unclassified_k__Fungi	o__unclassified_k__Fungi	f__unclassified_k__Fungi	g__unclassified_k__Fungi	s__unclassified_k__Fungi	OTU91
d__Eukaryota	k__Fungi	p__Fungi_phy_Incertae_sedis	c__Fungi_cls_Incertae_sedis	o__Fungi_ord_Incertae_sedis	f__Fungi_fam_Incertae_sedis	g__Desmococcus	s__Desmococcus_sp	OTU1225
d__Eukaryota	k__Fungi	p__unclassified_k__Fungi	c__unclassified_k__Fungi	o__unclassified_k__Fungi	f__unclassified_k__Fungi	g__unclassified_k__Fungi	s__unclassified_k__Fungi	OTU93
d__Eukaryota	k__Fungi	p__unclassified_k__Fungi	c__unclassified_k__Fungi	o__unclassified_k__Fungi	f__unclassified_k__Fungi	g__unclassified_k__Fungi	s__unclassified_k__Fungi	OTU1026
d__Eukaryota	k__Fungi	p__Basidiomycota	c__Agaricomycetes	o__Auriculariales	f__Exidiaceae	g__Basidiodendron	s__Basidiodendron_sp	OTU42
d__Eukaryota	k__Fungi	p__Ascomycota	c__unclassified_p__Ascomycota	o__unclassified_p__Ascomycota	f__unclassified_p__Ascomycota	g__unclassified_p__Ascomycota	s__unclassified_p__Ascomycota	OTU759
d__Eukaryota	k__Fungi	p__Ascomycota	c__Lecanoromycetes	o__Acarosporales	f__Acarosporaceae	g__unclassified_f__Acarosporaceae	s__unclassified_f__Acarosporaceae	OTU203
d__Eukaryota	k__Fungi	p__Ascomycota	c__Lecanoromycetes	o__unclassified_c__Lecanoromycetes	f__unclassified_c__Lecanoromycetes	g__unclassified_c__Lecanoromycetes	s__unclassified_c__Lecanoromycetes	OTU752
d__Eukaryota	k__Fungi	p__Ascomycota	c__Dothideomycetes	o__Capnodiales	f__Teratosphaeriaceae	g__Bryochiton	s__Bryochiton_monascus	OTU328
d__Eukaryota	k__Fungi	p__Ascomycota	c__Dothideomycetes	o__Pleosporales	f__Lentitheciaceae	g__Pleurophoma	s__Pleurophoma_sp	OTU1262
d__Eukaryota	k__Fungi	p__Ascomycota	c__Leotiomycetes	o__unclassified_c__Leotiomycetes	f__unclassified_c__Leotiomycetes	g__unclassified_c__Leotiomycetes	s__unclassified_c__Leotiomycetes	OTU22
d__Eukaryota	k__Fungi	p__Basidiomycota	c__Cystobasidiomycetes	o__Cystobasidiales	f__Cystobasidiaceae	g__Occultifur	s__Occultifur_sp	OTU15
d__Eukaryota	k__Fungi	p__unclassified_k__Fungi	c__unclassified_k__Fungi	o__unclassified_k__Fungi	f__unclassified_k__Fungi	g__unclassified_k__Fungi	s__unclassified_k__Fungi	OTU713
d__Eukaryota	k__Fungi	p__Ascomycota	c__Arthoniomycetes	o__Lichenostigmatales	f__Phaeococcomycetaceae	g__Phaeococcomyces	s__Phaeococcomyces_eucalypti	OTU5
d__Eukaryota	k__Fungi	p__Ascomycota	c__Lecanoromycetes	o__Lecanorales	f__unclassified_o__Lecanorales	g__unclassified_o__Lecanorales	s__Lecanorales_sp	OTU439
d__Eukaryota	k__Fungi	p__unclassified_k__Fungi	c__unclassified_k__Fungi	o__unclassified_k__Fungi	f__unclassified_k__Fungi	g__unclassified_k__Fungi	s__unclassified_k__Fungi	OTU76

Domain	Kingdom	Phylum	Class	Order	Family	Genus	Species	OTU
d__Eukaryota	k__Fungi	p__Ascomycota	c__Dothideomycetes	o__Pleosporales	f__Phaeosphaeriaceae	g__Neosetophoma	s__Neosetophoma_sp	OTU417
d__Eukaryota	k__Fungi	p__unclassified_k__Fungi	c__unclassified_k__Fungi	o__unclassified_k__Fungi	f__unclassified_k__Fungi	g__unclassified_k__Fungi	s__unclassified_k__Fungi	OTU715
d__Eukaryota	k__Fungi	p__unclassified_k__Fungi	c__unclassified_k__Fungi	o__unclassified_k__Fungi	f__unclassified_k__Fungi	g__unclassified_k__Fungi	s__unclassified_k__Fungi	OTU712
d__Eukaryota	k__Fungi	p__Ascomycota	c__Leotiomycetes	o__Helotiales	f__Dermateaceae	g__Cryptosporiopsis	s__Cryptosporiopsis_sp	OTU964
d__Eukaryota	k__Fungi	p__Ascomycota	c__unclassified_p__Ascomycota	o__unclassified_p__Ascomycota	f__unclassified_p__Ascomycota	g__unclassified_p__Ascomycota	s__unclassified_p__Ascomycota	OTU1246
d__Eukaryota	k__Fungi	p__unclassified_k__Fungi	c__unclassified_k__Fungi	o__unclassified_k__Fungi	f__unclassified_k__Fungi	g__unclassified_k__Fungi	s__unclassified_k__Fungi	OTU493
d__Eukaryota	k__Fungi	p__Ascomycota	c__Leotiomycetes	o__Helotiales	f__Dermateaceae	g__Cryptosporiopsis	s__Cryptosporiopsis_sp	OTU1029
d__Eukaryota	k__Fungi	p__Ascomycota	c__unclassified_p__Ascomycota	o__unclassified_p__Ascomycota	f__unclassified_p__Ascomycota	g__unclassified_p__Ascomycota	s__unclassified_p__Ascomycota	OTU948
d__Eukaryota	k__Fungi	p__Fungi_phy_Incertae_sedis	c__Fungi_cls_Incertae_sedis	o__Fungi_ord_Incertae_sedis	f__Fungi_fam_Incertae_sedis	g__Desmococcus	s__Desmococcus_sp	OTU97
d__Eukaryota	k__Fungi	p__unclassified_k__Fungi	c__unclassified_k__Fungi	o__unclassified_k__Fungi	f__unclassified_k__Fungi	g__unclassified_k__Fungi	s__unclassified_k__Fungi	OTU1032
d__Eukaryota	k__Fungi	p__Ascomycota	c__Leotiomycetes	o__Helotiales	f__Leotiaceae	g__Pezoloma	s__Pezoloma_ericae	OTU88
d__Eukaryota	k__Fungi	p__Ascomycota	c__Sordariomycetes	o__Coniochaetales	f__Coniochaetaceae	g__Coniochaeta	s__unclassified_g__Coniochaeta	OTU344
d__Eukaryota	k__Fungi	p__unclassified_k__Fungi	c__unclassified_k__Fungi	o__unclassified_k__Fungi	f__unclassified_k__Fungi	g__unclassified_k__Fungi	s__unclassified_k__Fungi	OTU1031
d__Eukaryota	k__Fungi	p__Chytridiomycota	c__unclassified_p__Chytridiomycota	o__unclassified_p__Chytridiomycota	f__unclassified_p__Chytridiomycota	g__unclassified_p__Chytridiomycota	s__unclassified_p__Chytridiomycota	OTU591
d__Eukaryota	k__Fungi	p__Monoblepharomycota	c__unclassified_p__Monoblepharomycota	o__unclassified_p__Monoblepharomycota	f__unclassified_p__Monoblepharomycota	g__unclassified_p__Monoblepharomycota	s__Monoblepharomycota_sp	OTU391
d__Eukaryota	k__Fungi	p__unclassified_k__Fungi	c__unclassified_k__Fungi	o__unclassified_k__Fungi	f__unclassified_k__Fungi	g__unclassified_k__Fungi	s__unclassified_k__Fungi	OTU1034
d__Eukaryota	k__Fungi	p__unclassified_k__Fungi	c__unclassified_k__Fungi	o__unclassified_k__Fungi	f__unclassified_k__Fungi	g__unclassified_k__Fungi	s__unclassified_k__Fungi	OTU1035
d__Eukaryota	k__Fungi	p__Ascomycota	c__Leotiomycetes	o__Helotiales	f__Helotiaceae	g__Collophora	s__Collophora_africana	OTU877
d__Eukaryota	k__Fungi	p__Basidiomycota	c__Tremellomycetes	o__Filobasidiales	f__Filobasidiaceae	g__Naganishia	s__unclassified_g__Naganishia	OTU1106
d__Eukaryota	k__Fungi	p__Ascomycota	c__unclassified_p__Ascomycota	o__unclassified_p__Ascomycota	f__unclassified_p__Ascomycota	g__unclassified_p__Ascomycota	s__unclassified_p__Ascomycota	OTU512
d__Eukaryota	k__Fungi	p__Ascomycota	c__Eurotiomycetes	o__unclassified_c__Eurotiomycetes	f__unclassified_c__Eurotiomycetes	g__unclassified_c__Eurotiomycetes	s__unclassified_c__Eurotiomycetes	OTU987
d__Eukaryota	k__Fungi	p__Ascomycota	c__Dothideomycetes	o__Pleosporales	f__unclassified_o__Pleosporales	g__unclassified_o__Pleosporales	s__unclassified_o__Pleosporales	OTU87
d__Eukaryota	k__Fungi	p__Ascomycota	c__Sordariomycetes	o__Hypocreales	f__Hypocreales_fam_Incertae_sedis	g__Sarocladium	s__Sarocladium_strictum	OTU427
d__Eukaryota	k__Fungi	p__unclassified_k__Fungi	c__unclassified_k__Fungi	o__unclassified_k__Fungi	f__unclassified_k__Fungi	g__unclassified_k__Fungi	s__unclassified_k__Fungi	OTU442
d__Eukaryota	k__Fungi	p__Ascomycota	c__unclassified_p__Ascomycota	o__unclassified_p__Ascomycota	f__unclassified_p__Ascomycota	g__unclassified_p__Ascomycota	s__unclassified_p__Ascomycota	OTU518
d__Eukaryota	k__Fungi	p__Ascomycota	c__Leotiomycetes	o__Helotiales	f__Helotiaceae	g__Tetracladium	s__Tetracladium_sp	OTU1082
d__Eukaryota	k__Fungi	p__Ascomycota	c__Sordariomycetes	o__unclassified_c__Sordariomycetes	f__unclassified_c__Sordariomycetes	g__unclassified_c__Sordariomycetes	s__unclassified_c__Sordariomycetes	OTU510
d__Eukaryota	k__Fungi	p__unclassified_k__Fungi	c__unclassified_k__Fungi	o__unclassified_k__Fungi	f__unclassified_k__Fungi	g__unclassified_k__Fungi	s__unclassified_k__Fungi	OTU445
d__Eukaryota	k__Fungi	p__unclassified_k__Fungi	c__unclassified_k__Fungi	o__unclassified_k__Fungi	f__unclassified_k__Fungi	g__unclassified_k__Fungi	s__unclassified_k__Fungi	OTU444
d__Eukaryota	k__Fungi	p__Ascomycota	c__unclassified_p__Ascomycota	o__unclassified_p__Ascomycota	f__unclassified_p__Ascomycota	g__unclassified_p__Ascomycota	s__unclassified_p__Ascomycota	OTU699

Domain	Kingdom	Phylum	Class	Order	Family	Genus	Species	OTU
d__Eukaryota	k__Fungi	p__Ascomycota	c__Leotiomycetes	o__Helotiales	f__unclassified_o__Helotiales	g__unclassified_o__Helotiales	s__unclassified_o__Helotiales	OTU708
d__Eukaryota	k__Fungi	p__Basidiomycota	c__Agaricostilbomycetes	o__unclassified_c__Agaricostilbomycetes	f__unclassified_c__Agaricostilbomycetes	g__unclassified_c__Agaricostilbomycetes	s__unclassified_c__Agaricostilbomycetes	OTU117
d__Eukaryota	k__Fungi	p__unclassified_k__Fungi	c__unclassified_k__Fungi	o__unclassified_k__Fungi	f__unclassified_k__Fungi	g__unclassified_k__Fungi	s__unclassified_k__Fungi	OTU523
d__Eukaryota	k__Fungi	p__unclassified_k__Fungi	c__unclassified_k__Fungi	o__unclassified_k__Fungi	f__unclassified_k__Fungi	g__unclassified_k__Fungi	s__unclassified_k__Fungi	OTU26
d__Eukaryota	k__Fungi	p__Ascomycota	c__Dothideomycetes	o__Pleosporales	f__Pleosporaceae	g__Comoclathris	s__Comoclathris_sp	OTU300
d__Eukaryota	k__Fungi	p__unclassified_k__Fungi	c__unclassified_k__Fungi	o__unclassified_k__Fungi	f__unclassified_k__Fungi	g__unclassified_k__Fungi	s__unclassified_k__Fungi	OTU338
d__Eukaryota	k__Fungi	p__Ascomycota	c__Leotiomycetes	o__Helotiales	f__Helotiaceae	g__Tetracladium	s__Tetracladium_sp	OTU447
d__Eukaryota	k__Fungi	p__unclassified_k__Fungi	c__unclassified_k__Fungi	o__unclassified_k__Fungi	f__unclassified_k__Fungi	g__unclassified_k__Fungi	s__unclassified_k__Fungi	OTU284
d__Eukaryota	k__Fungi	p__Ascomycota	c__Leotiomycetes	o__Helotiales	f__Leotiaceae	g__Neobulgaria	s__Neobulgaria_sp	OTU1100
d__Eukaryota	k__Fungi	p__unclassified_k__Fungi	c__unclassified_k__Fungi	o__unclassified_k__Fungi	f__unclassified_k__Fungi	g__unclassified_k__Fungi	s__unclassified_k__Fungi	OTU680
d__Eukaryota	k__Fungi	p__Ascomycota	c__Leotiomycetes	o__Helotiales	f__Dermateaceae	g__Naevala	s__Naevala_sp	OTU935
d__Eukaryota	k__Fungi	p__Ascomycota	c__Leotiomycetes	o__Helotiales	f__Helotiaceae	g__Tetracladium	s__Tetracladium_sp	OTU804
d__Eukaryota	k__Fungi	p__Basidiomycota	c__Agaricomycetes	o__Agaricales	f__Psathyrellaceae	g__Coprinellus	s__Coprinellus_disseminatus	OTU379
d__Eukaryota	k__Fungi	p__Basidiomycota	c__Agaricomycetes	o__Agaricales	f__Agaricaceae	g__Agaricus	s__unclassified_g__Agaricus	OTU792
d__Eukaryota	k__Fungi	p__Ascomycota	c__Pezizomycotina_cls_Incertae_sedis	o__Pezizomycotina_ord_Incertae_sedis	f__Pezizomycotina_fam_Incertae_sedis	g__Ciliophora	s__Ciliophora_sp	OTU1022
d__Eukaryota	k__Fungi	p__Ascomycota	c__Lecanoromycetes	o__Lecanorales	f__Lecanoraceae	g__Lecanora	s__unclassified_g__Lecanora	OTU691
d__Eukaryota	k__Fungi	p__Ascomycota	c__Leotiomycetes	o__unclassified_c__Leotiomycetes	f__unclassified_c__Leotiomycetes	g__unclassified_c__Leotiomycetes	s__unclassified_c__Leotiomycetes	OTU741
d__Eukaryota	k__Fungi	p__Ascomycota	c__Dothideomycetes	o__Pleosporales	f__Phaeosphaeriaceae	g__Juncaceicola	s__Juncaceicola_sp	OTU1023
d__Eukaryota	k__Fungi	p__Ascomycota	c__unclassified_p__Ascomycota	o__unclassified_p__Ascomycota	f__unclassified_p__Ascomycota	g__unclassified_p__Ascomycota	s__unclassified_p__Ascomycota	OTU1256
d__Eukaryota	k__Fungi	p__Ascomycota	c__Leotiomycetes	o__Helotiales	f__unclassified_o__Helotiales	g__unclassified_o__Helotiales	s__unclassified_o__Helotiales	OTU895
d__Eukaryota	k__Fungi	p__Ascomycota	c__Sordariomycetes	o__Sordariales	f__Sordariaceae	g__Sordaria	s__Sordaria_sp	OTU1192
d__Eukaryota	k__Fungi	p__Ascomycota	c__Leotiomycetes	o__Helotiales	f__unclassified_o__Helotiales	g__unclassified_o__Helotiales	s__unclassified_o__Helotiales	OTU893
d__Eukaryota	k__Fungi	p__Ascomycota	c__Dothideomycetes	o__Pleosporales	f__Pleosporales_fam_Incertae_sedis	g__Nigrograna	s__Nigrograna_cangshanensis	OTU232
d__Eukaryota	k__Fungi	p__Ascomycota	c__Leotiomycetes	o__Helotiales	f__Hyaloscyphaceae	g__Hyalopeziza	s__unclassified_g__Hyalopeziza	OTU889
d__Eukaryota	k__Fungi	p__unclassified_k__Fungi	c__unclassified_k__Fungi	o__unclassified_k__Fungi	f__unclassified_k__Fungi	g__unclassified_k__Fungi	s__unclassified_k__Fungi	OTU397
d__Eukaryota	k__Fungi	p__Ascomycota	c__Taphrinomycetes	o__Taphrinales	f__Protomycetaceae	g__Protomyces	s__unclassified_g__Protomyces	OTU176
d__Eukaryota	k__Fungi	p__Ascomycota	c__Eurotiomycetes	o__Eurotiales	f__Aspergillaceae	g__Aspergillus	s__Aspergillus_minisclerotigenes	OTU52
d__Eukaryota	k__Fungi	p__Basidiomycota	c__Agaricomycetes	o__Agaricales	f__Psathyrellaceae	g__Psathyrella	s__Psathyrella_candolleana	OTU912
d__Eukaryota	k__Fungi	p__Basidiomycota	c__Microbotryomycetes	o__Leucosporidiales	f__Leucosporidiaceae	g__Leucosporidium	s__Leucosporidium_sp	OTU212
d__Eukaryota	k__Fungi	p__Ascomycota	c__GS37	o__unclassified_c__GS37	f__unclassified_c__GS37	g__unclassified_c__GS37	s__GS37_sp_g__unclassified_c__GS37	OTU1180

Domain	Kingdom	Phylum	Class	Order	Family	Genus	Species	OTU
d__Eukaryota	k__Fungi	p__Ascomycota	c__Dothideomycetes	o__Pleosporales	f__Pleosporaceae	g__Alternaria	s__unclassified_g__Alternaria	OTU389
d__Eukaryota	k__Fungi	p__Ascomycota	c__Eurotiomycetes	o__Chaetothyriales	f__unclassified_o__Chaetothyriales	g__unclassified_o__Chaetothyriales	s__unclassified_o__Chaetothyriales	OTU847
d__Eukaryota	k__Fungi	p__unclassified_k__Fungi	c__unclassified_k__Fungi	o__unclassified_k__Fungi	f__unclassified_k__Fungi	g__unclassified_k__Fungi	s__unclassified_k__Fungi	OTU1011
d__Eukaryota	k__Fungi	p__Ascomycota	c__unclassified_p__Ascomycota	o__unclassified_p__Ascomycota	f__unclassified_p__Ascomycota	g__unclassified_p__Ascomycota	s__unclassified_p__Ascomycota	OTU358
d__Eukaryota	k__Fungi	p__Ascomycota	c__Leotiomycetes	o__Helotiales	f__Hyaloscyphaceae	g__Calycina	s__Calycina_sp	OTU1167
d__Eukaryota	k__Fungi	p__Ascomycota	c__Saccharomycetes	o__Saccharomycetales	f__Debaryomycetaceae	g__Meyerozyma	s__unclassified_g__Meyerozyma	OTU72
d__Eukaryota	k__Fungi	p__Ascomycota	c__Dothideomycetes	o__Pleosporales	f__Didymellaceae	g__Neoascochyta	s__Neoascochyta_paspali	OTU1252
d__Eukaryota	k__Fungi	p__Ascomycota	c__Pezizomycotina_cls_Incertae_sedis	o__Pezizomycotina_ord_Incertae_sedis	f__Pezizomycotina_fam_Incertae_sedis	g__Ciliophora	s__Ciliophora_sp	OTU454
d__Eukaryota	k__Fungi	p__Ascomycota	c__Leotiomycetes	o__Thelebolales	f__Pseudeurotiaceae	g__Gymnostellatospora	s__Gymnostellatospora_sp	OTU891
d__Eukaryota	k__Fungi	p__Ascomycota	c__Dothideomycetes	o__Capnodiales	f__Mycosphaerellaceae	g__Ramularia	s__Ramularia_aplospora	OTU48
d__Eukaryota	k__Fungi	p__unclassified_k__Fungi	c__unclassified_k__Fungi	o__unclassified_k__Fungi	f__unclassified_k__Fungi	g__unclassified_k__Fungi	s__unclassified_k__Fungi	OTU958
d__Eukaryota	k__Fungi	p__unclassified_k__Fungi	c__unclassified_k__Fungi	o__unclassified_k__Fungi	f__unclassified_k__Fungi	g__unclassified_k__Fungi	s__unclassified_k__Fungi	OTU780
d__Eukaryota	k__Fungi	p__Ascomycota	c__Eurotiomycetes	o__Chaetothyriales	f__Herpotrichiellaceae	g__Rhinocladiella	s__Rhinocladiella_sp	OTU402
d__Eukaryota	k__Fungi	p__Basidiomycota	c__Agaricomycetes	o__Agaricales	f__Strophariaceae	g__Pholiota	s__Pholiota_astragalina	OTU843
d__Eukaryota	k__Fungi	p__Ascomycota	c__Leotiomycetes	o__Helotiales	f__unclassified_o__Helotiales	g__unclassified_o__Helotiales	s__unclassified_o__Helotiales	OTU624
d__Eukaryota	k__Fungi	p__Ascomycota	c__Dothideomycetes	o__Capnodiales	f__Teratosphaeriaceae	g__Recurvomyces	s__unclassified_g__Recurvomyces	OTU234
d__Eukaryota	k__Fungi	p__Ascomycota	c__Dothideomycetes	o__Capnodiales	f__Cladosporiaceae	g__Cladosporium	s__Cladosporium_sphaerospermum	OTU1081
d__Eukaryota	k__Fungi	p__Ascomycota	c__Leotiomycetes	o__Helotiales	f__Helotiales_fam_Incertae_sedis	g__Cadophora	s__Cadophora_finlandica	OTU1157
d__Eukaryota	k__Fungi	p__Ascomycota	c__Dothideomycetes	o__Pleosporales	f__Phaeosphaeriaceae	g__Pseudoophiobolus	s__unclassified_g__Pseudoophiobolus	OTU1102
d__Eukaryota	k__Fungi	p__Ascomycota	c__Dothideomycetes	o__Capnodiales	f__Mycosphaerellaceae	g__Zymoseptoria	s__Zymoseptoria_sp	OTU1208
d__Eukaryota	k__Fungi	p__Ascomycota	c__Leotiomycetes	o__Helotiales	f__Helotiales_fam_Incertae_sedis	g__Cadophora	s__unclassified_g__Cadophora	OTU462
d__Eukaryota	k__Fungi	p__Ascomycota	c__Leotiomycetes	o__unclassified_c__Leotiomycetes	f__unclassified_c__Leotiomycetes	g__unclassified_c__Leotiomycetes	s__unclassified_c__Leotiomycetes	OTU791
d__Eukaryota	k__Fungi	p__Ascomycota	c__Eurotiomycetes	o__Eurotiales	f__Aspergillaceae	g__Penicillium	s__unclassified_g__Penicillium	OTU864
d__Eukaryota	k__Fungi	p__Basidiomycota	c__unclassified_p__Basidiomycota	o__unclassified_p__Basidiomycota	f__unclassified_p__Basidiomycota	g__unclassified_p__Basidiomycota	s__unclassified_p__Basidiomycota	OTU1212
d__Eukaryota	k__Fungi	p__unclassified_k__Fungi	c__unclassified_k__Fungi	o__unclassified_k__Fungi	f__unclassified_k__Fungi	g__unclassified_k__Fungi	s__unclassified_k__Fungi	OTU507
d__Eukaryota	k__Fungi	p__Ascomycota	c__Dothideomycetes	o__Venturiales	f__Sympoventuriaceae	g__Ochroconis	s__Ochroconis_bacilliformis	OTU361
d__Eukaryota	k__Fungi	p__Ascomycota	c__Sordariomycetes	o__Coniochaetales	f__Coniochaetaceae	g__Coniochaeta	s__unclassified_g__Coniochaeta	OTU273
d__Eukaryota	k__Fungi	p__Ascomycota	c__Leotiomycetes	o__Thelebolales	f__Pseudeurotiaceae	g__Leuconeurospora	s__unclassified_g__Leuconeurospora	OTU1000
d__Eukaryota	k__Fungi	p__Ascomycota	c__Dothideomycetes	o__Myriangiales	f__Myriangiales_fam_Incertae_sedis	g__Endosporium	s__Endosporium_sp	OTU367
d__Eukaryota	k__Fungi	p__Chytridiomycota	c__Polychytriomycetes	o__Polychytriales	f__unclassified_o__Polychytriales	g__unclassified_o__Polychytriales	s__Polychytriales_sp	OTU311
d__Eukaryota	k__Fungi	p__Chytridiomycota	c__unclassified_p__Chytridiomycota	o__unclassified_p__Chytridiomycota	f__unclassified_p__Chytridiomycota	g__unclassified_p__Chytridiomycota	s__unclassified_p__Chytridiomycota	OTU420

Domain	Kingdom	Phylum	Class	Order	Family	Genus	Species	OTU
d__Eukaryota	k__Fungi	p__Ascomycota	c__Lecanoromycetes	o__Peltigerales	f__Pannariaceae	g__unclassified_f__Pannariaceae	s__unclassified_f__Pannariaceae	OTU900
d__Eukaryota	k__Fungi	p__Ascomycota	c__Leotiomycetes	o__Helotiales	f__Helotiaceae	g__Hymenoscyphus	s__Hymenoscyphus_caudatus	OTU903
d__Eukaryota	k__Fungi	p__Basidiomycota	c__Microbotryomycetes	o__Microbotryales	f__unclassified_o__Microbotryales	g__unclassified_o__Microbotryales	s__unclassified_o__Microbotryales	OTU149
d__Eukaryota	k__Fungi	p__Mortierellomycota	c__unclassified_p__Mortierellomycota	o__unclassified_p__Mortierellomycota	f__unclassified_p__Mortierellomycota	g__unclassified_p__Mortierellomycota	s__unclassified_p__Mortierellomycota	OTU1006
d__Eukaryota	k__Fungi	p__Mortierellomycota	c__unclassified_p__Mortierellomycota	o__unclassified_p__Mortierellomycota	f__unclassified_p__Mortierellomycota	g__unclassified_p__Mortierellomycota	s__Mortierellomycotina	OTU1008
d__Eukaryota	k__Fungi	p__Ascomycota	c__Leotiomycetes	o__Helotiales	f__unclassified_o__Helotiales	g__unclassified_o__Helotiales	s__unclassified_o__Helotiales	OTU1253
d__Eukaryota	k__Fungi	p__Ascomycota	c__unclassified_p__Ascomycota	o__unclassified_p__Ascomycota	f__unclassified_p__Ascomycota	g__unclassified_p__Ascomycota	s__unclassified_p__Ascomycota	OTU805
d__Eukaryota	k__Fungi	p__unclassified_k__Fungi	c__unclassified_k__Fungi	o__unclassified_k__Fungi	f__unclassified_k__Fungi	g__unclassified_k__Fungi	s__unclassified_k__Fungi	OTU730
d__Eukaryota	k__Fungi	p__Ascomycota	c__Dothideomycetes	o__Pleosporales	f__Sporormiaceae	g__Sporormiella	s__Sporormiella_minimoides	OTU669
d__Eukaryota	k__Fungi	p__Ascomycota	c__Sordariomycetes	o__Sordariales	f__Cephalothecaceae	g__Phialemonium	s__Phialemonium_sp	OTU386
d__Eukaryota	k__Fungi	p__Ascomycota	c__Saccharomycetes	o__Saccharomycetales	f__Saccharomycetaceae	g__Issatchenkia	s__Issatchenkia_orientalis	OTU652
d__Eukaryota	k__Fungi	p__Ascomycota	c__Sordariomycetes	o__Hypocreales	f__Hypocreales_fam_Incertae_sedis	g__Emericellopsis	s__unclassified_g__Emericellopsis	OTU131
d__Eukaryota	k__Fungi	p__Ascomycota	c__unclassified_p__Ascomycota	o__unclassified_p__Ascomycota	f__unclassified_p__Ascomycota	g__unclassified_p__Ascomycota	s__unclassified_p__Ascomycota	OTU650
d__Eukaryota	k__Fungi	p__Basidiomycota	c__Tremellomycetes	o__Tremellales	f__Bulleribasidiaceae	g__Vishniacozyma	s__unclassified_g__Vishniacozyma	OTU897
d__Eukaryota	k__Fungi	p__Ascomycota	c__Eurotiomycetes	o__Verrucariales	f__Verrucariaceae	g__Verrucaria	s__Verrucaria_sp	OTU829
d__Eukaryota	k__Fungi	p__Mortierellomycota	c__Mortierellomycetes	o__Mortierellales	f__Mortierellaceae	g__Mortierella	s__Mortierella_simplex	OTU665
d__Eukaryota	k__Fungi	p__Ascomycota	c__Leotiomycetes	o__Helotiales	f__unclassified_o__Helotiales	g__unclassified_o__Helotiales	s__unclassified_o__Helotiales	OTU836
d__Eukaryota	k__Fungi	p__Ascomycota	c__Dothideomycetes	o__Pleosporales	f__Sporormiaceae	g__Sporormiella	s__Sporormiella_similis	OTU1162
d__Eukaryota	k__Fungi	p__Basidiomycota	c__Agaricomycetes	o__Hymenochaetales	f__Schizoporaceae	g__Xylodon	s__Xylodon_brevisetus	OTU592
d__Eukaryota	k__Fungi	p__Ascomycota	c__Dothideomycetes	o__Pleosporales	f__Phaeosphaeriaceae	g__Paraphoma	s__Paraphoma_sp	OTU1224
d__Eukaryota	k__Fungi	p__Ascomycota	c__Leotiomycetes	o__Helotiales	f__unclassified_o__Helotiales	g__unclassified_o__Helotiales	s__unclassified_o__Helotiales	OTU885
d__Eukaryota	k__Fungi	p__Ascomycota	c__Saccharomycetes	o__Saccharomycetales	f__Dipodascaceae	g__Dipodascus	s__Dipodascus_geotrichum	OTU204
d__Eukaryota	k__Fungi	p__unclassified_k__Fungi	c__unclassified_k__Fungi	o__unclassified_k__Fungi	f__unclassified_k__Fungi	g__unclassified_k__Fungi	s__unclassified_k__Fungi	OTU49
d__Eukaryota	k__Fungi	p__Ascomycota	c__Eurotiomycetes	o__Chaetothyriales	f__Herpotrichiellaceae	g__Heteroconium	s__Heteroconium_sp	OTU1201
d__Eukaryota	k__Fungi	p__Chytridiomycota	c__Chytridiomycetes	o__Chytridiales	f__unclassified_o__Chytridiales	g__unclassified_o__Chytridiales	s__Chytridiales_sp	OTU1020
d__Eukaryota	k__Fungi	p__Ascomycota	c__Dothideomycetes	o__Pleosporales	f__unclassified_o__Pleosporales	g__unclassified_o__Pleosporales	s__unclassified_o__Pleosporales	OTU824
d__Eukaryota	k__Fungi	p__Ascomycota	c__Dothideomycetes	o__Capnodiales	f__Teratosphaeriaceae	g__unclassified_f__Teratosphaeriaceae	s__unclassified_f__Teratosphaeriaceae	OTU837
d__Eukaryota	k__Fungi	p__Ascomycota	c__Leotiomycetes	o__Helotiales	f__Helotiaceae	g__unclassified_f__Helotiaceae	s__unclassified_f__Helotiaceae	OTU911
d__Eukaryota	k__Fungi	p__Basidiomycota	c__Agaricomycetes	o__Agaricales	f__Psathyrellaceae	g__Coprinellus	s__Coprinellus_radians	OTU514
d__Eukaryota	k__Fungi	p__Ascomycota	c__unclassified_p__Ascomycota	o__unclassified_p__Ascomycota	f__unclassified_p__Ascomycota	g__unclassified_p__Ascomycota	s__unclassified_p__Ascomycota	OTU876
d__Eukaryota	k__Fungi	p__Ascomycota	c__Dothideomycetes	o__Capnodiales	f__Mycosphaerellaceae	g__Mycodiella	s__unclassified_g__Mycodiella	OTU132

Domain	Kingdom	Phylum	Class	Order	Family	Genus	Species	OTU
d__Eukaryota	k__Fungi	p__Chytridiomycota	c__Spizellomycetes	o__Spizellomycetales	f__unclassified_o__Spizellomycetales	g__unclassified_o__Spizellomycetales	s__Spizellomycetales_sp	OTU1068
d__Eukaryota	k__Fungi	p__Ascomycota	c__Eurotiomycetes	o__Chaetothyriales	f__Trichomeriaceae	g__Bradymyces	s__Bradymyces_sp	OTU681
d__Eukaryota	k__Fungi	p__Basidiomycota	c__Agaricomycetes	o__unclassified_c__Agaricomycetes	f__unclassified_c__Agaricomycetes	g__unclassified_c__Agaricomycetes	s__unclassified_c__Agaricomycetes	OTU1244
d__Eukaryota	k__Fungi	p__Ascomycota	c__Leotiomycetes	o__Helotiales	f__unclassified_o__Helotiales	g__unclassified_o__Helotiales	s__unclassified_o__Helotiales	OTU1086
d__Eukaryota	k__Fungi	p__Ascomycota	c__Dothideomycetes	o__unclassified_c__Dothideomycetes	f__unclassified_c__Dothideomycetes	g__unclassified_c__Dothideomycetes	s__unclassified_c__Dothideomycetes	OTU175
d__Eukaryota	k__Fungi	p__Chytridiomycota	c__Spizellomycetes	o__Spizellomycetales	f__unclassified_o__Spizellomycetales	g__unclassified_o__Spizellomycetales	s__unclassified_o__Spizellomycetales	OTU880
d__Eukaryota	k__Fungi	p__Ascomycota	c__Eurotiomycetes	o__Chaetothyriales	f__Herpotrichiellaceae	g__Cladophialophora	s__Cladophialophora_proteae	OTU81
d__Eukaryota	k__Fungi	p__Ascomycota	c__Lecanoromycetes	o__Trapeliales	f__Trapeliaceae	g__unclassified_f__Trapeliaceae	s__unclassified_f__Trapeliaceae	OTU271
d__Eukaryota	k__Fungi	p__Ascomycota	c__Eurotiomycetes	o__Chaetothyriales	f__unclassified_o__Chaetothyriales	g__unclassified_o__Chaetothyriales	s__unclassified_o__Chaetothyriales	OTU993
d__Eukaryota	k__Fungi	p__Ascomycota	c__Dothideomycetes	o__Pleosporales	f__Pleosporaceae	g__Comoclathris	s__Comoclathris_sp	OTU41
d__Eukaryota	k__Fungi	p__Ascomycota	c__Leotiomycetes	o__Helotiales	f__unclassified_o__Helotiales	g__unclassified_o__Helotiales	s__unclassified_o__Helotiales	OTU1088
d__Eukaryota	k__Fungi	p__Ascomycota	c__Dothideomycetes	o__Pleosporales	f__Phaeosphaeriaceae	g__unclassified_f__Phaeosphaeriaceae	s__unclassified_f__Phaeosphaeriaceae	OTU324
d__Eukaryota	k__Fungi	p__unclassified_k__Fungi	c__unclassified_k__Fungi	o__unclassified_k__Fungi	f__unclassified_k__Fungi	g__unclassified_k__Fungi	s__unclassified_k__Fungi	OTU1059
d__Eukaryota	k__Fungi	p__Ascomycota	c__Leotiomycetes	o__Helotiales	f__Leotiaceae	g__Alatospora	s__Alatospora_acuminata	OTU1125
d__Eukaryota	k__Fungi	p__Ascomycota	c__Sordariomycetes	o__Hypocreales	f__Nectriaceae	g__Fusicolla	s__Fusicolla_merismoides	OTU354
d__Eukaryota	k__Fungi	p__Ascomycota	c__Pezizomycetes	o__Pezizales	f__Ascobolaceae	g__Ascobolus	s__Ascobolus_sp	OTU1028
d__Eukaryota	k__Fungi	p__Ascomycota	c__Dothideomycetes	o__Pleosporales	f__unclassified_o__Pleosporales	g__unclassified_o__Pleosporales	s__unclassified_o__Pleosporales	OTU685
d__Eukaryota	k__Fungi	p__Basidiomycota	c__Agaricomycetes	o__Atheliales	f__Atheliaceae	g__Amphinema	s__Amphinema_byssoides	OTU1163
d__Eukaryota	k__Fungi	p__Ascomycota	c__Sordariomycetes	o__Hypocreales	f__Hypocreales_fam_Incertae_sedis	g__Sarocladium	s__unclassified_g__Sarocladium	OTU509
d__Eukaryota	k__Fungi	p__Ascomycota	c__Dothideomycetes	o__Pleosporales	f__Cucurbitariaceae	g__Pyrenochaeta	s__unclassified_g__Pyrenochaeta	OTU1155
d__Eukaryota	k__Fungi	p__Ascomycota	c__unclassified_p__Ascomycota	o__unclassified_p__Ascomycota	f__unclassified_p__Ascomycota	g__unclassified_p__Ascomycota	s__unclassified_p__Ascomycota	OTU387
d__Eukaryota	k__Fungi	p__Ascomycota	c__unclassified_p__Ascomycota	o__unclassified_p__Ascomycota	f__unclassified_p__Ascomycota	g__unclassified_p__Ascomycota	s__unclassified_p__Ascomycota	OTU1197
d__Eukaryota	k__Fungi	p__Ascomycota	c__Sordariomycetes	o__Glomerellales	f__Plectosphaerellaceae	g__Cephalosporium	s__Cephalosporium_sp	OTU277
d__Eukaryota	k__Fungi	p__Ascomycota	c__Dothideomycetes	o__Pleosporales	f__Phaeosphaeriaceae	g__Phaeosphaeriopsis	s__Phaeosphaeriopsis_sp	OTU966
d__Eukaryota	k__Fungi	p__unclassified_k__Fungi	c__unclassified_k__Fungi	o__unclassified_k__Fungi	f__unclassified_k__Fungi	g__unclassified_k__Fungi	s__unclassified_k__Fungi	OTU168
d__Eukaryota	k__Fungi	p__Ascomycota	c__unclassified_p__Ascomycota	o__unclassified_p__Ascomycota	f__unclassified_p__Ascomycota	g__unclassified_p__Ascomycota	s__unclassified_p__Ascomycota	OTU548
d__Eukaryota	k__Fungi	p__Ascomycota	c__Leotiomycetes	o__Helotiales	f__Hyaloscyphaceae	g__Arachnopeziza	s__Arachnopeziza_aurata	OTU557
d__Eukaryota	k__Fungi	p__Ascomycota	c__unclassified_p__Ascomycota	o__unclassified_p__Ascomycota	f__unclassified_p__Ascomycota	g__unclassified_p__Ascomycota	s__unclassified_p__Ascomycota	OTU982
d__Eukaryota	k__Fungi	p__Ascomycota	c__Lecanoromycetes	o__Candelariales	f__Candelariaceae	g__unclassified_f__Candelariaceae	s__unclassified_f__Candelariaceae	OTU461
d__Eukaryota	k__Fungi	p__Ascomycota	c__Sordariomycetes	o__Xylariales	f__Bartaliniaceae	g__Truncatella	s__Truncatella_angustata	OTU813
d__Eukaryota	k__Fungi	p__Ascomycota	c__Eurotiomycetes	o__Chaetothyriales	f__unclassified_o__Chaetothyriales	g__unclassified_o__Chaetothyriales	s__unclassified_o__Chaetothyriales	OTU375

Domain	Kingdom	Phylum	Class	Order	Family	Genus	Species	OTU
d__Eukaryota	k__Fungi	p__Basidiomycota	c__Agaricostilbomycetes	o__Agaricostilbales	f__Chionosphaeraceae	g__unclassified_f__Chionosphaeraceae	s__unclassified_f__Chionosphaeraceae	OTU838
d__Eukaryota	k__Fungi	p__Basidiomycota	c__Agaricomycetes	o__Agaricales	f__Tricholomataceae	g__Collybia	s__unclassified_g__Collybia	OTU809
d__Eukaryota	k__Fungi	p__Ascomycota	c__Dothideomycetes	o__Dothideales	f__Dothideaceae	g__Celosporium	s__Celosporium_sp	OTU747
d__Eukaryota	k__Fungi	p__Basidiomycota	c__Tremellomycetes	o__Tremellales	f__Bulleribasidiaceae	g__Dioszegia	s__Dioszegia_sp	OTU727
d__Eukaryota	k__Fungi	p__unclassified_k__Fungi	c__unclassified_k__Fungi	o__unclassified_k__Fungi	f__unclassified_k__Fungi	g__unclassified_k__Fungi	s__unclassified_k__Fungi	OTU615
d__Eukaryota	k__Fungi	p__Basidiomycota	c__Cystobasidiomycetes	o__Cystobasidiales	f__Cystobasidiaceae	g__Cystobasidium	s__Cystobasidium_laryngis	OTU341
d__Eukaryota	k__Fungi	p__unclassified_k__Fungi	c__unclassified_k__Fungi	o__unclassified_k__Fungi	f__unclassified_k__Fungi	g__unclassified_k__Fungi	s__unclassified_k__Fungi	OTU613
d__Eukaryota	k__Fungi	p__Ascomycota	c__Leotiomycetes	o__Helotiales	f__Helotiaceae	g__Meliniomyces	s__Meliniomyces_bicolor	OTU1202
d__Eukaryota	k__Fungi	p__unclassified_k__Fungi	c__unclassified_k__Fungi	o__unclassified_k__Fungi	f__unclassified_k__Fungi	g__unclassified_k__Fungi	s__unclassified_k__Fungi	OTU611
d__Eukaryota	k__Fungi	p__Ascomycota	c__Dothideomycetes	o__Dothideales	f__Aureobasidiaceae	g__Kabatiella	s__Kabatiella_sp	OTU226
d__Eukaryota	k__Fungi	p__Ascomycota	c__Dothideomycetes	o__Pleosporales	f__Phaeosphaeriaceae	g__Sclerostagonospora	s__Sclerostagonospora_cycadis	OTU424
d__Eukaryota	k__Fungi	p__Basidiomycota	c__Agaricomycetes	o__Agaricales	f__Lycoperdaceae	g__Lycoperdon	s__Lycoperdon_sp	OTU243
d__Eukaryota	k__Fungi	p__Ascomycota	c__Pezizomycotina_cls_Incertae_sedis	o__Pezizomycotina_ord_Incertae_sedis	f__Pezizomycotina_fam_Incertae_sedis	g__Ciliophora	s__Ciliophora_sp	OTU1096
d__Eukaryota	k__Fungi	p__Ascomycota	c__Pezizomycetes	o__Pezizales	f__Tuberaceae	g__Tuber	s__unclassified_g__Tuber	OTU1191
d__Eukaryota	k__Fungi	p__Ascomycota	c__Lecanoromycetes	o__Lecanorales	f__Stereocaulaceae	g__Stereocaulon	s__unclassified_g__Stereocaulon	OTU276
d__Eukaryota	k__Fungi	p__Ascomycota	c__Dothideomycetes	o__Pleosporales	f__Phaeosphaeriaceae	g__Phaeosphaeria	s__Phaeosphaeria_microscopica	OTU142
d__Eukaryota	k__Fungi	p__unclassified_k__Fungi	c__unclassified_k__Fungi	o__unclassified_k__Fungi	f__unclassified_k__Fungi	g__unclassified_k__Fungi	s__unclassified_k__Fungi	OTU1174
d__Eukaryota	k__Fungi	p__Ascomycota	c__Sordariomycetes	o__Hypocreales	f__unclassified_o__Hypocreales	g__unclassified_o__Hypocreales	s__unclassified_o__Hypocreales	OTU770
d__Eukaryota	k__Fungi	p__Ascomycota	c__Leotiomycetes	o__unclassified_c__Leotiomycetes	f__unclassified_c__Leotiomycetes	g__unclassified_c__Leotiomycetes	s__unclassified_c__Leotiomycetes	OTU947
d__Eukaryota	k__Fungi	p__Basidiomycota	c__Agaricomycetes	o__Agaricales	f__Psathyrellaceae	g__Psathyrella	s__Psathyrella_squamosa	OTU19
d__Eukaryota	k__Fungi	p__Basidiomycota	c__Microbotryomycetes	o__Leucosporidiales	f__Leucosporidiaceae	g__Leucosporidium	s__unclassified_g__Leucosporidium	OTU246
d__Eukaryota	k__Fungi	p__Ascomycota	c__Dothideomycetes	o__Mytilinidales	f__Gloniaceae	g__Cenococcum	s__Cenococcum_geophilum	OTU1161
d__Eukaryota	k__Fungi	p__Ascomycota	c__Lecanoromycetes	o__Lecanorales	f__Pilocarpaceae	g__Leimonis	s__Leimonis_erratica	OTU870
d__Eukaryota	k__Fungi	p__Basidiomycota	c__Agaricomycetes	o__Corticiales	f__Corticiaceae	g__Corticium	s__Corticium_sp	OTU943
d__Eukaryota	k__Fungi	p__Ascomycota	c__Dothideomycetes	o__Pleosporales	f__Pleosporaceae	g__Curvularia	s__Curvularia_inaequalis	OTU542
d__Eukaryota	k__Fungi	p__unclassified_k__Fungi	c__unclassified_k__Fungi	o__unclassified_k__Fungi	f__unclassified_k__Fungi	g__unclassified_k__Fungi	s__unclassified_k__Fungi	OTU976
d__Eukaryota	k__Fungi	p__Ascomycota	c__Leotiomycetes	o__unclassified_c__Leotiomycetes	f__unclassified_c__Leotiomycetes	g__unclassified_c__Leotiomycetes	s__Leotiomycetes_sp	OTU888
d__Eukaryota	k__Fungi	p__Basidiomycota	c__Agaricomycetes	o__Gomphales	f__Gomphaceae	g__Ramaria	s__Ramaria_gracilis	OTU555
d__Eukaryota	k__Fungi	p__Chytridiomycota	c__unclassified_p__Chytridiomycota	o__unclassified_p__Chytridiomycota	f__unclassified_p__Chytridiomycota	g__unclassified_p__Chytridiomycota	s__unclassified_p__Chytridiomycota	OTU519
d__Eukaryota	k__Fungi	p__Ascomycota	c__Leotiomycetes	o__Helotiales	f__Helotiales_fam_Incertae_sedis	g__Cadophora	s__Cadophora_luteo-olivacea	OTU1164
d__Eukaryota	k__Fungi	p__Basidiomycota	c__Agaricomycetes	o__Agaricales	f__Strophariaceae	g__Pholiota	s__Pholiota_squarrosoides	OTU709

Domain	Kingdom	Phylum	Class	Order	Family	Genus	Species	OTU
d__Eukaryota	k__Fungi	p__Ascomycota	c__unclassified_p__Ascomycota	o__unclassified_p__Ascomycota	f__unclassified_p__Ascomycota	g__unclassified_p__Ascomycota	s__unclassified_p__Ascomycota	OTU546
d__Eukaryota	k__Fungi	p__Ascomycota	c__Dothideomycetes	o__Pleosporales	f__unclassified_o__Pleosporales	g__unclassified_o__Pleosporales	s__unclassified_o__Pleosporales	OTU822
d__Eukaryota	k__Fungi	p__Ascomycota	c__unclassified_p__Ascomycota	o__unclassified_p__Ascomycota	f__unclassified_p__Ascomycota	g__unclassified_p__Ascomycota	s__unclassified_p__Ascomycota	OTU693
d__Eukaryota	k__Fungi	p__Ascomycota	c__Leotiomycetes	o__Helotiales	f__Hyaloscyphaceae	g__Cistella	s__Cistella_sp	OTU1073
d__Eukaryota	k__Fungi	p__Basidiomycota	c__unclassified_p__Basidiomycota	o__unclassified_p__Basidiomycota	f__unclassified_p__Basidiomycota	g__unclassified_p__Basidiomycota	s__unclassified_p__Basidiomycota	OTU998
d__Eukaryota	k__Fungi	p__Fungi_phy_Incertae_sedis	c__Fungi_cls_Incertae_sedis	o__Fungi_ord_Incertae_sedis	f__Fungi_fam_Incertae_sedis	g__Desmococcus	s__Desmococcus_sp	OTU1083
d__Eukaryota	k__Fungi	p__Ascomycota	c__Leotiomycetes	o__Helotiales	f__Helotiales_fam_Incertae_sedis	g__Spirosphaera	s__Spirosphaera_minuta	OTU972
d__Eukaryota	k__Fungi	p__Ascomycota	c__unclassified_p__Ascomycota	o__unclassified_p__Ascomycota	f__unclassified_p__Ascomycota	g__unclassified_p__Ascomycota	s__unclassified_p__Ascomycota	OTU799
d__Eukaryota	k__Fungi	p__unclassified_k__Fungi	c__unclassified_k__Fungi	o__unclassified_k__Fungi	f__unclassified_k__Fungi	g__unclassified_k__Fungi	s__unclassified_k__Fungi	OTU1132
d__Eukaryota	k__Fungi	p__unclassified_k__Fungi	c__unclassified_k__Fungi	o__unclassified_k__Fungi	f__unclassified_k__Fungi	g__unclassified_k__Fungi	s__unclassified_k__Fungi	OTU1214
d__Eukaryota	k__Fungi	p__Ascomycota	c__unclassified_p__Ascomycota	o__unclassified_p__Ascomycota	f__unclassified_p__Ascomycota	g__unclassified_p__Ascomycota	s__unclassified_p__Ascomycota	OTU796
d__Eukaryota	k__Fungi	p__Ascomycota	c__Dothideomycetes	o__Capnodiales	f__Cladosporiaceae	g__Rachicladosporium	s__unclassified_g__Rachicladosporium	OTU231
d__Eukaryota	k__Fungi	p__Ascomycota	c__Sordariomycetes	o__Hypocreales	f__Hypocreaceae	g__Trichoderma	s__Trichoderma_gamsii	OTU102
d__Eukaryota	k__Fungi	p__unclassified_k__Fungi	c__unclassified_k__Fungi	o__unclassified_k__Fungi	f__unclassified_k__Fungi	g__unclassified_k__Fungi	s__unclassified_k__Fungi	OTU409
d__Eukaryota	k__Fungi	p__Ascomycota	c__Eurotiomycetes	o__Eurotiales	f__Aspergillaceae	g__Aspergillus	s__Aspergillus_penicillioides	OTU686
d__Eukaryota	k__Fungi	p__Ascomycota	c__Eurotiomycetes	o__Chaetothyriales	f__Herpotrichiellaceae	g__Exophiala	s__unclassified_g__Exophiala	OTU456
d__Eukaryota	k__Fungi	p__Basidiomycota	c__Cystobasidiomycetes	o__Cystobasidiomycetes_ord_Incertae_sedis	f__Symmetrosporaceae	g__Symmetrospora	s__unclassified_g__Symmetrospora	OTU1151
d__Eukaryota	k__Fungi	p__Ascomycota	c__Sordariomycetes	o__Hypocreales	f__Hypocreales_fam_Incertae_sedis	g__Sarocladium	s__Sarocladium_sp	OTU1169
d__Eukaryota	k__Fungi	p__Ascomycota	c__Orbiliomycetes	o__Orbiliales	f__Orbiliaceae	g__unclassified_f__Orbiliaceae	s__unclassified_f__Orbiliaceae	OTU1135
d__Eukaryota	k__Fungi	p__Ascomycota	c__Dothideomycetes	o__Pleosporales	f__Didymosphaeriaceae	g__Spegazzinia	s__Spegazzinia_tessarthra	OTU772
d__Eukaryota	k__Fungi	p__Ascomycota	c__Dothideomycetes	o__Capnodiales	f__unclassified_o__Capnodiales	g__unclassified_o__Capnodiales	s__unclassified_o__Capnodiales	OTU263
d__Eukaryota	k__Fungi	p__Ascomycota	c__Dothideomycetes	o__Pleosporales	f__unclassified_o__Pleosporales	g__unclassified_o__Pleosporales	s__unclassified_o__Pleosporales	OTU914
d__Eukaryota	k__Fungi	p__Ascomycota	c__Leotiomycetes	o__Helotiales	f__Helotiales_fam_Incertae_sedis	g__Chalara	s__unclassified_g__Chalara	OTU468
d__Eukaryota	k__Fungi	p__unclassified_k__Fungi	c__unclassified_k__Fungi	o__unclassified_k__Fungi	f__unclassified_k__Fungi	g__unclassified_k__Fungi	s__unclassified_k__Fungi	OTU1007
d__Eukaryota	k__Fungi	p__Ascomycota	c__Dothideomycetes	o__Pleosporales	f__unclassified_o__Pleosporales	g__unclassified_o__Pleosporales	s__unclassified_o__Pleosporales	OTU913
d__Eukaryota	k__Fungi	p__Fungi_phy_Incertae_sedis	c__Fungi_cls_Incertae_sedis	o__Fungi_ord_Incertae_sedis	f__Fungi_fam_Incertae_sedis	g__Desmococcus	s__Desmococcus_sp	OTU1074
d__Eukaryota	k__Fungi	p__Ascomycota	c__Sordariomycetes	o__Sordariales	f__Lasiosphaeriaceae	g__Schizothecium	s__Schizothecium_glutinans	OTU1050
d__Eukaryota	k__Fungi	p__Ascomycota	c__Sordariomycetes	o__Hypocreales	f__Clavicipitaceae	g__Pochonia	s__Pochonia_cordycepisociata	OTU812
d__Eukaryota	k__Fungi	p__Ascomycota	c__Leotiomycetes	o__Helotiales	f__Dermateaceae	g__Mollisia	s__Mollisia_sp	OTU929
d__Eukaryota	k__Fungi	p__Ascomycota	c__Dothideomycetes	o__Pleosporales	f__Phaeosphaeriaceae	g__unclassified_f__Phaeosphaeriaceae	s__unclassified_f__Phaeosphaeriaceae	OTU333

Domain	Kingdom	Phylum	Class	Order	Family	Genus	Species	OTU
d__Eukaryota	k__Fungi	p__Ascomycota	c__Sordariomycetes	o__Boliniales	f__Boliniaceae	g__unclassified_f__Boliniaceae	s__unclassified_f__Boliniaceae	OTU718
d__Eukaryota	k__Fungi	p__Ascomycota	c__Sordariomycetes	o__Hypocreales	f__Nectriaceae	g__Ilyonectria	s__Ilyonectria_mors-panacis	OTU806
d__Eukaryota	k__Fungi	p__Ascomycota	c__unclassified_p__Ascomycota	o__unclassified_p__Ascomycota	f__unclassified_p__Ascomycota	g__unclassified_p__Ascomycota	s__unclassified_p__Ascomycota	OTU647
d__Eukaryota	k__Fungi	p__Ascomycota	c__Dothideomycetes	o__Capnodiales	f__Capnodiales_fam_Incertae_sedis	g__Pseudovirgaria	s__Pseudovirgaria_grisea	OTU64
d__Eukaryota	k__Fungi	p__Ascomycota	c__Leotiomycetes	o__Helotiales	f__unclassified_o__Helotiales	g__unclassified_o__Helotiales	s__unclassified_o__Helotiales	OTU907
d__Eukaryota	k__Fungi	p__Ascomycota	c__Eurotiomycetes	o__Verrucariales	f__unclassified_o__Verrucariales	g__unclassified_o__Verrucariales	s__Verrucariales_sp	OTU1228
d__Eukaryota	k__Fungi	p__Chytridiomycota	c__unclassified_p__Chytridiomycota	o__unclassified_p__Chytridiomycota	f__unclassified_p__Chytridiomycota	g__unclassified_p__Chytridiomycota	s__unclassified_p__Chytridiomycota	OTU186
d__Eukaryota	k__Fungi	p__Ascomycota	c__Sordariomycetes	o__Hypocreales	f__Cordycipitaceae	g__Beauveria	s__Beauveria_pseudobassiana	OTU1095
d__Eukaryota	k__Fungi	p__Ascomycota	c__Eurotiomycetes	o__Chaetothyriales	f__Herpotrichiellaceae	g__Cladophialophora	s__unclassified_g__Cladophialophora	OTU504
d__Eukaryota	k__Fungi	p__Basidiomycota	c__Agaricomycetes	o__Agaricales	f__Psathyrellaceae	g__Coprinellus	s__Coprinellus_angulatus	OTU574
d__Eukaryota	k__Fungi	p__Ascomycota	c__Lecanoromycetes	o__Lecanorales	f__Micareaceae	g__Micarea	s__Micarea_lithinella	OTU31
d__Eukaryota	k__Fungi	p__Basidiomycota	c__Tremellomycetes	o__Tremellales	f__Bulleribasidiaceae	g__Dioszegia	s__Dioszegia_fristingensis	OTU1263
d__Eukaryota	k__Fungi	p__Basidiomycota	c__Microbotryomycetes	o__Microbotryales	f__Ustilentylomataceae	g__Ustilentyloma	s__Ustilentyloma_sp	OTU414
d__Eukaryota	k__Fungi	p__Ascomycota	c__Dothideomycetes	o__Capnodiales	f__unclassified_o__Capnodiales	g__unclassified_o__Capnodiales	s__Capnodiales_sp	OTU744
d__Eukaryota	k__Fungi	p__Ascomycota	c__Taphrinomycetes	o__Taphrinales	f__Taphrinaceae	g__Taphrina	s__unclassified_g__Taphrina	OTU90
d__Eukaryota	k__Fungi	p__Ascomycota	c__Dothideomycetes	o__Pleosporales	f__unclassified_o__Pleosporales	g__unclassified_o__Pleosporales	s__unclassified_o__Pleosporales	OTU82
d__Eukaryota	k__Fungi	p__Ascomycota	c__Sordariomycetes	o__Phomatosporales	f__Phomatosporaceae	g__Phomatospora	s__unclassified_g__Phomatospora	OTU1187
d__Eukaryota	k__Fungi	p__Ascomycota	c__Pezizomycetes	o__Pezizales	f__Sarcosomataceae	g__Donadinia	s__Donadinia_sibirica	OTU619
d__Eukaryota	k__Fungi	p__Ascomycota	c__Leotiomycetes	o__Helotiales	f__unclassified_o__Helotiales	g__unclassified_o__Helotiales	s__unclassified_o__Helotiales	OTU863
d__Eukaryota	k__Fungi	p__unclassified_k__Fungi	c__unclassified_k__Fungi	o__unclassified_k__Fungi	f__unclassified_k__Fungi	g__unclassified_k__Fungi	s__unclassified_k__Fungi	OTU272
d__Eukaryota	k__Fungi	p__unclassified_k__Fungi	c__unclassified_k__Fungi	o__unclassified_k__Fungi	f__unclassified_k__Fungi	g__unclassified_k__Fungi	s__unclassified_k__Fungi	OTU1093
d__Eukaryota	k__Fungi	p__Ascomycota	c__Leotiomycetes	o__unclassified_c__Leotiomycetes	f__unclassified_c__Leotiomycetes	g__unclassified_c__Leotiomycetes	s__unclassified_c__Leotiomycetes	OTU1250
d__Eukaryota	k__Fungi	p__Ascomycota	c__Dothideomycetes	o__Dothideales	f__unclassified_o__Dothideales	g__unclassified_o__Dothideales	s__Dothideales_sp	OTU671
d__Eukaryota	k__Fungi	p__unclassified_k__Fungi	c__unclassified_k__Fungi	o__unclassified_k__Fungi	f__unclassified_k__Fungi	g__unclassified_k__Fungi	s__unclassified_k__Fungi	OTU278
d__Eukaryota	k__Fungi	p__unclassified_k__Fungi	c__unclassified_k__Fungi	o__unclassified_k__Fungi	f__unclassified_k__Fungi	g__unclassified_k__Fungi	s__unclassified_k__Fungi	OTU1099
d__Eukaryota	k__Fungi	p__Ascomycota	c__unclassified_p__Ascomycota	o__unclassified_p__Ascomycota	f__unclassified_p__Ascomycota	g__unclassified_p__Ascomycota	s__unclassified_p__Ascomycota	OTU774
d__Eukaryota	k__Fungi	p__Ascomycota	c__Eurotiomycetes	o__Chaetothyriales	f__Herpotrichiellaceae	g__Coniosporium	s__Coniosporium_sp	OTU380
d__Eukaryota	k__Fungi	p__Ascomycota	c__Leotiomycetes	o__Helotiales	f__unclassified_o__Helotiales	g__unclassified_o__Helotiales	s__unclassified_o__Helotiales	OTU258
d__Eukaryota	k__Fungi	p__Ascomycota	c__Sordariomycetes	o__Hypocreales	f__Nectriaceae	g__Rodentomyces	s__Rodentomyces_sp	OTU369
d__Eukaryota	k__Fungi	p__Ascomycota	c__Dothideomycetes	o__Capnodiales	f__Extremaceae	g__unclassified_f__Extremaceae	s__Extremaceae_sp	OTU213
d__Eukaryota	k__Fungi	p__Ascomycota	c__Leotiomycetes	o__unclassified_c__Leotiomycetes	f__unclassified_c__Leotiomycetes	g__unclassified_c__Leotiomycetes	s__unclassified_c__Leotiomycetes	OTU261

Domain	Kingdom	Phylum	Class	Order	Family	Genus	Species	OTU
d__Eukaryota	k__Fungi	p__Chytridiomycota	c__unclassified_p__Chytridiomycota	o__unclassified_p__Chytridiomycota	f__unclassified_p__Chytridiomycota	g__unclassified_p__Chytridiomycota	s__unclassified_p__Chytridiomycota	OTU508
d__Eukaryota	k__Fungi	p__Ascomycota	c__Dothideomycetes	o__Dothideales	f__unclassified_o__Dothideales	g__unclassified_o__Dothideales	s__unclassified_o__Dothideales	OTU342
d__Eukaryota	k__Fungi	p__Ascomycota	c__Lecanoromycetes	o__Acarosporales	f__Acarosporaceae	g__unclassified_f__Acarosporaceae	s__unclassified_f__Acarosporaceae	OTU431
d__Eukaryota	k__Fungi	p__unclassified_k__Fungi	c__unclassified_k__Fungi	o__unclassified_k__Fungi	f__unclassified_k__Fungi	g__unclassified_k__Fungi	s__unclassified_k__Fungi	OTU827
d__Eukaryota	k__Fungi	p__Ascomycota	c__Leotiomycetes	o__unclassified_c__Leotiomycetes	f__unclassified_c__Leotiomycetes	g__unclassified_c__Leotiomycetes	s__unclassified_c__Leotiomycetes	OTU598
d__Eukaryota	k__Fungi	p__Ascomycota	c__Dothideomycetes	o__Pleosporales	f__unclassified_o__Pleosporales	g__unclassified_o__Pleosporales	s__Pleosporales_sp	OTU697
d__Eukaryota	k__Fungi	p__Ascomycota	c__Dothideomycetes	o__Pleosporales	f__unclassified_o__Pleosporales	g__unclassified_o__Pleosporales	s__unclassified_o__Pleosporales	OTU1113
d__Eukaryota	k__Fungi	p__Ascomycota	c__Pezizomycotina_cls_Incertae_sedis	o__Pezizomycotina_ord_Incertae_sedis	f__Pezizomycotina_fam_Incertae_sedis	g__Ciliophora	s__Ciliophora_sp	OTU1051
d__Eukaryota	k__Fungi	p__Basidiomycota	c__Agaricomycetes	o__Agaricales	f__Tricholomataceae	g__Dendrocollybia	s__Dendrocollybia_sp	OTU62
d__Eukaryota	k__Fungi	p__Ascomycota	c__unclassified_p__Ascomycota	o__unclassified_p__Ascomycota	f__unclassified_p__Ascomycota	g__unclassified_p__Ascomycota	s__unclassified_p__Ascomycota	OTU923
d__Eukaryota	k__Fungi	p__Ascomycota	c__Dothideomycetes	o__Pleosporales	f__Pleosporaceae	g__Curvularia	s__Curvularia_hawaiiensis	OTU625
d__Eukaryota	k__Fungi	p__Ascomycota	c__unclassified_p__Ascomycota	o__unclassified_p__Ascomycota	f__unclassified_p__Ascomycota	g__unclassified_p__Ascomycota	s__unclassified_p__Ascomycota	OTU920
d__Eukaryota	k__Fungi	p__unclassified_k__Fungi	c__unclassified_k__Fungi	o__unclassified_k__Fungi	f__unclassified_k__Fungi	g__unclassified_k__Fungi	s__unclassified_k__Fungi	OTU438
d__Eukaryota	k__Fungi	p__Ascomycota	c__unclassified_p__Ascomycota	o__unclassified_p__Ascomycota	f__unclassified_p__Ascomycota	g__unclassified_p__Ascomycota	s__unclassified_p__Ascomycota	OTU925
d__Eukaryota	k__Fungi	p__Basidiomycota	c__Agaricomycetes	o__Trechisporales	f__Hydnodontaceae	g__Trechispora	s__unclassified_g__Trechispora	OTU28
d__Eukaryota	k__Fungi	p__Ascomycota	c__Leotiomycetes	o__Helotiales	f__Helotiaceae	g__Articulospora	s__Articulospora_sp	OTU147
d__Eukaryota	k__Fungi	p__Ascomycota	c__Leotiomycetes	o__Helotiales	f__Helotiaceae	g__Calyptrozyma	s__Calyptrozyma_sp	OTU928
d__Eukaryota	k__Fungi	p__Ascomycota	c__Sordariomycetes	o__Trichosphaeriales	f__Trichosphaeriaceae	g__Nigrospora	s__Nigrospora_oryzae	OTU406
d__Eukaryota	k__Fungi	p__Ascomycota	c__Lecanoromycetes	o__Ostropales	f__Stictidaceae	g__Schizoxylon	s__Schizoxylon_berkeleyanum	OTU253
d__Eukaryota	k__Fungi	p__Basidiomycota	c__Agaricomycetes	o__Agaricales	f__Psathyrellaceae	g__Coprinopsis	s__Coprinopsis_jonesii	OTU800
d__Eukaryota	k__Fungi	p__Ascomycota	c__Orbiliomycetes	o__Orbiliales	f__Orbiliales_fam_Incertae_sedis	g__Lecophagus	s__unclassified_g__Lecophagus	OTU1012
d__Eukaryota	k__Fungi	p__Ascomycota	c__unclassified_p__Ascomycota	o__unclassified_p__Ascomycota	f__unclassified_p__Ascomycota	g__unclassified_p__Ascomycota	s__unclassified_p__Ascomycota	OTU372
d__Eukaryota	k__Fungi	p__Ascomycota	c__Dothideomycetes	o__Pleosporales	f__Amniculicolaceae	g__Murispora	s__Murispora_galii	OTU1215
d__Eukaryota	k__Fungi	p__Chytridiomycota	c__Spizellomycetes	o__Spizellomycetales	f__Powellomycetaceae	g__Powellomyces	s__Powellomyces_hirtus	OTU1103
d__Eukaryota	k__Fungi	p__Ascomycota	c__Dothideomycetes	o__Pleosporales	f__unclassified_o__Pleosporales	g__unclassified_o__Pleosporales	s__unclassified_o__Pleosporales	OTU1156
d__Eukaryota	k__Fungi	p__Basidiomycota	c__Microbotryomycetes	o__unclassified_c__Microbotryomycetes	f__unclassified_c__Microbotryomycetes	g__unclassified_c__Microbotryomycetes	s__unclassified_c__Microbotryomycetes	OTU1140
d__Eukaryota	k__Fungi	p__Basidiomycota	c__Cystobasidiomycetes	o__Cystobasidiomycetes_ord_Incertae_sedis	f__Buckleyzymaceae	g__Buckleyzyma	s__Buckleyzyma_aurantiaca	OTU1220
d__Eukaryota	k__Fungi	p__Basidiomycota	c__Microbotryomycetes	o__unclassified_c__Microbotryomycetes	f__unclassified_c__Microbotryomycetes	g__unclassified_c__Microbotryomycetes	s__Microbotryomycetes_sp	OTU1238
d__Eukaryota	k__Fungi	p__Mortierellomycota	c__Mortierellomycetes	o__Mortierellales	f__Mortierellaceae	g__Mortierella	s__Mortierella_exigua	OTU1069
d__Eukaryota	k__Fungi	p__unclassified_k__Fungi	c__unclassified_k__Fungi	o__unclassified_k__Fungi	f__unclassified_k__Fungi	g__unclassified_k__Fungi	s__unclassified_k__Fungi	OTU222

Domain	Kingdom	Phylum	Class	Order	Family	Genus	Species	OTU
d__Eukaryota	k__Fungi	p__Ascomycota	c__unclassified_p__Ascomycota	o__unclassified_p__Ascomycota	f__unclassified_p__Ascomycota	g__unclassified_p__Ascomycota	s__unclassified_p__Ascomycota	OTU378
d__Eukaryota	k__Fungi	p__unclassified_k__Fungi	c__unclassified_k__Fungi	o__unclassified_k__Fungi	f__unclassified_k__Fungi	g__unclassified_k__Fungi	s__unclassified_k__Fungi	OTU549
d__Eukaryota	k__Fungi	p__Ascomycota	c__Dothideomycetes	o__Pleosporales	f__Sporormiaceae	g__Preussia	s__Preussia_pilosella	OTU111
d__Eukaryota	k__Fungi	p__unclassified_k__Fungi	c__unclassified_k__Fungi	o__unclassified_k__Fungi	f__unclassified_k__Fungi	g__unclassified_k__Fungi	s__unclassified_k__Fungi	OTU225
d__Eukaryota	k__Fungi	p__Ascomycota	c__Dothideomycetes	o__Capnodiales	f__Mycosphaerellaceae	g__Septoria	s__Septoria_tanaceti	OTU1203
d__Eukaryota	k__Fungi	p__Ascomycota	c__Lecanoromycetes	o__Lecanorales	f__unclassified_o__Lecanorales	g__unclassified_o__Lecanorales	s__unclassified_o__Lecanorales	OTU737
d__Eukaryota	k__Fungi	p__Ascomycota	c__Leotiomycetes	o__Helotiales	f__unclassified_o__Helotiales	g__unclassified_o__Helotiales	s__unclassified_o__Helotiales	OTU634
d__Eukaryota	k__Fungi	p__Ascomycota	c__Sordariomycetes	o__Hypocreales	f__Ophiocordycipitaceae	g__Haptocillium	s__unclassified_g__Haptocillium	OTU1226
d__Eukaryota	k__Fungi	p__Ascomycota	c__unclassified_p__Ascomycota	o__unclassified_p__Ascomycota	f__unclassified_p__Ascomycota	g__unclassified_p__Ascomycota	s__unclassified_p__Ascomycota	OTU1053
d__Eukaryota	k__Fungi	p__Basidiomycota	c__Agaricomycetes	o__Thelephorales	f__Thelephoraceae	g__Tomentella	s__Tomentella_subtestacea	OTU3
d__Eukaryota	k__Fungi	p__unclassified_k__Fungi	c__unclassified_k__Fungi	o__unclassified_k__Fungi	f__unclassified_k__Fungi	g__unclassified_k__Fungi	s__unclassified_k__Fungi	OTU479
d__Eukaryota	k__Fungi	p__Chytridiomycota	c__Rhizophydiomycetes	o__Rhizophydiales	f__unclassified_o__Rhizophydiales	g__unclassified_o__Rhizophydiales	s__unclassified_o__Rhizophydiales	OTU390
d__Eukaryota	k__Fungi	p__Ascomycota	c__Leotiomycetes	o__Helotiales	f__Helotiaceae	g__Ascocoryne	s__Ascocoryne_cylichnium	OTU294
d__Eukaryota	k__Fungi	p__Ascomycota	c__unclassified_p__Ascomycota	o__unclassified_p__Ascomycota	f__unclassified_p__Ascomycota	g__unclassified_p__Ascomycota	s__unclassified_p__Ascomycota	OTU156
d__Eukaryota	k__Fungi	p__Ascomycota	c__Leotiomycetes	o__Thelebolales	f__Pseudeurotiaceae	g__Pseudogymnoascus	s__Pseudogymnoascus_sp	OTU275
d__Eukaryota	k__Fungi	p__Ascomycota	c__Sordariomycetes	o__Hypocreales	f__Nectriaceae	g__Volutella	s__Volutella_ciliata	OTU1130
d__Eukaryota	k__Fungi	p__Basidiomycota	c__Tremellomycetes	o__Tremellales	f__Bulleribasidiaceae	g__Dioszegia	s__Dioszegia_aurantiaca	OTU295
d__Eukaryota	k__Fungi	p__Basidiomycota	c__unclassified_p__Basidiomycota	o__unclassified_p__Basidiomycota	f__unclassified_p__Basidiomycota	g__unclassified_p__Basidiomycota	s__unclassified_p__Basidiomycota	OTU104
d__Eukaryota	k__Fungi	p__Ascomycota	c__Leotiomycetes	o__Helotiales	f__Myxotrichaceae	g__unclassified_f__Myxotrichaceae	s__unclassified_f__Myxotrichaceae	OTU1258
d__Eukaryota	k__Fungi	p__Basidiomycota	c__Agaricomycetes	o__Agaricales	f__Pterulaceae	g__Radulomyces	s__Radulomyces_confluens	OTU643
d__Eukaryota	k__Fungi	p__Ascomycota	c__Leotiomycetes	o__unclassified_c__Leotiomycetes	f__unclassified_c__Leotiomycetes	g__unclassified_c__Leotiomycetes	s__unclassified_c__Leotiomycetes	OTU975
d__Eukaryota	k__Fungi	p__Ascomycota	c__Sordariomycetes	o__Sordariales	f__Helminthosphaeriaceae	g__Endophragmiella	s__Endophragmiella_constricta	OTU818
d__Eukaryota	k__Fungi	p__Basidiomycota	c__Agaricomycetes	o__Russulales	f__Gloeocystidiellaceae	g__Gloeocystidiellum	s__unclassified_g__Gloeocystidiellum	OTU475
d__Eukaryota	k__Fungi	p__Ascomycota	c__unclassified_p__Ascomycota	o__unclassified_p__Ascomycota	f__unclassified_p__Ascomycota	g__unclassified_p__Ascomycota	s__unclassified_p__Ascomycota	OTU1133
d__Eukaryota	k__Fungi	p__unclassified_k__Fungi	c__unclassified_k__Fungi	o__unclassified_k__Fungi	f__unclassified_k__Fungi	g__unclassified_k__Fungi	s__unclassified_k__Fungi	OTU917
d__Eukaryota	k__Fungi	p__Ascomycota	c__Sordariomycetes	o__unclassified_c__Sordariomycetes	f__unclassified_c__Sordariomycetes	g__unclassified_c__Sordariomycetes	s__unclassified_c__Sordariomycetes	OTU821
d__Eukaryota	k__Fungi	p__Ascomycota	c__Sordariomycetes	o__Pleurotheciales	f__unclassified_o__Pleurotheciales	g__unclassified_o__Pleurotheciales	s__Pleurotheciales_sp	OTU1003
d__Eukaryota	k__Fungi	p__Basidiomycota	c__Tremellomycetes	o__Holtermanniales	f__Holtermanniales_fam_Incertae_sedis	g__Holtermanniella	s__Holtermanniella_sp	OTU321
d__Eukaryota	k__Fungi	p__Basidiomycota	c__Wallemiomycetes	o__Wallemiales	f__unclassified_o__Wallemiales	g__unclassified_o__Wallemiales	s__Wallemiales_sp	OTU364
d__Eukaryota	k__Fungi	p__Basidiomycota	c__Agaricomycetes	o__Cantharellales	f__Cantharellales_fam_Incertae_sedis	g__Sistotrema	s__Sistotrema_confluens	OTU678
d__Eukaryota	k__Fungi	p__Ascomycota	c__Dothideomycetes	o__Pleosporales	f__Periconiaceae	g__Periconia	s__Periconia_echinochloae	OTU172

**Domain**	**Kingdom**	**Phylum**	**Class**	**Order**	**Family**	**Genus**	**Species**	**OTU**
d__Eukaryota	k__Fungi	p__Ascomycota	c__Arthoniomycetes	o__Lichenostigmatales	f__Phaeococcomycetaceae	g__Phaeococcomyces	s__unclassified_g__Phaeococcomyces	OTU705
d__Eukaryota	k__Fungi	p__Ascomycota	c__Leotiomycetes	o__Thelebolales	f__Pseudeurotiaceae	g__Leuconeurospora	s__Leuconeurospora_sp	OTU418
d__Eukaryota	k__Fungi	p__Mortierellomycota	c__Mortierellomycetes	o__Mortierellales	f__Mortierellaceae	g__Mortierella	s__Mortierella_alpina	OTU446
d__Eukaryota	k__Fungi	p__Basidiomycota	c__Microbotryomycetes	o__Kriegeriales	f__Kriegeriaceae	g__Phenoliferia	s__unclassified_g__Phenoliferia	OTU359
d__Eukaryota	k__Fungi	p__Basidiomycota	c__Agaricomycetes	o__Trechisporales	f__Hydnodontaceae	g__Trechispora	s__Trechispora_microspora	OTU595
d__Eukaryota	k__Fungi	p__Ascomycota	c__Sordariomycetes	o__Coniochaetales	f__Coniochaetaceae	g__Coniochaeta	s__Coniochaeta_sp	OTU773
d__Eukaryota	k__Fungi	p__Ascomycota	c__Dothideomycetes	o__Pleosporales	f__Lophiostomataceae	g__unclassified_f__Lophiostomataceae	s__unclassified_f__Lophiostomataceae	OTU522
d__Eukaryota	k__Fungi	p__Ascomycota	c__Sordariomycetes	o__Microascales	f__Halosphaeriaceae	g__Cirrenalia	s__unclassified_g__Cirrenalia	OTU1044
d__Eukaryota	k__Fungi	p__Ascomycota	c__Dothideomycetes	o__Venturiales	f__Venturiaceae	g__Venturia	s__unclassified_g__Venturia	OTU297
d__Eukaryota	k__Fungi	p__Ascomycota	c__Leotiomycetes	o__unclassified_c__Leotiomycetes	f__unclassified_c__Leotiomycetes	g__unclassified_c__Leotiomycetes	s__unclassified_c__Leotiomycetes	OTU1175
d__Eukaryota	k__Fungi	p__Basidiomycota	c__Agaricomycetes	o__Polyporales	f__Meruliaceae	g__Phlebia	s__unclassified_g__Phlebia	OTU688
d__Eukaryota	k__Fungi	p__Ascomycota	c__Lecanoromycetes	o__Lecanorales	f__Lecanoraceae	g__unclassified_f__Lecanoraceae	s__unclassified_f__Lecanoraceae	OTU302
d__Eukaryota	k__Fungi	p__Ascomycota	c__Dothideomycetes	o__Capnodiales	f__Teratosphaeriaceae	g__Oleoguttula	s__Oleoguttula_mirabilis	OTU75
d__Eukaryota	k__Fungi	p__Ascomycota	c__Dothideomycetes	o__Pleosporales	f__Melanommataceae	g__unclassified_f__Melanommataceae	s__unclassified_f__Melanommataceae	OTU1127
d__Eukaryota	k__Fungi	p__unclassified_k__Fungi	c__unclassified_k__Fungi	o__unclassified_k__Fungi	f__unclassified_k__Fungi	g__unclassified_k__Fungi	s__unclassified_k__Fungi	OTU1070
d__Eukaryota	k__Fungi	p__Ascomycota	c__Leotiomycetes	o__Helotiales	f__unclassified_o__Helotiales	g__unclassified_o__Helotiales	s__unclassified_o__Helotiales	OTU842
d__Eukaryota	k__Fungi	p__Ascomycota	c__Dothideomycetes	o__Pleosporales	f__unclassified_o__Pleosporales	g__unclassified_o__Pleosporales	s__unclassified_o__Pleosporales	OTU790
d__Eukaryota	k__Fungi	p__Ascomycota	c__Sordariomycetes	o__Hypocreales	f__Hypocreales_fam_Incertae_sedis	g__Fusariella	s__unclassified_g__Fusariella	OTU1205
d__Eukaryota	k__Fungi	p__Basidiomycota	c__Agaricomycetes	o__Agaricales	f__Lycoperdaceae	g__Lycoperdon	s__Lycoperdon_pyriforme	OTU588
d__Eukaryota	k__Fungi	p__Mortierellomycota	c__Mortierellomycetes	o__Mortierellales	f__Mortierellaceae	g__Mortierella	s__Mortierella_sarnyensis	OTU689
d__Eukaryota	k__Fungi	p__unclassified_k__Fungi	c__unclassified_k__Fungi	o__unclassified_k__Fungi	f__unclassified_k__Fungi	g__unclassified_k__Fungi	s__unclassified_k__Fungi	OTU327
d__Eukaryota	k__Fungi	p__Basidiomycota	c__Agaricomycetes	o__Polyporales	f__Steccherinaceae	g__Junghuhnia	s__Junghuhnia_nitida	OTU1143
d__Eukaryota	k__Fungi	p__Fungi_phy_Incertae_sedis	c__Fungi_cls_Incertae_sedis	o__Fungi_ord_Incertae_sedis	f__Fungi_fam_Incertae_sedis	g__Desmococcus	s__Desmococcus_sp	OTU78
d__Eukaryota	k__Fungi	p__unclassified_k__Fungi	c__unclassified_k__Fungi	o__unclassified_k__Fungi	f__unclassified_k__Fungi	g__unclassified_k__Fungi	s__unclassified_k__Fungi	OTU1118
d__Eukaryota	k__Fungi	p__unclassified_k__Fungi	c__unclassified_k__Fungi	o__unclassified_k__Fungi	f__unclassified_k__Fungi	g__unclassified_k__Fungi	s__unclassified_k__Fungi	OTU220
d__Eukaryota	k__Fungi	p__Ascomycota	c__Dothideomycetes	o__Capnodiales	f__Mycosphaerellaceae	g__Mycocentrospora	s__Mycocentrospora_acerina	OTU1090
d__Eukaryota	k__Fungi	p__Ascomycota	c__Eurotiomycetes	o__Chaetothyriales	f__Herpotrichiellaceae	g__Exophiala	s__Exophiala_sp	OTU260
d__Eukaryota	k__Fungi	p__Basidiomycota	c__Agaricomycetes	o__Agaricales	f__Lyophyllaceae	g__Tephrocybe	s__Tephrocybe_putida	OTU503
d__Eukaryota	k__Fungi	p__unclassified_k__Fungi	c__unclassified_k__Fungi	o__unclassified_k__Fungi	f__unclassified_k__Fungi	g__unclassified_k__Fungi	s__unclassified_k__Fungi	OTU18
d__Eukaryota	k__Fungi	p__Ascomycota	c__Leotiomycetes	o__Helotiales	f__Vibrisseaceae	g__Phialocephala	s__Phialocephala_fortinii	OTU896
d__Eukaryota	k__Fungi	p__Ascomycota	c__Leotiomycetes	o__Helotiales	f__Hyaloscyphaceae	g__Polydesmia	s__Polydesmia_fructicola	OTU1045

Domain	Kingdom	Phylum	Class	Order	Family	Genus	Species	OTU
d__Eukaryota	k__Fungi	p__Ascomycota	c__Eurotiomycetes	o__Chaetothyriales	f__Herpotrichiellaceae	g__Exophiala	s__unclassified_g__Exophiala	OTU399
d__Eukaryota	k__Fungi	p__Basidiomycota	c__Agaricomycetes	o__Hymenochaetales	f__Rickenellaceae	g__Rickenella	s__Rickenella_fibula	OTU530
d__Eukaryota	k__Fungi	p__Ascomycota	c__Dothideomycetes	o__Pleosporales	f__unclassified_o__Pleosporales	g__unclassified_o__Pleosporales	s__unclassified_o__Pleosporales	OTU879
d__Eukaryota	k__Fungi	p__Ascomycota	c__Lecanoromycetes	o__Acarosporales	f__Acarosporaceae	g__Polysporina	s__Polysporina_subfuscescens	OTU538
d__Eukaryota	k__Fungi	p__unclassified_k__Fungi	c__unclassified_k__Fungi	o__unclassified_k__Fungi	f__unclassified_k__Fungi	g__unclassified_k__Fungi	s__unclassified_k__Fungi	OTU1235
d__Eukaryota	k__Fungi	p__unclassified_k__Fungi	c__unclassified_k__Fungi	o__unclassified_k__Fungi	f__unclassified_k__Fungi	g__unclassified_k__Fungi	s__unclassified_k__Fungi	OTU1236
d__Eukaryota	k__Fungi	p__Ascomycota	c__Dothideomycetes	o__Pleosporales	f__Didymosphaeriaceae	g__Pseudopithomyces	s__Pseudopithomyces_palmicola	OTU184
d__Eukaryota	k__Fungi	p__Ascomycota	c__Leotiomycetes	o__unclassified_c__Leotiomycetes	f__unclassified_c__Leotiomycetes	g__unclassified_c__Leotiomycetes	s__unclassified_c__Leotiomycetes	OTU817
d__Eukaryota	k__Fungi	p__Ascomycota	c__unclassified_p__Ascomycota	o__unclassified_p__Ascomycota	f__unclassified_p__Ascomycota	g__unclassified_p__Ascomycota	s__unclassified_p__Ascomycota	OTU988
d__Eukaryota	k__Fungi	p__Ascomycota	c__Dothideomycetes	o__Pleosporales	f__Leptosphaeriaceae	g__Leptosphaeria	s__unclassified_g__Leptosphaeria	OTU1217
d__Eukaryota	k__Fungi	p__Ascomycota	c__unclassified_p__Ascomycota	o__unclassified_p__Ascomycota	f__unclassified_p__Ascomycota	g__unclassified_p__Ascomycota	s__unclassified_p__Ascomycota	OTU593
d__Eukaryota	k__Fungi	p__Ascomycota	c__Dothideomycetes	o__Capnodiales	f__Mycosphaerellaceae	g__Sphaerulina	s__Sphaerulina_berberidis	OTU927
d__Eukaryota	k__Fungi	p__Ascomycota	c__Pezizomycotina_cls_Incertae_sedis	o__Pezizomycotina_ord_Incertae_sedis	f__Pezizomycotina_fam_Incertae_sedis	g__Ciliophora	s__Ciliophora_sp	OTU874
d__Eukaryota	k__Fungi	p__unclassified_k__Fungi	c__unclassified_k__Fungi	o__unclassified_k__Fungi	f__unclassified_k__Fungi	g__unclassified_k__Fungi	s__unclassified_k__Fungi	OTU1239
d__Eukaryota	k__Fungi	p__Basidiomycota	c__Agaricomycetes	o__Agaricales	f__Lyophyllaceae	g__Tephrocybe	s__Tephrocybe_anthracophila	OTU352
d__Eukaryota	k__Fungi	p__Chytridiomycota	c__Rhizophydiomycetes	o__Rhizophydiales	f__unclassified_o__Rhizophydiales	g__unclassified_o__Rhizophydiales	s__unclassified_o__Rhizophydiales	OTU1114
d__Eukaryota	k__Fungi	p__Ascomycota	c__Dothideomycetes	o__Pleosporales	f__Periconiaceae	g__Periconia	s__Periconia_minutissima	OTU921
d__Eukaryota	k__Fungi	p__Ascomycota	c__Leotiomycetes	o__Helotiales	f__unclassified_o__Helotiales	g__unclassified_o__Helotiales	s__unclassified_o__Helotiales	OTU1172
d__Eukaryota	k__Fungi	p__Basidiomycota	c__Agaricomycetes	o__Thelephorales	f__Thelephoraceae	g__Tomentella	s__Tomentella_sp	OTU596
d__Eukaryota	k__Fungi	p__Ascomycota	c__Sordariomycetes	o__Hypocreales	f__Nectriaceae	g__Fusarium	s__Fusarium_asiaticum	OTU267
d__Eukaryota	k__Fungi	p__Ascomycota	c__Leotiomycetes	o__Helotiales	f__Leotiaceae	g__Pezoloma	s__Pezoloma_ericae	OTU883
d__Eukaryota	k__Fungi	p__unclassified_k__Fungi	c__unclassified_k__Fungi	o__unclassified_k__Fungi	f__unclassified_k__Fungi	g__unclassified_k__Fungi	s__unclassified_k__Fungi	OTU223
d__Eukaryota	k__Fungi	p__unclassified_k__Fungi	c__unclassified_k__Fungi	o__unclassified_k__Fungi	f__unclassified_k__Fungi	g__unclassified_k__Fungi	s__unclassified_k__Fungi	OTU854
d__Eukaryota	k__Fungi	p__Ascomycota	c__Eurotiomycetes	o__Verrucariales	f__Verrucariaceae	g__Verrucaria	s__Verrucaria_margacea	OTU1209
d__Eukaryota	k__Fungi	p__Ascomycota	c__unclassified_p__Ascomycota	o__unclassified_p__Ascomycota	f__unclassified_p__Ascomycota	g__unclassified_p__Ascomycota	s__unclassified_p__Ascomycota	OTU753
d__Eukaryota	k__Fungi	p__Ascomycota	c__Arthoniomycetes	o__Lichenostigmatales	f__unclassified_o__Lichenostigmatales	g__unclassified_o__Lichenostigmatales	s__Lichenostigmatales_sp	OTU1004
d__Eukaryota	k__Fungi	p__unclassified_k__Fungi	c__unclassified_k__Fungi	o__unclassified_k__Fungi	f__unclassified_k__Fungi	g__unclassified_k__Fungi	s__unclassified_k__Fungi	OTU683
d__Eukaryota	k__Fungi	p__Ascomycota	c__Dothideomycetes	o__Botryosphaeriales	f__Botryosphaeriaceae	g__Phaeobotryon	s__unclassified_g__Phaeobotryon	OTU953
d__Eukaryota	k__Fungi	p__Basidiomycota	c__Agaricomycetes	o__Hymenochaetales	f__Schizoporaceae	g__Hyphodontia	s__Hyphodontia_pallidula	OTU848
d__Eukaryota	k__Fungi	p__Ascomycota	c__Dothideomycetes	o__Pleosporales	f__Pleomassariaceae	g__unclassified_f__Pleomassariaceae	s__unclassified_f__Pleomassariaceae	OTU336
d__Eukaryota	k__Fungi	p__unclassified_k__Fungi	c__unclassified_k__Fungi	o__unclassified_k__Fungi	f__unclassified_k__Fungi	g__unclassified_k__Fungi	s__unclassified_k__Fungi	OTU729

Domain	Kingdom	Phylum	Class	Order	Family	Genus	Species	OTU
d__Eukaryota	k__Fungi	p__unclassified_k__Fungi	c__unclassified_k__Fungi	o__unclassified_k__Fungi	f__unclassified_k__Fungi	g__unclassified_k__Fungi	s__unclassified_k__Fungi	OTU856
d__Eukaryota	k__Fungi	p__Basidiomycota	c__Agaricomycetes	o__Agaricales	f__Lycoperdaceae	g__Lycoperdon	s__Lycoperdon_nigrescens	OTU157
d__Eukaryota	k__Fungi	p__Ascomycota	c__Eurotiomycetes	o__Sclerococcales	f__Sclerococcaceae	g__Sclerococcum	s__Sclerococcum_simplex	OTU618
d__Eukaryota	k__Fungi	p__Ascomycota	c__Eurotiomycetes	o__Chaetothyriales	f__unclassified_o__Chaetothyriales	g__unclassified_o__Chaetothyriales	s__Chaetothyriales_sp	OTU711
d__Eukaryota	k__Fungi	p__Ascomycota	c__Sordariomycetes	o__Myrmecridiales	f__Myrmecridiaceae	g__Myrmecridium	s__Myrmecridium_schulzeri	OTU1186
d__Eukaryota	k__Fungi	p__Ascomycota	c__Sordariomycetes	o__Xylariales	f__Xylariaceae	g__Nemania	s__Nemania_beaumontii	OTU714
d__Eukaryota	k__Fungi	p__Ascomycota	c__Leotiomycetes	o__unclassified_c__Leotiomycetes	f__unclassified_c__Leotiomycetes	g__unclassified_c__Leotiomycetes	s__unclassified_c__Leotiomycetes	OTU894
d__Eukaryota	k__Fungi	p__Basidiomycota	c__Agaricostilbomycetes	o__unclassified_c__Agaricostilbomycetes	f__unclassified_c__Agaricostilbomycetes	g__unclassified_c__Agaricostilbomycetes	s__unclassified_c__Agaricostilbomycetes	OTU200
d__Eukaryota	k__Fungi	p__Ascomycota	c__Dothideomycetes	o__Pleosporales	f__Leptosphaeriaceae	g__Leptosphaeria	s__unclassified_g__Leptosphaeria	OTU601
d__Eukaryota	k__Fungi	p__unclassified_k__Fungi	c__unclassified_k__Fungi	o__unclassified_k__Fungi	f__unclassified_k__Fungi	g__unclassified_k__Fungi	s__unclassified_k__Fungi	OTU1129
d__Eukaryota	k__Fungi	p__unclassified_k__Fungi	c__unclassified_k__Fungi	o__unclassified_k__Fungi	f__unclassified_k__Fungi	g__unclassified_k__Fungi	s__unclassified_k__Fungi	OTU859
d__Eukaryota	k__Fungi	p__Ascomycota	c__Dothideomycetes	o__Pleosporales	f__Melanommataceae	g__Pleotrichocladium	s__Pleotrichocladium_opacum	OTU846
d__Eukaryota	k__Fungi	p__Ascomycota	c__Sordariomycetes	o__Xylariales	f__Microdochiaceae	g__Microdochium	s__Microdochium_bolleyi	OTU385
d__Eukaryota	k__Fungi	p__Ascomycota	c__Lecanoromycetes	o__Trapeliales	f__Trapeliaceae	g__Trapelia	s__Trapelia_sp	OTU934
d__Eukaryota	k__Fungi	p__Basidiomycota	c__Microbotryomycetes	o__Microbotryomycetes_ord_Incertae_sedis	f__unclassified_o__Microbotryomycetes_ord_Incertae_sedis	g__unclassified_o__Microbotryomycetes_ord_Incertae_sedis	s__unclassified_o__Microbotryomycetes_ord_Incertae_sedis	OTU662
d__Eukaryota	k__Fungi	p__Ascomycota	c__Sordariomycetes	o__Hypocreales	f__Tilachlidiaceae	g__unclassified_f__Tilachlidiaceae	s__unclassified_f__Tilachlidiaceae	OTU1024
d__Eukaryota	k__Fungi	p__Ascomycota	c__Eurotiomycetes	o__Chaetothyriales	f__unclassified_o__Chaetothyriales	g__unclassified_o__Chaetothyriales	s__unclassified_o__Chaetothyriales	OTU1211
d__Eukaryota	k__Fungi	p__unclassified_k__Fungi	c__unclassified_k__Fungi	o__unclassified_k__Fungi	f__unclassified_k__Fungi	g__unclassified_k__Fungi	s__unclassified_k__Fungi	OTU1040
d__Eukaryota	k__Fungi	p__Basidiomycota	c__Microbotryomycetes	o__unclassified_c__Microbotryomycetes	f__unclassified_c__Microbotryomycetes	g__unclassified_c__Microbotryomycetes	s__Microbotryomycetes_sp	OTU970
d__Eukaryota	k__Fungi	p__Ascomycota	c__Eurotiomycetes	o__Verrucariales	f__Verrucariaceae	g__unclassified_f__Verrucariaceae	s__unclassified_f__Verrucariaceae	OTU776
d__Eukaryota	k__Fungi	p__Ascomycota	c__unclassified_p__Ascomycota	o__unclassified_p__Ascomycota	f__unclassified_p__Ascomycota	g__unclassified_p__Ascomycota	s__unclassified_p__Ascomycota	OTU1009
d__Eukaryota	k__Fungi	p__Ascomycota	c__Sordariomycetes	o__Sordariales	f__Lasiosphaeriaceae	g__Apodus	s__Apodus_sp	OTU1057
d__Eukaryota	k__Fungi	p__Ascomycota	c__Lecanoromycetes	o__Lecanorales	f__unclassified_o__Lecanorales	g__unclassified_o__Lecanorales	s__unclassified_o__Lecanorales	OTU110
d__Eukaryota	k__Fungi	p__Ascomycota	c__Sordariomycetes	o__Xylariales	f__Xylariaceae	g__Hypoxylon	s__Hypoxylon_perforatum	OTU210
d__Eukaryota	k__Fungi	p__Ascomycota	c__Leotiomycetes	o__Helotiales	f__Helotiaceae	g__Tetracladium	s__Tetracladium_sp	OTU27
d__Eukaryota	k__Fungi	p__Ascomycota	c__unclassified_p__Ascomycota	o__unclassified_p__Ascomycota	f__unclassified_p__Ascomycota	g__unclassified_p__Ascomycota	s__unclassified_p__Ascomycota	OTU158
d__Eukaryota	k__Fungi	p__Ascomycota	c__Sordariomycetes	o__Sordariales	f__Lasiosphaeriaceae	g__Schizothecium	s__unclassified_g__Schizothecium	OTU1128
d__Eukaryota	k__Fungi	p__Ascomycota	c__Dothideomycetes	o__Capnodiales	f__Teratosphaeriaceae	g__unclassified_f__Teratosphaeriaceae	s__unclassified_f__Teratosphaeriaceae	OTU901
d__Eukaryota	k__Fungi	p__Basidiomycota	c__Microbotryomycetes	o__Microbotryomycetes_ord_Incertae_sedi	f__unclassified_o__Microbotryomycetes	g__unclassified_o__Microbotryomycetes_	s__unclassified_o__Microbotryomycetes_o	OTU245

Domain	Kingdom	Phylum	Class	Order	Family	Genus	Species	OTU
				s	_ord_Incertae_sedis	ord_Incertae_sedis	rd_Incertae_sedis	
d__Eukaryota	k__Fungi	p__Ascomycota	c__Dothideomycetes	o__Capnodiales	f__Extremaceae	g__unclassified_f__Extremaceae	s__Extremaceae_sp	OTU1098
d__Eukaryota	k__Fungi	p__Ascomycota	c__Dothideomycetes	o__Valsariales	f__Valsariaceae	g__Myrmaecium	s__Myrmaecium_fulvopruinatum	OTU803
d__Eukaryota	k__Fungi	p__Ascomycota	c__Sordariomycetes	o__Glomerellales	f__Plectosphaerellaceae	g__Gibellulopsis	s__Gibellulopsis_piscis	OTU1091
d__Eukaryota	k__Fungi	p__Basidiomycota	c__Tremellomycetes	o__Cystofilobasidiales	f__Mrakiaceae	g__Mrakia	s__Mrakia_frigida	OTU350
d__Eukaryota	k__Fungi	p__Basidiomycota	c__Agaricomycetes	o__Russulales	f__Peniophoraceae	g__unclassified_f__Peniophoraceae	s__Peniophoraceae_sp	OTU138
d__Eukaryota	k__Fungi	p__Basidiomycota	c__Wallemiomycetes	o__Wallemiales	f__unclassified_o__Wallemiales	g__unclassified_o__Wallemiales	s__Wallemiales_sp	OTU1001
d__Eukaryota	k__Fungi	p__Ascomycota	c__Leotiomycetes	o__Helotiales	f__Hyaloscyphaceae	g__Gyoerffyella	s__Gyoerffyella_sp	OTU828
d__Eukaryota	k__Fungi	p__Ascomycota	c__unclassified_p__Ascomycota	o__unclassified_p__Ascomycota	f__unclassified_p__Ascomycota	g__unclassified_p__Ascomycota	s__unclassified_p__Ascomycota	OTU155
d__Eukaryota	k__Fungi	p__Ascomycota	c__Pezizomycotina_cls_Incertae_sedis	o__Pezizomycotina_ord_Incertae_sedis	f__Pezizomycotina_fam_Incertae_sedis	g__Ciliophora	s__Ciliophora_sp	OTU1062
d__Eukaryota	k__Fungi	p__unclassified_k__Fungi	c__unclassified_k__Fungi	o__unclassified_k__Fungi	f__unclassified_k__Fungi	g__unclassified_k__Fungi	s__unclassified_k__Fungi	OTU535
d__Eukaryota	k__Fungi	p__Ascomycota	c__Leotiomycetes	o__Helotiales	f__Hyaloscyphaceae	g__unclassified_f__Hyaloscyphaceae	s__Hyaloscyphaceae_sp	OTU1002
d__Eukaryota	k__Fungi	p__unclassified_k__Fungi	c__unclassified_k__Fungi	o__unclassified_k__Fungi	f__unclassified_k__Fungi	g__unclassified_k__Fungi	s__unclassified_k__Fungi	OTU533
d__Eukaryota	k__Fungi	p__unclassified_k__Fungi	c__unclassified_k__Fungi	o__unclassified_k__Fungi	f__unclassified_k__Fungi	g__unclassified_k__Fungi	s__unclassified_k__Fungi	OTU1193
d__Eukaryota	k__Fungi	p__Ascomycota	c__Dothideomycetes	o__Pleosporales	f__Phaeosphaeriaceae	g__Paraphoma	s__Paraphoma_radicina	OTU1049
d__Eukaryota	k__Fungi	p__Ascomycota	c__Eurotiomycetes	o__Eurotiales	f__Aspergillaceae	g__Aspergillus	s__Aspergillus_proliferans	OTU602
d__Eukaryota	k__Fungi	p__Basidiomycota	c__Agaricomycetes	o__Sebacinales	f__unclassified_o__Sebacinales	g__unclassified_o__Sebacinales	s__unclassified_o__Sebacinales	OTU834
d__Eukaryota	k__Fungi	p__Basidiomycota	c__Microbotryomycetes	o__Microbotryomycetes_ord_Incertae_sedis	f__Chrysozymaceae	g__Bannozyma	s__Bannozyma_sp	OTU211
d__Eukaryota	k__Fungi	p__Ascomycota	c__Dothideomycetes	o__Pleosporales	f__unclassified_o__Pleosporales	g__unclassified_o__Pleosporales	s__unclassified_o__Pleosporales	OTU108
d__Eukaryota	k__Fungi	p__Basidiomycota	c__Agaricomycetes	o__Agaricales	f__Crepidotaceae	g__Crepidotus	s__Crepidotus_mollis	OTU646
d__Eukaryota	k__Fungi	p__Ascomycota	c__Leotiomycetes	o__Helotiales	f__unclassified_o__Helotiales	g__unclassified_o__Helotiales	s__unclassified_o__Helotiales	OTU944
d__Eukaryota	k__Fungi	p__Ascomycota	c__Dothideomycetes	o__Capnodiales	f__Mycosphaerellaceae	g__unclassified_f__Mycosphaerellaceae	s__unclassified_f__Mycosphaerellaceae	OTU938
d__Eukaryota	k__Fungi	p__unclassified_k__Fungi	c__unclassified_k__Fungi	o__unclassified_k__Fungi	f__unclassified_k__Fungi	g__unclassified_k__Fungi	s__unclassified_k__Fungi	OTU63
d__Eukaryota	k__Fungi	p__Ascomycota	c__Leotiomycetes	o__unclassified_c__Leotiomycetes	f__unclassified_c__Leotiomycetes	g__unclassified_c__Leotiomycetes	s__Leotiomycetes_sp	OTU949
d__Eukaryota	k__Fungi	p__Ascomycota	c__Eurotiomycetes	o__Chaetothyriales	f__Trichomeriaceae	g__Bradymyces	s__Bradymyces_alpinus	OTU1251
d__Eukaryota	k__Fungi	p__Ascomycota	c__Leotiomycetes	o__Helotiales	f__Hyaloscyphaceae	g__Arachnopeziza	s__unclassified_g__Arachnopeziza	OTU1158
d__Eukaryota	k__Fungi	p__Ascomycota	c__unclassified_p__Ascomycota	o__unclassified_p__Ascomycota	f__unclassified_p__Ascomycota	g__unclassified_p__Ascomycota	s__unclassified_p__Ascomycota	OTU990
d__Eukaryota	k__Fungi	p__Ascomycota	c__Eurotiomycetes	o__Chaetothyriales	f__unclassified_o__Chaetothyriales	g__unclassified_o__Chaetothyriales	s__Chaetothyriales_sp	OTU1231
d__Eukaryota	k__Fungi	p__Fungi_phy_Incertae_sedis	c__Fungi_cls_Incertae_sedis	o__Fungi_ord_Incertae_sedis	f__Fungi_fam_Incertae_sedis	g__Desmococcus	s__Desmococcus_sp	OTU1119
d__Eukaryota	k__Fungi	p__Ascomycota	c__Dothideomycetes	o__Pleosporales	f__Sporormiaceae	g__Sporormiella	s__Sporormiella_minima	OTU979

**Domain**	**Kingdom**	**Phylum**	**Class**	**Order**	**Family**	**Genus**	**Species**	**OTU**
d__Eukaryota	k__Fungi	p__Ascomycota	c__unclassified_p__Ascomycota	o__unclassified_p__Ascomycota	f__unclassified_p__Ascomycota	g__unclassified_p__Ascomycota	s__unclassified_p__Ascomycota	OTU1097

Table S4. Rarefied bacterial OTUs table based on V3-V4 hypervariable region of the bacterial 16S rRNA gene Illumina Sequencing.

**Domain**	**Kingdom**	**Phylum**	**Class**	**Order**	**Family**	**Genus**	**Species**	**OTU**
d__Bacteria	k__norank_ d__Bacteria	p__Verrucomicrobiota	c__Verrucomicrobiae	o__Chthoniobacterales	f__Chthoniobacteraceae	g__Candidatus_Udaeobacter	s__uncultured_Verrucomicrobia_bacterium_g__Candidatus_Udaeobacter	OTU1162
d__Bacteria	k__norank_ d__Bacteria	p__Bacteroidota	c__Bacteroidia	o__Sphingobacteriales	f__AKYH767	g__norank_f__AKYH767	s__unclassified_g__norank_f__AKYH767	OTU417
d__Bacteria	k__norank_ d__Bacteria	p__Actinobacteriota	c__Actinobacteria	o__Propionibacteriales	f__Nocardioidaceae	g__Nocardioides	s__unclassified_g__Nocardioides	OTU1576
d__Bacteria	k__norank_ d__Bacteria	p__Proteobacteria	c__Alphaproteobacteria	o__Caulobacterales	f__Hyphomonadaceae	g__SWB02	s__uncultured_bacterium_g__SWB02	OTU2211
d__Bacteria	k__norank_ d__Bacteria	p__Chloroflexi	c__Ktedonobacteria	o__B12-WMSP1	f__norank_o__B12-WMSP1	g__norank_f__norank_o__B12-WMSP 1	s__uncultured_bacterium_g__norank_f__norank_o__B12-WMSP1	OTU970
d__Bacteria	k__norank_ d__Bacteria	p__Proteobacteria	c__Alphaproteobacteria	o__Rickettsiales	f__Rickettsiaceae	g__Candidatus_Megaira	s__uncultured_bacterium_g__Candidatus_Megaira	OTU2019
d__Bacteria	k__norank_ d__Bacteria	p__Proteobacteria	c__Gammaproteobacteria	o__Burkholderiales	f__Oxalobacteraceae	g__Aquaspirillum_arcticum_group	s__uncultured_bacterium_g__Aquaspirillum_arcticum_group	OTU2578
d__Bacteria	k__norank_ d__Bacteria	p__Proteobacteria	c__Alphaproteobacteria	o__Caulobacterales	f__Caulobacteraceae	g__Phenylobacterium	s__unclassified_g__Phenylobacterium	OTU2347
d__Bacteria	k__norank_ d__Bacteria	p__Proteobacteria	c__Gammaproteobacteria	o__Diplorickettsiales	f__Diplorickettsiaceae	g__Aquicella	s__uncultured_gamma_proteobacterium_g__Aquicella	OTU2133
d__Bacteria	k__norank_ d__Bacteria	p__Bdellovibrionota	c__Oligoflexia	o__0319-6G20	f__norank_o__0319-6G20	g__norank_f__norank_o__0319-6G20	s__unclassified_g__norank_f__norank_o__0319-6G20	OTU272
d__Bacteria	k__norank_ d__Bacteria	p__Proteobacteria	c__Gammaproteobacteria	o__Xanthomonadales	f__Xanthomonadaceae	g__Lysobacter	s__unclassified_g__Lysobacter	OTU2190
d__Bacteria	k__norank_ d__Bacteria	p__Proteobacteria	c__Gammaproteobacteria	o__Burkholderiales	f__Comamonadaceae	g__unclassified_f__Comamonadaceae	s__unclassified_f__Comamonadaceae	OTU750
d__Bacteria	k__norank_ d__Bacteria	p__Chloroflexi	c__Ktedonobacteria	o__Ktedonobacterales	f__Ktedonobacteraceae	g__norank_f__Ktedonobacteraceae	s__uncultured_bacterium_g__norank_f__Ktedonobacteraceae	OTU2034
d__Bacteria	k__norank_ d__Bacteria	p__Myxococcota	c__Polyangia	o__Polyangiales	f__BIrii41	g__norank_f__BIrii41	s__unclassified_g__norank_f__BIrii41	OTU2289
d__Bacteria	k__norank_ d__Bacteria	p__Elusimicrobiota	c__Elusimicrobia	o__Lineage_IV	f__norank_o__Lineage_IV	g__norank_f__norank_o__Lineage_IV	s__uncultured_bacterium_g__norank_f__norank_o__Lineage_IV	OTU789

d__Bacteria	k__norank_d__Bacteria	p__Gemmatimonadota	c__Gemmatimonadetes	o__Gemmatimonadales	f__Gemmatimonadaceae	g__norank_f__Gemmatimonadaceae	s__uncultured_Gemmatimonadetes_bacterium_g__norank_f__Gemmatimonadaceae	OTU1014
d__Bacteria	k__norank_d__Bacteria	p__Patescibacteria	c__Saccharimonadia	o__Saccharimonadales	f__Saccharimonadaceae	g__TM7a	s__uncultured_bacterium_g__TM7a	OTU2209
d__Bacteria	k__norank_d__Bacteria	p__Elusimicrobiota	c__Lineage_IIa	o__norank_c__Lineage_IIa	f__norank_o__norank_c__Lineage_IIa	g__norank_f__norank_o__norank_c__Lineage_IIa	s__uncultured_bacterium_g__norank_f__norank_o__norank_c__Lineage_IIa	OTU928
d__Bacteria	k__norank_d__Bacteria	p__Proteobacteria	c__Gammaproteobacteria	o__Burkholderiales	f__Comamonadaceae	g__norank_f__Comamonadaceae	s__Ideonella_sp._g__norank	OTU1706
d__Bacteria	k__norank_d__Bacteria	p__Proteobacteria	c__Alphaproteobacteria	o__Acetobacterales	f__Acetobacteraceae	g__norank_f__Acetobacteraceae	s__unclassified_g__norank_f__Acetobacteraceae	OTU1678
d__Bacteria	k__norank_d__Bacteria	p__Proteobacteria	c__Gammaproteobacteria	o__Burkholderiales	f__Nitrosomonadaceae	g__Ellin6067	s__unclassified_g__Ellin6067	OTU1255
d__Bacteria	k__norank_d__Bacteria	p__Proteobacteria	c__Gammaproteobacteria	o__Xanthomonadales	f__Xanthomonadaceae	g__unclassified_f__Xanthomonadaceae	s__unclassified_f__Xanthomonadaceae	OTU199
d__Bacteria	k__norank_d__Bacteria	p__Myxococcota	c__Polyangia	o__Polyangiales	f__Polyangiaceae	g__norank_f__Polyangiaceae	s__uncultured_delta_proteobacterium_g__norank_f__Polyangiaceae	OTU2705
d__Bacteria	k__norank_d__Bacteria	p__Actinobacteriota	c__Thermoleophilia	o__Solirubrobacterales	f__Solirubrobacteraceae	g__Conexibacter	s__unclassified_g__Conexibacter	OTU2561
d__Bacteria	k__norank_d__Bacteria	p__Bdellovibrionota	c__Bdellovibrionia	o__Bdellovibrionales	f__Bdellovibrionaceae	g__Bdellovibrio	s__uncultured_bacterium_g__Bdellovibrio	OTU137
d__Bacteria	k__norank_d__Bacteria	p__Firmicutes	c__Bacilli	o__Paenibacillales	f__Paenibacillaceae	g__Paenibacillus	s__unclassified_g__Paenibacillus	OTU2166
d__Bacteria	k__norank_d__Bacteria	p__Proteobacteria	c__Alphaproteobacteria	o__Rhizobiales	f__Xanthobacteraceae	g__Rhodoplanes	s__unclassified_g__Rhodoplanes	OTU2327
d__Bacteria	k__norank_d__Bacteria	p__Patescibacteria	c__Saccharimonadia	o__Saccharimonadales	f__norank_o__Saccharimonadales	g__norank_f__norank_o__Saccharimonadales	s__uncultured_bacterium_g__norank_f__norank_o__Saccharimonadales	OTU2429
d__Bacteria	k__norank_d__Bacteria	p__WPS-2	c__norank_p__WPS-2	o__norank_c__norank_p__WPS-2	f__norank_o__norank_c__norank_p__WPS-2	g__norank_f__norank_o__norank_c__norank_p__WPS-2	s__unclassified_g__norank_f__norank_o__norank_c__norank_p__WPS-2	OTU1970
d__Bacteria	k__norank_d__Bacteria	p__Chloroflexi	c__Chloroflexia	o__Elev-1554	f__norank_o__Elev-1554	g__norank_f__norank_o__Elev-1554	s__uncultured_bacterium_g__norank_f__norank_o__Elev-1554	OTU1010
d__Bacteria	k__norank_d__Bacteria	p__Myxococcota	c__Myxococcia	o__Myxococcales	f__Myxococcaceae	g__P3OB-42	s__unclassified_g__P3OB-42	OTU1515

d__Bacteria	k__norank_ d__Bacteria	p__Bdellovibrionota	c__Bdellovibrionia	o__Bdellovibrionales	f__Bdellovibrionaceae	g__Bdellovibrio	s__uncultured_bacterium_g__Bdellovibrio	OTU198
d__Bacteria	k__norank_ d__Bacteria	p__Chloroflexi	c__Ktedonobacteria	o__Ktedonobacterales	f__Ktedonobacteraceae	g__HSB_OF53-F07	s__unclassified_g__HSB_OF53-F07	OTU1700
d__Bacteria	k__norank_ d__Bacteria	p__Myxococcota	c__Polyangia	o__Polyangiales	f__BIrii41	g__norank_f__BIrii41	s__uncultured_bacterium_g__norank_f__BIrii41	OTU1393
d__Bacteria	k__norank_ d__Bacteria	p__Bdellovibrionota	c__Oligoflexia	o__0319-6G20	f__norank_o__0319-6G20	g__norank_f__norank_o__0319-6G20	s__marine_metagenome_g__norank_f__norank_o__0319-6G20	OTU875
d__Bacteria	k__norank_ d__Bacteria	p__Bdellovibrionota	c__Bdellovibrionia	o__Bdellovibrionales	f__Bdellovibrionaceae	g__Bdellovibrio	s__uncultured_bacterium_g__Bdellovibrio	OTU2744
d__Bacteria	k__norank_ d__Bacteria	p__Chloroflexi	c__Chloroflexia	o__Chloroflexales	f__Herpetosiphonaceae	g__Herpetosiphon	s__uncultured_Chloroflexi_bacterium_g__Herpetosiphon	OTU2231
d__Bacteria	k__norank_ d__Bacteria	p__Acidobacteriota	c__Blastocatellia	o__11-24	f__norank_o__11-24	g__norank_f__norank_o__11-24	s__unclassified_g__norank_f__norank_o__11-24	OTU1252
d__Bacteria	k__norank_ d__Bacteria	p__Actinobacteriota	c__Acidimicrobiia	o__unclassified_c__Acidimi crobiia	f__unclassified_c__Acidimicrobiia	g__unclassified_c__Acidimicrobiia	s__unclassified_c__Acidimicrobiia	OTU479
d__Bacteria	k__norank_ d__Bacteria	p__Myxococcota	c__Polyangia	o__Polyangiales	f__BIrii41	g__norank_f__BIrii41	s__uncultured_delta_proteobacterium_g__norank_f__BIrii41	OTU2087
d__Bacteria	k__norank_ d__Bacteria	p__Proteobacteria	c__Gammaproteobacteria	o__Xanthomonadales	f__Rhodanobacteraceae	g__Rhodanobacter	s__uncultured_Xanthomonadaceae_bacterium_g__Rhodanobacter	OTU2028
d__Bacteria	k__norank_ d__Bacteria	p__Bacteroidota	c__Bacteroidia	o__Chitinophagales	f__Chitinophagaceae	g__Flavitalea	s__uncultured_bacterium_g__Flavitalea	OTU2285
d__Bacteria	k__norank_ d__Bacteria	p__Gemmatimonadota	c__Gemmatimonadetes	o__Gemmatimonadales	f__Gemmatimonadaceae	g__norank_f__Gemmatimonadaceae	s__uncultured_bacterium_contig00007	OTU582
d__Bacteria	k__norank_ d__Bacteria	p__Chloroflexi	c__Ktedonobacteria	o__C0119	f__norank_o__C0119	g__norank_f__norank_o__C0119	s__uncultured_bacterium_g__norank_f__norank_o__C0119	OTU885
d__Bacteria	k__norank_ d__Bacteria	p__Patescibacteria	c__Parcubacteria	o__norank_c__Parcubacteria	f__norank_o__norank_c__Parcubacteria	g__norank_f__norank_o__norank_c__ Parcubacteria	s__uncultured_bacterium_g__norank_f__norank_o__norank_c__Parcubacteria	OTU1710
d__Bacteria	k__norank_ d__Bacteria	p__Chloroflexi	c__Ktedonobacteria	o__C0119	f__norank_o__C0119	g__norank_f__norank_o__C0119	s__unclassified_g__norank_f__norank_o__C0119	OTU1505
d__Bacteria	k__norank_ d__Bacteria	p__Firmicutes	c__Clostridia	o__Peptostreptococcales-Tis sierellales	f__Peptostreptococcaceae	g__Romboutsia	s__Romboutsia_ilealis	OTU2384

d__Bacteria	k__norank_d__Bacteria	p__Proteobacteria	c__Gammaproteobacteria	o__Gammaproteobacteria_Incertae_Sedis	f__unclassified_o__Gammaproteobacteria_Incertae_Sedis	g__Candidatus_Ovatusbacter	s__uncultured_proteobacterium_g__Candidatus_Ovatusbacter	OTU805
d__Bacteria	k__norank_d__Bacteria	p__Proteobacteria	c__Alphaproteobacteria	o__Rhodobacterales	f__Rhodobacteraceae	g__Yoonia-Loktanella	s__Loktanella_fryxellensis	OTU2353
d__Bacteria	k__norank_d__Bacteria	p__Acidobacteriota	c__Blastocatellia	o__DS-100	f__norank_o__DS-100	g__norank_f__norank_o__DS-100	s__uncultured_Acidobacteria_bacterium_g__norank_f__norank_o__DS-100	OTU2151
d__Bacteria	k__norank_d__Bacteria	p__Acidobacteriota	c__Holophagae	o__Subgroup_7	f__norank_o__Subgroup_7	g__norank_f__norank_o__Subgroup_7	s__unclassified_g__norank_f__norank_o__Subgroup_7	OTU820
d__Bacteria	k__norank_d__Bacteria	p__Myxococcota	c__Polyangia	o__mle1-27	f__norank_o__mle1-27	g__norank_f__norank_o__mle1-27	s__metagenome_g__norank_f__norank_o__mle1-27	OTU874
d__Bacteria	k__norank_d__Bacteria	p__Bacteroidota	c__Bacteroidia	o__Cytophagales	f__Hymenobacteraceae	g__Hymenobacter	s__unclassified_g__Hymenobacter	OTU615
d__Bacteria	k__norank_d__Bacteria	p__Patescibacteria	c__Saccharimonadia	o__Saccharimonadales	f__norank_o__Saccharimonadales	g__norank_f__norank_o__Saccharimonadales	s__unclassified_g__norank_f__norank_o__Saccharimonadales	OTU2541
d__Bacteria	k__norank_d__Bacteria	p__Myxococcota	c__Polyangia	o__Polyangiales	f__Polyangiaceae	g__Pajaroellobacter	s__unclassified_g__Pajaroellobacter	OTU2536
d__Bacteria	k__norank_d__Bacteria	p__Planctomycetota	c__Phycisphaerae	o__Phycisphaerales	f__Phycisphaeraceae	g__SM1A02	s__metagenome_g__SM1A02	OTU545
d__Bacteria	k__norank_d__Bacteria	p__Cyanobacteria	c__Cyanobacteriia	o__Chloroplast	f__norank_o__Chloroplast	g__norank_f__norank_o__Chloroplast	s__uncultured_Chlorella	OTU2892
d__Bacteria	k__norank_d__Bacteria	p__Proteobacteria	c__Alphaproteobacteria	o__Rhizobiales	f__Devosiaceae	g__unclassified_f__Devosiaceae	s__unclassified_f__Devosiaceae	OTU2167
d__Bacteria	k__norank_d__Bacteria	p__Patescibacteria	c__Saccharimonadia	o__Saccharimonadales	f__norank_o__Saccharimonadales	g__norank_f__norank_o__Saccharimonadales	s__unclassified_g__norank_f__norank_o__Saccharimonadales	OTU288
d__Bacteria	k__norank_d__Bacteria	p__Proteobacteria	c__Alphaproteobacteria	o__Micavibrionales	f__Micavibrionaceae	g__norank_f__Micavibrionaceae	s__uncultured_Alphaproteobacteria_bacterium_g__norank_f__Micavibrionaceae	OTU708
d__Bacteria	k__norank_d__Bacteria	p__Firmicutes	c__Bacilli	o__Lactobacillales	f__Vagococcaceae	g__Vagococcus	s__uncultured_bacterium_g__Vagococcus	OTU2940
d__Bacteria	k__norank_d__Bacteria	p__Proteobacteria	c__Gammaproteobacteria	o__Gammaproteobacteria_Incertae_Sedis	f__unclassified_o__Gammaproteobacteria_Incertae_Sedis	g__Candidatus_Ovatusbacter	s__uncultured_bacterium_g__Candidatus_Ovatusbacter	OTU1527
d__Bacteria	k__norank_d__Bacteria	p__Bacteroidota	c__Bacteroidia	o__Sphingobacteriales	f__Sphingobacteriaceae	g__Mucilaginibacter	s__uncultured_bacterium_g__Mucilaginibacter	OTU1717

d__Bacteria	k__norank_d__Bacteria	p__Verrucomicrobiota	c__Verrucomicrobiae	o__Verrucomicrobiales	f__Verrucomicrobiaceae	g__Roseimicrobium	s__metagenome_g__Roseimicrobium	OTU2572
d__Bacteria	k__norank_d__Bacteria	p__Armatimonadota	c__norank_p__Armatimonadota	o__norank_c__norank_p__Armatimonadota	f__norank_o__norank_c__norank_p__Armatimonadota	g__norank_f__norank_o__norank_c__norank_p__Armatimonadota	s__uncultured_bacterium_g__norank_f__norank_o__norank_c__norank_p__Armatimonadota	OTU456
d__Bacteria	k__norank_d__Bacteria	p__Bacteroidota	c__Kapabacteria	o__Kapabacteriales	f__norank_o__Kapabacteriales	g__norank_f__norank_o__Kapabacteriales	s__uncultured_Chlorobiales_bacterium_g__norank	OTU1296
d__Bacteria	k__norank_d__Bacteria	p__Cyanobacteria	c__Cyanobacteriia	o__Chloroplast	f__norank_o__Chloroplast	g__norank_f__norank_o__Chloroplast	s__unclassified_g__norank_f__norank_o__Chloroplast	OTU1092
d__Bacteria	k__norank_d__Bacteria	p__Bdellovibrionota	c__Oligoflexia	o__0319-6G20	f__norank_o__0319-6G20	g__norank_f__norank_o__0319-6G20	s__unclassified_g__norank_f__norank_o__0319-6G20	OTU850
d__Bacteria	k__norank_d__Bacteria	p__Chloroflexi	c__Ktedonobacteria	o__Ktedonobacterales	f__Ktedonobacteraceae	g__JG30a-KF-32	s__uncultured_Chloroflexi_bacterium_g__JG30a-KF-32	OTU2040
d__Bacteria	k__norank_d__Bacteria	p__Gemmatimonadota	c__Gemmatimonadetes	o__Gemmatimonadales	f__Gemmatimonadaceae	g__Gemmatimonas	s__unclassified_g__Gemmatimonas	OTU1290
d__Bacteria	k__norank_d__Bacteria	p__Proteobacteria	c__Gammaproteobacteria	o__Burkholderiales	f__Burkholderiaceae	g__Burkholderia-Caballeronia-Paraburkholderia	s__unclassified_g__Burkholderia-Caballeronia-Paraburkholderia	OTU2564
d__Bacteria	k__norank_d__Bacteria	p__Actinobacteriota	c__Thermoleophilia	o__Gaiellales	f__Gaiellaceae	g__Gaiella	s__metagenome_g__Gaiella	OTU633
d__Bacteria	k__norank_d__Bacteria	p__Chloroflexi	c__Ktedonobacteria	o__B10-SB3A	f__norank_o__B10-SB3A	g__norank_f__norank_o__B10-SB3A	s__uncultured_bacterium_g__norank_f__norank_o__B10-SB3A	OTU1798
d__Bacteria	k__norank_d__Bacteria	p__Elusimicrobiota	c__Lineage_IIa	o__norank_c__Lineage_IIa	f__norank_o__norank_c__Lineage_IIa	g__norank_f__norank_o__norank_c__Lineage_IIa	s__unclassified_g__norank_f__norank_o__norank_c__Lineage_IIa	OTU1507
d__Bacteria	k__norank_d__Bacteria	p__Bdellovibrionota	c__Oligoflexia	o__0319-6G20	f__norank_o__0319-6G20	g__norank_f__norank_o__0319-6G20	s__metagenome_g__norank_f__norank_o__0319-6G20	OTU63
d__Bacteria	k__norank_d__Bacteria	p__Verrucomicrobiota	c__Chlamydiae	o__Chlamydiales	f__Parachlamydiaceae	g__unclassified_f__Parachlamydiaceae	s__unclassified_f__Parachlamydiaceae	OTU951
d__Bacteria	k__norank_d__Bacteria	p__unclassified_k__norank_d__Bacteria	c__unclassified_k__norank_d__Bacteria	o__unclassified_k__norank_d__Bacteria	f__unclassified_k__norank_d__Bacteria	g__unclassified_k__norank_d__Bacteria	s__unclassified_k__norank_d__Bacteria	OTU1057
d__Bacteria	k__norank_d__Bacteria	p__Chloroflexi	c__Chloroflexia	o__Kallotenuales	f__AKIW781	g__norank_f__AKIW781	s__uncultured_bacterium_g__norank_f__AKIW781	OTU2005
d__Bacteria	k__norank_d__Bacteria	p__Bacteroidota	c__Bacteroidia	o__Chitinophagales	f__Chitinophagaceae	g__Flavisolibacter	s__uncultured_bacterium_g__Flavisolibacter	OTU305

d__Bacteria	k__norank_ d__Bacteria	p__Planctomycetota	c__Planctomycetes	o__Isosphaerales	f__Isosphaeraceae	g__norank_f__Isosphaeraceae	s__unclassified_g__norank_f__Isosphaeraceae	OTU1711
d__Bacteria	k__norank_ d__Bacteria	p__Bdellovibrionota	c__Oligoflexia	o__0319-6G20	f__norank_o__0319-6G20	g__norank_f__norank_o__0319-6G20	s__unclassified_g__norank_f__norank_o__0319-6G20	OTU179
d__Bacteria	k__norank_ d__Bacteria	p__Bdellovibrionota	c__Oligoflexia	o__0319-6G20	f__norank_o__0319-6G20	g__norank_f__norank_o__0319-6G20	s__metagenome_g__norank_f__norank_o__0319-6G20	OTU662
d__Bacteria	k__norank_ d__Bacteria	p__Bdellovibrionota	c__Oligoflexia	o__0319-6G20	f__norank_o__0319-6G20	g__norank_f__norank_o__0319-6G20	s__unclassified_g__norank_f__norank_o__0319-6G20	OTU2515
d__Bacteria	k__norank_ d__Bacteria	p__Chloroflexi	c__TK10	o__norank_c__TK10	f__norank_o__norank_c__TK10	g__norank_f__norank_o__norank_c__ TK10	s__unclassified_g__norank_f__norank_o__norank_c__TK10	OTU1344
d__Bacteria	k__norank_ d__Bacteria	p__Verrucomicrobiota	c__Verrucomicrobiae	o__Chthoniobacterales	f__Chthoniobacteraceae	g__Chthoniobacter	s__uncultured_Verrucomicrobia_bacterium_g__Chthoniobacter	OTU2749
d__Bacteria	k__norank_ d__Bacteria	p__Bacteroidota	c__Kapabacteria	o__Kapabacteriales	f__norank_o__Kapabacteriales	g__norank_f__norank_o__Kapabacteri ales	s__uncultured_bacterium_KF-JG30-B11	OTU217
d__Bacteria	k__norank_ d__Bacteria	p__Myxococcota	c__Polyangia	o__mle1-27	f__norank_o__mle1-27	g__norank_f__norank_o__mle1-27	s__unclassified_g__norank_f__norank_o__mle1-27	OTU1079
d__Bacteria	k__norank_ d__Bacteria	p__Chloroflexi	c__Ktedonobacteria	o__B12-WMSP1	f__norank_o__B12-WMSP1	g__norank_f__norank_o__B12-WMSP 1	s__uncultured_bacterium_g__norank_f__norank_o__B12-WMSP1	OTU1054
d__Bacteria	k__norank_ d__Bacteria	p__Proteobacteria	c__Gammaproteobacteria	o__Xanthomonadales	f__Rhodanobacteraceae	g__Pseudofulvimonas	s__unclassified_g__Pseudofulvimonas	OTU657
d__Bacteria	k__norank_ d__Bacteria	p__Proteobacteria	c__Gammaproteobacteria	o__Gammaproteobacteria_I ncertae_Sedis	f__unclassified_o__Gammaproteobacteri a_Incertae_Sedis	g__Candidatus_Ovatusbacter	s__uncultured_bacterium_g__Candidatus_Ovatusbacter	OTU727
d__Bacteria	k__norank_ d__Bacteria	p__Proteobacteria	c__Gammaproteobacteria	o__CCD24	f__norank_o__CCD24	g__norank_f__norank_o__CCD24	s__metagenome_g__norank_f__norank_o__CCD24	OTU2223
d__Bacteria	k__norank_ d__Bacteria	p__Firmicutes	c__Clostridia	o__Peptostreptococcales-Tis sierellales	f__Peptostreptococcaceae	g__Paeniclostridium	s__unclassified_g__Paeniclostridium	OTU139
d__Bacteria	k__norank_ d__Bacteria	p__Patescibacteria	c__Saccharimonadia	o__Saccharimonadales	f__norank_o__Saccharimonadales	g__norank_f__norank_o__Saccharimo nadales	s__uncultured_Candidatus_Saccharibacteria_bacterium_g__norank_f__norank	OTU2979
d__Bacteria	k__norank_ d__Bacteria	p__Actinobacteriota	c__Actinobacteria	o__Micrococcales	f__Intrasporangiaceae	g__Arsenicicoccus	s__Arsenicicoccus_sp._oral_taxon_190	OTU509
d__Bacteria	k__norank_ d__Bacteria	p__Proteobacteria	c__Alphaproteobacteria	o__Rhizobiales	f__Rhizobiales_Incertae_Sedis	g__Nordella	s__uncultured_bacterium_g__Nordella	OTU392

d__Bacteria	k__norank_d__Bacteria	p__Proteobacteria	c__Alphaproteobacteria	o__Acetobacterales	f__Acetobacteraceae	g__Roseomonas	s__unclassified_g__Roseomonas	OTU2922
d__Bacteria	k__norank_d__Bacteria	p__Patescibacteria	c__Saccharimonadia	o__Saccharimonadales	f__norank_o__Saccharimonadales	g__norank_f__norank_o__Saccharimonadales	s__uncultured_candidate_division_WS5_bacterium	OTU2239
d__Bacteria	k__norank_d__Bacteria	p__Cyanobacteria	c__Sericytochromatia	o__norank_c__Sericytochromatia	f__norank_o__norank_c__Sericytochromatia	g__norank_f__norank_o__norank_c__Sericytochromatia	s__uncultured_bacterium_g__norank_f__norank_o__norank_c__Sericytochromatia	OTU1687
d__Bacteria	k__norank_d__Bacteria	p__Chloroflexi	c__Ktedonobacteria	o__C0119	f__norank_o__C0119	g__norank_f__norank_o__C0119	s__uncultured_bacterium_g__norank_f__norank_o__C0119	OTU1773
d__Bacteria	k__norank_d__Bacteria	p__Myxococcota	c__Myxococcia	o__Myxococcales	f__Myxococcaceae	g__P3OB-42	s__unclassified_g__P3OB-42	OTU1053
d__Bacteria	k__norank_d__Bacteria	p__Actinobacteriota	c__Thermoleophilia	o__Solirubrobacterales	f__67-14	g__norank_f__67-14	s__uncultured_soil_bacterium_g__norank_f__67-14	OTU1295
d__Bacteria	k__norank_d__Bacteria	p__Actinobacteriota	c__Acidimicrobiia	o__Microtrichales	f__Ilumatobacteraceae	g__norank_f__Ilumatobacteraceae	s__uncultured_bacterium_g__norank_f__Ilumatobacteraceae	OTU899
d__Bacteria	k__norank_d__Bacteria	p__Proteobacteria	c__Alphaproteobacteria	o__Acetobacterales	f__Acetobacteraceae	g__norank_f__Acetobacteraceae	s__unclassified_g__norank_f__Acetobacteraceae	OTU21
d__Bacteria	k__norank_d__Bacteria	p__Actinobacteriota	c__Actinobacteria	o__Streptomycetales	f__Streptomycetaceae	g__E1B-B3-114	s__uncultured_actinobacterium_g__E1B-B3-114	OTU2867
d__Bacteria	k__norank_d__Bacteria	p__Gemmatimonadota	c__S0134_terrestrial_group	o__norank_c__S0134_terrestrial_group	f__norank_o__norank_c__S0134_terrestrial_group	g__norank_f__norank_o__norank_c__S0134_terrestrial_group	s__unclassified_g__norank_f__norank_o__norank_c__S0134_terrestrial_group	OTU618
d__Bacteria	k__norank_d__Bacteria	p__Gemmatimonadota	c__S0134_terrestrial_group	o__norank_c__S0134_terrestrial_group	f__norank_o__norank_c__S0134_terrestrial_group	g__norank_f__norank_o__norank_c__S0134_terrestrial_group	s__unclassified_g__norank_f__norank_o__norank_c__S0134_terrestrial_group	OTU616
d__Bacteria	k__norank_d__Bacteria	p__Proteobacteria	c__Gammaproteobacteria	o__Burkholderiales	f__Comamonadaceae	g__unclassified_f__Comamonadaceae	s__unclassified_f__Comamonadaceae	OTU101
d__Bacteria	k__norank_d__Bacteria	p__Actinobacteriota	c__Acidimicrobiia	o__IMCC26256	f__norank_o__IMCC26256	g__norank_f__norank_o__IMCC26256	s__uncultured_actinobacterium_g__norank_f__norank_o__IMCC26256	OTU1115
d__Bacteria	k__norank_d__Bacteria	p__Planctomycetota	c__Planctomycetes	o__Gemmatales	f__Gemmataceae	g__norank_f__Gemmataceae	s__uncultured_planctomycete_g__norank_f__Gemmataceae	OTU1088
d__Bacteria	k__norank_d__Bacteria	p__Actinobacteriota	c__Acidimicrobiia	o__norank_c__Acidimicrobiia	f__norank_o__norank_c__Acidimicrobiia	g__norank_f__norank_o__norank_c__Acidimicrobiia	s__uncultured_bacterium_g__norank_f__norank_o__norank_c__Acidimicrobiia	OTU1347
d__Bacteria	k__norank_d__Bacteria	p__Chloroflexi	c__Chloroflexia	o__Thermomicrobiales	f__JG30-KF-CM45	g__norank_f__JG30-KF-CM45	s__uncultured_bacterium_g__norank_f__JG30-KF-CM45	OTU568

d__Bacteria	k__norank_ d__Bacteria	p__Actinobacteriota	c__Actinobacteria	o__Propionibacteriales	f__Propionibacteriaceae	g__unclassified_f__Propionibacteriace ae	s__unclassified_f__Propionibacteriaceae	OTU2488
d__Bacteria	k__norank_ d__Bacteria	p__Proteobacteria	c__Alphaproteobacteria	o__Rhizobiales	f__Beijerinckiaceae	g__Methylobacterium-Methylorubrum	s__unclassified_g__Methylobacterium-Methylorubrum	OTU1922
d__Bacteria	k__norank_ d__Bacteria	p__Deinococcota	c__Deinococci	o__Deinococcales	f__Trueperaceae	g__Truepera	s__uncultured_bacterium_g__Truepera	OTU2629
d__Bacteria	k__norank_ d__Bacteria	p__Planctomycetota	c__Planctomycetes	o__Isosphaerales	f__Isosphaeraceae	g__Singulisphaera	s__uncultured_bacterium_g__Singulisphaera	OTU823
d__Bacteria	k__norank_ d__Bacteria	p__Proteobacteria	c__Gammaproteobacteria	o__Acidiferrobacterales	f__Acidiferrobacteraceae	g__norank_f__Acidiferrobacteraceae	s__uncultured_bacterium_g__norank_f__Acidiferrobacteraceae	OTU85
d__Bacteria	k__norank_ d__Bacteria	p__Bdellovibrionota	c__Oligoflexia	o__Oligoflexales	f__norank_o__Oligoflexales	g__norank_f__norank_o__Oligoflexale s	s__unclassified_g__norank_f__norank_o__Oligoflexales	OTU484
d__Bacteria	k__norank_ d__Bacteria	p__Deinococcota	c__Deinococci	o__Deinococcales	f__Trueperaceae	g__Truepera	s__uncultured_Deinococcales_bacterium	OTU2871
d__Bacteria	k__norank_ d__Bacteria	p__Chloroflexi	c__Chloroflexia	o__Chloroflexales	f__Roseiflexaceae	g__norank_f__Roseiflexaceae	s__uncultured_bacterium_g__norank_f__Roseiflexaceae	OTU1695
d__Bacteria	k__norank_ d__Bacteria	p__Armatimonadota	c__Armatimonadia	o__Armatimonadales	f__Armatimonadaceae	g__Armatimonas	s__uncultured_bacterium_g__Armatimonas	OTU1577
d__Bacteria	k__norank_ d__Bacteria	p__Bacteroidota	c__Bacteroidia	o__Cytophagales	f__Spirosomaceae	g__Rudanella	s__uncultured_bacterium_g__Rudanella	OTU213
d__Bacteria	k__norank_ d__Bacteria	p__Deinococcota	c__Deinococci	o__Deinococcales	f__Deinococcaceae	g__Deinococcus	s__uncultured_bacterium_g__Deinococcus	OTU1554
d__Bacteria	k__norank_ d__Bacteria	p__Bacteroidota	c__Bacteroidia	o__Chitinophagales	f__Chitinophagaceae	g__Cnuella	s__uncultured_bacterium_g__Cnuella	OTU263
d__Bacteria	k__norank_ d__Bacteria	p__Chloroflexi	c__Chloroflexia	o__Thermomicrobiales	f__unclassified_o__Thermomicrobiales	g__unclassified_o__Thermomicrobiale s	s__unclassified_o__Thermomicrobiales	OTU802
d__Bacteria	k__norank_ d__Bacteria	p__Myxococcota	c__Myxococcia	o__Myxococcales	f__Myxococcaceae	g__P3OB-42	s__uncultured_bacterium_g__P3OB-42	OTU411
d__Bacteria	k__norank_ d__Bacteria	p__Patescibacteria	c__Saccharimonadia	o__Saccharimonadales	f__norank_o__Saccharimonadales	g__norank_f__norank_o__Saccharimo nadales	s__unclassified_g__norank_f__norank_o__Saccharimonadales	OTU2945
d__Bacteria	k__norank_ d__Bacteria	p__Bdellovibrionota	c__Oligoflexia	o__Oligoflexales	f__norank_o__Oligoflexales	g__Oligoflexus	s__uncultured_bacterium_g__Oligoflexus	OTU366

d__Bacteria	k__norank_d__Bacteria	p__Patescibacteria	c__Saccharimonadia	o__Saccharimonadales	f__norank_o__Saccharimonadales	g__norank_f__norank_o__Saccharimonadales	s__unclassified_g__norank_f__norank_o__Saccharimonadales	OTU2341
d__Bacteria	k__norank_d__Bacteria	p__Bdellovibrionota	c__Oligoflexia	o__0319-6G20	f__norank_o__0319-6G20	g__norank_f__norank_o__0319-6G20	s__unclassified_g__norank_f__norank_o__0319-6G20	OTU1813
d__Bacteria	k__norank_d__Bacteria	p__Chloroflexi	c__KD4-96	o__norank_c__KD4-96	f__norank_o__norank_c__KD4-96	g__norank_f__norank_o__norank_c__KD4-96	s__uncultured_bacterium_g__norank_f__norank_o__norank_c__KD4-96	OTU1064
d__Bacteria	k__norank_d__Bacteria	p__Actinobacteriota	c__Acidimicrobiia	o__Microtrichales	f__Ilumatobacteraceae	g__norank_f__Ilumatobacteraceae	s__uncultured_bacterium_g__norank_f__Ilumatobacteraceae	OTU2010
d__Bacteria	k__norank_d__Bacteria	p__Proteobacteria	c__Gammaproteobacteria	o__Steroidobacterales	f__Steroidobacteraceae	g__norank_f__Steroidobacteraceae	s__metagenome_g__norank_f__Steroidobacteraceae	OTU2492
d__Bacteria	k__norank_d__Bacteria	p__Planctomycetota	c__Planctomycetes	o__Isosphaerales	f__Isosphaeraceae	g__norank_f__Isosphaeraceae	s__uncultured_bacterium_g__norank_f__Isosphaeraceae	OTU1734
d__Bacteria	k__norank_d__Bacteria	p__Chloroflexi	c__Anaerolineae	o__SBR1031	f__A4b	g__OLB13	s__uncultured_bacterium_g__OLB13	OTU2106
d__Bacteria	k__norank_d__Bacteria	p__Bacteroidota	c__Bacteroidia	o__Cytophagales	f__Spirosomaceae	g__Dyadobacter	s__unclassified_g__Dyadobacter	OTU2305
d__Bacteria	k__norank_d__Bacteria	p__Proteobacteria	c__Gammaproteobacteria	o__Xanthomonadales	f__Rhodanobacteraceae	g__Ahniella	s__unclassified_g__Ahniella	OTU1337
d__Bacteria	k__norank_d__Bacteria	p__Dependentiae	c__Babeliae	o__Babeliales	f__unclassified_o__Babeliales	g__unclassified_o__Babeliales	s__unclassified_o__Babeliales	OTU1858
d__Bacteria	k__norank_d__Bacteria	p__Bacteroidota	c__Bacteroidia	o__Sphingobacteriales	f__env.OPS_17	g__norank_f__env.OPS_17	s__uncultured_Bacteroidetes_bacterium_g__norank_f__env.OPS_17	OTU790
d__Bacteria	k__norank_d__Bacteria	p__Myxococcota	c__Polyangia	o__Blfdi19	f__norank_o__Blfdi19	g__norank_f__norank_o__Blfdi19	s__metagenome_g__norank_f__norank_o__Blfdi19	OTU244
d__Bacteria	k__norank_d__Bacteria	p__Bacteroidota	c__Bacteroidia	o__Chitinophagales	f__Chitinophagaceae	g__Ferruginibacter	s__uncultured_Chitinophagaceae_bacterium_g__Ferruginibacter	OTU1716
d__Bacteria	k__norank_d__Bacteria	p__Proteobacteria	c__Alphaproteobacteria	o__Rhizobiales	f__Beijerinckiaceae	g__FFCH5858	s__uncultured_bacterium_g__FFCH5858	OTU2505
d__Bacteria	k__norank_d__Bacteria	p__Proteobacteria	c__Gammaproteobacteria	o__Burkholderiales	f__Oxalobacteraceae	g__unclassified_f__Oxalobacteraceae	s__unclassified_f__Oxalobacteraceae	OTU1670
d__Bacteria	k__norank_d__Bacteria	p__Bdellovibrionota	c__Oligoflexia	o__Oligoflexales	f__norank_o__Oligoflexales	g__Oligoflexus	s__unclassified_g__Oligoflexus	OTU2195

d__Bacteria	k__norank_ d__Bacteria	p__Planctomycetota	c__Planctomycetes	o__Gemmatales	f__Gemmataceae	g__norank_f__Gemmataceae	s__unclassified_g__norank_f__Gemmataceae	OTU754
d__Bacteria	k__norank_ d__Bacteria	p__Planctomycetota	c__Planctomycetes	o__Gemmatales	f__Gemmataceae	g__norank_f__Gemmataceae	s__uncultured_bacterium_g__norank_f__Gemmataceae	OTU18
d__Bacteria	k__norank_ d__Bacteria	p__Chloroflexi	c__Ktedonobacteria	o__C0119	f__norank_o__C0119	g__norank_f__norank_o__C0119	s__unclassified_g__norank_f__norank_o__C0119	OTU1350
d__Bacteria	k__norank_ d__Bacteria	p__Actinobacteriota	c__Actinobacteria	o__Frankiales	f__Frankiaceae	g__Jatrophihabitans	s__Jatrophihabitans_sp.	OTU944
d__Bacteria	k__norank_ d__Bacteria	p__Acidobacteriota	c__Holophagae	o__Subgroup_7	f__norank_o__Subgroup_7	g__norank_f__norank_o__Subgroup_7	s__uncultured_Acidobacteria_bacterium_g__norank_f__norank_o__Subgroup_7	OTU451
d__Bacteria	k__norank_ d__Bacteria	p__Planctomycetota	c__Planctomycetes	o__Isosphaerales	f__Isosphaeraceae	g__Singulisphaera	s__uncultured_bacterium_g__Singulisphaera	OTU2743
d__Bacteria	k__norank_ d__Bacteria	p__Bacteroidota	c__Bacteroidia	o__Sphingobacteriales	f__env.OPS_17	g__norank_f__env.OPS_17	s__uncultured_bacterium_g__norank_f__env.OPS_17	OTU241
d__Bacteria	k__norank_ d__Bacteria	p__Proteobacteria	c__Gammaproteobacteria	o__Burkholderiales	f__Oxalobacteraceae	g__Undibacterium	s__uncultured_beta_proteobacterium_g__Undibacterium	OTU29
d__Bacteria	k__norank_ d__Bacteria	p__Gemmatimonadota	c__Longimicrobia	o__Longimicrobiales	f__Longimicrobiaceae	g__Longimicrobium	s__uncultured_bacterium_g__Longimicrobium	OTU285
d__Bacteria	k__norank_ d__Bacteria	p__Proteobacteria	c__Gammaproteobacteria	o__JG36-TzT-191	f__norank_o__JG36-TzT-191	g__norank_f__norank_o__JG36-TzT-1 91	s__uncultured_bacterium_g__norank_f__norank_o__JG36-TzT-191	OTU1721
d__Bacteria	k__norank_ d__Bacteria	p__Myxococcota	c__Polyangia	o__Polyangiales	f__Phaselicystidaceae	g__Phaselicystis	s__unclassified_g__Phaselicystis	OTU376
d__Bacteria	k__norank_ d__Bacteria	p__Acidobacteriota	c__Blastocatellia	o__Pyrinomonadales	f__Pyrinomonadaceae	g__RB41	s__uncultured_Acidobacteria_bacterium_g__RB41	OTU687
d__Bacteria	k__norank_ d__Bacteria	p__Bacteroidota	c__Bacteroidia	o__Cytophagales	f__Spirosomaceae	g__Persicitalea	s__uncultured_bacterium_g__Persicitalea	OTU390
d__Bacteria	k__norank_ d__Bacteria	p__Actinobacteriota	c__Acidimicrobiia	o__Microtrichales	f__Ilumatobacteraceae	g__norank_f__Ilumatobacteraceae	s__uncultured_Acidimicrobiales_bacterium_g__norank_f__Ilumatobacteraceae	OTU1655
d__Bacteria	k__norank_ d__Bacteria	p__Proteobacteria	c__Gammaproteobacteria	o__Burkholderiales	f__Nitrosomonadaceae	g__mle1-7	s__uncultured_bacterium_g__mle1-7	OTU1541
d__Bacteria	k__norank_ d__Bacteria	p__Gemmatimonadota	c__Gemmatimonadetes	o__Gemmatimonadales	f__Gemmatimonadaceae	g__Roseisolibacter	s__uncultured_bacterium_g__Roseisolibacter	OTU2959

d__Bacteria	k__norank_ d__Bacteria	p__Chloroflexi	c__Chloroflexia	o__Thermomicrobiales	f__AKYG1722	g__norank_f__AKYG1722	s__unclassified_g__norank_f__AKYG1722	OTU2252
d__Bacteria	k__norank_ d__Bacteria	p__Bdellovibrionota	c__Oligoflexia	o__Oligoflexales	f__norank_o__Oligoflexales	g__norank_f__norank_o__Oligoflexale s	s__unclassified_g__norank_f__norank_o__Oligoflexales	OTU660
d__Bacteria	k__norank_ d__Bacteria	p__unclassified_k__no rank_d__Bacteria	c__unclassified_k__noran k_d__Bacteria	o__unclassified_k__norank_ d__Bacteria	f__unclassified_k__norank_d__Bacteria	g__unclassified_k__norank_d__Bacteri a	s__unclassified_k__norank_d__Bacteria	OTU1981
d__Bacteria	k__norank_ d__Bacteria	p__Proteobacteria	c__Gammaproteobacteria	o__Burkholderiales	f__SC-I-84	g__norank_f__SC-I-84	s__unclassified_g__norank_f__SC-I-84	OTU1635
d__Bacteria	k__norank_ d__Bacteria	p__Firmicutes	c__Bacilli	o__Lactobacillales	f__Lactobacillaceae	g__Lactobacillus	s__Lactobacillus_reuteri	OTU576
d__Bacteria	k__norank_ d__Bacteria	p__Proteobacteria	c__Gammaproteobacteria	o__Burkholderiales	f__unclassified_o__Burkholderiales	g__unclassified_o__Burkholderiales	s__unclassified_o__Burkholderiales	OTU2017
d__Bacteria	k__norank_ d__Bacteria	p__Proteobacteria	c__Alphaproteobacteria	o__Paracaedibacterales	f__Paracaedibacteraceae	g__Candidatus_Captivus	s__uncultured_bacterium_g__Candidatus_Captivus	OTU1692
d__Bacteria	k__norank_ d__Bacteria	p__Acidobacteriota	c__Acidobacteriae	o__Acidobacteriales	f__norank_o__Acidobacteriales	g__norank_f__norank_o__Acidobacter iales	s__unclassified_g__norank_f__norank_o__Acidobacteriales	OTU1200
d__Bacteria	k__norank_ d__Bacteria	p__Proteobacteria	c__Alphaproteobacteria	o__Acetobacterales	f__Acetobacteraceae	g__Rhodovarius	s__uncultured_bacterium_g__Rhodovarius	OTU2303
d__Bacteria	k__norank_ d__Bacteria	p__Proteobacteria	c__Gammaproteobacteria	o__Burkholderiales	f__SC-I-84	g__norank_f__SC-I-84	s__uncultured_bacterium_g__norank_f__SC-I-84	OTU2417
d__Bacteria	k__norank_ d__Bacteria	p__Proteobacteria	c__Gammaproteobacteria	o__Burkholderiales	f__Rhodocyclaceae	g__Dechloromonas	s__metagenome_g__Dechloromonas	OTU664
d__Bacteria	k__norank_ d__Bacteria	p__Proteobacteria	c__Gammaproteobacteria	o__Burkholderiales	f__Comamonadaceae	g__Ramlibacter	s__Ramlibacter_sp.	OTU402
d__Bacteria	k__norank_ d__Bacteria	p__Myxococcota	c__Myxococcia	o__Myxococcales	f__Myxococcaceae	g__P3OB-42	s__unclassified_g__P3OB-42	OTU3
d__Bacteria	k__norank_ d__Bacteria	p__Myxococcota	c__Polyangia	o__Polyangiales	f__Sandaracinaceae	g__norank_f__Sandaracinaceae	s__unclassified_g__norank_f__Sandaracinaceae	OTU2606
d__Bacteria	k__norank_ d__Bacteria	p__Myxococcota	c__Myxococcia	o__Myxococcales	f__Anaeromyxobacteraceae	g__Anaeromyxobacter	s__uncultured_bacterium_g__Anaeromyxobacter	OTU877
d__Bacteria	k__norank_ d__Bacteria	p__Patescibacteria	c__Saccharimonadia	o__Saccharimonadales	f__norank_o__Saccharimonadales	g__norank_f__norank_o__Saccharimo nadales	s__uncultured_bacterium_g__norank_f__norank_o__Saccharimonadales	OTU2410

d__Bacteria	k__norank_ d__Bacteria	p__Proteobacteria	c__Alphaproteobacteria	o__Acetobacterales	f__Acetobacteraceae	g__Acidiphilium	s__unclassified_g__Acidiphilium	OTU76
d__Bacteria	k__norank_ d__Bacteria	p__Gemmatimonadota	c__Gemmatimonadetes	o__Gemmatimonadales	f__Gemmatimonadaceae	g__unclassified_f__Gemmatimonadace ae	s__unclassified_f__Gemmatimonadaceae	OTU629
d__Bacteria	k__norank_ d__Bacteria	p__Myxococcota	c__Polyangia	o__mle1-27	f__norank_o__mle1-27	g__norank_f__norank_o__mle1-27	s__unclassified_g__norank_f__norank_o__mle1-27	OTU1918
d__Bacteria	k__norank_ d__Bacteria	p__Actinobacteriota	c__Thermoleophilia	o__Solirubrobacterales	f__Solirubrobacteraceae	g__Parviterribacter	s__uncultured_bacterium_g__Parviterribacter	OTU2468
d__Bacteria	k__norank_ d__Bacteria	p__Actinobacteriota	c__Actinobacteria	o__Frankiales	f__norank_o__Frankiales	g__norank_f__norank_o__Frankiales	s__unclassified_g__norank_f__norank_o__Frankiales	OTU2374
d__Bacteria	k__norank_ d__Bacteria	p__Actinobacteriota	c__Actinobacteria	o__Propionibacteriales	f__Propionibacteriaceae	g__Propioniciclava	s__uncultured_bacterium_g__Propioniciclava	OTU2181
d__Bacteria	k__norank_ d__Bacteria	p__Chloroflexi	c__KD4-96	o__norank_c__KD4-96	f__norank_o__norank_c__KD4-96	g__norank_f__norank_o__norank_c__ KD4-96	s__uncultured_bacterium_g__norank_f__norank_o__norank_c__KD4-96	OTU2654
d__Bacteria	k__norank_ d__Bacteria	p__Chloroflexi	c__Chloroflexia	o__Thermomicrobiales	f__JG30-KF-CM45	g__norank_f__JG30-KF-CM45	s__uncultured_bacterium_g__norank_f__JG30-KF-CM45	OTU1915
d__Bacteria	k__norank_ d__Bacteria	p__Bacteroidota	c__Bacteroidia	o__Chitinophagales	f__Chitinophagaceae	g__Aurantisolimonas	s__unclassified_g__Aurantisolimonas	OTU1278
d__Bacteria	k__norank_ d__Bacteria	p__Myxococcota	c__Polyangia	o__Polyangiales	f__Eel-36e1D6	g__norank_f__Eel-36e1D6	s__unclassified_g__norank_f__Eel-36e1D6	OTU700
d__Bacteria	k__norank_ d__Bacteria	p__Proteobacteria	c__Alphaproteobacteria	o__Acetobacterales	f__Acetobacteraceae	g__unclassified_f__Acetobacteraceae	s__unclassified_f__Acetobacteraceae	OTU886
d__Bacteria	k__norank_ d__Bacteria	p__Proteobacteria	c__Alphaproteobacteria	o__Rhizobiales	f__norank_o__Rhizobiales	g__norank_f__norank_o__Rhizobiales	s__uncultured_bacterium_g__norank_f__norank_o__Rhizobiales	OTU1808
d__Bacteria	k__norank_ d__Bacteria	p__unclassified_k__no rank_d__Bacteria	c__unclassified_k__noran k_d__Bacteria	o__unclassified_k__norank_ d__Bacteria	f__unclassified_k__norank_d__Bacteria	g__unclassified_k__norank_d__Bacteri a	s__unclassified_k__norank_d__Bacteria	OTU301
d__Bacteria	k__norank_ d__Bacteria	p__Proteobacteria	c__Gammaproteobacteria	o__Diplorickettsiales	f__Diplorickettsiaceae	g__Aquicella	s__unclassified_g__Aquicella	OTU2729
d__Bacteria	k__norank_ d__Bacteria	p__Deinococcota	c__Deinococci	o__Deinococcales	f__Deinococcaceae	g__Deinococcus	s__unclassified_g__Deinococcus	OTU1733
d__Bacteria	k__norank_ d__Bacteria	p__Deinococcota	c__Deinococci	o__Deinococcales	f__Deinococcaceae	g__Deinococcus	s__unclassified_g__Deinococcus	OTU1736

d__Bacteria	k__norank_ d__Bacteria	p__Armatimonadota	c__Chthonomonadetes	o__Chthonomonadales	f__Chthonomonadaceae	g__Chthonomonas	s__uncultured_bacterium_g__Chthonomonas	OTU1022
d__Bacteria	k__norank_ d__Bacteria	p__Myxococcota	c__Polyangia	o__Haliangiales	f__Haliangiaceae	g__Haliangium	s__uncultured_bacterium_g__Haliangium	OTU1214
d__Bacteria	k__norank_ d__Bacteria	p__Proteobacteria	c__Gammaproteobacteria	o__Legionellales	f__Legionellaceae	g__Legionella	s__unclassified_g__Legionella	OTU646
d__Bacteria	k__norank_ d__Bacteria	p__Verrucomicrobiota	c__Verrucomicrobiae	o__Opitutales	f__Opitutaceae	g__Lacunisphaera	s__uncultured_bacterium_g__Lacunisphaera	OTU773
d__Bacteria	k__norank_ d__Bacteria	p__Bacteroidota	c__Bacteroidia	o__Cytophagales	f__Spirosomaceae	g__Spirosoma	s__Spirosoma_spitsbergense_DSM_19989	OTU1696
d__Bacteria	k__norank_ d__Bacteria	p__Armatimonadota	c__Fimbriimonadia	o__Fimbriimonadales	f__Fimbriimonadaceae	g__norank_f__Fimbriimonadaceae	s__unclassified_g__norank_f__Fimbriimonadaceae	OTU2075
d__Bacteria	k__norank_ d__Bacteria	p__Actinobacteriota	c__Thermoleophilia	o__Solirubrobacterales	f__Solirubrobacteraceae	g__unclassified_f__Solirubrobacterace ae	s__unclassified_f__Solirubrobacteraceae	OTU2481
d__Bacteria	k__norank_ d__Bacteria	p__Actinobacteriota	c__Actinobacteria	o__PeM15	f__norank_o__PeM15	g__norank_f__norank_o__PeM15	s__metagenome_g__norank_f__norank_o__PeM15	OTU572
d__Bacteria	k__norank_ d__Bacteria	p__Chloroflexi	c__Chloroflexia	o__Chloroflexales	f__Roseiflexaceae	g__norank_f__Roseiflexaceae	s__uncultured_soil_bacterium_g__norank_f__Roseiflexaceae	OTU631
d__Bacteria	k__norank_ d__Bacteria	p__Myxococcota	c__Polyangia	o__Haliangiales	f__Haliangiaceae	g__Haliangium	s__unclassified_g__Haliangium	OTU1638
d__Bacteria	k__norank_ d__Bacteria	p__Bdellovibrionota	c__Oligoflexia	o__0319-6G20	f__norank_o__0319-6G20	g__norank_f__norank_o__0319-6G20	s__unclassified_g__norank_f__norank_o__0319-6G20	OTU462
d__Bacteria	k__norank_ d__Bacteria	p__Actinobacteriota	c__Actinobacteria	o__Corynebacteriales	f__Mycobacteriaceae	g__Mycobacterium	s__unclassified_g__Mycobacterium	OTU2592
d__Bacteria	k__norank_ d__Bacteria	p__Cyanobacteria	c__Sericytochromatia	o__norank_c__Sericytochro matia	f__norank_o__norank_c__Sericytochrom atia	g__norank_f__norank_o__norank_c__ Sericytochromatia	s__unclassified_g__norank_f__norank_o__norank_c__Sericytochromatia	OTU612
d__Bacteria	k__norank_ d__Bacteria	p__Actinobacteriota	c__Acidimicrobiia	o__IMCC26256	f__norank_o__IMCC26256	g__norank_f__norank_o__IMCC26256	s__unclassified_g__norank_f__norank_o__IMCC26256	OTU2105
d__Bacteria	k__norank_ d__Bacteria	p__Actinobacteriota	c__Actinobacteria	o__Frankiales	f__Acidothermaceae	g__Acidothermus	s__unclassified_g__Acidothermus	OTU1769
d__Bacteria	k__norank_ d__Bacteria	p__Actinobacteriota	c__Thermoleophilia	o__Solirubrobacterales	f__unclassified_o__Solirubrobacterales	g__unclassified_o__Solirubrobacterale s	s__unclassified_o__Solirubrobacterales	OTU372

d__Bacteria	k__norank_d__Bacteria	p__Actinobacteriota	c__Acidimicrobiia	o__unclassified_c__Acidimicrobiia	f__unclassified_c__Acidimicrobiia	g__unclassified_c__Acidimicrobiia	s__unclassified_c__Acidimicrobiia	OTU262
d__Bacteria	k__norank_d__Bacteria	p__unclassified_k__norank_d__Bacteria	c__unclassified_k__norank_d__Bacteria	o__unclassified_k__norank_d__Bacteria	f__unclassified_k__norank_d__Bacteria	g__unclassified_k__norank_d__Bacteria	s__unclassified_k__norank_d__Bacteria	OTU1888
d__Bacteria	k__norank_d__Bacteria	p__Patescibacteria	c__Saccharimonadia	o__Saccharimonadales	f__LWQ8	g__norank_f__LWQ8	s__uncultured_bacterium_g__norank_f__LWQ8	OTU2360
d__Bacteria	k__norank_d__Bacteria	p__Chloroflexi	c__Ktedonobacteria	o__Ktedonobacterales	f__Ktedonobacteraceae	g__JG30a-KF-32	s__uncultured_Chloroflexi_bacterium_g__JG30a-KF-32	OTU1905
d__Bacteria	k__norank_d__Bacteria	p__Proteobacteria	c__Gammaproteobacteria	o__Gammaproteobacteria_Incertae_Sedis	f__unclassified_o__Gammaproteobacteria_Incertae_Sedis	g__Candidatus_Ovatusbacter	s__uncultured_bacterium_g__Candidatus_Ovatusbacter	OTU883
d__Bacteria	k__norank_d__Bacteria	p__Chloroflexi	c__Chloroflexia	o__Thermomicrobiales	f__JG30-KF-CM45	g__norank_f__JG30-KF-CM45	s__uncultured_bacterium_g__norank_f__JG30-KF-CM45	OTU1116
d__Bacteria	k__norank_d__Bacteria	p__Chloroflexi	c__Ktedonobacteria	o__Ktedonobacterales	f__Ktedonobacteraceae	g__JG30a-KF-32	s__uncultured_Chloroflexi_bacterium_g__JG30a-KF-32	OTU1901
d__Bacteria	k__norank_d__Bacteria	p__Proteobacteria	c__Alphaproteobacteria	o__Acetobacterales	f__Acetobacteraceae	g__Endobacter	s__uncultured_bacterium_g__Endobacter	OTU709
d__Bacteria	k__norank_d__Bacteria	p__SAR324_cladeMarine_group_B	c__norank_p__SAR324_cladeMarine_group_B	o__norank_c__norank_p__SAR324_cladeMarine_group_B	f__norank_o__norank_c__norank_p__SAR324_cladeMarine_group_B	g__norank_f__norank_o__norank_c__norank_p__SAR324_cladeMarine_group_B	s__uncultured_bacterium_g__norank_f__norank_o__norank_c__norank_p__SAR324_cladeMarine_group_B	OTU406
d__Bacteria	k__norank_d__Bacteria	p__Myxococcota	c__Polyangia	o__Haliangiales	f__Haliangiaceae	g__Haliangium	s__uncultured_bacterium_g__Haliangium	OTU225
d__Bacteria	k__norank_d__Bacteria	p__Gemmatimonadota	c__Gemmatimonadetes	o__Gemmatimonadales	f__Gemmatimonadaceae	g__Roseisolibacter	s__uncultured_bacterium_g__Roseisolibacter	OTU1098
d__Bacteria	k__norank_d__Bacteria	p__Proteobacteria	c__Alphaproteobacteria	o__Acetobacterales	f__Acetobacteraceae	g__Acidiphilium	s__uncultured_bacterium_g__Acidiphilium	OTU1909
d__Bacteria	k__norank_d__Bacteria	p__Myxococcota	c__Polyangia	o__Blfdi19	f__norank_o__Blfdi19	g__norank_f__norank_o__Blfdi19	s__Anaeromyxobacter_dehalogenans_g__norank	OTU565
d__Bacteria	k__norank_d__Bacteria	p__Proteobacteria	c__Alphaproteobacteria	o__Sphingomonadales	f__Sphingomonadaceae	g__unclassified_f__Sphingomonadaceae	s__unclassified_f__Sphingomonadaceae	OTU2256
d__Bacteria	k__norank_d__Bacteria	p__Actinobacteriota	c__Thermoleophilia	o__Gaiellales	f__norank_o__Gaiellales	g__norank_f__norank_o__Gaiellales	s__metagenome_g__norank_f__norank_o__Gaiellales	OTU357
d__Bacteria	k__norank_d__Bacteria	p__Chloroflexi	c__Anaerolineae	o__SBR1031	f__norank_o__SBR1031	g__norank_f__norank_o__SBR1031	s__uncultured_Gemmatimonadetes_bacterium_g__norank_f__norank	OTU1465

d__Bacteria	k__norank_d__Bacteria	p__Cyanobacteria	c__Cyanobacteriia	o__Chloroplast	f__norank_o__Chloroplast	g__norank_f__norank_o__Chloroplast	s__unclassified_g__norank_f__norank_o__Chloroplast	OTU1221
d__Bacteria	k__norank_d__Bacteria	p__Gemmatimonadota	c__Gemmatimonadetes	o__Gemmatimonadales	f__Gemmatimonadaceae	g__Gemmatimonas	s__uncultured_bacterium_g__Gemmatimonas	OTU1567
d__Bacteria	k__norank_d__Bacteria	p__Myxococcota	c__Polyangia	o__mle1-27	f__norank_o__mle1-27	g__norank_f__norank_o__mle1-27	s__unclassified_g__norank_f__norank_o__mle1-27	OTU1865
d__Bacteria	k__norank_d__Bacteria	p__Firmicutes	c__Bacilli	o__Paenibacillales	f__Paenibacillaceae	g__Paenibacillus	s__unclassified_g__Paenibacillus	OTU1536
d__Bacteria	k__norank_d__Bacteria	p__Chloroflexi	c__unclassified_p__Chloroflexi	o__unclassified_p__Chloroflexi	f__unclassified_p__Chloroflexi	g__unclassified_p__Chloroflexi	s__unclassified_p__Chloroflexi	OTU1516
d__Bacteria	k__norank_d__Bacteria	p__Actinobacteriota	c__Acidimicrobiia	o__IMCC26256	f__norank_o__IMCC26256	g__norank_f__norank_o__IMCC26256	s__uncultured_bacterium_g__norank_f__norank_o__IMCC26256	OTU1389
d__Bacteria	k__norank_d__Bacteria	p__Chloroflexi	c__Chloroflexia	o__Thermomicrobiales	f__JG30-KF-CM45	g__norank_f__JG30-KF-CM45	s__metagenome_g__norank_f__JG30-KF-CM45	OTU202
d__Bacteria	k__norank_d__Bacteria	p__Actinobacteriota	c__Thermoleophilia	o__Solirubrobacterales	f__Solirubrobacteraceae	g__Conexibacter	s__unclassified_g__Conexibacter	OTU1279
d__Bacteria	k__norank_d__Bacteria	p__Proteobacteria	c__Alphaproteobacteria	o__Acetobacterales	f__Acetobacteraceae	g__unclassified_f__Acetobacteraceae	s__unclassified_f__Acetobacteraceae	OTU1852
d__Bacteria	k__norank_d__Bacteria	p__Acidobacteriota	c__Acidobacteriae	o__Solibacterales	f__Solibacteraceae	g__Candidatus_Solibacter	s__uncultured_bacterium_g__Candidatus_Solibacter	OTU1978
d__Bacteria	k__norank_d__Bacteria	p__Actinobacteriota	c__Thermoleophilia	o__Gaiellales	f__norank_o__Gaiellales	g__norank_f__norank_o__Gaiellales	s__unclassified_g__norank_f__norank_o__Gaiellales	OTU2502
d__Bacteria	k__norank_d__Bacteria	p__Chloroflexi	c__Dehalococcoidia	o__S085	f__norank_o__S085	g__norank_f__norank_o__S085	s__metagenome_g__norank_f__norank_o__S085	OTU2219
d__Bacteria	k__norank_d__Bacteria	p__Patescibacteria	c__WWE3	o__norank_c__WWE3	f__norank_o__norank_c__WWE3	g__norank_f__norank_o__norank_c__WWE3	s__unclassified_g__norank_f__norank_o__norank_c__WWE3	OTU1522
d__Bacteria	k__norank_d__Bacteria	p__Proteobacteria	c__Gammaproteobacteria	o__Vibrionales	f__Vibrionaceae	g__Vibrio	s__Vibrio_campbellii	OTU1663
d__Bacteria	k__norank_d__Bacteria	p__Armatimonadota	c__norank_p__Armatimonadota	o__norank_c__norank_p__Armatimonadota	f__norank_o__norank_c__norank_p__Armatimonadota	g__norank_f__norank_o__norank_c__norank_p__Armatimonadota	s__uncultured_actinobacterium_g__norank_f__norank_o__norank_c__norank_p__Armatimonadota	OTU265
d__Bacteria	k__norank_d__Bacteria	p__unclassified_k__norank_d__Bacteria	c__unclassified_k__norank_d__Bacteria	o__unclassified_k__norank_d__Bacteria	f__unclassified_k__norank_d__Bacteria	g__unclassified_k__norank_d__Bacteria	s__unclassified_k__norank_d__Bacteria	OTU472

d__Bacteria	k__norank_d__Bacteria	p__Myxococcota	c__Polyangia	o__Polyangiales	f__Sandaracinaceae	g__norank_f__Sandaracinaceae	s__metagenome_g__norank_f__Sandaracinaceae	OTU1428
d__Bacteria	k__norank_d__Bacteria	p__Bacteroidota	c__Bacteroidia	o__Sphingobacteriales	f__Sphingobacteriaceae	g__Arcticibacter	s__uncultured_bacterium_g__Arcticibacter	OTU313
d__Bacteria	k__norank_d__Bacteria	p__Patescibacteria	c__unclassified_p__Patescibacteria	o__unclassified_p__Patescibacteria	f__unclassified_p__Patescibacteria	g__unclassified_p__Patescibacteria	s__unclassified_p__Patescibacteria	OTU996
d__Bacteria	k__norank_d__Bacteria	p__Planctomycetota	c__Planctomycetes	o__Gemmatales	f__Gemmataceae	g__norank_f__Gemmataceae	s__uncultured_bacterium_g__norank_f__Gemmataceae	OTU1238
d__Bacteria	k__norank_d__Bacteria	p__Proteobacteria	c__Gammaproteobacteria	o__Burkholderiales	f__Nitrosomonadaceae	g__Ellin6067	s__unclassified_g__Ellin6067	OTU954
d__Bacteria	k__norank_d__Bacteria	p__Armatimonadota	c__norank_p__Armatimonadota	o__norank_c__norank_p__Armatimonadota	f__norank_o__norank_c__norank_p__Armatimonadota	g__norank_f__norank_o__norank_c__norank_p__Armatimonadota	s__uncultured_soil_bacterium_g__norank_f__norank_o__norank_c__norank_p__Armatimonadota	OTU1531
d__Bacteria	k__norank_d__Bacteria	p__Actinobacteriota	c__Actinobacteria	o__Propionibacteriales	f__Nocardioidaceae	g__Nocardioides	s__unclassified_g__Nocardioides	OTU2521
d__Bacteria	k__norank_d__Bacteria	p__Armatimonadota	c__norank_p__Armatimonadota	o__norank_c__norank_p__Armatimonadota	f__norank_o__norank_c__norank_p__Armatimonadota	g__norank_f__norank_o__norank_c__norank_p__Armatimonadota	s__uncultured_bacterium_g__norank_f__norank_o__norank_c__norank_p__Armatimonadota	OTU911
d__Bacteria	k__norank_d__Bacteria	p__Chloroflexi	c__Anaerolineae	o__SBR1031	f__norank_o__SBR1031	g__norank_f__norank_o__SBR1031	s__metagenome_g__norank_f__norank_o__SBR1031	OTU605
d__Bacteria	k__norank_d__Bacteria	p__Bacteroidota	c__Bacteroidia	o__Sphingobacteriales	f__KD3-93	g__norank_f__KD3-93	s__metagenome_g__norank_f__KD3-93	OTU2278
d__Bacteria	k__norank_d__Bacteria	p__Actinobacteriota	c__Actinobacteria	o__Pseudonocardiales	f__Pseudonocardiaceae	g__Pseudonocardia	s__uncultured_bacterium_g__Pseudonocardia	OTU1260
d__Bacteria	k__norank_d__Bacteria	p__Patescibacteria	c__Saccharimonadia	o__Saccharimonadales	f__WWH38	g__norank_f__WWH38	s__unclassified_g__norank_f__WWH38	OTU962
d__Bacteria	k__norank_d__Bacteria	p__Myxococcota	c__Myxococcia	o__Myxococcales	f__Myxococcaceae	g__P3OB-42	s__metagenome_g__P3OB-42	OTU2063
d__Bacteria	k__norank_d__Bacteria	p__Proteobacteria	c__Gammaproteobacteria	o__Burkholderiales	f__Comamonadaceae	g__Hydrogenophaga	s__unclassified_g__Hydrogenophaga	OTU2567
d__Bacteria	k__norank_d__Bacteria	p__Planctomycetota	c__BD7-11	o__norank_c__BD7-11	f__norank_o__norank_c__BD7-11	g__norank_f__norank_o__norank_c__BD7-11	s__uncultured_bacterium_g__norank_f__norank_o__norank_c__BD7-11	OTU819
d__Bacteria	k__norank_d__Bacteria	p__Proteobacteria	c__Alphaproteobacteria	o__Paracaedibacterales	f__Paracaedibacteraceae	g__norank_f__Paracaedibacteraceae	s__metagenome_g__norank_f__Paracaedibacteraceae	OTU1979

d__Bacteria	k__norank_ d__Bacteria	p__Chloroflexi	c__Anaerolineae	o__SBR1031	f__norank_o__SBR1031	g__norank_f__norank_o__SBR1031	s__uncultured_Gemmatimonadetes_bacterium_g__norank_f__norank	OTU2178
d__Bacteria	k__norank_ d__Bacteria	p__Actinobacteriota	c__Actinobacteria	o__Frankiales	f__unclassified_o__Frankiales	g__unclassified_o__Frankiales	s__unclassified_o__Frankiales	OTU1752
d__Bacteria	k__norank_ d__Bacteria	p__Armatimonadota	c__norank_p__Armatimon adota	o__norank_c__norank_p__ Armatimonadota	f__norank_o__norank_c__norank_p__Ar matimonadota	g__norank_f__norank_o__norank_c__ norank_p__Armatimonadota	s__uncultured_Firmicutes_bacterium_g__norank_f__norank_o__norank_c__norank _p__Armatimonadota	OTU2623
d__Bacteria	k__norank_ d__Bacteria	p__Bacteroidota	c__Bacteroidia	o__Cytophagales	f__Spirosomaceae	g__Spirosoma	s__uncultured_bacterium_g__Spirosoma	OTU153
d__Bacteria	k__norank_ d__Bacteria	p__Patescibacteria	c__Saccharimonadia	o__Saccharimonadales	f__norank_o__Saccharimonadales	g__norank_f__norank_o__Saccharimo nadales	s__uncultured_bacterium_g__norank_f__norank_o__Saccharimonadales	OTU1517
d__Bacteria	k__norank_ d__Bacteria	p__Acidobacteriota	c__Acidobacteriae	o__Solibacterales	f__Solibacteraceae	g__Candidatus_Solibacter	s__unclassified_g__Candidatus_Solibacter	OTU1853
d__Bacteria	k__norank_ d__Bacteria	p__Actinobacteriota	c__Thermoleophilia	o__Gaiellales	f__norank_o__Gaiellales	g__norank_f__norank_o__Gaiellales	s__metagenome_g__norank_f__norank_o__Gaiellales	OTU2577
d__Bacteria	k__norank_ d__Bacteria	p__Proteobacteria	c__Alphaproteobacteria	o__Rhizobiales	f__Beijerinckiaceae	g__Methylorosula	s__uncultured_bacterium_g__Methylorosula	OTU300
d__Bacteria	k__norank_ d__Bacteria	p__Chloroflexi	c__Ktedonobacteria	o__C0119	f__norank_o__C0119	g__norank_f__norank_o__C0119	s__uncultured_soil_bacterium_g__norank_f__norank_o__C0119	OTU1470
d__Bacteria	k__norank_ d__Bacteria	p__Bacteroidota	c__Bacteroidia	o__Cytophagales	f__Microscillaceae	g__norank_f__Microscillaceae	s__metagenome_g__norank_f__Microscillaceae	OTU2286
d__Bacteria	k__norank_ d__Bacteria	p__Actinobacteriota	c__Actinobacteria	o__Frankiales	f__Frankiaceae	g__Jatrophihabitans	s__uncultured_bacterium_g__Jatrophihabitans	OTU1699
d__Bacteria	k__norank_ d__Bacteria	p__Bacteroidota	c__Bacteroidia	o__Cytophagales	f__Hymenobacteraceae	g__Hymenobacter	s__unclassified_g__Hymenobacter	OTU1916
d__Bacteria	k__norank_ d__Bacteria	p__Bacteroidota	c__Bacteroidia	o__Cytophagales	f__Microscillaceae	g__norank_f__Microscillaceae	s__unclassified_g__norank_f__Microscillaceae	OTU2192
d__Bacteria	k__norank_ d__Bacteria	p__Bdellovibrionota	c__Bdellovibrionia	o__Bacteriovoracales	f__Bacteriovoracaceae	g__Peredibacter	s__unclassified_g__Peredibacter	OTU184
d__Bacteria	k__norank_ d__Bacteria	p__Bacteroidota	c__Bacteroidia	o__Flavobacteriales	f__Flavobacteriaceae	g__Flavobacterium	s__unclassified_g__Flavobacterium	OTU1834
d__Bacteria	k__norank_ d__Bacteria	p__Bacteroidota	c__Bacteroidia	o__Chitinophagales	f__Chitinophagaceae	g__Parasegetibacter	s__unclassified_g__Parasegetibacter	OTU169

d__Bacteria	k__norank_ d__Bacteria	p__Proteobacteria	c__Gammaproteobacteria	o__Burkholderiales	f__Comamonadaceae	g__Variovorax	s__metagenome_g__Variovorax	OTU259
d__Bacteria	k__norank_ d__Bacteria	p__Planctomycetota	c__Planctomycetes	o__Gemmatales	f__Gemmataceae	g__norank_f__Gemmataceae	s__unclassified_g__norank_f__Gemmataceae	OTU1142
d__Bacteria	k__norank_ d__Bacteria	p__Myxococcota	c__Myxococcia	o__Myxococcales	f__Myxococcaceae	g__unclassified_f__Myxococcaceae	s__unclassified_f__Myxococcaceae	OTU39
d__Bacteria	k__norank_ d__Bacteria	p__Bacteroidota	c__Bacteroidia	o__Flavobacteriales	f__Weeksellaceae	g__Empedobacter	s__Empedobacter_brevis_g__Empedobacter	OTU2987
d__Bacteria	k__norank_ d__Bacteria	p__Proteobacteria	c__Gammaproteobacteria	o__Diplorickettsiales	f__Diplorickettsiaceae	g__norank_f__Diplorickettsiaceae	s__unclassified_g__norank_f__Diplorickettsiaceae	OTU780
d__Bacteria	k__norank_ d__Bacteria	p__Armatimonadota	c__Armatimonadia	o__Armatimonadales	f__norank_o__Armatimonadales	g__norank_f__norank_o__Armatimona dales	s__uncultured_bacterium_g__norank_f__norank_o__Armatimonadales	OTU2993
d__Bacteria	k__norank_ d__Bacteria	p__Bacteroidota	c__Bacteroidia	o__Sphingobacteriales	f__Sphingobacteriaceae	g__Pedobacter	s__uncultured_Bacteroidetes_bacterium_g__Pedobacter	OTU284
d__Bacteria	k__norank_ d__Bacteria	p__Proteobacteria	c__Alphaproteobacteria	o__Rhizobiales	f__Pleomorphomonadaceae	g__Prosthecomicrobium	s__unclassified_g__Prosthecomicrobium	OTU423
d__Bacteria	k__norank_ d__Bacteria	p__Proteobacteria	c__Alphaproteobacteria	o__Rhizobiales	f__Devosiaceae	g__Devosia	s__unclassified_g__Devosia	OTU2158
d__Bacteria	k__norank_ d__Bacteria	p__Acidobacteriota	c__Holophagae	o__Subgroup_7	f__norank_o__Subgroup_7	g__norank_f__norank_o__Subgroup_7	s__unclassified_g__norank_f__norank_o__Subgroup_7	OTU853
d__Bacteria	k__norank_ d__Bacteria	p__Cyanobacteria	c__Sericytochromatia	o__norank_c__Sericytochro matia	f__norank_o__norank_c__Sericytochrom atia	g__norank_f__norank_o__norank_c__ Sericytochromatia	s__unclassified_g__norank_f__norank_o__norank_c__Sericytochromatia	OTU1690
d__Bacteria	k__norank_ d__Bacteria	p__Bacteroidota	c__Bacteroidia	o__Cytophagales	f__norank_o__Cytophagales	g__norank_f__norank_o__Cytophagale s	s__uncultured_bacterium_g__norank_f__norank_o__Cytophagales	OTU614
d__Bacteria	k__norank_ d__Bacteria	p__Cyanobacteria	c__Cyanobacteriia	o__Chloroplast	f__norank_o__Chloroplast	g__norank_f__norank_o__Chloroplast	s__Ettlia_pseudoalveolaris	OTU677
d__Bacteria	k__norank_ d__Bacteria	p__Cyanobacteria	c__Cyanobacteriia	o__Chloroplast	f__norank_o__Chloroplast	g__norank_f__norank_o__Chloroplast	s__Dryopteris_fragrans	OTU1805
d__Bacteria	k__norank_ d__Bacteria	p__Bdellovibrionota	c__Oligoflexia	o__0319-6G20	f__norank_o__0319-6G20	g__norank_f__norank_o__0319-6G20	s__unclassified_g__norank_f__norank_o__0319-6G20	OTU1803
d__Bacteria	k__norank_ d__Bacteria	p__Bdellovibrionota	c__Oligoflexia	o__0319-6G20	f__norank_o__0319-6G20	g__norank_f__norank_o__0319-6G20	s__unclassified_g__norank_f__norank_o__0319-6G20	OTU833

d__Bacteria	k__norank_d__Bacteria	p__Gemmatimonadota	c__Gemmatimonadetes	o__Gemmatimonadales	f__Gemmatimonadaceae	g__norank_f__Gemmatimonadaceae	s__uncultured_prokaryote_g__norank_f__Gemmatimonadaceae	OTU2571
d__Bacteria	k__norank_d__Bacteria	p__Proteobacteria	c__Alphaproteobacteria	o__Rhodobacterales	f__Rhodobacteraceae	g__Defluviimonas	s__uncultured_bacterium_g__Defluviimonas	OTU506
d__Bacteria	k__norank_d__Bacteria	p__Actinobacteriota	c__Acidimicrobiia	o__Microtrichales	f__norank_o__Microtrichales	g__norank_f__norank_o__Microtrichales	s__uncultured_bacterium_g__norank_f__norank_o__Microtrichales	OTU1358
d__Bacteria	k__norank_d__Bacteria	p__Campilobacterota	c__Campylobacteria	o__Campylobacterales	f__Arcobacteraceae	g__Halarcobacter	s__Halarcobacter_bivalviorum	OTU501
d__Bacteria	k__norank_d__Bacteria	p__Actinobacteriota	c__unclassified_p__Actinobacteriota	o__unclassified_p__Actinobacteriota	f__unclassified_p__Actinobacteriota	g__unclassified_p__Actinobacteriota	s__unclassified_p__Actinobacteriota	OTU1512
d__Bacteria	k__norank_d__Bacteria	p__Myxococcota	c__Polyangia	o__Haliangiales	f__Haliangiaceae	g__Haliangium	s__unclassified_g__Haliangium	OTU1890
d__Bacteria	k__norank_d__Bacteria	p__Proteobacteria	c__Alphaproteobacteria	o__Acetobacterales	f__Acetobacteraceae	g__Craurococcus-Caldovatus	s__uncultured_bacterium_g__Craurococcus-Caldovatus	OTU881
d__Bacteria	k__norank_d__Bacteria	p__Proteobacteria	c__Alphaproteobacteria	o__Caulobacterales	f__Hyphomonadaceae	g__SWB02	s__uncultured_bacterium_g__SWB02	OTU1586
d__Bacteria	k__norank_d__Bacteria	p__Planctomycetota	c__Planctomycetes	o__Isosphaerales	f__Isosphaeraceae	g__unclassified_f__Isosphaeraceae	s__unclassified_f__Isosphaeraceae	OTU1751
d__Bacteria	k__norank_d__Bacteria	p__Firmicutes	c__Bacilli	o__Bacillales	f__Planococcaceae	g__Sporosarcina	s__Sporosarcina_globispora	OTU1672
d__Bacteria	k__norank_d__Bacteria	p__Actinobacteriota	c__Acidimicrobiia	o__IMCC26256	f__norank_o__IMCC26256	g__norank_f__norank_o__IMCC26256	s__uncultured_bacterium_g__norank_f__norank_o__IMCC26256	OTU1034
d__Bacteria	k__norank_d__Bacteria	p__Proteobacteria	c__Gammaproteobacteria	o__Xanthomonadales	f__Rhodanobacteraceae	g__norank_f__Rhodanobacteraceae	s__uncultured_soil_bacterium_g__norank_f__Rhodanobacteraceae	OTU2609
d__Bacteria	k__norank_d__Bacteria	p__Acidobacteriota	c__Acidobacteriae	o__Bryobacterales	f__Bryobacteraceae	g__Bryobacter	s__unclassified_g__Bryobacter	OTU1051
d__Bacteria	k__norank_d__Bacteria	p__Chloroflexi	c__Ktedonobacteria	o__Ktedonobacterales	f__JG30-KF-AS9	g__norank_f__JG30-KF-AS9	s__uncultured_bacterium_g__norank_f__JG30-KF-AS9	OTU1963
d__Bacteria	k__norank_d__Bacteria	p__Actinobacteriota	c__Acidimicrobiia	o__IMCC26256	f__norank_o__IMCC26256	g__norank_f__norank_o__IMCC26256	s__uncultured_Ferrimicrobium_sp._g__norank	OTU1488
d__Bacteria	k__norank_d__Bacteria	p__Bdellovibrionota	c__Bdellovibrionia	o__Bdellovibrionales	f__Bdellovibrionaceae	g__Bdellovibrio	s__uncultured_bacterium_g__Bdellovibrio	OTU78

d__Bacteria	k__norank_ d__Bacteria	p__Planctomycetota	c__Planctomycetes	o__Gemmatales	f__Gemmataceae	g__norank_f__Gemmataceae	s__unclassified_g__norank_f__Gemmataceae	OTU1037
d__Bacteria	k__norank_ d__Bacteria	p__Gemmatimonadota	c__Gemmatimonadetes	o__Gemmatimonadales	f__Gemmatimonadaceae	g__Roseisolibacter	s__unclassified_g__Roseisolibacter	OTU1680
d__Bacteria	k__norank_ d__Bacteria	p__Cyanobacteria	c__Cyanobacteriia	o__Phormidesmiales	f__Nodosilineaceae	g__Nodosilinea_PCC-7104	s__uncultured_bacterium_g__Nodosilinea_PCC-7104	OTU2886
d__Bacteria	k__norank_ d__Bacteria	p__Proteobacteria	c__Gammaproteobacteria	o__Burkholderiales	f__Comamonadaceae	g__Rhizobacter	s__unclassified_g__Rhizobacter	OTU1772
d__Bacteria	k__norank_ d__Bacteria	p__Chloroflexi	c__Chloroflexia	o__Chloroflexales	f__Roseiflexaceae	g__norank_f__Roseiflexaceae	s__uncultured_bacterium_g__norank_f__Roseiflexaceae	OTU2244
d__Bacteria	k__norank_ d__Bacteria	p__Chloroflexi	c__Anaerolineae	o__SBR1031	f__norank_o__SBR1031	g__norank_f__norank_o__SBR1031	s__uncultured_Gemmatimonadetes_bacterium_g__norank_f__norank	OTU1401
d__Bacteria	k__norank_ d__Bacteria	p__Bacteroidota	c__Bacteroidia	o__Cytophagales	f__Hymenobacteraceae	g__Adhaeribacter	s__uncultured_Hymenobacter_group_bacterium_g__Adhaeribacter	OTU2213
d__Bacteria	k__norank_ d__Bacteria	p__Actinobacteriota	c__Thermoleophilia	o__Solirubrobacterales	f__Solirubrobacteraceae	g__norank_f__Solirubrobacteraceae	s__Colobanthus_quitensis_g__norank_f__Solirubrobacteraceae	OTU2474
d__Bacteria	k__norank_ d__Bacteria	p__Proteobacteria	c__Gammaproteobacteria	o__Burkholderiales	f__A21b	g__norank_f__A21b	s__uncultured_bacterium_g__norank_f__A21b	OTU1545
d__Bacteria	k__norank_ d__Bacteria	p__Bacteroidota	c__Bacteroidia	o__Cytophagales	f__Hymenobacteraceae	g__Hymenobacter	s__unclassified_g__Hymenobacter	OTU2733
d__Bacteria	k__norank_ d__Bacteria	p__Chloroflexi	c__Ktedonobacteria	o__B12-WMSP1	f__norank_o__B12-WMSP1	g__norank_f__norank_o__B12-WMSP 1	s__uncultured_bacterium_g__norank_f__norank_o__B12-WMSP1	OTU803
d__Bacteria	k__norank_ d__Bacteria	p__Bacteroidota	c__Bacteroidia	o__Cytophagales	f__Hymenobacteraceae	g__Hymenobacter	s__unclassified_g__Hymenobacter	OTU2737
d__Bacteria	k__norank_ d__Bacteria	p__Chloroflexi	c__Chloroflexia	o__Chloroflexales	f__Roseiflexaceae	g__norank_f__Roseiflexaceae	s__uncultured_bacterium_g__norank_f__Roseiflexaceae	OTU624
d__Bacteria	k__norank_ d__Bacteria	p__Gemmatimonadota	c__Gemmatimonadetes	o__Gemmatimonadales	f__Gemmatimonadaceae	g__unclassified_f__Gemmatimonadace ae	s__unclassified_f__Gemmatimonadaceae	OTU1012
d__Bacteria	k__norank_ d__Bacteria	p__Proteobacteria	c__Alphaproteobacteria	o__Rhizobiales	f__Rhizobiaceae	g__Aureimonas	s__uncultured_bacterium_g__Aureimonas	OTU526
d__Bacteria	k__norank_ d__Bacteria	p__Actinobacteriota	c__Actinobacteria	o__Frankiales	f__norank_o__Frankiales	g__norank_f__norank_o__Frankiales	s__uncultured_bacterium_g__norank_f__norank_o__Frankiales	OTU125

d__Bacteria	k__norank_d__Bacteria	p__Actinobacteriota	c__MB-A2-108	o__norank_c__MB-A2-108	f__norank_o__norank_c__MB-A2-108	g__norank_f__norank_o__norank_c__MB-A2-108	s__unclassified_g__norank_f__norank_o__norank_c__MB-A2-108	OTU1524
d__Bacteria	k__norank_d__Bacteria	p__Acidobacteriota	c__Acidobacteriae	o__Bryobacterales	f__Bryobacteraceae	g__Bryobacter	s__unclassified_g__Bryobacter	OTU1940
d__Bacteria	k__norank_d__Bacteria	p__Proteobacteria	c__Gammaproteobacteria	o__Burkholderiales	f__Nitrosomonadaceae	g__mle1-7	s__unclassified_g__mle1-7	OTU113
d__Bacteria	k__norank_d__Bacteria	p__Planctomycetota	c__Planctomycetes	o__Gemmatales	f__Gemmataceae	g__norank_f__Gemmataceae	s__uncultured_bacterium_g__norank_f__Gemmataceae	OTU1339
d__Bacteria	k__norank_d__Bacteria	p__Actinobacteriota	c__Actinobacteria	o__Propionibacteriales	f__Propionibacteriaceae	g__Microlunatus	s__unclassified_g__Microlunatus	OTU298
d__Bacteria	k__norank_d__Bacteria	p__Actinobacteriota	c__Actinobacteria	o__0319-7L14	f__norank_o__0319-7L14	g__norank_f__norank_o__0319-7L14	s__uncultured_bacterium_g__norank_f__norank_o__0319-7L14	OTU1550
d__Bacteria	k__norank_d__Bacteria	p__Armatimonadota	c__norank_p__Armatimonadota	o__norank_c__norank_p__Armatimonadota	f__norank_o__norank_c__norank_p__Armatimonadota	g__norank_f__norank_o__norank_c__norank_p__Armatimonadota	s__unclassified_g__norank_f__norank_o__norank_c__norank_p__Armatimonadota	OTU1163
d__Bacteria	k__norank_d__Bacteria	p__Actinobacteriota	c__Actinobacteria	o__Frankiales	f__Geodermatophilaceae	g__Blastococcus	s__uncultured_bacterium_g__Blastococcus	OTU226
d__Bacteria	k__norank_d__Bacteria	p__Proteobacteria	c__Gammaproteobacteria	o__Burkholderiales	f__Comamonadaceae	g__Variovorax	s__unclassified_g__Variovorax	OTU2522
d__Bacteria	k__norank_d__Bacteria	p__Chloroflexi	c__Chloroflexia	o__Thermomicrobiales	f__JG30-KF-CM45	g__norank_f__JG30-KF-CM45	s__uncultured_soil_bacterium_g__norank_f__JG30-KF-CM45	OTU712
d__Bacteria	k__norank_d__Bacteria	p__Myxococcota	c__Polyangia	o__Haliangiales	f__Haliangiaceae	g__Haliangium	s__unclassified_g__Haliangium	OTU98
d__Bacteria	k__norank_d__Bacteria	p__Proteobacteria	c__Gammaproteobacteria	o__Burkholderiales	f__Oxalobacteraceae	g__Noviherbaspirillum	s__unclassified_g__Noviherbaspirillum	OTU42
d__Bacteria	k__norank_d__Bacteria	p__Patescibacteria	c__Saccharimonadia	o__Saccharimonadales	f__LWQ8	g__norank_f__LWQ8	s__uncultured_bacterium_g__norank_f__LWQ8	OTU2694
d__Bacteria	k__norank_d__Bacteria	p__Chloroflexi	c__Chloroflexia	o__Kallotenuales	f__AKIW781	g__norank_f__AKIW781	s__uncultured_bacterium_g__norank_f__AKIW781	OTU2160
d__Bacteria	k__norank_d__Bacteria	p__Proteobacteria	c__Gammaproteobacteria	o__Burkholderiales	f__Comamonadaceae	g__Caenimonas	s__uncultured_bacterium_g__Caenimonas	OTU2463
d__Bacteria	k__norank_d__Bacteria	p__Myxococcota	c__Myxococcia	o__Myxococcales	f__Myxococcaceae	g__P3OB-42	s__unclassified_g__P3OB-42	OTU907

d__Bacteria	k__norank_ d__Bacteria	p__Bdellovibrionota	c__Bdellovibrionia	o__Bdellovibrionales	f__Bdellovibrionaceae	g__Bdellovibrio	s__uncultured_bacterium_g__Bdellovibrio	OTU2758
d__Bacteria	k__norank_ d__Bacteria	p__Chloroflexi	c__Ktedonobacteria	o__C0119	f__norank_o__C0119	g__norank_f__norank_o__C0119	s__uncultured_bacterium_g__norank_f__norank_o__C0119	OTU2514
d__Bacteria	k__norank_ d__Bacteria	p__Armatimonadota	c__Fimbriimonadia	o__Fimbriimonadales	f__Fimbriimonadaceae	g__norank_f__Fimbriimonadaceae	s__uncultured_Armatimonadetes_bacterium_g__norank_f__Fimbriimonadaceae	OTU1166
d__Bacteria	k__norank_ d__Bacteria	p__Planctomycetota	c__Planctomycetes	o__Gemmatales	f__Gemmataceae	g__norank_f__Gemmataceae	s__uncultured_bacterium_g__norank_f__Gemmataceae	OTU1203
d__Bacteria	k__norank_ d__Bacteria	p__Chloroflexi	c__Ktedonobacteria	o__C0119	f__norank_o__C0119	g__norank_f__norank_o__C0119	s__uncultured_bacterium_g__norank_f__norank_o__C0119	OTU678
d__Bacteria	k__norank_ d__Bacteria	p__Abditibacteriota	c__Abditibacteria	o__Abditibacteriales	f__Abditibacteriaceae	g__Abditibacterium	s__uncultured_bacterium_g__Abditibacterium	OTU1373
d__Bacteria	k__norank_ d__Bacteria	p__Bacteroidota	c__Bacteroidia	o__Sphingobacteriales	f__AKYH767	g__norank_f__AKYH767	s__uncultured_bacterium_g__norank_f__AKYH767	OTU2084
d__Bacteria	k__norank_ d__Bacteria	p__Armatimonadota	c__Armatimonadia	o__Armatimonadales	f__norank_o__Armatimonadales	g__norank_f__norank_o__Armatimona dales	s__unclassified_g__norank_f__norank_o__Armatimonadales	OTU1847
d__Bacteria	k__norank_ d__Bacteria	p__Chloroflexi	c__JG30-KF-CM66	o__norank_c__JG30-KF-C M66	f__norank_o__norank_c__JG30-KF-CM 66	g__norank_f__norank_o__norank_c__ JG30-KF-CM66	s__unclassified_g__norank_f__norank_o__norank_c__JG30-KF-CM66	OTU1049
d__Bacteria	k__norank_ d__Bacteria	p__Proteobacteria	c__Gammaproteobacteria	o__Coxiellales	f__Coxiellaceae	g__Coxiella	s__unclassified_g__Coxiella	OTU2723
d__Bacteria	k__norank_ d__Bacteria	p__Myxococcota	c__Polyangia	o__Haliangiales	f__Haliangiaceae	g__Haliangium	s__unclassified_g__Haliangium	OTU127
d__Bacteria	k__norank_ d__Bacteria	p__Firmicutes	c__Bacilli	o__Bacillales	f__Bacillaceae	g__Bacillus	s__Bacillus_niacini_g__Bacillus	OTU317
d__Bacteria	k__norank_ d__Bacteria	p__Patescibacteria	c__Saccharimonadia	o__Saccharimonadales	f__norank_o__Saccharimonadales	g__norank_f__norank_o__Saccharimo nadales	s__unclassified_g__norank_f__norank_o__Saccharimonadales	OTU1924
d__Bacteria	k__norank_ d__Bacteria	p__Chloroflexi	c__Ktedonobacteria	o__C0119	f__norank_o__C0119	g__norank_f__norank_o__C0119	s__unclassified_g__norank_f__norank_o__C0119	OTU321
d__Bacteria	k__norank_ d__Bacteria	p__Proteobacteria	c__Gammaproteobacteria	o__Burkholderiales	f__SC-I-84	g__norank_f__SC-I-84	s__unclassified_g__norank_f__SC-I-84	OTU52
d__Bacteria	k__norank_ d__Bacteria	p__Bdellovibrionota	c__Bdellovibrionia	o__Bacteriovoracales	f__Bacteriovoracaceae	g__Peredibacter	s__unclassified_g__Peredibacter	OTU275

d__Bacteria	k__norank_ d__Bacteria	p__Armatimonadota	c__Armatimonadia	o__Armatimonadales	f__norank_o__Armatimonadales	g__norank_f__norank_o__Armatimonadales	s__uncultured_bacterium_g__norank_f__norank_o__Armatimonadales	OTU1766
d__Bacteria	k__norank_ d__Bacteria	p__Actinobacteriota	c__Thermoleophilia	o__Gaiellales	f__norank_o__Gaiellales	g__norank_f__norank_o__Gaiellales	s__unclassified_g__norank_f__norank_o__Gaiellales	OTU783
d__Bacteria	k__norank_ d__Bacteria	p__Armatimonadota	c__Chthonomonadetes	o__Chthonomonadales	f__Chthonomonadaceae	g__Chthonomonas	s__uncultured_bacterium_g__Chthonomonas	OTU798
d__Bacteria	k__norank_ d__Bacteria	p__Proteobacteria	c__Alphaproteobacteria	o__Rhizobiales	f__Beijerinckiaceae	g__Psychroglaciecola	s__unclassified_g__Psychroglaciecola	OTU2913
d__Bacteria	k__norank_ d__Bacteria	p__Patescibacteria	c__Saccharimonadia	o__Saccharimonadales	f__norank_o__Saccharimonadales	g__norank_f__norank_o__Saccharimonadales	s__uncultured_bacterium_g__norank_f__norank_o__Saccharimonadales	OTU891
d__Bacteria	k__norank_ d__Bacteria	p__Bdellovibrionota	c__Oligoflexia	o__Oligoflexales	f__norank_o__Oligoflexales	g__Oligoflexus	s__metagenome_g__Oligoflexus	OTU2069
d__Bacteria	k__norank_ d__Bacteria	p__Bacteroidota	c__Bacteroidia	o__Sphingobacteriales	f__env.OPS_17	g__norank_f__env.OPS_17	s__uncultured_Bacteroidetes_bacterium_g__norank_f__env.OPS_17	OTU1718
d__Bacteria	k__norank_ d__Bacteria	p__Cyanobacteria	c__Cyanobacteriia	o__Chloroplast	f__norank_o__Chloroplast	g__norank_f__norank_o__Chloroplast	s__uncultured_phototrophic_eukaryote_g__norank	OTU49
d__Bacteria	k__norank_ d__Bacteria	p__Acidobacteriota	c__Holophagae	o__Subgroup_7	f__norank_o__Subgroup_7	g__norank_f__norank_o__Subgroup_7	s__uncultured_bacterium_g__norank_f__norank_o__Subgroup_7	OTU1628
d__Bacteria	k__norank_ d__Bacteria	p__Acidobacteriota	c__Holophagae	o__Subgroup_7	f__norank_o__Subgroup_7	g__norank_f__norank_o__Subgroup_7	s__uncultured_Acidobacteria_bacterium_g__norank_f__norank_o__Subgroup_7	OTU842
d__Bacteria	k__norank_ d__Bacteria	p__Proteobacteria	c__Alphaproteobacteria	o__Rickettsiales	f__Mitochondria	g__norank_f__Mitochondria	s__Tetraphis_pellucida_g__norank	OTU2039
d__Bacteria	k__norank_ d__Bacteria	p__Planctomycetota	c__Planctomycetes	o__Isosphaerales	f__Isosphaeraceae	g__Tundrisphaera	s__uncultured_bacterium_g__Tundrisphaera	OTU985
d__Bacteria	k__norank_ d__Bacteria	p__RCP2-54	c__norank_p__RCP2-54	o__norank_c__norank_p__RCP2-54	f__norank_o__norank_c__norank_p__RCP2-54	g__norank_f__norank_o__norank_c__norank_p__RCP2-54	s__metagenome_g__norank_f__norank_o__norank_c__norank_p__RCP2-54	OTU2599
d__Bacteria	k__norank_ d__Bacteria	p__Verrucomicrobiota	c__Verrucomicrobiae	o__Chthoniobacterales	f__Chthoniobacteraceae	g__Candidatus_Udaeobacter	s__uncultured_bacterium_g__Candidatus_Udaeobacter	OTU793
d__Bacteria	k__norank_ d__Bacteria	p__Bacteroidota	c__Bacteroidia	o__Sphingobacteriales	f__env.OPS_17	g__norank_f__env.OPS_17	s__uncultured_Bacteroidetes_bacterium_g__norank_f__env.OPS_17	OTU909
d__Bacteria	k__norank_ d__Bacteria	p__Bacteroidota	c__Bacteroidia	o__Cytophagales	f__Hymenobacteraceae	g__Hymenobacter	s__unclassified_g__Hymenobacter	OTU1284

d__Bacteria	k__norank_ d__Bacteria	p__Proteobacteria	c__Gammaproteobacteria	o__Burkholderiales	f__Gallionellaceae	g__Candidatus_Nitrotoga	s__uncultured_bacterium_g__Candidatus_Nitrotoga	OTU2426
d__Bacteria	k__norank_ d__Bacteria	p__Myxococcota	c__Myxococcia	o__Myxococcales	f__Anaeromyxobacteraceae	g__Anaeromyxobacter	s__unclassified_g__Anaeromyxobacter	OTU2097
d__Bacteria	k__norank_ d__Bacteria	p__Acidobacteriota	c__Acidobacteriae	o__Solibacterales	f__Solibacteraceae	g__Candidatus_Solibacter	s__uncultured_bacterium_g__Candidatus_Solibacter	OTU111
d__Bacteria	k__norank_ d__Bacteria	p__Firmicutes	c__Bacilli	o__Lactobacillales	f__Lactobacillaceae	g__Lactobacillus	s__Lactobacillus_murinus	OTU485
d__Bacteria	k__norank_ d__Bacteria	p__Actinobacteriota	c__Actinobacteria	o__Frankiales	f__Acidothermaceae	g__Acidothermus	s__unclassified_g__Acidothermus	OTU2544
d__Bacteria	k__norank_ d__Bacteria	p__Proteobacteria	c__Alphaproteobacteria	o__Rhodobacterales	f__Rhodobacteraceae	g__Paracoccus	s__unclassified_g__Paracoccus	OTU500
d__Bacteria	k__norank_ d__Bacteria	p__Chloroflexi	c__Anaerolineae	o__SBR1031	f__norank_o__SBR1031	g__norank_f__norank_o__SBR1031	s__uncultured_bacterium_g__norank_f__norank_o__SBR1031	OTU947
d__Bacteria	k__norank_ d__Bacteria	p__Firmicutes	c__Clostridia	o__Peptostreptococcales-Tis sierellales	f__Peptostreptococcaceae	g__Peptostreptococcus	s__uncultured_organism_g__Peptostreptococcus	OTU371
d__Bacteria	k__norank_ d__Bacteria	p__Proteobacteria	c__Gammaproteobacteria	o__Burkholderiales	f__SC-I-84	g__norank_f__SC-I-84	s__uncultured_bacterium_g__norank_f__SC-I-84	OTU864
d__Bacteria	k__norank_ d__Bacteria	p__Chloroflexi	c__Anaerolineae	o__SBR1031	f__A4b	g__norank_f__A4b	s__uncultured_bacterium_g__norank_f__A4b	OTU2159
d__Bacteria	k__norank_ d__Bacteria	p__Chloroflexi	c__Chloroflexia	o__Thermomicrobiales	f__JG30-KF-CM45	g__norank_f__JG30-KF-CM45	s__unclassified_g__norank_f__JG30-KF-CM45	OTU224
d__Bacteria	k__norank_ d__Bacteria	p__Cyanobacteria	c__Cyanobacteriia	o__Chloroplast	f__norank_o__Chloroplast	g__norank_f__norank_o__Chloroplast	s__Xylochloris_irregularis	OTU2689
d__Bacteria	k__norank_ d__Bacteria	p__Bacteroidota	c__Bacteroidia	o__Cytophagales	f__Hymenobacteraceae	g__Hymenobacter	s__Hymenobacter_sp._APR13	OTU1540
d__Bacteria	k__norank_ d__Bacteria	p__Acidobacteriota	c__Acidobacteriae	o__unclassified_c__Acidoba cteriae	f__unclassified_c__Acidobacteriae	g__unclassified_c__Acidobacteriae	s__unclassified_c__Acidobacteriae	OTU1693
d__Bacteria	k__norank_ d__Bacteria	p__Actinobacteriota	c__Actinobacteria	o__Frankiales	f__Acidothermaceae	g__Acidothermus	s__unclassified_g__Acidothermus	OTU1845
d__Bacteria	k__norank_ d__Bacteria	p__Dependentiae	c__Babeliae	o__Babeliales	f__unclassified_o__Babeliales	g__unclassified_o__Babeliales	s__unclassified_o__Babeliales	OTU604

d__Bacteria	k__norank_ d__Bacteria	p__Bacteroidota	c__Bacteroidia	o__Chitinophagales	f__Chitinophagaceae	g__norank_f__Chitinophagaceae	s__unclassified_g__norank_f__Chitinophagaceae	OTU1941
d__Bacteria	k__norank_ d__Bacteria	p__Acidobacteriota	c__Blastocatellia	o__Blastocatellales	f__Blastocatellaceae	g__Blastocatella	s__uncultured_Acidobacteria_bacterium_g__Blastocatella	OTU197
d__Bacteria	k__norank_ d__Bacteria	p__Patescibacteria	c__Saccharimonadia	o__Saccharimonadales	f__norank_o__Saccharimonadales	g__norank_f__norank_o__Saccharimo nadales	s__uncultured_bacterium_g__norank_f__norank_o__Saccharimonadales	OTU2717
d__Bacteria	k__norank_ d__Bacteria	p__Acidobacteriota	c__Holophagae	o__Subgroup_7	f__norank_o__Subgroup_7	g__norank_f__norank_o__Subgroup_7	s__unclassified_g__norank_f__norank_o__Subgroup_7	OTU353
d__Bacteria	k__norank_ d__Bacteria	p__Cyanobacteria	c__Cyanobacteriia	o__Chloroplast	f__norank_o__Chloroplast	g__norank_f__norank_o__Chloroplast	s__uncultured_eubacterium_WD215	OTU2132
d__Bacteria	k__norank_ d__Bacteria	p__Proteobacteria	c__Alphaproteobacteria	o__Acetobacterales	f__Acetobacteraceae	g__Acidiphilium	s__uncultured_soil_bacterium_g__Acidiphilium	OTU1967
d__Bacteria	k__norank_ d__Bacteria	p__Acidobacteriota	c__Acidobacteriae	o__Acidobacteriales	f__Acidobacteriaceae_Subgroup_1	g__Granulicella	s__uncultured_Acidobacteria_bacterium_g__Granulicella	OTU1862
d__Bacteria	k__norank_ d__Bacteria	p__Gemmatimonadota	c__Gemmatimonadetes	o__Gemmatimonadales	f__Gemmatimonadaceae	g__norank_f__Gemmatimonadaceae	s__Gemmatimonadetes_bacterium_LX87	OTU1549
d__Bacteria	k__norank_ d__Bacteria	p__Gemmatimonadota	c__Gemmatimonadetes	o__Gemmatimonadales	f__Gemmatimonadaceae	g__Gemmatimonas	s__unclassified_g__Gemmatimonas	OTU1383
d__Bacteria	k__norank_ d__Bacteria	p__Proteobacteria	c__Alphaproteobacteria	o__Sphingomonadales	f__Sphingomonadaceae	g__unclassified_f__Sphingomonadacea e	s__unclassified_f__Sphingomonadaceae	OTU2245
d__Bacteria	k__norank_ d__Bacteria	p__Chloroflexi	c__Anaerolineae	o__SBR1031	f__norank_o__SBR1031	g__norank_f__norank_o__SBR1031	s__uncultured_Gemmatimonadetes_bacterium_g__norank_f__norank	OTU1459
d__Bacteria	k__norank_ d__Bacteria	p__Chloroflexi	c__Anaerolineae	o__SBR1031	f__norank_o__SBR1031	g__norank_f__norank_o__SBR1031	s__uncultured_Gemmatimonadetes_bacterium_g__norank_f__norank	OTU1456
d__Bacteria	k__norank_ d__Bacteria	p__Bacteroidota	c__Bacteroidia	o__Sphingobacteriales	f__Sphingobacteriaceae	g__Mucilaginibacter	s__unclassified_g__Mucilaginibacter	OTU2012
d__Bacteria	k__norank_ d__Bacteria	p__Acidobacteriota	c__Vicinamibacteria	o__Vicinamibacterales	f__norank_o__Vicinamibacterales	g__norank_f__norank_o__Vicinamiba cterales	s__unclassified_g__norank_f__norank_o__Vicinamibacterales	OTU2925
d__Bacteria	k__norank_ d__Bacteria	p__Bacteroidota	c__Bacteroidia	o__Chitinophagales	f__Chitinophagaceae	g__Aurantisolimonas	s__unclassified_g__Aurantisolimonas	OTU252
d__Bacteria	k__norank_ d__Bacteria	p__Chloroflexi	c__Anaerolineae	o__norank_c__Anaerolineae	f__norank_o__norank_c__Anaerolineae	g__norank_f__norank_o__norank_c__ Anaerolineae	s__uncultured_Bellilinea_sp._g__norank_f__norank	OTU1484

d__Bacteria	k__norank_ d__Bacteria	p__Proteobacteria	c__Alphaproteobacteria	o__Rhizobiales	f__Beijerinckiaceae	g__norank_f__Beijerinckiaceae	s__unclassified_g__norank_f__Beijerinckiaceae	OTU2906
d__Bacteria	k__norank_ d__Bacteria	p__Proteobacteria	c__Alphaproteobacteria	o__Caulobacterales	f__Caulobacteraceae	g__Brevundimonas	s__unclassified_g__Brevundimonas	OTU1474
d__Bacteria	k__norank_ d__Bacteria	p__Bacteroidota	c__Bacteroidia	o__Sphingobacteriales	f__AKYH767	g__norank_f__AKYH767	s__uncultured_bacterium_g__norank_f__AKYH767	OTU1275
d__Bacteria	k__norank_ d__Bacteria	p__unclassified_k__no rank_d__Bacteria	c__unclassified_k__noran k_d__Bacteria	o__unclassified_k__norank_ d__Bacteria	f__unclassified_k__norank_d__Bacteria	g__unclassified_k__norank_d__Bacteri a	s__unclassified_k__norank_d__Bacteria	OTU311
d__Bacteria	k__norank_ d__Bacteria	p__Bacteroidota	c__Bacteroidia	o__Chitinophagales	f__Saprospiraceae	g__norank_f__Saprospiraceae	s__uncultured_Devosia_sp._g__norank_f__Saprospiraceae	OTU2582
d__Bacteria	k__norank_ d__Bacteria	p__unclassified_k__no rank_d__Bacteria	c__unclassified_k__noran k_d__Bacteria	o__unclassified_k__norank_ d__Bacteria	f__unclassified_k__norank_d__Bacteria	g__unclassified_k__norank_d__Bacteri a	s__unclassified_k__norank_d__Bacteria	OTU316
d__Bacteria	k__norank_ d__Bacteria	p__Elusimicrobiota	c__Lineage_IIa	o__norank_c__Lineage_IIa	f__norank_o__norank_c__Lineage_IIa	g__norank_f__norank_o__norank_c__ Lineage_IIa	s__uncultured_bacterium_g__norank_f__norank_o__norank_c__Lineage_IIa	OTU1155
d__Bacteria	k__norank_ d__Bacteria	p__Proteobacteria	c__Gammaproteobacteria	o__Diplorickettsiales	f__Diplorickettsiaceae	g__norank_f__Diplorickettsiaceae	s__uncultured_Legionellales_bacterium_g__norank_f__Diplorickettsiaceae	OTU752
d__Bacteria	k__norank_ d__Bacteria	p__Proteobacteria	c__Gammaproteobacteria	o__Burkholderiales	f__Burkholderiaceae	g__Lautropia	s__metagenome_g__Lautropia	OTU2787
d__Bacteria	k__norank_ d__Bacteria	p__Bacteroidota	c__Bacteroidia	o__Chitinophagales	f__Chitinophagaceae	g__Ferruginibacter	s__uncultured_Bacteroidetes_bacterium_g__Ferruginibacter	OTU784
d__Bacteria	k__norank_ d__Bacteria	p__Proteobacteria	c__Alphaproteobacteria	o__Sphingomonadales	f__Sphingomonadaceae	g__Qipengyuania	s__unclassified_g__Qipengyuania	OTU520
d__Bacteria	k__norank_ d__Bacteria	p__Acidobacteriota	c__Blastocatellia	o__11-24	f__norank_o__11-24	g__norank_f__norank_o__11-24	s__uncultured_bacterium_g__norank_f__norank_o__11-24	OTU1513
d__Bacteria	k__norank_ d__Bacteria	p__Myxococcota	c__Polyangia	o__Haliangiales	f__Haliangiaceae	g__Haliangium	s__unclassified_g__Haliangium	OTU937
d__Bacteria	k__norank_ d__Bacteria	p__Proteobacteria	c__Gammaproteobacteria	o__Oceanospirillales	f__Pseudohongiellaceae	g__BIyi10	s__unclassified_g__BIyi10	OTU2126
d__Bacteria	k__norank_ d__Bacteria	p__Myxococcota	c__Polyangia	o__Haliangiales	f__Haliangiaceae	g__Haliangium	s__uncultured_bacterium_g__Haliangium	OTU1412
d__Bacteria	k__norank_ d__Bacteria	p__Proteobacteria	c__Alphaproteobacteria	o__Elsterales	f__norank_o__Elsterales	g__norank_f__norank_o__Elsterales	s__uncultured_bacterium_g__norank_f__norank_o__Elsterales	OTU2008

d__Bacteria	k__norank_ d__Bacteria	p__Proteobacteria	c__Gammaproteobacteria	o__Legionellales	f__Legionellaceae	g__Legionella	s__uncultured_bacterium_g__Legionella	OTU2091
d__Bacteria	k__norank_ d__Bacteria	p__Proteobacteria	c__Gammaproteobacteria	o__Salinisphaerales	f__Solimonadaceae	g__Polycyclovorans	s__unclassified_g__Polycyclovorans	OTU1171
d__Bacteria	k__norank_ d__Bacteria	p__Gemmatimonadota	c__Gemmatimonadetes	o__Gemmatimonadales	f__Gemmatimonadaceae	g__Gemmatimonas	s__unclassified_g__Gemmatimonas	OTU2391
d__Bacteria	k__norank_ d__Bacteria	p__Armatimonadota	c__Chthonomonadetes	o__Chthonomonadales	f__norank_o__Chthonomonadales	g__norank_f__norank_o__Chthonomo nadales	s__uncultured_bacterium_g__norank_f__norank_o__Chthonomonadales	OTU1704
d__Bacteria	k__norank_ d__Bacteria	p__Chloroflexi	c__Ktedonobacteria	o__C0119	f__norank_o__C0119	g__norank_f__norank_o__C0119	s__uncultured_bacterium_g__norank_f__norank_o__C0119	OTU1071
d__Bacteria	k__norank_ d__Bacteria	p__Actinobacteriota	c__Acidimicrobiia	o__Microtrichales	f__Ilumatobacteraceae	g__CL500-29_marine_group	s__metagenome_g__CL500-29_marine_group	OTU1464
d__Bacteria	k__norank_ d__Bacteria	p__Cyanobacteria	c__Vampirivibrionia	o__Obscuribacterales	f__Obscuribacteraceae	g__norank_f__Obscuribacteraceae	s__uncultured_bacterium_g__norank_f__Obscuribacteraceae	OTU10
d__Bacteria	k__norank_ d__Bacteria	p__Patescibacteria	c__Saccharimonadia	o__Saccharimonadales	f__norank_o__Saccharimonadales	g__norank_f__norank_o__Saccharimo nadales	s__uncultured_bacterium_g__norank_f__norank_o__Saccharimonadales	OTU1473
d__Bacteria	k__norank_ d__Bacteria	p__Acidobacteriota	c__Blastocatellia	o__Pyrinomonadales	f__Pyrinomonadaceae	g__RB41	s__unclassified_g__RB41	OTU1648
d__Bacteria	k__norank_ d__Bacteria	p__Acidobacteriota	c__Blastocatellia	o__Pyrinomonadales	f__Pyrinomonadaceae	g__RB41	s__unclassified_g__RB41	OTU1645
d__Bacteria	k__norank_ d__Bacteria	p__Actinobacteriota	c__Thermoleophilia	o__Solirubrobacterales	f__Solirubrobacteraceae	g__Conexibacter	s__uncultured_bacterium_g__Conexibacter	OTU1503
d__Bacteria	k__norank_ d__Bacteria	p__Planctomycetota	c__Planctomycetes	o__Isosphaerales	f__Isosphaeraceae	g__norank_f__Isosphaeraceae	s__uncultured_bacterium_g__norank_f__Isosphaeraceae	OTU2626
d__Bacteria	k__norank_ d__Bacteria	p__Chloroflexi	c__Ktedonobacteria	o__Ktedonobacterales	f__Ktedonobacteraceae	g__unclassified_f__Ktedonobacteracea e	s__unclassified_f__Ktedonobacteraceae	OTU1807
d__Bacteria	k__norank_ d__Bacteria	p__Myxococcota	c__Polyangia	o__Polyangiales	f__Sandaracinaceae	g__Sandaracinus	s__uncultured_bacterium_g__Sandaracinus	OTU546
d__Bacteria	k__norank_ d__Bacteria	p__Actinobacteriota	c__Thermoleophilia	o__Solirubrobacterales	f__67-14	g__norank_f__67-14	s__uncultured_Conexibacteraceae_bacterium_g__norank_f__67-14	OTU1308
d__Bacteria	k__norank_ d__Bacteria	p__Actinobacteriota	c__Acidimicrobiia	o__IMCC26256	f__norank_o__IMCC26256	g__norank_f__norank_o__IMCC26256	s__unclassified_g__norank_f__norank_o__IMCC26256	OTU1659

d__Bacteria	k__norank_ d__Bacteria	p__Patescibacteria	c__unclassified_p__Patescibacteria	o__unclassified_p__Patescibacteria	f__unclassified_p__Patescibacteria	g__unclassified_p__Patescibacteria	s__unclassified_p__Patescibacteria	OTU1974
d__Bacteria	k__norank_ d__Bacteria	p__Chloroflexi	c__Chloroflexia	o__Chloroflexales	f__Chloroflexaceae	g__FFCH7168	s__uncultured_bacterium_g__FFCH7168	OTU2840
d__Bacteria	k__norank_ d__Bacteria	p__Chloroflexi	c__Ktedonobacteria	o__Ktedonobacterales	f__Ktedonobacteraceae	g__unclassified_f__Ktedonobacteraceae	s__unclassified_f__Ktedonobacteraceae	OTU1574
d__Bacteria	k__norank_ d__Bacteria	p__Verrucomicrobiota	c__Verrucomicrobiae	o__Chthoniobacterales	f__Chthoniobacteraceae	g__Candidatus_Udaeobacter	s__unclassified_g__Candidatus_Udaeobacter	OTU1702
d__Bacteria	k__norank_ d__Bacteria	p__Bacteroidota	c__Bacteroidia	o__Cytophagales	f__Spirosomaceae	g__Spirosoma	s__uncultured_bacterium_g__Spirosoma	OTU2291
d__Bacteria	k__norank_ d__Bacteria	p__Actinobacteriota	c__Actinobacteria	o__Micrococcales	f__Microbacteriaceae	g__Subtercola	s__Subtercola_boreus	OTU1542
d__Bacteria	k__norank_ d__Bacteria	p__Myxococcota	c__Polyangia	o__Polyangiales	f__Polyangiaceae	g__Polyangium	s__metagenome_g__Polyangium	OTU1592
d__Bacteria	k__norank_ d__Bacteria	p__Myxococcota	c__Polyangia	o__mle1-27	f__norank_o__mle1-27	g__norank_f__norank_o__mle1-27	s__unclassified_g__norank_f__norank_o__mle1-27	OTU943
d__Bacteria	k__norank_ d__Bacteria	p__Bacteroidota	c__Bacteroidia	o__Chitinophagales	f__Chitinophagaceae	g__unclassified_f__Chitinophagaceae	s__unclassified_f__Chitinophagaceae	OTU2300
d__Bacteria	k__norank_ d__Bacteria	p__Acidobacteriota	c__Acidobacteriae	o__Subgroup_15	f__norank_o__Subgroup_15	g__norank_f__norank_o__Subgroup_15	s__uncultured_bacterium_g__norank_f__norank_o__Subgroup_15	OTU679
d__Bacteria	k__norank_ d__Bacteria	p__Chloroflexi	c__Ktedonobacteria	o__Ktedonobacterales	f__Ktedonobacteraceae	g__Ktedonobacter	s__uncultured_bacterium_g__Ktedonobacter	OTU34
d__Bacteria	k__norank_ d__Bacteria	p__Cyanobacteria	c__Cyanobacteriia	o__Chloroplast	f__norank_o__Chloroplast	g__norank_f__norank_o__Chloroplast	s__unclassified_g__norank_f__norank_o__Chloroplast	OTU135
d__Bacteria	k__norank_ d__Bacteria	p__Bacteroidota	c__Bacteroidia	o__Chitinophagales	f__Saprospiraceae	g__norank_f__Saprospiraceae	s__uncultured_bacterium_g__norank_f__Saprospiraceae	OTU369
d__Bacteria	k__norank_ d__Bacteria	p__Actinobacteriota	c__Actinobacteria	o__Corynebacteriales	f__Nocardiaceae	g__Smaragdicoccus	s__uncultured_bacterium_g__Smaragdicoccus	OTU457
d__Bacteria	k__norank_ d__Bacteria	p__Cyanobacteria	c__Cyanobacteriia	o__Chloroplast	f__norank_o__Chloroplast	g__norank_f__norank_o__Chloroplast	s__unclassified_g__norank_f__norank_o__Chloroplast	OTU138
d__Bacteria	k__norank_ d__Bacteria	p__Planctomycetota	c__Planctomycetes	o__Gemmatales	f__Gemmataceae	g__norank_f__Gemmataceae	s__unclassified_g__norank_f__Gemmataceae	OTU2621

d__Bacteria	k__norank_d__Bacteria	p__Bacteroidota	c__Bacteroidia	o__Sphingobacteriales	f__Sphingobacteriaceae	g__unclassified_f__Sphingobacteriaceae	s__unclassified_f__Sphingobacteriaceae	OTU2282
d__Bacteria	k__norank_d__Bacteria	p__Armatimonadota	c__norank_p__Armatimonadota	o__norank_c__norank_p__Armatimonadota	f__norank_o__norank_c__norank_p__Armatimonadota	g__norank_f__norank_o__norank_c__norank_p__Armatimonadota	s__uncultured_bacterium_g__norank_f__norank_o__norank_c__norank_p__Armatimonadota	OTU714
d__Bacteria	k__norank_d__Bacteria	p__Myxococcota	c__Myxococcia	o__Myxococcales	f__Myxococcaceae	g__Archangium	s__Archangium_gephyra	OTU446
d__Bacteria	k__norank_d__Bacteria	p__Acidobacteriota	c__Subgroup_22	o__norank_c__Subgroup_22	f__norank_o__norank_c__Subgroup_22	g__norank_f__norank_o__norank_c__Subgroup_22	s__unclassified_g__norank_f__norank_o__norank_c__Subgroup_22	OTU427
d__Bacteria	k__norank_d__Bacteria	p__Chloroflexi	c__Ktedonobacteria	o__C0119	f__norank_o__C0119	g__norank_f__norank_o__C0119	s__uncultured_bacterium_g__norank_f__norank_o__C0119	OTU1570
d__Bacteria	k__norank_d__Bacteria	p__Gemmatimonadota	c__Longimicrobia	o__Longimicrobiales	f__Longimicrobiaceae	g__norank_f__Longimicrobiaceae	s__uncultured_bacterium_g__norank_f__Longimicrobiaceae	OTU281
d__Bacteria	k__norank_d__Bacteria	p__Bacteroidota	c__Bacteroidia	o__Bacteroidales	f__Porphyromonadaceae	g__Porphyromonas	s__uncultured_bacterium_g__Porphyromonas	OTU404
d__Bacteria	k__norank_d__Bacteria	p__Elusimicrobiota	c__Lineage_IIa	o__norank_c__Lineage_IIa	f__norank_o__norank_c__Lineage_IIa	g__norank_f__norank_o__norank_c__Lineage_IIa	s__unclassified_g__norank_f__norank_o__norank_c__Lineage_IIa	OTU1745
d__Bacteria	k__norank_d__Bacteria	p__Patescibacteria	c__Saccharimonadia	o__Saccharimonadales	f__WWH38	g__norank_f__WWH38	s__uncultured_bacterium_g__norank_f__WWH38	OTU956
d__Bacteria	k__norank_d__Bacteria	p__Firmicutes	c__Bacilli	o__Paenibacillales	f__Paenibacillaceae	g__Paenibacillus	s__unclassified_g__Paenibacillus	OTU1187
d__Bacteria	k__norank_d__Bacteria	p__Chloroflexi	c__Chloroflexia	o__Thermomicrobiales	f__AKYG1722	g__norank_f__AKYG1722	s__uncultured_bacterium_g__norank_f__AKYG1722	OTU1612
d__Bacteria	k__norank_d__Bacteria	p__Actinobacteriota	c__Thermoleophilia	o__Gaiellales	f__norank_o__Gaiellales	g__norank_f__norank_o__Gaiellales	s__uncultured_soil_bacterium_g__norank_f__norank_o__Gaiellales	OTU1525
d__Bacteria	k__norank_d__Bacteria	p__Firmicutes	c__Bacilli	o__Entomoplasmatales	f__type_III	g__norank_f__type_III	s__uncultured_Mollicutes_bacterium_g__norank	OTU1381
d__Bacteria	k__norank_d__Bacteria	p__Chloroflexi	c__AD3	o__norank_c__AD3	f__norank_o__norank_c__AD3	g__norank_f__norank_o__norank_c__AD3	s__unclassified_g__norank_f__norank_o__norank_c__AD3	OTU1335
d__Bacteria	k__norank_d__Bacteria	p__unclassified_k__norank_d__Bacteria	c__unclassified_k__norank_d__Bacteria	o__unclassified_k__norank_d__Bacteria	f__unclassified_k__norank_d__Bacteria	g__unclassified_k__norank_d__Bacteria	s__unclassified_k__norank_d__Bacteria	OTU1062
d__Bacteria	k__norank_d__Bacteria	p__Proteobacteria	c__Gammaproteobacteria	o__unclassified_c__Gammaproteobacteria	f__unclassified_c__Gammaproteobacteria	g__unclassified_c__Gammaproteobacteria	s__unclassified_c__Gammaproteobacteria	OTU1280

d__Bacteria	k__norank_d__Bacteria	p__Armatimonadota	c__Armatimonadia	o__Armatimonadales	f__norank_o__Armatimonadales	g__norank_f__norank_o__Armatimonadales	s__uncultured_bacterium_g__norank_f__norank_o__Armatimonadales	OTU2199
d__Bacteria	k__norank_d__Bacteria	p__Chloroflexi	c__Ktedonobacteria	o__C0119	f__norank_o__C0119	g__norank_f__norank_o__C0119	s__uncultured_bacterium_g__norank_f__norank_o__C0119	OTU2470
d__Bacteria	k__norank_d__Bacteria	p__Proteobacteria	c__Gammaproteobacteria	o__Pseudomonadales	f__Moraxellaceae	g__Acinetobacter	s__Acinetobacter_johnsonii	OTU930
d__Bacteria	k__norank_d__Bacteria	p__Chloroflexi	c__Anaerolineae	o__Anaerolineales	f__Anaerolineaceae	g__norank_f__Anaerolineaceae	s__uncultured_Bellilinea_sp._g__norank_f__Anaerolineaceae	OTU2553
d__Bacteria	k__norank_d__Bacteria	p__Proteobacteria	c__Gammaproteobacteria	o__Diplorickettsiales	f__Diplorickettsiaceae	g__norank_f__Diplorickettsiaceae	s__unclassified_g__norank_f__Diplorickettsiaceae	OTU553
d__Bacteria	k__norank_d__Bacteria	p__Armatimonadota	c__Chthonomonadetes	o__Chthonomonadales	f__Chthonomonadaceae	g__Chthonomonas	s__uncultured_bacterium_g__Chthonomonas	OTU1855
d__Bacteria	k__norank_d__Bacteria	p__Bdellovibrionota	c__Oligoflexia	o__Oligoflexales	f__norank_o__Oligoflexales	g__Oligoflexus	s__Thymallus_thymallus_grayling_g__Oligoflexus	OTU177
d__Bacteria	k__norank_d__Bacteria	p__unclassified_k__norank_d__Bacteria	c__unclassified_k__norank_d__Bacteria	o__unclassified_k__norank_d__Bacteria	f__unclassified_k__norank_d__Bacteria	g__unclassified_k__norank_d__Bacteria	s__unclassified_k__norank_d__Bacteria	OTU901
d__Bacteria	k__norank_d__Bacteria	p__Actinobacteriota	c__Acidimicrobiia	o__Microtrichales	f__Microtrichaceae	g__IMCC26207	s__metagenome_g__IMCC26207	OTU2077
d__Bacteria	k__norank_d__Bacteria	p__Planctomycetota	c__Planctomycetes	o__Isosphaerales	f__Isosphaeraceae	g__Tundrisphaera	s__uncultured_bacterium_g__Tundrisphaera	OTU999
d__Bacteria	k__norank_d__Bacteria	p__Proteobacteria	c__Gammaproteobacteria	o__Burkholderiales	f__SC-I-84	g__norank_f__SC-I-84	s__uncultured_beta_proteobacterium_g__norank_f__SC-I-84	OTU2556
d__Bacteria	k__norank_d__Bacteria	p__Acidobacteriota	c__Blastocatellia	o__Blastocatellales	f__Blastocatellaceae	g__norank_f__Blastocatellaceae	s__uncultured_bacterium_g__norank_f__Blastocatellaceae	OTU2947
d__Bacteria	k__norank_d__Bacteria	p__Firmicutes	c__Bacilli	o__Lactobacillales	f__Aerococcaceae	g__Abiotrophia	s__uncultured_bacterium_g__Abiotrophia	OTU502
d__Bacteria	k__norank_d__Bacteria	p__Proteobacteria	c__Gammaproteobacteria	o__Steroidobacterales	f__Steroidobacteraceae	g__norank_f__Steroidobacteraceae	s__unclassified_g__norank_f__Steroidobacteraceae	OTU2446
d__Bacteria	k__norank_d__Bacteria	p__Proteobacteria	c__Alphaproteobacteria	o__Rhodobacterales	f__Rhodobacteraceae	g__Rubellimicrobium	s__uncultured_bacterium_g__Rubellimicrobium	OTU493
d__Bacteria	k__norank_d__Bacteria	p__Planctomycetota	c__Planctomycetes	o__Gemmatales	f__Gemmataceae	g__norank_f__Gemmataceae	s__uncultured_planctomycete_g__norank_f__Gemmataceae	OTU919

d__Bacteria	k__norank_d__Bacteria	p__Gemmatimonadota	c__Longimicrobia	o__Longimicrobiales	f__Longimicrobiaceae	g__norank_f__Longimicrobiaceae	s__metagenome_g__norank_f__Longimicrobiaceae	OTU2447
d__Bacteria	k__norank_d__Bacteria	p__Proteobacteria	c__Alphaproteobacteria	o__Tistrellales	f__Geminicoccaceae	g__Candidatus_Alysiosphaera	s__uncultured_Alphaproteobacteria_bacterium_g__Candidatus_Alysiosphaera	OTU487
d__Bacteria	k__norank_d__Bacteria	p__Firmicutes	c__Bacilli	o__Bacillales	f__Bacillaceae	g__Bacillus	s__Bacillus_anthracis_g__Bacillus	OTU593
d__Bacteria	k__norank_d__Bacteria	p__Gemmatimonadota	c__Gemmatimonadetes	o__Gemmatimonadales	f__Gemmatimonadaceae	g__unclassified_f__Gemmatimonadaceae	s__unclassified_f__Gemmatimonadaceae	OTU1006
d__Bacteria	k__norank_d__Bacteria	p__Chloroflexi	c__Ktedonobacteria	o__C0119	f__norank_o__C0119	g__norank_f__norank_o__C0119	s__uncultured_bacterium_g__norank_f__norank_o__C0119	OTU1977
d__Bacteria	k__norank_d__Bacteria	p__Proteobacteria	c__Alphaproteobacteria	o__Sphingomonadales	f__Sphingomonadaceae	g__Sphingobium	s__unclassified_g__Sphingobium	OTU2352
d__Bacteria	k__norank_d__Bacteria	p__Actinobacteriota	c__MB-A2-108	o__norank_c__MB-A2-108	f__norank_o__norank_c__MB-A2-108	g__norank_f__norank_o__norank_c__MB-A2-108	s__unclassified_g__norank_f__norank_o__norank_c__MB-A2-108	OTU1518
d__Bacteria	k__norank_d__Bacteria	p__Patescibacteria	c__Saccharimonadia	o__Saccharimonadales	f__norank_o__Saccharimonadales	g__norank_f__norank_o__Saccharimonadales	s__unclassified_g__norank_f__norank_o__Saccharimonadales	OTU1510
d__Bacteria	k__norank_d__Bacteria	p__Proteobacteria	c__Gammaproteobacteria	o__Coxiellales	f__Coxiellaceae	g__Coxiella	s__uncultured_bacterium_g__Coxiella	OTU1656
d__Bacteria	k__norank_d__Bacteria	p__Chloroflexi	c__Chloroflexia	o__Thermomicrobiales	f__JG30-KF-CM45	g__norank_f__JG30-KF-CM45	s__unclassified_g__norank_f__JG30-KF-CM45	OTU2753
d__Bacteria	k__norank_d__Bacteria	p__RCP2-54	c__norank_p__RCP2-54	o__norank_c__norank_p__RCP2-54	f__norank_o__norank_c__norank_p__RCP2-54	g__norank_f__norank_o__norank_c__norank_p__RCP2-54	s__unclassified_g__norank_f__norank_o__norank_c__norank_p__RCP2-54	OTU920
d__Bacteria	k__norank_d__Bacteria	p__Proteobacteria	c__Gammaproteobacteria	o__Xanthomonadales	f__Xanthomonadaceae	g__Silanimonas	s__uncultured_bacterium_g__Silanimonas	OTU2129
d__Bacteria	k__norank_d__Bacteria	p__Actinobacteriota	c__Actinobacteria	o__Frankiales	f__Acidothermaceae	g__Acidothermus	s__uncultured_bacterium_g__Acidothermus	OTU1758
d__Bacteria	k__norank_d__Bacteria	p__Chloroflexi	c__KD4-96	o__norank_c__KD4-96	f__norank_o__norank_c__KD4-96	g__norank_f__norank_o__norank_c__KD4-96	s__unclassified_g__norank_f__norank_o__norank_c__KD4-96	OTU2206
d__Bacteria	k__norank_d__Bacteria	p__Verrucomicrobiota	c__Chlamydiae	o__Chlamydiales	f__unclassified_o__Chlamydiales	g__unclassified_o__Chlamydiales	s__unclassified_o__Chlamydiales	OTU1148
d__Bacteria	k__norank_d__Bacteria	p__Acidobacteriota	c__Blastocatellia	o__Pyrinomonadales	f__Pyrinomonadaceae	g__RB41	s__uncultured_Acidobacteria_bacterium_g__RB41	OTU829

d__Bacteria	k__norank_ d__Bacteria	p__Verrucomicrobiota	c__Verrucomicrobiae	o__Chthoniobacterales	f__Chthoniobacteraceae	g__Candidatus_Udaeobacter	s__unclassified_g__Candidatus_Udaeobacter	OTU697
d__Bacteria	k__norank_ d__Bacteria	p__Proteobacteria	c__Gammaproteobacteria	o__R7C24	f__norank_o__R7C24	g__norank_f__norank_o__R7C24	s__metagenome_g__norank_f__norank_o__R7C24	OTU2292
d__Bacteria	k__norank_ d__Bacteria	p__Proteobacteria	c__Alphaproteobacteria	o__Caulobacterales	f__Caulobacteraceae	g__Asticcacaulis	s__uncultured_bacterium_g__Asticcacaulis	OTU242
d__Bacteria	k__norank_ d__Bacteria	p__Proteobacteria	c__Alphaproteobacteria	o__Rhodospirillales	f__norank_o__Rhodospirillales	g__norank_f__norank_o__Rhodospirill ales	s__unclassified_g__norank_f__norank_o__Rhodospirillales	OTU286
d__Bacteria	k__norank_ d__Bacteria	p__Acidobacteriota	c__unclassified_p__Acido bacteriota	o__unclassified_p__Acidoba cteriota	f__unclassified_p__Acidobacteriota	g__unclassified_p__Acidobacteriota	s__unclassified_p__Acidobacteriota	OTU1352
d__Bacteria	k__norank_ d__Bacteria	p__Proteobacteria	c__Gammaproteobacteria	o__Diplorickettsiales	f__Diplorickettsiaceae	g__norank_f__Diplorickettsiaceae	s__metagenome_g__norank_f__Diplorickettsiaceae	OTU2445
d__Bacteria	k__norank_ d__Bacteria	p__Myxococcota	c__Polyangia	o__Polyangiales	f__Phaselicystidaceae	g__Phaselicystis	s__unclassified_g__Phaselicystis	OTU1991
d__Bacteria	k__norank_ d__Bacteria	p__Actinobacteriota	c__Actinobacteria	o__Micrococcales	f__Microbacteriaceae	g__unclassified_f__Microbacteriaceae	s__unclassified_f__Microbacteriaceae	OTU2730
d__Bacteria	k__norank_ d__Bacteria	p__Proteobacteria	c__Alphaproteobacteria	o__Reyranellales	f__Reyranellaceae	g__Reyranella	s__metagenome_g__Reyranella	OTU1191
d__Bacteria	k__norank_ d__Bacteria	p__Patescibacteria	c__Saccharimonadia	o__Saccharimonadales	f__unclassified_o__Saccharimonadales	g__unclassified_o__Saccharimonadale s	s__unclassified_o__Saccharimonadales	OTU1526
d__Bacteria	k__norank_ d__Bacteria	p__Myxococcota	c__Polyangia	o__Haliangiales	f__Haliangiaceae	g__Haliangium	s__unclassified_g__Haliangium	OTU2756
d__Bacteria	k__norank_ d__Bacteria	p__Proteobacteria	c__Gammaproteobacteria	o__Burkholderiales	f__SC-I-84	g__norank_f__SC-I-84	s__uncultured_bacterium_g__norank_f__SC-I-84	OTU2433
d__Bacteria	k__norank_ d__Bacteria	p__Armatimonadota	c__Chthonomonadetes	o__Chthonomonadales	f__Chthonomonadaceae	g__Chthonomonas	s__uncultured_Armatimonadetes_bacterium_g__Chthonomonas	OTU776
d__Bacteria	k__norank_ d__Bacteria	p__Bacteroidota	c__Bacteroidia	o__Chitinophagales	f__norank_o__Chitinophagales	g__norank_f__norank_o__Chitinophag ales	s__uncultured_bacterium_g__norank_f__norank_o__Chitinophagales	OTU289
d__Bacteria	k__norank_ d__Bacteria	p__Bacteroidota	c__Rhodothermia	o__Rhodothermales	f__Rhodothermaceae	g__norank_f__Rhodothermaceae	s__uncultured_bacterium_g__norank_f__Rhodothermaceae	OTU2875
d__Bacteria	k__norank_ d__Bacteria	p__Bacteroidota	c__Bacteroidia	o__Cytophagales	f__Spirosomaceae	g__Dyadobacter	s__unclassified_g__Dyadobacter	OTU307

d__Bacteria	k__norank_d__Bacteria	p__Myxococcota	c__Polyangia	o__mle1-27	f__norank_o__mle1-27	g__norank_f__norank_o__mle1-27	s__unclassified_g__norank_f__norank_o__mle1-27	OTU1822
d__Bacteria	k__norank_d__Bacteria	p__Armatimonadota	c__Chthonomonadetes	o__Chthonomonadales	f__Chthonomonadaceae	g__Chthonomonas	s__uncultured_bacterium_g__Chthonomonas	OTU1033
d__Bacteria	k__norank_d__Bacteria	p__Armatimonadota	c__Chthonomonadetes	o__Chthonomonadales	f__Chthonomonadaceae	g__Chthonomonas	s__uncultured_bacterium_g__Chthonomonas	OTU1035
d__Bacteria	k__norank_d__Bacteria	p__Proteobacteria	c__Alphaproteobacteria	o__Caulobacterales	f__Caulobacteraceae	g__Phenylobacterium	s__metagenome_g__Phenylobacterium	OTU2319
d__Bacteria	k__norank_d__Bacteria	p__Bacteroidota	c__Bacteroidia	o__Flavobacteriales	f__Flavobacteriaceae	g__Flavobacterium	s__unclassified_g__Flavobacterium	OTU1297
d__Bacteria	k__norank_d__Bacteria	p__Proteobacteria	c__Alphaproteobacteria	o__Sphingomonadales	f__Sphingomonadaceae	g__Altererythrobacter	s__uncultured_bacterium_g__Altererythrobacter	OTU2829
d__Bacteria	k__norank_d__Bacteria	p__Cyanobacteria	c__Cyanobacteriia	o__Chloroplast	f__norank_o__Chloroplast	g__norank_f__norank_o__Chloroplast	s__uncultured_Chroococcidiopsis_sp._g__norank	OTU2836
d__Bacteria	k__norank_d__Bacteria	p__Patescibacteria	c__Saccharimonadia	o__Saccharimonadales	f__norank_o__Saccharimonadales	g__norank_f__norank_o__Saccharimonadales	s__uncultured_bacterium_g__norank_f__norank_o__Saccharimonadales	OTU2888
d__Bacteria	k__norank_d__Bacteria	p__Gemmatimonadota	c__Gemmatimonadetes	o__Gemmatimonadales	f__Gemmatimonadaceae	g__Roseisolibacter	s__uncultured_bacterium_g__Roseisolibacter	OTU1151
d__Bacteria	k__norank_d__Bacteria	p__Bdellovibrionota	c__Oligoflexia	o__Oligoflexales	f__norank_o__Oligoflexales	g__Oligoflexus	s__metagenome_g__Oligoflexus	OTU243
d__Bacteria	k__norank_d__Bacteria	p__Cyanobacteria	c__Cyanobacteriia	o__Chloroplast	f__norank_o__Chloroplast	g__norank_f__norank_o__Chloroplast	s__unclassified_g__norank_f__norank_o__Chloroplast	OTU2339
d__Bacteria	k__norank_d__Bacteria	p__Chloroflexi	c__Anaerolineae	o__Caldilineales	f__Caldilineaceae	g__norank_f__Caldilineaceae	s__unclassified_g__norank_f__Caldilineaceae	OTU2820
d__Bacteria	k__norank_d__Bacteria	p__Acidobacteriota	c__Acidobacteriae	o__Solibacterales	f__Solibacteraceae	g__Candidatus_Solibacter	s__uncultured_forest_soil_bacterium_g__Candidatus_Solibacter	OTU1859
d__Bacteria	k__norank_d__Bacteria	p__Armatimonadota	c__Armatimonadia	o__Armatimonadales	f__norank_o__Armatimonadales	g__norank_f__norank_o__Armatimonadales	s__uncultured_bacterium_g__norank_f__norank_o__Armatimonadales	OTU1997
d__Bacteria	k__norank_d__Bacteria	p__Campilobacterota	c__Campylobacteria	o__Campylobacterales	f__Arcobacteraceae	g__unclassified_f__Arcobacteraceae	s__unclassified_f__Arcobacteraceae	OTU396
d__Bacteria	k__norank_d__Bacteria	p__Proteobacteria	c__Alphaproteobacteria	o__Defluviicoccales	f__Defluviicoccaceae	g__Defluviicoccus	s__metagenome_g__Defluviicoccus	OTU2249

d__Bacteria	k__norank_ d__Bacteria	p__Myxococcota	c__Myxococcia	o__Myxococcales	f__Myxococcaceae	g__P3OB-42	s__unclassified_g__P3OB-42	OTU2060
d__Bacteria	k__norank_ d__Bacteria	p__Acidobacteriota	c__Holophagae	o__Subgroup_7	f__norank_o__Subgroup_7	g__norank_f__norank_o__Subgroup_7	s__uncultured_bacterium_g__norank_f__norank_o__Subgroup_7	OTU2523
d__Bacteria	k__norank_ d__Bacteria	p__Proteobacteria	c__Gammaproteobacteria	o__unclassified_c__Gamma proteobacteria	f__unclassified_c__Gammaproteobacteri a	g__unclassified_c__Gammaproteobact eria	s__unclassified_c__Gammaproteobacteria	OTU2735
d__Bacteria	k__norank_ d__Bacteria	p__Latescibacterota	c__norank_p__Latescibact erota	o__norank_c__norank_p__L atescibacterota	f__norank_o__norank_c__norank_p__La tescibacterota	g__norank_f__norank_o__norank_c__ norank_p__Latescibacterota	s__metagenome_g__norank_f__norank_o__norank_c__norank_p__Latescibacterota	OTU426
d__Bacteria	k__norank_ d__Bacteria	p__Bacteroidota	c__Bacteroidia	o__Sphingobacteriales	f__Sphingobacteriaceae	g__Mucilaginibacter	s__unclassified_g__Mucilaginibacter	OTU757
d__Bacteria	k__norank_ d__Bacteria	p__Proteobacteria	c__Gammaproteobacteria	o__Pseudomonadales	f__Moraxellaceae	g__Psychrobacter	s__Psychrobacter_arcticus_273-4	OTU539
d__Bacteria	k__norank_ d__Bacteria	p__Proteobacteria	c__Gammaproteobacteria	o__Burkholderiales	f__Burkholderiaceae	g__Lautropia	s__metagenome_g__Lautropia	OTU348
d__Bacteria	k__norank_ d__Bacteria	p__unclassified_k__no rank_d__Bacteria	c__unclassified_k__noran k_d__Bacteria	o__unclassified_k__norank_ d__Bacteria	f__unclassified_k__norank_d__Bacteria	g__unclassified_k__norank_d__Bacteri a	s__unclassified_k__norank_d__Bacteria	OTU1591
d__Bacteria	k__norank_ d__Bacteria	p__Chloroflexi	c__Anaerolineae	o__RBG-13-54-9	f__norank_o__RBG-13-54-9	g__norank_f__norank_o__RBG-13-54- 9	s__uncultured_sludge_bacterium_A31	OTU2104
d__Bacteria	k__norank_ d__Bacteria	p__Bdellovibrionota	c__Oligoflexia	o__0319-6G20	f__norank_o__0319-6G20	g__norank_f__norank_o__0319-6G20	s__marine_metagenome_g__norank_f__norank_o__0319-6G20	OTU41
d__Bacteria	k__norank_ d__Bacteria	p__Proteobacteria	c__Gammaproteobacteria	o__Diplorickettsiales	f__Diplorickettsiaceae	g__norank_f__Diplorickettsiaceae	s__unclassified_g__norank_f__Diplorickettsiaceae	OTU1138
d__Bacteria	k__norank_ d__Bacteria	p__Bacteroidota	c__Bacteroidia	o__Sphingobacteriales	f__AKYH767	g__norank_f__AKYH767	s__uncultured_Bacteroidetes_bacterium_g__norank_f__AKYH767	OTU1089
d__Bacteria	k__norank_ d__Bacteria	p__Bacteroidota	c__Bacteroidia	o__Flavobacteriales	f__Flavobacteriaceae	g__Flavobacterium	s__unclassified_g__Flavobacterium	OTU2590
d__Bacteria	k__norank_ d__Bacteria	p__Armatimonadota	c__norank_p__Armatimon adota	o__norank_c__norank_p__ Armatimonadota	f__norank_o__norank_c__norank_p__Ar matimonadota	g__norank_f__norank_o__norank_c__ norank_p__Armatimonadota	s__unclassified_g__norank_f__norank_o__norank_c__norank_p__Armatimonadota	OTU942
d__Bacteria	k__norank_ d__Bacteria	p__Actinobacteriota	c__Acidimicrobiia	o__norank_c__Acidimicrobi ia	f__norank_o__norank_c__Acidimicrobiia	g__norank_f__norank_o__norank_c__ Acidimicrobiia	s__uncultured_forest_soil_bacterium_g__norank_f__norank_o__norank_c__Acidim icrobiia	OTU1945
d__Bacteria	k__norank_ d__Bacteria	p__Acidobacteriota	c__Vicinamibacteria	o__Vicinamibacterales	f__Vicinamibacteraceae	g__norank_f__Vicinamibacteraceae	s__uncultured_Acidobacteriaceae_bacterium_g__norank_f__Vicinamibacteraceae	OTU1409

d__Bacteria	k__norank_ d__Bacteria	p__Bacteroidota	c__Bacteroidia	o__Chitinophagales	f__Chitinophagaceae	g__Flavisolibacter	s__unclassified_g__Flavisolibacter	OTU1173
d__Bacteria	k__norank_ d__Bacteria	p__Patescibacteria	c__Parcubacteria	o__Candidatus_Kaiserbacter ia	f__norank_o__Candidatus_Kaiserbacteria	g__norank_f__norank_o__Candidatus_ Kaiserbacteria	s__unclassified_g__norank_f__norank_o__Candidatus_Kaiserbacteria	OTU147
d__Bacteria	k__norank_ d__Bacteria	p__Proteobacteria	c__Gammaproteobacteria	o__Burkholderiales	f__SC-I-84	g__norank_f__SC-I-84	s__uncultured_Burkholderiales_bacterium_g__norank_f__SC-I-84	OTU1565
d__Bacteria	k__norank_ d__Bacteria	p__Dependentiae	c__Babeliae	o__Babeliales	f__Vermiphilaceae	g__norank_f__Vermiphilaceae	s__uncultured_bacterium_g__norank_f__Vermiphilaceae	OTU2082
d__Bacteria	k__norank_ d__Bacteria	p__Chloroflexi	c__Ktedonobacteria	o__Ktedonobacterales	f__JG30-KF-AS9	g__norank_f__JG30-KF-AS9	s__uncultured_bacterium_g__norank_f__JG30-KF-AS9	OTU1841
d__Bacteria	k__norank_ d__Bacteria	p__Myxococcota	c__Polyangia	o__Haliangiales	f__Haliangiaceae	g__Haliangium	s__metagenome_g__Haliangium	OTU1103
d__Bacteria	k__norank_ d__Bacteria	p__Chloroflexi	c__Ktedonobacteria	o__C0119	f__norank_o__C0119	g__norank_f__norank_o__C0119	s__unclassified_g__norank_f__norank_o__C0119	OTU1521
d__Bacteria	k__norank_ d__Bacteria	p__Proteobacteria	c__Alphaproteobacteria	o__Elsterales	f__norank_o__Elsterales	g__norank_f__norank_o__Elsterales	s__uncultured_bacterium_g__norank_f__norank_o__Elsterales	OTU1664
d__Bacteria	k__norank_ d__Bacteria	p__Acidobacteriota	c__Acidobacteriae	o__Acidobacteriales	f__unclassified_o__Acidobacteriales	g__unclassified_o__Acidobacteriales	s__unclassified_o__Acidobacteriales	OTU1965
d__Bacteria	k__norank_ d__Bacteria	p__Myxococcota	c__Polyangia	o__Polyangiales	f__Sandaracinaceae	g__norank_f__Sandaracinaceae	s__uncultured_bacterium_g__norank_f__Sandaracinaceae	OTU2152
d__Bacteria	k__norank_ d__Bacteria	p__Chloroflexi	c__Ktedonobacteria	o__Ktedonobacterales	f__Ktedonobacteraceae	g__HSB_OF53-F07	s__uncultured_Ktedonobacter_sp._g__HSB_OF53-F07	OTU1651
d__Bacteria	k__norank_ d__Bacteria	p__Proteobacteria	c__Gammaproteobacteria	o__Diplorickettsiales	f__Diplorickettsiaceae	g__norank_f__Diplorickettsiaceae	s__unclassified_g__norank_f__Diplorickettsiaceae	OTU701
d__Bacteria	k__norank_ d__Bacteria	p__Acidobacteriota	c__Acidobacteriae	o__Subgroup_2	f__norank_o__Subgroup_2	g__norank_f__norank_o__Subgroup_2	s__uncultured_forest_soil_bacterium_g__norank_f__norank_o__Subgroup_2	OTU1842
d__Bacteria	k__norank_ d__Bacteria	p__Bacteroidota	c__Bacteroidia	o__Cytophagales	f__Microscillaceae	g__Ohtaekwangia	s__metagenome_g__Ohtaekwangia	OTU2628
d__Bacteria	k__norank_ d__Bacteria	p__Deinococcota	c__Deinococci	o__Deinococcales	f__Deinococcaceae	g__Deinococcus	s__unclassified_g__Deinococcus	OTU126
d__Bacteria	k__norank_ d__Bacteria	p__Actinobacteriota	c__Actinobacteria	o__Frankiales	f__Acidothermaceae	g__Acidothermus	s__uncultured_Thermomonosporaceae_bacterium	OTU2614

d__Bacteria	k__norank_ d__Bacteria	p__Verrucomicrobiota	c__Verrucomicrobiae	o__Pedosphaerales	f__Pedosphaeraceae	g__norank_f__Pedosphaeraceae	s__uncultured_soil_bacterium_g__norank_f__Pedosphaeraceae	OTU759
d__Bacteria	k__norank_ d__Bacteria	p__Planctomycetota	c__Planctomycetes	o__Gemmatales	f__Gemmataceae	g__norank_f__Gemmataceae	s__uncultured_bacterium_g__norank_f__Gemmataceae	OTU1866
d__Bacteria	k__norank_ d__Bacteria	p__Actinobacteriota	c__Actinobacteria	o__Frankiales	f__norank_o__Frankiales	g__norank_f__norank_o__Frankiales	s__uncultured_actinobacterium_g__norank_f__norank_o__Frankiales	OTU1176
d__Bacteria	k__norank_ d__Bacteria	p__Chloroflexi	c__Anaerolineae	o__Caldilineales	f__Caldilineaceae	g__norank_f__Caldilineaceae	s__uncultured_bacterium_g__norank_f__Caldilineaceae	OTU949
d__Bacteria	k__norank_ d__Bacteria	p__Armatimonadota	c__Chthonomonadetes	o__Chthonomonadales	f__norank_o__Chthonomonadales	g__norank_f__norank_o__Chthonomo nadales	s__uncultured_bacterium_g__norank_f__norank_o__Chthonomonadales	OTU1067
d__Bacteria	k__norank_ d__Bacteria	p__Firmicutes	c__Clostridia	o__Clostridiales	f__Clostridiaceae	g__Clostridium_sensu_stricto_1	s__Clostridium_beijerinckii	OTU2622
d__Bacteria	k__norank_ d__Bacteria	p__Bacteroidota	c__Bacteroidia	o__Cytophagales	f__Spirosomaceae	g__Dyadobacter	s__uncultured_bacterium_g__Dyadobacter	OTU2330
d__Bacteria	k__norank_ d__Bacteria	p__Actinobacteriota	c__Thermoleophilia	o__Gaiellales	f__norank_o__Gaiellales	g__norank_f__norank_o__Gaiellales	s__unclassified_g__norank_f__norank_o__Gaiellales	OTU2560
d__Bacteria	k__norank_ d__Bacteria	p__Chloroflexi	c__Ktedonobacteria	o__Ktedonobacterales	f__Ktedonobacteraceae	g__unclassified_f__Ktedonobacteracea e	s__unclassified_f__Ktedonobacteraceae	OTU1707
d__Bacteria	k__norank_ d__Bacteria	p__Nitrospirota	c__Nitrospiria	o__Nitrospirales	f__Nitrospiraceae	g__Nitrospira	s__uncultured_organism_g__Nitrospira	OTU1502
d__Bacteria	k__norank_ d__Bacteria	p__Planctomycetota	c__Planctomycetes	o__Gemmatales	f__Gemmataceae	g__norank_f__Gemmataceae	s__unclassified_g__norank_f__Gemmataceae	OTU1861
d__Bacteria	k__norank_ d__Bacteria	p__Patescibacteria	c__Saccharimonadia	o__Saccharimonadales	f__LWQ8	g__norank_f__LWQ8	s__uncultured_bacterium_g__norank_f__LWQ8	OTU1453
d__Bacteria	k__norank_ d__Bacteria	p__Actinobacteriota	c__Acidimicrobiia	o__Microtrichales	f__Ilumatobacteraceae	g__norank_f__Ilumatobacteraceae	s__unclassified_g__norank_f__Ilumatobacteraceae	OTU1097
d__Bacteria	k__norank_ d__Bacteria	p__Acidobacteriota	c__Vicinamibacteria	o__Vicinamibacterales	f__norank_o__Vicinamibacterales	g__norank_f__norank_o__Vicinamiba cterales	s__uncultured_bacterium_g__norank_f__norank_o__Vicinamibacterales	OTU185
d__Bacteria	k__norank_ d__Bacteria	p__Acidobacteriota	c__Blastocatellia	o__Blastocatellales	f__Blastocatellaceae	g__norank_f__Blastocatellaceae	s__uncultured_bacterium_g__norank_f__Blastocatellaceae	OTU832
d__Bacteria	k__norank_ d__Bacteria	p__Cyanobacteria	c__Cyanobacteriia	o__Pseudanabaenales	f__Pseudanabaenaceae	g__Synechococcus_PCC-7502	s__uncultured_Antarctic_cyanobacterium_g__Synechococcus_PCC-7502	OTU791

d__Bacteria	k__norank_ d__Bacteria	p__Cyanobacteria	c__Vampirivibrionia	o__Obscuribacterales	f__Obscuribacteraceae	g__norank_f__Obscuribacteraceae	s__uncultured_bacterium_g__norank_f__Obscuribacteraceae	OTU1059
d__Bacteria	k__norank_ d__Bacteria	p__Bacteroidota	c__Bacteroidia	o__Cytophagales	f__Spirosomaceae	g__Spirosoma	s__Spirosoma_endophyticum	OTU2284
d__Bacteria	k__norank_ d__Bacteria	p__Acidobacteriota	c__Blastocatellia	o__11-24	f__norank_o__11-24	g__norank_f__norank_o__11-24	s__uncultured_bacterium_g__norank_f__norank_o__11-24	OTU580
d__Bacteria	k__norank_ d__Bacteria	p__Patescibacteria	c__Saccharimonadia	o__Saccharimonadales	f__norank_o__Saccharimonadales	g__norank_f__norank_o__Saccharimo nadales	s__unclassified_g__norank_f__norank_o__Saccharimonadales	OTU2988
d__Bacteria	k__norank_ d__Bacteria	p__Actinobacteriota	c__Actinobacteria	o__Propionibacteriales	f__Nocardioidaceae	g__Nocardioides	s__metagenome_g__Nocardioides	OTU1537
d__Bacteria	k__norank_ d__Bacteria	p__Actinobacteriota	c__Actinobacteria	o__Propionibacteriales	f__Propionibacteriaceae	g__Friedmanniella	s__unclassified_g__Friedmanniella	OTU2716
d__Bacteria	k__norank_ d__Bacteria	p__Verrucomicrobiota	c__Verrucomicrobiae	o__Pedosphaerales	f__Pedosphaeraceae	g__norank_f__Pedosphaeraceae	s__uncultured_soil_bacterium_g__norank_f__Pedosphaeraceae	OTU648
d__Bacteria	k__norank_ d__Bacteria	p__Planctomycetota	c__BD7-11	o__norank_c__BD7-11	f__norank_o__norank_c__BD7-11	g__norank_f__norank_o__norank_c__ BD7-11	s__uncultured_bacterium_g__norank_f__norank_o__norank_c__BD7-11	OTU837
d__Bacteria	k__norank_ d__Bacteria	p__Bacteroidota	c__Kryptonia	o__Kryptoniales	f__BSV26	g__norank_f__BSV26	s__uncultured_soil_bacterium_g__norank_f__BSV26	OTU395
d__Bacteria	k__norank_ d__Bacteria	p__Actinobacteriota	c__Thermoleophilia	o__Gaiellales	f__norank_o__Gaiellales	g__norank_f__norank_o__Gaiellales	s__uncultured_Solirubrobacter_sp._g__norank	OTU1361
d__Bacteria	k__norank_ d__Bacteria	p__Bacteroidota	c__Bacteroidia	o__Chitinophagales	f__Chitinophagaceae	g__norank_f__Chitinophagaceae	s__unclassified_g__norank_f__Chitinophagaceae	OTU785
d__Bacteria	k__norank_ d__Bacteria	p__Proteobacteria	c__Alphaproteobacteria	o__Rhizobiales	f__Rhodomicrobiaceae	g__Rhodomicrobium	s__uncultured_Rhodomicrobium_sp._g__Rhodomicrobium	OTU1332
d__Bacteria	k__norank_ d__Bacteria	p__Chloroflexi	c__Chloroflexia	o__Chloroflexales	f__Roseiflexaceae	g__norank_f__Roseiflexaceae	s__unclassified_g__norank_f__Roseiflexaceae	OTU2316
d__Bacteria	k__norank_ d__Bacteria	p__Dependentiae	c__Babeliae	o__Babeliales	f__Vermiphilaceae	g__norank_f__Vermiphilaceae	s__uncultured_bacterium_g__norank_f__Vermiphilaceae	OTU1281
d__Bacteria	k__norank_ d__Bacteria	p__Armatimonadota	c__Chthonomonadetes	o__Chthonomonadales	f__norank_o__Chthonomonadales	g__norank_f__norank_o__Chthonomo nadales	s__uncultured_bacterium_g__norank_f__norank_o__Chthonomonadales	OTU1202
d__Bacteria	k__norank_ d__Bacteria	p__Proteobacteria	c__Gammaproteobacteria	o__Diplorickettsiales	f__Diplorickettsiaceae	g__Aquicella	s__uncultured_bacterium_g__Aquicella	OTU903

d__Bacteria	k__norank_d__Bacteria	p__Abditibacteriota	c__Abditibacteria	o__Abditibacteriales	f__Abditibacteriaceae	g__Abditibacterium	s__uncultured_endolithic_bacterium_g__Abditibacterium	OTU2849
d__Bacteria	k__norank_d__Bacteria	p__Actinobacteriota	c__MB-A2-108	o__norank_c__MB-A2-108	f__norank_o__norank_c__MB-A2-108	g__norank_f__norank_o__norank_c__MB-A2-108	s__uncultured_bacterium_g__norank_f__norank_o__norank_c__MB-A2-108	OTU1418
d__Bacteria	k__norank_d__Bacteria	p__Patescibacteria	c__Saccharimonadia	o__Saccharimonadales	f__norank_o__Saccharimonadales	g__norank_f__norank_o__Saccharimonadales	s__unclassified_g__norank_f__norank_o__Saccharimonadales	OTU1050
d__Bacteria	k__norank_d__Bacteria	p__Chloroflexi	c__TK10	o__norank_c__TK10	f__norank_o__norank_c__TK10	g__norank_f__norank_o__norank_c__TK10	s__unclassified_g__norank_f__norank_o__norank_c__TK10	OTU1087
d__Bacteria	k__norank_d__Bacteria	p__Bacteroidota	c__Bacteroidia	o__Chitinophagales	f__Chitinophagaceae	g__Segetibacter	s__uncultured_bacterium_g__Segetibacter	OTU93
d__Bacteria	k__norank_d__Bacteria	p__Proteobacteria	c__Alphaproteobacteria	o__Rhizobiales	f__Rhizobiaceae	g__Allorhizobium-Neorhizobium-Pararhizobium-Rhizobium	s__unclassified_g__Allorhizobium-Neorhizobium-Pararhizobium-Rhizobium	OTU2222
d__Bacteria	k__norank_d__Bacteria	p__Acidobacteriota	c__Holophagae	o__Subgroup_7	f__norank_o__Subgroup_7	g__norank_f__norank_o__Subgroup_7	s__uncultured_proteobacterium_g__norank_f__norank_o__Subgroup_7	OTU1468
d__Bacteria	k__norank_d__Bacteria	p__Patescibacteria	c__Saccharimonadia	o__Saccharimonadales	f__LWQ8	g__norank_f__LWQ8	s__uncultured_Candidatus_Saccharibacteria_bacterium_g__norank_f__LWQ8	OTU218
d__Bacteria	k__norank_d__Bacteria	p__Planctomycetota	c__Planctomycetes	o__Gemmatales	f__Gemmataceae	g__norank_f__Gemmataceae	s__unclassified_g__norank_f__Gemmataceae	OTU795
d__Bacteria	k__norank_d__Bacteria	p__Bacteroidota	c__Bacteroidia	o__Cytophagales	f__Hymenobacteraceae	g__Hymenobacter	s__unclassified_g__Hymenobacter	OTU82
d__Bacteria	k__norank_d__Bacteria	p__Proteobacteria	c__Alphaproteobacteria	o__Rickettsiales	f__Rickettsiaceae	g__unclassified_f__Rickettsiaceae	s__unclassified_f__Rickettsiaceae	OTU747
d__Bacteria	k__norank_d__Bacteria	p__Actinobacteriota	c__Actinobacteria	o__Propionibacteriales	f__Propionibacteriaceae	g__Tessaracoccus	s__uncultured_bacterium_g__Tessaracoccus	OTU2797
d__Bacteria	k__norank_d__Bacteria	p__Bdellovibrionota	c__Bdellovibrionia	o__Bdellovibrionales	f__Bdellovibrionaceae	g__Bdellovibrio	s__uncultured_bacterium_g__Bdellovibrio	OTU64
d__Bacteria	k__norank_d__Bacteria	p__Bacteroidota	c__Bacteroidia	o__Sphingobacteriales	f__Sphingobacteriaceae	g__Pedobacter	s__unclassified_g__Pedobacter	OTU2524
d__Bacteria	k__norank_d__Bacteria	p__Patescibacteria	c__Saccharimonadia	o__Saccharimonadales	f__norank_o__Saccharimonadales	g__norank_f__norank_o__Saccharimonadales	s__uncultured_bacterium_g__norank_f__norank_o__Saccharimonadales	OTU1195
d__Bacteria	k__norank_d__Bacteria	p__Verrucomicrobiota	c__Chlamydiae	o__Chlamydiales	f__Parachlamydiaceae	g__Candidatus_Protochlamydia	s__unclassified_g__Candidatus_Protochlamydia	OTU940

d__Bacteria	k__norank_ d__Bacteria	p__Acidobacteriota	c__Vicinamibacteria	o__Vicinamibacterales	f__norank_o__Vicinamibacterales	g__norank_f__norank_o__Vicinamiba cterales	s__unclassified_g__norank_f__norank_o__Vicinamibacterales	OTU863
d__Bacteria	k__norank_ d__Bacteria	p__Chloroflexi	c__Ktedonobacteria	o__Ktedonobacterales	f__Ktedonobacteraceae	g__norank_f__Ktedonobacteraceae	s__uncultured_bacterium_g__norank_f__Ktedonobacteraceae	OTU1920
d__Bacteria	k__norank_ d__Bacteria	p__Acidobacteriota	c__Acidobacteriae	o__Bryobacterales	f__Bryobacteraceae	g__Bryobacter	s__unclassified_g__Bryobacter	OTU1193
d__Bacteria	k__norank_ d__Bacteria	p__Acidobacteriota	c__Acidobacteriae	o__Acidobacteriales	f__norank_o__Acidobacteriales	g__norank_f__norank_o__Acidobacter iales	s__uncultured_Acidobacteriaceae_bacterium_g__norank_f__norank	OTU1078
d__Bacteria	k__norank_ d__Bacteria	p__Proteobacteria	c__Gammaproteobacteria	o__Burkholderiales	f__Comamonadaceae	g__Acidovorax	s__unclassified_g__Acidovorax	OTU95
d__Bacteria	k__norank_ d__Bacteria	p__Proteobacteria	c__Alphaproteobacteria	o__Micavibrionales	f__norank_o__Micavibrionales	g__norank_f__norank_o__Micavibrion ales	s__uncultured_gamma_proteobacterium_g__norank_f__norank_o__Micavibrionales	OTU2279
d__Bacteria	k__norank_ d__Bacteria	p__Cyanobacteria	c__Sericytochromatia	o__norank_c__Sericytochro matia	f__norank_o__norank_c__Sericytochrom atia	g__norank_f__norank_o__norank_c__ Sericytochromatia	s__unclassified_g__norank_f__norank_o__norank_c__Sericytochromatia	OTU349
d__Bacteria	k__norank_ d__Bacteria	p__Chloroflexi	c__Chloroflexia	o__Thermomicrobiales	f__JG30-KF-CM45	g__norank_f__JG30-KF-CM45	s__uncultured_bacterium_g__norank_f__JG30-KF-CM45	OTU588
d__Bacteria	k__norank_ d__Bacteria	p__Chloroflexi	c__Chloroflexia	o__Thermomicrobiales	f__AKYG1722	g__norank_f__AKYG1722	s__metagenome_g__norank_f__AKYG1722	OTU2751
d__Bacteria	k__norank_ d__Bacteria	p__Actinobacteriota	c__Actinobacteria	o__Propionibacteriales	f__Nocardioidaceae	g__Nocardioides	s__Nocardioides_sp._g__Nocardioides	OTU163
d__Bacteria	k__norank_ d__Bacteria	p__Myxococcota	c__Myxococcia	o__Myxococcales	f__Myxococcaceae	g__P3OB-42	s__uncultured_bacterium_g__P3OB-42	OTU917
d__Bacteria	k__norank_ d__Bacteria	p__Firmicutes	c__Bacilli	o__Alicyclobacillales	f__Alicyclobacillaceae	g__Effusibacillus	s__Alicyclobacillaceae_bacterium_JAM-FM0301	OTU96
d__Bacteria	k__norank_ d__Bacteria	p__Chloroflexi	c__AD3	o__norank_c__AD3	f__norank_o__norank_c__AD3	g__norank_f__norank_o__norank_c__ AD3	s__unclassified_g__norank_f__norank_o__norank_c__AD3	OTU2002
d__Bacteria	k__norank_ d__Bacteria	p__Bacteroidota	c__Bacteroidia	o__Chitinophagales	f__Chitinophagaceae	g__Terrimonas	s__uncultured_bacterium_g__Terrimonas	OTU2304
d__Bacteria	k__norank_ d__Bacteria	p__Proteobacteria	c__Alphaproteobacteria	o__Rhizobiales	f__Hyphomicrobiaceae	g__Pedomicrobium	s__unclassified_g__Pedomicrobium	OTU2948
d__Bacteria	k__norank_ d__Bacteria	p__Firmicutes	c__Bacilli	o__Staphylococcales	f__Gemellaceae	g__Gemella	s__uncultured_bacterium_g__Gemella	OTU425

d__Bacteria	k__norank_d__Bacteria	p__Elusimicrobiota	c__Lineage_IIb	o__norank_c__Lineage_IIb	f__norank_o__norank_c__Lineage_IIb	g__norank_f__norank_o__norank_c__Lineage_IIb	s__uncultured_bacterium_g__norank_f__norank_o__norank_c__Lineage_IIb	OTU1197
d__Bacteria	k__norank_d__Bacteria	p__Patescibacteria	c__Saccharimonadia	o__Saccharimonadales	f__unclassified_o__Saccharimonadales	g__unclassified_o__Saccharimonadales	s__unclassified_o__Saccharimonadales	OTU806
d__Bacteria	k__norank_d__Bacteria	p__Proteobacteria	c__Alphaproteobacteria	o__Elsterales	f__norank_o__Elsterales	g__norank_f__norank_o__Elsterales	s__uncultured_Alphaproteobacteria_bacterium_g__norank_f__norank_o__Elsterales	OTU1378
d__Bacteria	k__norank_d__Bacteria	p__Actinobacteriota	c__Actinobacteria	o__Pseudonocardiales	f__Pseudonocardiaceae	g__Actinomycetospora	s__uncultured_bacterium_g__Actinomycetospora	OTU1490
d__Bacteria	k__norank_d__Bacteria	p__Actinobacteriota	c__Thermoleophilia	o__Solirubrobacterales	f__67-14	g__norank_f__67-14	s__unclassified_g__norank_f__67-14	OTU1302
d__Bacteria	k__norank_d__Bacteria	p__Bacteroidota	c__Bacteroidia	o__Sphingobacteriales	f__Sphingobacteriaceae	g__Mucilaginibacter	s__unclassified_g__Mucilaginibacter	OTU2530
d__Bacteria	k__norank_d__Bacteria	p__Myxococcota	c__Polyangia	o__Polyangiales	f__Polyangiaceae	g__Aetherobacter	s__uncultured_bacterium_g__Aetherobacter	OTU1170
d__Bacteria	k__norank_d__Bacteria	p__Nitrospirota	c__Nitrospiria	o__Nitrospirales	f__Nitrospiraceae	g__Nitrospira	s__unclassified_g__Nitrospira	OTU2022
d__Bacteria	k__norank_d__Bacteria	p__Bacteroidota	c__Bacteroidia	o__Flavobacteriales	f__Flavobacteriaceae	g__Flavobacterium	s__uncultured_bacterium_g__Flavobacterium	OTU2982
d__Bacteria	k__norank_d__Bacteria	p__Actinobacteriota	c__Actinobacteria	o__Propionibacteriales	f__Nocardioidaceae	g__Nocardioides	s__metagenome_g__Nocardioides	OTU1318
d__Bacteria	k__norank_d__Bacteria	p__Actinobacteriota	c__Actinobacteria	o__Propionibacteriales	f__Nocardioidaceae	g__Nocardioides	s__Nocardioides_sp._g__Nocardioides	OTU1257
d__Bacteria	k__norank_d__Bacteria	p__Proteobacteria	c__Gammaproteobacteria	o__Diplorickettsiales	f__Diplorickettsiaceae	g__Aquicella	s__uncultured_bacterium_g__Aquicella	OTU2827
d__Bacteria	k__norank_d__Bacteria	p__Myxococcota	c__Polyangia	o__Haliangiales	f__Haliangiaceae	g__Haliangium	s__unclassified_g__Haliangium	OTU1325
d__Bacteria	k__norank_d__Bacteria	p__Proteobacteria	c__Gammaproteobacteria	o__Coxiellales	f__Coxiellaceae	g__Coxiella	s__unclassified_g__Coxiella	OTU932
d__Bacteria	k__norank_d__Bacteria	p__Acidobacteriota	c__Vicinamibacteria	o__Vicinamibacterales	f__Vicinamibacteraceae	g__norank_f__Vicinamibacteraceae	s__unclassified_g__norank_f__Vicinamibacteraceae	OTU2154
d__Bacteria	k__norank_d__Bacteria	p__Planctomycetota	c__Planctomycetes	o__Isosphaerales	f__Isosphaeraceae	g__Tundrisphaera	s__uncultured_planctomycete_g__Tundrisphaera	OTU2822

d__Bacteria	k__norank_d__Bacteria	p__Myxococcota	c__Myxococcia	o__Myxococcales	f__Anaeromyxobacteraceae	g__Anaeromyxobacter	s__unclassified_g__Anaeromyxobacter	OTU1230
d__Bacteria	k__norank_d__Bacteria	p__Proteobacteria	c__Alphaproteobacteria	o__Rickettsiales	f__SM2D12	g__norank_f__SM2D12	s__uncultured_Hyphomicrobiaceae_bacterium_g__norank	OTU2891
d__Bacteria	k__norank_d__Bacteria	p__Bdellovibrionota	c__Oligoflexia	o__Oligoflexales	f__norank_o__Oligoflexales	g__Oligoflexus	s__unclassified_g__Oligoflexus	OTU2747
d__Bacteria	k__norank_d__Bacteria	p__Actinobacteriota	c__Thermoleophilia	o__Solirubrobacterales	f__Solirubrobacteraceae	g__Parviterribacter	s__uncultured_bacterium_g__Parviterribacter	OTU2711
d__Bacteria	k__norank_d__Bacteria	p__Bacteroidota	c__Bacteroidia	o__Chitinophagales	f__Chitinophagaceae	g__Segetibacter	s__uncultured_Chitinophagaceae_bacterium_g__Segetibacter	OTU2287
d__Bacteria	k__norank_d__Bacteria	p__Proteobacteria	c__Alphaproteobacteria	o__Rhizobiales	f__Beijerinckiaceae	g__unclassified_f__Beijerinckiaceae	s__unclassified_f__Beijerinckiaceae	OTU2483
d__Bacteria	k__norank_d__Bacteria	p__Myxococcota	c__Myxococcia	o__Myxococcales	f__Anaeromyxobacteraceae	g__Anaeromyxobacter	s__unclassified_g__Anaeromyxobacter	OTU36
d__Bacteria	k__norank_d__Bacteria	p__Chloroflexi	c__Anaerolineae	o__SBR1031	f__norank_o__SBR1031	g__norank_f__norank_o__SBR1031	s__uncultured_Gemmatimonadetes_bacterium_g__norank_f__norank	OTU1836
d__Bacteria	k__norank_d__Bacteria	p__Myxococcota	c__Polyangia	o__Polyangiales	f__Sandaracinaceae	g__norank_f__Sandaracinaceae	s__uncultured_soil_bacterium_g__norank_f__Sandaracinaceae	OTU2150
d__Bacteria	k__norank_d__Bacteria	p__Proteobacteria	c__Gammaproteobacteria	o__Burkholderiales	f__Oxalobacteraceae	g__Duganella	s__uncultured_bacterium_g__Duganella	OTU2025
d__Bacteria	k__norank_d__Bacteria	p__Chloroflexi	c__KD4-96	o__norank_c__KD4-96	f__norank_o__norank_c__KD4-96	g__norank_f__norank_o__norank_c__KD4-96	s__uncultured_bacterium_g__norank_f__norank_o__norank_c__KD4-96	OTU381
d__Bacteria	k__norank_d__Bacteria	p__Bacteroidota	c__Bacteroidia	o__Chitinophagales	f__Chitinophagaceae	g__Sediminibacterium	s__unclassified_g__Sediminibacterium	OTU1086
d__Bacteria	k__norank_d__Bacteria	p__Bdellovibrionota	c__Oligoflexia	o__0319-6G20	f__norank_o__0319-6G20	g__norank_f__norank_o__0319-6G20	s__unclassified_g__norank_f__norank_o__0319-6G20	OTU133
d__Bacteria	k__norank_d__Bacteria	p__Gemmatimonadota	c__Gemmatimonadetes	o__Gemmatimonadales	f__Gemmatimonadaceae	g__unclassified_f__Gemmatimonadaceae	s__unclassified_f__Gemmatimonadaceae	OTU2647
d__Bacteria	k__norank_d__Bacteria	p__Proteobacteria	c__Gammaproteobacteria	o__Diplorickettsiales	f__Diplorickettsiaceae	g__Aquicella	s__unclassified_g__Aquicella	OTU586
d__Bacteria	k__norank_d__Bacteria	p__Planctomycetota	c__Planctomycetes	o__Gemmatales	f__Gemmataceae	g__norank_f__Gemmataceae	s__unclassified_g__norank_f__Gemmataceae	OTU905

d__Bacteria	k__norank_ d__Bacteria	p__Latescibacterota	c__norank_p__Latescibacterota	o__norank_c__norank_p__Latescibacterota	f__norank_o__norank_c__norank_p__Latescibacterota	g__norank_f__norank_o__norank_c__norank_p__Latescibacterota	s__metagenome_g__norank_f__norank_o__norank_c__norank_p__Latescibacterota	OTU398
d__Bacteria	k__norank_ d__Bacteria	p__Cyanobacteria	c__Cyanobacteriia	o__Chloroplast	f__norank_o__Chloroplast	g__norank_f__norank_o__Chloroplast	s__unclassified_g__norank_f__norank_o__Chloroplast	OTU2018
d__Bacteria	k__norank_ d__Bacteria	p__Elusimicrobiota	c__Lineage_IIa	o__norank_c__Lineage_IIa	f__norank_o__norank_c__Lineage_IIa	g__norank_f__norank_o__norank_c__Lineage_IIa	s__uncultured_bacterium_g__norank_f__norank_o__norank_c__Lineage_IIa	OTU1323
d__Bacteria	k__norank_ d__Bacteria	p__Proteobacteria	c__Gammaproteobacteria	o__Diplorickettsiales	f__Diplorickettsiaceae	g__norank_f__Diplorickettsiaceae	s__unclassified_g__norank_f__Diplorickettsiaceae	OTU71
d__Bacteria	k__norank_ d__Bacteria	p__Myxococcota	c__Polyangia	o__Polyangiales	f__BIrii41	g__norank_f__BIrii41	s__uncultured_bacterium_g__norank_f__BIrii41	OTU763
d__Bacteria	k__norank_ d__Bacteria	p__Actinobacteriota	c__Actinobacteria	o__Frankiales	f__norank_o__Frankiales	g__norank_f__norank_o__Frankiales	s__unclassified_g__norank_f__norank_o__Frankiales	OTU1114
d__Bacteria	k__norank_ d__Bacteria	p__Myxococcota	c__Myxococcia	o__Myxococcales	f__Myxococcaceae	g__P3OB-42	s__uncultured_bacterium_g__P3OB-42	OTU277
d__Bacteria	k__norank_ d__Bacteria	p__Chloroflexi	c__Anaerolineae	o__SBR1031	f__A4b	g__norank_f__A4b	s__unclassified_g__norank_f__A4b	OTU1583
d__Bacteria	k__norank_ d__Bacteria	p__Actinobacteriota	c__unclassified_p__Actinobacteriota	o__unclassified_p__Actinobacteriota	f__unclassified_p__Actinobacteriota	g__unclassified_p__Actinobacteriota	s__unclassified_p__Actinobacteriota	OTU2538
d__Bacteria	k__norank_ d__Bacteria	p__Myxococcota	c__Myxococcia	o__Myxococcales	f__Myxococcaceae	g__P3OB-42	s__uncultured_Archangiaceae_bacterium_g__P3OB-42	OTU1675
d__Bacteria	k__norank_ d__Bacteria	p__Proteobacteria	c__Gammaproteobacteria	o__Burkholderiales	f__Burkholderiaceae	g__Burkholderia-Caballeronia-Paraburkholderia	s__Paraburkholderia_tropica	OTU16
d__Bacteria	k__norank_ d__Bacteria	p__Actinobacteriota	c__Actinobacteria	o__Micrococcales	f__Microbacteriaceae	g__Amnibacterium	s__uncultured_bacterium_g__Amnibacterium	OTU2877
d__Bacteria	k__norank_ d__Bacteria	p__Nitrospirota	c__Nitrospiria	o__Nitrospirales	f__Nitrospiraceae	g__Nitrospira	s__unclassified_g__Nitrospira	OTU971
d__Bacteria	k__norank_ d__Bacteria	p__Planctomycetota	c__Planctomycetes	o__Gemmatales	f__Gemmataceae	g__norank_f__Gemmataceae	s__unclassified_g__norank_f__Gemmataceae	OTU1723
d__Bacteria	k__norank_ d__Bacteria	p__Patescibacteria	c__Saccharimonadia	o__Saccharimonadales	f__Saccharimonadaceae	g__TM7a	s__uncultured_bacterium_g__TM7a	OTU2823
d__Bacteria	k__norank_ d__Bacteria	p__Planctomycetota	c__Planctomycetes	o__Isosphaerales	f__Isosphaeraceae	g__norank_f__Isosphaeraceae	s__uncultured_bacterium_g__norank_f__Isosphaeraceae	OTU699

d__Bacteria	k__norank_ d__Bacteria	p__Chloroflexi	c__Anaerolineae	o__SBR1031	f__A4b	g__norank_f__A4b	s__uncultured_gamma_proteobacterium_g__norank_f__A4b	OTU323
d__Bacteria	k__norank_ d__Bacteria	p__WPS-2	c__norank_p__WPS-2	o__norank_c__norank_p__ WPS-2	f__norank_o__norank_c__norank_p__W PS-2	g__norank_f__norank_o__norank_c__ norank_p__WPS-2	s__uncultured_bacterium_g__norank_f__norank_o__norank_c__norank_p__WPS-2	OTU1983
d__Bacteria	k__norank_ d__Bacteria	p__Acidobacteriota	c__Blastocatellia	o__Pyrinomonadales	f__Pyrinomonadaceae	g__RB41	s__unclassified_g__RB41	OTU890
d__Bacteria	k__norank_ d__Bacteria	p__Abditibacteriota	c__Abditibacteria	o__Abditibacteriales	f__Abditibacteriaceae	g__Abditibacterium	s__uncultured_bacterium_g__Abditibacterium	OTU2992
d__Bacteria	k__norank_ d__Bacteria	p__Planctomycetota	c__Planctomycetes	o__Gemmatales	f__Gemmataceae	g__norank_f__Gemmataceae	s__uncultured_planctomycete_g__norank_f__Gemmataceae	OTU682
d__Bacteria	k__norank_ d__Bacteria	p__Chloroflexi	c__Chloroflexia	o__Thermomicrobiales	f__JG30-KF-CM45	g__norank_f__JG30-KF-CM45	s__metagenome_g__norank_f__JG30-KF-CM45	OTU1408
d__Bacteria	k__norank_ d__Bacteria	p__Myxococcota	c__Polyangia	o__Blfdi19	f__norank_o__Blfdi19	g__norank_f__norank_o__Blfdi19	s__metagenome_g__norank_f__norank_o__Blfdi19	OTU561
d__Bacteria	k__norank_ d__Bacteria	p__Chloroflexi	c__TK10	o__norank_c__TK10	f__norank_o__norank_c__TK10	g__norank_f__norank_o__norank_c__ TK10	s__unclassified_g__norank_f__norank_o__norank_c__TK10	OTU718
d__Bacteria	k__norank_ d__Bacteria	p__Chloroflexi	c__Chloroflexia	o__Chloroflexales	f__Roseiflexaceae	g__norank_f__Roseiflexaceae	s__uncultured_bacterium_g__norank_f__Roseiflexaceae	OTU2616
d__Bacteria	k__norank_ d__Bacteria	p__WPS-2	c__norank_p__WPS-2	o__norank_c__norank_p__ WPS-2	f__norank_o__norank_c__norank_p__W PS-2	g__norank_f__norank_o__norank_c__ norank_p__WPS-2	s__metagenome_g__norank_f__norank_o__norank_c__norank_p__WPS-2	OTU693
d__Bacteria	k__norank_ d__Bacteria	p__Chloroflexi	c__Chloroflexia	o__Kallotenuales	f__AKIW781	g__norank_f__AKIW781	s__uncultured_soil_bacterium_g__norank_f__AKIW781	OTU1179
d__Bacteria	k__norank_ d__Bacteria	p__Acidobacteriota	c__Holophagae	o__Subgroup_7	f__norank_o__Subgroup_7	g__norank_f__norank_o__Subgroup_7	s__unclassified_g__norank_f__norank_o__Subgroup_7	OTU1403
d__Bacteria	k__norank_ d__Bacteria	p__Acidobacteriota	c__Acidobacteriae	o__Bryobacterales	f__Bryobacteraceae	g__Bryobacter	s__metagenome_g__Bryobacter	OTU2189
d__Bacteria	k__norank_ d__Bacteria	p__Proteobacteria	c__Alphaproteobacteria	o__Rhizobiales	f__Beijerinckiaceae	g__Microvirga	s__uncultured_bacterium_g__Microvirga	OTU2726
d__Bacteria	k__norank_ d__Bacteria	p__Patescibacteria	c__Saccharimonadia	o__Saccharimonadales	f__norank_o__Saccharimonadales	g__norank_f__norank_o__Saccharimo nadales	s__unclassified_g__norank_f__norank_o__Saccharimonadales	OTU961
d__Bacteria	k__norank_ d__Bacteria	p__Bacteroidota	c__Bacteroidia	o__Sphingobacteriales	f__Sphingobacteriaceae	g__Pedobacter	s__Pedobacter_boryungensis	OTU2887

d__Bacteria	k__norank_d__Bacteria	p__Actinobacteriota	c__Acidimicrobiia	o__Microtrichales	f__Ilumatobacteraceae	g__CL500-29_marine_group	s__unclassified_g__CL500-29_marine_group	OTU2900
d__Bacteria	k__norank_d__Bacteria	p__Proteobacteria	c__Alphaproteobacteria	o__Acetobacterales	f__Acetobacteraceae	g__Acidiphilium	s__uncultured_bacterium_g__Acidiphilium	OTU1851
d__Bacteria	k__norank_d__Bacteria	p__Armatimonadota	c__norank_p__Armatimonadota	o__norank_c__norank_p__Armatimonadota	f__norank_o__norank_c__norank_p__Armatimonadota	g__norank_f__norank_o__norank_c__norank_p__Armatimonadota	s__uncultured_bacterium_g__norank_f__norank_o__norank_c__norank_p__Armatimonadota	OTU499
d__Bacteria	k__norank_d__Bacteria	p__Proteobacteria	c__Alphaproteobacteria	o__Acetobacterales	f__Acetobacteraceae	g__Rhodovastum	s__metagenome_g__Rhodovastum	OTU1894
d__Bacteria	k__norank_d__Bacteria	p__Proteobacteria	c__Gammaproteobacteria	o__Diplorickettsiales	f__Diplorickettsiaceae	g__norank_f__Diplorickettsiaceae	s__gamma_proteobacterium_WY1	OTU2408
d__Bacteria	k__norank_d__Bacteria	p__Firmicutes	c__Bacilli	o__Alicyclobacillales	f__Alicyclobacillaceae	g__Effusibacillus	s__uncultured_organism_g__Effusibacillus	OTU294
d__Bacteria	k__norank_d__Bacteria	p__Gemmatimonadota	c__Gemmatimonadetes	o__Gemmatimonadales	f__Gemmatimonadaceae	g__Gemmatimonas	s__unclassified_g__Gemmatimonas	OTU1626
d__Bacteria	k__norank_d__Bacteria	p__Actinobacteriota	c__Acidimicrobiia	o__norank_c__Acidimicrobiia	f__norank_o__norank_c__Acidimicrobiia	g__norank_f__norank_o__norank_c__Acidimicrobiia	s__uncultured_bacterium_g__norank_f__norank_o__norank_c__Acidimicrobiia	OTU1483
d__Bacteria	k__norank_d__Bacteria	p__Proteobacteria	c__Alphaproteobacteria	o__norank_c__Alphaproteobacteria	f__norank_o__norank_c__Alphaproteobacteria	g__norank_f__norank_o__norank_c__Alphaproteobacteria	s__uncultured_Rhizobiales_bacterium_g__norank_f__norank	OTU470
d__Bacteria	k__norank_d__Bacteria	p__Bdellovibrionota	c__Oligoflexia	o__0319-6G20	f__norank_o__0319-6G20	g__norank_f__norank_o__0319-6G20	s__uncultured_bacterium_g__norank_f__norank_o__0319-6G20	OTU647
d__Bacteria	k__norank_d__Bacteria	p__Proteobacteria	c__Gammaproteobacteria	o__unclassified_c__Gammaproteobacteria	f__unclassified_c__Gammaproteobacteria	g__unclassified_c__Gammaproteobacteria	s__unclassified_c__Gammaproteobacteria	OTU737
d__Bacteria	k__norank_d__Bacteria	p__Proteobacteria	c__Gammaproteobacteria	o__Burkholderiales	f__Sulfuricellaceae	g__unclassified_f__Sulfuricellaceae	s__unclassified_f__Sulfuricellaceae	OTU683
d__Bacteria	k__norank_d__Bacteria	p__Bacteroidota	c__Bacteroidia	o__Chitinophagales	f__Chitinophagaceae	g__Ferruginibacter	s__unclassified_g__Ferruginibacter	OTU2283
d__Bacteria	k__norank_d__Bacteria	p__Bacteroidota	c__Bacteroidia	o__Chitinophagales	f__Chitinophagaceae	g__Ferruginibacter	s__unclassified_g__Ferruginibacter	OTU2281
d__Bacteria	k__norank_d__Bacteria	p__Actinobacteriota	c__Acidimicrobiia	o__Microtrichales	f__Iamiaceae	g__Iamia	s__metagenome_g__Iamia	OTU2356
d__Bacteria	k__norank_d__Bacteria	p__Chloroflexi	c__JG30-KF-CM66	o__norank_c__JG30-KF-CM66	f__norank_o__norank_c__JG30-KF-CM66	g__norank_f__norank_o__norank_c__JG30-KF-CM66	s__metagenome_g__norank_f__norank_o__norank_c__JG30-KF-CM66	OTU915

d__Bacteria	k__norank_ d__Bacteria	p__Cyanobacteria	c__Sericytochromatia	o__norank_c__Sericytochromatia	f__norank_o__norank_c__Sericytochromatia	g__norank_f__norank_o__norank_c__ Sericytochromatia	s__uncultured_soil_bacterium_g__norank_f__norank_o__norank_c__Sericytochromatia	OTU2296
d__Bacteria	k__norank_ d__Bacteria	p__Actinobacteriota	c__Acidimicrobiia	o__Acidimicrobiales	f__Acidimicrobiaceae	g__norank_f__Acidimicrobiaceae	s__uncultured_bacterium_g__norank_f__Acidimicrobiaceae	OTU2631
d__Bacteria	k__norank_ d__Bacteria	p__Bacteroidota	c__Bacteroidia	o__Chitinophagales	f__Chitinophagaceae	g__Flavisolibacter	s__uncultured_bacterium_g__Flavisolibacter	OTU1809
d__Bacteria	k__norank_ d__Bacteria	p__Proteobacteria	c__Gammaproteobacteria	o__Burkholderiales	f__Comamonadaceae	g__Rhizobacter	s__unclassified_g__Rhizobacter	OTU2322
d__Bacteria	k__norank_ d__Bacteria	p__Actinobacteriota	c__Actinobacteria	o__Frankiales	f__Nakamurellaceae	g__Nakamurella	s__unclassified_g__Nakamurella	OTU968
d__Bacteria	k__norank_ d__Bacteria	p__Proteobacteria	c__Alphaproteobacteria	o__Acetobacterales	f__Acetobacteraceae	g__unclassified_f__Acetobacteraceae	s__unclassified_f__Acetobacteraceae	OTU627
d__Bacteria	k__norank_ d__Bacteria	p__Bacteroidota	c__Bacteroidia	o__Sphingobacteriales	f__NS11-12_marine_group	g__norank_f__NS11-12_marine_group	s__uncultured_bacterium_g__norank_f__NS11-12_marine_group	OTU1268
d__Bacteria	k__norank_ d__Bacteria	p__Chloroflexi	c__Anaerolineae	o__SBR1031	f__A4b	g__norank_f__A4b	s__uncultured_bacterium_g__norank_f__A4b	OTU2215
d__Bacteria	k__norank_ d__Bacteria	p__Myxococcota	c__Polyangia	o__Haliangiales	f__Haliangiaceae	g__Haliangium	s__uncultured_bacterium_g__Haliangium	OTU2271
d__Bacteria	k__norank_ d__Bacteria	p__Planctomycetota	c__Planctomycetes	o__Gemmatales	f__Gemmataceae	g__norank_f__Gemmataceae	s__uncultured_bacterium_g__norank_f__Gemmataceae	OTU703
d__Bacteria	k__norank_ d__Bacteria	p__Proteobacteria	c__Alphaproteobacteria	o__Sphingomonadales	f__Sphingomonadaceae	g__Novosphingobium	s__unclassified_g__Novosphingobium	OTU827
d__Bacteria	k__norank_ d__Bacteria	p__Bacteroidota	c__Bacteroidia	o__Chitinophagales	f__Saprospiraceae	g__norank_f__Saprospiraceae	s__unclassified_g__norank_f__Saprospiraceae	OTU2299
d__Bacteria	k__norank_ d__Bacteria	p__Chloroflexi	c__Anaerolineae	o__Caldilineales	f__Caldilineaceae	g__norank_f__Caldilineaceae	s__unclassified_g__norank_f__Caldilineaceae	OTU922
d__Bacteria	k__norank_ d__Bacteria	p__Chloroflexi	c__Ktedonobacteria	o__Ktedonobacterales	f__Ktedonobacteraceae	g__JG30a-KF-32	s__uncultured_bacterium_g__JG30a-KF-32	OTU1776
d__Bacteria	k__norank_ d__Bacteria	p__Actinobacteriota	c__Acidimicrobiia	o__Microtrichales	f__Ilumatobacteraceae	g__CL500-29_marine_group	s__metagenome_g__CL500-29_marine_group	OTU1256
d__Bacteria	k__norank_ d__Bacteria	p__Planctomycetota	c__Planctomycetes	o__Gemmatales	f__Gemmataceae	g__norank_f__Gemmataceae	s__uncultured_planctomycete_g__norank_f__Gemmataceae	OTU2016

d__Bacteria	k__norank_d__Bacteria	p__Acidobacteriota	c__Blastocatellia	o__DS-100	f__norank_o__DS-100	g__norank_f__norank_o__DS-100	s__unclassified_g__norank_f__norank_o__DS-100	OTU1340
d__Bacteria	k__norank_d__Bacteria	p__Chloroflexi	c__Chloroflexia	o__Chloroflexales	f__Roseiflexaceae	g__norank_f__Roseiflexaceae	s__uncultured_bacterium_g__norank_f__Roseiflexaceae	OTU1189
d__Bacteria	k__norank_d__Bacteria	p__Bacteroidota	c__Bacteroidia	o__Chitinophagales	f__Chitinophagaceae	g__Terrimonas	s__uncultured_Bacteroidetes_bacterium_g__Terrimonas	OTU1605
d__Bacteria	k__norank_d__Bacteria	p__Acidobacteriota	c__Blastocatellia	o__DS-100	f__norank_o__DS-100	g__norank_f__norank_o__DS-100	s__unclassified_g__norank_f__norank_o__DS-100	OTU1346
d__Bacteria	k__norank_d__Bacteria	p__Chloroflexi	c__AD3	o__norank_c__AD3	f__norank_o__norank_c__AD3	g__norank_f__norank_o__norank_c__AD3	s__unclassified_g__norank_f__norank_o__norank_c__AD3	OTU1933
d__Bacteria	k__norank_d__Bacteria	p__Proteobacteria	c__Gammaproteobacteria	o__Burkholderiales	f__Oxalobacteraceae	g__unclassified_f__Oxalobacteraceae	s__unclassified_f__Oxalobacteraceae	OTU2210
d__Bacteria	k__norank_d__Bacteria	p__Acidobacteriota	c__Acidobacteriae	o__Acidobacteriales	f__norank_o__Acidobacteriales	g__norank_f__norank_o__Acidobacteriales	s__uncultured_bacterium_g__norank_f__norank_o__Acidobacteriales	OTU1192
d__Bacteria	k__norank_d__Bacteria	p__Bacteroidota	c__Bacteroidia	o__Sphingobacteriales	f__Sphingobacteriaceae	g__Pedobacter	s__unclassified_g__Pedobacter	OTU276
d__Bacteria	k__norank_d__Bacteria	p__Chloroflexi	c__KD4-96	o__norank_c__KD4-96	f__norank_o__norank_c__KD4-96	g__norank_f__norank_o__norank_c__KD4-96	s__uncultured_bacterium_g__norank_f__norank_o__norank_c__KD4-96	OTU522
d__Bacteria	k__norank_d__Bacteria	p__Cyanobacteria	c__Cyanobacteriia	o__Chloroplast	f__norank_o__Chloroplast	g__norank_f__norank_o__Chloroplast	s__unclassified_g__norank_f__norank_o__Chloroplast	OTU13
d__Bacteria	k__norank_d__Bacteria	p__Proteobacteria	c__Gammaproteobacteria	o__PLTA13	f__norank_o__PLTA13	g__norank_f__norank_o__PLTA13	s__unclassified_g__norank_f__norank_o__PLTA13	OTU1289
d__Bacteria	k__norank_d__Bacteria	p__Bacteroidota	c__Bacteroidia	o__Cytophagales	f__Microscillaceae	g__norank_f__Microscillaceae	s__metagenome_g__norank_f__Microscillaceae	OTU2312
d__Bacteria	k__norank_d__Bacteria	p__Actinobacteriota	c__Thermoleophilia	o__Gaiellales	f__norank_o__Gaiellales	g__norank_f__norank_o__Gaiellales	s__unclassified_g__norank_f__norank_o__Gaiellales	OTU1359
d__Bacteria	k__norank_d__Bacteria	p__Proteobacteria	c__Gammaproteobacteria	o__Xanthomonadales	f__Xanthomonadaceae	g__Stenotrophomonas	s__unclassified_g__Stenotrophomonas	OTU2933
d__Bacteria	k__norank_d__Bacteria	p__Actinobacteriota	c__Thermoleophilia	o__Gaiellales	f__norank_o__Gaiellales	g__norank_f__norank_o__Gaiellales	s__unclassified_g__norank_f__norank_o__Gaiellales	OTU2412
d__Bacteria	k__norank_d__Bacteria	p__Chloroflexi	c__JG30-KF-CM66	o__norank_c__JG30-KF-CM66	f__norank_o__norank_c__JG30-KF-CM66	g__norank_f__norank_o__norank_c__JG30-KF-CM66	s__uncultured_bacterium_g__norank_f__norank_o__norank_c__JG30-KF-CM66	OTU1748

d__Bacteria	k__norank_ d__Bacteria	p__Nitrospirota	c__Nitrospiria	o__Nitrospirales	f__Nitrospiraceae	g__Nitrospira	s__unclassified_g__Nitrospira	OTU1244
d__Bacteria	k__norank_ d__Bacteria	p__Acidobacteriota	c__Subgroup_5	o__norank_c__Subgroup_5	f__norank_o__norank_c__Subgroup_5	g__norank_f__norank_o__norank_c__ Subgroup_5	s__unclassified_g__norank_f__norank_o__norank_c__Subgroup_5	OTU1376
d__Bacteria	k__norank_ d__Bacteria	p__Myxococcota	c__Polyangia	o__Haliangiales	f__Haliangiaceae	g__Haliangium	s__unclassified_g__Haliangium	OTU913
d__Bacteria	k__norank_ d__Bacteria	p__Bdellovibrionota	c__Oligoflexia	o__Oligoflexales	f__norank_o__Oligoflexales	g__norank_f__norank_o__Oligoflexale s	s__unclassified_g__norank_f__norank_o__Oligoflexales	OTU2742
d__Bacteria	k__norank_ d__Bacteria	p__Bacteroidota	c__Bacteroidia	o__Cytophagales	f__Spirosomaceae	g__Spirosoma	s__uncultured_bacterium_g__Spirosoma	OTU2340
d__Bacteria	k__norank_ d__Bacteria	p__Bacteroidota	c__Bacteroidia	o__Cytophagales	f__norank_o__Cytophagales	g__norank_f__norank_o__Cytophagale s	s__uncultured_Bacteroidetes_bacterium_g__norank_f__norank_o__Cytophagales	OTU315
d__Bacteria	k__norank_ d__Bacteria	p__Bacteroidota	c__Bacteroidia	o__Cytophagales	f__Spirosomaceae	g__Spirosoma	s__uncultured_bacterium_g__Spirosoma	OTU2344
d__Bacteria	k__norank_ d__Bacteria	p__Myxococcota	c__Myxococcia	o__Myxococcales	f__Anaeromyxobacteraceae	g__Anaeromyxobacter	s__unclassified_g__Anaeromyxobacter	OTU902
d__Bacteria	k__norank_ d__Bacteria	p__Cyanobacteria	c__Cyanobacteriia	o__Chloroplast	f__norank_o__Chloroplast	g__norank_f__norank_o__Chloroplast	s__uncultured_Cyanobacterium_sp._g__norank_f__norank	OTU340
d__Bacteria	k__norank_ d__Bacteria	p__Bacteroidota	c__Bacteroidia	o__Sphingobacteriales	f__AKYH767	g__norank_f__AKYH767	s__metagenome_g__norank_f__AKYH767	OTU1270
d__Bacteria	k__norank_ d__Bacteria	p__Bdellovibrionota	c__Oligoflexia	o__0319-6G20	f__norank_o__0319-6G20	g__norank_f__norank_o__0319-6G20	s__Bdellovibrionales_bacterium_GWC1_52_8	OTU1780
d__Bacteria	k__norank_ d__Bacteria	p__Verrucomicrobiota	c__Chlamydiae	o__Chlamydiales	f__Parachlamydiaceae	g__norank_f__Parachlamydiaceae	s__metagenome_g__norank_f__Parachlamydiaceae	OTU530
d__Bacteria	k__norank_ d__Bacteria	p__Acidobacteriota	c__Acidobacteriae	o__Bryobacterales	f__Bryobacteraceae	g__Bryobacter	s__unclassified_g__Bryobacter	OTU1076
d__Bacteria	k__norank_ d__Bacteria	p__Bacteroidota	c__Bacteroidia	o__Sphingobacteriales	f__AKYH767	g__norank_f__AKYH767	s__uncultured_bacterium_g__norank_f__AKYH767	OTU436
d__Bacteria	k__norank_ d__Bacteria	p__Proteobacteria	c__Gammaproteobacteria	o__Salinisphaerales	f__Solimonadaceae	g__Alkanibacter	s__metagenome_g__Alkanibacter	OTU892
d__Bacteria	k__norank_ d__Bacteria	p__Firmicutes	c__Bacilli	o__Bacillales	f__Bacillaceae	g__Bacillus	s__unclassified_g__Bacillus	OTU1259

d__Bacteria	k__norank_d__Bacteria	p__Chloroflexi	c__Chloroflexia	o__Thermomicrobiales	f__JG30-KF-CM45	g__norank_f__JG30-KF-CM45	s__metagenome_g__norank_f__JG30-KF-CM45	OTU1444
d__Bacteria	k__norank_d__Bacteria	p__Patescibacteria	c__Saccharimonadia	o__Saccharimonadales	f__norank_o__Saccharimonadales	g__norank_f__norank_o__Saccharimonadales	s__unclassified_g__norank_f__norank_o__Saccharimonadales	OTU1063
d__Bacteria	k__norank_d__Bacteria	p__Proteobacteria	c__Alphaproteobacteria	o__Acetobacterales	f__Acetobacteraceae	g__Craurococcus-Caldovatus	s__uncultured_bacterium_g__Craurococcus-Caldovatus	OTU2791
d__Bacteria	k__norank_d__Bacteria	p__Actinobacteriota	c__Acidimicrobiia	o__IMCC26256	f__norank_o__IMCC26256	g__norank_f__norank_o__IMCC26256	s__uncultured_actinobacterium_g__norank_f__norank_o__IMCC26256	OTU1497
d__Bacteria	k__norank_d__Bacteria	p__Myxococcota	c__Myxococcia	o__Myxococcales	f__Myxococcaceae	g__norank_f__Myxococcaceae	s__uncultured_delta_proteobacterium_g__norank_f__Myxococcaceae	OTU455
d__Bacteria	k__norank_d__Bacteria	p__Proteobacteria	c__Gammaproteobacteria	o__Burkholderiales	f__Alcaligenaceae	g__Achromobacter	s__unclassified_g__Achromobacter	OTU19
d__Bacteria	k__norank_d__Bacteria	p__Cyanobacteria	c__Cyanobacteriia	o__Chloroplast	f__norank_o__Chloroplast	g__norank_f__norank_o__Chloroplast	s__unclassified_g__norank_f__norank_o__Chloroplast	OTU2901
d__Bacteria	k__norank_d__Bacteria	p__Bacteroidota	c__Kapabacteria	o__Kapabacteriales	f__norank_o__Kapabacteriales	g__norank_f__norank_o__Kapabacteriales	s__uncultured_bacterium_g__norank_f__norank_o__Kapabacteriales	OTU774
d__Bacteria	k__norank_d__Bacteria	p__Patescibacteria	c__Saccharimonadia	o__Saccharimonadales	f__Saccharimonadaceae	g__TM7a	s__uncultured_bacterium_g__TM7a	OTU1253
d__Bacteria	k__norank_d__Bacteria	p__Patescibacteria	c__Saccharimonadia	o__Saccharimonadales	f__unclassified_o__Saccharimonadales	g__unclassified_o__Saccharimonadales	s__unclassified_o__Saccharimonadales	OTU257
d__Bacteria	k__norank_d__Bacteria	p__Verrucomicrobiota	c__Verrucomicrobiae	o__Chthoniobacterales	f__Chthoniobacteraceae	g__Chthoniobacter	s__uncultured_bacterium_g__Chthoniobacter	OTU2722
d__Bacteria	k__norank_d__Bacteria	p__Bdellovibrionota	c__Oligoflexia	o__0319-6G20	f__norank_o__0319-6G20	g__norank_f__norank_o__0319-6G20	s__unclassified_g__norank_f__norank_o__0319-6G20	OTU1119
d__Bacteria	k__norank_d__Bacteria	p__Acidobacteriota	c__Vicinamibacteria	o__Vicinamibacterales	f__norank_o__Vicinamibacterales	g__norank_f__norank_o__Vicinamibacterales	s__uncultured_bacterium_259	OTU840
d__Bacteria	k__norank_d__Bacteria	p__Bacteroidota	c__Bacteroidia	o__Cytophagales	f__Hymenobacteraceae	g__Hymenobacter	s__Hymenobacter_sp._PAMC_26628	OTU79
d__Bacteria	k__norank_d__Bacteria	p__Actinobacteriota	c__Acidimicrobiia	o__Microtrichales	f__Iamiaceae	g__Iamia	s__unclassified_g__Iamia	OTU2876
d__Bacteria	k__norank_d__Bacteria	p__Verrucomicrobiota	c__Verrucomicrobiae	o__Pedosphaerales	f__Pedosphaeraceae	g__norank_f__Pedosphaeraceae	s__unclassified_g__norank_f__Pedosphaeraceae	OTU1937

d__Bacteria	k__norank_d__Bacteria	p__Actinobacteriota	c__Actinobacteria	o__Streptosporangiales	f__Streptosporangiales_Incertae_Sedis	g__Motilibacter	s__uncultured_bacterium_g__Motilibacter	OTU1455
d__Bacteria	k__norank_d__Bacteria	p__Chloroflexi	c__TK10	o__norank_c__TK10	f__norank_o__norank_c__TK10	g__norank_f__norank_o__norank_c__TK10	s__metagenome_g__norank_f__norank_o__norank_c__TK10	OTU525
d__Bacteria	k__norank_d__Bacteria	p__Bacteroidota	c__Bacteroidia	o__Cytophagales	f__Spirosomaceae	g__Spirosoma	s__unclassified_g__Spirosoma	OTU2644
d__Bacteria	k__norank_d__Bacteria	p__Armatimonadota	c__Armatimonadia	o__Armatimonadales	f__norank_o__Armatimonadales	g__norank_f__norank_o__Armatimonadales	s__unclassified_g__norank_f__norank_o__Armatimonadales	OTU704
d__Bacteria	k__norank_d__Bacteria	p__Verrucomicrobiota	c__Verrucomicrobiae	o__Chthoniobacterales	f__Xiphinematobacteraceae	g__Candidatus_Xiphinematobacter	s__unclassified_g__Candidatus_Xiphinematobacter	OTU1823
d__Bacteria	k__norank_d__Bacteria	p__Bacteroidota	c__Kapabacteria	o__Kapabacteriales	f__norank_o__Kapabacteriales	g__norank_f__norank_o__Kapabacteriales	s__uncultured_bacterium_KF-JG30-B11	OTU1001
d__Bacteria	k__norank_d__Bacteria	p__Bacteroidota	c__Bacteroidia	o__Flavobacteriales	f__Flavobacteriaceae	g__Flavobacterium	s__Flavobacterium_limicola	OTU182
d__Bacteria	k__norank_d__Bacteria	p__SAR324_cladeMarine_group_B	c__norank_p__SAR324_cladeMarine_group_B	o__norank_c__norank_p__SAR324_cladeMarine_group_B	f__norank_o__norank_c__norank_p__SAR324_cladeMarine_group_B	g__norank_f__norank_o__norank_c__norank_p__SAR324_cladeMarine_group_B	s__uncultured_bacterium_g__norank_f__norank_o__norank_c__norank_p__SAR324_cladeMarine_group_B	OTU164
d__Bacteria	k__norank_d__Bacteria	p__Bacteroidota	c__Bacteroidia	o__Cytophagales	f__Hymenobacteraceae	g__Hymenobacter	s__Hymenobacter_sp._R-36548	OTU454
d__Bacteria	k__norank_d__Bacteria	p__Nitrospirota	c__Nitrospiria	o__Nitrospirales	f__Nitrospiraceae	g__Nitrospira	s__unclassified_g__Nitrospira	OTU1799
d__Bacteria	k__norank_d__Bacteria	p__Patescibacteria	c__Saccharimonadia	o__Saccharimonadales	f__norank_o__Saccharimonadales	g__norank_f__norank_o__Saccharimonadales	s__uncultured_bacterium_g__norank_f__norank_o__Saccharimonadales	OTU155
d__Bacteria	k__norank_d__Bacteria	p__Actinobacteriota	c__Thermoleophilia	o__Solirubrobacterales	f__67-14	g__norank_f__67-14	s__Solirubrobacterales_bacterium_67-14	OTU2533
d__Bacteria	k__norank_d__Bacteria	p__Acidobacteriota	c__Blastocatellia	o__Blastocatellales	f__Blastocatellaceae	g__Blastocatella	s__uncultured_bacterium_g__Blastocatella	OTU447
d__Bacteria	k__norank_d__Bacteria	p__Proteobacteria	c__Gammaproteobacteria	o__Burkholderiales	f__A21b	g__norank_f__A21b	s__uncultured_bacterium_g__norank_f__A21b	OTU1306
d__Bacteria	k__norank_d__Bacteria	p__Acidobacteriota	c__Blastocatellia	o__11-24	f__norank_o__11-24	g__norank_f__norank_o__11-24	s__unclassified_g__norank_f__norank_o__11-24	OTU1398
d__Bacteria	k__norank_d__Bacteria	p__Bdellovibrionota	c__Bdellovibrionia	o__Bdellovibrionales	f__Bdellovibrionaceae	g__Bdellovibrio	s__metagenome_g__Bdellovibrio	OTU644

d__Bacteria	k__norank_d__Bacteria	p__Verrucomicrobiota	c__Verrucomicrobiae	o__Chthoniobacterales	f__Chthoniobacteraceae	g__Chthoniobacter	s__unclassified_g__Chthoniobacter	OTU1227
d__Bacteria	k__norank_d__Bacteria	p__Elusimicrobiota	c__Lineage_IIb	o__norank_c__Lineage_IIb	f__norank_o__norank_c__Lineage_IIb	g__norank_f__norank_o__norank_c__Lineage_IIb	s__uncultured_bacterium_g__norank_f__norank_o__norank_c__Lineage_IIb	OTU1186
d__Bacteria	k__norank_d__Bacteria	p__Acidobacteriota	c__Blastocatellia	o__11-24	f__norank_o__11-24	g__norank_f__norank_o__11-24	s__unclassified_g__norank_f__norank_o__11-24	OTU1247
d__Bacteria	k__norank_d__Bacteria	p__Gemmatimonadota	c__Gemmatimonadetes	o__Gemmatimonadales	f__Gemmatimonadaceae	g__norank_f__Gemmatimonadaceae	s__uncultured_Gemmatimonadetes_bacterium_g__norank_f__Gemmatimonadaceae	OTU569
d__Bacteria	k__norank_d__Bacteria	p__Proteobacteria	c__Alphaproteobacteria	o__Rhizobiales	f__Hyphomicrobiaceae	g__norank_f__Hyphomicrobiaceae	s__metagenome_g__norank_f__Hyphomicrobiaceae	OTU419
d__Bacteria	k__norank_d__Bacteria	p__Actinobacteriota	c__unclassified_p__Actinobacteriota	o__unclassified_p__Actinobacteriota	f__unclassified_p__Actinobacteriota	g__unclassified_p__Actinobacteriota	s__unclassified_p__Actinobacteriota	OTU1506
d__Bacteria	k__norank_d__Bacteria	p__Proteobacteria	c__Alphaproteobacteria	o__Caulobacterales	f__Caulobacteraceae	g__Caulobacter	s__unclassified_g__Caulobacter	OTU1588
d__Bacteria	k__norank_d__Bacteria	p__Myxococcota	c__Polyangia	o__Polyangiales	f__Polyangiaceae	g__Pajaroellobacter	s__uncultured_Polyangiaceae_bacterium_g__Pajaroellobacter	OTU207
d__Bacteria	k__norank_d__Bacteria	p__Chloroflexi	c__Ktedonobacteria	o__Ktedonobacterales	f__JG30-KF-AS9	g__norank_f__JG30-KF-AS9	s__unclassified_g__norank_f__JG30-KF-AS9	OTU1804
d__Bacteria	k__norank_d__Bacteria	p__Chloroflexi	c__JG30-KF-CM66	o__norank_c__JG30-KF-CM66	f__norank_o__norank_c__JG30-KF-CM66	g__norank_f__norank_o__norank_c__JG30-KF-CM66	s__uncultured_bacterium_g__norank_f__norank_o__norank_c__JG30-KF-CM66	OTU2073
d__Bacteria	k__norank_d__Bacteria	p__Myxococcota	c__Polyangia	o__Blfdi19	f__norank_o__Blfdi19	g__norank_f__norank_o__Blfdi19	s__unclassified_g__norank_f__norank_o__Blfdi19	OTU927
d__Bacteria	k__norank_d__Bacteria	p__Armatimonadota	c__norank_p__Armatimonadota	o__norank_c__norank_p__Armatimonadota	f__norank_o__norank_c__norank_p__Armatimonadota	g__norank_f__norank_o__norank_c__norank_p__Armatimonadota	s__unclassified_g__norank_f__norank_o__norank_c__norank_p__Armatimonadota	OTU1801
d__Bacteria	k__norank_d__Bacteria	p__Proteobacteria	c__Alphaproteobacteria	o__Elsterales	f__URHD0088	g__norank_f__URHD0088	s__uncultured_bacterium_g__norank_f__URHD0088	OTU1826
d__Bacteria	k__norank_d__Bacteria	p__Bacteroidota	c__Bacteroidia	o__Chitinophagales	f__Saprospiraceae	g__norank_f__Saprospiraceae	s__uncultured_Haliscomenobacter_sp._g__norank_f__Saprospiraceae	OTU622
d__Bacteria	k__norank_d__Bacteria	p__Bacteroidota	c__Bacteroidia	o__Flavobacteriales	f__Flavobacteriaceae	g__Flavobacterium	s__unclassified_g__Flavobacterium	OTU2320
d__Bacteria	k__norank_d__Bacteria	p__Proteobacteria	c__Alphaproteobacteria	o__Tistrellales	f__Geminicoccaceae	g__Candidatus_Alysiosphaera	s__metagenome_g__Candidatus_Alysiosphaera	OTU560

d__Bacteria	k__norank_ d__Bacteria	p__Verrucomicrobiota	c__Chlamydiae	o__Chlamydiales	f__Parachlamydiaceae	g__Candidatus_Protochlamydia	s__unclassified_g__Candidatus_Protochlamydia	OTU424
d__Bacteria	k__norank_ d__Bacteria	p__Acidobacteriota	c__Subgroup_11	o__norank_c__Subgroup_11	f__norank_o__norank_c__Subgroup_11	g__norank_f__norank_o__norank_c__ Subgroup_11	s__uncultured_bacterium_g__norank_f__norank_o__norank_c__Subgroup_11	OTU1564
d__Bacteria	k__norank_ d__Bacteria	p__WPS-2	c__norank_p__WPS-2	o__norank_c__norank_p__ WPS-2	f__norank_o__norank_c__norank_p__W PS-2	g__norank_f__norank_o__norank_c__ norank_p__WPS-2	s__uncultured_bacterium_g__norank_f__norank_o__norank_c__norank_p__WPS-2	OTU1694
d__Bacteria	k__norank_ d__Bacteria	p__Bdellovibrionota	c__Bdellovibrionia	o__Bdellovibrionales	f__Bdellovibrionaceae	g__Bdellovibrio	s__unclassified_g__Bdellovibrio	OTU209
d__Bacteria	k__norank_ d__Bacteria	p__Bdellovibrionota	c__Bdellovibrionia	o__Bdellovibrionales	f__Bdellovibrionaceae	g__Bdellovibrio	s__uncultured_bacterium_g__Bdellovibrio	OTU54
d__Bacteria	k__norank_ d__Bacteria	p__Actinobacteriota	c__Actinobacteria	o__Frankiales	f__Nakamurellaceae	g__Nakamurella	s__unclassified_g__Nakamurella	OTU563
d__Bacteria	k__norank_ d__Bacteria	p__Firmicutes	c__Bacilli	o__Bacillales	f__Bacillaceae	g__Bacillus	s__unclassified_g__Bacillus	OTU1386
d__Bacteria	k__norank_ d__Bacteria	p__Bacteroidota	c__Bacteroidia	o__Cytophagales	f__Hymenobacteraceae	g__Adhaeribacter	s__uncultured_bacterium_g__Adhaeribacter	OTU2734
d__Bacteria	k__norank_ d__Bacteria	p__Chloroflexi	c__unclassified_p__Chlor oflexi	o__unclassified_p__Chlorofl exi	f__unclassified_p__Chloroflexi	g__unclassified_p__Chloroflexi	s__unclassified_p__Chloroflexi	OTU2972
d__Bacteria	k__norank_ d__Bacteria	p__Proteobacteria	c__Gammaproteobacteria	o__Burkholderiales	f__Nitrosomonadaceae	g__IS-44	s__metagenome_g__IS-44	OTU2083
d__Bacteria	k__norank_ d__Bacteria	p__Gemmatimonadota	c__Gemmatimonadetes	o__Gemmatimonadales	f__Gemmatimonadaceae	g__norank_f__Gemmatimonadaceae	s__uncultured_Gemmatimonadetes_bacterium_g__norank_f__Gemmatimonadaceae	OTU585
d__Bacteria	k__norank_ d__Bacteria	p__Planctomycetota	c__Planctomycetes	o__Gemmatales	f__Gemmataceae	g__norank_f__Gemmataceae	s__unclassified_g__norank_f__Gemmataceae	OTU1477
d__Bacteria	k__norank_ d__Bacteria	p__Proteobacteria	c__Alphaproteobacteria	o__unclassified_c__Alphapr oteobacteria	f__unclassified_c__Alphaproteobacteria	g__unclassified_c__Alphaproteobacteri a	s__unclassified_c__Alphaproteobacteria	OTU1582
d__Bacteria	k__norank_ d__Bacteria	p__Proteobacteria	c__Gammaproteobacteria	o__Xanthomonadales	f__Rhodanobacteraceae	g__Dokdonella	s__unclassified_g__Dokdonella	OTU2136
d__Bacteria	k__norank_ d__Bacteria	p__Bacteroidota	c__Bacteroidia	o__Flavobacteriales	f__NS9_marine_group	g__norank_f__NS9_marine_group	s__metagenome_g__norank_f__NS9_marine_group	OTU2141
d__Bacteria	k__norank_ d__Bacteria	p__Actinobacteriota	c__Acidimicrobiia	o__Microtrichales	f__norank_o__Microtrichales	g__norank_f__norank_o__Microtrichal es	s__uncultured_bacterium_g__norank_f__norank_o__Microtrichales	OTU2687

d__Bacteria	k__norank_d__Bacteria	p__Proteobacteria	c__Alphaproteobacteria	o__Rhizobiales	f__Hyphomicrobiaceae	g__Pedomicrobium	s__uncultured_Hyphomicrobium_sp._g__Pedomicrobium	OTU498
d__Bacteria	k__norank_d__Bacteria	p__Actinobacteriota	c__Acidimicrobiia	o__Microtrichales	f__unclassified_o__Microtrichales	g__unclassified_o__Microtrichales	s__unclassified_o__Microtrichales	OTU2896
d__Bacteria	k__norank_d__Bacteria	p__Acidobacteriota	c__Holophagae	o__Subgroup_7	f__norank_o__Subgroup_7	g__norank_f__norank_o__Subgroup_7	s__uncultured_Acidobacteria_bacterium_g__norank_f__norank_o__Subgroup_7	OTU2435
d__Bacteria	k__norank_d__Bacteria	p__Chloroflexi	c__TK10	o__norank_c__TK10	f__norank_o__norank_c__TK10	g__norank_f__norank_o__norank_c__TK10	s__uncultured_bacterium_g__norank_f__norank_o__norank_c__TK10	OTU2171
d__Bacteria	k__norank_d__Bacteria	p__WPS-2	c__norank_p__WPS-2	o__norank_c__norank_p__WPS-2	f__norank_o__norank_c__norank_p__WPS-2	g__norank_f__norank_o__norank_c__norank_p__WPS-2	s__uncultured_bacterium_g__norank_f__norank_o__norank_c__norank_p__WPS-2	OTU1217
d__Bacteria	k__norank_d__Bacteria	p__Bdellovibrionota	c__Oligoflexia	o__0319-6G20	f__norank_o__0319-6G20	g__norank_f__norank_o__0319-6G20	s__uncultured_Bacteriovorax_sp._g__norank	OTU26
d__Bacteria	k__norank_d__Bacteria	p__Chloroflexi	c__AD3	o__norank_c__AD3	f__norank_o__norank_c__AD3	g__norank_f__norank_o__norank_c__AD3	s__uncultured_bacterium_g__norank_f__norank_o__norank_c__AD3	OTU1778
d__Bacteria	k__norank_d__Bacteria	p__Verrucomicrobiota	c__Verrucomicrobiae	o__Chthoniobacterales	f__Chthoniobacteraceae	g__Chthoniobacter	s__metagenome_g__Chthoniobacter	OTU2113
d__Bacteria	k__norank_d__Bacteria	p__Acidobacteriota	c__Acidobacteriae	o__Bryobacterales	f__Bryobacteraceae	g__Bryobacter	s__unclassified_g__Bryobacter	OTU1180
d__Bacteria	k__norank_d__Bacteria	p__Myxococcota	c__Polyangia	o__Polyangiales	f__unclassified_o__Polyangiales	g__unclassified_o__Polyangiales	s__unclassified_o__Polyangiales	OTU1990
d__Bacteria	k__norank_d__Bacteria	p__Proteobacteria	c__Gammaproteobacteria	o__Gammaproteobacteria_Incertae_Sedis	f__unclassified_o__Gammaproteobacteria_Incertae_Sedis	g__Acidibacter	s__uncultured_proteobacterium_g__Acidibacter	OTU2096
d__Bacteria	k__norank_d__Bacteria	p__Proteobacteria	c__Alphaproteobacteria	o__Rhizobiales	f__Rhizobiales_Incertae_Sedis	g__Bauldia	s__metagenome_g__Bauldia	OTU1042
d__Bacteria	k__norank_d__Bacteria	p__Proteobacteria	c__Gammaproteobacteria	o__Gammaproteobacteria_Incertae_Sedis	f__unclassified_o__Gammaproteobacteria_Incertae_Sedis	g__Acidibacter	s__uncultured_proteobacterium_g__Acidibacter	OTU2092
d__Bacteria	k__norank_d__Bacteria	p__Chloroflexi	c__Chloroflexia	o__Chloroflexales	f__Chloroflexaceae	g__FFCH7168	s__uncultured_bacterium_g__FFCH7168	OTU558
d__Bacteria	k__norank_d__Bacteria	p__Armatimonadota	c__norank_p__Armatimonadota	o__norank_c__norank_p__Armatimonadota	f__norank_o__norank_c__norank_p__Armatimonadota	g__norank_f__norank_o__norank_c__norank_p__Armatimonadota	s__metagenome_g__norank_f__norank_o__norank_c__norank_p__Armatimonadota	OTU2718
d__Bacteria	k__norank_d__Bacteria	p__Chloroflexi	c__Ktedonobacteria	o__C0119	f__norank_o__C0119	g__norank_f__norank_o__C0119	s__uncultured_bacterium_g__norank_f__norank_o__C0119	OTU1026

d__Bacteria	k__norank_ d__Bacteria	p__Firmicutes	c__Bacilli	o__Thermoactinomycetales	f__Thermoactinomycetaceae	g__norank_f__Thermoactinomycetacea e	s__uncultured_bacterium_g__norank_f__Thermoactinomycetaceae	OTU1508
d__Bacteria	k__norank_ d__Bacteria	p__Bacteroidota	c__Bacteroidia	o__Sphingobacteriales	f__Sphingobacteriaceae	g__Sphingobacterium	s__Sphingobacterium_faecium	OTU2889
d__Bacteria	k__norank_ d__Bacteria	p__Proteobacteria	c__Gammaproteobacteria	o__Xanthomonadales	f__Rhodanobacteraceae	g__norank_f__Rhodanobacteraceae	s__uncultured_proteobacterium_g__norank_f__Rhodanobacteraceae	OTU2088
d__Bacteria	k__norank_ d__Bacteria	p__Bacteroidota	c__Bacteroidia	o__Cytophagales	f__Hymenobacteraceae	g__Hymenobacter	s__metagenome_g__Hymenobacter	OTU191
d__Bacteria	k__norank_ d__Bacteria	p__Fibrobacterota	c__Fibrobacteria	o__Fibrobacterales	f__Fibrobacteraceae	g__possible_genus_04	s__metagenome_g__possible_genus_04	OTU2162
d__Bacteria	k__norank_ d__Bacteria	p__Chloroflexi	c__KD4-96	o__norank_c__KD4-96	f__norank_o__norank_c__KD4-96	g__norank_f__norank_o__norank_c__ KD4-96	s__unclassified_g__norank_f__norank_o__norank_c__KD4-96	OTU869
d__Bacteria	k__norank_ d__Bacteria	p__Gemmatimonadota	c__Gemmatimonadetes	o__Gemmatimonadales	f__Gemmatimonadaceae	g__Roseisolibacter	s__unclassified_g__Roseisolibacter	OTU343
d__Bacteria	k__norank_ d__Bacteria	p__Proteobacteria	c__Alphaproteobacteria	o__Rhizobiales	f__Beijerinckiaceae	g__Roseiarcus	s__uncultured_bacterium_g__Roseiarcus	OTU1921
d__Bacteria	k__norank_ d__Bacteria	p__Bacteroidota	c__Bacteroidia	o__Cytophagales	f__Spirosomaceae	g__Larkinella	s__unclassified_g__Larkinella	OTU2318
d__Bacteria	k__norank_ d__Bacteria	p__Gemmatimonadota	c__Gemmatimonadetes	o__Gemmatimonadales	f__Gemmatimonadaceae	g__unclassified_f__Gemmatimonadace ae	s__unclassified_f__Gemmatimonadaceae	OTU1220
d__Bacteria	k__norank_ d__Bacteria	p__Bacteroidota	c__Bacteroidia	o__Cytophagales	f__Microscillaceae	g__norank_f__Microscillaceae	s__unclassified_g__norank_f__Microscillaceae	OTU1551
d__Bacteria	k__norank_ d__Bacteria	p__Chloroflexi	c__Ktedonobacteria	o__B12-WMSP1	f__norank_o__B12-WMSP1	g__norank_f__norank_o__B12-WMSP 1	s__uncultured_bacterium_g__norank_f__norank_o__B12-WMSP1	OTU1821
d__Bacteria	k__norank_ d__Bacteria	p__Acidobacteriota	c__Acidobacteriae	o__Elev-16S-1166	f__norank_o__Elev-16S-1166	g__norank_f__norank_o__Elev-16S-11 66	s__uncultured_bacterium_g__norank_f__norank_o__Elev-16S-1166	OTU609
d__Bacteria	k__norank_ d__Bacteria	p__Actinobacteriota	c__Actinobacteria	o__Frankiales	f__Geodermatophilaceae	g__Blastococcus	s__uncultured_Blastococcus_sp._g__Blastococcus	OTU1011
d__Bacteria	k__norank_ d__Bacteria	p__Myxococcota	c__Polyangia	o__Polyangiales	f__Sandaracinaceae	g__norank_f__Sandaracinaceae	s__metagenome_g__norank_f__Sandaracinaceae	OTU2476
d__Bacteria	k__norank_ d__Bacteria	p__Proteobacteria	c__Gammaproteobacteria	o__Salinisphaerales	f__Solimonadaceae	g__norank_f__Solimonadaceae	s__Sinobacteraceae_bacterium_g__norank	OTU200

d__Bacteria	k__norank_d__Bacteria	p__Acidobacteriota	c__Blastocatellia	o__DS-100	f__norank_o__DS-100	g__norank_f__norank_o__DS-100	s__uncultured_bacterium_g__norank_f__norank_o__DS-100	OTU1293
d__Bacteria	k__norank_d__Bacteria	p__Deinococcota	c__Deinococci	o__Thermales	f__Thermaceae	g__Meiothermus	s__Meiothermus_silvanus_DSM_9946	OTU531
d__Bacteria	k__norank_d__Bacteria	p__unclassified_k__norank_d__Bacteria	c__unclassified_k__norank_d__Bacteria	o__unclassified_k__norank_d__Bacteria	f__unclassified_k__norank_d__Bacteria	g__unclassified_k__norank_d__Bacteria	s__unclassified_k__norank_d__Bacteria	OTU854
d__Bacteria	k__norank_d__Bacteria	p__unclassified_k__norank_d__Bacteria	c__unclassified_k__norank_d__Bacteria	o__unclassified_k__norank_d__Bacteria	f__unclassified_k__norank_d__Bacteria	g__unclassified_k__norank_d__Bacteria	s__unclassified_k__norank_d__Bacteria	OTU855
d__Bacteria	k__norank_d__Bacteria	p__Gemmatimonadota	c__Gemmatimonadetes	o__Gemmatimonadales	f__Gemmatimonadaceae	g__Roseisolibacter	s__unclassified_g__Roseisolibacter	OTU73
d__Bacteria	k__norank_d__Bacteria	p__Actinobacteriota	c__Acidimicrobiia	o__Microtrichales	f__Ilumatobacteraceae	g__norank_f__Ilumatobacteraceae	s__uncultured_Actinomycetales_bacterium_g__norank_f__Ilumatobacteraceae	OTU167
d__Bacteria	k__norank_d__Bacteria	p__Proteobacteria	c__Alphaproteobacteria	o__Acetobacterales	f__Acetobacteraceae	g__Rhodopila	s__unclassified_g__Rhodopila	OTU705
d__Bacteria	k__norank_d__Bacteria	p__Acidobacteriota	c__Holophagae	o__Holophagales	f__Holophagaceae	g__Holophaga	s__unclassified_g__Holophaga	OTU44
d__Bacteria	k__norank_d__Bacteria	p__Actinobacteriota	c__Actinobacteria	o__Micrococcales	f__unclassified_o__Micrococcales	g__unclassified_o__Micrococcales	s__unclassified_o__Micrococcales	OTU2815
d__Bacteria	k__norank_d__Bacteria	p__Bacteroidota	c__Bacteroidia	o__Cytophagales	f__Hymenobacteraceae	g__Hymenobacter	s__Hymenobacter_gelipurpurascens	OTU2187
d__Bacteria	k__norank_d__Bacteria	p__Chloroflexi	c__Anaerolineae	o__Caldilineales	f__Caldilineaceae	g__norank_f__Caldilineaceae	s__uncultured_bacterium_g__norank_f__Caldilineaceae	OTU2725
d__Bacteria	k__norank_d__Bacteria	p__Proteobacteria	c__Gammaproteobacteria	o__Burkholderiales	f__Methylophilaceae	g__Methylotenera	s__uncultured_bacterium_g__Methylotenera	OTU35
d__Bacteria	k__norank_d__Bacteria	p__Bdellovibrionota	c__Oligoflexia	o__0319-6G20	f__norank_o__0319-6G20	g__norank_f__norank_o__0319-6G20	s__unclassified_g__norank_f__norank_o__0319-6G20	OTU782
d__Bacteria	k__norank_d__Bacteria	p__Bdellovibrionota	c__Oligoflexia	o__0319-6G20	f__norank_o__0319-6G20	g__norank_f__norank_o__0319-6G20	s__uncultured_bacterium_g__norank_f__norank_o__0319-6G20	OTU2746
d__Bacteria	k__norank_d__Bacteria	p__Proteobacteria	c__Gammaproteobacteria	o__Burkholderiales	f__Methylophilaceae	g__Methylotenera	s__uncultured_soil_bacterium_g__Methylotenera	OTU2134
d__Bacteria	k__norank_d__Bacteria	p__Chloroflexi	c__JG30-KF-CM66	o__norank_c__JG30-KF-CM66	f__norank_o__norank_c__JG30-KF-CM66	g__norank_f__norank_o__norank_c__JG30-KF-CM66	s__uncultured_bacterium_g__norank_f__norank_o__norank_c__JG30-KF-CM66	OTU1911

d__Bacteria	k__norank_ d__Bacteria	p__Bdellovibrionota	c__Bdellovibrionia	o__Bdellovibrionales	f__Bdellovibrionaceae	g__Bdellovibrio	s__uncultured_bacterium_g__Bdellovibrio	OTU1811
d__Bacteria	k__norank_ d__Bacteria	p__Bdellovibrionota	c__Oligoflexia	o__0319-6G20	f__norank_o__0319-6G20	g__norank_f__norank_o__0319-6G20	s__unclassified_g__norank_f__norank_o__0319-6G20	OTU416
d__Bacteria	k__norank_ d__Bacteria	p__Acidobacteriota	c__Thermoanaerobaculia	o__Thermoanaerobaculales	f__Thermoanaerobaculaceae	g__Subgroup_10	s__uncultured_soil_bacterium_g__Subgroup_10	OTU2377
d__Bacteria	k__norank_ d__Bacteria	p__Myxococcota	c__Myxococcia	o__Myxococcales	f__Myxococcaceae	g__P3OB-42	s__unclassified_g__P3OB-42	OTU448
d__Bacteria	k__norank_ d__Bacteria	p__Myxococcota	c__Polyangia	o__Haliangiales	f__Haliangiaceae	g__Haliangium	s__uncultured_proteobacterium_g__Haliangium	OTU2624
d__Bacteria	k__norank_ d__Bacteria	p__GAL15	c__norank_p__GAL15	o__norank_c__norank_p__ GAL15	f__norank_o__norank_c__norank_p__G AL15	g__norank_f__norank_o__norank_c__ norank_p__GAL15	s__uncultured_bacterium_g__norank_f__norank_o__norank_c__norank_p__GAL15	OTU1571
d__Bacteria	k__norank_ d__Bacteria	p__Firmicutes	c__Bacilli	o__Alicyclobacillales	f__Alicyclobacillaceae	g__Effusibacillus	s__unclassified_g__Effusibacillus	OTU303
d__Bacteria	k__norank_ d__Bacteria	p__Chloroflexi	c__KD4-96	o__norank_c__KD4-96	f__norank_o__norank_c__KD4-96	g__norank_f__norank_o__norank_c__ KD4-96	s__unclassified_g__norank_f__norank_o__norank_c__KD4-96	OTU1068
d__Bacteria	k__norank_ d__Bacteria	p__Patescibacteria	c__Saccharimonadia	o__Saccharimonadales	f__Saccharimonadaceae	g__TM7a	s__uncultured_bacterium_g__TM7a	OTU2850
d__Bacteria	k__norank_ d__Bacteria	p__Patescibacteria	c__Parcubacteria	o__norank_c__Parcubacteria	f__norank_o__norank_c__Parcubacteria	g__norank_f__norank_o__norank_c__ Parcubacteria	s__uncultured_bacterium_g__norank_f__norank_o__norank_c__Parcubacteria	OTU312
d__Bacteria	k__norank_ d__Bacteria	p__Gemmatimonadota	c__Gemmatimonadetes	o__Gemmatimonadales	f__Gemmatimonadaceae	g__Gemmatimonas	s__unclassified_g__Gemmatimonas	OTU235
d__Bacteria	k__norank_ d__Bacteria	p__Bacteroidota	c__Bacteroidia	o__Chitinophagales	f__Chitinophagaceae	g__Ferruginibacter	s__uncultured_bacterium_g__Ferruginibacter	OTU2272
d__Bacteria	k__norank_ d__Bacteria	p__Patescibacteria	c__Saccharimonadia	o__Saccharimonadales	f__norank_o__Saccharimonadales	g__norank_f__norank_o__Saccharimo nadales	s__metagenome_g__norank_f__norank_o__Saccharimonadales	OTU2635
d__Bacteria	k__norank_ d__Bacteria	p__Verrucomicrobiota	c__Verrucomicrobiae	o__Pedosphaerales	f__Pedosphaeraceae	g__norank_f__Pedosphaeraceae	s__unclassified_g__norank_f__Pedosphaeraceae	OTU1109
d__Bacteria	k__norank_ d__Bacteria	p__Planctomycetota	c__Planctomycetes	o__Isosphaerales	f__Isosphaeraceae	g__unclassified_f__Isosphaeraceae	s__unclassified_f__Isosphaeraceae	OTU1825
d__Bacteria	k__norank_ d__Bacteria	p__Bacteroidota	c__Bacteroidia	o__Cytophagales	f__Microscillaceae	g__Ohtaekwangia	s__uncultured_bacterium_g__Ohtaekwangia	OTU2800

d__Bacteria	k__norank_ d__Bacteria	p__Bdellovibrionota	c__Oligoflexia	o__0319-6G20	f__norank_o__0319-6G20	g__norank_f__norank_o__0319-6G20	s__uncultured_Syntrophobacteraceae_bacterium_g__norank	OTU74
d__Bacteria	k__norank_ d__Bacteria	p__Myxococcota	c__Polyangia	o__Polyangiales	f__Polyangiaceae	g__Pajaroellobacter	s__unclassified_g__Pajaroellobacter	OTU1673
d__Bacteria	k__norank_ d__Bacteria	p__Firmicutes	c__Clostridia	o__Oscillospirales	f__Ethanoligenenaceae	g__unclassified_f__Ethanoligenenacea e	s__unclassified_f__Ethanoligenenaceae	OTU1848
d__Bacteria	k__norank_ d__Bacteria	p__Patescibacteria	c__Saccharimonadia	o__Saccharimonadales	f__WWH38	g__norank_f__WWH38	s__uncultured_bacterium_g__norank_f__WWH38	OTU1379
d__Bacteria	k__norank_ d__Bacteria	p__Actinobacteriota	c__Actinobacteria	o__Micrococcales	f__Cellulomonadaceae	g__Actinotalea	s__uncultured_bacterium_g__Actinotalea	OTU2681
d__Bacteria	k__norank_ d__Bacteria	p__Actinobacteriota	c__Actinobacteria	o__Corynebacteriales	f__Nocardiaceae	g__Rhodococcus	s__unclassified_g__Rhodococcus	OTU577
d__Bacteria	k__norank_ d__Bacteria	p__Actinobacteriota	c__MB-A2-108	o__norank_c__MB-A2-108	f__norank_o__norank_c__MB-A2-108	g__norank_f__norank_o__norank_c__ MB-A2-108	s__uncultured_bacterium_g__norank_f__norank_o__norank_c__MB-A2-108	OTU1338
d__Bacteria	k__norank_ d__Bacteria	p__Gemmatimonadota	c__Gemmatimonadetes	o__Gemmatimonadales	f__Gemmatimonadaceae	g__Gemmatimonas	s__unclassified_g__Gemmatimonas	OTU1090
d__Bacteria	k__norank_ d__Bacteria	p__Proteobacteria	c__Gammaproteobacteria	o__JG36-TzT-191	f__norank_o__JG36-TzT-191	g__norank_f__norank_o__JG36-TzT-1 91	s__uncultured_proteobacterium_g__norank_f__norank_o__JG36-TzT-191	OTU2500
d__Bacteria	k__norank_ d__Bacteria	p__Deinococcota	c__Deinococci	o__Deinococcales	f__Deinococcaceae	g__Deinococcus	s__Deinococcus_sp._A23.3	OTU2601
d__Bacteria	k__norank_ d__Bacteria	p__Planctomycetota	c__Planctomycetes	o__Isosphaerales	f__Isosphaeraceae	g__norank_f__Isosphaeraceae	s__uncultured_bacterium_g__norank_f__Isosphaeraceae	OTU702
d__Bacteria	k__norank_ d__Bacteria	p__Verrucomicrobiota	c__Verrucomicrobiae	o__Chthoniobacterales	f__Chthoniobacteraceae	g__Candidatus_Udaeobacter	s__unclassified_g__Candidatus_Udaeobacter	OTU1906
d__Bacteria	k__norank_ d__Bacteria	p__Proteobacteria	c__Gammaproteobacteria	o__Xanthomonadales	f__Xanthomonadaceae	g__Lysobacter	s__unclassified_g__Lysobacter	OTU2956
d__Bacteria	k__norank_ d__Bacteria	p__Firmicutes	c__Bacilli	o__Lactobacillales	f__Streptococcaceae	g__Streptococcus	s__unclassified_g__Streptococcus	OTU466
d__Bacteria	k__norank_ d__Bacteria	p__Bdellovibrionota	c__Bdellovibrionia	o__Bdellovibrionales	f__Bdellovibrionaceae	g__OM27_clade	s__metagenome_g__OM27_clade	OTU246
d__Bacteria	k__norank_ d__Bacteria	p__Firmicutes	c__Bacilli	o__Entomoplasmatales	f__type_III	g__norank_f__type_III	s__uncultured_Mollicutes_bacterium_g__norank	OTU1572

d__Bacteria	k__norank_ d__Bacteria	p__Bacteroidota	c__Bacteroidia	o__Flavobacteriales	f__Flavobacteriaceae	g__Flavobacterium	s__unclassified_g__Flavobacterium	OTU1343
d__Bacteria	k__norank_ d__Bacteria	p__Firmicutes	c__Negativicutes	o__Veillonellales-Selenomo nadales	f__Veillonellaceae	g__Veillonella	s__unclassified_g__Veillonella	OTU397
d__Bacteria	k__norank_ d__Bacteria	p__Proteobacteria	c__Gammaproteobacteria	o__Diplorickettsiales	f__Diplorickettsiaceae	g__Aquicella	s__unclassified_g__Aquicella	OTU1732
d__Bacteria	k__norank_ d__Bacteria	p__Actinobacteriota	c__Acidimicrobiia	o__Microtrichales	f__Iamiaceae	g__Iamia	s__unclassified_g__Iamia	OTU532
d__Bacteria	k__norank_ d__Bacteria	p__Myxococcota	c__Polyangia	o__Haliangiales	f__Haliangiaceae	g__Haliangium	s__uncultured_bacterium_g__Haliangium	OTU816
d__Bacteria	k__norank_ d__Bacteria	p__Proteobacteria	c__Alphaproteobacteria	o__Rhizobiales	f__norank_o__Rhizobiales	g__norank_f__norank_o__Rhizobiales	s__uncultured_bacterium_g__norank_f__norank_o__Rhizobiales	OTU2911
d__Bacteria	k__norank_ d__Bacteria	p__unclassified_k__no rank_d__Bacteria	c__unclassified_k__noran k_d__Bacteria	o__unclassified_k__norank_ d__Bacteria	f__unclassified_k__norank_d__Bacteria	g__unclassified_k__norank_d__Bacteri a	s__unclassified_k__norank_d__Bacteria	OTU1436
d__Bacteria	k__norank_ d__Bacteria	p__Patescibacteria	c__Saccharimonadia	o__Saccharimonadales	f__unclassified_o__Saccharimonadales	g__unclassified_o__Saccharimonadale s	s__unclassified_o__Saccharimonadales	OTU715
d__Bacteria	k__norank_ d__Bacteria	p__Bdellovibrionota	c__Oligoflexia	o__0319-6G20	f__norank_o__0319-6G20	g__norank_f__norank_o__0319-6G20	s__unclassified_g__norank_f__norank_o__0319-6G20	OTU1215
d__Bacteria	k__norank_ d__Bacteria	p__Bdellovibrionota	c__Oligoflexia	o__0319-6G20	f__norank_o__0319-6G20	g__norank_f__norank_o__0319-6G20	s__unclassified_g__norank_f__norank_o__0319-6G20	OTU1216
d__Bacteria	k__norank_ d__Bacteria	p__Armatimonadota	c__Fimbriimonadia	o__Fimbriimonadales	f__Fimbriimonadaceae	g__norank_f__Fimbriimonadaceae	s__uncultured_bacterium_g__norank_f__Fimbriimonadaceae	OTU1637
d__Bacteria	k__norank_ d__Bacteria	p__Chloroflexi	c__Anaerolineae	o__Anaerolineales	f__Anaerolineaceae	g__norank_f__Anaerolineaceae	s__unclassified_g__norank_f__Anaerolineaceae	OTU1434
d__Bacteria	k__norank_ d__Bacteria	p__Actinobacteriota	c__Actinobacteria	o__Propionibacteriales	f__Nocardioidaceae	g__Nocardioides	s__unclassified_g__Nocardioides	OTU440
d__Bacteria	k__norank_ d__Bacteria	p__Actinobacteriota	c__Actinobacteria	o__Micrococcales	f__Cellulomonadaceae	g__Cellulomonas	s__unclassified_g__Cellulomonas	OTU2066
d__Bacteria	k__norank_ d__Bacteria	p__Proteobacteria	c__Alphaproteobacteria	o__Caulobacterales	f__Caulobacteraceae	g__norank_f__Caulobacteraceae	s__metagenome_g__norank_f__Caulobacteraceae	OTU1875
d__Bacteria	k__norank_ d__Bacteria	p__Bdellovibrionota	c__Bdellovibrionia	o__Bdellovibrionales	f__Bdellovibrionaceae	g__OM27_clade	s__unclassified_g__OM27_clade	OTU2068

d__Bacteria	k__norank_d__Bacteria	p__Chloroflexi	c__Chloroflexia	o__Kallotenuales	f__AKIW781	g__norank_f__AKIW781	s__uncultured_bacterium_g__norank_f__AKIW781	OTU2918
d__Bacteria	k__norank_d__Bacteria	p__Chloroflexi	c__TK10	o__norank_c__TK10	f__norank_o__norank_c__TK10	g__norank_f__norank_o__norank_c__TK10	s__uncultured_bacterium_g__norank_f__norank_o__norank_c__TK10	OTU2899
d__Bacteria	k__norank_d__Bacteria	p__Bdellovibrionota	c__Oligoflexia	o__0319-6G20	f__norank_o__0319-6G20	g__norank_f__norank_o__0319-6G20	s__metagenome_g__norank_f__norank_o__0319-6G20	OTU280
d__Bacteria	k__norank_d__Bacteria	p__Bacteroidota	c__Bacteroidia	o__Chitinophagales	f__Chitinophagaceae	g__Puia	s__unclassified_g__Puia	OTU2302
d__Bacteria	k__norank_d__Bacteria	p__Actinobacteriota	c__Acidimicrobiia	o__IMCC26256	f__norank_o__IMCC26256	g__norank_f__norank_o__IMCC26256	s__unclassified_g__norank_f__norank_o__IMCC26256	OTU990
d__Bacteria	k__norank_d__Bacteria	p__Actinobacteriota	c__Actinobacteria	o__Propionibacteriales	f__Propionibacteriaceae	g__unclassified_f__Propionibacteriaceae	s__unclassified_f__Propionibacteriaceae	OTU2194
d__Bacteria	k__norank_d__Bacteria	p__Verrucomicrobiota	c__Chlamydiae	o__Chlamydiales	f__Parachlamydiaceae	g__unclassified_f__Parachlamydiaceae	s__unclassified_f__Parachlamydiaceae	OTU1985
d__Bacteria	k__norank_d__Bacteria	p__Myxococcota	c__Myxococcia	o__Myxococcales	f__Anaeromyxobacteraceae	g__Anaeromyxobacter	s__unclassified_g__Anaeromyxobacter	OTU408
d__Bacteria	k__norank_d__Bacteria	p__Patescibacteria	c__Gracilibacteria	o__Candidatus_Peribacteria	f__norank_o__Candidatus_Peribacteria	g__norank_f__norank_o__Candidatus_Peribacteria	s__unclassified_g__norank_f__norank_o__Candidatus_Peribacteria	OTU965
d__Bacteria	k__norank_d__Bacteria	p__Sumerlaeota	c__Sumerlaeia	o__Sumerlaeales	f__Sumerlaeaceae	g__Sumerlaea	s__uncultured_Acidobacteria_bacterium_g__Sumerlaea	OTU165
d__Bacteria	k__norank_d__Bacteria	p__Chloroflexi	c__Chloroflexia	o__Kallotenuales	f__AKIW781	g__norank_f__AKIW781	s__unclassified_g__norank_f__AKIW781	OTU6
d__Bacteria	k__norank_d__Bacteria	p__Chloroflexi	c__Chloroflexia	o__Chloroflexales	f__Roseiflexaceae	g__norank_f__Roseiflexaceae	s__unclassified_g__norank_f__Roseiflexaceae	OTU524
d__Bacteria	k__norank_d__Bacteria	p__Planctomycetota	c__Planctomycetes	o__Gemmatales	f__Gemmataceae	g__norank_f__Gemmataceae	s__uncultured_bacterium_g__norank_f__Gemmataceae	OTU2934
d__Bacteria	k__norank_d__Bacteria	p__Cyanobacteria	c__Cyanobacteriia	o__Chloroplast	f__norank_o__Chloroplast	g__norank_f__norank_o__Chloroplast	s__unclassified_g__norank_f__norank_o__Chloroplast	OTU2860
d__Bacteria	k__norank_d__Bacteria	p__Verrucomicrobiota	c__Verrucomicrobiae	o__Chthoniobacterales	f__Chthoniobacteraceae	g__Chthoniobacter	s__uncultured_bacterium_g__Chthoniobacter	OTU895
d__Bacteria	k__norank_d__Bacteria	p__Gemmatimonadota	c__Gemmatimonadetes	o__Gemmatimonadales	f__Gemmatimonadaceae	g__Roseisolibacter	s__unclassified_g__Roseisolibacter	OTU1563

d__Bacteria	k__norank_ d__Bacteria	p__Patescibacteria	c__Saccharimonadia	o__Saccharimonadales	f__Saccharimonadaceae	g__TM7a	s__uncultured_bacterium_g__TM7a	OTU268
d__Bacteria	k__norank_ d__Bacteria	p__Chloroflexi	c__Chloroflexia	o__Chloroflexales	f__Roseiflexaceae	g__norank_f__Roseiflexaceae	s__uncultured_bacterium_g__norank_f__Roseiflexaceae	OTU2164
d__Bacteria	k__norank_ d__Bacteria	p__Acidobacteriota	c__Acidobacteriae	o__Acidobacteriales	f__Acidobacteriaceae_Subgroup_1	g__Acidipila	s__uncultured_Acidobacteria_bacterium_g__Acidipila	OTU1857
d__Bacteria	k__norank_ d__Bacteria	p__Bacteroidota	c__SJA-28	o__norank_c__SJA-28	f__norank_o__norank_c__SJA-28	g__norank_f__norank_o__norank_c__ SJA-28	s__unclassified_g__norank_f__norank_o__norank_c__SJA-28	OTU1143
d__Bacteria	k__norank_ d__Bacteria	p__Deinococcota	c__Deinococci	o__Deinococcales	f__Deinococcaceae	g__Deinococcus	s__Deinococcus_misasensis	OTU157
d__Bacteria	k__norank_ d__Bacteria	p__Proteobacteria	c__Gammaproteobacteria	o__Burkholderiales	f__unclassified_o__Burkholderiales	g__unclassified_o__Burkholderiales	s__unclassified_o__Burkholderiales	OTU1160
d__Bacteria	k__norank_ d__Bacteria	p__Actinobacteriota	c__Thermoleophilia	o__Solirubrobacterales	f__67-14	g__norank_f__67-14	s__unclassified_g__norank_f__67-14	OTU1364
d__Bacteria	k__norank_ d__Bacteria	p__Proteobacteria	c__Alphaproteobacteria	o__Sphingomonadales	f__Sphingomonadaceae	g__Sphingomonas	s__unclassified_g__Sphingomonas	OTU1439
d__Bacteria	k__norank_ d__Bacteria	p__Bacteroidota	c__Bacteroidia	o__Cytophagales	f__Hymenobacteraceae	g__Hymenobacter	s__metagenome_g__Hymenobacter	OTU2732
d__Bacteria	k__norank_ d__Bacteria	p__Cyanobacteria	c__Cyanobacteriia	o__Chloroplast	f__norank_o__Chloroplast	g__norank_f__norank_o__Chloroplast	s__Fusochloris_perforata	OTU1753
d__Bacteria	k__norank_ d__Bacteria	p__Proteobacteria	c__Gammaproteobacteria	o__Diplorickettsiales	f__Diplorickettsiaceae	g__norank_f__Diplorickettsiaceae	s__metagenome_g__norank_f__Diplorickettsiaceae	OTU651
d__Bacteria	k__norank_ d__Bacteria	p__Patescibacteria	c__Saccharimonadia	o__Saccharimonadales	f__LWQ8	g__norank_f__LWQ8	s__unclassified_g__norank_f__LWQ8	OTU254
d__Bacteria	k__norank_ d__Bacteria	p__Patescibacteria	c__Saccharimonadia	o__Saccharimonadales	f__norank_o__Saccharimonadales	g__norank_f__norank_o__Saccharimo nadales	s__uncultured_bacterium_g__norank_f__norank_o__Saccharimonadales	OTU542
d__Bacteria	k__norank_ d__Bacteria	p__Cyanobacteria	c__Cyanobacteriia	o__Chloroplast	f__norank_o__Chloroplast	g__norank_f__norank_o__Chloroplast	s__Oophila_amblystomatis	OTU112
d__Bacteria	k__norank_ d__Bacteria	p__Gemmatimonadota	c__Gemmatimonadetes	o__Gemmatimonadales	f__Gemmatimonadaceae	g__norank_f__Gemmatimonadaceae	s__uncultured_Gemmatimonadetes_bacterium_g__norank_f__Gemmatimonadaceae	OTU2772
d__Bacteria	k__norank_ d__Bacteria	p__Patescibacteria	c__Saccharimonadia	o__Saccharimonadales	f__LWQ8	g__norank_f__LWQ8	s__uncultured_bacterium_g__norank_f__LWQ8	OTU2895

d__Bacteria	k__norank_d__Bacteria	p__Chloroflexi	c__Anaerolineae	o__RBG-13-54-9	f__norank_o__RBG-13-54-9	g__norank_f__norank_o__RBG-13-54-9	s__unclassified_g__norank_f__norank_o__RBG-13-54-9	OTU1367
d__Bacteria	k__norank_d__Bacteria	p__Dependentiae	c__Babeliae	o__Babeliales	f__Vermiphilaceae	g__norank_f__Vermiphilaceae	s__unclassified_g__norank_f__Vermiphilaceae	OTU1007
d__Bacteria	k__norank_d__Bacteria	p__Actinobacteriota	c__Actinobacteria	o__Micrococcales	f__Intrasporangiaceae	g__unclassified_f__Intrasporangiaceae	s__unclassified_f__Intrasporangiaceae	OTU1461
d__Bacteria	k__norank_d__Bacteria	p__Bdellovibrionota	c__Oligoflexia	o__0319-6G20	f__norank_o__0319-6G20	g__norank_f__norank_o__0319-6G20	s__unclassified_g__norank_f__norank_o__0319-6G20	OTU1856
d__Bacteria	k__norank_d__Bacteria	p__Chloroflexi	c__Chloroflexia	o__Thermomicrobiales	f__JG30-KF-CM45	g__norank_f__JG30-KF-CM45	s__metagenome_g__norank_f__JG30-KF-CM45	OTU1457
d__Bacteria	k__norank_d__Bacteria	p__Bdellovibrionota	c__Bdellovibrionia	o__Bacteriovoracales	f__Bacteriovoracaceae	g__Peredibacter	s__unclassified_g__Peredibacter	OTU2051
d__Bacteria	k__norank_d__Bacteria	p__Cyanobacteria	c__Sericytochromatia	o__norank_c__Sericytochromatia	f__norank_o__norank_c__Sericytochromatia	g__norank_f__norank_o__norank_c__Sericytochromatia	s__unclassified_g__norank_f__norank_o__norank_c__Sericytochromatia	OTU1709
d__Bacteria	k__norank_d__Bacteria	p__Proteobacteria	c__Alphaproteobacteria	o__Sphingomonadales	f__Sphingomonadaceae	g__Sphingomonas	s__uncultured_Sphingomonadaceae_bacterium_g__Sphingomonas	OTU2893
d__Bacteria	k__norank_d__Bacteria	p__Cyanobacteria	c__Cyanobacteriia	o__Chloroplast	f__norank_o__Chloroplast	g__norank_f__norank_o__Chloroplast	s__Chlorosarcina_brevispinosa	OTU2989
d__Bacteria	k__norank_d__Bacteria	p__Acidobacteriota	c__Acidobacteriae	o__Bryobacterales	f__Bryobacteraceae	g__Bryobacter	s__unclassified_g__Bryobacter	OTU1860
d__Bacteria	k__norank_d__Bacteria	p__Proteobacteria	c__Gammaproteobacteria	o__Burkholderiales	f__Comamonadaceae	g__unclassified_f__Comamonadaceae	s__unclassified_f__Comamonadaceae	OTU2409
d__Bacteria	k__norank_d__Bacteria	p__Verrucomicrobiota	c__Chlamydiae	o__Chlamydiales	f__Parachlamydiaceae	g__unclassified_f__Parachlamydiaceae	s__unclassified_f__Parachlamydiaceae	OTU2216
d__Bacteria	k__norank_d__Bacteria	p__Chloroflexi	c__JG30-KF-CM66	o__norank_c__JG30-KF-CM66	f__norank_o__norank_c__JG30-KF-CM66	g__norank_f__norank_o__norank_c__JG30-KF-CM66	s__uncultured_bacterium_g__norank_f__norank_o__norank_c__JG30-KF-CM66	OTU889
d__Bacteria	k__norank_d__Bacteria	p__Verrucomicrobiota	c__Verrucomicrobiae	o__Pedosphaerales	f__Pedosphaeraceae	g__DEV114	s__uncultured_bacterium_g__DEV114	OTU748
d__Bacteria	k__norank_d__Bacteria	p__Chloroflexi	c__Anaerolineae	o__Caldilineales	f__Caldilineaceae	g__norank_f__Caldilineaceae	s__uncultured_bacterium_g__norank_f__Caldilineaceae	OTU562
d__Bacteria	k__norank_d__Bacteria	p__Bacteroidota	c__Bacteroidia	o__Cytophagales	f__Hymenobacteraceae	g__Hymenobacter	s__unclassified_g__Hymenobacter	OTU1324

d__Bacteria	k__norank_d__Bacteria	p__Fibrobacterota	c__Fibrobacteria	o__Fibrobacterales	f__Fibrobacteraceae	g__possible_genus_04	s__unclassified_g__possible_genus_04	OTU394
d__Bacteria	k__norank_d__Bacteria	p__Actinobacteriota	c__Thermoleophilia	o__Solirubrobacterales	f__67-14	g__norank_f__67-14	s__uncultured_actinobacterium_g__norank_f__67-14	OTU1641
d__Bacteria	k__norank_d__Bacteria	p__Patescibacteria	c__Saccharimonadia	o__Saccharimonadales	f__norank_o__Saccharimonadales	g__norank_f__norank_o__Saccharimonadales	s__uncultured_gamma_proteobacterium_g__norank_f__norank_o__Saccharimonadales	OTU564
d__Bacteria	k__norank_d__Bacteria	p__Chloroflexi	c__AD3	o__norank_c__AD3	f__norank_o__norank_c__AD3	g__norank_f__norank_o__norank_c__AD3	s__uncultured_bacterium_g__norank_f__norank_o__norank_c__AD3	OTU2011
d__Bacteria	k__norank_d__Bacteria	p__Proteobacteria	c__Gammaproteobacteria	o__Burkholderiales	f__SC-I-84	g__norank_f__SC-I-84	s__unclassified_g__norank_f__SC-I-84	OTU1476
d__Bacteria	k__norank_d__Bacteria	p__Proteobacteria	c__Gammaproteobacteria	o__Diplorickettsiales	f__Diplorickettsiaceae	g__norank_f__Diplorickettsiaceae	s__unclassified_g__norank_f__Diplorickettsiaceae	OTU332
d__Bacteria	k__norank_d__Bacteria	p__Acidobacteriota	c__Acidobacteriae	o__Bryobacterales	f__Bryobacteraceae	g__Bryobacter	s__unclassified_g__Bryobacter	OTU512
d__Bacteria	k__norank_d__Bacteria	p__Proteobacteria	c__Alphaproteobacteria	o__Rickettsiales	f__AB1	g__norank_f__AB1	s__unclassified_g__norank_f__AB1	OTU825
d__Bacteria	k__norank_d__Bacteria	p__Gemmatimonadota	c__Gemmatimonadetes	o__Gemmatimonadales	f__Gemmatimonadaceae	g__norank_f__Gemmatimonadaceae	s__uncultured_Gemmatimonadetes_bacterium_g__norank_f__Gemmatimonadaceae	OTU775
d__Bacteria	k__norank_d__Bacteria	p__Firmicutes	c__Desulfitobacteriia	o__Desulfitobacteriales	f__Desulfitobacteriaceae	g__Desulfosporosinus	s__uncultured_Firmicutes_bacterium_g__Desulfosporosinus	OTU2196
d__Bacteria	k__norank_d__Bacteria	p__Proteobacteria	c__Alphaproteobacteria	o__Rhizobiales	f__Rhizobiales_Incertae_Sedis	g__norank_f__Rhizobiales_Incertae_Sedis	s__uncultured_Alphaproteobacteria_bacterium_g__norank_f__Rhizobiales_Incertae_Sedis	OTU1201
d__Bacteria	k__norank_d__Bacteria	p__Bacteroidota	c__Bacteroidia	o__Cytophagales	f__Spirosomaceae	g__Dyadobacter	s__metagenome_g__Dyadobacter	OTU1559
d__Bacteria	k__norank_d__Bacteria	p__Chloroflexi	c__Anaerolineae	o__SBR1031	f__norank_o__SBR1031	g__norank_f__norank_o__SBR1031	s__unclassified_g__norank_f__norank_o__SBR1031	OTU1301
d__Bacteria	k__norank_d__Bacteria	p__Acidobacteriota	c__Vicinamibacteria	o__Vicinamibacterales	f__norank_o__Vicinamibacterales	g__norank_f__norank_o__Vicinamibacterales	s__uncultured_Acidobacteriaceae_bacterium_g__norank_f__norank_o__Vicinamibacterales	OTU2916
d__Bacteria	k__norank_d__Bacteria	p__Proteobacteria	c__Gammaproteobacteria	o__Diplorickettsiales	f__Diplorickettsiaceae	g__norank_f__Diplorickettsiaceae	s__metagenome_g__norank_f__Diplorickettsiaceae	OTU2021
d__Bacteria	k__norank_d__Bacteria	p__Patescibacteria	c__Saccharimonadia	o__Saccharimonadales	f__norank_o__Saccharimonadales	g__norank_f__norank_o__Saccharimonadales	s__TM7_phylum_sp._oral_clone_FR058	OTU656

d__Bacteria	k__norank_d__Bacteria	p__Chloroflexi	c__TK10	o__norank_c__TK10	f__norank_o__norank_c__TK10	g__norank_f__norank_o__norank_c__TK10	s__uncultured_bacterium_g__norank_f__norank_o__norank_c__TK10	OTU473
d__Bacteria	k__norank_d__Bacteria	p__Actinobacteriota	c__Thermoleophilia	o__Solirubrobacterales	f__67-14	g__norank_f__67-14	s__uncultured_bacterium_g__norank_f__67-14	OTU2595
d__Bacteria	k__norank_d__Bacteria	p__Bacteroidota	c__Bacteroidia	o__Sphingobacteriales	f__Sphingobacteriaceae	g__Pedobacter	s__unclassified_g__Pedobacter	OTU2325
d__Bacteria	k__norank_d__Bacteria	p__Chloroflexi	c__Chloroflexia	o__Kallotenuales	f__AKIW781	g__norank_f__AKIW781	s__uncultured_bacterium_g__norank_f__AKIW781	OTU1112
d__Bacteria	k__norank_d__Bacteria	p__Actinobacteriota	c__Thermoleophilia	o__Solirubrobacterales	f__unclassified_o__Solirubrobacterales	g__unclassified_o__Solirubrobacterales	s__unclassified_o__Solirubrobacterales	OTU2221
d__Bacteria	k__norank_d__Bacteria	p__Bacteroidota	c__SJA-28	o__norank_c__SJA-28	f__norank_o__norank_c__SJA-28	g__norank_f__norank_o__norank_c__SJA-28	s__metagenome_g__norank_f__norank_o__norank_c__SJA-28	OTU1450
d__Bacteria	k__norank_d__Bacteria	p__Cyanobacteria	c__Sericytochromatia	o__norank_c__Sericytochromatia	f__norank_o__norank_c__Sericytochromatia	g__norank_f__norank_o__norank_c__Sericytochromatia	s__metagenome_g__norank_f__norank_o__norank_c__Sericytochromatia	OTU772
d__Bacteria	k__norank_d__Bacteria	p__Gemmatimonadota	c__Longimicrobia	o__Longimicrobiales	f__Longimicrobiaceae	g__norank_f__Longimicrobiaceae	s__uncultured_soil_bacterium_g__norank_f__Longimicrobiaceae	OTU2960
d__Bacteria	k__norank_d__Bacteria	p__Actinobacteriota	c__Thermoleophilia	o__Solirubrobacterales	f__Solirubrobacteraceae	g__Solirubrobacter	s__unclassified_g__Solirubrobacter	OTU1480
d__Bacteria	k__norank_d__Bacteria	p__Proteobacteria	c__Gammaproteobacteria	o__Burkholderiales	f__TRA3-20	g__norank_f__TRA3-20	s__unclassified_g__norank_f__TRA3-20	OTU2950
d__Bacteria	k__norank_d__Bacteria	p__Proteobacteria	c__Alphaproteobacteria	o__Acetobacterales	f__Acetobacteraceae	g__Roseomonas	s__unclassified_g__Roseomonas	OTU2855
d__Bacteria	k__norank_d__Bacteria	p__Acidobacteriota	c__Holophagae	o__Subgroup_7	f__norank_o__Subgroup_7	g__norank_f__norank_o__Subgroup_7	s__unclassified_g__norank_f__norank_o__Subgroup_7	OTU1500
d__Bacteria	k__norank_d__Bacteria	p__Acidobacteriota	c__Blastocatellia	o__Pyrinomonadales	f__Pyrinomonadaceae	g__RB41	s__uncultured_bacterium_g__RB41	OTU765
d__Bacteria	k__norank_d__Bacteria	p__Bacteroidota	c__Rhodothermia	o__Rhodothermales	f__Rhodothermaceae	g__Rubrivirga	s__uncultured_bacterium_g__Rubrivirga	OTU474
d__Bacteria	k__norank_d__Bacteria	p__Bacteroidota	c__Bacteroidia	o__Flavobacteriales	f__Weeksellaceae	g__Chryseobacterium	s__unclassified_g__Chryseobacterium	OTU2214
d__Bacteria	k__norank_d__Bacteria	p__Acidobacteriota	c__Acidobacteriae	o__Acidobacteriales	f__Acidobacteriaceae_Subgroup_1	g__Granulicella	s__uncultured_bacterium_g__Granulicella	OTU1899

d__Bacteria	k__norank_d__Bacteria	p__Bdellovibrionota	c__Oligoflexia	o__0319-6G20	f__norank_o__0319-6G20	g__norank_f__norank_o__0319-6G20	s__unclassified_g__norank_f__norank_o__0319-6G20	OTU1929
d__Bacteria	k__norank_d__Bacteria	p__Verrucomicrobiota	c__Verrucomicrobiae	o__Chthoniobacterales	f__Chthoniobacteraceae	g__Candidatus_Udaeobacter	s__uncultured_Verrucomicrobia_bacterium_g__Candidatus_Udaeobacter	OTU1032
d__Bacteria	k__norank_d__Bacteria	p__Patescibacteria	c__Parcubacteria	o__norank_c__Parcubacteria	f__norank_o__norank_c__Parcubacteria	g__norank_f__norank_o__norank_c__Parcubacteria	s__uncultured_bacterium_g__norank_f__norank_o__norank_c__Parcubacteria	OTU2545
d__Bacteria	k__norank_d__Bacteria	p__Bacteroidota	c__Bacteroidia	o__Sphingobacteriales	f__AKYH767	g__norank_f__AKYH767	s__uncultured_Bacteroidetes_bacterium_g__norank_f__AKYH767	OTU645
d__Bacteria	k__norank_d__Bacteria	p__Actinobacteriota	c__Acidimicrobiia	o__IMCC26256	f__norank_o__IMCC26256	g__norank_f__norank_o__IMCC26256	s__uncultured_actinobacterium_g__norank_f__norank_o__IMCC26256	OTU488
d__Bacteria	k__norank_d__Bacteria	p__Chloroflexi	c__Ktedonobacteria	o__C0119	f__norank_o__C0119	g__norank_f__norank_o__C0119	s__uncultured_bacterium_g__norank_f__norank_o__C0119	OTU1682
d__Bacteria	k__norank_d__Bacteria	p__Acidobacteriota	c__Holophagae	o__Subgroup_7	f__norank_o__Subgroup_7	g__norank_f__norank_o__Subgroup_7	s__uncultured_bacterium_g__norank_f__norank_o__Subgroup_7	OTU666
d__Bacteria	k__norank_d__Bacteria	p__Chloroflexi	c__TK10	o__norank_c__TK10	f__norank_o__norank_c__TK10	g__norank_f__norank_o__norank_c__TK10	s__uncultured_Chloroflexi_bacterium_g__norank_f__norank_o__norank_c__TK10	OTU1864
d__Bacteria	k__norank_d__Bacteria	p__Bacteroidota	c__Bacteroidia	o__Cytophagales	f__Hymenobacteraceae	g__Hymenobacter	s__unclassified_g__Hymenobacter	OTU240
d__Bacteria	k__norank_d__Bacteria	p__Chloroflexi	c__Anaerolineae	o__SBR1031	f__A4b	g__norank_f__A4b	s__unclassified_g__norank_f__A4b	OTU2294
d__Bacteria	k__norank_d__Bacteria	p__Bacteroidota	c__Bacteroidia	o__Flavobacteriales	f__Flavobacteriaceae	g__Flavobacterium	s__unclassified_g__Flavobacterium	OTU388
d__Bacteria	k__norank_d__Bacteria	p__Planctomycetota	c__Planctomycetes	o__Isosphaerales	f__Isosphaeraceae	g__norank_f__Isosphaeraceae	s__unclassified_g__norank_f__Isosphaeraceae	OTU1740
d__Bacteria	k__norank_d__Bacteria	p__Bdellovibrionota	c__Oligoflexia	o__0319-6G20	f__norank_o__0319-6G20	g__norank_f__norank_o__0319-6G20	s__unclassified_g__norank_f__norank_o__0319-6G20	OTU333
d__Bacteria	k__norank_d__Bacteria	p__Armatimonadota	c__norank_p__Armatimonadota	o__norank_c__norank_p__Armatimonadota	f__norank_o__norank_c__norank_p__Armatimonadota	g__norank_f__norank_o__norank_c__norank_p__Armatimonadota	s__metagenome_g__norank_f__norank_o__norank_c__norank_p__Armatimonadota	OTU468
d__Bacteria	k__norank_d__Bacteria	p__Bdellovibrionota	c__Oligoflexia	o__0319-6G20	f__norank_o__0319-6G20	g__norank_f__norank_o__0319-6G20	s__uncultured_gamma_proteobacterium_g__norank_f__norank_o__0319-6G20	OTU2044
d__Bacteria	k__norank_d__Bacteria	p__Actinobacteriota	c__Acidimicrobiia	o__Microtrichales	f__norank_o__Microtrichales	g__norank_f__norank_o__Microtrichales	s__unclassified_g__norank_f__norank_o__Microtrichales	OTU274

d__Bacteria	k__norank_d__Bacteria	p__Actinobacteriota	c__Actinobacteria	o__Propionibacteriales	f__Nocardioidaceae	g__Nocardioides	s__unclassified_g__Nocardioides	OTU2580
d__Bacteria	k__norank_d__Bacteria	p__Verrucomicrobiota	c__Verrucomicrobiae	o__Verrucomicrobiales	f__Verrucomicrobiaceae	g__Prosthecobacter	s__metagenome_g__Prosthecobacter	OTU271
d__Bacteria	k__norank_d__Bacteria	p__Armatimonadota	c__Armatimonadia	o__Armatimonadales	f__Armatimonadaceae	g__Armatimonas	s__uncultured_beta_proteobacterium_g__Armatimonas	OTU653
d__Bacteria	k__norank_d__Bacteria	p__Bacteroidota	c__Bacteroidia	o__Chitinophagales	f__Chitinophagaceae	g__Aurantisolimonas	s__metagenome_g__Aurantisolimonas	OTU393
d__Bacteria	k__norank_d__Bacteria	p__Deinococcota	c__Deinococci	o__Deinococcales	f__Deinococcaceae	g__Deinococcus	s__unclassified_g__Deinococcus	OTU1578
d__Bacteria	k__norank_d__Bacteria	p__Acidobacteriota	c__Vicinamibacteria	o__Vicinamibacterales	f__norank_o__Vicinamibacterales	g__norank_f__norank_o__Vicinamibacterales	s__unclassified_g__norank_f__norank_o__Vicinamibacterales	OTU1460
d__Bacteria	k__norank_d__Bacteria	p__Cyanobacteria	c__Sericytochromatia	o__norank_c__Sericytochromatia	f__norank_o__norank_c__Sericytochromatia	g__norank_f__norank_o__norank_c__Sericytochromatia	s__metagenome_g__norank_f__norank_o__norank_c__Sericytochromatia	OTU142
d__Bacteria	k__norank_d__Bacteria	p__Actinobacteriota	c__Acidimicrobiia	o__Microtrichales	f__norank_o__Microtrichales	g__norank_f__norank_o__Microtrichales	s__metagenome_g__norank_f__norank_o__Microtrichales	OTU587
d__Bacteria	k__norank_d__Bacteria	p__Chloroflexi	c__Anaerolineae	o__SBR1031	f__A4b	g__norank_f__A4b	s__uncultured_bacterium_g__norank_f__A4b	OTU2232
d__Bacteria	k__norank_d__Bacteria	p__Armatimonadota	c__Chthonomonadetes	o__Chthonomonadales	f__Chthonomonadaceae	g__Chthonomonas	s__uncultured_bacterium_g__Chthonomonas	OTU797
d__Bacteria	k__norank_d__Bacteria	p__Proteobacteria	c__Alphaproteobacteria	o__unclassified_c__Alphaproteobacteria	f__unclassified_c__Alphaproteobacteria	g__unclassified_c__Alphaproteobacteria	s__unclassified_c__Alphaproteobacteria	OTU2724
d__Bacteria	k__norank_d__Bacteria	p__Chloroflexi	c__Ktedonobacteria	o__Ktedonobacterales	f__Ktedonobacteraceae	g__HSB_OF53-F07	s__uncultured_Ktedonobacter_sp._g__HSB_OF53-F07	OTU2038
d__Bacteria	k__norank_d__Bacteria	p__Chloroflexi	c__Ktedonobacteria	o__Ktedonobacterales	f__Ktedonobacteraceae	g__norank_f__Ktedonobacteraceae	s__unclassified_g__norank_f__Ktedonobacteraceae	OTU124
d__Bacteria	k__norank_d__Bacteria	p__Bacteroidota	c__Bacteroidia	o__Sphingobacteriales	f__AKYH767	g__norank_f__AKYH767	s__unclassified_g__norank_f__AKYH767	OTU445
d__Bacteria	k__norank_d__Bacteria	p__Proteobacteria	c__Gammaproteobacteria	o__Burkholderiales	f__SC-I-84	g__norank_f__SC-I-84	s__metagenome_g__norank_f__SC-I-84	OTU2373
d__Bacteria	k__norank_d__Bacteria	p__Chloroflexi	c__Chloroflexia	o__Kallotenuales	f__AKIW781	g__norank_f__AKIW781	s__uncultured_bacterium_g__norank_f__AKIW781	OTU2349

d__Bacteria	k__norank_ d__Bacteria	p__Patescibacteria	c__Saccharimonadia	o__Saccharimonadales	f__norank_o__Saccharimonadales	g__norank_f__norank_o__Saccharimo nadales	s__uncultured_bacterium_g__norank_f__norank_o__Saccharimonadales	OTU958
d__Bacteria	k__norank_ d__Bacteria	p__Acidobacteriota	c__Vicinamibacteria	o__Subgroup_17	f__norank_o__Subgroup_17	g__norank_f__norank_o__Subgroup_1 7	s__uncultured_Acidobacteriales_bacterium_g__norank_f__norank_o__Subgroup_17	OTU1432
d__Bacteria	k__norank_ d__Bacteria	p__Actinobacteriota	c__Actinobacteria	o__Micromonosporales	f__Micromonosporaceae	g__Dactylosporangium	s__uncultured_bacterium_g__Dactylosporangium	OTU2379
d__Bacteria	k__norank_ d__Bacteria	p__Verrucomicrobiota	c__Chlamydiae	o__Chlamydiales	f__Parachlamydiaceae	g__Parachlamydia	s__unclassified_g__Parachlamydia	OTU859
d__Bacteria	k__norank_ d__Bacteria	p__Cyanobacteria	c__Cyanobacteriia	o__Cyanobacteriales	f__Chroococcidiopsaceae	g__Crinalium_SAG_22.89	s__uncultured_cyanobacterium_g__Crinalium_SAG_22.89	OTU1129
d__Bacteria	k__norank_ d__Bacteria	p__Proteobacteria	c__Alphaproteobacteria	o__Sphingomonadales	f__Sphingomonadaceae	g__unclassified_f__Sphingomonadacea e	s__unclassified_f__Sphingomonadaceae	OTU2169
d__Bacteria	k__norank_ d__Bacteria	p__Myxococcota	c__Polyangia	o__Polyangiales	f__Polyangiaceae	g__norank_f__Polyangiaceae	s__metagenome_g__norank_f__Polyangiaceae	OTU2706
d__Bacteria	k__norank_ d__Bacteria	p__Bacteroidota	c__Bacteroidia	o__Chitinophagales	f__Chitinophagaceae	g__norank_f__Chitinophagaceae	s__uncultured_Chitinophaga_sp._g__norank	OTU1319
d__Bacteria	k__norank_ d__Bacteria	p__Patescibacteria	c__Parcubacteria	o__Candidatus_Kaiserbacter ia	f__norank_o__Candidatus_Kaiserbacteria	g__norank_f__norank_o__Candidatus_ Kaiserbacteria	s__metagenome_g__norank_f__norank_o__Candidatus_Kaiserbacteria	OTU2958
d__Bacteria	k__norank_ d__Bacteria	p__Actinobacteriota	c__Thermoleophilia	o__Gaiellales	f__norank_o__Gaiellales	g__norank_f__norank_o__Gaiellales	s__bacterium_Ellin6517	OTU1829
d__Bacteria	k__norank_ d__Bacteria	p__Chloroflexi	c__AD3	o__norank_c__AD3	f__norank_o__norank_c__AD3	g__norank_f__norank_o__norank_c__ AD3	s__unclassified_g__norank_f__norank_o__norank_c__AD3	OTU1955
d__Bacteria	k__norank_ d__Bacteria	p__Proteobacteria	c__Alphaproteobacteria	o__Rhizobiales	f__Rhizobiaceae	g__Allorhizobium-Neorhizobium-Parar hizobium-Rhizobium	s__Pararhizobium_giardinii	OTU523
d__Bacteria	k__norank_ d__Bacteria	p__Actinobacteriota	c__Actinobacteria	o__Micrococcales	f__Microbacteriaceae	g__unclassified_f__Microbacteriaceae	s__unclassified_f__Microbacteriaceae	OTU2869
d__Bacteria	k__norank_ d__Bacteria	p__Patescibacteria	c__Saccharimonadia	o__Saccharimonadales	f__norank_o__Saccharimonadales	g__norank_f__norank_o__Saccharimo nadales	s__uncultured_Candidatus_Saccharibacteria_bacterium_g__norank_f__norank	OTU154
d__Bacteria	k__norank_ d__Bacteria	p__Chloroflexi	c__Chloroflexia	o__Thermomicrobiales	f__JG30-KF-CM45	g__norank_f__JG30-KF-CM45	s__unclassified_g__norank_f__JG30-KF-CM45	OTU1688
d__Bacteria	k__norank_ d__Bacteria	p__unclassified_k__no rank_d__Bacteria	c__unclassified_k__noran k_d__Bacteria	o__unclassified_k__norank_ d__Bacteria	f__unclassified_k__norank_d__Bacteria	g__unclassified_k__norank_d__Bacteri a	s__unclassified_k__norank_d__Bacteria	OTU849

d__Bacteria	k__norank_d__Bacteria	p__Cyanobacteria	c__Cyanobacteriia	o__Chloroplast	f__norank_o__Chloroplast	g__norank_f__norank_o__Chloroplast	s__unclassified_g__norank_f__norank_o__Chloroplast	OTU876
d__Bacteria	k__norank_d__Bacteria	p__Proteobacteria	c__Gammaproteobacteria	o__Diplorickettsiales	f__Diplorickettsiaceae	g__Aquicella	s__uncultured_bacterium_g__Aquicella	OTU688
d__Bacteria	k__norank_d__Bacteria	p__Chloroflexi	c__AD3	o__norank_c__AD3	f__norank_o__norank_c__AD3	g__norank_f__norank_o__norank_c__AD3	s__uncultured_bacterium_g__norank_f__norank_o__norank_c__AD3	OTU1703
d__Bacteria	k__norank_d__Bacteria	p__Bdellovibrionota	c__Bdellovibrionia	o__Bdellovibrionales	f__Bdellovibrionaceae	g__OM27_clade	s__uncultured_bacterium_g__OM27_clade	OTU2205
d__Bacteria	k__norank_d__Bacteria	p__Proteobacteria	c__Alphaproteobacteria	o__Caulobacterales	f__Caulobacteraceae	g__unclassified_f__Caulobacteraceae	s__unclassified_f__Caulobacteraceae	OTU2890
d__Bacteria	k__norank_d__Bacteria	p__Armatimonadota	c__Armatimonadia	o__Armatimonadales	f__norank_o__Armatimonadales	g__norank_f__norank_o__Armatimonadales	s__uncultured_bacterium_g__norank_f__norank_o__Armatimonadales	OTU186
d__Bacteria	k__norank_d__Bacteria	p__Gemmatimonadota	c__Gemmatimonadetes	o__Gemmatimonadales	f__Gemmatimonadaceae	g__Gemmatimonas	s__unclassified_g__Gemmatimonas	OTU2527
d__Bacteria	k__norank_d__Bacteria	p__Proteobacteria	c__Gammaproteobacteria	o__Burkholderiales	f__Burkholderiaceae	g__Burkholderia-Caballeronia-Paraburkholderia	s__Paraburkholderia_silvatlantica	OTU529
d__Bacteria	k__norank_d__Bacteria	p__Gemmatimonadota	c__Gemmatimonadetes	o__Gemmatimonadales	f__Gemmatimonadaceae	g__norank_f__Gemmatimonadaceae	s__unclassified_g__norank_f__Gemmatimonadaceae	OTU552
d__Bacteria	k__norank_d__Bacteria	p__Proteobacteria	c__Gammaproteobacteria	o__Burkholderiales	f__Comamonadaceae	g__unclassified_f__Comamonadaceae	s__unclassified_f__Comamonadaceae	OTU2396
d__Bacteria	k__norank_d__Bacteria	p__Firmicutes	c__Bacilli	o__Entomoplasmatales	f__type_III	g__norank_f__type_III	s__unclassified_g__norank_f__type_III	OTU1619
d__Bacteria	k__norank_d__Bacteria	p__Bacteroidota	c__Bacteroidia	o__Chitinophagales	f__Chitinophagaceae	g__Lacibacter	s__uncultured_bacterium_g__Lacibacter	OTU414
d__Bacteria	k__norank_d__Bacteria	p__Proteobacteria	c__Alphaproteobacteria	o__Acetobacterales	f__Acetobacteraceae	g__Roseomonas	s__unclassified_g__Roseomonas	OTU120
d__Bacteria	k__norank_d__Bacteria	p__Cyanobacteria	c__Cyanobacteriia	o__Leptolyngbyales	f__Leptolyngbyaceae	g__Phormidesmis_ANT.L52.6	s__uncultured_bacterium_g__Phormidesmis_ANT.L52.6	OTU1616
d__Bacteria	k__norank_d__Bacteria	p__Bacteroidota	c__Bacteroidia	o__Sphingobacteriales	f__AKYH767	g__norank_f__AKYH767	s__metagenome_g__norank_f__AKYH767	OTU1514
d__Bacteria	k__norank_d__Bacteria	p__Firmicutes	c__Clostridia	o__Eubacteriales	f__Eubacteriaceae	g__Acetobacterium	s__uncultured_bacterium_g__Acetobacterium	OTU1579

d__Bacteria	k__norank_ d__Bacteria	p__Planctomycetota	c__BD7-11	o__norank_c__BD7-11	f__norank_o__norank_c__BD7-11	g__norank_f__norank_o__norank_c__ BD7-11	s__uncultured_bacterium_g__norank_f__norank_o__norank_c__BD7-11	OTU852
d__Bacteria	k__norank_ d__Bacteria	p__Cyanobacteria	c__Cyanobacteriia	o__Chloroplast	f__norank_o__Chloroplast	g__norank_f__norank_o__Chloroplast	s__unclassified_g__norank_f__norank_o__Chloroplast	OTU1353
d__Bacteria	k__norank_ d__Bacteria	p__Planctomycetota	c__Planctomycetes	o__Isosphaerales	f__Isosphaeraceae	g__norank_f__Isosphaeraceae	s__uncultured_bacterium_g__norank_f__Isosphaeraceae	OTU2745
d__Bacteria	k__norank_ d__Bacteria	p__Planctomycetota	c__Planctomycetes	o__Isosphaerales	f__Isosphaeraceae	g__norank_f__Isosphaeraceae	s__uncultured_bacterium_g__norank_f__Isosphaeraceae	OTU716
d__Bacteria	k__norank_ d__Bacteria	p__Proteobacteria	c__Gammaproteobacteria	o__Pseudomonadales	f__Pseudomonadaceae	g__Pseudomonas	s__Pseudomonas_psychrotolerans	OTU2701
d__Bacteria	k__norank_ d__Bacteria	p__Actinobacteriota	c__Thermoleophilia	o__Gaiellales	f__norank_o__Gaiellales	g__norank_f__norank_o__Gaiellales	s__metagenome_g__norank_f__norank_o__Gaiellales	OTU2444
d__Bacteria	k__norank_ d__Bacteria	p__Proteobacteria	c__Gammaproteobacteria	o__Burkholderiales	f__TRA3-20	g__norank_f__TRA3-20	s__uncultured_beta_proteobacterium_g__norank_f__TRA3-20	OTU724
d__Bacteria	k__norank_ d__Bacteria	p__Chloroflexi	c__Chloroflexia	o__Thermomicrobiales	f__JG30-KF-CM45	g__norank_f__JG30-KF-CM45	s__metagenome_g__norank_f__JG30-KF-CM45	OTU2880
d__Bacteria	k__norank_ d__Bacteria	p__Myxococcota	c__Polyangia	o__Haliangiales	f__Haliangiaceae	g__Haliangium	s__unclassified_g__Haliangium	OTU287
d__Bacteria	k__norank_ d__Bacteria	p__Bacteroidota	c__Bacteroidia	o__Flavobacteriales	f__Flavobacteriaceae	g__Flavobacterium	s__uncultured_bacterium_g__Flavobacterium	OTU2833
d__Bacteria	k__norank_ d__Bacteria	p__Bdellovibrionota	c__Oligoflexia	o__Oligoflexales	f__norank_o__Oligoflexales	g__norank_f__norank_o__Oligoflexale s	s__uncultured_bacterium_Ak20-3	OTU872
d__Bacteria	k__norank_ d__Bacteria	p__Bacteroidota	c__Bacteroidia	o__Cytophagales	f__Hymenobacteraceae	g__Hymenobacter	s__metagenome_g__Hymenobacter	OTU1606
d__Bacteria	k__norank_ d__Bacteria	p__Firmicutes	c__Clostridia	o__Clostridiales	f__Clostridiaceae	g__Clostridium_sensu_stricto_1	s__unclassified_g__Clostridium_sensu_stricto_1	OTU1830
d__Bacteria	k__norank_ d__Bacteria	p__Gemmatimonadota	c__Gemmatimonadetes	o__Gemmatimonadales	f__Gemmatimonadaceae	g__Gemmatimonas	s__uncultured_bacterium_g__Gemmatimonas	OTU1239
d__Bacteria	k__norank_ d__Bacteria	p__Bacteroidota	c__Bacteroidia	o__Chitinophagales	f__Saprospiraceae	g__norank_f__Saprospiraceae	s__unclassified_g__norank_f__Saprospiraceae	OTU405
d__Bacteria	k__norank_ d__Bacteria	p__Planctomycetota	c__Planctomycetes	o__Gemmatales	f__Gemmataceae	g__norank_f__Gemmataceae	s__unclassified_g__norank_f__Gemmataceae	OTU723

d__Bacteria	k__norank_d__Bacteria	p__Bacteroidota	c__Bacteroidia	o__Chitinophagales	f__Chitinophagaceae	g__Segetibacter	s__unclassified_g__Segetibacter	OTU214
d__Bacteria	k__norank_d__Bacteria	p__Actinobacteriota	c__Actinobacteria	o__Propionibacteriales	f__Nocardioidaceae	g__Nocardioides	s__Nocardioides_plantarum	OTU2754
d__Bacteria	k__norank_d__Bacteria	p__Cyanobacteria	c__Cyanobacteriia	o__Leptolyngbyales	f__Leptolyngbyaceae	g__Chamaesiphon_PCC-7430	s__uncultured_bacterium_g__Chamaesiphon_PCC-7430	OTU91
d__Bacteria	k__norank_d__Bacteria	p__Chloroflexi	c__Chloroflexia	o__Chloroflexales	f__Chloroflexaceae	g__unclassified_f__Chloroflexaceae	s__unclassified_f__Chloroflexaceae	OTU1538
d__Bacteria	k__norank_d__Bacteria	p__unclassified_k__norank_d__Bacteria	c__unclassified_k__norank_d__Bacteria	o__unclassified_k__norank_d__Bacteria	f__unclassified_k__norank_d__Bacteria	g__unclassified_k__norank_d__Bacteria	s__unclassified_k__norank_d__Bacteria	OTU936
d__Bacteria	k__norank_d__Bacteria	p__unclassified_k__norank_d__Bacteria	c__unclassified_k__norank_d__Bacteria	o__unclassified_k__norank_d__Bacteria	f__unclassified_k__norank_d__Bacteria	g__unclassified_k__norank_d__Bacteria	s__unclassified_k__norank_d__Bacteria	OTU931
d__Bacteria	k__norank_d__Bacteria	p__Proteobacteria	c__Gammaproteobacteria	o__Burkholderiales	f__Procabacteriaceae	g__Procabacter	s__Candidatus_Procabacter_sp._OEW1	OTU92
d__Bacteria	k__norank_d__Bacteria	p__Patescibacteria	c__Saccharimonadia	o__Saccharimonadales	f__LWQ8	g__norank_f__LWQ8	s__uncultured_Candidatus_Saccharibacteria_bacterium_g__norank_f__LWQ8	OTU2007
d__Bacteria	k__norank_d__Bacteria	p__Proteobacteria	c__Gammaproteobacteria	o__Ga0077536	f__norank_o__Ga0077536	g__norank_f__norank_o__Ga0077536	s__uncultured_bacterium_g__norank_f__norank_o__Ga0077536	OTU2573
d__Bacteria	k__norank_d__Bacteria	p__Proteobacteria	c__Gammaproteobacteria	o__Gammaproteobacteria_Incertae_Sedis	f__unclassified_o__Gammaproteobacteria_Incertae_Sedis	g__norank_f__unclassified	s__uncultured_soil_bacterium_g__norank_f__unclassified	OTU1421
d__Bacteria	k__norank_d__Bacteria	p__Gemmatimonadota	c__Gemmatimonadetes	o__Gemmatimonadales	f__Gemmatimonadaceae	g__norank_f__Gemmatimonadaceae	s__metagenome_g__norank_f__Gemmatimonadaceae	OTU334
d__Bacteria	k__norank_d__Bacteria	p__Actinobacteriota	c__Actinobacteria	o__Micrococcales	f__Micrococcaceae	g__Renibacterium	s__unclassified_g__Renibacterium	OTU843
d__Bacteria	k__norank_d__Bacteria	p__Acidobacteriota	c__Thermoanaerobaculia	o__Thermoanaerobaculales	f__Thermoanaerobaculaceae	g__Subgroup_10	s__uncultured_bacterium_g__Subgroup_10	OTU421
d__Bacteria	k__norank_d__Bacteria	p__Bacteroidota	c__Bacteroidia	o__Cytophagales	f__Cytophagaceae	g__Rhodocytophaga	s__uncultured_Bacteroidetes_bacterium_g__Rhodocytophaga	OTU2917
d__Bacteria	k__norank_d__Bacteria	p__Proteobacteria	c__Gammaproteobacteria	o__Xanthomonadales	f__Rhodanobacteraceae	g__Rudaea	s__uncultured_gamma_proteobacterium_g__Rudaea	OTU1958
d__Bacteria	k__norank_d__Bacteria	p__Actinobacteriota	c__Thermoleophilia	o__Solirubrobacterales	f__Solirubrobacteraceae	g__Solirubrobacter	s__uncultured_bacterium_g__Solirubrobacter	OTU2381

d__Bacteria	k__norank_d__Bacteria	p__Acidobacteriota	c__Acidobacteriae	o__Bryobacterales	f__Bryobacteraceae	g__Bryobacter	s__unclassified_g__Bryobacter	OTU2342
d__Bacteria	k__norank_d__Bacteria	p__Actinobacteriota	c__MB-A2-108	o__norank_c__MB-A2-108	f__norank_o__norank_c__MB-A2-108	g__norank_f__norank_o__norank_c__MB-A2-108	s__unclassified_g__norank_f__norank_o__norank_c__MB-A2-108	OTU2184
d__Bacteria	k__norank_d__Bacteria	p__Chloroflexi	c__OLB14	o__norank_c__OLB14	f__norank_o__norank_c__OLB14	g__norank_f__norank_o__norank_c__OLB14	s__bacterium_SCGC_AG-212-C10	OTU613
d__Bacteria	k__norank_d__Bacteria	p__Bacteroidota	c__Bacteroidia	o__Sphingobacteriales	f__env.OPS_17	g__norank_f__env.OPS_17	s__uncultured_Bacteroidetes_bacterium_g__norank_f__env.OPS_17	OTU1080
d__Bacteria	k__norank_d__Bacteria	p__Bdellovibrionota	c__Oligoflexia	o__0319-6G20	f__norank_o__0319-6G20	g__norank_f__norank_o__0319-6G20	s__unclassified_g__norank_f__norank_o__0319-6G20	OTU1939
d__Bacteria	k__norank_d__Bacteria	p__Actinobacteriota	c__Actinobacteria	o__Pseudonocardiales	f__Pseudonocardiaceae	g__Pseudonocardia	s__uncultured_bacterium_g__Pseudonocardia	OTU160
d__Bacteria	k__norank_d__Bacteria	p__unclassified_k__norank_d__Bacteria	c__unclassified_k__norank_d__Bacteria	o__unclassified_k__norank_d__Bacteria	f__unclassified_k__norank_d__Bacteria	g__unclassified_k__norank_d__Bacteria	s__unclassified_k__norank_d__Bacteria	OTU1222
d__Bacteria	k__norank_d__Bacteria	p__Acidobacteriota	c__Blastocatellia	o__Pyrinomonadales	f__Pyrinomonadaceae	g__RB41	s__uncultured_bacterium_g__RB41	OTU1229
d__Bacteria	k__norank_d__Bacteria	p__Armatimonadota	c__norank_p__Armatimonadota	o__norank_c__norank_p__Armatimonadota	f__norank_o__norank_c__norank_p__Armatimonadota	g__norank_f__norank_o__norank_c__norank_p__Armatimonadota	s__uncultured_bacterium_g__norank_f__norank_o__norank_c__norank_p__Armatimonadota	OTU824
d__Bacteria	k__norank_d__Bacteria	p__Deinococcota	c__Deinococci	o__Deinococcales	f__Deinococcaceae	g__norank_f__Deinococcaceae	s__uncultured_bacterium_g__norank_f__Deinococcaceae	OTU1448
d__Bacteria	k__norank_d__Bacteria	p__unclassified_k__norank_d__Bacteria	c__unclassified_k__norank_d__Bacteria	o__unclassified_k__norank_d__Bacteria	f__unclassified_k__norank_d__Bacteria	g__unclassified_k__norank_d__Bacteria	s__unclassified_k__norank_d__Bacteria	OTU948
d__Bacteria	k__norank_d__Bacteria	p__Cyanobacteria	c__Vampirivibrionia	o__Obscuribacterales	f__Obscuribacteraceae	g__norank_f__Obscuribacteraceae	s__uncultured_bacterium_g__norank_f__Obscuribacteraceae	OTU2036
d__Bacteria	k__norank_d__Bacteria	p__Chloroflexi	c__Ktedonobacteria	o__C0119	f__norank_o__C0119	g__norank_f__norank_o__C0119	s__uncultured_bacterium_g__norank_f__norank_o__C0119	OTU1833
d__Bacteria	k__norank_d__Bacteria	p__Patescibacteria	c__Saccharimonadia	o__Saccharimonadales	f__unclassified_o__Saccharimonadales	g__unclassified_o__Saccharimonadales	s__unclassified_o__Saccharimonadales	OTU1263
d__Bacteria	k__norank_d__Bacteria	p__Acidobacteriota	c__Acidobacteriae	o__Acidobacteriales	f__norank_o__Acidobacteriales	g__norank_f__norank_o__Acidobacteriales	s__unclassified_g__norank_f__norank_o__Acidobacteriales	OTU1056
d__Bacteria	k__norank_d__Bacteria	p__SAR324_cladeMarine_group_B	c__norank_p__SAR324_cladeMarine_group_B	o__norank_c__norank_p__SAR324_cladeMarine_group_B	f__norank_o__norank_c__norank_p__SAR324_cladeMarine_group_B	g__norank_f__norank_o__norank_c__norank_p__SAR324_cladeMarine_group_B	s__unclassified_g__norank_f__norank_o__norank_c__norank_p__SAR324_cladeMarine_group_B	OTU2944

d__Bacteria	k__norank_d__Bacteria	p__Proteobacteria	c__Gammaproteobacteria	o__Xanthomonadales	f__Rhodanobacteraceae	g__norank_f__Rhodanobacteraceae	s__uncultured_bacterium_g__norank_f__Rhodanobacteraceae	OTU175
d__Bacteria	k__norank_d__Bacteria	p__Acidobacteriota	c__Acidobacteriae	o__Acidobacteriales	f__norank_o__Acidobacteriales	g__norank_f__norank_o__Acidobacteriales	s__uncultured_proteobacterium_g__norank_f__norank_o__Acidobacteriales	OTU1313
d__Bacteria	k__norank_d__Bacteria	p__Proteobacteria	c__Gammaproteobacteria	o__Burkholderiales	f__Burkholderiaceae	g__Robbsia	s__uncultured_Burkholderia_sp._g__Robbsia	OTU1657
d__Bacteria	k__norank_d__Bacteria	p__Acidobacteriota	c__Blastocatellia	o__Blastocatellales	f__Blastocatellaceae	g__JGI_0001001-H03	s__uncultured_bacterium_g__JGI_0001001-H03	OTU1147
d__Bacteria	k__norank_d__Bacteria	p__Acidobacteriota	c__Acidobacteriae	o__Bryobacterales	f__Bryobacteraceae	g__Bryobacter	s__uncultured_bacterium_g__Bryobacter	OTU1987
d__Bacteria	k__norank_d__Bacteria	p__Bdellovibrionota	c__Bdellovibrionia	o__Bacteriovoracales	f__Bacteriovoracaceae	g__Peredibacter	s__metagenome_g__Peredibacter	OTU335
d__Bacteria	k__norank_d__Bacteria	p__Actinobacteriota	c__Thermoleophilia	o__Gaiellales	f__Gaiellaceae	g__Gaiella	s__unclassified_g__Gaiella	OTU2471
d__Bacteria	k__norank_d__Bacteria	p__Myxococcota	c__Polyangia	o__Polyangiales	f__BIrii41	g__norank_f__BIrii41	s__uncultured_bacterium_g__norank_f__BIrii41	OTU299
d__Bacteria	k__norank_d__Bacteria	p__Armatimonadota	c__Armatimonadia	o__Armatimonadales	f__norank_o__Armatimonadales	g__norank_f__norank_o__Armatimonadales	s__uncultured_bacterium_g__norank_f__norank_o__Armatimonadales	OTU884
d__Bacteria	k__norank_d__Bacteria	p__Verrucomicrobiota	c__Chlamydiae	o__Chlamydiales	f__Criblamydiaceae	g__norank_f__Criblamydiaceae	s__uncultured_organism_g__norank_f__Criblamydiaceae	OTU1356
d__Bacteria	k__norank_d__Bacteria	p__Planctomycetota	c__Planctomycetes	o__Gemmatales	f__Gemmataceae	g__norank_f__Gemmataceae	s__unclassified_g__norank_f__Gemmataceae	OTU1070
d__Bacteria	k__norank_d__Bacteria	p__unclassified_k__norank_d__Bacteria	c__unclassified_k__norank_d__Bacteria	o__unclassified_k__norank_d__Bacteria	f__unclassified_k__norank_d__Bacteria	g__unclassified_k__norank_d__Bacteria	s__unclassified_k__norank_d__Bacteria	OTU1689
d__Bacteria	k__norank_d__Bacteria	p__Actinobacteriota	c__Actinobacteria	o__Micrococcales	f__Intrasporangiaceae	g__Ornithinimicrobium	s__unclassified_g__Ornithinimicrobium	OTU2700
d__Bacteria	k__norank_d__Bacteria	p__Chloroflexi	c__Ktedonobacteria	o__C0119	f__norank_o__C0119	g__norank_f__norank_o__C0119	s__uncultured_bacterium_g__norank_f__norank_o__C0119	OTU1787
d__Bacteria	k__norank_d__Bacteria	p__Proteobacteria	c__Alphaproteobacteria	o__Rhizobiales	f__Rhizobiales_Incertae_Sedis	g__Bauldia	s__uncultured_bacterium_g__Bauldia	OTU2274
d__Bacteria	k__norank_d__Bacteria	p__Bdellovibrionota	c__Bdellovibrionia	o__Bdellovibrionales	f__Bdellovibrionaceae	g__Bdellovibrio	s__unclassified_g__Bdellovibrio	OTU314

d__Bacteria	k__norank_ d__Bacteria	p__Chloroflexi	c__Ktedonobacteria	o__C0119	f__norank_o__C0119	g__norank_f__norank_o__C0119	s__uncultured_bacterium_g__norank_f__norank_o__C0119	OTU1781
d__Bacteria	k__norank_ d__Bacteria	p__Proteobacteria	c__Gammaproteobacteria	o__Cellvibrionales	f__Spongiibacteraceae	g__BD1-7_clade	s__uncultured_Haliea_sp._g__BD1-7_clade	OTU2047
d__Bacteria	k__norank_ d__Bacteria	p__Planctomycetota	c__Planctomycetes	o__Isosphaerales	f__Isosphaeraceae	g__unclassified_f__Isosphaeraceae	s__unclassified_f__Isosphaeraceae	OTU787
d__Bacteria	k__norank_ d__Bacteria	p__Actinobacteriota	c__Thermoleophilia	o__Solirubrobacterales	f__67-14	g__norank_f__67-14	s__uncultured_soil_bacterium_g__norank_f__67-14	OTU2490
d__Bacteria	k__norank_ d__Bacteria	p__Chloroflexi	c__Anaerolineae	o__SBR1031	f__A4b	g__norank_f__A4b	s__uncultured_soil_bacterium_g__norank_f__A4b	OTU2323
d__Bacteria	k__norank_ d__Bacteria	p__Proteobacteria	c__Alphaproteobacteria	o__Rhizobiales	f__Rhizobiaceae	g__Mesorhizobium	s__Mesorhizobium_mediterraneum	OTU611
d__Bacteria	k__norank_ d__Bacteria	p__Actinobacteriota	c__Acidimicrobiia	o__Microtrichales	f__Iamiaceae	g__Iamia	s__uncultured_bacterium_g__Iamia	OTU574
d__Bacteria	k__norank_ d__Bacteria	p__Actinobacteriota	c__Thermoleophilia	o__Solirubrobacterales	f__Solirubrobacteraceae	g__Conexibacter	s__unclassified_g__Conexibacter	OTU309
d__Bacteria	k__norank_ d__Bacteria	p__Bacteroidota	c__Bacteroidia	o__Flavobacteriales	f__Weeksellaceae	g__Chryseobacterium	s__unclassified_g__Chryseobacterium	OTU1326
d__Bacteria	k__norank_ d__Bacteria	p__Myxococcota	c__Polyangia	o__Haliangiales	f__Haliangiaceae	g__Haliangium	s__uncultured_bacterium_g__Haliangium	OTU1327
d__Bacteria	k__norank_ d__Bacteria	p__Gemmatimonadota	c__Gemmatimonadetes	o__Gemmatimonadales	f__Gemmatimonadaceae	g__unclassified_f__Gemmatimonadace ae	s__unclassified_f__Gemmatimonadaceae	OTU407
d__Bacteria	k__norank_ d__Bacteria	p__Bdellovibrionota	c__Bdellovibrionia	o__Bdellovibrionales	f__Bdellovibrionaceae	g__Bdellovibrio	s__unclassified_g__Bdellovibrio	OTU2741
d__Bacteria	k__norank_ d__Bacteria	p__Armatimonadota	c__norank_p__Armatimon adota	o__norank_c__norank_p__ Armatimonadota	f__norank_o__norank_c__norank_p__Ar matimonadota	g__norank_f__norank_o__norank_c__ norank_p__Armatimonadota	s__uncultured_Armatimonadetes_bacterium_g__norank_f__norank_o__norank_c__ norank	OTU1868
d__Bacteria	k__norank_ d__Bacteria	p__Proteobacteria	c__Alphaproteobacteria	o__Acetobacterales	f__Acetobacteraceae	g__norank_f__Acetobacteraceae	s__uncultured_bacterium_g__norank_f__Acetobacteraceae	OTU1065
d__Bacteria	k__norank_ d__Bacteria	p__Chloroflexi	c__Chloroflexia	o__Elev-1554	f__norank_o__Elev-1554	g__norank_f__norank_o__Elev-1554	s__uncultured_bacterium_g__norank_f__norank_o__Elev-1554	OTU57
d__Bacteria	k__norank_ d__Bacteria	p__Proteobacteria	c__Gammaproteobacteria	o__Xanthomonadales	f__Rhodanobacteraceae	g__Ahniella	s__uncultured_bacterium_g__Ahniella	OTU306

d__Bacteria	k__norank_d__Bacteria	p__Proteobacteria	c__Gammaproteobacteria	o__Xanthomonadales	f__Xanthomonadaceae	g__Stenotrophomonas	s__Stenotrophomonas_rhizophila_g__Stenotrophomonas	OTU1013
d__Bacteria	k__norank_d__Bacteria	p__Armatimonadota	c__Armatimonadia	o__Armatimonadales	f__norank_o__Armatimonadales	g__norank_f__norank_o__Armatimonadales	s__uncultured_bacterium_g__norank_f__norank_o__Armatimonadales	OTU1039
d__Bacteria	k__norank_d__Bacteria	p__Chloroflexi	c__Ktedonobacteria	o__Ktedonobacterales	f__Ktedonobacteraceae	g__HSB_OF53-F07	s__unclassified_g__HSB_OF53-F07	OTU1789
d__Bacteria	k__norank_d__Bacteria	p__Acidobacteriota	c__Vicinamibacteria	o__Vicinamibacterales	f__norank_o__Vicinamibacterales	g__norank_f__norank_o__Vicinamibacterales	s__metagenome_g__norank_f__norank_o__Vicinamibacterales	OTU2617
d__Bacteria	k__norank_d__Bacteria	p__Planctomycetota	c__Planctomycetes	o__Gemmatales	f__Gemmataceae	g__norank_f__Gemmataceae	s__unclassified_g__norank_f__Gemmataceae	OTU929
d__Bacteria	k__norank_d__Bacteria	p__Proteobacteria	c__Gammaproteobacteria	o__Xanthomonadales	f__Rhodanobacteraceae	g__Rhodanobacter	s__uncultured_Xanthomonadaceae_bacterium_g__Rhodanobacter	OTU865
d__Bacteria	k__norank_d__Bacteria	p__Proteobacteria	c__Gammaproteobacteria	o__Legionellales	f__Legionellaceae	g__Legionella	s__uncultured_bacterium_g__Legionella	OTU122
d__Bacteria	k__norank_d__Bacteria	p__Planctomycetota	c__Planctomycetes	o__Gemmatales	f__Gemmataceae	g__norank_f__Gemmataceae	s__unclassified_g__norank_f__Gemmataceae	OTU926
d__Bacteria	k__norank_d__Bacteria	p__Proteobacteria	c__Alphaproteobacteria	o__Rhodospirillales	f__norank_o__Rhodospirillales	g__norank_f__norank_o__Rhodospirillales	s__unclassified_g__norank_f__norank_o__Rhodospirillales	OTU906
d__Bacteria	k__norank_d__Bacteria	p__Chloroflexi	c__Ktedonobacteria	o__unclassified_c__Ktedonobacteria	f__unclassified_c__Ktedonobacteria	g__unclassified_c__Ktedonobacteria	s__unclassified_c__Ktedonobacteria	OTU1994
d__Bacteria	k__norank_d__Bacteria	p__Patescibacteria	c__Dojkabacteria	o__norank_c__Dojkabacteria	f__norank_o__norank_c__Dojkabacteria	g__norank_f__norank_o__norank_c__Dojkabacteria	s__unclassified_g__norank_f__norank_o__norank_c__Dojkabacteria	OTU659
d__Bacteria	k__norank_d__Bacteria	p__Proteobacteria	c__Gammaproteobacteria	o__Pseudomonadales	f__Moraxellaceae	g__Enhydrobacter	s__Moraxella_osloensis_g__Enhydrobacter	OTU2809
d__Bacteria	k__norank_d__Bacteria	p__Bacteroidota	c__Bacteroidia	o__Cytophagales	f__Hymenobacteraceae	g__Hymenobacter	s__unclassified_g__Hymenobacter	OTU1134
d__Bacteria	k__norank_d__Bacteria	p__Chloroflexi	c__Gitt-GS-136	o__norank_c__Gitt-GS-136	f__norank_o__norank_c__Gitt-GS-136	g__norank_f__norank_o__norank_c__Gitt-GS-136	s__unclassified_g__norank_f__norank_o__norank_c__Gitt-GS-136	OTU465
d__Bacteria	k__norank_d__Bacteria	p__unclassified_k__norank_d__Bacteria	c__unclassified_k__norank_d__Bacteria	o__unclassified_k__norank_d__Bacteria	f__unclassified_k__norank_d__Bacteria	g__unclassified_k__norank_d__Bacteria	s__unclassified_k__norank_d__Bacteria	OTU713
d__Bacteria	k__norank_d__Bacteria	p__Proteobacteria	c__Alphaproteobacteria	o__Rhodobacterales	f__Rhodobacteraceae	g__Amaricoccus	s__metagenome_g__Amaricoccus	OTU549

d__Bacteria	k__norank_d__Bacteria	p__Patescibacteria	c__Saccharimonadia	o__Saccharimonadales	f__unclassified_o__Saccharimonadales	g__unclassified_o__Saccharimonadales	s__unclassified_o__Saccharimonadales	OTU1040
d__Bacteria	k__norank_d__Bacteria	p__Chloroflexi	c__Ktedonobacteria	o__B12-WMSP1	f__norank_o__B12-WMSP1	g__norank_f__norank_o__B12-WMSP1	s__uncultured_Chloroflexi_bacterium_g__norank_f__norank_o__B12-WMSP1	OTU1992
d__Bacteria	k__norank_d__Bacteria	p__Proteobacteria	c__Alphaproteobacteria	o__Sphingomonadales	f__Sphingomonadaceae	g__Sphingomonas	s__unclassified_g__Sphingomonas	OTU2345
d__Bacteria	k__norank_d__Bacteria	p__Myxococcota	c__Polyangia	o__Haliangiales	f__Haliangiaceae	g__Haliangium	s__unclassified_g__Haliangium	OTU671
d__Bacteria	k__norank_d__Bacteria	p__Bacteroidota	c__Bacteroidia	o__Cytophagales	f__Hymenobacteraceae	g__Hymenobacter	s__Hymenobacter_fastidiosus	OTU70
d__Bacteria	k__norank_d__Bacteria	p__Gemmatimonadota	c__Gemmatimonadetes	o__Gemmatimonadales	f__Gemmatimonadaceae	g__norank_f__Gemmatimonadaceae	s__uncultured_Gemmatimonadales_bacterium_g__norank_f__Gemmatimonadaceae	OTU1314
d__Bacteria	k__norank_d__Bacteria	p__Proteobacteria	c__Gammaproteobacteria	o__Burkholderiales	f__Comamonadaceae	g__Caenimonas	s__unclassified_g__Caenimonas	OTU1285
d__Bacteria	k__norank_d__Bacteria	p__Myxococcota	c__Polyangia	o__Polyangiales	f__BIrii41	g__norank_f__BIrii41	s__unclassified_g__norank_f__BIrii41	OTU2127
d__Bacteria	k__norank_d__Bacteria	p__Gemmatimonadota	c__Gemmatimonadetes	o__Gemmatimonadales	f__Gemmatimonadaceae	g__unclassified_f__Gemmatimonadaceae	s__unclassified_f__Gemmatimonadaceae	OTU2546
d__Bacteria	k__norank_d__Bacteria	p__Actinobacteriota	c__Acidimicrobiia	o__Microtrichales	f__Ilumatobacteraceae	g__norank_f__Ilumatobacteraceae	s__uncultured_organism_g__norank_f__Ilumatobacteraceae	OTU1654
d__Bacteria	k__norank_d__Bacteria	p__Actinobacteriota	c__Acidimicrobiia	o__norank_c__Acidimicrobiia	f__norank_o__norank_c__Acidimicrobiia	g__norank_f__norank_o__norank_c__Acidimicrobiia	s__uncultured_Iamia_sp._g__norank	OTU261
d__Bacteria	k__norank_d__Bacteria	p__Planctomycetota	c__Phycisphaerae	o__Phycisphaerales	f__Phycisphaeraceae	g__CL500-3	s__uncultured_bacterium_g__CL500-3	OTU2217
d__Bacteria	k__norank_d__Bacteria	p__Chloroflexi	c__Chloroflexia	o__Thermomicrobiales	f__AKYG1722	g__norank_f__AKYG1722	s__uncultured_Alphaproteobacteria_bacterium_g__norank_f__AKYG1722	OTU2859
d__Bacteria	k__norank_d__Bacteria	p__Chloroflexi	c__TK10	o__norank_c__TK10	f__norank_o__norank_c__TK10	g__norank_f__norank_o__norank_c__TK10	s__unclassified_g__norank_f__norank_o__norank_c__TK10	OTU734
d__Bacteria	k__norank_d__Bacteria	p__Actinobacteriota	c__Acidimicrobiia	o__unclassified_c__Acidimicrobiia	f__unclassified_c__Acidimicrobiia	g__unclassified_c__Acidimicrobiia	s__unclassified_c__Acidimicrobiia	OTU359
d__Bacteria	k__norank_d__Bacteria	p__Chloroflexi	c__Chloroflexia	o__Thermomicrobiales	f__JG30-KF-CM45	g__norank_f__JG30-KF-CM45	s__uncultured_bacterium_g__norank_f__JG30-KF-CM45	OTU2659

d__Bacteria	k__norank_d__Bacteria	p__Myxococcota	c__Polyangia	o__mle1-27	f__norank_o__mle1-27	g__norank_f__norank_o__mle1-27	s__metagenome_g__norank_f__norank_o__mle1-27	OTU770
d__Bacteria	k__norank_d__Bacteria	p__Verrucomicrobiota	c__Verrucomicrobiae	o__Verrucomicrobiales	f__Verrucomicrobiaceae	g__norank_f__Verrucomicrobiaceae	s__uncultured_verrucomicrobium_DEV009	OTU2370
d__Bacteria	k__norank_d__Bacteria	p__Myxococcota	c__Polyangia	o__Polyangiales	f__Polyangiaceae	g__Pajaroellobacter	s__uncultured_bacterium_g__Pajaroellobacter	OTU1957
d__Bacteria	k__norank_d__Bacteria	p__Chloroflexi	c__Ktedonobacteria	o__C0119	f__norank_o__C0119	g__norank_f__norank_o__C0119	s__unclassified_g__norank_f__norank_o__C0119	OTU1954
d__Bacteria	k__norank_d__Bacteria	p__Chloroflexi	c__Chloroflexia	o__Thermomicrobiales	f__JG30-KF-CM45	g__norank_f__JG30-KF-CM45	s__unclassified_g__norank_f__JG30-KF-CM45	OTU2603
d__Bacteria	k__norank_d__Bacteria	p__Cyanobacteria	c__Cyanobacteriia	o__Cyanobacteriales	f__Chroococcidiopsaceae	g__Chlorogloea_SAG_10.99	s__uncultured_cyanobacterium_g__Chlorogloea_SAG_10.99	OTU796
d__Bacteria	k__norank_d__Bacteria	p__Myxococcota	c__Polyangia	o__mle1-27	f__norank_o__mle1-27	g__norank_f__norank_o__mle1-27	s__unclassified_g__norank_f__norank_o__mle1-27	OTU2570
d__Bacteria	k__norank_d__Bacteria	p__Patescibacteria	c__Saccharimonadia	o__Saccharimonadales	f__norank_o__Saccharimonadales	g__norank_f__norank_o__Saccharimonadales	s__unclassified_g__norank_f__norank_o__Saccharimonadales	OTU987
d__Bacteria	k__norank_d__Bacteria	p__Cyanobacteria	c__Cyanobacteriia	o__Chloroplast	f__norank_o__Chloroplast	g__norank_f__norank_o__Chloroplast	s__Desmochloris_halophila	OTU2927
d__Bacteria	k__norank_d__Bacteria	p__Proteobacteria	c__Alphaproteobacteria	o__Rhizobiales	f__Beijerinckiaceae	g__1174-901-12	s__unclassified_g__1174-901-12	OTU1878
d__Bacteria	k__norank_d__Bacteria	p__Chloroflexi	c__Anaerolineae	o__Caldilineales	f__Caldilineaceae	g__norank_f__Caldilineaceae	s__unclassified_g__norank_f__Caldilineaceae	OTU2516
d__Bacteria	k__norank_d__Bacteria	p__Patescibacteria	c__Saccharimonadia	o__Saccharimonadales	f__LWQ8	g__norank_f__LWQ8	s__unclassified_g__norank_f__LWQ8	OTU1043
d__Bacteria	k__norank_d__Bacteria	p__Patescibacteria	c__Parcubacteria	o__Candidatus_Kaiserbacteria	f__norank_o__Candidatus_Kaiserbacteria	g__norank_f__norank_o__Candidatus_Kaiserbacteria	s__metagenome_g__norank_f__norank_o__Candidatus_Kaiserbacteria	OTU2727
d__Bacteria	k__norank_d__Bacteria	p__Bdellovibrionota	c__Oligoflexia	o__0319-6G20	f__norank_o__0319-6G20	g__norank_f__norank_o__0319-6G20	s__uncultured_bacterium_g__norank_f__norank_o__0319-6G20	OTU755
d__Bacteria	k__norank_d__Bacteria	p__Acidobacteriota	c__Acidobacteriae	o__Subgroup_2	f__norank_o__Subgroup_2	g__norank_f__norank_o__Subgroup_2	s__unclassified_g__norank_f__norank_o__Subgroup_2	OTU1788
d__Bacteria	k__norank_d__Bacteria	p__unclassified_k__norank_d__Bacteria	c__unclassified_k__norank_d__Bacteria	o__unclassified_k__norank_d__Bacteria	f__unclassified_k__norank_d__Bacteria	g__unclassified_k__norank_d__Bacteria	s__unclassified_k__norank_d__Bacteria	OTU2001

d__Bacteria	k__norank_ d__Bacteria	p__Actinobacteriota	c__Thermoleophilia	o__Gaiellales	f__norank_o__Gaiellales	g__norank_f__norank_o__Gaiellales	s__metagenome_g__norank_f__norank_o__Gaiellales	OTU2183
d__Bacteria	k__norank_ d__Bacteria	p__Patescibacteria	c__Saccharimonadia	o__Saccharimonadales	f__unclassified_o__Saccharimonadales	g__unclassified_o__Saccharimonadale s	s__unclassified_o__Saccharimonadales	OTU2653
d__Bacteria	k__norank_ d__Bacteria	p__Armatimonadota	c__Chthonomonadetes	o__Chthonomonadales	f__norank_o__Chthonomonadales	g__norank_f__norank_o__Chthonomo nadales	s__uncultured_bacterium_g__norank_f__norank_o__Chthonomonadales	OTU1145
d__Bacteria	k__norank_ d__Bacteria	p__Chloroflexi	c__Ktedonobacteria	o__C0119	f__norank_o__C0119	g__norank_f__norank_o__C0119	s__uncultured_bacterium_g__norank_f__norank_o__C0119	OTU1225
d__Bacteria	k__norank_ d__Bacteria	p__Bacteroidota	c__Bacteroidia	o__Cytophagales	f__Hymenobacteraceae	g__Adhaeribacter	s__Hymenobacter_sp._29F	OTU2788
d__Bacteria	k__norank_ d__Bacteria	p__Proteobacteria	c__Gammaproteobacteria	o__Diplorickettsiales	f__Diplorickettsiaceae	g__Aquicella	s__unclassified_g__Aquicella	OTU1083
d__Bacteria	k__norank_ d__Bacteria	p__Proteobacteria	c__Alphaproteobacteria	o__Elsterales	f__norank_o__Elsterales	g__norank_f__norank_o__Elsterales	s__metagenome_g__norank_f__norank_o__Elsterales	OTU632
d__Bacteria	k__norank_ d__Bacteria	p__Actinobacteriota	c__Thermoleophilia	o__Solirubrobacterales	f__Solirubrobacteraceae	g__norank_f__Solirubrobacteraceae	s__Solirubrobacterales_bacterium_g__norank	OTU2425
d__Bacteria	k__norank_ d__Bacteria	p__Bacteroidota	c__Bacteroidia	o__Chitinophagales	f__Chitinophagaceae	g__Ferruginibacter	s__uncultured_Bacteroidetes_bacterium_g__Ferruginibacter	OTU2275
d__Bacteria	k__norank_ d__Bacteria	p__Chloroflexi	c__Ktedonobacteria	o__C0119	f__norank_o__C0119	g__norank_f__norank_o__C0119	s__uncultured_Chloroflexi_bacterium_g__norank_f__norank_o__C0119	OTU1844
d__Bacteria	k__norank_ d__Bacteria	p__Patescibacteria	c__Saccharimonadia	o__Saccharimonadales	f__norank_o__Saccharimonadales	g__norank_f__norank_o__Saccharimo nadales	s__unclassified_g__norank_f__norank_o__Saccharimonadales	OTU2520
d__Bacteria	k__norank_ d__Bacteria	p__Deinococcota	c__Deinococci	o__Deinococcales	f__Deinococcaceae	g__Deinococcus	s__unclassified_g__Deinococcus	OTU1782
d__Bacteria	k__norank_ d__Bacteria	p__Actinobacteriota	c__Actinobacteria	o__Frankiales	f__norank_o__Frankiales	g__norank_f__norank_o__Frankiales	s__unclassified_g__norank_f__norank_o__Frankiales	OTU87
d__Bacteria	k__norank_ d__Bacteria	p__Deinococcota	c__Deinococci	o__Deinococcales	f__Deinococcaceae	g__Deinococcus	s__unclassified_g__Deinococcus	OTU1784
d__Bacteria	k__norank_ d__Bacteria	p__Armatimonadota	c__norank_p__Armatimon adota	o__norank_c__norank_p__ Armatimonadota	f__norank_o__norank_c__norank_p__Ar matimonadota	g__norank_f__norank_o__norank_c__ norank_p__Armatimonadota	s__unclassified_g__norank_f__norank_o__norank_c__norank_p__Armatimonadota	OTU733
d__Bacteria	k__norank_ d__Bacteria	p__Bacteroidota	c__Bacteroidia	o__Sphingobacteriales	f__Sphingobacteriaceae	g__Pedobacter	s__unclassified_g__Pedobacter	OTU215

d__Bacteria	k__norank_ d__Bacteria	p__Deinococcota	c__Deinococci	o__Deinococcales	f__Deinococcaceae	g__norank_f__Deinococcaceae	s__uncultured_bacterium_g__norank_f__Deinococcaceae	OTU1205
d__Bacteria	k__norank_ d__Bacteria	p__Proteobacteria	c__Alphaproteobacteria	o__Rickettsiales	f__Mitochondria	g__norank_f__Mitochondria	s__unclassified_g__norank_f__Mitochondria	OTU861
d__Bacteria	k__norank_ d__Bacteria	p__Myxococcota	c__Polyangia	o__Polyangiales	f__Sandaracinaceae	g__norank_f__Sandaracinaceae	s__metagenome_g__norank_f__Sandaracinaceae	OTU279
d__Bacteria	k__norank_ d__Bacteria	p__WPS-2	c__norank_p__WPS-2	o__norank_c__norank_p__ WPS-2	f__norank_o__norank_c__norank_p__W PS-2	g__norank_f__norank_o__norank_c__ norank_p__WPS-2	s__uncultured_bacterium_g__norank_f__norank_o__norank_c__norank_p__WPS-2	OTU1975
d__Bacteria	k__norank_ d__Bacteria	p__Nitrospirota	c__Nitrospiria	o__Nitrospirales	f__Nitrospiraceae	g__Nitrospira	s__unclassified_g__Nitrospira	OTU696
d__Bacteria	k__norank_ d__Bacteria	p__Planctomycetota	c__BD7-11	o__norank_c__BD7-11	f__norank_o__norank_c__BD7-11	g__norank_f__norank_o__norank_c__ BD7-11	s__uncultured_bacterium_g__norank_f__norank_o__norank_c__BD7-11	OTU848
d__Bacteria	k__norank_ d__Bacteria	p__Proteobacteria	c__Gammaproteobacteria	o__Coxiellales	f__Coxiellaceae	g__Coxiella	s__unclassified_g__Coxiella	OTU1132
d__Bacteria	k__norank_ d__Bacteria	p__Patescibacteria	c__Saccharimonadia	o__Saccharimonadales	f__norank_o__Saccharimonadales	g__norank_f__norank_o__Saccharimo nadales	s__unclassified_g__norank_f__norank_o__Saccharimonadales	OTU370
d__Bacteria	k__norank_ d__Bacteria	p__Firmicutes	c__Bacilli	o__Bacillales	f__Bacillaceae	g__Bacillus	s__Bacillus_nealsonii	OTU1283
d__Bacteria	k__norank_ d__Bacteria	p__Actinobacteriota	c__Actinobacteria	o__Frankiales	f__norank_o__Frankiales	g__norank_f__norank_o__Frankiales	s__uncultured_bacterium_g__norank_f__norank_o__Frankiales	OTU1999
d__Bacteria	k__norank_ d__Bacteria	p__Proteobacteria	c__Gammaproteobacteria	o__Xanthomonadales	f__Rhodanobacteraceae	g__Ahniella	s__uncultured_bacterium_g__Ahniella	OTU2054
d__Bacteria	k__norank_ d__Bacteria	p__Actinobacteriota	c__Acidimicrobiia	o__norank_c__Acidimicrobi ia	f__norank_o__norank_c__Acidimicrobiia	g__norank_f__norank_o__norank_c__ Acidimicrobiia	s__uncultured_Acidimicrobiia_bacterium_g__norank	OTU1111
d__Bacteria	k__norank_ d__Bacteria	p__Patescibacteria	c__Saccharimonadia	o__Saccharimonadales	f__LWQ8	g__norank_f__LWQ8	s__unclassified_g__norank_f__LWQ8	OTU1106
d__Bacteria	k__norank_ d__Bacteria	p__Gemmatimonadota	c__Gemmatimonadetes	o__Gemmatimonadales	f__Gemmatimonadaceae	g__Gemmatimonas	s__uncultured_bacterium_g__Gemmatimonas	OTU1005
d__Bacteria	k__norank_ d__Bacteria	p__Bacteroidota	c__Bacteroidia	o__Flavobacteriales	f__Weeksellaceae	g__Chryseobacterium	s__unclassified_g__Chryseobacterium	OTU2969
d__Bacteria	k__norank_ d__Bacteria	p__Actinobacteriota	c__Actinobacteria	o__Propionibacteriales	f__Nocardioidaceae	g__Nocardioides	s__uncultured_bacterium_g__Nocardioides	OTU291

d__Bacteria	k__norank_d__Bacteria	p__Myxococcota	c__Myxococcia	o__Myxococcales	f__Anaeromyxobacteraceae	g__Anaeromyxobacter	s__unclassified_g__Anaeromyxobacter	OTU921
d__Bacteria	k__norank_d__Bacteria	p__Acidobacteriota	c__Vicinamibacteria	o__Subgroup_17	f__norank_o__Subgroup_17	g__norank_f__norank_o__Subgroup_17	s__unclassified_g__norank_f__norank_o__Subgroup_17	OTU799
d__Bacteria	k__norank_d__Bacteria	p__Chloroflexi	c__Chloroflexia	o__Thermomicrobiales	f__JG30-KF-CM45	g__norank_f__JG30-KF-CM45	s__uncultured_bacterium_g__norank_f__JG30-KF-CM45	OTU2501
d__Bacteria	k__norank_d__Bacteria	p__Chloroflexi	c__JG30-KF-CM66	o__norank_c__JG30-KF-CM66	f__norank_o__norank_c__JG30-KF-CM66	g__norank_f__norank_o__norank_c__JG30-KF-CM66	s__uncultured_Armatimonadetes_bacterium_g__norank_f__norank_o__norank	OTU1893
d__Bacteria	k__norank_d__Bacteria	p__Cyanobacteria	c__Cyanobacteriia	o__Chloroplast	f__norank_o__Chloroplast	g__norank_f__norank_o__Chloroplast	s__unclassified_g__norank_f__norank_o__Chloroplast	OTU1031
d__Bacteria	k__norank_d__Bacteria	p__Planctomycetota	c__Planctomycetes	o__Isosphaerales	f__Isosphaeraceae	g__unclassified_f__Isosphaeraceae	s__unclassified_f__Isosphaeraceae	OTU1159
d__Bacteria	k__norank_d__Bacteria	p__Acidobacteriota	c__Blastocatellia	o__Blastocatellales	f__Blastocatellaceae	g__norank_f__Blastocatellaceae	s__uncultured_Acidobacteria_bacterium_g__norank_f__Blastocatellaceae	OTU2905
d__Bacteria	k__norank_d__Bacteria	p__Firmicutes	c__Bacilli	o__Alicyclobacillales	f__Alicyclobacillaceae	g__Tumebacillus	s__uncultured_bacterium_g__Tumebacillus	OTU1232
d__Bacteria	k__norank_d__Bacteria	p__Myxococcota	c__Polyangia	o__Polyangiales	f__Sandaracinaceae	g__norank_f__Sandaracinaceae	s__uncultured_soil_bacterium_g__norank_f__Sandaracinaceae	OTU2388
d__Bacteria	k__norank_d__Bacteria	p__Proteobacteria	c__Gammaproteobacteria	o__Burkholderiales	f__Comamonadaceae	g__Ideonella	s__uncultured_bacterium_g__Ideonella	OTU2539
d__Bacteria	k__norank_d__Bacteria	p__Chloroflexi	c__P2-11E	o__norank_c__P2-11E	f__norank_o__norank_c__P2-11E	g__norank_f__norank_o__norank_c__P2-11E	s__uncultured_bacterium_g__norank_f__norank_o__norank_c__P2-11E	OTU1104
d__Bacteria	k__norank_d__Bacteria	p__Verrucomicrobiota	c__Verrucomicrobiae	o__Pedosphaerales	f__Pedosphaeraceae	g__norank_f__Pedosphaeraceae	s__unclassified_g__norank_f__Pedosphaeraceae	OTU959
d__Bacteria	k__norank_d__Bacteria	p__Chloroflexi	c__Ktedonobacteria	o__C0119	f__norank_o__C0119	g__norank_f__norank_o__C0119	s__uncultured_Chloroflexi_bacterium_g__norank_f__norank_o__C0119	OTU1934
d__Bacteria	k__norank_d__Bacteria	p__Planctomycetota	c__Phycisphaerae	o__Phycisphaerales	f__Phycisphaeraceae	g__norank_f__Phycisphaeraceae	s__metagenome_g__norank_f__Phycisphaeraceae	OTU515
d__Bacteria	k__norank_d__Bacteria	p__Bdellovibrionota	c__Bdellovibrionia	o__Bdellovibrionales	f__Bdellovibrionaceae	g__Bdellovibrio	s__uncultured_soil_bacterium_g__Bdellovibrio	OTU1652
d__Bacteria	k__norank_d__Bacteria	p__Myxococcota	c__Polyangia	o__Haliangiales	f__Haliangiaceae	g__Haliangium	s__uncultured_delta_proteobacterium_g__Haliangium	OTU2591

d__Bacteria	k__norank_d__Bacteria	p__Acidobacteriota	c__Blastocatellia	o__Blastocatellales	f__Blastocatellaceae	g__JGI_0001001-H03	s__unclassified_g__JGI_0001001-H03	OTU2313
d__Bacteria	k__norank_d__Bacteria	p__Proteobacteria	c__Gammaproteobacteria	o__Burkholderiales	f__TRA3-20	g__norank_f__TRA3-20	s__metagenome_g__norank_f__TRA3-20	OTU2495
d__Bacteria	k__norank_d__Bacteria	p__Proteobacteria	c__Alphaproteobacteria	o__Rhodobacterales	f__Rhodobacteraceae	g__unclassified_f__Rhodobacteraceae	s__unclassified_f__Rhodobacteraceae	OTU1118
d__Bacteria	k__norank_d__Bacteria	p__FCPU426	c__norank_p__FCPU426	o__norank_c__norank_p__FCPU426	f__norank_o__norank_c__norank_p__FCPU426	g__norank_f__norank_o__norank_c__norank_p__FCPU426	s__metagenome_g__norank_f__norank_o__norank_c__norank_p__FCPU426	OTU934
d__Bacteria	k__norank_d__Bacteria	p__Myxococcota	c__Myxococcia	o__Myxococcales	f__Myxococcaceae	g__norank_f__Myxococcaceae	s__unclassified_g__norank_f__Myxococcaceae	OTU401
d__Bacteria	k__norank_d__Bacteria	p__Actinobacteriota	c__Acidimicrobiia	o__norank_c__Acidimicrobiia	f__norank_o__norank_c__Acidimicrobiia	g__norank_f__norank_o__norank_c__Acidimicrobiia	s__metagenome_g__norank_f__norank_o__norank_c__Acidimicrobiia	OTU1701
d__Bacteria	k__norank_d__Bacteria	p__Chloroflexi	c__TK10	o__norank_c__TK10	f__norank_o__norank_c__TK10	g__norank_f__norank_o__norank_c__TK10	s__unclassified_g__norank_f__norank_o__norank_c__TK10	OTU1199
d__Bacteria	k__norank_d__Bacteria	p__Acidobacteriota	c__Acidobacteriae	o__Acidobacteriales	f__Acidobacteriaceae_Subgroup_1	g__Granulicella	s__unclassified_g__Granulicella	OTU1976
d__Bacteria	k__norank_d__Bacteria	p__Myxococcota	c__Polyangia	o__Blfdi19	f__norank_o__Blfdi19	g__norank_f__norank_o__Blfdi19	s__uncultured_bacterium_g__norank_f__norank_o__Blfdi19	OTU745
d__Bacteria	k__norank_d__Bacteria	p__Proteobacteria	c__Alphaproteobacteria	o__Acetobacterales	f__Acetobacteraceae	g__unclassified_f__Acetobacteraceae	s__unclassified_f__Acetobacteraceae	OTU1312
d__Bacteria	k__norank_d__Bacteria	p__Bacteroidota	c__Bacteroidia	o__Chitinophagales	f__Chitinophagaceae	g__Segetibacter	s__uncultured_bacterium_g__Segetibacter	OTU1593
d__Bacteria	k__norank_d__Bacteria	p__Verrucomicrobiota	c__Verrucomicrobiae	o__Chthoniobacterales	f__Chthoniobacteraceae	g__Chthoniobacter	s__uncultured_bacterium_g__Chthoniobacter	OTU2667
d__Bacteria	k__norank_d__Bacteria	p__Bacteroidota	c__Bacteroidia	o__Bacteroidales	f__Prevotellaceae	g__Prevotella	s__Prevotella_melaninogenica	OTU652
d__Bacteria	k__norank_d__Bacteria	p__Patescibacteria	c__Saccharimonadia	o__Saccharimonadales	f__LWQ8	g__norank_f__LWQ8	s__uncultured_Candidatus_Saccharibacteria_bacterium_g__norank_f__LWQ8	OTU1101
d__Bacteria	k__norank_d__Bacteria	p__Acidobacteriota	c__Vicinamibacteria	o__Vicinamibacterales	f__norank_o__Vicinamibacterales	g__norank_f__norank_o__Vicinamibacterales	s__uncultured_bacterium_g__norank_f__norank_o__Vicinamibacterales	OTU517
d__Bacteria	k__norank_d__Bacteria	p__Chloroflexi	c__JG30-KF-CM66	o__norank_c__JG30-KF-CM66	f__norank_o__norank_c__JG30-KF-CM66	g__norank_f__norank_o__norank_c__JG30-KF-CM66	s__uncultured_Chloroflexi_bacterium_g__norank_f__norank_o__norank_c__JG30-KF-CM66	OTU649

d__Bacteria	k__norank_ d__Bacteria	p__Verrucomicrobiota	c__Chlamydiae	o__Chlamydiales	f__Simkaniaceae	g__norank_f__Simkaniaceae	s__unclassified_g__norank_f__Simkaniaceae	OTU1299
d__Bacteria	k__norank_ d__Bacteria	p__Chloroflexi	c__Chloroflexia	o__Thermomicrobiales	f__JG30-KF-CM45	g__norank_f__JG30-KF-CM45	s__unclassified_g__norank_f__JG30-KF-CM45	OTU2234
d__Bacteria	k__norank_ d__Bacteria	p__Patescibacteria	c__Saccharimonadia	o__Saccharimonadales	f__WWH38	g__norank_f__WWH38	s__uncultured_bacterium_g__norank_f__WWH38	OTU910
d__Bacteria	k__norank_ d__Bacteria	p__Acidobacteriota	c__Blastocatellia	o__Pyrinomonadales	f__Pyrinomonadaceae	g__RB41	s__uncultured_bacterium_g__RB41	OTU1712
d__Bacteria	k__norank_ d__Bacteria	p__Chloroflexi	c__OLB14	o__norank_c__OLB14	f__norank_o__norank_c__OLB14	g__norank_f__norank_o__norank_c__ OLB14	s__metagenome_g__norank_f__norank_o__norank_c__OLB14	OTU673
d__Bacteria	k__norank_ d__Bacteria	p__Acidobacteriota	c__Vicinamibacteria	o__Vicinamibacterales	f__Vicinamibacteraceae	g__norank_f__Vicinamibacteraceae	s__uncultured_Acidobacteria_bacterium_g__norank_f__Vicinamibacteraceae	OTU2262
d__Bacteria	k__norank_ d__Bacteria	p__Proteobacteria	c__Gammaproteobacteria	o__Burkholderiales	f__Neisseriaceae	g__norank_f__Neisseriaceae	s__metagenome_g__norank_f__Neisseriaceae	OTU698
d__Bacteria	k__norank_ d__Bacteria	p__Chloroflexi	c__Ktedonobacteria	o__Ktedonobacterales	f__Ktedonobacteraceae	g__JG30a-KF-32	s__uncultured_Ktedobacteria_bacterium_g__JG30a-KF-32	OTU1761
d__Bacteria	k__norank_ d__Bacteria	p__Proteobacteria	c__Alphaproteobacteria	o__Rhizobiales	f__Xanthobacteraceae	g__norank_f__Xanthobacteraceae	s__unclassified_g__norank_f__Xanthobacteraceae	OTU1265
d__Bacteria	k__norank_ d__Bacteria	p__Firmicutes	c__Bacilli	o__Bacillales	f__Planococcaceae	g__Lysinibacillus	s__Lysinibacillus_massiliensis	OTU1501
d__Bacteria	k__norank_ d__Bacteria	p__Bacteroidota	c__Bacteroidia	o__Sphingobacteriales	f__Sphingobacteriaceae	g__unclassified_f__Sphingobacteriacea e	s__unclassified_f__Sphingobacteriaceae	OTU1194
d__Bacteria	k__norank_ d__Bacteria	p__Gemmatimonadota	c__Gemmatimonadetes	o__Gemmatimonadales	f__Gemmatimonadaceae	g__norank_f__Gemmatimonadaceae	s__uncultured_Gemmatimonadetes_bacterium_g__norank_f__Gemmatimonadaceae	OTU543
d__Bacteria	k__norank_ d__Bacteria	p__Firmicutes	c__Bacilli	o__Lactobacillales	f__Carnobacteriaceae	g__Trichococcus	s__unclassified_g__Trichococcus	OTU2317
d__Bacteria	k__norank_ d__Bacteria	p__Acidobacteriota	c__Holophagae	o__Subgroup_7	f__norank_o__Subgroup_7	g__norank_f__norank_o__Subgroup_7	s__uncultured_Acidobacteriales_bacterium_g__norank_f__norank_o__Subgroup_7	OTU822
d__Bacteria	k__norank_ d__Bacteria	p__Proteobacteria	c__Alphaproteobacteria	o__Rhizobiales	f__A0839	g__norank_f__A0839	s__uncultured_bacterium_g__norank_f__A0839	OTU602
d__Bacteria	k__norank_ d__Bacteria	p__Actinobacteriota	c__Actinobacteria	o__Micrococcales	f__Intrasporangiaceae	g__Ornithinimicrobium	s__unclassified_g__Ornithinimicrobium	OTU475

d__Bacteria	k__norank_d__Bacteria	p__Planctomycetota	c__Phycisphaerae	o__Phycisphaerales	f__Phycisphaeraceae	g__SM1A02	s__metagenome_g__SM1A02	OTU2145
d__Bacteria	k__norank_d__Bacteria	p__Cyanobacteria	c__Cyanobacteriia	o__Cyanobacteriales	f__Phormidiaceae	g__Tychonema_CCAP_1459-11B	s__uncultured_bacterium_g__Tychonema_CCAP_1459-11B	OTU2212
d__Bacteria	k__norank_d__Bacteria	p__Actinobacteriota	c__Actinobacteria	o__unclassified_c__Actinobacteria	f__unclassified_c__Actinobacteria	g__unclassified_c__Actinobacteria	s__unclassified_c__Actinobacteria	OTU2862
d__Bacteria	k__norank_d__Bacteria	p__Actinobacteriota	c__Acidimicrobiia	o__unclassified_c__Acidimicrobiia	f__unclassified_c__Acidimicrobiia	g__unclassified_c__Acidimicrobiia	s__unclassified_c__Acidimicrobiia	OTU2000
d__Bacteria	k__norank_d__Bacteria	p__Verrucomicrobiota	c__Verrucomicrobiae	o__Chthoniobacterales	f__Chthoniobacteraceae	g__Candidatus_Udaeobacter	s__uncultured_Verrucomicrobia_bacterium_g__Candidatus_Udaeobacter	OTU792
d__Bacteria	k__norank_d__Bacteria	p__Elusimicrobiota	c__Lineage_IIb	o__norank_c__Lineage_IIb	f__norank_o__norank_c__Lineage_IIb	g__norank_f__norank_o__norank_c__Lineage_IIb	s__uncultured_soil_bacterium_g__norank_f__norank_o__norank_c__Lineage_IIb	OTU1819
d__Bacteria	k__norank_d__Bacteria	p__Myxococcota	c__Polyangia	o__Polyangiales	f__Polyangiaceae	g__Pajaroellobacter	s__uncultured_bacterium_g__Pajaroellobacter	OTU1810
d__Bacteria	k__norank_d__Bacteria	p__Myxococcota	c__Polyangia	o__Polyangiales	f__Polyangiaceae	g__Pajaroellobacter	s__uncultured_bacterium_g__Pajaroellobacter	OTU1928
d__Bacteria	k__norank_d__Bacteria	p__Proteobacteria	c__Gammaproteobacteria	o__R7C24	f__norank_o__R7C24	g__norank_f__norank_o__R7C24	s__metagenome_g__norank_f__norank_o__R7C24	OTU1355
d__Bacteria	k__norank_d__Bacteria	p__Actinobacteriota	c__Acidimicrobiia	o__Microtrichales	f__Microtrichaceae	g__norank_f__Microtrichaceae	s__metagenome_g__norank_f__Microtrichaceae	OTU1105
d__Bacteria	k__norank_d__Bacteria	p__Gemmatimonadota	c__Longimicrobia	o__Longimicrobiales	f__Longimicrobiaceae	g__norank_f__Longimicrobiaceae	s__unclassified_g__norank_f__Longimicrobiaceae	OTU2168
d__Bacteria	k__norank_d__Bacteria	p__Myxococcota	c__Polyangia	o__Blfdi19	f__norank_o__Blfdi19	g__norank_f__norank_o__Blfdi19	s__metagenome_g__norank_f__norank_o__Blfdi19	OTU551
d__Bacteria	k__norank_d__Bacteria	p__Chloroflexi	c__Ktedonobacteria	o__B10-SB3A	f__norank_o__B10-SB3A	g__norank_f__norank_o__B10-SB3A	s__uncultured_bacterium_g__norank_f__norank_o__B10-SB3A	OTU983
d__Bacteria	k__norank_d__Bacteria	p__Chloroflexi	c__AD3	o__norank_c__AD3	f__norank_o__norank_c__AD3	g__norank_f__norank_o__norank_c__AD3	s__uncultured_bacterium_g__norank_f__norank_o__norank_c__AD3	OTU1746
d__Bacteria	k__norank_d__Bacteria	p__Myxococcota	c__Polyangia	o__Polyangiales	f__Polyangiaceae	g__Pajaroellobacter	s__uncultured_bacterium_g__Pajaroellobacter	OTU1925
d__Bacteria	k__norank_d__Bacteria	p__Actinobacteriota	c__Thermoleophilia	o__Solirubrobacterales	f__67-14	g__norank_f__67-14	s__uncultured_soil_bacterium_g__norank_f__67-14	OTU739

d__Bacteria	k__norank_ d__Bacteria	p__Patescibacteria	c__Saccharimonadia	o__Saccharimonadales	f__LWQ8	g__norank_f__LWQ8	s__uncultured_bacterium_g__norank_f__LWQ8	OTU960
d__Bacteria	k__norank_ d__Bacteria	p__Elusimicrobiota	c__Elusimicrobia	o__Lineage_IV	f__norank_o__Lineage_IV	g__norank_f__norank_o__Lineage_IV	s__uncultured_soil_bacterium_g__norank_f__norank_o__Lineage_IV	OTU1093
d__Bacteria	k__norank_ d__Bacteria	p__Proteobacteria	c__Gammaproteobacteria	o__Xanthomonadales	f__Xanthomonadaceae	g__Pseudoxanthomonas	s__uncultured_bacterium_g__Pseudoxanthomonas	OTU2811
d__Bacteria	k__norank_ d__Bacteria	p__Bacteroidota	c__Bacteroidia	o__Chitinophagales	f__Chitinophagaceae	g__Edaphobaculum	s__unclassified_g__Edaphobaculum	OTU2549
d__Bacteria	k__norank_ d__Bacteria	p__Cyanobacteria	c__Sericytochromatia	o__norank_c__Sericytochro matia	f__norank_o__norank_c__Sericytochrom atia	g__norank_f__norank_o__norank_c__ Sericytochromatia	s__unclassified_g__norank_f__norank_o__norank_c__Sericytochromatia	OTU204
d__Bacteria	k__norank_ d__Bacteria	p__Verrucomicrobiota	c__Chlamydiae	o__Chlamydiales	f__Parachlamydiaceae	g__Neochlamydia	s__metagenome_g__Neochlamydia	OTU1982
d__Bacteria	k__norank_ d__Bacteria	p__Cyanobacteria	c__Sericytochromatia	o__norank_c__Sericytochro matia	f__norank_o__norank_c__Sericytochrom atia	g__norank_f__norank_o__norank_c__ Sericytochromatia	s__unclassified_g__norank_f__norank_o__norank_c__Sericytochromatia	OTU201
d__Bacteria	k__norank_ d__Bacteria	p__Myxococcota	c__Polyangia	o__Polyangiales	f__Polyangiaceae	g__Pajaroellobacter	s__metagenome_g__Pajaroellobacter	OTU658
d__Bacteria	k__norank_ d__Bacteria	p__Deinococcota	c__Deinococci	o__Deinococcales	f__Deinococcaceae	g__Deinococcus	s__uncultured_bacterium_g__Deinococcus	OTU2
d__Bacteria	k__norank_ d__Bacteria	p__unclassified_k__no rank_d__Bacteria	c__unclassified_k__noran k_d__Bacteria	o__unclassified_k__norank_ d__Bacteria	f__unclassified_k__norank_d__Bacteria	g__unclassified_k__norank_d__Bacteri a	s__unclassified_k__norank_d__Bacteria	OTU1231
d__Bacteria	k__norank_ d__Bacteria	p__Proteobacteria	c__Gammaproteobacteria	o__Burkholderiales	f__Burkholderiaceae	g__Burkholderia-Caballeronia-Parabur kholderia	s__Paraburkholderia_ferrariae	OTU115
d__Bacteria	k__norank_ d__Bacteria	p__Proteobacteria	c__Gammaproteobacteria	o__Burkholderiales	f__A21b	g__norank_f__A21b	s__unclassified_g__norank_f__A21b	OTU2557
d__Bacteria	k__norank_ d__Bacteria	p__Chloroflexi	c__Chloroflexia	o__Kallotenuales	f__AKIW781	g__norank_f__AKIW781	s__uncultured_bacterium_g__norank_f__AKIW781	OTU320
d__Bacteria	k__norank_ d__Bacteria	p__Actinobacteriota	c__Acidimicrobiia	o__Microtrichales	f__norank_o__Microtrichales	g__norank_f__norank_o__Microtrichal es	s__wastewater_metagenome_g__norank_f__norank_o__Microtrichales	OTU2156
d__Bacteria	k__norank_ d__Bacteria	p__Proteobacteria	c__Gammaproteobacteria	o__Burkholderiales	f__A21b	g__norank_f__A21b	s__unclassified_g__norank_f__A21b	OTU2552
d__Bacteria	k__norank_ d__Bacteria	p__Actinobacteriota	c__Thermoleophilia	o__Solirubrobacterales	f__Solirubrobacteraceae	g__Solirubrobacter	s__uncultured_actinobacterium_g__Solirubrobacter	OTU2480

d__Bacteria	k__norank_d__Bacteria	p__Proteobacteria	c__Gammaproteobacteria	o__Diplorickettsiales	f__Diplorickettsiaceae	g__Aquicella	s__unclassified_g__Aquicella	OTU732
d__Bacteria	k__norank_d__Bacteria	p__Actinobacteriota	c__Thermoleophilia	o__Gaiellales	f__norank_o__Gaiellales	g__norank_f__norank_o__Gaiellales	s__unclassified_g__norank_f__norank_o__Gaiellales	OTU121
d__Bacteria	k__norank_d__Bacteria	p__Bacteroidota	c__Bacteroidia	o__Cytophagales	f__Microscillaceae	g__Ohtaekwangia	s__unclassified_g__Ohtaekwangia	OTU2386
d__Bacteria	k__norank_d__Bacteria	p__Chloroflexi	c__Ktedonobacteria	o__C0119	f__norank_o__C0119	g__norank_f__norank_o__C0119	s__uncultured_candidate_division_SAM_bacterium	OTU489
d__Bacteria	k__norank_d__Bacteria	p__Chloroflexi	c__OLB14	o__norank_c__OLB14	f__norank_o__norank_c__OLB14	g__norank_f__norank_o__norank_c__OLB14	s__uncultured_bacterium_g__norank_f__norank_o__norank_c__OLB14	OTU1617
d__Bacteria	k__norank_d__Bacteria	p__Proteobacteria	c__Gammaproteobacteria	o__Burkholderiales	f__Methylophilaceae	g__Methylotenera	s__uncultured_bacterium_g__Methylotenera	OTU2366
d__Bacteria	k__norank_d__Bacteria	p__Planctomycetota	c__Planctomycetes	o__Gemmatales	f__Gemmataceae	g__norank_f__Gemmataceae	s__uncultured_bacterium_g__norank_f__Gemmataceae	OTU1030
d__Bacteria	k__norank_d__Bacteria	p__Chloroflexi	c__AD3	o__norank_c__AD3	f__norank_o__norank_c__AD3	g__norank_f__norank_o__norank_c__AD3	s__unclassified_g__norank_f__norank_o__norank_c__AD3	OTU1791
d__Bacteria	k__norank_d__Bacteria	p__Patescibacteria	c__Saccharimonadia	o__Saccharimonadales	f__unclassified_o__Saccharimonadales	g__unclassified_o__Saccharimonadales	s__unclassified_o__Saccharimonadales	OTU1072
d__Bacteria	k__norank_d__Bacteria	p__Actinobacteriota	c__Actinobacteria	o__Corynebacteriales	f__Segniliparaceae	g__norank_f__Segniliparaceae	s__Rhodococcus_sp._MN8-9	OTU211
d__Bacteria	k__norank_d__Bacteria	p__Patescibacteria	c__Berkelbacteria	o__norank_c__Berkelbacteria	f__norank_o__norank_c__Berkelbacteria	g__norank_f__norank_o__norank_c__Berkelbacteria	s__Candidatus_Berkelbacteria_bacterium_CG2_30_43_20	OTU337
d__Bacteria	k__norank_d__Bacteria	p__Patescibacteria	c__Saccharimonadia	o__Saccharimonadales	f__WWH38	g__norank_f__WWH38	s__uncultured_bacterium_g__norank_f__WWH38	OTU1596
d__Bacteria	k__norank_d__Bacteria	p__Patescibacteria	c__Saccharimonadia	o__Saccharimonadales	f__LWQ8	g__norank_f__LWQ8	s__unclassified_g__norank_f__LWQ8	OTU2921
d__Bacteria	k__norank_d__Bacteria	p__Bacteroidota	c__Bacteroidia	o__Bacteroidales	f__Prevotellaceae	g__Prevotella	s__Prevotella_shahii	OTU403
d__Bacteria	k__norank_d__Bacteria	p__Chloroflexi	c__Gitt-GS-136	o__norank_c__Gitt-GS-136	f__norank_o__norank_c__Gitt-GS-136	g__norank_f__norank_o__norank_c__Gitt-GS-136	s__uncultured_bacterium_g__norank_f__norank_o__norank_c__Gitt-GS-136	OTU2427
d__Bacteria	k__norank_d__Bacteria	p__Elusimicrobiota	c__Elusimicrobia	o__Lineage_IV	f__norank_o__Lineage_IV	g__norank_f__norank_o__Lineage_IV	s__uncultured_bacterium_g__norank_f__norank_o__Lineage_IV	OTU760

d__Bacteria	k__norank_d__Bacteria	p__Bdellovibrionota	c__Oligoflexia	o__Oligoflexales	f__norank_o__Oligoflexales	g__norank_f__norank_o__Oligoflexales	s__uncultured_bacterium_g__norank_f__norank_o__Oligoflexales	OTU2695
d__Bacteria	k__norank_d__Bacteria	p__Bacteroidota	c__Bacteroidia	o__Bacteroidales	f__Porphyromonadaceae	g__Porphyromonas	s__uncultured_bacterium_g__Porphyromonas	OTU439
d__Bacteria	k__norank_d__Bacteria	p__Chloroflexi	c__Anaerolineae	o__unclassified_c__Anaerolineae	f__unclassified_c__Anaerolineae	g__unclassified_c__Anaerolineae	s__unclassified_c__Anaerolineae	OTU1831
d__Bacteria	k__norank_d__Bacteria	p__Armatimonadota	c__Chthonomonadetes	o__Chthonomonadales	f__norank_o__Chthonomonadales	g__norank_f__norank_o__Chthonomonadales	s__uncultured_bacterium_g__norank_f__norank_o__Chthonomonadales	OTU1137
d__Bacteria	k__norank_d__Bacteria	p__Proteobacteria	c__Alphaproteobacteria	o__Rhodobacterales	f__Rhodobacteraceae	g__unclassified_f__Rhodobacteraceae	s__unclassified_f__Rhodobacteraceae	OTU2149
d__Bacteria	k__norank_d__Bacteria	p__Chloroflexi	c__Chloroflexia	o__Elev-1554	f__norank_o__Elev-1554	g__norank_f__norank_o__Elev-1554	s__uncultured_bacterium_g__norank_f__norank_o__Elev-1554	OTU912
d__Bacteria	k__norank_d__Bacteria	p__Patescibacteria	c__Parcubacteria	o__Candidatus_Kaiserbacteria	f__norank_o__Candidatus_Kaiserbacteria	g__norank_f__norank_o__Candidatus_Kaiserbacteria	s__unclassified_g__norank_f__norank_o__Candidatus_Kaiserbacteria	OTU814
d__Bacteria	k__norank_d__Bacteria	p__Proteobacteria	c__Alphaproteobacteria	o__Acetobacterales	f__Acetobacteraceae	g__unclassified_f__Acetobacteraceae	s__unclassified_f__Acetobacteraceae	OTU492
d__Bacteria	k__norank_d__Bacteria	p__Bdellovibrionota	c__Oligoflexia	o__0319-6G20	f__norank_o__0319-6G20	g__norank_f__norank_o__0319-6G20	s__unclassified_g__norank_f__norank_o__0319-6G20	OTU2835
d__Bacteria	k__norank_d__Bacteria	p__Bdellovibrionota	c__Bdellovibrionia	o__Bacteriovoracales	f__Bacteriovoracaceae	g__Peredibacter	s__microbial_mat_metagenome_g__Peredibacter	OTU2499
d__Bacteria	k__norank_d__Bacteria	p__Proteobacteria	c__Gammaproteobacteria	o__Burkholderiales	f__Comamonadaceae	g__unclassified_f__Comamonadaceae	s__unclassified_f__Comamonadaceae	OTU731
d__Bacteria	k__norank_d__Bacteria	p__Actinobacteriota	c__Acidimicrobiia	o__Microtrichales	f__Iamiaceae	g__Iamia	s__metagenome_g__Iamia	OTU2259
d__Bacteria	k__norank_d__Bacteria	p__Gemmatimonadota	c__Gemmatimonadetes	o__Gemmatimonadales	f__Gemmatimonadaceae	g__Gemmatimonas	s__uncultured_bacterium_g__Gemmatimonas	OTU540
d__Bacteria	k__norank_d__Bacteria	p__Myxococcota	c__Myxococcia	o__Myxococcales	f__Anaeromyxobacteraceae	g__Anaeromyxobacter	s__uncultured_delta_proteobacterium_g__Anaeromyxobacter	OTU45
d__Bacteria	k__norank_d__Bacteria	p__Chloroflexi	c__Anaerolineae	o__SBR1031	f__A4b	g__norank_f__A4b	s__unclassified_g__norank_f__A4b	OTU619
d__Bacteria	k__norank_d__Bacteria	p__Planctomycetota	c__Planctomycetes	o__Gemmatales	f__Gemmataceae	g__norank_f__Gemmataceae	s__uncultured_bacterium_g__norank_f__Gemmataceae	OTU538

d__Bacteria	k__norank_d__Bacteria	p__Patescibacteria	c__Saccharimonadia	o__Saccharimonadales	f__LWQ8	g__norank_f__LWQ8	s__uncultured_bacterium_g__norank_f__LWQ8	OTU364
d__Bacteria	k__norank_d__Bacteria	p__Bacteroidota	c__Kapabacteria	o__Kapabacteriales	f__norank_o__Kapabacteriales	g__norank_f__norank_o__Kapabacteriales	s__unclassified_g__norank_f__norank_o__Kapabacteriales	OTU2607
d__Bacteria	k__norank_d__Bacteria	p__Patescibacteria	c__Saccharimonadia	o__Saccharimonadales	f__LWQ8	g__norank_f__LWQ8	s__uncultured_bacterium_g__norank_f__LWQ8	OTU361
d__Bacteria	k__norank_d__Bacteria	p__Bacteroidota	c__Bacteroidia	o__Cytophagales	f__Spirosomaceae	g__Rudanella	s__uncultured_bacterium_g__Rudanella	OTU2295
d__Bacteria	k__norank_d__Bacteria	p__Bacteroidota	c__Bacteroidia	o__Cytophagales	f__Hymenobacteraceae	g__Hymenobacter	s__unclassified_g__Hymenobacter	OTU131
d__Bacteria	k__norank_d__Bacteria	p__Acidobacteriota	c__Acidobacteriae	o__norank_c__Acidobacteriae	f__norank_o__norank_c__Acidobacteriae	g__Paludibaculum	s__uncultured_bacterium_g__Paludibaculum	OTU422
d__Bacteria	k__norank_d__Bacteria	p__Cyanobacteria	c__Cyanobacteriia	o__Chloroplast	f__norank_o__Chloroplast	g__norank_f__norank_o__Chloroplast	s__unclassified_g__norank_f__norank_o__Chloroplast	OTU23
d__Bacteria	k__norank_d__Bacteria	p__Patescibacteria	c__Saccharimonadia	o__Saccharimonadales	f__norank_o__Saccharimonadales	g__norank_f__norank_o__Saccharimonadales	s__unclassified_g__norank_f__norank_o__Saccharimonadales	OTU2235
d__Bacteria	k__norank_d__Bacteria	p__Myxococcota	c__Myxococcia	o__Myxococcales	f__Myxococcaceae	g__unclassified_f__Myxococcaceae	s__unclassified_f__Myxococcaceae	OTU2308
d__Bacteria	k__norank_d__Bacteria	p__Patescibacteria	c__Saccharimonadia	o__Saccharimonadales	f__LWQ8	g__norank_f__LWQ8	s__uncultured_soil_bacterium_g__norank_f__LWQ8	OTU1786
d__Bacteria	k__norank_d__Bacteria	p__Proteobacteria	c__Alphaproteobacteria	o__norank_c__Alphaproteobacteria	f__norank_o__norank_c__Alphaproteobacteria	g__norank_f__norank_o__norank_c__Alphaproteobacteria	s__uncultured_bacterium_g__norank_f__norank_o__norank_c__Alphaproteobacteria	OTU2182
d__Bacteria	k__norank_d__Bacteria	p__Chloroflexi	c__TK10	o__norank_c__TK10	f__norank_o__norank_c__TK10	g__norank_f__norank_o__norank_c__TK10	s__unclassified_g__norank_f__norank_o__norank_c__TK10	OTU2638
d__Bacteria	k__norank_d__Bacteria	p__Acidobacteriota	c__Vicinamibacteria	o__Vicinamibacterales	f__Vicinamibacteraceae	g__norank_f__Vicinamibacteraceae	s__unclassified_g__norank_f__Vicinamibacteraceae	OTU1575
d__Bacteria	k__norank_d__Bacteria	p__Elusimicrobiota	c__Elusimicrobia	o__Lineage_IV	f__norank_o__Lineage_IV	g__norank_f__norank_o__Lineage_IV	s__unclassified_g__norank_f__norank_o__Lineage_IV	OTU1023
d__Bacteria	k__norank_d__Bacteria	p__Gemmatimonadota	c__Gemmatimonadetes	o__Gemmatimonadales	f__Gemmatimonadaceae	g__norank_f__Gemmatimonadaceae	s__uncultured_bacterium_contig00007	OTU1646
d__Bacteria	k__norank_d__Bacteria	p__Proteobacteria	c__Gammaproteobacteria	o__Burkholderiales	f__Sutterellaceae	g__AAP99	s__uncultured_bacterium_g__AAP99	OTU46

d__Bacteria	k__norank_ d__Bacteria	p__Bacteroidota	c__Bacteroidia	o__Chitinophagales	f__Chitinophagaceae	g__norank_f__Chitinophagaceae	s__unclassified_g__norank_f__Chitinophagaceae	OTU1016
d__Bacteria	k__norank_ d__Bacteria	p__Bacteroidota	c__Bacteroidia	o__Flavobacteriales	f__Flavobacteriaceae	g__Flavobacterium	s__unclassified_g__Flavobacterium	OTU149
d__Bacteria	k__norank_ d__Bacteria	p__Actinobacteriota	c__Actinobacteria	o__Micrococcales	f__Intrasporangiaceae	g__unclassified_f__Intrasporangiaceae	s__unclassified_f__Intrasporangiaceae	OTU84
d__Bacteria	k__norank_ d__Bacteria	p__Acidobacteriota	c__Vicinamibacteria	o__Subgroup_17	f__norank_o__Subgroup_17	g__norank_f__norank_o__Subgroup_1 7	s__uncultured_Acidobacteria_bacterium_g__norank_f__norank_o__Subgroup_17	OTU1276
d__Bacteria	k__norank_ d__Bacteria	p__Verrucomicrobiota	c__Verrucomicrobiae	o__Chthoniobacterales	f__Xiphinematobacteraceae	g__Candidatus_Xiphinematobacter	s__uncultured_bacterium_g__Candidatus_Xiphinematobacter	OTU744
d__Bacteria	k__norank_ d__Bacteria	p__Chloroflexi	c__Chloroflexia	o__Thermomicrobiales	f__JG30-KF-CM45	g__norank_f__JG30-KF-CM45	s__uncultured_Chloroflexi_bacterium_g__norank_f__JG30-KF-CM45	OTU1971
d__Bacteria	k__norank_ d__Bacteria	p__Patescibacteria	c__Saccharimonadia	o__Saccharimonadales	f__norank_o__Saccharimonadales	g__norank_f__norank_o__Saccharimo nadales	s__unclassified_g__norank_f__norank_o__Saccharimonadales	OTU193
d__Bacteria	k__norank_ d__Bacteria	p__Proteobacteria	c__Gammaproteobacteria	o__Burkholderiales	f__Nitrosomonadaceae	g__MND1	s__unclassified_g__MND1	OTU2455
d__Bacteria	k__norank_ d__Bacteria	p__Acidobacteriota	c__Vicinamibacteria	o__Vicinamibacterales	f__norank_o__Vicinamibacterales	g__norank_f__norank_o__Vicinamiba cterales	s__unclassified_g__norank_f__norank_o__Vicinamibacterales	OTU1471
d__Bacteria	k__norank_ d__Bacteria	p__Acidobacteriota	c__Thermoanaerobaculia	o__Thermoanaerobaculales	f__Thermoanaerobaculaceae	g__Subgroup_10	s__uncultured_bacterium_g__Subgroup_10	OTU2559
d__Bacteria	k__norank_ d__Bacteria	p__Deinococcota	c__Deinococci	o__Deinococcales	f__Deinococcaceae	g__Deinococcus	s__Deinococcus_sp._2A5	OTU145
d__Bacteria	k__norank_ d__Bacteria	p__Acidobacteriota	c__Holophagae	o__Subgroup_7	f__norank_o__Subgroup_7	g__norank_f__norank_o__Subgroup_7	s__uncultured_soil_bacterium_g__norank_f__norank_o__Subgroup_7	OTU373
d__Bacteria	k__norank_ d__Bacteria	p__Bdellovibrionota	c__Oligoflexia	o__Oligoflexales	f__norank_o__Oligoflexales	g__norank_f__norank_o__Oligoflexale s	s__unclassified_g__norank_f__norank_o__Oligoflexales	OTU2661
d__Bacteria	k__norank_ d__Bacteria	p__Myxococcota	c__Myxococcia	o__Myxococcales	f__Myxococcaceae	g__norank_f__Myxococcaceae	s__uncultured_bacterium_g__norank_f__Myxococcaceae	OTU2830
d__Bacteria	k__norank_ d__Bacteria	p__Proteobacteria	c__Alphaproteobacteria	o__Rickettsiales	f__SM2D12	g__norank_f__SM2D12	s__metagenome_g__norank_f__SM2D12	OTU2802
d__Bacteria	k__norank_ d__Bacteria	p__Planctomycetota	c__Planctomycetes	o__Gemmatales	f__Gemmataceae	g__norank_f__Gemmataceae	s__unclassified_g__norank_f__Gemmataceae	OTU2697

d__Bacteria	k__norank_ d__Bacteria	p__Chloroflexi	c__Chloroflexia	o__Thermomicrobiales	f__JG30-KF-CM45	g__norank_f__JG30-KF-CM45	s__metagenome_g__norank_f__JG30-KF-CM45	OTU264
d__Bacteria	k__norank_ d__Bacteria	p__Bdellovibrionota	c__Bdellovibrionia	o__Bdellovibrionales	f__Bdellovibrionaceae	g__Bdellovibrio	s__uncultured_bacterium_g__Bdellovibrio	OTU2731
d__Bacteria	k__norank_ d__Bacteria	p__Bdellovibrionota	c__Bdellovibrionia	o__Bdellovibrionales	f__Bdellovibrionaceae	g__Bdellovibrio	s__uncultured_bacterium_g__Bdellovibrio	OTU1759
d__Bacteria	k__norank_ d__Bacteria	p__Bdellovibrionota	c__Oligoflexia	o__0319-6G20	f__norank_o__0319-6G20	g__norank_f__norank_o__0319-6G20	s__uncultured_Roseobacter_sp._g__norank_f__norank	OTU362
d__Bacteria	k__norank_ d__Bacteria	p__Bdellovibrionota	c__Bdellovibrionia	o__Bdellovibrionales	f__Bdellovibrionaceae	g__Bdellovibrio	s__uncultured_bacterium_g__Bdellovibrio	OTU1755
d__Bacteria	k__norank_ d__Bacteria	p__Acidobacteriota	c__Blastocatellia	o__11-24	f__norank_o__11-24	g__norank_f__norank_o__11-24	s__uncultured_bacterium_g__norank_f__norank_o__11-24	OTU1272
d__Bacteria	k__norank_ d__Bacteria	p__Patescibacteria	c__Saccharimonadia	o__Saccharimonadales	f__norank_o__Saccharimonadales	g__norank_f__norank_o__Saccharimo nadales	s__uncultured_bacterium_g__norank_f__norank_o__Saccharimonadales	OTU1206
d__Bacteria	k__norank_ d__Bacteria	p__Planctomycetota	c__Planctomycetes	o__Gemmatales	f__Gemmataceae	g__norank_f__Gemmataceae	s__uncultured_planctomycete_g__norank_f__Gemmataceae	OTU1000
d__Bacteria	k__norank_ d__Bacteria	p__Myxococcota	c__Polyangia	o__mle1-27	f__norank_o__mle1-27	g__norank_f__norank_o__mle1-27	s__uncultured_Polyangiaceae_bacterium_g__norank_f__norank	OTU801
d__Bacteria	k__norank_ d__Bacteria	p__Actinobacteriota	c__Acidimicrobiia	o__IMCC26256	f__norank_o__IMCC26256	g__norank_f__norank_o__IMCC26256	s__actinobacterium_YJF2-33	OTU1783
d__Bacteria	k__norank_ d__Bacteria	p__Acidobacteriota	c__Vicinamibacteria	o__Vicinamibacterales	f__norank_o__Vicinamibacterales	g__norank_f__norank_o__Vicinamiba cterales	s__unclassified_g__norank_f__norank_o__Vicinamibacterales	OTU1267
d__Bacteria	k__norank_ d__Bacteria	p__Proteobacteria	c__Alphaproteobacteria	o__Acetobacterales	f__Acetobacteraceae	g__Rubritepida	s__uncultured_bacterium_g__Rubritepida	OTU2953
d__Bacteria	k__norank_ d__Bacteria	p__Patescibacteria	c__Parcubacteria	o__norank_c__Parcubacteria	f__norank_o__norank_c__Parcubacteria	g__norank_f__norank_o__norank_c__ Parcubacteria	s__uncultured_bacterium_g__norank_f__norank_o__norank_c__Parcubacteria	OTU2575
d__Bacteria	k__norank_ d__Bacteria	p__Planctomycetota	c__Planctomycetes	o__Isosphaerales	f__Isosphaeraceae	g__Tundrisphaera	s__unclassified_g__Tundrisphaera	OTU48
d__Bacteria	k__norank_ d__Bacteria	p__Proteobacteria	c__Alphaproteobacteria	o__Paracaedibacterales	f__Paracaedibacteraceae	g__Candidatus_Captivus	s__uncultured_bacterium_g__Candidatus_Captivus	OTU297
d__Bacteria	k__norank_ d__Bacteria	p__Chloroflexi	c__unclassified_p__Chlor oflexi	o__unclassified_p__Chlorofl exi	f__unclassified_p__Chloroflexi	g__unclassified_p__Chloroflexi	s__unclassified_p__Chloroflexi	OTU1486

d__Bacteria	k__norank_d__Bacteria	p__Deinococcota	c__Deinococci	o__Deinococcales	f__Deinococcaceae	g__Deinococcus	s__unclassified_g__Deinococcus	OTU107
d__Bacteria	k__norank_d__Bacteria	p__Planctomycetota	c__Planctomycetes	o__Isosphaerales	f__Isosphaeraceae	g__Candidatus_Nostocoida	s__uncultured_Singulisphaera_sp._g__Candidatus_Nostocoida	OTU7
d__Bacteria	k__norank_d__Bacteria	p__Fusobacteriota	c__Fusobacteriia	o__Fusobacteriales	f__Leptotrichiaceae	g__Hypnocyclicus	s__unclassified_g__Hypnocyclicus	OTU60
d__Bacteria	k__norank_d__Bacteria	p__Actinobacteriota	c__Actinobacteria	o__Propionibacteriales	f__Nocardioidaceae	g__Nocardioides	s__unclassified_g__Nocardioides	OTU2343
d__Bacteria	k__norank_d__Bacteria	p__Actinobacteriota	c__Acidimicrobiia	o__IMCC26256	f__norank_o__IMCC26256	g__norank_f__norank_o__IMCC26256	s__bacterium_enrichment_culture_clone_auto73_4W	OTU1777
d__Bacteria	k__norank_d__Bacteria	p__Bacteroidota	c__Bacteroidia	o__Sphingobacteriales	f__env.OPS_17	g__norank_f__env.OPS_17	s__uncultured_Bacteroidetes_bacterium_g__norank_f__env.OPS_17	OTU2332
d__Bacteria	k__norank_d__Bacteria	p__Gemmatimonadota	c__Gemmatimonadetes	o__Gemmatimonadales	f__Gemmatimonadaceae	g__Gemmatimonas	s__unclassified_g__Gemmatimonas	OTU974
d__Bacteria	k__norank_d__Bacteria	p__Gemmatimonadota	c__Gemmatimonadetes	o__Gemmatimonadales	f__Gemmatimonadaceae	g__Gemmatimonas	s__unclassified_g__Gemmatimonas	OTU973
d__Bacteria	k__norank_d__Bacteria	p__Chloroflexi	c__Ktedonobacteria	o__C0119	f__norank_o__C0119	g__norank_f__norank_o__C0119	s__uncultured_bacterium_g__norank_f__norank_o__C0119	OTU1493
d__Bacteria	k__norank_d__Bacteria	p__Acidobacteriota	c__Blastocatellia	o__Blastocatellales	f__Blastocatellaceae	g__Stenotrophobacter	s__uncultured_bacterium_g__Stenotrophobacter	OTU2434
d__Bacteria	k__norank_d__Bacteria	p__WPS-2	c__norank_p__WPS-2	o__norank_c__norank_p__WPS-2	f__norank_o__norank_c__norank_p__WPS-2	g__norank_f__norank_o__norank_c__norank_p__WPS-2	s__uncultured_bacterium_g__norank_f__norank_o__norank_c__norank_p__WPS-2	OTU813
d__Bacteria	k__norank_d__Bacteria	p__Gemmatimonadota	c__Gemmatimonadetes	o__Gemmatimonadales	f__Gemmatimonadaceae	g__Gemmatimonas	s__unclassified_g__Gemmatimonas	OTU979
d__Bacteria	k__norank_d__Bacteria	p__Armatimonadota	c__norank_p__Armatimonadota	o__norank_c__norank_p__Armatimonadota	f__norank_o__norank_c__norank_p__Armatimonadota	g__norank_f__norank_o__norank_c__norank_p__Armatimonadota	s__metagenome_g__norank_f__norank_o__norank_c__norank_p__Armatimonadota	OTU267
d__Bacteria	k__norank_d__Bacteria	p__unclassified_k__norank_d__Bacteria	c__unclassified_k__norank_d__Bacteria	o__unclassified_k__norank_d__Bacteria	f__unclassified_k__norank_d__Bacteria	g__unclassified_k__norank_d__Bacteria	s__unclassified_k__norank_d__Bacteria	OTU2076
d__Bacteria	k__norank_d__Bacteria	p__Gemmatimonadota	c__Gemmatimonadetes	o__Gemmatimonadales	f__Gemmatimonadaceae	g__unclassified_f__Gemmatimonadaceae	s__unclassified_f__Gemmatimonadaceae	OTU969
d__Bacteria	k__norank_d__Bacteria	p__Proteobacteria	c__Alphaproteobacteria	o__Holosporales	f__Holosporaceae	g__norank_f__Holosporaceae	s__uncultured_bacterium_g__norank_f__Holosporaceae	OTU1662

d__Bacteria	k__norank_ d__Bacteria	p__Armatimonadota	c__Armatimonadia	o__Armatimonadales	f__norank_o__Armatimonadales	g__norank_f__norank_o__Armatimona dales	s__unclassified_g__norank_f__norank_o__Armatimonadales	OTU375
d__Bacteria	k__norank_ d__Bacteria	p__Chloroflexi	c__Chloroflexia	o__Thermomicrobiales	f__AKYG1722	g__norank_f__AKYG1722	s__uncultured_bacterium_g__norank_f__AKYG1722	OTU2267
d__Bacteria	k__norank_ d__Bacteria	p__Acidobacteriota	c__Blastocatellia	o__Blastocatellales	f__Blastocatellaceae	g__Aridibacter	s__uncultured_bacterium_g__Aridibacter	OTU2335
d__Bacteria	k__norank_ d__Bacteria	p__Bacteroidota	c__Bacteroidia	o__Cytophagales	f__Hymenobacteraceae	g__Hymenobacter	s__uncultured_Bacteroidetes_bacterium_g__Hymenobacter	OTU148
d__Bacteria	k__norank_ d__Bacteria	p__Chloroflexi	c__Anaerolineae	o__SBR1031	f__norank_o__SBR1031	g__norank_f__norank_o__SBR1031	s__uncultured_soil_bacterium_g__norank_f__norank_o__SBR1031	OTU2207
d__Bacteria	k__norank_ d__Bacteria	p__Patescibacteria	c__Saccharimonadia	o__Saccharimonadales	f__norank_o__Saccharimonadales	g__norank_f__norank_o__Saccharimo nadales	s__uncultured_bacterium_g__norank_f__norank_o__Saccharimonadales	OTU2331
d__Bacteria	k__norank_ d__Bacteria	p__Bacteroidota	c__Bacteroidia	o__Cytophagales	f__Microscillaceae	g__norank_f__Microscillaceae	s__uncultured_bacterium_g__norank_f__Microscillaceae	OTU2062
d__Bacteria	k__norank_ d__Bacteria	p__Acidobacteriota	c__Thermoanaerobaculia	o__Thermoanaerobaculales	f__Thermoanaerobaculaceae	g__Subgroup_10	s__unclassified_g__Subgroup_10	OTU2122
d__Bacteria	k__norank_ d__Bacteria	p__Proteobacteria	c__Alphaproteobacteria	o__Sphingomonadales	f__Sphingomonadaceae	g__Sphingomonas	s__Sphingomonas_sp._LRR22	OTU2269
d__Bacteria	k__norank_ d__Bacteria	p__Proteobacteria	c__Alphaproteobacteria	o__Micropepsales	f__Micropepsaceae	g__norank_f__Micropepsaceae	s__metagenome_g__norank_f__Micropepsaceae	OTU1984
d__Bacteria	k__norank_ d__Bacteria	p__Acidobacteriota	c__Thermoanaerobaculia	o__Thermoanaerobaculales	f__Thermoanaerobaculaceae	g__Subgroup_10	s__unclassified_g__Subgroup_10	OTU1618
d__Bacteria	k__norank_ d__Bacteria	p__Myxococcota	c__Polyangia	o__Haliangiales	f__Haliangiaceae	g__Haliangium	s__uncultured_bacterium_g__Haliangium	OTU830
d__Bacteria	k__norank_ d__Bacteria	p__Cyanobacteria	c__Cyanobacteriia	o__Chloroplast	f__norank_o__Chloroplast	g__norank_f__norank_o__Chloroplast	s__unclassified_g__norank_f__norank_o__Chloroplast	OTU2932
d__Bacteria	k__norank_ d__Bacteria	p__Proteobacteria	c__Gammaproteobacteria	o__Burkholderiales	f__Oxalobacteraceae	g__Massilia	s__Massilia_violaceinigra	OTU100
d__Bacteria	k__norank_ d__Bacteria	p__WPS-2	c__norank_p__WPS-2	o__norank_c__norank_p__ WPS-2	f__norank_o__norank_c__norank_p__W PS-2	g__norank_f__norank_o__norank_c__ norank_p__WPS-2	s__uncultured_bacterium_g__norank_f__norank_o__norank_c__norank_p__WPS-2	OTU1944
d__Bacteria	k__norank_ d__Bacteria	p__Proteobacteria	c__Gammaproteobacteria	o__Xanthomonadales	f__Xanthomonadaceae	g__Lysobacter	s__Lysobacter_soli	OTU188

d__Bacteria	k__norank_ d__Bacteria	p__Bdellovibrionota	c__Oligoflexia	o__0319-6G20	f__norank_o__0319-6G20	g__norank_f__norank_o__0319-6G20	s__bacterium_enrichment_culture_clone_B1262011	OTU1210
d__Bacteria	k__norank_ d__Bacteria	p__Proteobacteria	c__Alphaproteobacteria	o__Rhizobiales	f__Beijerinckiaceae	g__Psychroglaciecola	s__uncultured_bacterium_g__Psychroglaciecola	OTU1879
d__Bacteria	k__norank_ d__Bacteria	p__Chloroflexi	c__Chloroflexia	o__Thermomicrobiales	f__JG30-KF-CM45	g__norank_f__JG30-KF-CM45	s__uncultured_bacterium_g__norank_f__JG30-KF-CM45	OTU389
d__Bacteria	k__norank_ d__Bacteria	p__Acidobacteriota	c__Acidobacteriae	o__Solibacterales	f__Solibacteraceae	g__Candidatus_Solibacter	s__metagenome_g__Candidatus_Solibacter	OTU1947
d__Bacteria	k__norank_ d__Bacteria	p__Acidobacteriota	c__Vicinamibacteria	o__Vicinamibacterales	f__Vicinamibacteraceae	g__norank_f__Vicinamibacteraceae	s__unclassified_g__norank_f__Vicinamibacteraceae	OTU273
d__Bacteria	k__norank_ d__Bacteria	p__Actinobacteriota	c__Actinobacteria	o__Kineosporiales	f__Kineosporiaceae	g__unclassified_f__Kineosporiaceae	s__unclassified_f__Kineosporiaceae	OTU2673
d__Bacteria	k__norank_ d__Bacteria	p__Actinobacteriota	c__Actinobacteria	o__Corynebacteriales	f__Mycobacteriaceae	g__Mycobacterium	s__unclassified_g__Mycobacterium	OTU1404
d__Bacteria	k__norank_ d__Bacteria	p__Actinobacteriota	c__Actinobacteria	o__Streptosporangiales	f__Streptosporangiaceae	g__Streptosporangium	s__unclassified_g__Streptosporangium	OTU1887
d__Bacteria	k__norank_ d__Bacteria	p__Gemmatimonadota	c__Longimicrobia	o__Longimicrobiales	f__Longimicrobiaceae	g__norank_f__Longimicrobiaceae	s__uncultured_bacterium_g__norank_f__Longimicrobiaceae	OTU2984
d__Bacteria	k__norank_ d__Bacteria	p__Cyanobacteria	c__Cyanobacteriia	o__Cyanobacteriales	f__Coleofasciculaceae	g__Wilmottia_Ant-Ph58	s__unclassified_g__Wilmottia_Ant-Ph58	OTU2931
d__Bacteria	k__norank_ d__Bacteria	p__Proteobacteria	c__Alphaproteobacteria	o__Rickettsiales	f__Mitochondria	g__norank_f__Mitochondria	s__unclassified_g__norank_f__Mitochondria	OTU1892
d__Bacteria	k__norank_ d__Bacteria	p__Patescibacteria	c__Saccharimonadia	o__Saccharimonadales	f__unclassified_o__Saccharimonadales	g__unclassified_o__Saccharimonadale s	s__unclassified_o__Saccharimonadales	OTU282
d__Bacteria	k__norank_ d__Bacteria	p__unclassified_k__no rank_d__Bacteria	c__unclassified_k__noran k_d__Bacteria	o__unclassified_k__norank_ d__Bacteria	f__unclassified_k__norank_d__Bacteria	g__unclassified_k__norank_d__Bacteri a	s__unclassified_k__norank_d__Bacteria	OTU717
d__Bacteria	k__norank_ d__Bacteria	p__Myxococcota	c__Polyangia	o__Polyangiales	f__Polyangiaceae	g__Minicystis	s__uncultured_bacterium_g__Minicystis	OTU741
d__Bacteria	k__norank_ d__Bacteria	p__Deinococcota	c__Deinococci	o__Deinococcales	f__Deinococcaceae	g__Deinococcus	s__unclassified_g__Deinococcus	OTU2555
d__Bacteria	k__norank_ d__Bacteria	p__Proteobacteria	c__Gammaproteobacteria	o__Burkholderiales	f__Oxalobacteraceae	g__unclassified_f__Oxalobacteraceae	s__unclassified_f__Oxalobacteraceae	OTU1568

d__Bacteria	k__norank_ d__Bacteria	p__Proteobacteria	c__Gammaproteobacteria	o__Burkholderiales	f__Nitrosomonadaceae	g__mle1-7	s__uncultured_bacterium_g__mle1-7	OTU1394
d__Bacteria	k__norank_ d__Bacteria	p__Proteobacteria	c__Gammaproteobacteria	o__Burkholderiales	f__Nitrosomonadaceae	g__mle1-7	s__uncultured_bacterium_g__mle1-7	OTU1391
d__Bacteria	k__norank_ d__Bacteria	p__Chloroflexi	c__Chloroflexia	o__Elev-1554	f__norank_o__Elev-1554	g__norank_f__norank_o__Elev-1554	s__uncultured_bacterium_g__norank_f__norank_o__Elev-1554	OTU1883
d__Bacteria	k__norank_ d__Bacteria	p__Chloroflexi	c__AD3	o__norank_c__AD3	f__norank_o__norank_c__AD3	g__norank_f__norank_o__norank_c__ AD3	s__unclassified_g__norank_f__norank_o__norank_c__AD3	OTU769
d__Bacteria	k__norank_ d__Bacteria	p__Proteobacteria	c__Gammaproteobacteria	o__Diplorickettsiales	f__Diplorickettsiaceae	g__norank_f__Diplorickettsiaceae	s__unclassified_g__norank_f__Diplorickettsiaceae	OTU1158
d__Bacteria	k__norank_ d__Bacteria	p__Actinobacteriota	c__Acidimicrobiia	o__Microtrichales	f__Iamiaceae	g__Iamia	s__metagenome_g__Iamia	OTU592
d__Bacteria	k__norank_ d__Bacteria	p__Bacteroidota	c__Bacteroidia	o__Chitinophagales	f__Chitinophagaceae	g__Ferruginibacter	s__uncultured_bacterium_g__Ferruginibacter	OTU1307
d__Bacteria	k__norank_ d__Bacteria	p__Bdellovibrionota	c__Bdellovibrionia	o__Bdellovibrionales	f__Bdellovibrionaceae	g__OM27_clade	s__unclassified_g__OM27_clade	OTU639
d__Bacteria	k__norank_ d__Bacteria	p__Proteobacteria	c__Gammaproteobacteria	o__Burkholderiales	f__SC-I-84	g__norank_f__SC-I-84	s__metagenome_g__norank_f__SC-I-84	OTU963
d__Bacteria	k__norank_ d__Bacteria	p__Bdellovibrionota	c__Bdellovibrionia	o__Bdellovibrionales	f__Bdellovibrionaceae	g__Bdellovibrio	s__metagenome_g__Bdellovibrio	OTU67
d__Bacteria	k__norank_ d__Bacteria	p__Proteobacteria	c__Gammaproteobacteria	o__Legionellales	f__Legionellaceae	g__Legionella	s__unclassified_g__Legionella	OTU2242
d__Bacteria	k__norank_ d__Bacteria	p__Gemmatimonadota	c__Gemmatimonadetes	o__Gemmatimonadales	f__Gemmatimonadaceae	g__norank_f__Gemmatimonadaceae	s__Gemmatimonadetes_bacterium_SCN_70-22	OTU329
d__Bacteria	k__norank_ d__Bacteria	p__Acidobacteriota	c__Acidobacteriae	o__norank_c__Acidobacteri ae	f__norank_o__norank_c__Acidobacteriae	g__Paludibaculum	s__metagenome_g__Paludibaculum	OTU670
d__Bacteria	k__norank_ d__Bacteria	p__Planctomycetota	c__Planctomycetes	o__Gemmatales	f__Gemmataceae	g__norank_f__Gemmataceae	s__uncultured_bacterium_g__norank_f__Gemmataceae	OTU860
d__Bacteria	k__norank_ d__Bacteria	p__Actinobacteriota	c__Acidimicrobiia	o__unclassified_c__Acidimi crobiia	f__unclassified_c__Acidimicrobiia	g__unclassified_c__Acidimicrobiia	s__unclassified_c__Acidimicrobiia	OTU1110
d__Bacteria	k__norank_ d__Bacteria	p__Proteobacteria	c__Alphaproteobacteria	o__Rhizobiales	f__Beijerinckiaceae	g__1174-901-12	s__unclassified_g__1174-901-12	OTU1069

d__Bacteria	k__norank_d__Bacteria	p__Verrucomicrobiota	c__Verrucomicrobiae	o__Chthoniobacterales	f__Chthoniobacteraceae	g__Candidatus_Udaeobacter	s__unclassified_g__Candidatus_Udaeobacter	OTU1926
d__Bacteria	k__norank_d__Bacteria	p__Chloroflexi	c__Ktedonobacteria	o__Ktedonobacterales	f__Ktedonobacteraceae	g__1921-2	s__unclassified_g__1921-2	OTU1917
d__Bacteria	k__norank_d__Bacteria	p__Myxococcota	c__Myxococcia	o__Myxococcales	f__Myxococcaceae	g__norank_f__Myxococcaceae	s__uncultured_bacterium_g__norank_f__Myxococcaceae	OTU650
d__Bacteria	k__norank_d__Bacteria	p__Planctomycetota	c__Planctomycetes	o__Gemmatales	f__Gemmataceae	g__norank_f__Gemmataceae	s__unclassified_g__norank_f__Gemmataceae	OTU1333
d__Bacteria	k__norank_d__Bacteria	p__Planctomycetota	c__Planctomycetes	o__Isosphaerales	f__Isosphaeraceae	g__Tundrisphaera	s__uncultured_bacterium_g__Tundrisphaera	OTU1131
d__Bacteria	k__norank_d__Bacteria	p__Proteobacteria	c__Gammaproteobacteria	o__Pseudomonadales	f__Moraxellaceae	g__Acinetobacter	s__Acinetobacter_sp._TTH0-4	OTU238
d__Bacteria	k__norank_d__Bacteria	p__Acidobacteriota	c__Vicinamibacteria	o__Vicinamibacterales	f__norank_o__Vicinamibacterales	g__norank_f__norank_o__Vicinamibacterales	s__unclassified_g__norank_f__norank_o__Vicinamibacterales	OTU1438
d__Bacteria	k__norank_d__Bacteria	p__Bacteroidota	c__Bacteroidia	o__Chitinophagales	f__Saprospiraceae	g__norank_f__Saprospiraceae	s__unclassified_g__norank_f__Saprospiraceae	OTU1321
d__Bacteria	k__norank_d__Bacteria	p__Proteobacteria	c__Alphaproteobacteria	o__Rhizobiales	f__Rhizobiales_Incertae_Sedis	g__norank_f__Rhizobiales_Incertae_Sedis	s__unclassified_g__norank_f__Rhizobiales_Incertae_Sedis	OTU497
d__Bacteria	k__norank_d__Bacteria	p__Chloroflexi	c__Chloroflexia	o__Thermomicrobiales	f__JG30-KF-CM45	g__norank_f__JG30-KF-CM45	s__metagenome_g__norank_f__JG30-KF-CM45	OTU1475
d__Bacteria	k__norank_d__Bacteria	p__Planctomycetota	c__Planctomycetes	o__Isosphaerales	f__Isosphaeraceae	g__unclassified_f__Isosphaeraceae	s__unclassified_f__Isosphaeraceae	OTU1036
d__Bacteria	k__norank_d__Bacteria	p__Proteobacteria	c__Gammaproteobacteria	o__Burkholderiales	f__Comamonadaceae	g__unclassified_f__Comamonadaceae	s__unclassified_f__Comamonadaceae	OTU1029
d__Bacteria	k__norank_d__Bacteria	p__Chloroflexi	c__Ktedonobacteria	o__C0119	f__norank_o__C0119	g__norank_f__norank_o__C0119	s__uncultured_bacterium_g__norank_f__norank_o__C0119	OTU1108
d__Bacteria	k__norank_d__Bacteria	p__Bacteroidota	c__Bacteroidia	o__Cytophagales	f__Hymenobacteraceae	g__Hymenobacter	s__unclassified_g__Hymenobacter	OTU104
d__Bacteria	k__norank_d__Bacteria	p__Gemmatimonadota	c__Longimicrobia	o__Longimicrobiales	f__Longimicrobiaceae	g__YC-ZSS-LKJ147	s__uncultured_bacterium_g__YC-ZSS-LKJ147	OTU350
d__Bacteria	k__norank_d__Bacteria	p__Acidobacteriota	c__Vicinamibacteria	o__Vicinamibacterales	f__norank_o__Vicinamibacterales	g__norank_f__norank_o__Vicinamibacterales	s__uncultured_Acidobacteria_bacterium_g__norank_f__norank_o__Vicinamibacterales	OTU1415

d__Bacteria	k__norank_ d__Bacteria	p__Cyanobacteria	c__Cyanobacteriia	o__Chloroplast	f__norank_o__Chloroplast	g__norank_f__norank_o__Chloroplast	s__Prototheca_wickerhamii_g__norank_f__norank	OTU363
d__Bacteria	k__norank_ d__Bacteria	p__Actinobacteriota	c__Actinobacteria	o__Pseudonocardiales	f__Pseudonocardiaceae	g__Amycolatopsis	s__unclassified_g__Amycolatopsis	OTU994
d__Bacteria	k__norank_ d__Bacteria	p__Chloroflexi	c__Ktedonobacteria	o__C0119	f__norank_o__C0119	g__norank_f__norank_o__C0119	s__uncultured_bacterium_g__norank_f__norank_o__C0119	OTU1107
d__Bacteria	k__norank_ d__Bacteria	p__Gemmatimonadota	c__S0134_terrestrial_grou p	o__norank_c__S0134_terres trial_group	f__norank_o__norank_c__S0134_terrestr ial_group	g__norank_f__norank_o__norank_c__ S0134_terrestrial_group	s__metagenome_g__norank_f__norank_o__norank_c__S0134_terrestrial_group	OTU2586
d__Bacteria	k__norank_ d__Bacteria	p__Proteobacteria	c__Gammaproteobacteria	o__Burkholderiales	f__Oxalobacteraceae	g__Massilia	s__uncultured_bacterium_g__Massilia	OTU378
d__Bacteria	k__norank_ d__Bacteria	p__Patescibacteria	c__Parcubacteria	o__norank_c__Parcubacteria	f__norank_o__norank_c__Parcubacteria	g__norank_f__norank_o__norank_c__ Parcubacteria	s__uncultured_bacterium_g__norank_f__norank_o__norank_c__Parcubacteria	OTU326
d__Bacteria	k__norank_ d__Bacteria	p__Proteobacteria	c__Gammaproteobacteria	o__Oceanospirillales	f__Pseudohongiellaceae	g__BIyi10	s__unclassified_g__BIyi10	OTU2100
d__Bacteria	k__norank_ d__Bacteria	p__Chloroflexi	c__JG30-KF-CM66	o__norank_c__JG30-KF-C M66	f__norank_o__norank_c__JG30-KF-CM 66	g__norank_f__norank_o__norank_c__ JG30-KF-CM66	s__uncultured_bacterium_g__norank_f__norank_o__norank_c__JG30-KF-CM66	OTU706
d__Bacteria	k__norank_ d__Bacteria	p__Proteobacteria	c__Gammaproteobacteria	o__Diplorickettsiales	f__Diplorickettsiaceae	g__norank_f__Diplorickettsiaceae	s__unclassified_g__norank_f__Diplorickettsiaceae	OTU33
d__Bacteria	k__norank_ d__Bacteria	p__Bacteroidota	c__Bacteroidia	o__Chitinophagales	f__Saprospiraceae	g__norank_f__Saprospiraceae	s__uncultured_bacterium_g__norank_f__Saprospiraceae	OTU2625
d__Bacteria	k__norank_ d__Bacteria	p__Planctomycetota	c__Planctomycetes	o__Gemmatales	f__Gemmataceae	g__norank_f__Gemmataceae	s__unclassified_g__norank_f__Gemmataceae	OTU946
d__Bacteria	k__norank_ d__Bacteria	p__Proteobacteria	c__Gammaproteobacteria	o__WD260	f__norank_o__WD260	g__norank_f__norank_o__WD260	s__uncultured_bacterium_g__norank_f__norank_o__WD260	OTU2394
d__Bacteria	k__norank_ d__Bacteria	p__Bacteroidota	c__Bacteroidia	o__Cytophagales	f__Hymenobacteraceae	g__Hymenobacter	s__uncultured_bacterium_g__Hymenobacter	OTU1728
d__Bacteria	k__norank_ d__Bacteria	p__Chloroflexi	c__JG30-KF-CM66	o__norank_c__JG30-KF-C M66	f__norank_o__norank_c__JG30-KF-CM 66	g__norank_f__norank_o__norank_c__ JG30-KF-CM66	s__unclassified_g__norank_f__norank_o__norank_c__JG30-KF-CM66	OTU1017
d__Bacteria	k__norank_ d__Bacteria	p__Bacteroidota	c__Kapabacteria	o__Kapabacteriales	f__norank_o__Kapabacteriales	g__norank_f__norank_o__Kapabacteri ales	s__uncultured_bacterium_g__norank_f__norank_o__Kapabacteriales	OTU2954
d__Bacteria	k__norank_ d__Bacteria	p__Gemmatimonadota	c__Gemmatimonadetes	o__Gemmatimonadales	f__Gemmatimonadaceae	g__unclassified_f__Gemmatimonadace ae	s__unclassified_f__Gemmatimonadaceae	OTU550

d__Bacteria	k__norank_d__Bacteria	p__Myxococcota	c__Polyangia	o__Haliangiales	f__Haliangiaceae	g__Haliangium	s__uncultured_bacterium_g__Haliangium	OTU620
d__Bacteria	k__norank_d__Bacteria	p__Acidobacteriota	c__Acidobacteriae	o__Solibacterales	f__Solibacteraceae	g__Candidatus_Solibacter	s__uncultured_bacterium_g__Candidatus_Solibacter	OTU1953
d__Bacteria	k__norank_d__Bacteria	p__Proteobacteria	c__Gammaproteobacteria	o__Burkholderiales	f__Oxalobacteraceae	g__Aquaspirillum_arcticum_group	s__uncultured_bacterium_g__Aquaspirillum_arcticum_group	OTU2027
d__Bacteria	k__norank_d__Bacteria	p__Chloroflexi	c__Ktedonobacteria	o__Ktedonobacterales	f__Ktedonobacteraceae	g__unclassified_f__Ktedonobacteraceae	s__unclassified_f__Ktedonobacteraceae	OTU1724
d__Bacteria	k__norank_d__Bacteria	p__Proteobacteria	c__Alphaproteobacteria	o__Rhizobiales	f__Rhizobiaceae	g__Mesorhizobium	s__unclassified_g__Mesorhizobium	OTU711
d__Bacteria	k__norank_d__Bacteria	p__Chloroflexi	c__Ktedonobacteria	o__C0119	f__norank_o__C0119	g__norank_f__norank_o__C0119	s__uncultured_bacterium_g__norank_f__norank_o__C0119	OTU496
d__Bacteria	k__norank_d__Bacteria	p__Patescibacteria	c__Saccharimonadia	o__Saccharimonadales	f__norank_o__Saccharimonadales	g__norank_f__norank_o__Saccharimonadales	s__metagenome_g__norank_f__norank_o__Saccharimonadales	OTU2518
d__Bacteria	k__norank_d__Bacteria	p__Armatimonadota	c__norank_p__Armatimonadota	o__norank_c__norank_p__Armatimonadota	f__norank_o__norank_c__norank_p__Armatimonadota	g__norank_f__norank_o__norank_c__norank_p__Armatimonadota	s__metagenome_g__norank_f__norank_o__norank_c__norank_p__Armatimonadota	OTU2759
d__Bacteria	k__norank_d__Bacteria	p__unclassified_k__norank_d__Bacteria	c__unclassified_k__norank_d__Bacteria	o__unclassified_k__norank_d__Bacteria	f__unclassified_k__norank_d__Bacteria	g__unclassified_k__norank_d__Bacteria	s__unclassified_k__norank_d__Bacteria	OTU380
d__Bacteria	k__norank_d__Bacteria	p__Verrucomicrobiota	c__Verrucomicrobiae	o__Verrucomicrobiales	f__Verrucomicrobiaceae	g__norank_f__Verrucomicrobiaceae	s__uncultured_bacterium_g__norank_f__Verrucomicrobiaceae	OTU2099
d__Bacteria	k__norank_d__Bacteria	p__Actinobacteriota	c__Thermoleophilia	o__Gaiellales	f__norank_o__Gaiellales	g__norank_f__norank_o__Gaiellales	s__unclassified_g__norank_f__norank_o__Gaiellales	OTU720
d__Bacteria	k__norank_d__Bacteria	p__Actinobacteriota	c__Thermoleophilia	o__Solirubrobacterales	f__Solirubrobacteraceae	g__Conexibacter	s__uncultured_bacterium_g__Conexibacter	OTU2393
d__Bacteria	k__norank_d__Bacteria	p__Actinobacteriota	c__Acidimicrobiia	o__Actinomarinales	f__norank_o__Actinomarinales	g__norank_f__norank_o__Actinomarinales	s__unclassified_g__norank_f__norank_o__Actinomarinales	OTU608
d__Bacteria	k__norank_d__Bacteria	p__Gemmatimonadota	c__Gemmatimonadetes	o__Gemmatimonadales	f__Gemmatimonadaceae	g__Gemmatimonas	s__unclassified_g__Gemmatimonas	OTU625
d__Bacteria	k__norank_d__Bacteria	p__Patescibacteria	c__Saccharimonadia	o__Saccharimonadales	f__norank_o__Saccharimonadales	g__norank_f__norank_o__Saccharimonadales	s__uncultured_bacterium_g__norank_f__norank_o__Saccharimonadales	OTU2782
d__Bacteria	k__norank_d__Bacteria	p__Actinobacteriota	c__Thermoleophilia	o__Solirubrobacterales	f__Solirubrobacteraceae	g__Conexibacter	s__unclassified_g__Conexibacter	OTU2400

d__Bacteria	k__norank_ d__Bacteria	p__Myxococcota	c__Myxococcia	o__Myxococcales	f__Anaeromyxobacteraceae	g__Anaeromyxobacter	s__uncultured_bacterium_g__Anaeromyxobacter	OTU567
d__Bacteria	k__norank_ d__Bacteria	p__Cyanobacteria	c__Cyanobacteriia	o__Oxyphotobacteria_Incertae_Sedis	f__unclassified_o__Oxyphotobacteria_Incertae_Sedis	g__Leptolyngbya_ANT.L52.2	s__uncultured_bacterium_g__Leptolyngbya_ANT.L52.2	OTU2852
d__Bacteria	k__norank_ d__Bacteria	p__Proteobacteria	c__Gammaproteobacteria	o__Legionellales	f__Legionellaceae	g__Legionella	s__uncultured_bacterium_g__Legionella	OTU641
d__Bacteria	k__norank_ d__Bacteria	p__Bacteroidota	c__Bacteroidia	o__Chitinophagales	f__Chitinophagaceae	g__Flavisolibacter	s__unclassified_g__Flavisolibacter	OTU1610
d__Bacteria	k__norank_ d__Bacteria	p__Actinobacteriota	c__Thermoleophilia	o__Gaiellales	f__norank_o__Gaiellales	g__norank_f__norank_o__Gaiellales	s__unclassified_g__norank_f__norank_o__Gaiellales	OTU1828
d__Bacteria	k__norank_ d__Bacteria	p__Deinococcota	c__Deinococci	o__Deinococcales	f__Deinococcaceae	g__Deinococcus	s__uncultured_bacterium_g__Deinococcus	OTU2693
d__Bacteria	k__norank_ d__Bacteria	p__Bdellovibrionota	c__Oligoflexia	o__Oligoflexales	f__norank_o__Oligoflexales	g__norank_f__norank_o__Oligoflexales	s__unclassified_g__norank_f__norank_o__Oligoflexales	OTU2188
d__Bacteria	k__norank_ d__Bacteria	p__Myxococcota	c__Polyangia	o__Polyangiales	f__Sandaracinaceae	g__norank_f__Sandaracinaceae	s__unclassified_g__norank_f__Sandaracinaceae	OTU2024
d__Bacteria	k__norank_ d__Bacteria	p__Proteobacteria	c__Gammaproteobacteria	o__Burkholderiales	f__Comamonadaceae	g__unclassified_f__Comamonadaceae	s__unclassified_f__Comamonadaceae	OTU503
d__Bacteria	k__norank_ d__Bacteria	p__Bacteroidota	c__Bacteroidia	o__Flavobacteriales	f__Flavobacteriaceae	g__Flavobacterium	s__unclassified_g__Flavobacterium	OTU2819
d__Bacteria	k__norank_ d__Bacteria	p__Acidobacteriota	c__Holophagae	o__Subgroup_7	f__norank_o__Subgroup_7	g__norank_f__norank_o__Subgroup_7	s__unclassified_g__norank_f__norank_o__Subgroup_7	OTU432
d__Bacteria	k__norank_ d__Bacteria	p__Myxococcota	c__Polyangia	o__Haliangiales	f__Haliangiaceae	g__Haliangium	s__metagenome_g__Haliangium	OTU1552
d__Bacteria	k__norank_ d__Bacteria	p__Acidobacteriota	c__Vicinamibacteria	o__Vicinamibacterales	f__norank_o__Vicinamibacterales	g__norank_f__norank_o__Vicinamibacterales	s__uncultured_Acidobacteria_bacterium_g__norank_f__norank_o__Vicinamibacterales	OTU1385
d__Bacteria	k__norank_ d__Bacteria	p__Actinobacteriota	c__Thermoleophilia	o__Solirubrobacterales	f__Solirubrobacteraceae	g__unclassified_f__Solirubrobacteraceae	s__unclassified_f__Solirubrobacteraceae	OTU2395
d__Bacteria	k__norank_ d__Bacteria	p__Cyanobacteria	c__unclassified_p__Cyanobacteria	o__unclassified_p__Cyanobacteria	f__unclassified_p__Cyanobacteria	g__unclassified_p__Cyanobacteria	s__unclassified_p__Cyanobacteria	OTU1235
d__Bacteria	k__norank_ d__Bacteria	p__Chloroflexi	c__Anaerolineae	o__SBR1031	f__unclassified_o__SBR1031	g__unclassified_o__SBR1031	s__unclassified_o__SBR1031	OTU967

d__Bacteria	k__norank_ d__Bacteria	p__Actinobacteriota	c__Acidimicrobiia	o__Microtrichales	f__Ilumatobacteraceae	g__CL500-29_marine_group	s__metagenome_g__CL500-29_marine_group	OTU2438
d__Bacteria	k__norank_ d__Bacteria	p__Proteobacteria	c__Gammaproteobacteria	o__Burkholderiales	f__unclassified_o__Burkholderiales	g__unclassified_o__Burkholderiales	s__unclassified_o__Burkholderiales	OTU2779
d__Bacteria	k__norank_ d__Bacteria	p__Firmicutes	c__Bacilli	o__Paenibacillales	f__Paenibacillaceae	g__Paenibacillus	s__unclassified_g__Paenibacillus	OTU2411
d__Bacteria	k__norank_ d__Bacteria	p__Chloroflexi	c__Anaerolineae	o__Caldilineales	f__Caldilineaceae	g__norank_f__Caldilineaceae	s__unclassified_g__norank_f__Caldilineaceae	OTU1426
d__Bacteria	k__norank_ d__Bacteria	p__Chloroflexi	c__Ktedonobacteria	o__Ktedonobacterales	f__Ktedonobacteraceae	g__1921-2	s__uncultured_bacterium_g__1921-2	OTU1802
d__Bacteria	k__norank_ d__Bacteria	p__Chloroflexi	c__Chloroflexia	o__Chloroflexales	f__Roseiflexaceae	g__unclassified_f__Roseiflexaceae	s__unclassified_f__Roseiflexaceae	OTU2618
d__Bacteria	k__norank_ d__Bacteria	p__Acidobacteriota	c__Vicinamibacteria	o__Vicinamibacterales	f__Vicinamibacteraceae	g__norank_f__Vicinamibacteraceae	s__uncultured_Acidobacteriales_bacterium_g__norank_f__Vicinamibacteraceae	OTU1320
d__Bacteria	k__norank_ d__Bacteria	p__Chloroflexi	c__Ktedonobacteria	o__C0119	f__norank_o__C0119	g__norank_f__norank_o__C0119	s__unclassified_g__norank_f__norank_o__C0119	OTU2678
d__Bacteria	k__norank_ d__Bacteria	p__Patescibacteria	c__Saccharimonadia	o__Saccharimonadales	f__unclassified_o__Saccharimonadales	g__unclassified_o__Saccharimonadale s	s__unclassified_o__Saccharimonadales	OTU2049
d__Bacteria	k__norank_ d__Bacteria	p__Acidobacteriota	c__Acidobacteriae	o__Acidobacteriales	f__Acidobacteriaceae_Subgroup_1	g__norank_f__Acidobacteriaceae_Sub group_1	s__uncultured_bacterium_g__norank_f__Acidobacteriaceae_Subgroup_1	OTU1181
d__Bacteria	k__norank_ d__Bacteria	p__Firmicutes	c__Bacilli	o__Lactobacillales	f__Streptococcaceae	g__Streptococcus	s__unclassified_g__Streptococcus	OTU430
d__Bacteria	k__norank_ d__Bacteria	p__Patescibacteria	c__Saccharimonadia	o__Saccharimonadales	f__norank_o__Saccharimonadales	g__norank_f__norank_o__Saccharimo nadales	s__uncultured_bacterium_g__norank_f__norank_o__Saccharimonadales	OTU2138
d__Bacteria	k__norank_ d__Bacteria	p__Gemmatimonadota	c__Longimicrobia	o__Longimicrobiales	f__Longimicrobiaceae	g__Longimicrobium	s__uncultured_bacterium_g__Longimicrobium	OTU2805
d__Bacteria	k__norank_ d__Bacteria	p__Proteobacteria	c__Alphaproteobacteria	o__Rickettsiales	f__Mitochondria	g__norank_f__Mitochondria	s__uncultured_bacterium_g__norank_f__Mitochondria	OTU1019
d__Bacteria	k__norank_ d__Bacteria	p__Chloroflexi	c__Anaerolineae	o__Caldilineales	f__Caldilineaceae	g__norank_f__Caldilineaceae	s__unclassified_g__norank_f__Caldilineaceae	OTU360
d__Bacteria	k__norank_ d__Bacteria	p__Gemmatimonadota	c__Gemmatimonadetes	o__Gemmatimonadales	f__Gemmatimonadaceae	g__Gemmatimonas	s__unclassified_g__Gemmatimonas	OTU575

d__Bacteria	k__norank_d__Bacteria	p__Myxococcota	c__Polyangia	o__Polyangiales	f__Sandaracinaceae	g__Sandaracinus	s__unclassified_g__Sandaracinus	OTU761
d__Bacteria	k__norank_d__Bacteria	p__Myxococcota	c__Polyangia	o__Haliangiales	f__Haliangiaceae	g__Haliangium	s__unclassified_g__Haliangium	OTU1277
d__Bacteria	k__norank_d__Bacteria	p__Firmicutes	c__Bacilli	o__Paenibacillales	f__Paenibacillaceae	g__Paenibacillus	s__unclassified_g__Paenibacillus	OTU1640
d__Bacteria	k__norank_d__Bacteria	p__Armatimonadota	c__Fimbriimonadia	o__Fimbriimonadales	f__Fimbriimonadaceae	g__norank_f__Fimbriimonadaceae	s__unclassified_g__norank_f__Fimbriimonadaceae	OTU980
d__Bacteria	k__norank_d__Bacteria	p__Acidobacteriota	c__Vicinamibacteria	o__Vicinamibacterales	f__norank_o__Vicinamibacterales	g__norank_f__norank_o__Vicinamibacterales	s__unclassified_g__norank_f__norank_o__Vicinamibacterales	OTU1447
d__Bacteria	k__norank_d__Bacteria	p__Actinobacteriota	c__Thermoleophilia	o__Solirubrobacterales	f__Solirubrobacteraceae	g__unclassified_f__Solirubrobacteraceae	s__unclassified_f__Solirubrobacteraceae	OTU2424
d__Bacteria	k__norank_d__Bacteria	p__Acidobacteriota	c__Vicinamibacteria	o__Vicinamibacterales	f__norank_o__Vicinamibacterales	g__norank_f__norank_o__Vicinamibacterales	s__unclassified_g__norank_f__norank_o__Vicinamibacterales	OTU1441
d__Bacteria	k__norank_d__Bacteria	p__Planctomycetota	c__Planctomycetes	o__Isosphaerales	f__Isosphaeraceae	g__norank_f__Isosphaeraceae	s__uncultured_bacterium_g__norank_f__Isosphaeraceae	OTU2783
d__Bacteria	k__norank_d__Bacteria	p__Acidobacteriota	c__Blastocatellia	o__11-24	f__norank_o__11-24	g__norank_f__norank_o__11-24	s__unclassified_g__norank_f__norank_o__11-24	OTU743
d__Bacteria	k__norank_d__Bacteria	p__Actinobacteriota	c__Actinobacteria	o__Kineosporiales	f__Kineosporiaceae	g__Quadrisphaera	s__uncultured_bacterium_g__Quadrisphaera	OTU2401
d__Bacteria	k__norank_d__Bacteria	p__Myxococcota	c__Myxococcia	o__Myxococcales	f__Myxococcaceae	g__P3OB-42	s__uncultured_bacterium_g__P3OB-42	OTU1174
d__Bacteria	k__norank_d__Bacteria	p__Actinobacteriota	c__Thermoleophilia	o__Solirubrobacterales	f__Solirubrobacteraceae	g__Solirubrobacter	s__uncultured_bacterium_g__Solirubrobacter	OTU2473
d__Bacteria	k__norank_d__Bacteria	p__Proteobacteria	c__Alphaproteobacteria	o__Caulobacterales	f__Hyphomonadaceae	g__Hirschia	s__metagenome_g__Hirschia	OTU247
d__Bacteria	k__norank_d__Bacteria	p__Bacteroidota	c__Bacteroidia	o__Flavobacteriales	f__Flavobacteriaceae	g__Flavobacterium	s__uncultured_Bacteroidetes_bacterium_g__Flavobacterium	OTU1349
d__Bacteria	k__norank_d__Bacteria	p__Proteobacteria	c__Alphaproteobacteria	o__Sphingomonadales	f__Sphingomonadaceae	g__Sphingorhabdus	s__uncultured_bacterium_g__Sphingorhabdus	OTU2346
d__Bacteria	k__norank_d__Bacteria	p__Patescibacteria	c__Saccharimonadia	o__Saccharimonadales	f__LWQ8	g__norank_f__LWQ8	s__unclassified_g__norank_f__LWQ8	OTU684

d__Bacteria	k__norank_d__Bacteria	p__Bacteroidota	c__Bacteroidia	o__Sphingobacteriales	f__AKYH767	g__norank_f__AKYH767	s__uncultured_bacterium_g__norank_f__AKYH767	OTU2608
d__Bacteria	k__norank_d__Bacteria	p__Chloroflexi	c__Chloroflexia	o__Thermomicrobiales	f__JG30-KF-CM45	g__norank_f__JG30-KF-CM45	s__uncultured_soil_bacterium_g__norank_f__JG30-KF-CM45	OTU2699
d__Bacteria	k__norank_d__Bacteria	p__Gemmatimonadota	c__Gemmatimonadetes	o__Gemmatimonadales	f__Gemmatimonadaceae	g__Gemmatimonas	s__uncultured_Gemmatimonadaceae_bacterium_g__Gemmatimonas	OTU1478
d__Bacteria	k__norank_d__Bacteria	p__Bacteroidota	c__Bacteroidia	o__Flavobacteriales	f__Weeksellaceae	g__Chryseobacterium	s__Chryseobacterium_sp._3008163	OTU1292
d__Bacteria	k__norank_d__Bacteria	p__Cyanobacteria	c__Cyanobacteriia	o__Chloroplast	f__norank_o__Chloroplast	g__norank_f__norank_o__Chloroplast	s__unclassified_g__norank_f__norank_o__Chloroplast	OTU51
d__Bacteria	k__norank_d__Bacteria	p__Proteobacteria	c__Alphaproteobacteria	o__Sphingomonadales	f__Sphingomonadaceae	g__Sphingomonas	s__unclassified_g__Sphingomonas	OTU278
d__Bacteria	k__norank_d__Bacteria	p__Gemmatimonadota	c__Gemmatimonadetes	o__Gemmatimonadales	f__Gemmatimonadaceae	g__Gemmatimonas	s__uncultured_Gemmatimonadaceae_bacterium_g__Gemmatimonas	OTU1472
d__Bacteria	k__norank_d__Bacteria	p__Bacteroidota	c__Bacteroidia	o__Cytophagales	f__Cytophagaceae	g__Rhodocytophaga	s__unclassified_g__Rhodocytophaga	OTU2658
d__Bacteria	k__norank_d__Bacteria	p__Proteobacteria	c__Gammaproteobacteria	o__Burkholderiales	f__SC-I-84	g__norank_f__SC-I-84	s__uncultured_soil_bacterium_g__norank_f__SC-I-84	OTU882
d__Bacteria	k__norank_d__Bacteria	p__Actinobacteriota	c__Acidimicrobiia	o__Microtrichales	f__norank_o__Microtrichales	g__norank_f__norank_o__Microtrichales	s__metagenome_g__norank_f__norank_o__Microtrichales	OTU2646
d__Bacteria	k__norank_d__Bacteria	p__Cyanobacteria	c__Cyanobacteriia	o__Chloroplast	f__norank_o__Chloroplast	g__norank_f__norank_o__Chloroplast	s__Bacillaria_paxillifer	OTU2973
d__Bacteria	k__norank_d__Bacteria	p__Cyanobacteria	c__Cyanobacteriia	o__Chloroplast	f__norank_o__Chloroplast	g__norank_f__norank_o__Chloroplast	s__Bacillaria_paxillifer	OTU2974
d__Bacteria	k__norank_d__Bacteria	p__Proteobacteria	c__Gammaproteobacteria	o__Steroidobacterales	f__Steroidobacteraceae	g__norank_f__Steroidobacteraceae	s__uncultured_bacterium_g__norank_f__Steroidobacteraceae	OTU1743
d__Bacteria	k__norank_d__Bacteria	p__Proteobacteria	c__Gammaproteobacteria	o__Burkholderiales	f__Comamonadaceae	g__Ramlibacter	s__uncultured_bacterium_g__Ramlibacter	OTU1674
d__Bacteria	k__norank_d__Bacteria	p__Proteobacteria	c__Alphaproteobacteria	o__Micropepsales	f__Micropepsaceae	g__norank_f__Micropepsaceae	s__unclassified_g__norank_f__Micropepsaceae	OTU1372
d__Bacteria	k__norank_d__Bacteria	p__Verrucomicrobiota	c__Chlamydiae	o__Chlamydiales	f__Parachlamydiaceae	g__Neochlamydia	s__uncultured_Chlamydiales_bacterium_g__Neochlamydia	OTU1317

d__Bacteria	k__norank_ d__Bacteria	p__Myxococcota	c__Myxococcia	o__Myxococcales	f__Anaeromyxobacteraceae	g__Anaeromyxobacter	s__uncultured_delta_proteobacterium_g__Anaeromyxobacter	OTU2406
d__Bacteria	k__norank_ d__Bacteria	p__Bacteroidota	c__Bacteroidia	o__Chitinophagales	f__Chitinophagaceae	g__Ferruginibacter	s__unclassified_g__Ferruginibacter	OTU221
d__Bacteria	k__norank_ d__Bacteria	p__Proteobacteria	c__Alphaproteobacteria	o__Elsterales	f__norank_o__Elsterales	g__norank_f__norank_o__Elsterales	s__unclassified_g__norank_f__norank_o__Elsterales	OTU1886
d__Bacteria	k__norank_ d__Bacteria	p__Proteobacteria	c__Alphaproteobacteria	o__Acetobacterales	f__Acetobacteraceae	g__unclassified_f__Acetobacteraceae	s__unclassified_f__Acetobacteraceae	OTU1756
d__Bacteria	k__norank_ d__Bacteria	p__Proteobacteria	c__Alphaproteobacteria	o__Rhizobiales	f__Xanthobacteraceae	g__norank_f__Xanthobacteraceae	s__unclassified_g__norank_f__Xanthobacteraceae	OTU1024
d__Bacteria	k__norank_ d__Bacteria	p__Bdellovibrionota	c__Oligoflexia	o__0319-6G20	f__norank_o__0319-6G20	g__norank_f__norank_o__0319-6G20	s__uncultured_bacterium_g__norank_f__norank_o__0319-6G20	OTU194
d__Bacteria	k__norank_ d__Bacteria	p__Chloroflexi	c__Anaerolineae	o__SBR1031	f__A4b	g__norank_f__A4b	s__unclassified_g__norank_f__A4b	OTU1258
d__Bacteria	k__norank_ d__Bacteria	p__unclassified_k__no rank_d__Bacteria	c__unclassified_k__noran k_d__Bacteria	o__unclassified_k__norank_ d__Bacteria	f__unclassified_k__norank_d__Bacteria	g__unclassified_k__norank_d__Bacteri a	s__unclassified_k__norank_d__Bacteria	OTU2240
d__Bacteria	k__norank_ d__Bacteria	p__Planctomycetota	c__BD7-11	o__norank_c__BD7-11	f__norank_o__norank_c__BD7-11	g__norank_f__norank_o__norank_c__ BD7-11	s__uncultured_bacterium_g__norank_f__norank_o__norank_c__BD7-11	OTU794
d__Bacteria	k__norank_ d__Bacteria	p__Bdellovibrionota	c__Bdellovibrionia	o__Bdellovibrionales	f__Bdellovibrionaceae	g__Bdellovibrio	s__unclassified_g__Bdellovibrio	OTU20
d__Bacteria	k__norank_ d__Bacteria	p__Proteobacteria	c__Gammaproteobacteria	o__Burkholderiales	f__Comamonadaceae	g__Leptothrix	s__metagenome_g__Leptothrix	OTU1336
d__Bacteria	k__norank_ d__Bacteria	p__Patescibacteria	c__Saccharimonadia	o__Saccharimonadales	f__norank_o__Saccharimonadales	g__norank_f__norank_o__Saccharimo nadales	s__uncultured_bacterium_g__norank_f__norank_o__Saccharimonadales	OTU590
d__Bacteria	k__norank_ d__Bacteria	p__Chloroflexi	c__Ktedonobacteria	o__Ktedonobacterales	f__Ktedonobacteraceae	g__HSB_OF53-F07	s__uncultured_Ktedonobacter_sp._g__HSB_OF53-F07	OTU1463
d__Bacteria	k__norank_ d__Bacteria	p__Chloroflexi	c__AD3	o__norank_c__AD3	f__norank_o__norank_c__AD3	g__norank_f__norank_o__norank_c__ AD3	s__uncultured_Chloroflexi_bacterium_g__norank_f__norank_o__norank_c__AD3	OTU1169
d__Bacteria	k__norank_ d__Bacteria	p__Bacteroidota	c__Bacteroidia	o__Cytophagales	f__Spirosomaceae	g__Fibrella	s__uncultured_bacterium_g__Fibrella	OTU1668
d__Bacteria	k__norank_ d__Bacteria	p__Actinobacteriota	c__Thermoleophilia	o__Gaiellales	f__norank_o__Gaiellales	g__norank_f__norank_o__Gaiellales	s__unclassified_g__norank_f__norank_o__Gaiellales	OTU1796

d__Bacteria	k__norank_ d__Bacteria	p__Acidobacteriota	c__Blastocatellia	o__11-24	f__norank_o__11-24	g__norank_f__norank_o__11-24	s__unclassified_g__norank_f__norank_o__11-24	OTU1535
d__Bacteria	k__norank_ d__Bacteria	p__Acidobacteriota	c__Acidobacteriae	o__Bryobacterales	f__Bryobacteraceae	g__Bryobacter	s__unclassified_g__Bryobacter	OTU1120
d__Bacteria	k__norank_ d__Bacteria	p__Proteobacteria	c__Alphaproteobacteria	o__Acetobacterales	f__Acetobacteraceae	g__unclassified_f__Acetobacteraceae	s__unclassified_f__Acetobacteraceae	OTU1950
d__Bacteria	k__norank_ d__Bacteria	p__Bacteroidota	c__Bacteroidia	o__Flavobacteriales	f__Flavobacteriaceae	g__Myroides	s__uncultured_bacterium_g__Myroides	OTU2963
d__Bacteria	k__norank_ d__Bacteria	p__Patescibacteria	c__Saccharimonadia	o__Saccharimonadales	f__unclassified_o__Saccharimonadales	g__unclassified_o__Saccharimonadale s	s__unclassified_o__Saccharimonadales	OTU292
d__Bacteria	k__norank_ d__Bacteria	p__Cyanobacteria	c__Sericytochromatia	o__norank_c__Sericytochro matia	f__norank_o__norank_c__Sericytochrom atia	g__norank_f__norank_o__norank_c__ Sericytochromatia	s__uncultured_bacterium_g__norank_f__norank_o__norank_c__Sericytochromatia	OTU1677
d__Bacteria	k__norank_ d__Bacteria	p__Patescibacteria	c__Saccharimonadia	o__Saccharimonadales	f__norank_o__Saccharimonadales	g__norank_f__norank_o__Saccharimo nadales	s__unclassified_g__norank_f__norank_o__Saccharimonadales	OTU2790
d__Bacteria	k__norank_ d__Bacteria	p__Chloroflexi	c__Ktedonobacteria	o__Ktedonobacterales	f__Ktedonobacteraceae	g__norank_f__Ktedonobacteraceae	s__uncultured_bacterium_g__norank_f__Ktedonobacteraceae	OTU17
d__Bacteria	k__norank_ d__Bacteria	p__Planctomycetota	c__Planctomycetes	o__Isosphaerales	f__Isosphaeraceae	g__norank_f__Isosphaeraceae	s__uncultured_bacterium_g__norank_f__Isosphaeraceae	OTU1124
d__Bacteria	k__norank_ d__Bacteria	p__Planctomycetota	c__Planctomycetes	o__Isosphaerales	f__Isosphaeraceae	g__norank_f__Isosphaeraceae	s__unclassified_g__norank_f__Isosphaeraceae	OTU690
d__Bacteria	k__norank_ d__Bacteria	p__Chloroflexi	c__Anaerolineae	o__SBR1031	f__norank_o__SBR1031	g__norank_f__norank_o__SBR1031	s__uncultured_soil_bacterium_g__norank_f__norank_o__SBR1031	OTU1362
d__Bacteria	k__norank_ d__Bacteria	p__Patescibacteria	c__Saccharimonadia	o__Saccharimonadales	f__LWQ8	g__norank_f__LWQ8	s__unclassified_g__norank_f__LWQ8	OTU1449
d__Bacteria	k__norank_ d__Bacteria	p__Proteobacteria	c__Alphaproteobacteria	o__Rhodospirillales	f__Rhodospirillaceae	g__norank_f__Rhodospirillaceae	s__metagenome_g__norank_f__Rhodospirillaceae	OTU2581
d__Bacteria	k__norank_ d__Bacteria	p__Bacteroidota	c__Bacteroidia	o__Chitinophagales	f__Chitinophagaceae	g__Flavisolibacter	s__unclassified_g__Flavisolibacter	OTU2990
d__Bacteria	k__norank_ d__Bacteria	p__Bdellovibrionota	c__Oligoflexia	o__0319-6G20	f__norank_o__0319-6G20	g__norank_f__norank_o__0319-6G20	s__uncultured_Roseobacter_sp._g__norank_f__norank	OTU2721
d__Bacteria	k__norank_ d__Bacteria	p__Actinobacteriota	c__Thermoleophilia	o__Solirubrobacterales	f__67-14	g__norank_f__67-14	s__metagenome_g__norank_f__67-14	OTU2461

d__Bacteria	k__norank_d__Bacteria	p__Chloroflexi	c__Ktedonobacteria	o__Ktedonobacterales	f__Ktedonobacteraceae	g__1921-2	s__unclassified_g__1921-2	OTU1900
d__Bacteria	k__norank_d__Bacteria	p__Acidobacteriota	c__Blastocatellia	o__Pyrinomonadales	f__Pyrinomonadaceae	g__RB41	s__metagenome_g__RB41	OTU2236
d__Bacteria	k__norank_d__Bacteria	p__Cyanobacteria	c__Vampirivibrionia	o__Obscuribacterales	f__Obscuribacteraceae	g__norank_f__Obscuribacteraceae	s__uncultured_bacterium_g__norank_f__Obscuribacteraceae	OTU1154
d__Bacteria	k__norank_d__Bacteria	p__Cyanobacteria	c__Cyanobacteriia	o__Chloroplast	f__norank_o__Chloroplast	g__norank_f__norank_o__Chloroplast	s__unclassified_g__norank_f__norank_o__Chloroplast	OTU2941
d__Bacteria	k__norank_d__Bacteria	p__Actinobacteriota	c__Thermoleophilia	o__Solirubrobacterales	f__67-14	g__norank_f__67-14	s__unclassified_g__norank_f__67-14	OTU2453
d__Bacteria	k__norank_d__Bacteria	p__Actinobacteriota	c__Thermoleophilia	o__Solirubrobacterales	f__67-14	g__norank_f__67-14	s__unclassified_g__norank_f__67-14	OTU2450
d__Bacteria	k__norank_d__Bacteria	p__Actinobacteriota	c__Actinobacteria	o__Propionibacteriales	f__Propionibacteriaceae	g__Micropruina	s__unclassified_g__Micropruina	OTU668
d__Bacteria	k__norank_d__Bacteria	p__Actinobacteriota	c__Acidimicrobiia	o__IMCC26256	f__norank_o__IMCC26256	g__norank_f__norank_o__IMCC26256	s__unclassified_g__norank_f__norank_o__IMCC26256	OTU1262
d__Bacteria	k__norank_d__Bacteria	p__Patescibacteria	c__Saccharimonadia	o__Saccharimonadales	f__norank_o__Saccharimonadales	g__norank_f__norank_o__Saccharimonadales	s__unclassified_g__norank_f__norank_o__Saccharimonadales	OTU1245
d__Bacteria	k__norank_d__Bacteria	p__Proteobacteria	c__Gammaproteobacteria	o__Diplorickettsiales	f__Diplorickettsiaceae	g__norank_f__Diplorickettsiaceae	s__uncultured_bacterium_g__norank_f__Diplorickettsiaceae	OTU1330
d__Bacteria	k__norank_d__Bacteria	p__Actinobacteriota	c__Actinobacteria	o__Corynebacteriales	f__Nocardiaceae	g__Rhodococcus	s__Rhodococcus_erythropolis	OTU2675
d__Bacteria	k__norank_d__Bacteria	p__Proteobacteria	c__Gammaproteobacteria	o__Burkholderiales	f__Burkholderiaceae	g__Burkholderia-Caballeronia-Paraburkholderia	s__unclassified_g__Burkholderia-Caballeronia-Paraburkholderia	OTU38
d__Bacteria	k__norank_d__Bacteria	p__Proteobacteria	c__Gammaproteobacteria	o__Xanthomonadales	f__Xanthomonadaceae	g__Arenimonas	s__uncultured_bacterium_g__Arenimonas	OTU2135
d__Bacteria	k__norank_d__Bacteria	p__Bdellovibrionota	c__Oligoflexia	o__Oligoflexales	f__norank_o__Oligoflexales	g__Oligoflexus	s__uncultured_bacterium_g__Oligoflexus	OTU1002
d__Bacteria	k__norank_d__Bacteria	p__Proteobacteria	c__Gammaproteobacteria	o__Burkholderiales	f__Comamonadaceae	g__unclassified_f__Comamonadaceae	s__unclassified_f__Comamonadaceae	OTU123
d__Bacteria	k__norank_d__Bacteria	p__Firmicutes	c__Clostridia	o__Lachnospirales	f__Lachnospiraceae	g__Stomatobaculum	s__uncultured_bacterium_g__Stomatobaculum	OTU638

d__Bacteria	k__norank_ d__Bacteria	p__Bdellovibrionota	c__Oligoflexia	o__0319-6G20	f__norank_o__0319-6G20	g__norank_f__norank_o__0319-6G20	s__metagenome_g__norank_f__norank_o__0319-6G20	OTU2715
d__Bacteria	k__norank_ d__Bacteria	p__Actinobacteriota	c__Acidimicrobiia	o__Microtrichales	f__norank_o__Microtrichales	g__norank_f__norank_o__Microtrichal es	s__unclassified_g__norank_f__norank_o__Microtrichales	OTU1812
d__Bacteria	k__norank_ d__Bacteria	p__Myxococcota	c__Polyangia	o__Polyangiales	f__BIrii41	g__norank_f__BIrii41	s__metagenome_g__norank_f__BIrii41	OTU2261
d__Bacteria	k__norank_ d__Bacteria	p__Verrucomicrobiota	c__Chlamydiae	o__Chlamydiales	f__Parachlamydiaceae	g__Candidatus_Protochlamydia	s__unclassified_g__Candidatus_Protochlamydia	OTU2818
d__Bacteria	k__norank_ d__Bacteria	p__Bdellovibrionota	c__Oligoflexia	o__0319-6G20	f__norank_o__0319-6G20	g__norank_f__norank_o__0319-6G20	s__marine_metagenome_g__norank_f__norank_o__0319-6G20	OTU1165
d__Bacteria	k__norank_ d__Bacteria	p__Bacteroidota	c__Bacteroidia	o__Cytophagales	f__Spirosomaceae	g__Spirosoma	s__Spirosoma_rigui	OTU2512
d__Bacteria	k__norank_ d__Bacteria	p__Actinobacteriota	c__Actinobacteria	o__Propionibacteriales	f__Propionibacteriaceae	g__Luteococcus	s__uncultured_bacterium_g__Luteococcus	OTU2812
d__Bacteria	k__norank_ d__Bacteria	p__Planctomycetota	c__Planctomycetes	o__Isosphaerales	f__Isosphaeraceae	g__Candidatus_Nostocoida	s__uncultured_bacterium_g__Candidatus_Nostocoida	OTU957
d__Bacteria	k__norank_ d__Bacteria	p__Bacteroidota	c__Bacteroidia	o__Chitinophagales	f__Saprospiraceae	g__Phaeodactylibacter	s__unclassified_g__Phaeodactylibacter	OTU308
d__Bacteria	k__norank_ d__Bacteria	p__Bdellovibrionota	c__Bdellovibrionia	o__Bdellovibrionales	f__Bdellovibrionaceae	g__OM27_clade	s__unclassified_g__OM27_clade	OTU450
d__Bacteria	k__norank_ d__Bacteria	p__Proteobacteria	c__Gammaproteobacteria	o__Diplorickettsiales	f__Diplorickettsiaceae	g__Aquicella	s__uncultured_bacterium_g__Aquicella	OTU2696
d__Bacteria	k__norank_ d__Bacteria	p__Bdellovibrionota	c__Bdellovibrionia	o__Bdellovibrionales	f__Bdellovibrionaceae	g__Bdellovibrio	s__unclassified_g__Bdellovibrio	OTU2193
d__Bacteria	k__norank_ d__Bacteria	p__Armatimonadota	c__Armatimonadia	o__Armatimonadales	f__norank_o__Armatimonadales	g__norank_f__norank_o__Armatimona dales	s__uncultured_bacterium_g__norank_f__norank_o__Armatimonadales	OTU933
d__Bacteria	k__norank_ d__Bacteria	p__Bacteroidota	c__Bacteroidia	o__Chitinophagales	f__Chitinophagaceae	g__Cnuella	s__uncultured_Sphingobacteriales_bacterium_g__Cnuella	OTU655
d__Bacteria	k__norank_ d__Bacteria	p__Armatimonadota	c__norank_p__Armatimon adota	o__norank_c__norank_p__ Armatimonadota	f__norank_o__norank_c__norank_p__Ar matimonadota	g__norank_f__norank_o__norank_c__ norank_p__Armatimonadota	s__unclassified_g__norank_f__norank_o__norank_c__norank_p__Armatimonadota	OTU1074
d__Bacteria	k__norank_ d__Bacteria	p__Proteobacteria	c__Gammaproteobacteria	o__Burkholderiales	f__A21b	g__norank_f__A21b	s__unclassified_g__norank_f__A21b	OTU862

d__Bacteria	k__norank_ d__Bacteria	p__Acidobacteriota	c__Acidobacteriae	o__Bryobacterales	f__Bryobacteraceae	g__Bryobacter	s__unclassified_g__Bryobacter	OTU674
d__Bacteria	k__norank_ d__Bacteria	p__Chloroflexi	c__TK10	o__norank_c__TK10	f__norank_o__norank_c__TK10	g__norank_f__norank_o__norank_c__ TK10	s__uncultured_bacterium_g__norank_f__norank_o__norank_c__TK10	OTU2627
d__Bacteria	k__norank_ d__Bacteria	p__Bdellovibrionota	c__Oligoflexia	o__Oligoflexales	f__norank_o__Oligoflexales	g__norank_f__norank_o__Oligoflexale s	s__unclassified_g__norank_f__norank_o__Oligoflexales	OTU504
d__Bacteria	k__norank_ d__Bacteria	p__Actinobacteriota	c__Acidimicrobiia	o__Microtrichales	f__Microtrichaceae	g__norank_f__Microtrichaceae	s__uncultured_bacterium_g__norank_f__Microtrichaceae	OTU25
d__Bacteria	k__norank_ d__Bacteria	p__Verrucomicrobiota	c__Verrucomicrobiae	o__Verrucomicrobiales	f__Rubritaleaceae	g__Luteolibacter	s__uncultured_bacterium_g__Luteolibacter	OTU2078
d__Bacteria	k__norank_ d__Bacteria	p__Actinobacteriota	c__Acidimicrobiia	o__IMCC26256	f__norank_o__IMCC26256	g__norank_f__norank_o__IMCC26256	s__metagenome_g__norank_f__norank_o__IMCC26256	OTU1509
d__Bacteria	k__norank_ d__Bacteria	p__Dependentiae	c__Babeliae	o__Babeliales	f__Vermiphilaceae	g__norank_f__Vermiphilaceae	s__uncultured_bacterium_g__norank_f__Vermiphilaceae	OTU1433
d__Bacteria	k__norank_ d__Bacteria	p__Proteobacteria	c__Alphaproteobacteria	o__Rhizobiales	f__Hyphomicrobiaceae	g__Hyphomicrobium	s__uncultured_bacterium_g__Hyphomicrobium	OTU2506
d__Bacteria	k__norank_ d__Bacteria	p__Myxococcota	c__Polyangia	o__Haliangiales	f__Haliangiaceae	g__Haliangium	s__uncultured_bacterium_g__Haliangium	OTU845
d__Bacteria	k__norank_ d__Bacteria	p__Bacteroidota	c__SJA-28	o__norank_c__SJA-28	f__norank_o__norank_c__SJA-28	g__norank_f__norank_o__norank_c__ SJA-28	s__uncultured_Ignavibacterium_sp._g__norank_f__norank	OTU2220
d__Bacteria	k__norank_ d__Bacteria	p__Firmicutes	c__Clostridia	o__norank_c__Clostridia	f__Gracilibacteraceae	g__Lutispora	s__uncultured_bacterium_g__Lutispora	OTU2103
d__Bacteria	k__norank_ d__Bacteria	p__Bdellovibrionota	c__Bdellovibrionia	o__Bacteriovoracales	f__Bacteriovoracaceae	g__Peredibacter	s__metagenome_g__Peredibacter	OTU1881
d__Bacteria	k__norank_ d__Bacteria	p__WPS-2	c__norank_p__WPS-2	o__norank_c__norank_p__ WPS-2	f__norank_o__norank_c__norank_p__W PS-2	g__norank_f__norank_o__norank_c__ norank_p__WPS-2	s__metagenome_g__norank_f__norank_o__norank_c__norank_p__WPS-2	OTU143
d__Bacteria	k__norank_ d__Bacteria	p__Proteobacteria	c__Gammaproteobacteria	o__Acidiferrobacterales	f__Acidiferrobacteraceae	g__norank_f__Acidiferrobacteraceae	s__uncultured_bacterium_g__norank_f__Acidiferrobacteraceae	OTU40
d__Bacteria	k__norank_ d__Bacteria	p__Bacteroidota	c__Bacteroidia	o__Sphingobacteriales	f__AKYH767	g__norank_f__AKYH767	s__uncultured_bacterium_g__norank_f__AKYH767	OTU846
d__Bacteria	k__norank_ d__Bacteria	p__Actinobacteriota	c__Thermoleophilia	o__Solirubrobacterales	f__Solirubrobacteraceae	g__Patulibacter	s__Patulibacter_ginsengiterrae	OTU800

d__Bacteria	k__norank_d__Bacteria	p__Bacteroidota	c__Bacteroidia	o__Cytophagales	f__norank_o__Cytophagales	g__norank_f__norank_o__Cytophagales	s__uncultured_bacterium_g__norank_f__norank_o__Cytophagales	OTU1399
d__Bacteria	k__norank_d__Bacteria	p__Armatimonadota	c__Fimbriimonadia	o__Fimbriimonadales	f__Fimbriimonadaceae	g__norank_f__Fimbriimonadaceae	s__unclassified_g__norank_f__Fimbriimonadaceae	OTU667
d__Bacteria	k__norank_d__Bacteria	p__Bacteroidota	c__Bacteroidia	o__Cytophagales	f__Cytophagaceae	g__unclassified_f__Cytophagaceae	s__unclassified_f__Cytophagaceae	OTU1234
d__Bacteria	k__norank_d__Bacteria	p__Proteobacteria	c__Alphaproteobacteria	o__Caulobacterales	f__Caulobacteraceae	g__Caulobacter	s__unclassified_g__Caulobacter	OTU514
d__Bacteria	k__norank_d__Bacteria	p__Bacteroidota	c__Bacteroidia	o__Cytophagales	f__Hymenobacteraceae	g__Hymenobacter	s__unclassified_g__Hymenobacter	OTU2268
d__Bacteria	k__norank_d__Bacteria	p__Proteobacteria	c__Gammaproteobacteria	o__Xanthomonadales	f__Rhodanobacteraceae	g__norank_f__Rhodanobacteraceae	s__uncultured_bacterium_g__norank_f__Rhodanobacteraceae	OTU2357
d__Bacteria	k__norank_d__Bacteria	p__Deinococcota	c__Deinococci	o__Deinococcales	f__Deinococcaceae	g__Deinococcus	s__unclassified_g__Deinococcus	OTU2547
d__Bacteria	k__norank_d__Bacteria	p__Proteobacteria	c__Gammaproteobacteria	o__Burkholderiales	f__Burkholderiaceae	g__Lautropia	s__metagenome_g__Lautropia	OTU1311
d__Bacteria	k__norank_d__Bacteria	p__Acidobacteriota	c__Holophagae	o__Subgroup_7	f__norank_o__Subgroup_7	g__norank_f__norank_o__Subgroup_7	s__uncultured_Acidobacteria_bacterium_g__norank_f__norank_o__Subgroup_7	OTU1546
d__Bacteria	k__norank_d__Bacteria	p__Bacteroidota	c__Bacteroidia	o__Chitinophagales	f__Chitinophagaceae	g__Terrimonas	s__unclassified_g__Terrimonas	OTU1595
d__Bacteria	k__norank_d__Bacteria	p__Actinobacteriota	c__Actinobacteria	o__Micrococcales	f__Micrococcaceae	g__Pseudarthrobacter	s__unclassified_g__Pseudarthrobacter	OTU541
d__Bacteria	k__norank_d__Bacteria	p__Patescibacteria	c__Parcubacteria	o__Candidatus_Kaiserbacteria	f__norank_o__Candidatus_Kaiserbacteria	g__norank_f__norank_o__Candidatus_Kaiserbacteria	s__Parcubacteria_group_bacterium_CG1_02_50_68	OTU2939
d__Bacteria	k__norank_d__Bacteria	p__Gemmatimonadota	c__Longimicrobia	o__Longimicrobiales	f__Longimicrobiaceae	g__Longimicrobium	s__uncultured_bacterium_g__Longimicrobium	OTU232
d__Bacteria	k__norank_d__Bacteria	p__Armatimonadota	c__norank_p__Armatimonadota	o__norank_c__norank_p__Armatimonadota	f__norank_o__norank_c__norank_p__Armatimonadota	g__norank_f__norank_o__norank_c__norank_p__Armatimonadota	s__unclassified_g__norank_f__norank_o__norank_c__norank_p__Armatimonadota	OTU1153
d__Bacteria	k__norank_d__Bacteria	p__Proteobacteria	c__Alphaproteobacteria	o__Sphingomonadales	f__Sphingomonadaceae	g__Altererythrobacter	s__metagenome_g__Altererythrobacter	OTU2612
d__Bacteria	k__norank_d__Bacteria	p__Deinococcota	c__Deinococci	o__Deinococcales	f__Deinococcaceae	g__Deinococcus	s__Deinococcus_sp._R-36479	OTU58

d__Bacteria	k__norank_ d__Bacteria	p__Firmicutes	c__Bacilli	o__Bacillales	f__Planococcaceae	g__Caryophanon	s__uncultured_bacterium_g__Caryophanon	OTU1288
d__Bacteria	k__norank_ d__Bacteria	p__Proteobacteria	c__Gammaproteobacteria	o__Legionellales	f__Legionellaceae	g__Legionella	s__unclassified_g__Legionella	OTU2413
d__Bacteria	k__norank_ d__Bacteria	p__Cyanobacteria	c__Cyanobacteriia	o__Chloroplast	f__norank_o__Chloroplast	g__norank_f__norank_o__Chloroplast	s__Oogamochlamys_gigantea	OTU136
d__Bacteria	k__norank_ d__Bacteria	p__Chloroflexi	c__AD3	o__norank_c__AD3	f__norank_o__norank_c__AD3	g__norank_f__norank_o__norank_c__ AD3	s__uncultured_Chloroflexi_bacterium_g__norank_f__norank_o__norank_c__AD3	OTU2519
d__Bacteria	k__norank_ d__Bacteria	p__Gemmatimonadota	c__Gemmatimonadetes	o__Gemmatimonadales	f__Gemmatimonadaceae	g__unclassified_f__Gemmatimonadace ae	s__unclassified_f__Gemmatimonadaceae	OTU786
d__Bacteria	k__norank_ d__Bacteria	p__Chloroflexi	c__Anaerolineae	o__SBR1031	f__A4b	g__norank_f__A4b	s__uncultured_bacterium_g__norank_f__A4b	OTU2243
d__Bacteria	k__norank_ d__Bacteria	p__Proteobacteria	c__Gammaproteobacteria	o__Burkholderiales	f__A21b	g__norank_f__A21b	s__uncultured_bacterium_g__norank_f__A21b	OTU2656
d__Bacteria	k__norank_ d__Bacteria	p__Bdellovibrionota	c__Oligoflexia	o__0319-6G20	f__norank_o__0319-6G20	g__norank_f__norank_o__0319-6G20	s__unclassified_g__norank_f__norank_o__0319-6G20	OTU2813
d__Bacteria	k__norank_ d__Bacteria	p__Cyanobacteria	c__Vampirivibrionia	o__Obscuribacterales	f__Obscuribacteraceae	g__norank_f__Obscuribacteraceae	s__uncultured_bacterium_g__norank_f__Obscuribacteraceae	OTU410
d__Bacteria	k__norank_ d__Bacteria	p__Chloroflexi	c__Ktedonobacteria	o__B12-WMSP1	f__norank_o__B12-WMSP1	g__norank_f__norank_o__B12-WMSP 1	s__unclassified_g__norank_f__norank_o__B12-WMSP1	OTU1015
d__Bacteria	k__norank_ d__Bacteria	p__Proteobacteria	c__Gammaproteobacteria	o__Coxiellales	f__Coxiellaceae	g__Coxiella	s__unclassified_g__Coxiella	OTU1117
d__Bacteria	k__norank_ d__Bacteria	p__Myxococcota	c__Polyangia	o__Polyangiales	f__Polyangiaceae	g__Pajaroellobacter	s__uncultured_bacterium_g__Pajaroellobacter	OTU753
d__Bacteria	k__norank_ d__Bacteria	p__Acidobacteriota	c__Vicinamibacteria	o__Vicinamibacterales	f__norank_o__Vicinamibacterales	g__norank_f__norank_o__Vicinamiba cterales	s__unclassified_g__norank_f__norank_o__Vicinamibacterales	OTU2509
d__Bacteria	k__norank_ d__Bacteria	p__Chloroflexi	c__Anaerolineae	o__SBR1031	f__norank_o__SBR1031	g__norank_f__norank_o__SBR1031	s__uncultured_Caldilineaceae_bacterium_g__norank_f__norank_o__SBR1031	OTU2369
d__Bacteria	k__norank_ d__Bacteria	p__Planctomycetota	c__vadinHA49	o__norank_c__vadinHA49	f__norank_o__norank_c__vadinHA49	g__norank_f__norank_o__norank_c__ vadinHA49	s__unclassified_g__norank_f__norank_o__norank_c__vadinHA49	OTU812
d__Bacteria	k__norank_ d__Bacteria	p__Myxococcota	c__Polyangia	o__Haliangiales	f__Haliangiaceae	g__Haliangium	s__unclassified_g__Haliangium	OTU1003

d__Bacteria	k__norank_d__Bacteria	p__Acidobacteriota	c__Blastocatellia	o__Blastocatellales	f__Blastocatellaceae	g__JGI_0001001-H03	s__metagenome_g__JGI_0001001-H03	OTU1073
d__Bacteria	k__norank_d__Bacteria	p__Bdellovibrionota	c__Oligoflexia	o__Silvanigrellales	f__Silvanigrellaceae	g__norank_f__Silvanigrellaceae	s__uncultured_bacterium_g__norank_f__Silvanigrellaceae	OTU2604
d__Bacteria	k__norank_d__Bacteria	p__Patescibacteria	c__Saccharimonadia	o__Saccharimonadales	f__norank_o__Saccharimonadales	g__norank_f__norank_o__Saccharimonadales	s__metagenome_g__norank_f__norank_o__Saccharimonadales	OTU2690
d__Bacteria	k__norank_d__Bacteria	p__Chloroflexi	c__Chloroflexia	o__Elev-1554	f__norank_o__Elev-1554	g__norank_f__norank_o__Elev-1554	s__uncultured_bacterium_g__norank_f__norank_o__Elev-1554	OTU1095
d__Bacteria	k__norank_d__Bacteria	p__Proteobacteria	c__Alphaproteobacteria	o__Acetobacterales	f__Acetobacteraceae	g__unclassified_f__Acetobacteraceae	s__unclassified_f__Acetobacteraceae	OTU237
d__Bacteria	k__norank_d__Bacteria	p__Patescibacteria	c__Saccharimonadia	o__Saccharimonadales	f__norank_o__Saccharimonadales	g__norank_f__norank_o__Saccharimonadales	s__uncultured_bacterium_g__norank_f__norank_o__Saccharimonadales	OTU2554
d__Bacteria	k__norank_d__Bacteria	p__Acidobacteriota	c__Holophagae	o__Subgroup_7	f__norank_o__Subgroup_7	g__norank_f__norank_o__Subgroup_7	s__unclassified_g__norank_f__norank_o__Subgroup_7	OTU681
d__Bacteria	k__norank_d__Bacteria	p__Acidobacteriota	c__Holophagae	o__Subgroup_7	f__norank_o__Subgroup_7	g__norank_f__norank_o__Subgroup_7	s__unclassified_g__norank_f__norank_o__Subgroup_7	OTU935
d__Bacteria	k__norank_d__Bacteria	p__Chloroflexi	c__Anaerolineae	o__RBG-13-54-9	f__norank_o__RBG-13-54-9	g__norank_f__norank_o__RBG-13-54-9	s__uncultured_sludge_bacterium_A31	OTU1454
d__Bacteria	k__norank_d__Bacteria	p__Patescibacteria	c__Saccharimonadia	o__Saccharimonadales	f__LWQ8	g__norank_f__LWQ8	s__uncultured_bacterium_g__norank_f__LWQ8	OTU304
d__Bacteria	k__norank_d__Bacteria	p__Bacteroidota	c__Bacteroidia	o__Cytophagales	f__Hymenobacteraceae	g__Hymenobacter	s__unclassified_g__Hymenobacter	OTU116
d__Bacteria	k__norank_d__Bacteria	p__Actinobacteriota	c__Thermoleophilia	o__Solirubrobacterales	f__Solirubrobacteraceae	g__Conexibacter	s__Solirubrobacterales_bacterium_g__Conexibacter	OTU2971
d__Bacteria	k__norank_d__Bacteria	p__Actinobacteriota	c__Actinobacteria	o__Frankiales	f__Nakamurellaceae	g__Nakamurella	s__metagenome_g__Nakamurella	OTU1458
d__Bacteria	k__norank_d__Bacteria	p__Firmicutes	c__Clostridia	o__Clostridiales	f__Clostridiaceae	g__Clostridium_sensu_stricto_13	s__Clostridium_estertheticum	OTU2351
d__Bacteria	k__norank_d__Bacteria	p__Bdellovibrionota	c__Bdellovibrionia	o__Bacteriovoracales	f__Bacteriovoracaceae	g__Peredibacter	s__unclassified_g__Peredibacter	OTU183
d__Bacteria	k__norank_d__Bacteria	p__Patescibacteria	c__Saccharimonadia	o__Saccharimonadales	f__Saccharimonadaceae	g__TM7x	s__uncultured_bacterium_g__TM7x	OTU642

d__Bacteria	k__norank_ d__Bacteria	p__Acidobacteriota	c__Vicinamibacteria	o__Subgroup_17	f__norank_o__Subgroup_17	g__norank_f__norank_o__Subgroup_1 7	s__uncultured_bacterium_g__norank_f__norank_o__Subgroup_17	OTU601
d__Bacteria	k__norank_ d__Bacteria	p__Chloroflexi	c__TK10	o__norank_c__TK10	f__norank_o__norank_c__TK10	g__norank_f__norank_o__norank_c__ TK10	s__unclassified_g__norank_f__norank_o__norank_c__TK10	OTU1523
d__Bacteria	k__norank_ d__Bacteria	p__Proteobacteria	c__Alphaproteobacteria	o__Acetobacterales	f__Acetobacteraceae	g__Acidiphilium	s__uncultured_soil_bacterium_g__Acidiphilium	OTU1854
d__Bacteria	k__norank_ d__Bacteria	p__Deinococcota	c__Deinococci	o__Deinococcales	f__Deinococcaceae	g__Deinococcus	s__Deinococcus_aerolatus	OTU2861
d__Bacteria	k__norank_ d__Bacteria	p__Planctomycetota	c__vadinHA49	o__norank_c__vadinHA49	f__norank_o__norank_c__vadinHA49	g__norank_f__norank_o__norank_c__ vadinHA49	s__uncultured_bacterium_g__norank_f__norank_o__norank_c__vadinHA49	OTU302
d__Bacteria	k__norank_ d__Bacteria	p__Chloroflexi	c__Chloroflexia	o__Chloroflexales	f__Herpetosiphonaceae	g__Herpetosiphon	s__uncultured_Chloroflexi_bacterium_g__Herpetosiphon	OTU1580
d__Bacteria	k__norank_ d__Bacteria	p__Actinobacteriota	c__Thermoleophilia	o__Solirubrobacterales	f__67-14	g__norank_f__67-14	s__uncultured_actinobacterium_g__norank_f__67-14	OTU2664
d__Bacteria	k__norank_ d__Bacteria	p__Actinobacteriota	c__Actinobacteria	o__Micrococcales	f__Intrasporangiaceae	g__Aquipuribacter	s__uncultured_bacterium_g__Aquipuribacter	OTU2771
d__Bacteria	k__norank_ d__Bacteria	p__Chloroflexi	c__Anaerolineae	o__SBR1031	f__A4b	g__norank_f__A4b	s__wastewater_metagenome_g__norank_f__A4b	OTU339
d__Bacteria	k__norank_ d__Bacteria	p__Actinobacteriota	c__Actinobacteria	o__Pseudonocardiales	f__Pseudonocardiaceae	g__Pseudonocardia	s__Pseudonocardia_sp.	OTU1085
d__Bacteria	k__norank_ d__Bacteria	p__Myxococcota	c__Polyangia	o__Haliangiales	f__Haliangiaceae	g__Haliangium	s__uncultured_bacterium_g__Haliangium	OTU637
d__Bacteria	k__norank_ d__Bacteria	p__Bdellovibrionota	c__Oligoflexia	o__Oligoflexales	f__norank_o__Oligoflexales	g__Oligoflexus	s__metagenome_g__Oligoflexus	OTU2123
d__Bacteria	k__norank_ d__Bacteria	p__Cyanobacteria	c__Cyanobacteriia	o__Pseudanabaenales	f__Pseudanabaenaceae	g__Pseudanabaena_PCC-7429	s__unclassified_g__Pseudanabaena_PCC-7429	OTU132
d__Bacteria	k__norank_ d__Bacteria	p__Proteobacteria	c__Gammaproteobacteria	o__Burkholderiales	f__TRA3-20	g__norank_f__TRA3-20	s__unclassified_g__norank_f__TRA3-20	OTU2137
d__Bacteria	k__norank_ d__Bacteria	p__Chloroflexi	c__Chloroflexia	o__Thermomicrobiales	f__JG30-KF-CM45	g__norank_f__JG30-KF-CM45	s__unclassified_g__norank_f__JG30-KF-CM45	OTU1569
d__Bacteria	k__norank_ d__Bacteria	p__Proteobacteria	c__Gammaproteobacteria	o__Steroidobacterales	f__Steroidobacteraceae	g__Steroidobacter	s__unclassified_g__Steroidobacter	OTU719

d__Bacteria	k__norank_ d__Bacteria	p__Proteobacteria	c__Gammaproteobacteria	o__Burkholderiales	f__Burkholderiaceae	g__Lautropia	s__metagenome_g__Lautropia	OTU486
d__Bacteria	k__norank_ d__Bacteria	p__Proteobacteria	c__Alphaproteobacteria	o__Caulobacterales	f__Caulobacteraceae	g__PMMR1	s__uncultured_bacterium_g__PMMR1	OTU2943
d__Bacteria	k__norank_ d__Bacteria	p__Proteobacteria	c__Alphaproteobacteria	o__Acetobacterales	f__Acetobacteraceae	g__Roseomonas	s__Roseomonas_frigidaquae	OTU2334
d__Bacteria	k__norank_ d__Bacteria	p__Bacteroidota	c__Bacteroidia	o__Sphingobacteriales	f__Sphingobacteriaceae	g__Pedobacter	s__uncultured_bacterium_g__Pedobacter	OTU1366
d__Bacteria	k__norank_ d__Bacteria	p__Proteobacteria	c__Gammaproteobacteria	o__Burkholderiales	f__Nitrosomonadaceae	g__MND1	s__unclassified_g__MND1	OTU2398
d__Bacteria	k__norank_ d__Bacteria	p__Chloroflexi	c__JG30-KF-CM66	o__norank_c__JG30-KF-C M66	f__norank_o__norank_c__JG30-KF-CM 66	g__norank_f__norank_o__norank_c__ JG30-KF-CM66	s__metagenome_g__norank_f__norank_o__norank_c__JG30-KF-CM66	OTU547
d__Bacteria	k__norank_ d__Bacteria	p__Acidobacteriota	c__Blastocatellia	o__Blastocatellales	f__Blastocatellaceae	g__Blastocatella	s__unclassified_g__Blastocatella	OTU129
d__Bacteria	k__norank_ d__Bacteria	p__Acidobacteriota	c__Acidobacteriae	o__Subgroup_2	f__norank_o__Subgroup_2	g__norank_f__norank_o__Subgroup_2	s__unclassified_g__norank_f__norank_o__Subgroup_2	OTU1760
d__Bacteria	k__norank_ d__Bacteria	p__Myxococcota	c__Myxococcia	o__Myxococcales	f__Myxococcaceae	g__norank_f__Myxococcaceae	s__uncultured_bacterium_g__norank_f__Myxococcaceae	OTU654
d__Bacteria	k__norank_ d__Bacteria	p__Actinobacteriota	c__Thermoleophilia	o__Gaiellales	f__norank_o__Gaiellales	g__norank_f__norank_o__Gaiellales	s__unclassified_g__norank_f__norank_o__Gaiellales	OTU1485
d__Bacteria	k__norank_ d__Bacteria	p__Myxococcota	c__Polyangia	o__Polyangiales	f__Sandaracinaceae	g__norank_f__Sandaracinaceae	s__uncultured_soil_bacterium_g__norank_f__Sandaracinaceae	OTU1224
d__Bacteria	k__norank_ d__Bacteria	p__Acidobacteriota	c__Acidobacteriae	o__Bryobacterales	f__Bryobacteraceae	g__Bryobacter	s__unclassified_g__Bryobacter	OTU2336
d__Bacteria	k__norank_ d__Bacteria	p__Bacteroidota	c__Bacteroidia	o__Sphingobacteriales	f__Sphingobacteriaceae	g__Pedobacter	s__unclassified_g__Pedobacter	OTU2266
d__Bacteria	k__norank_ d__Bacteria	p__Bacteroidota	c__Bacteroidia	o__Cytophagales	f__Spirosomaceae	g__Fibrella	s__Fibrella_sp._ES10-3-2-2	OTU141
d__Bacteria	k__norank_ d__Bacteria	p__Actinobacteriota	c__Thermoleophilia	o__Gaiellales	f__norank_o__Gaiellales	g__norank_f__norank_o__Gaiellales	s__metagenome_g__norank_f__norank_o__Gaiellales	OTU904
d__Bacteria	k__norank_ d__Bacteria	p__Proteobacteria	c__Gammaproteobacteria	o__Xanthomonadales	f__Xanthomonadaceae	g__norank_f__Xanthomonadaceae	s__uncultured_gamma_proteobacterium_g__norank_f__Xanthomonadaceae	OTU1667

d__Bacteria	k__norank_d__Bacteria	p__Armatimonadota	c__Armatimonadia	o__Armatimonadales	f__norank_o__Armatimonadales	g__norank_f__norank_o__Armatimonadales	s__uncultured_soil_bacterium_g__norank_f__norank_o__Armatimonadales	OTU2037
d__Bacteria	k__norank_d__Bacteria	p__Actinobacteriota	c__Actinobacteria	o__Micrococcales	f__Micrococcaceae	g__Arthrobacter	s__unclassified_g__Arthrobacter	OTU607
d__Bacteria	k__norank_d__Bacteria	p__SAR324_cladeMarine_group_B	c__norank_p__SAR324_cladeMarine_group_B	o__norank_c__norank_p__SAR324_cladeMarine_group_B	f__norank_o__norank_c__norank_p__SAR324_cladeMarine_group_B	g__norank_f__norank_o__norank_c__norank_p__SAR324_cladeMarine_group_B	s__unclassified_g__norank_f__norank_o__norank_c__norank_p__SAR324_cladeMarine_group_B	OTU1481
d__Bacteria	k__norank_d__Bacteria	p__Myxococcota	c__Polyangia	o__Polyangiales	f__Sandaracinaceae	g__norank_f__Sandaracinaceae	s__metagenome_g__norank_f__Sandaracinaceae	OTU1587
d__Bacteria	k__norank_d__Bacteria	p__Actinobacteriota	c__Actinobacteria	o__Micrococcales	f__Dermatophilaceae	g__unclassified_f__Dermatophilaceae	s__unclassified_f__Dermatophilaceae	OTU2297
d__Bacteria	k__norank_d__Bacteria	p__Bacteroidota	c__Bacteroidia	o__Chitinophagales	f__Chitinophagaceae	g__Chitinophaga	s__metagenome_g__Chitinophaga	OTU231
d__Bacteria	k__norank_d__Bacteria	p__Cyanobacteria	c__Sericytochromatia	o__norank_c__Sericytochromatia	f__norank_o__norank_c__Sericytochromatia	g__norank_f__norank_o__norank_c__Sericytochromatia	s__unclassified_g__norank_f__norank_o__norank_c__Sericytochromatia	OTU686
d__Bacteria	k__norank_d__Bacteria	p__Acidobacteriota	c__Acidobacteriae	o__Subgroup_2	f__norank_o__Subgroup_2	g__norank_f__norank_o__Subgroup_2	s__uncultured_forest_soil_bacterium_g__norank_f__norank_o__Subgroup_2	OTU1653
d__Bacteria	k__norank_d__Bacteria	p__Bacteroidota	c__Bacteroidia	o__Sphingobacteriales	f__unclassified_o__Sphingobacteriales	g__unclassified_o__Sphingobacteriales	s__unclassified_o__Sphingobacteriales	OTU1729
d__Bacteria	k__norank_d__Bacteria	p__Firmicutes	c__Bacilli	o__Entomoplasmatales	f__type_III	g__norank_f__type_III	s__uncultured_Mollicutes_bacterium_g__norank	OTU1530
d__Bacteria	k__norank_d__Bacteria	p__Actinobacteriota	c__Actinobacteria	o__Propionibacteriales	f__Nocardioidaceae	g__Nocardioides	s__unclassified_g__Nocardioides	OTU2486
d__Bacteria	k__norank_d__Bacteria	p__Actinobacteriota	c__Acidimicrobiia	o__Microtrichales	f__Microtrichaceae	g__norank_f__Microtrichaceae	s__metagenome_g__norank_f__Microtrichaceae	OTU1300
d__Bacteria	k__norank_d__Bacteria	p__Myxococcota	c__Polyangia	o__Polyangiales	f__Sandaracinaceae	g__norank_f__Sandaracinaceae	s__unclassified_g__norank_f__Sandaracinaceae	OTU2101
d__Bacteria	k__norank_d__Bacteria	p__Chloroflexi	c__Ktedonobacteria	o__Ktedonobacterales	f__Ktedonobacteraceae	g__HSB_OF53-F07	s__unclassified_g__HSB_OF53-F07	OTU1904
d__Bacteria	k__norank_d__Bacteria	p__Proteobacteria	c__Gammaproteobacteria	o__Burkholderiales	f__SC-I-84	g__norank_f__SC-I-84	s__uncultured_bacterium_g__norank_f__SC-I-84	OTU2793
d__Bacteria	k__norank_d__Bacteria	p__Bacteroidota	c__Bacteroidia	o__Sphingobacteriales	f__AKYH767	g__norank_f__AKYH767	s__uncultured_Bacteroidetes_bacterium_g__norank_f__AKYH767	OTU556

d__Bacteria	k__norank_ d__Bacteria	p__Proteobacteria	c__Alphaproteobacteria	o__Rickettsiales	f__Mitochondria	g__norank_f__Mitochondria	s__unclassified_g__norank_f__Mitochondria	OTU888
d__Bacteria	k__norank_ d__Bacteria	p__Proteobacteria	c__Gammaproteobacteria	o__Burkholderiales	f__Oxalobacteraceae	g__Noviherbaspirillum	s__uncultured_proteobacterium_g__Noviherbaspirillum	OTU255
d__Bacteria	k__norank_ d__Bacteria	p__Proteobacteria	c__Alphaproteobacteria	o__Rhizobiales	f__Xanthobacteraceae	g__Bradyrhizobium	s__unclassified_g__Bradyrhizobium	OTU1968
d__Bacteria	k__norank_ d__Bacteria	p__Myxococcota	c__Polyangia	o__Haliangiales	f__Haliangiaceae	g__Haliangium	s__unclassified_g__Haliangium	OTU1396
d__Bacteria	k__norank_ d__Bacteria	p__Myxococcota	c__Polyangia	o__Haliangiales	f__Haliangiaceae	g__Haliangium	s__uncultured_Myxococcales_bacterium_g__Haliangium	OTU925
d__Bacteria	k__norank_ d__Bacteria	p__Cyanobacteria	c__Cyanobacteriia	o__Chloroplast	f__norank_o__Chloroplast	g__norank_f__norank_o__Chloroplast	s__Klebsormidium_nitens	OTU75
d__Bacteria	k__norank_ d__Bacteria	p__Bacteroidota	c__Bacteroidia	o__Flavobacteriales	f__Weeksellaceae	g__Chryseobacterium	s__unclassified_g__Chryseobacterium	OTU1157
d__Bacteria	k__norank_ d__Bacteria	p__Proteobacteria	c__Alphaproteobacteria	o__norank_c__Alphaproteo bacteria	f__norank_o__norank_c__Alphaproteoba cteria	g__norank_f__norank_o__norank_c__ Alphaproteobacteria	s__unclassified_g__norank_f__norank_o__norank_c__Alphaproteobacteria	OTU1800
d__Bacteria	k__norank_ d__Bacteria	p__Proteobacteria	c__Gammaproteobacteria	o__Burkholderiales	f__Comamonadaceae	g__Rhizobacter	s__uncultured_bacterium_g__Rhizobacter	OTU102
d__Bacteria	k__norank_ d__Bacteria	p__Bacteroidota	c__Bacteroidia	o__Flavobacteriales	f__Flavobacteriaceae	g__Flavobacterium	s__Antarctic_bacterium_R-7515	OTU229
d__Bacteria	k__norank_ d__Bacteria	p__Chloroflexi	c__Anaerolineae	o__Caldilineales	f__Caldilineaceae	g__norank_f__Caldilineaceae	s__unclassified_g__norank_f__Caldilineaceae	OTU2310
d__Bacteria	k__norank_ d__Bacteria	p__Bacteroidota	c__Rhodothermia	o__Rhodothermales	f__Rhodothermaceae	g__norank_f__Rhodothermaceae	s__unclassified_g__norank_f__Rhodothermaceae	OTU643
d__Bacteria	k__norank_ d__Bacteria	p__Proteobacteria	c__Alphaproteobacteria	o__Azospirillales	f__Azospirillaceae	g__Skermanella	s__uncultured_bacterium_g__Skermanella	OTU2780
d__Bacteria	k__norank_ d__Bacteria	p__Proteobacteria	c__Gammaproteobacteria	o__Burkholderiales	f__A21b	g__norank_f__A21b	s__uncultured_bacterium_g__norank_f__A21b	OTU873
d__Bacteria	k__norank_ d__Bacteria	p__Chloroflexi	c__Ktedonobacteria	o__B12-WMSP1	f__norank_o__B12-WMSP1	g__norank_f__norank_o__B12-WMSP 1	s__unclassified_g__norank_f__norank_o__B12-WMSP1	OTU1871
d__Bacteria	k__norank_ d__Bacteria	p__Proteobacteria	c__Alphaproteobacteria	o__Rhodobacterales	f__Rhodobacteraceae	g__Amaricoccus	s__metagenome_g__Amaricoccus	OTU2865

d__Bacteria	k__norank_ d__Bacteria	p__Firmicutes	c__Bacilli	o__Erysipelotrichales	f__Erysipelotrichaceae	g__ZOR0006	s__uncultured_bacterium_g__ZOR0006	OTU59
d__Bacteria	k__norank_ d__Bacteria	p__Patescibacteria	c__Saccharimonadia	o__Saccharimonadales	f__LWQ8	g__norank_f__LWQ8	s__uncultured_Candidatus_Saccharibacteria_bacterium_g__norank_f__LWQ8	OTU32
d__Bacteria	k__norank_ d__Bacteria	p__Proteobacteria	c__Gammaproteobacteria	o__211ds20	f__norank_o__211ds20	g__norank_f__norank_o__211ds20	s__uncultured_bacterium_g__norank_f__norank_o__211ds20	OTU2246
d__Bacteria	k__norank_ d__Bacteria	p__Actinobacteriota	c__Acidimicrobiia	o__unclassified_c__Acidimi crobiia	f__unclassified_c__Acidimicrobiia	g__unclassified_c__Acidimicrobiia	s__unclassified_c__Acidimicrobiia	OTU2208
d__Bacteria	k__norank_ d__Bacteria	p__Proteobacteria	c__Alphaproteobacteria	o__Micavibrionales	f__norank_o__Micavibrionales	g__norank_f__norank_o__Micavibrion ales	s__uncultured_bacterium_g__norank_f__norank_o__Micavibrionales	OTU518
d__Bacteria	k__norank_ d__Bacteria	p__Acidobacteriota	c__Holophagae	o__Subgroup_7	f__norank_o__Subgroup_7	g__norank_f__norank_o__Subgroup_7	s__unclassified_g__norank_f__norank_o__Subgroup_7	OTU1873
d__Bacteria	k__norank_ d__Bacteria	p__Bacteroidota	c__Bacteroidia	o__Flavobacteriales	f__Flavobacteriaceae	g__Flavobacterium	s__Flavobacterium_urumqiense	OTU594
d__Bacteria	k__norank_ d__Bacteria	p__Actinobacteriota	c__Thermoleophilia	o__unclassified_c__Thermol eophilia	f__unclassified_c__Thermoleophilia	g__unclassified_c__Thermoleophilia	s__unclassified_c__Thermoleophilia	OTU1370
d__Bacteria	k__norank_ d__Bacteria	p__Acidobacteriota	c__Vicinamibacteria	o__Subgroup_17	f__norank_o__Subgroup_17	g__norank_f__norank_o__Subgroup_1 7	s__uncultured_bacterium_g__norank_f__norank_o__Subgroup_17	OTU2613
d__Bacteria	k__norank_ d__Bacteria	p__Bacteroidota	c__Rhodothermia	o__Rhodothermales	f__Rhodothermaceae	g__norank_f__Rhodothermaceae	s__unclassified_g__norank_f__Rhodothermaceae	OTU2128
d__Bacteria	k__norank_ d__Bacteria	p__Deinococcota	c__Deinococci	o__Deinococcales	f__Deinococcaceae	g__Deinococcus	s__uncultured_bacterium_g__Deinococcus	OTU103
d__Bacteria	k__norank_ d__Bacteria	p__Actinobacteriota	c__Actinobacteria	o__Frankiales	f__Cryptosporangiaceae	g__Cryptosporangium	s__Cryptosporangium_japonicum	OTU2203
d__Bacteria	k__norank_ d__Bacteria	p__Planctomycetota	c__Planctomycetes	o__Gemmatales	f__Gemmataceae	g__norank_f__Gemmataceae	s__unclassified_g__norank_f__Gemmataceae	OTU1136
d__Bacteria	k__norank_ d__Bacteria	p__Armatimonadota	c__Chthonomonadetes	o__Chthonomonadales	f__Chthonomonadaceae	g__Chthonomonas	s__uncultured_bacterium_g__Chthonomonas	OTU2067
d__Bacteria	k__norank_ d__Bacteria	p__Gemmatimonadota	c__Gemmatimonadetes	o__Gemmatimonadales	f__Gemmatimonadaceae	g__Gemmatimonas	s__uncultured_soil_bacterium_g__Gemmatimonas	OTU2532
d__Bacteria	k__norank_ d__Bacteria	p__Actinobacteriota	c__Thermoleophilia	o__Gaiellales	f__unclassified_o__Gaiellales	g__unclassified_o__Gaiellales	s__unclassified_o__Gaiellales	OTU1250

d__Bacteria	k__norank_ d__Bacteria	p__Acidobacteriota	c__Acidobacteriae	o__Solibacterales	f__Solibacteraceae	g__Candidatus_Solibacter	s__uncultured_Acidobacteria_bacterium_g__Candidatus_Solibacter	OTU880
d__Bacteria	k__norank_ d__Bacteria	p__Elusimicrobiota	c__Lineage_IIa	o__norank_c__Lineage_IIa	f__norank_o__norank_c__Lineage_IIa	g__norank_f__norank_o__norank_c__ Lineage_IIa	s__uncultured_bacterium_g__norank_f__norank_o__norank_c__Lineage_IIa	OTU1018
d__Bacteria	k__norank_ d__Bacteria	p__Myxococcota	c__Polyangia	o__Blfdi19	f__norank_o__Blfdi19	g__norank_f__norank_o__Blfdi19	s__unclassified_g__norank_f__norank_o__Blfdi19	OTU2636
d__Bacteria	k__norank_ d__Bacteria	p__Chloroflexi	c__Anaerolineae	o__SBR1031	f__norank_o__SBR1031	g__norank_f__norank_o__SBR1031	s__wastewater_metagenome_g__norank_f__norank_o__SBR1031	OTU528
d__Bacteria	k__norank_ d__Bacteria	p__Bacteroidota	c__Bacteroidia	o__Cytophagales	f__Cytophagaceae	g__Rhodocytophaga	s__unclassified_g__Rhodocytophaga	OTU2915
d__Bacteria	k__norank_ d__Bacteria	p__Acidobacteriota	c__Holophagae	o__Subgroup_7	f__norank_o__Subgroup_7	g__norank_f__norank_o__Subgroup_7	s__unclassified_g__norank_f__norank_o__Subgroup_7	OTU2418
d__Bacteria	k__norank_ d__Bacteria	p__Bacteroidota	c__Bacteroidia	o__Flavobacteriales	f__Weeksellaceae	g__Chryseobacterium	s__unclassified_g__Chryseobacterium	OTU2708
d__Bacteria	k__norank_ d__Bacteria	p__Acidobacteriota	c__Acidobacteriae	o__Bryobacterales	f__Bryobacteraceae	g__Bryobacter	s__uncultured_bacterium_g__Bryobacter	OTU2558
d__Bacteria	k__norank_ d__Bacteria	p__Bdellovibrionota	c__Oligoflexia	o__0319-6G20	f__norank_o__0319-6G20	g__norank_f__norank_o__0319-6G20	s__unclassified_g__norank_f__norank_o__0319-6G20	OTU2676
d__Bacteria	k__norank_ d__Bacteria	p__Bdellovibrionota	c__Bdellovibrionia	o__Bacteriovoracales	f__Bacteriovoracaceae	g__Bacteriovorax	s__uncultured_bacterium_g__Bacteriovorax	OTU2672
d__Bacteria	k__norank_ d__Bacteria	p__Bacteroidota	c__Bacteroidia	o__Chitinophagales	f__Saprospiraceae	g__norank_f__Saprospiraceae	s__metagenome_g__norank_f__Saprospiraceae	OTU596
d__Bacteria	k__norank_ d__Bacteria	p__Actinobacteriota	c__Acidimicrobiia	o__Microtrichales	f__norank_o__Microtrichales	g__norank_f__norank_o__Microtrichal es	s__uncultured_actinobacterium_g__norank_f__norank_o__Microtrichales	OTU2224
d__Bacteria	k__norank_ d__Bacteria	p__Firmicutes	c__Bacilli	o__Paenibacillales	f__Paenibacillaceae	g__Paenibacillus	s__Paenibacillus_odorifer	OTU663
d__Bacteria	k__norank_ d__Bacteria	p__Proteobacteria	c__Alphaproteobacteria	o__norank_c__Alphaproteo bacteria	f__norank_o__norank_c__Alphaproteoba cteria	g__norank_f__norank_o__norank_c__ Alphaproteobacteria	s__bacterium_Ellin6089	OTU1876
d__Bacteria	k__norank_ d__Bacteria	p__Proteobacteria	c__Alphaproteobacteria	o__Sphingomonadales	f__Sphingomonadaceae	g__Sphingomonas	s__metagenome_g__Sphingomonas	OTU239
d__Bacteria	k__norank_ d__Bacteria	p__Myxococcota	c__Polyangia	o__Polyangiales	f__Sandaracinaceae	g__Sandaracinus	s__uncultured_bacterium_g__Sandaracinus	OTU2562

d__Bacteria	k__norank_ d__Bacteria	p__Chloroflexi	c__Anaerolineae	o__SBR1031	f__A4b	g__norank_f__A4b	s__metagenome_g__norank_f__A4b	OTU2414
d__Bacteria	k__norank_ d__Bacteria	p__Chloroflexi	c__Ktedonobacteria	o__C0119	f__norank_o__C0119	g__norank_f__norank_o__C0119	s__uncultured_bacterium_g__norank_f__norank_o__C0119	OTU1666
d__Bacteria	k__norank_ d__Bacteria	p__Chloroflexi	c__Chloroflexia	o__Chloroflexales	f__Chloroflexaceae	g__Chloronema	s__unclassified_g__Chloronema	OTU2619
d__Bacteria	k__norank_ d__Bacteria	p__Bdellovibrionota	c__Bdellovibrionia	o__Bdellovibrionales	f__Bdellovibrionaceae	g__Bdellovibrio	s__unclassified_g__Bdellovibrio	OTU347
d__Bacteria	k__norank_ d__Bacteria	p__Planctomycetota	c__Planctomycetes	o__Gemmatales	f__Gemmataceae	g__norank_f__Gemmataceae	s__uncultured_bacterium_g__norank_f__Gemmataceae	OTU847
d__Bacteria	k__norank_ d__Bacteria	p__Bacteroidota	c__Bacteroidia	o__Chitinophagales	f__Chitinophagaceae	g__Terrimonas	s__uncultured_Chitinophagaceae_bacterium_g__Terrimonas	OTU1520
d__Bacteria	k__norank_ d__Bacteria	p__Bacteroidota	c__Bacteroidia	o__Flavobacteriales	f__Flavobacteriaceae	g__Flavobacterium	s__unclassified_g__Flavobacterium	OTU1840
d__Bacteria	k__norank_ d__Bacteria	p__Proteobacteria	c__Gammaproteobacteria	o__Burkholderiales	f__Burkholderiaceae	g__Mycoavidus	s__unclassified_g__Mycoavidus	OTU2125
d__Bacteria	k__norank_ d__Bacteria	p__Planctomycetota	c__Planctomycetes	o__Gemmatales	f__Gemmataceae	g__norank_f__Gemmataceae	s__unclassified_g__norank_f__Gemmataceae	OTU1354
d__Bacteria	k__norank_ d__Bacteria	p__Proteobacteria	c__Gammaproteobacteria	o__Burkholderiales	f__Comamonadaceae	g__Caenimonas	s__uncultured_bacterium_g__Caenimonas	OTU2228
d__Bacteria	k__norank_ d__Bacteria	p__Myxococcota	c__Myxococcia	o__Myxococcales	f__Anaeromyxobacteraceae	g__Anaeromyxobacter	s__uncultured_bacterium_g__Anaeromyxobacter	OTU2029
d__Bacteria	k__norank_ d__Bacteria	p__Chloroflexi	c__Chloroflexia	o__Thermomicrobiales	f__JG30-KF-CM45	g__norank_f__JG30-KF-CM45	s__uncultured_bacterium_g__norank_f__JG30-KF-CM45	OTU1348
d__Bacteria	k__norank_ d__Bacteria	p__Bdellovibrionota	c__Oligoflexia	o__0319-6G20	f__norank_o__0319-6G20	g__norank_f__norank_o__0319-6G20	s__unclassified_g__norank_f__norank_o__0319-6G20	OTU1896
d__Bacteria	k__norank_ d__Bacteria	p__Proteobacteria	c__Gammaproteobacteria	o__Burkholderiales	f__Sutterellaceae	g__norank_f__Sutterellaceae	s__unclassified_g__norank_f__Sutterellaceae	OTU2131
d__Bacteria	k__norank_ d__Bacteria	p__Cyanobacteria	c__Cyanobacteriia	o__Chloroplast	f__norank_o__Chloroplast	g__norank_f__norank_o__Chloroplast	s__unclassified_g__norank_f__norank_o__Chloroplast	OTU692
d__Bacteria	k__norank_ d__Bacteria	p__Actinobacteriota	c__Thermoleophilia	o__Solirubrobacterales	f__unclassified_o__Solirubrobacterales	g__unclassified_o__Solirubrobacterale s	s__unclassified_o__Solirubrobacterales	OTU2367

d__Bacteria	k__norank_d__Bacteria	p__Chloroflexi	c__Anaerolineae	o__SBR1031	f__A4b	g__norank_f__A4b	s__uncultured_soil_bacterium_g__norank_f__A4b	OTU2448
d__Bacteria	k__norank_d__Bacteria	p__Cyanobacteria	c__Cyanobacteriia	o__Chloroplast	f__norank_o__Chloroplast	g__norank_f__norank_o__Chloroplast	s__unclassified_g__norank_f__norank_o__Chloroplast	OTU1
d__Bacteria	k__norank_d__Bacteria	p__Armatimonadota	c__Chthonomonadetes	o__Chthonomonadales	f__Chthonomonadaceae	g__Chthonomonas	s__unclassified_g__Chthonomonas	OTU1927
d__Bacteria	k__norank_d__Bacteria	p__Chloroflexi	c__unclassified_p__Chloroflexi	o__unclassified_p__Chloroflexi	f__unclassified_p__Chloroflexi	g__unclassified_p__Chloroflexi	s__unclassified_p__Chloroflexi	OTU2494
d__Bacteria	k__norank_d__Bacteria	p__Actinobacteriota	c__Actinobacteria	o__Frankiales	f__Sporichthyaceae	g__norank_f__Sporichthyaceae	s__uncultured_bacterium_g__norank_f__Sporichthyaceae	OTU2878
d__Bacteria	k__norank_d__Bacteria	p__Dependentiae	c__Babeliae	o__Babeliales	f__Vermiphilaceae	g__norank_f__Vermiphilaceae	s__uncultured_bacterium_g__norank_f__Vermiphilaceae	OTU2792
d__Bacteria	k__norank_d__Bacteria	p__Bacteroidota	c__Bacteroidia	o__Cytophagales	f__Cytophagaceae	g__Siphonobacter	s__uncultured_bacterium_g__Siphonobacter	OTU172
d__Bacteria	k__norank_d__Bacteria	p__Actinobacteriota	c__Actinobacteria	o__Frankiales	f__Acidothermaceae	g__Acidothermus	s__unclassified_g__Acidothermus	OTU1795
d__Bacteria	k__norank_d__Bacteria	p__WPS-2	c__norank_p__WPS-2	o__norank_c__norank_p__WPS-2	f__norank_o__norank_c__norank_p__WPS-2	g__norank_f__norank_o__norank_c__norank_p__WPS-2	s__uncultured_Firmicutes_bacterium_g__norank_f__norank_o__norank_c__norank_p__WPS-2	OTU1935
d__Bacteria	k__norank_d__Bacteria	p__Dependentiae	c__Babeliae	o__Babeliales	f__Vermiphilaceae	g__norank_f__Vermiphilaceae	s__uncultured_bacterium_g__norank_f__Vermiphilaceae	OTU2799
d__Bacteria	k__norank_d__Bacteria	p__Bacteroidota	c__Bacteroidia	o__Cytophagales	f__Hymenobacteraceae	g__Hymenobacter	s__unclassified_g__Hymenobacter	OTU203
d__Bacteria	k__norank_d__Bacteria	p__Actinobacteriota	c__Thermoleophilia	o__Gaiellales	f__Gaiellaceae	g__Gaiella	s__uncultured_actinobacterium_g__Gaiella	OTU449
d__Bacteria	k__norank_d__Bacteria	p__Proteobacteria	c__Alphaproteobacteria	o__Acetobacterales	f__Acetobacteraceae	g__Acidiphilium	s__uncultured_bacterium_g__Acidiphilium	OTU1196
d__Bacteria	k__norank_d__Bacteria	p__Verrucomicrobiota	c__Verrucomicrobiae	o__Chthoniobacterales	f__Chthoniobacteraceae	g__Candidatus_Udaeobacter	s__uncultured_Verrucomicrobia_bacterium_g__Candidatus_Udaeobacter	OTU779
d__Bacteria	k__norank_d__Bacteria	p__Abditibacteriota	c__Abditibacteria	o__Abditibacteriales	f__Abditibacteriaceae	g__Abditibacterium	s__unclassified_g__Abditibacterium	OTU2824
d__Bacteria	k__norank_d__Bacteria	p__Armatimonadota	c__norank_p__Armatimonadota	o__norank_c__norank_p__Armatimonadota	f__norank_o__norank_c__norank_p__Armatimonadota	g__norank_f__norank_o__norank_c__norank_p__Armatimonadota	s__unclassified_g__norank_f__norank_o__norank_c__norank_p__Armatimonadota	OTU896

d__Bacteria	k__norank_ d__Bacteria	p__Bacteroidota	c__Bacteroidia	o__Cytophagales	f__Spirosomaceae	g__Spirosoma	s__unclassified_g__Spirosoma	OTU355
d__Bacteria	k__norank_ d__Bacteria	p__Chloroflexi	c__Ktedonobacteria	o__C0119	f__norank_o__C0119	g__norank_f__norank_o__C0119	s__uncultured_bacterium_g__norank_f__norank_o__C0119	OTU993
d__Bacteria	k__norank_ d__Bacteria	p__Actinobacteriota	c__Acidimicrobiia	o__Microtrichales	f__norank_o__Microtrichales	g__norank_f__norank_o__Microtrichal es	s__metagenome_g__norank_f__norank_o__Microtrichales	OTU159
d__Bacteria	k__norank_ d__Bacteria	p__Proteobacteria	c__Gammaproteobacteria	o__Xanthomonadales	f__Xanthomonadaceae	g__unclassified_f__Xanthomonadacea e	s__unclassified_f__Xanthomonadaceae	OTU249
d__Bacteria	k__norank_ d__Bacteria	p__Actinobacteriota	c__Actinobacteria	o__Micrococcales	f__Microbacteriaceae	g__unclassified_f__Microbacteriaceae	s__unclassified_f__Microbacteriaceae	OTU2929
d__Bacteria	k__norank_ d__Bacteria	p__Planctomycetota	c__Planctomycetes	o__Gemmatales	f__Gemmataceae	g__norank_f__Gemmataceae	s__uncultured_bacterium_g__norank_f__Gemmataceae	OTU1632
d__Bacteria	k__norank_ d__Bacteria	p__Acidobacteriota	c__Acidobacteriae	o__Subgroup_2	f__norank_o__Subgroup_2	g__norank_f__norank_o__Subgroup_2	s__uncultured_bacterium_g__norank_f__norank_o__Subgroup_2	OTU1738
d__Bacteria	k__norank_ d__Bacteria	p__Proteobacteria	c__Gammaproteobacteria	o__Burkholderiales	f__Hydrogenophilaceae	g__Thiobacillus	s__unclassified_g__Thiobacillus	OTU630
d__Bacteria	k__norank_ d__Bacteria	p__Myxococcota	c__Myxococcia	o__Myxococcales	f__Myxococcaceae	g__unclassified_f__Myxococcaceae	s__unclassified_f__Myxococcaceae	OTU978
d__Bacteria	k__norank_ d__Bacteria	p__Proteobacteria	c__Gammaproteobacteria	o__Xanthomonadales	f__Rhodanobacteraceae	g__Rhodanobacter	s__uncultured_Dokdonella_sp._g__Rhodanobacter	OTU900
d__Bacteria	k__norank_ d__Bacteria	p__Firmicutes	c__Bacilli	o__Entomoplasmatales	f__unclassified_o__Entomoplasmatales	g__unclassified_o__Entomoplasmatale s	s__unclassified_o__Entomoplasmatales	OTU1251
d__Bacteria	k__norank_ d__Bacteria	p__Bacteroidota	c__Bacteroidia	o__Sphingobacteriales	f__Sphingobacteriaceae	g__unclassified_f__Sphingobacteriacea e	s__unclassified_f__Sphingobacteriaceae	OTU2838
d__Bacteria	k__norank_ d__Bacteria	p__SAR324_cladeMar ine_group_B	c__norank_p__SAR324_c ladeMarine_group_B	o__norank_c__norank_p__S AR324_cladeMarine_group _B	f__norank_o__norank_c__norank_p__SA R324_cladeMarine_group_B	g__norank_f__norank_o__norank_c__ norank_p__SAR324_cladeMarine_gro up_B	s__unclassified_g__norank_f__norank_o__norank_c__norank_p__SAR324_cladeM arine_group_B	OTU161
d__Bacteria	k__norank_ d__Bacteria	p__Proteobacteria	c__Alphaproteobacteria	o__Rhizobiales	f__Kaistiaceae	g__Kaistia	s__uncultured_bacterium_g__Kaistia	OTU2912
d__Bacteria	k__norank_ d__Bacteria	p__Proteobacteria	c__Alphaproteobacteria	o__norank_c__Alphaproteo bacteria	f__norank_o__norank_c__Alphaproteoba cteria	g__norank_f__norank_o__norank_c__ Alphaproteobacteria	s__uncultured_Bradyrhizobiaceae_bacterium_g__norank	OTU1316
d__Bacteria	k__norank_ d__Bacteria	p__Proteobacteria	c__Gammaproteobacteria	o__Gammaproteobacteria_I ncertae_Sedis	f__unclassified_o__Gammaproteobacteri a_Incertae_Sedis	g__Candidatus_Ovatusbacter	s__uncultured_bacterium_g__Candidatus_Ovatusbacter	OTU1091

d__Bacteria	k__norank_ d__Bacteria	p__Proteobacteria	c__Alphaproteobacteria	o__Rhodospirillales	f__norank_o__Rhodospirillales	g__norank_f__norank_o__Rhodospirill ales	s__unclassified_g__norank_f__norank_o__Rhodospirillales	OTU319
d__Bacteria	k__norank_ d__Bacteria	p__Proteobacteria	c__Gammaproteobacteria	o__Burkholderiales	f__SC-I-84	g__norank_f__SC-I-84	s__unclassified_g__norank_f__SC-I-84	OTU1435
d__Bacteria	k__norank_ d__Bacteria	p__Proteobacteria	c__Gammaproteobacteria	o__Burkholderiales	f__Nitrosomonadaceae	g__Ellin6067	s__unclassified_g__Ellin6067	OTU2478
d__Bacteria	k__norank_ d__Bacteria	p__Bacteroidota	c__Rhodothermia	o__Rhodothermales	f__Rhodothermaceae	g__Rubrivirga	s__uncultured_bacterium_g__Rubrivirga	OTU634
d__Bacteria	k__norank_ d__Bacteria	p__Bdellovibrionota	c__Oligoflexia	o__0319-6G20	f__norank_o__0319-6G20	g__norank_f__norank_o__0319-6G20	s__marine_metagenome_g__norank_f__norank_o__0319-6G20	OTU1060
d__Bacteria	k__norank_ d__Bacteria	p__Elusimicrobiota	c__Elusimicrobia	o__Lineage_IV	f__norank_o__Lineage_IV	g__norank_f__norank_o__Lineage_IV	s__uncultured_Termite_group_1_bacterium_g__norank_f__norank_o__Lineage_IV	OTU469
d__Bacteria	k__norank_ d__Bacteria	p__Patescibacteria	c__Saccharimonadia	o__Saccharimonadales	f__LWQ8	g__norank_f__LWQ8	s__uncultured_bacterium_g__norank_f__LWQ8	OTU1469
d__Bacteria	k__norank_ d__Bacteria	p__Bacteroidota	c__Bacteroidia	o__Cytophagales	f__Spirosomaceae	g__Runella	s__Runella_slithyformis_DSM_19594	OTU230
d__Bacteria	k__norank_ d__Bacteria	p__Actinobacteriota	c__Thermoleophilia	o__Solirubrobacterales	f__67-14	g__norank_f__67-14	s__uncultured_bacterium_g__norank_f__67-14	OTU2796
d__Bacteria	k__norank_ d__Bacteria	p__Chloroflexi	c__AD3	o__norank_c__AD3	f__norank_o__norank_c__AD3	g__norank_f__norank_o__norank_c__ AD3	s__unclassified_g__norank_f__norank_o__norank_c__AD3	OTU1775
d__Bacteria	k__norank_ d__Bacteria	p__Bdellovibrionota	c__Bdellovibrionia	o__Bdellovibrionales	f__Bdellovibrionaceae	g__Bdellovibrio	s__unclassified_g__Bdellovibrio	OTU2368
d__Bacteria	k__norank_ d__Bacteria	p__Bacteroidota	c__Bacteroidia	o__Cytophagales	f__Hymenobacteraceae	g__Hymenobacter	s__unclassified_g__Hymenobacter	OTU2415
d__Bacteria	k__norank_ d__Bacteria	p__Acidobacteriota	c__Thermoanaerobaculia	o__Thermoanaerobaculales	f__Thermoanaerobaculaceae	g__Subgroup_10	s__metagenome_g__Subgroup_10	OTU2094
d__Bacteria	k__norank_ d__Bacteria	p__Deinococcota	c__Deinococci	o__Deinococcales	f__Deinococcaceae	g__Deinococcus	s__unclassified_g__Deinococcus	OTU97
d__Bacteria	k__norank_ d__Bacteria	p__Proteobacteria	c__Alphaproteobacteria	o__Elsterales	f__norank_o__Elsterales	g__norank_f__norank_o__Elsterales	s__uncultured_bacterium_g__norank_f__norank_o__Elsterales	OTU2593
d__Bacteria	k__norank_ d__Bacteria	p__Chloroflexi	c__Ktedonobacteria	o__C0119	f__norank_o__C0119	g__norank_f__norank_o__C0119	s__uncultured_bacterium_g__norank_f__norank_o__C0119	OTU1919

d__Bacteria	k__norank_ d__Bacteria	p__Gemmatimonadota	c__Gemmatimonadetes	o__Gemmatimonadales	f__Gemmatimonadaceae	g__Gemmatimonas	s__unclassified_g__Gemmatimonas	OTU1041
d__Bacteria	k__norank_ d__Bacteria	p__Bacteroidota	c__Bacteroidia	o__Sphingobacteriales	f__env.OPS_17	g__norank_f__env.OPS_17	s__unclassified_g__norank_f__env.OPS_17	OTU2354
d__Bacteria	k__norank_ d__Bacteria	p__Bdellovibrionota	c__Oligoflexia	o__0319-6G20	f__norank_o__0319-6G20	g__norank_f__norank_o__0319-6G20	s__unclassified_g__norank_f__norank_o__0319-6G20	OTU804
d__Bacteria	k__norank_ d__Bacteria	p__Verrucomicrobiota	c__Chlamydiae	o__Chlamydiales	f__unclassified_o__Chlamydiales	g__unclassified_o__Chlamydiales	s__unclassified_o__Chlamydiales	OTU534
d__Bacteria	k__norank_ d__Bacteria	p__Verrucomicrobiota	c__Verrucomicrobiae	o__Pedosphaerales	f__Pedosphaeraceae	g__ADurb.Bin063-1	s__uncultured_bacterium_g__ADurb.Bin063-1	OTU1765
d__Bacteria	k__norank_ d__Bacteria	p__Bacteroidota	c__Bacteroidia	o__Chitinophagales	f__Chitinophagaceae	g__Segetibacter	s__uncultured_bacterium_g__Segetibacter	OTU1140
d__Bacteria	k__norank_ d__Bacteria	p__Bacteroidota	c__Bacteroidia	o__Chitinophagales	f__Chitinophagaceae	g__Segetibacter	s__uncultured_bacterium_g__Segetibacter	OTU1630
d__Bacteria	k__norank_ d__Bacteria	p__Verrucomicrobiota	c__Verrucomicrobiae	o__Chthoniobacterales	f__Chthoniobacteraceae	g__Chthoniobacter	s__unclassified_g__Chthoniobacter	OTU2924
d__Bacteria	k__norank_ d__Bacteria	p__Proteobacteria	c__Gammaproteobacteria	o__Pseudomonadales	f__Moraxellaceae	g__norank_f__Moraxellaceae	s__uncultured_gamma_proteobacterium_g__norank_f__Moraxellaceae	OTU1996
d__Bacteria	k__norank_ d__Bacteria	p__Actinobacteriota	c__Actinobacteria	o__Micrococcales	f__Micrococcaceae	g__Paenarthrobacter	s__unclassified_g__Paenarthrobacter	OTU1213
d__Bacteria	k__norank_ d__Bacteria	p__Acidobacteriota	c__Acidobacteriae	o__Bryobacterales	f__Bryobacteraceae	g__Bryobacter	s__unclassified_g__Bryobacter	OTU1989
d__Bacteria	k__norank_ d__Bacteria	p__Myxococcota	c__Polyangia	o__Haliangiales	f__Haliangiaceae	g__Haliangium	s__uncultured_bacterium_g__Haliangium	OTU2432
d__Bacteria	k__norank_ d__Bacteria	p__Acidobacteriota	c__Vicinamibacteria	o__Vicinamibacterales	f__norank_o__Vicinamibacterales	g__norank_f__norank_o__Vicinamiba cterales	s__uncultured_Acidobacteriaceae_bacterium_g__norank_f__norank_o__Vicinamiba cterales	OTU452
d__Bacteria	k__norank_ d__Bacteria	p__Bacteroidota	c__Bacteroidia	o__Sphingobacteriales	f__Sphingobacteriaceae	g__unclassified_f__Sphingobacteriacea e	s__unclassified_f__Sphingobacteriaceae	OTU505
d__Bacteria	k__norank_ d__Bacteria	p__Bacteroidota	c__Bacteroidia	o__Cytophagales	f__Spirosomaceae	g__Rudanella	s__unclassified_g__Rudanella	OTU174
d__Bacteria	k__norank_ d__Bacteria	p__Myxococcota	c__Polyangia	o__Haliangiales	f__Haliangiaceae	g__Haliangium	s__unclassified_g__Haliangium	OTU2405

d__Bacteria	k__norank_d__Bacteria	p__Cyanobacteria	c__Sericytochromatia	o__norank_c__Sericytochromatia	f__norank_o__norank_c__Sericytochromatia	g__norank_f__norank_o__norank_c__Sericytochromatia	s__unclassified_g__norank_f__norank_o__norank_c__Sericytochromatia	OTU1966
d__Bacteria	k__norank_d__Bacteria	p__Myxococcota	c__Polyangia	o__Polyangiales	f__Sandaracinaceae	g__norank_f__Sandaracinaceae	s__metagenome_g__norank_f__Sandaracinaceae	OTU1334
d__Bacteria	k__norank_d__Bacteria	p__Deinococcota	c__Deinococci	o__Deinococcales	f__Deinococcaceae	g__Deinococcus	s__Deinococcus_sp.	OTU206
d__Bacteria	k__norank_d__Bacteria	p__Proteobacteria	c__Alphaproteobacteria	o__Acetobacterales	f__Acetobacteraceae	g__Roseomonas	s__Roseomonas_arctica	OTU415
d__Bacteria	k__norank_d__Bacteria	p__Patescibacteria	c__Parcubacteria	o__Candidatus_Kaiserbacteria	f__norank_o__Candidatus_Kaiserbacteria	g__norank_f__norank_o__Candidatus_Kaiserbacteria	s__unclassified_g__norank_f__norank_o__Candidatus_Kaiserbacteria	OTU511
d__Bacteria	k__norank_d__Bacteria	p__Patescibacteria	c__Saccharimonadia	o__Saccharimonadales	f__LWQ8	g__norank_f__LWQ8	s__uncultured_bacterium_g__norank_f__LWQ8	OTU1762
d__Bacteria	k__norank_d__Bacteria	p__Bacteroidota	c__Bacteroidia	o__Chitinophagales	f__Chitinophagaceae	g__Segetibacter	s__uncultured_bacterium_g__Segetibacter	OTU809
d__Bacteria	k__norank_d__Bacteria	p__Firmicutes	c__Bacilli	o__Paenibacillales	f__Paenibacillaceae	g__Paenibacillus	s__unclassified_g__Paenibacillus	OTU2440
d__Bacteria	k__norank_d__Bacteria	p__Bdellovibrionota	c__Oligoflexia	o__0319-6G20	f__norank_o__0319-6G20	g__norank_f__norank_o__0319-6G20	s__unclassified_g__norank_f__norank_o__0319-6G20	OTU2015
d__Bacteria	k__norank_d__Bacteria	p__Acidobacteriota	c__Acidobacteriae	o__Acidobacteriales	f__norank_o__Acidobacteriales	g__norank_f__norank_o__Acidobacteriales	s__unclassified_g__norank_f__norank_o__Acidobacteriales	OTU1075
d__Bacteria	k__norank_d__Bacteria	p__Chloroflexi	c__Ktedonobacteria	o__C0119	f__norank_o__C0119	g__norank_f__norank_o__C0119	s__uncultured_bacterium_g__norank_f__norank_o__C0119	OTU1052
d__Bacteria	k__norank_d__Bacteria	p__Planctomycetota	c__Planctomycetes	o__Isosphaerales	f__Isosphaeraceae	g__Aquisphaera	s__uncultured_bacterium_g__Aquisphaera	OTU1993
d__Bacteria	k__norank_d__Bacteria	p__Proteobacteria	c__Alphaproteobacteria	o__Rhizobiales	f__Beijerinckiaceae	g__Bosea	s__uncultured_bacterium_g__Bosea	OTU374
d__Bacteria	k__norank_d__Bacteria	p__unclassified_k__norank_d__Bacteria	c__unclassified_k__norank_d__Bacteria	o__unclassified_k__norank_d__Bacteria	f__unclassified_k__norank_d__Bacteria	g__unclassified_k__norank_d__Bacteria	s__unclassified_k__norank_d__Bacteria	OTU2130
d__Bacteria	k__norank_d__Bacteria	p__Proteobacteria	c__Alphaproteobacteria	o__Acetobacterales	f__Acetobacteraceae	g__unclassified_f__Acetobacteraceae	s__unclassified_f__Acetobacteraceae	OTU1988
d__Bacteria	k__norank_d__Bacteria	p__Bacteroidota	c__Bacteroidia	o__Chitinophagales	f__Chitinophagaceae	g__Flaviaesturariibacter	s__unclassified_g__Flaviaesturariibacter	OTU176

d__Bacteria	k__norank_d__Bacteria	p__Bacteroidota	c__Bacteroidia	o__Chitinophagales	f__Chitinophagaceae	g__Ferruginibacter	s__unclassified_g__Ferruginibacter	OTU2350
d__Bacteria	k__norank_d__Bacteria	p__Chloroflexi	c__AD3	o__norank_c__AD3	f__norank_o__norank_c__AD3	g__norank_f__norank_o__norank_c__AD3	s__unclassified_g__norank_f__norank_o__norank_c__AD3	OTU675
d__Bacteria	k__norank_d__Bacteria	p__Chloroflexi	c__AD3	o__norank_c__AD3	f__norank_o__norank_c__AD3	g__norank_f__norank_o__norank_c__AD3	s__unclassified_g__norank_f__norank_o__norank_c__AD3	OTU676
d__Bacteria	k__norank_d__Bacteria	p__Actinobacteriota	c__Acidimicrobiia	o__IMCC26256	f__norank_o__IMCC26256	g__norank_f__norank_o__IMCC26256	s__unclassified_g__norank_f__norank_o__IMCC26256	OTU738
d__Bacteria	k__norank_d__Bacteria	p__WPS-2	c__norank_p__WPS-2	o__norank_c__norank_p__WPS-2	f__norank_o__norank_c__norank_p__WPS-2	g__norank_f__norank_o__norank_c__norank_p__WPS-2	s__uncultured_bacterium_g__norank_f__norank_o__norank_c__norank_p__WPS-2	OTU1884
d__Bacteria	k__norank_d__Bacteria	p__Patescibacteria	c__Saccharimonadia	o__Saccharimonadales	f__LWQ8	g__norank_f__LWQ8	s__unclassified_g__norank_f__LWQ8	OTU597
d__Bacteria	k__norank_d__Bacteria	p__Armatimonadota	c__Chthonomonadetes	o__Chthonomonadales	f__Chthonomonadaceae	g__Chthonomonas	s__uncultured_bacterium_g__Chthonomonas	OTU1683
d__Bacteria	k__norank_d__Bacteria	p__WPS-2	c__norank_p__WPS-2	o__norank_c__norank_p__WPS-2	f__norank_o__norank_c__norank_p__WPS-2	g__norank_f__norank_o__norank_c__norank_p__WPS-2	s__uncultured_bacterium_g__norank_f__norank_o__norank_c__norank_p__WPS-2	OTU1882
d__Bacteria	k__norank_d__Bacteria	p__Dependentiae	c__Babeliae	o__Babeliales	f__unclassified_o__Babeliales	g__unclassified_o__Babeliales	s__unclassified_o__Babeliales	OTU458
d__Bacteria	k__norank_d__Bacteria	p__Patescibacteria	c__Saccharimonadia	o__Saccharimonadales	f__norank_o__Saccharimonadales	g__norank_f__norank_o__Saccharimonadales	s__uncultured_bacterium_g__norank_f__norank_o__Saccharimonadales	OTU916
d__Bacteria	k__norank_d__Bacteria	p__Actinobacteriota	c__Actinobacteria	o__unclassified_c__Actinobacteria	f__unclassified_c__Actinobacteria	g__unclassified_c__Actinobacteria	s__unclassified_c__Actinobacteria	OTU1223
d__Bacteria	k__norank_d__Bacteria	p__Proteobacteria	c__Gammaproteobacteria	o__Diplorickettsiales	f__Diplorickettsiaceae	g__Aquicella	s__uncultured_bacterium_g__Aquicella	OTU379
d__Bacteria	k__norank_d__Bacteria	p__Actinobacteriota	c__Thermoleophilia	o__unclassified_c__Thermoleophilia	f__unclassified_c__Thermoleophilia	g__unclassified_c__Thermoleophilia	s__unclassified_c__Thermoleophilia	OTU1400
d__Bacteria	k__norank_d__Bacteria	p__Patescibacteria	c__Saccharimonadia	o__Saccharimonadales	f__LWQ8	g__norank_f__LWQ8	s__uncultured_bacterium_g__norank_f__LWQ8	OTU2531
d__Bacteria	k__norank_d__Bacteria	p__Myxococcota	c__Polyangia	o__Haliangiales	f__Haliangiaceae	g__Haliangium	s__unclassified_g__Haliangium	OTU835
d__Bacteria	k__norank_d__Bacteria	p__Bacteroidota	c__Bacteroidia	o__Cytophagales	f__Cyclobacteriaceae	g__Algoriphagus	s__uncultured_Bacteroidetes_bacterium_g__Algoriphagus	OTU173

d__Bacteria	k__norank_d__Bacteria	p__Proteobacteria	c__Gammaproteobacteria	o__Burkholderiales	f__Burkholderiaceae	g__Ralstonia	s__Ralstonia_pickettii	OTU866
d__Bacteria	k__norank_d__Bacteria	p__Patescibacteria	c__Saccharimonadia	o__Saccharimonadales	f__norank_o__Saccharimonadales	g__norank_f__norank_o__Saccharimonadales	s__unclassified_g__norank_f__norank_o__Saccharimonadales	OTU1121
d__Bacteria	k__norank_d__Bacteria	p__Proteobacteria	c__Gammaproteobacteria	o__Burkholderiales	f__Nitrosomonadaceae	g__mle1-7	s__unclassified_g__mle1-7	OTU1440
d__Bacteria	k__norank_d__Bacteria	p__Firmicutes	c__Bacilli	o__Lactobacillales	f__Streptococcaceae	g__Streptococcus	s__Streptococcus_anginosus	OTU382
d__Bacteria	k__norank_d__Bacteria	p__Deinococcota	c__Deinococci	o__Deinococcales	f__Trueperaceae	g__Truepera	s__uncultured_Deinococcales_bacterium	OTU2985
d__Bacteria	k__norank_d__Bacteria	p__Verrucomicrobiota	c__Chlamydiae	o__Chlamydiales	f__Parachlamydiaceae	g__Candidatus_Protochlamydia	s__metagenome_g__Candidatus_Protochlamydia	OTU1261
d__Bacteria	k__norank_d__Bacteria	p__Patescibacteria	c__Saccharimonadia	o__Saccharimonadales	f__unclassified_o__Saccharimonadales	g__unclassified_o__Saccharimonadales	s__unclassified_o__Saccharimonadales	OTU1722
d__Bacteria	k__norank_d__Bacteria	p__Proteobacteria	c__Alphaproteobacteria	o__Tistrellales	f__Geminicoccaceae	g__Geminicoccus	s__uncultured_bacterium_g__Geminicoccus	OTU519
d__Bacteria	k__norank_d__Bacteria	p__Verrucomicrobiota	c__Chlamydiae	o__Chlamydiales	f__Parachlamydiaceae	g__Candidatus_Protochlamydia	s__unclassified_g__Candidatus_Protochlamydia	OTU811
d__Bacteria	k__norank_d__Bacteria	p__Proteobacteria	c__Gammaproteobacteria	o__Burkholderiales	f__Comamonadaceae	g__Comamonas	s__unclassified_g__Comamonas	OTU2902
d__Bacteria	k__norank_d__Bacteria	p__Bdellovibrionota	c__Oligoflexia	o__053A03-B-DI-P58	f__norank_o__053A03-B-DI-P58	g__norank_f__norank_o__053A03-B-DI-P58	s__uncultured_bacterium_g__norank_f__norank_o__053A03-B-DI-P58	OTU1903
d__Bacteria	k__norank_d__Bacteria	p__Proteobacteria	c__Alphaproteobacteria	o__Caulobacterales	f__Caulobacteraceae	g__norank_f__Caulobacteraceae	s__metagenome_g__norank_f__Caulobacteraceae	OTU2566
d__Bacteria	k__norank_d__Bacteria	p__Actinobacteriota	c__Acidimicrobiia	o__Microtrichales	f__Ilumatobacteraceae	g__norank_f__Ilumatobacteraceae	s__uncultured_bacterium_g__norank_f__Ilumatobacteraceae	OTU2655
d__Bacteria	k__norank_d__Bacteria	p__Verrucomicrobiota	c__Verrucomicrobiae	o__Chthoniobacterales	f__Chthoniobacteraceae	g__Chthoniobacter	s__metagenome_g__Chthoniobacter	OTU2280
d__Bacteria	k__norank_d__Bacteria	p__Proteobacteria	c__Gammaproteobacteria	o__Legionellales	f__Legionellaceae	g__Legionella	s__uncultured_bacterium_g__Legionella	OTU2795
d__Bacteria	k__norank_d__Bacteria	p__Acidobacteriota	c__Acidobacteriae	o__Subgroup_2	f__norank_o__Subgroup_2	g__norank_f__norank_o__Subgroup_2	s__unclassified_g__norank_f__norank_o__Subgroup_2	OTU1615

d__Bacteria	k__norank_d__Bacteria	p__Proteobacteria	c__Gammaproteobacteria	o__Burkholderiales	f__Comamonadaceae	g__unclassified_f__Comamonadaceae	s__unclassified_f__Comamonadaceae	OTU86
d__Bacteria	k__norank_d__Bacteria	p__Acidobacteriota	c__Holophagae	o__Subgroup_7	f__norank_o__Subgroup_7	g__norank_f__norank_o__Subgroup_7	s__uncultured_Acidobacteria_bacterium_g__norank_f__norank_o__Subgroup_7	OTU1558
d__Bacteria	k__norank_d__Bacteria	p__Bacteroidota	c__Bacteroidia	o__Chitinophagales	f__unclassified_o__Chitinophagales	g__unclassified_o__Chitinophagales	s__unclassified_o__Chitinophagales	OTU1556
d__Bacteria	k__norank_d__Bacteria	p__Verrucomicrobiota	c__Verrucomicrobiae	o__Pedosphaerales	f__Pedosphaeraceae	g__norank_f__Pedosphaeraceae	s__unclassified_g__norank_f__Pedosphaeraceae	OTU856
d__Bacteria	k__norank_d__Bacteria	p__Bacteroidota	c__Bacteroidia	o__Chitinophagales	f__Chitinophagaceae	g__Edaphobaculum	s__unclassified_g__Edaphobaculum	OTU1600
d__Bacteria	k__norank_d__Bacteria	p__Actinobacteriota	c__Acidimicrobiia	o__Microtrichales	f__Ilumatobacteraceae	g__CL500-29_marine_group	s__unclassified_g__CL500-29_marine_group	OTU327
d__Bacteria	k__norank_d__Bacteria	p__Chloroflexi	c__Chloroflexia	o__Thermomicrobiales	f__JG30-KF-CM45	g__norank_f__JG30-KF-CM45	s__uncultured_Chloroflexi_bacterium_g__norank_f__JG30-KF-CM45	OTU37
d__Bacteria	k__norank_d__Bacteria	p__Gemmatimonadota	c__Gemmatimonadetes	o__Gemmatimonadales	f__Gemmatimonadaceae	g__norank_f__Gemmatimonadaceae	s__uncultured_soil_bacterium_g__norank_f__Gemmatimonadaceae	OTU603
d__Bacteria	k__norank_d__Bacteria	p__Proteobacteria	c__Gammaproteobacteria	o__Diplorickettsiales	f__Diplorickettsiaceae	g__norank_f__Diplorickettsiaceae	s__unclassified_g__norank_f__Diplorickettsiaceae	OTU735
d__Bacteria	k__norank_d__Bacteria	p__Myxococcota	c__Polyangia	o__Haliangiales	f__Haliangiaceae	g__Haliangium	s__uncultured_soil_bacterium_g__Haliangium	OTU981
d__Bacteria	k__norank_d__Bacteria	p__Chloroflexi	c__Dehalococcoidia	o__S085	f__norank_o__S085	g__norank_f__norank_o__S085	s__metagenome_g__norank_f__norank_o__S085	OTU2611
d__Bacteria	k__norank_d__Bacteria	p__Chloroflexi	c__Ktedonobacteria	o__Ktedonobacterales	f__Ktedonobacteraceae	g__JG30a-KF-32	s__unclassified_g__JG30a-KF-32	OTU1910
d__Bacteria	k__norank_d__Bacteria	p__Verrucomicrobiota	c__Chlamydiae	o__Chlamydiales	f__unclassified_o__Chlamydiales	g__unclassified_o__Chlamydiales	s__unclassified_o__Chlamydiales	OTU1377
d__Bacteria	k__norank_d__Bacteria	p__Bacteroidota	c__Rhodothermia	o__Rhodothermales	f__Rhodothermaceae	g__norank_f__Rhodothermaceae	s__unclassified_g__norank_f__Rhodothermaceae	OTU346
d__Bacteria	k__norank_d__Bacteria	p__Chloroflexi	c__Ktedonobacteria	o__Ktedonobacterales	f__Ktedonobacteraceae	g__JG30a-KF-32	s__uncultured_Chloroflexi_bacterium_g__JG30a-KF-32	OTU1998
d__Bacteria	k__norank_d__Bacteria	p__Chloroflexi	c__Chloroflexia	o__Thermomicrobiales	f__JG30-KF-CM45	g__norank_f__JG30-KF-CM45	s__metagenome_g__norank_f__JG30-KF-CM45	OTU2688

d__Bacteria	k__norank_ d__Bacteria	p__Proteobacteria	c__Gammaproteobacteria	o__Xanthomonadales	f__Xanthomonadaceae	g__Arenimonas	s__unclassified_g__Arenimonas	OTU2465
d__Bacteria	k__norank_ d__Bacteria	p__Verrucomicrobiota	c__Verrucomicrobiae	o__Chthoniobacterales	f__Chthoniobacteraceae	g__Chthoniobacter	s__uncultured_bacterium_g__Chthoniobacter	OTU2946
d__Bacteria	k__norank_ d__Bacteria	p__Gemmatimonadota	c__Gemmatimonadetes	o__Gemmatimonadales	f__Gemmatimonadaceae	g__Gemmatimonas	s__unclassified_g__Gemmatimonas	OTU2416
d__Bacteria	k__norank_ d__Bacteria	p__Cyanobacteria	c__Cyanobacteriia	o__Chloroplast	f__norank_o__Chloroplast	g__norank_f__norank_o__Chloroplast	s__unclassified_g__norank_f__norank_o__Chloroplast	OTU1959
d__Bacteria	k__norank_ d__Bacteria	p__Chloroflexi	c__Ktedonobacteria	o__C0119	f__norank_o__C0119	g__norank_f__norank_o__C0119	s__uncultured_soil_bacterium_g__norank_f__norank_o__C0119	OTU1764
d__Bacteria	k__norank_ d__Bacteria	p__Myxococcota	c__Polyangia	o__unclassified_c__Polyang ia	f__unclassified_c__Polyangia	g__unclassified_c__Polyangia	s__unclassified_c__Polyangia	OTU2378
d__Bacteria	k__norank_ d__Bacteria	p__Chloroflexi	c__Chloroflexia	o__Thermomicrobiales	f__JG30-KF-CM45	g__norank_f__JG30-KF-CM45	s__uncultured_soil_bacterium_g__norank_f__JG30-KF-CM45	OTU2218
d__Bacteria	k__norank_ d__Bacteria	p__Actinobacteriota	c__Actinobacteria	o__Kineosporiales	f__Kineosporiaceae	g__Angustibacter	s__unclassified_g__Angustibacter	OTU2551
d__Bacteria	k__norank_ d__Bacteria	p__Proteobacteria	c__Alphaproteobacteria	o__Sphingomonadales	f__Sphingomonadaceae	g__Sphingomonas	s__unclassified_g__Sphingomonas	OTU1146
d__Bacteria	k__norank_ d__Bacteria	p__Cyanobacteria	c__Cyanobacteriia	o__Chloroplast	f__norank_o__Chloroplast	g__norank_f__norank_o__Chloroplast	s__uncultured_Chlorophyta_g__norank	OTU1794
d__Bacteria	k__norank_ d__Bacteria	p__Proteobacteria	c__Gammaproteobacteria	o__Burkholderiales	f__Nitrosomonadaceae	g__MND1	s__metagenome_g__MND1	OTU2484
d__Bacteria	k__norank_ d__Bacteria	p__Myxococcota	c__Myxococcia	o__Myxococcales	f__Myxococcaceae	g__P3OB-42	s__unclassified_g__P3OB-42	OTU767
d__Bacteria	k__norank_ d__Bacteria	p__Actinobacteriota	c__Actinobacteria	o__Propionibacteriales	f__Propionibacteriaceae	g__Propioniciclava	s__unclassified_g__Propioniciclava	OTU2185
d__Bacteria	k__norank_ d__Bacteria	p__Proteobacteria	c__Gammaproteobacteria	o__Burkholderiales	f__Nitrosomonadaceae	g__Ellin6067	s__uncultured_bacterium_g__Ellin6067	OTU2475
d__Bacteria	k__norank_ d__Bacteria	p__Gemmatimonadota	c__Gemmatimonadetes	o__Gemmatimonadales	f__Gemmatimonadaceae	g__Gemmatimonas	s__unclassified_g__Gemmatimonas	OTU2894
d__Bacteria	k__norank_ d__Bacteria	p__Myxococcota	c__Myxococcia	o__Myxococcales	f__Anaeromyxobacteraceae	g__Anaeromyxobacter	s__uncultured_bacterium_g__Anaeromyxobacter	OTU995

d__Bacteria	k__norank_ d__Bacteria	p__Proteobacteria	c__Alphaproteobacteria	o__Sphingomonadales	f__Sphingomonadaceae	g__Sphingomonas	s__uncultured_Alphaproteobacteria_bacterium_g__Sphingomonas	OTU2935
d__Bacteria	k__norank_ d__Bacteria	p__Bdellovibrionota	c__Bdellovibrionia	o__Bacteriovoracales	f__Bacteriovoracaceae	g__Peredibacter	s__unclassified_g__Peredibacter	OTU345
d__Bacteria	k__norank_ d__Bacteria	p__Bdellovibrionota	c__Bdellovibrionia	o__Bdellovibrionales	f__Bdellovibrionaceae	g__Bdellovibrio	s__Bdellovibrio_exovorus_JSS	OTU661
d__Bacteria	k__norank_ d__Bacteria	p__Bdellovibrionota	c__Bdellovibrionia	o__Bdellovibrionales	f__Bdellovibrionaceae	g__Bdellovibrio	s__metagenome_g__Bdellovibrio	OTU152
d__Bacteria	k__norank_ d__Bacteria	p__Chloroflexi	c__Chloroflexia	o__Chloroflexales	f__Herpetosiphonaceae	g__Herpetosiphon	s__uncultured_Chloroflexi_bacterium_g__Herpetosiphon	OTU2491
d__Bacteria	k__norank_ d__Bacteria	p__Bdellovibrionota	c__Oligoflexia	o__0319-6G20	f__norank_o__0319-6G20	g__norank_f__norank_o__0319-6G20	s__unclassified_g__norank_f__norank_o__0319-6G20	OTU2680
d__Bacteria	k__norank_ d__Bacteria	p__Chloroflexi	c__Ktedonobacteria	o__C0119	f__norank_o__C0119	g__norank_f__norank_o__C0119	s__unclassified_g__norank_f__norank_o__C0119	OTU1139
d__Bacteria	k__norank_ d__Bacteria	p__Chloroflexi	c__AD3	o__norank_c__AD3	f__norank_o__norank_c__AD3	g__norank_f__norank_o__norank_c__ AD3	s__unclassified_g__norank_f__norank_o__norank_c__AD3	OTU1233
d__Bacteria	k__norank_ d__Bacteria	p__Acidobacteriota	c__Vicinamibacteria	o__Subgroup_17	f__norank_o__Subgroup_17	g__norank_f__norank_o__Subgroup_1 7	s__unclassified_g__norank_f__norank_o__Subgroup_17	OTU1446
d__Bacteria	k__norank_ d__Bacteria	p__Proteobacteria	c__Alphaproteobacteria	o__Sphingomonadales	f__Sphingomonadaceae	g__Altererythrobacter	s__unclassified_g__Altererythrobacter	OTU2247
d__Bacteria	k__norank_ d__Bacteria	p__Bacteroidota	c__Bacteroidia	o__Chitinophagales	f__Chitinophagaceae	g__Segetibacter	s__unclassified_g__Segetibacter	OTU2529
d__Bacteria	k__norank_ d__Bacteria	p__Bacteroidota	c__Bacteroidia	o__Cytophagales	f__Spirosomaceae	g__Spirosoma	s__unclassified_g__Spirosoma	OTU166
d__Bacteria	k__norank_ d__Bacteria	p__Bacteroidota	c__Bacteroidia	o__Chitinophagales	f__norank_o__Chitinophagales	g__norank_f__norank_o__Chitinophag ales	s__unclassified_g__norank_f__norank_o__Chitinophagales	OTU195
d__Bacteria	k__norank_ d__Bacteria	p__Chloroflexi	c__Ktedonobacteria	o__C0119	f__norank_o__C0119	g__norank_f__norank_o__C0119	s__uncultured_bacterium_g__norank_f__norank_o__C0119	OTU2576
d__Bacteria	k__norank_ d__Bacteria	p__Bdellovibrionota	c__Bdellovibrionia	o__Bdellovibrionales	f__Bdellovibrionaceae	g__Bdellovibrio	s__uncultured_bacterium_g__Bdellovibrio	OTU544
d__Bacteria	k__norank_ d__Bacteria	p__Proteobacteria	c__Alphaproteobacteria	o__Rhizobiales	f__Labraceae	g__Labrys	s__unclassified_g__Labrys	OTU758

d__Bacteria	k__norank_ d__Bacteria	p__Bacteroidota	c__Bacteroidia	o__Chitinophagales	f__Chitinophagaceae	g__Flavisolibacter	s__uncultured_bacterium_g__Flavisolibacter	OTU2270
d__Bacteria	k__norank_ d__Bacteria	p__Proteobacteria	c__Alphaproteobacteria	o__Acetobacterales	f__Acetobacteraceae	g__Acidicaldus	s__uncultured_Acetobacteraceae_bacterium_g__Acidicaldus	OTU2535
d__Bacteria	k__norank_ d__Bacteria	p__Actinobacteriota	c__Thermoleophilia	o__Solirubrobacterales	f__Solirubrobacteraceae	g__Conexibacter	s__Conexibacter_sp.	OTU2657
d__Bacteria	k__norank_ d__Bacteria	p__Acidobacteriota	c__Acidobacteriae	o__Acidobacteriales	f__Acidobacteriaceae_Subgroup_1	g__Granulicella	s__unclassified_g__Granulicella	OTU12
d__Bacteria	k__norank_ d__Bacteria	p__Chloroflexi	c__KD4-96	o__norank_c__KD4-96	f__norank_o__norank_c__KD4-96	g__norank_f__norank_o__norank_c__ KD4-96	s__unclassified_g__norank_f__norank_o__norank_c__KD4-96	OTU1178
d__Bacteria	k__norank_ d__Bacteria	p__Bacteroidota	c__Bacteroidia	o__Chitinophagales	f__norank_o__Chitinophagales	g__norank_f__norank_o__Chitinophag ales	s__unclassified_g__norank_f__norank_o__Chitinophagales	OTU283
d__Bacteria	k__norank_ d__Bacteria	p__Actinobacteriota	c__Actinobacteria	o__Propionibacteriales	f__Nocardioidaceae	g__Nocardioides	s__unclassified_g__Nocardioides	OTU2816
d__Bacteria	k__norank_ d__Bacteria	p__unclassified_k__no rank_d__Bacteria	c__unclassified_k__noran k_d__Bacteria	o__unclassified_k__norank_ d__Bacteria	f__unclassified_k__norank_d__Bacteria	g__unclassified_k__norank_d__Bacteri a	s__unclassified_k__norank_d__Bacteria	OTU2750
d__Bacteria	k__norank_ d__Bacteria	p__Actinobacteriota	c__Thermoleophilia	o__Solirubrobacterales	f__67-14	g__norank_f__67-14	s__uncultured_bacterium_g__norank_f__67-14	OTU1452
d__Bacteria	k__norank_ d__Bacteria	p__Chloroflexi	c__Ktedonobacteria	o__C0119	f__norank_o__C0119	g__norank_f__norank_o__C0119	s__uncultured_bacterium_g__norank_f__norank_o__C0119	OTU1874
d__Bacteria	k__norank_ d__Bacteria	p__Proteobacteria	c__Gammaproteobacteria	o__Xanthomonadales	f__Rhodanobacteraceae	g__Tahibacter	s__uncultured_bacterium_g__Tahibacter	OTU2095
d__Bacteria	k__norank_ d__Bacteria	p__Verrucomicrobiota	c__Verrucomicrobiae	o__Verrucomicrobiales	f__Verrucomicrobiaceae	g__norank_f__Verrucomicrobiaceae	s__uncultured_Verrucomicrobia_bacterium_g__norank_f__Verrucomicrobiaceae	OTU635
d__Bacteria	k__norank_ d__Bacteria	p__Planctomycetota	c__Planctomycetes	o__Isosphaerales	f__Isosphaeraceae	g__norank_f__Isosphaeraceae	s__unclassified_g__norank_f__Isosphaeraceae	OTU836
d__Bacteria	k__norank_ d__Bacteria	p__Chloroflexi	c__AD3	o__norank_c__AD3	f__norank_o__norank_c__AD3	g__norank_f__norank_o__norank_c__ AD3	s__unclassified_g__norank_f__norank_o__norank_c__AD3	OTU1183
d__Bacteria	k__norank_ d__Bacteria	p__Actinobacteriota	c__Thermoleophilia	o__Solirubrobacterales	f__Solirubrobacteraceae	g__Solirubrobacter	s__metagenome_g__Solirubrobacter	OTU2981
d__Bacteria	k__norank_ d__Bacteria	p__Bdellovibrionota	c__Bdellovibrionia	o__Bdellovibrionales	f__Bdellovibrionaceae	g__Bdellovibrio	s__uncultured_bacterium_g__Bdellovibrio	OTU507

d__Bacteria	k__norank_d__Bacteria	p__Acidobacteriota	c__Acidobacteriae	o__Subgroup_12	f__norank_o__Subgroup_12	g__norank_f__norank_o__Subgroup_12	s__uncultured_bacterium_g__norank_f__norank_o__Subgroup_12	OTU1021
d__Bacteria	k__norank_d__Bacteria	p__Planctomycetota	c__Planctomycetes	o__Gemmatales	f__Gemmataceae	g__norank_f__Gemmataceae	s__uncultured_planctomycete_g__norank_f__Gemmataceae	OTU1816
d__Bacteria	k__norank_d__Bacteria	p__Patescibacteria	c__Saccharimonadia	o__Saccharimonadales	f__LWQ8	g__norank_f__LWQ8	s__uncultured_bacterium_g__norank_f__LWQ8	OTU2043
d__Bacteria	k__norank_d__Bacteria	p__Actinobacteriota	c__Thermoleophilia	o__Gaiellales	f__Gaiellaceae	g__Gaiella	s__uncultured_bacterium_g__Gaiella	OTU2387
d__Bacteria	k__norank_d__Bacteria	p__Patescibacteria	c__Saccharimonadia	o__Saccharimonadales	f__norank_o__Saccharimonadales	g__norank_f__norank_o__Saccharimonadales	s__unclassified_g__norank_f__norank_o__Saccharimonadales	OTU2114
d__Bacteria	k__norank_d__Bacteria	p__unclassified_k__norank_d__Bacteria	c__unclassified_k__norank_d__Bacteria	o__unclassified_k__norank_d__Bacteria	f__unclassified_k__norank_d__Bacteria	g__unclassified_k__norank_d__Bacteria	s__unclassified_k__norank_d__Bacteria	OTU1566
d__Bacteria	k__norank_d__Bacteria	p__Patescibacteria	c__Saccharimonadia	o__Saccharimonadales	f__norank_o__Saccharimonadales	g__norank_f__norank_o__Saccharimonadales	s__uncultured_soil_bacterium_g__norank_f__norank_o__Saccharimonadales	OTU1122
d__Bacteria	k__norank_d__Bacteria	p__Gemmatimonadota	c__Gemmatimonadetes	o__Gemmatimonadales	f__Gemmatimonadaceae	g__Gemmatimonas	s__uncultured_bacterium_g__Gemmatimonas	OTU2740
d__Bacteria	k__norank_d__Bacteria	p__Proteobacteria	c__Alphaproteobacteria	o__Rhizobiales	f__A0839	g__norank_f__A0839	s__metagenome_g__norank_f__A0839	OTU1242
d__Bacteria	k__norank_d__Bacteria	p__Actinobacteriota	c__Acidimicrobiia	o__IMCC26256	f__norank_o__IMCC26256	g__norank_f__norank_o__IMCC26256	s__uncultured_Acidothermaceae_bacterium	OTU1342
d__Bacteria	k__norank_d__Bacteria	p__Planctomycetota	c__Planctomycetes	o__Isosphaerales	f__Isosphaeraceae	g__norank_f__Isosphaeraceae	s__unclassified_g__norank_f__Isosphaeraceae	OTU2785
d__Bacteria	k__norank_d__Bacteria	p__Acidobacteriota	c__Thermoanaerobaculia	o__Thermoanaerobaculales	f__Thermoanaerobaculaceae	g__Subgroup_10	s__Holophaga_sp._WY42	OTU2117
d__Bacteria	k__norank_d__Bacteria	p__Proteobacteria	c__Gammaproteobacteria	o__Burkholderiales	f__Burkholderiaceae	g__Burkholderia-Caballeronia-Paraburkholderia	s__uncultured_Burkholderiaceae_bacterium_g__Burkholderia-Caballeronia-Paraburkholderia	OTU2467
d__Bacteria	k__norank_d__Bacteria	p__Chloroflexi	c__Chloroflexia	o__Thermomicrobiales	f__JG30-KF-CM45	g__norank_f__JG30-KF-CM45	s__uncultured_Chloroflexi_bacterium_g__norank_f__JG30-KF-CM45	OTU2072
d__Bacteria	k__norank_d__Bacteria	p__Chloroflexi	c__Dehalococcoidia	o__S085	f__norank_o__S085	g__norank_f__norank_o__S085	s__uncultured_bacterium_g__norank_f__norank_o__S085	OTU1425
d__Bacteria	k__norank_d__Bacteria	p__Proteobacteria	c__Gammaproteobacteria	o__Burkholderiales	f__Comamonadaceae	g__unclassified_f__Comamonadaceae	s__unclassified_f__Comamonadaceae	OTU2942

d__Bacteria	k__norank_d__Bacteria	p__Armatimonadota	c__Armatimonadia	o__Armatimonadales	f__norank_o__Armatimonadales	g__norank_f__norank_o__Armatimonadales	s__uncultured_Armatimonadetes_bacterium_g__norank_f__norank_o__Armatimonadales	OTU598
d__Bacteria	k__norank_d__Bacteria	p__Chloroflexi	c__Anaerolineae	o__SBR1031	f__norank_o__SBR1031	g__norank_f__norank_o__SBR1031	s__unclassified_g__norank_f__norank_o__SBR1031	OTU1357
d__Bacteria	k__norank_d__Bacteria	p__Patescibacteria	c__Microgenomatia	o__Candidatus_Levybacteria	f__norank_o__Candidatus_Levybacteria	g__norank_f__norank_o__Candidatus_Levybacteria	s__uncultured_bacterium_g__norank_f__norank_o__Candidatus_Levybacteria	OTU721
d__Bacteria	k__norank_d__Bacteria	p__Proteobacteria	c__Alphaproteobacteria	o__Rickettsiales	f__Mitochondria	g__norank_f__Mitochondria	s__unclassified_g__norank_f__Mitochondria	OTU1757
d__Bacteria	k__norank_d__Bacteria	p__Cyanobacteria	c__Sericytochromatia	o__norank_c__Sericytochromatia	f__norank_o__norank_c__Sericytochromatia	g__norank_f__norank_o__norank_c__Sericytochromatia	s__uncultured_cyanobacterium_g__norank_f__norank_o__norank_c__Sericytochromatia	OTU2253
d__Bacteria	k__norank_d__Bacteria	p__Bdellovibrionota	c__Oligoflexia	o__Oligoflexales	f__norank_o__Oligoflexales	g__norank_f__norank_o__Oligoflexales	s__uncultured_bacterium_g__norank_f__norank_o__Oligoflexales	OTU2714
d__Bacteria	k__norank_d__Bacteria	p__Bacteroidota	c__Bacteroidia	o__Flavobacteriales	f__Flavobacteriaceae	g__Flavobacterium	s__unclassified_g__Flavobacterium	OTU2898
d__Bacteria	k__norank_d__Bacteria	p__Patescibacteria	c__Saccharimonadia	o__Saccharimonadales	f__LWQ8	g__norank_f__LWQ8	s__uncultured_bacterium_g__norank_f__LWQ8	OTU330
d__Bacteria	k__norank_d__Bacteria	p__Bdellovibrionota	c__Bdellovibrionia	o__Bdellovibrionales	f__Bdellovibrionaceae	g__Bdellovibrio	s__unclassified_g__Bdellovibrio	OTU2709
d__Bacteria	k__norank_d__Bacteria	p__Bdellovibrionota	c__Oligoflexia	o__0319-6G20	f__norank_o__0319-6G20	g__norank_f__norank_o__0319-6G20	s__unclassified_g__norank_f__norank_o__0319-6G20	OTU621
d__Bacteria	k__norank_d__Bacteria	p__Proteobacteria	c__Gammaproteobacteria	o__Enterobacterales	f__Yersiniaceae	g__Yersinia	s__Yersinia_enterocolitica_g__Yersinia	OTU2955
d__Bacteria	k__norank_d__Bacteria	p__WPS-2	c__norank_p__WPS-2	o__norank_c__norank_p__WPS-2	f__norank_o__norank_c__norank_p__WPS-2	g__norank_f__norank_o__norank_c__norank_p__WPS-2	s__metagenome_g__norank_f__norank_o__norank_c__norank_p__WPS-2	OTU728
d__Bacteria	k__norank_d__Bacteria	p__Actinobacteriota	c__Thermoleophilia	o__Solirubrobacterales	f__Solirubrobacteraceae	g__Conexibacter	s__unclassified_g__Conexibacter	OTU2679
d__Bacteria	k__norank_d__Bacteria	p__Actinobacteriota	c__Actinobacteria	o__Propionibacteriales	f__Nocardioidaceae	g__Nocardioides	s__unclassified_g__Nocardioides	OTU2677
d__Bacteria	k__norank_d__Bacteria	p__Actinobacteriota	c__Actinobacteria	o__Streptosporangiales	f__Thermomonosporaceae	g__Actinocorallia	s__metagenome_g__Actinocorallia	OTU2632
d__Bacteria	k__norank_d__Bacteria	p__Acidobacteriota	c__Blastocatellia	o__DS-100	f__norank_o__DS-100	g__norank_f__norank_o__DS-100	s__unclassified_g__norank_f__norank_o__DS-100	OTU1431

d__Bacteria	k__norank_ d__Bacteria	p__Gemmatimonadota	c__Gemmatimonadetes	o__Gemmatimonadales	f__Gemmatimonadaceae	g__Gemmatimonas	s__unclassified_g__Gemmatimonas	OTU1557
d__Bacteria	k__norank_ d__Bacteria	p__Chloroflexi	c__JG30-KF-CM66	o__norank_c__JG30-KF-C M66	f__norank_o__norank_c__JG30-KF-CM 66	g__norank_f__norank_o__norank_c__ JG30-KF-CM66	s__metagenome_g__norank_f__norank_o__norank_c__JG30-KF-CM66	OTU1228
d__Bacteria	k__norank_ d__Bacteria	p__Chloroflexi	c__Anaerolineae	o__SBR1031	f__norank_o__SBR1031	g__norank_f__norank_o__SBR1031	s__wastewater_metagenome_g__norank_f__norank_o__SBR1031	OTU1519
d__Bacteria	k__norank_ d__Bacteria	p__Bdellovibrionota	c__Bdellovibrionia	o__Bdellovibrionales	f__Bdellovibrionaceae	g__OM27_clade	s__unclassified_g__OM27_clade	OTU290
d__Bacteria	k__norank_ d__Bacteria	p__Bdellovibrionota	c__Bdellovibrionia	o__Bdellovibrionales	f__Bdellovibrionaceae	g__Bdellovibrio	s__uncultured_bacterium_g__Bdellovibrio	OTU2610
d__Bacteria	k__norank_ d__Bacteria	p__Chloroflexi	c__Ktedonobacteria	o__C0119	f__norank_o__C0119	g__norank_f__norank_o__C0119	s__uncultured_bacterium_g__norank_f__norank_o__C0119	OTU2597
d__Bacteria	k__norank_ d__Bacteria	p__Bacteroidota	c__Bacteroidia	o__Chitinophagales	f__Chitinophagaceae	g__Cnuella	s__uncultured_bacterium_g__Cnuella	OTU2786
d__Bacteria	k__norank_ d__Bacteria	p__Dependentiae	c__Babeliae	o__Babeliales	f__Vermiphilaceae	g__norank_f__Vermiphilaceae	s__uncultured_bacterium_g__norank_f__Vermiphilaceae	OTU2257
d__Bacteria	k__norank_ d__Bacteria	p__Gemmatimonadota	c__Gemmatimonadetes	o__Gemmatimonadales	f__Gemmatimonadaceae	g__Gemmatimonas	s__uncultured_bacterium_g__Gemmatimonas	OTU352
d__Bacteria	k__norank_ d__Bacteria	p__Chloroflexi	c__Ktedonobacteria	o__C0119	f__norank_o__C0119	g__norank_f__norank_o__C0119	s__unclassified_g__norank_f__norank_o__C0119	OTU1287
d__Bacteria	k__norank_ d__Bacteria	p__Proteobacteria	c__Alphaproteobacteria	o__Acetobacterales	f__Acetobacteraceae	g__Acidiphilium	s__uncultured_bacterium_g__Acidiphilium	OTU66
d__Bacteria	k__norank_ d__Bacteria	p__Gemmatimonadota	c__Gemmatimonadetes	o__Gemmatimonadales	f__Gemmatimonadaceae	g__Gemmatimonas	s__uncultured_bacterium_g__Gemmatimonas	OTU354
d__Bacteria	k__norank_ d__Bacteria	p__Myxococcota	c__Myxococcia	o__Myxococcales	f__Myxococcaceae	g__unclassified_f__Myxococcaceae	s__unclassified_f__Myxococcaceae	OTU746
d__Bacteria	k__norank_ d__Bacteria	p__Actinobacteriota	c__Thermoleophilia	o__Gaiellales	f__norank_o__Gaiellales	g__norank_f__norank_o__Gaiellales	s__bacterium_Ellin6515	OTU1429
d__Bacteria	k__norank_ d__Bacteria	p__Acidobacteriota	c__Vicinamibacteria	o__Vicinamibacterales	f__Vicinamibacteraceae	g__Vicinamibacter	s__uncultured_bacterium_g__Vicinamibacter	OTU2752
d__Bacteria	k__norank_ d__Bacteria	p__Actinobacteriota	c__Thermoleophilia	o__Solirubrobacterales	f__67-14	g__norank_f__67-14	s__unclassified_g__norank_f__67-14	OTU1416

d__Bacteria	k__norank_ d__Bacteria	p__Proteobacteria	c__Alphaproteobacteria	o__Rhizobiales	f__Methyloligellaceae	g__norank_f__Methyloligellaceae	s__unclassified_g__norank_f__Methyloligellaceae	OTU2142
d__Bacteria	k__norank_ d__Bacteria	p__Proteobacteria	c__Alphaproteobacteria	o__Caulobacterales	f__Hyphomonadaceae	g__norank_f__Hyphomonadaceae	s__uncultured_bacterium_g__norank_f__Hyphomonadaceae	OTU2359
d__Bacteria	k__norank_ d__Bacteria	p__Actinobacteriota	c__Thermoleophilia	o__Gaiellales	f__norank_o__Gaiellales	g__norank_f__norank_o__Gaiellales	s__bacterium_Ellin6515	OTU1422
d__Bacteria	k__norank_ d__Bacteria	p__Cyanobacteria	c__Vampirivibrionia	o__Obscuribacterales	f__Obscuribacteraceae	g__norank_f__Obscuribacteraceae	s__uncultured_bacterium_g__norank_f__Obscuribacteraceae	OTU778
d__Bacteria	k__norank_ d__Bacteria	p__Proteobacteria	c__Gammaproteobacteria	o__Diplorickettsiales	f__Diplorickettsiaceae	g__Aquicella	s__unclassified_g__Aquicella	OTU834
d__Bacteria	k__norank_ d__Bacteria	p__Chloroflexi	c__Chloroflexia	o__Thermomicrobiales	f__JG30-KF-CM45	g__norank_f__JG30-KF-CM45	s__unclassified_g__norank_f__JG30-KF-CM45	OTU2873
d__Bacteria	k__norank_ d__Bacteria	p__Gemmatimonadota	c__Longimicrobia	o__Longimicrobiales	f__Longimicrobiaceae	g__norank_f__Longimicrobiaceae	s__unclassified_g__norank_f__Longimicrobiaceae	OTU2640
d__Bacteria	k__norank_ d__Bacteria	p__Actinobacteriota	c__Acidimicrobiia	o__IMCC26256	f__norank_o__IMCC26256	g__norank_f__norank_o__IMCC26256	s__uncultured_bacterium_g__norank_f__norank_o__IMCC26256	OTU2428
d__Bacteria	k__norank_ d__Bacteria	p__Planctomycetota	c__Planctomycetes	o__Isosphaerales	f__Isosphaeraceae	g__Singulisphaera	s__unclassified_g__Singulisphaera	OTU28
d__Bacteria	k__norank_ d__Bacteria	p__Patescibacteria	c__Saccharimonadia	o__Saccharimonadales	f__LWQ8	g__norank_f__LWQ8	s__uncultured_bacterium_g__norank_f__LWQ8	OTU1839
d__Bacteria	k__norank_ d__Bacteria	p__Actinobacteriota	c__Thermoleophilia	o__norank_c__Thermoleoph ilia	f__norank_o__norank_c__Thermoleophil ia	g__norank_f__norank_o__norank_c__ Thermoleophilia	s__uncultured_bacterium_g__norank_f__norank_o__norank_c__Thermoleophilia	OTU1374
d__Bacteria	k__norank_ d__Bacteria	p__Dependentiae	c__Babeliae	o__Babeliales	f__Vermiphilaceae	g__norank_f__Vermiphilaceae	s__uncultured_bacterium_g__norank_f__Vermiphilaceae	OTU2670
d__Bacteria	k__norank_ d__Bacteria	p__Verrucomicrobiota	c__Chlamydiae	o__Chlamydiales	f__Parachlamydiaceae	g__unclassified_f__Parachlamydiaceae	s__unclassified_f__Parachlamydiaceae	OTU722
d__Bacteria	k__norank_ d__Bacteria	p__Actinobacteriota	c__Thermoleophilia	o__Solirubrobacterales	f__67-14	g__norank_f__67-14	s__uncultured_actinobacterium_g__norank_f__67-14	OTU2437
d__Bacteria	k__norank_ d__Bacteria	p__Actinobacteriota	c__Actinobacteria	o__Propionibacteriales	f__Nocardioidaceae	g__unclassified_f__Nocardioidaceae	s__unclassified_f__Nocardioidaceae	OTU2937
d__Bacteria	k__norank_ d__Bacteria	p__Bacteroidota	c__Bacteroidia	o__Sphingobacteriales	f__Sphingobacteriaceae	g__Pedobacter	s__Pedobacter_sp._PACM_27299	OTU939

d__Bacteria	k__norank_ d__Bacteria	p__Actinobacteriota	c__Actinobacteria	o__Corynebacteriales	f__Mycobacteriaceae	g__Mycobacterium	s__unclassified_g__Mycobacterium	OTU1679
d__Bacteria	k__norank_ d__Bacteria	p__Acidobacteriota	c__Acidobacteriae	o__Solibacterales	f__Solibacteraceae	g__Candidatus_Solibacter	s__unclassified_g__Candidatus_Solibacter	OTU555
d__Bacteria	k__norank_ d__Bacteria	p__Chloroflexi	c__JG30-KF-CM66	o__norank_c__JG30-KF-C M66	f__norank_o__norank_c__JG30-KF-CM 66	g__norank_f__norank_o__norank_c__ JG30-KF-CM66	s__uncultured_soil_bacterium_g__norank_f__norank_o__norank_c__JG30-KF-CM 66	OTU1797
d__Bacteria	k__norank_ d__Bacteria	p__Myxococcota	c__Myxococcia	o__Myxococcales	f__Myxococcaceae	g__norank_f__Myxococcaceae	s__uncultured_bacterium_g__norank_f__Myxococcaceae	OTU2951
d__Bacteria	k__norank_ d__Bacteria	p__Sumerlaeota	c__Sumerlaeia	o__Sumerlaeales	f__Sumerlaeaceae	g__Sumerlaea	s__uncultured_Desulfocaldus_sp._g__Sumerlaea	OTU626
d__Bacteria	k__norank_ d__Bacteria	p__Bacteroidota	c__Bacteroidia	o__Chitinophagales	f__Chitinophagaceae	g__Dinghuibacter	s__uncultured_bacterium_g__Dinghuibacter	OTU1621
d__Bacteria	k__norank_ d__Bacteria	p__Proteobacteria	c__Alphaproteobacteria	o__Reyranellales	f__Reyranellaceae	g__norank_f__Reyranellaceae	s__metagenome_g__norank_f__Reyranellaceae	OTU2086
d__Bacteria	k__norank_ d__Bacteria	p__unclassified_k__no rank_d__Bacteria	c__unclassified_k__noran k_d__Bacteria	o__unclassified_k__norank_ d__Bacteria	f__unclassified_k__norank_d__Bacteria	g__unclassified_k__norank_d__Bacteri a	s__unclassified_k__norank_d__Bacteria	OTU2639
d__Bacteria	k__norank_ d__Bacteria	p__Bacteroidota	c__Bacteroidia	o__Chitinophagales	f__Chitinophagaceae	g__Ferruginibacter	s__uncultured_Bacteroidetes_bacterium_g__Ferruginibacter	OTU2307
d__Bacteria	k__norank_ d__Bacteria	p__Bacteroidota	c__Bacteroidia	o__Cytophagales	f__Hymenobacteraceae	g__Hymenobacter	s__unclassified_g__Hymenobacter	OTU478
d__Bacteria	k__norank_ d__Bacteria	p__Myxococcota	c__Polyangia	o__mle1-27	f__norank_o__mle1-27	g__norank_f__norank_o__mle1-27	s__unclassified_g__norank_f__norank_o__mle1-27	OTU818
d__Bacteria	k__norank_ d__Bacteria	p__Patescibacteria	c__unclassified_p__Patesc ibacteria	o__unclassified_p__Patescib acteria	f__unclassified_p__Patescibacteria	g__unclassified_p__Patescibacteria	s__unclassified_p__Patescibacteria	OTU964
d__Bacteria	k__norank_ d__Bacteria	p__Planctomycetota	c__Planctomycetes	o__Isosphaerales	f__Isosphaeraceae	g__norank_f__Isosphaeraceae	s__uncultured_Singulisphaera_sp._g__norank	OTU1952
d__Bacteria	k__norank_ d__Bacteria	p__Bacteroidota	c__Bacteroidia	o__Cytophagales	f__Spirosomaceae	g__Spirosoma	s__uncultured_bacterium_g__Spirosoma	OTU2452
d__Bacteria	k__norank_ d__Bacteria	p__Verrucomicrobiota	c__Verrucomicrobiae	o__Chthoniobacterales	f__Chthoniobacteraceae	g__Chthoniobacter	s__unclassified_g__Chthoniobacter	OTU2803
d__Bacteria	k__norank_ d__Bacteria	p__Acidobacteriota	c__Blastocatellia	o__Pyrinomonadales	f__Pyrinomonadaceae	g__RB41	s__uncultured_bacterium_g__RB41	OTU878

d__Bacteria	k__norank_ d__Bacteria	p__Planctomycetota	c__Planctomycetes	o__Gemmatales	f__Gemmataceae	g__norank_f__Gemmataceae	s__unclassified_g__norank_f__Gemmataceae	OTU2648
d__Bacteria	k__norank_ d__Bacteria	p__Proteobacteria	c__Alphaproteobacteria	o__Acetobacterales	f__Acetobacteraceae	g__norank_f__Acetobacteraceae	s__metagenome_g__norank_f__Acetobacteraceae	OTU1190
d__Bacteria	k__norank_ d__Bacteria	p__Bdellovibrionota	c__Bdellovibrionia	o__Bdellovibrionales	f__Bdellovibrionaceae	g__Bdellovibrio	s__uncultured_bacterium_g__Bdellovibrio	OTU1096
d__Bacteria	k__norank_ d__Bacteria	p__Cyanobacteria	c__Cyanobacteriia	o__Chloroplast	f__norank_o__Chloroplast	g__norank_f__norank_o__Chloroplast	s__unclassified_g__norank_f__norank_o__Chloroplast	OTU1726
d__Bacteria	k__norank_ d__Bacteria	p__Cyanobacteria	c__Cyanobacteriia	o__Chloroplast	f__norank_o__Chloroplast	g__norank_f__norank_o__Chloroplast	s__unclassified_g__norank_f__norank_o__Chloroplast	OTU1725
d__Bacteria	k__norank_ d__Bacteria	p__Proteobacteria	c__Alphaproteobacteria	o__Rhizobiales	f__Hyphomicrobiaceae	g__Hyphomicrobium	s__unclassified_g__Hyphomicrobium	OTU2143
d__Bacteria	k__norank_ d__Bacteria	p__Planctomycetota	c__OM190	o__norank_c__OM190	f__norank_o__norank_c__OM190	g__norank_f__norank_o__norank_c__ OM190	s__uncultured_bacterium_g__norank_f__norank_o__norank_c__OM190	OTU2163
d__Bacteria	k__norank_ d__Bacteria	p__Actinobacteriota	c__Thermoleophilia	o__Solirubrobacterales	f__Solirubrobacteraceae	g__Conexibacter	s__uncultured_actinobacterium_g__Conexibacter	OTU2458
d__Bacteria	k__norank_ d__Bacteria	p__Actinobacteriota	c__Actinobacteria	o__Frankiales	f__Sporichthyaceae	g__Sporichthya	s__unclassified_g__Sporichthya	OTU2594
d__Bacteria	k__norank_ d__Bacteria	p__Proteobacteria	c__Gammaproteobacteria	o__Burkholderiales	f__TRA3-20	g__norank_f__TRA3-20	s__unclassified_g__norank_f__TRA3-20	OTU2013
d__Bacteria	k__norank_ d__Bacteria	p__Planctomycetota	c__Phycisphaerae	o__Phycisphaerales	f__Phycisphaeraceae	g__SM1A02	s__unclassified_g__SM1A02	OTU2642
d__Bacteria	k__norank_ d__Bacteria	p__Chloroflexi	c__Anaerolineae	o__SBR1031	f__norank_o__SBR1031	g__norank_f__norank_o__SBR1031	s__unclassified_g__norank_f__norank_o__SBR1031	OTU2118
d__Bacteria	k__norank_ d__Bacteria	p__Proteobacteria	c__Alphaproteobacteria	o__Acetobacterales	f__Acetobacteraceae	g__Acidiphilium	s__uncultured_bacterium_g__Acidiphilium	OTU31
d__Bacteria	k__norank_ d__Bacteria	p__Bacteroidota	c__Bacteroidia	o__Cytophagales	f__Hymenobacteraceae	g__Hymenobacter	s__uncultured_Bacteroidetes_bacterium_g__Hymenobacter	OTU117
d__Bacteria	k__norank_ d__Bacteria	p__Chloroflexi	c__Ktedonobacteria	o__Ktedonobacterales	f__Ktedonobacteraceae	g__JG30a-KF-32	s__unclassified_g__JG30a-KF-32	OTU1835
d__Bacteria	k__norank_ d__Bacteria	p__Bacteroidota	c__Bacteroidia	o__Chitinophagales	f__Chitinophagaceae	g__Ferruginibacter	s__uncultured_bacterium_g__Ferruginibacter	OTU1055

d__Bacteria	k__norank_ d__Bacteria	p__Chloroflexi	c__Chloroflexia	o__Kallotenuales	f__AKIW781	g__norank_f__AKIW781	s__uncultured_endolithic_bacterium_g__norank_f__AKIW781	OTU2847
d__Bacteria	k__norank_ d__Bacteria	p__Chloroflexi	c__Ktedonobacteria	o__C0119	f__norank_o__C0119	g__norank_f__norank_o__C0119	s__uncultured_candidate_division_SAM_bacterium	OTU1849
d__Bacteria	k__norank_ d__Bacteria	p__Proteobacteria	c__Gammaproteobacteria	o__Legionellales	f__Legionellaceae	g__Legionella	s__uncultured_bacterium_g__Legionella	OTU391
d__Bacteria	k__norank_ d__Bacteria	p__Bacteroidota	c__Bacteroidia	o__Cytophagales	f__Cytophagaceae	g__Cytophaga	s__unclassified_g__Cytophaga	OTU1898
d__Bacteria	k__norank_ d__Bacteria	p__Verrucomicrobiota	c__Verrucomicrobiae	o__Pedosphaerales	f__Pedosphaeraceae	g__norank_f__Pedosphaeraceae	s__metagenome_g__norank_f__Pedosphaeraceae	OTU1099
d__Bacteria	k__norank_ d__Bacteria	p__Chloroflexi	c__TK10	o__norank_c__TK10	f__norank_o__norank_c__TK10	g__norank_f__norank_o__norank_c__ TK10	s__unclassified_g__norank_f__norank_o__norank_c__TK10	OTU196
d__Bacteria	k__norank_ d__Bacteria	p__Patescibacteria	c__Saccharimonadia	o__Saccharimonadales	f__norank_o__Saccharimonadales	g__norank_f__norank_o__Saccharimo nadales	s__unclassified_g__norank_f__norank_o__Saccharimonadales	OTU2662
d__Bacteria	k__norank_ d__Bacteria	p__Actinobacteriota	c__Actinobacteria	o__Micrococcales	f__Microbacteriaceae	g__Microbacterium	s__unclassified_g__Microbacterium	OTU212
d__Bacteria	k__norank_ d__Bacteria	p__Armatimonadota	c__Chthonomonadetes	o__Chthonomonadales	f__norank_o__Chthonomonadales	g__norank_f__norank_o__Chthonomo nadales	s__unclassified_g__norank_f__norank_o__Chthonomonadales	OTU1715
d__Bacteria	k__norank_ d__Bacteria	p__Chloroflexi	c__Chloroflexia	o__Thermomicrobiales	f__AKYG1722	g__norank_f__AKYG1722	s__uncultured_bacterium_g__norank_f__AKYG1722	OTU2348
d__Bacteria	k__norank_ d__Bacteria	p__Proteobacteria	c__Gammaproteobacteria	o__Beggiatoales	f__Beggiatoaceae	g__norank_f__Beggiatoaceae	s__uncultured_gamma_proteobacterium_g__norank_f__Beggiatoaceae	OTU56
d__Bacteria	k__norank_ d__Bacteria	p__Bacteroidota	c__Bacteroidia	o__Cytophagales	f__Hymenobacteraceae	g__Hymenobacter	s__unclassified_g__Hymenobacter	OTU256
d__Bacteria	k__norank_ d__Bacteria	p__Cyanobacteria	c__Cyanobacteriia	o__Cyanobacteriales	f__Phormidiaceae	g__Tychonema_CCAP_1459-11B	s__unclassified_g__Tychonema_CCAP_1459-11B	OTU2365
d__Bacteria	k__norank_ d__Bacteria	p__Proteobacteria	c__Gammaproteobacteria	o__Gammaproteobacteria_I ncertae_Sedis	f__unclassified_o__Gammaproteobacteri a_Incertae_Sedis	g__Acidibacter	s__unclassified_g__Acidibacter	OTU2510
d__Bacteria	k__norank_ d__Bacteria	p__Actinobacteriota	c__Actinobacteria	o__Pseudonocardiales	f__Pseudonocardiaceae	g__Crossiella	s__uncultured_bacterium_g__Crossiella	OTU1044
d__Bacteria	k__norank_ d__Bacteria	p__Myxococcota	c__Polyangia	o__Haliangiales	f__Haliangiaceae	g__Haliangium	s__unclassified_g__Haliangium	OTU1241

d__Bacteria	k__norank_ d__Bacteria	p__Gemmatimonadota	c__Gemmatimonadetes	o__Gemmatimonadales	f__Gemmatimonadaceae	g__norank_f__Gemmatimonadaceae	s__uncultured_Alphaproteobacteria_bacterium_g__norank_f__Gemmatimonadaceae	OTU1642
d__Bacteria	k__norank_ d__Bacteria	p__Proteobacteria	c__Alphaproteobacteria	o__Rhizobiales	f__Beijerinckiaceae	g__Methylobacterium-Methylorubrum	s__uncultured_bacterium_g__Methylobacterium-Methylorubrum	OTU24
d__Bacteria	k__norank_ d__Bacteria	p__Bacteroidota	c__Bacteroidia	o__Cytophagales	f__Hymenobacteraceae	g__Hymenobacter	s__uncultured_bacterium_g__Hymenobacter	OTU2831
d__Bacteria	k__norank_ d__Bacteria	p__Proteobacteria	c__Gammaproteobacteria	o__Burkholderiales	f__Burkholderiaceae	g__Limnobacter	s__uncultured_bacterium_g__Limnobacter	OTU1271
d__Bacteria	k__norank_ d__Bacteria	p__Planctomycetota	c__vadinHA49	o__norank_c__vadinHA49	f__norank_o__norank_c__vadinHA49	g__norank_f__norank_o__norank_c__ vadinHA49	s__uncultured_bacterium_g__norank_f__norank_o__norank_c__vadinHA49	OTU2033
d__Bacteria	k__norank_ d__Bacteria	p__Proteobacteria	c__Gammaproteobacteria	o__Oceanospirillales	f__Pseudohongiellaceae	g__BIyi10	s__uncultured_bacterium_g__BIyi10	OTU2120
d__Bacteria	k__norank_ d__Bacteria	p__Proteobacteria	c__Alphaproteobacteria	o__Sphingomonadales	f__Sphingomonadaceae	g__Sphingoaurantiacus	s__uncultured_bacterium_g__Sphingoaurantiacus	OTU2251
d__Bacteria	k__norank_ d__Bacteria	p__Verrucomicrobiota	c__Verrucomicrobiae	o__Verrucomicrobiales	f__Verrucomicrobiaceae	g__norank_f__Verrucomicrobiaceae	s__uncultured_verrucomicrobium_DEV006	OTU2070
d__Bacteria	k__norank_ d__Bacteria	p__Firmicutes	c__Clostridia	o__Peptostreptococcales-Tis sierellales	f__Fusibacteraceae	g__Fusibacter	s__unclassified_g__Fusibacter	OTU1832
d__Bacteria	k__norank_ d__Bacteria	p__Bacteroidota	c__Bacteroidia	o__Cytophagales	f__Spirosomaceae	g__Spirosoma	s__unclassified_g__Spirosoma	OTU341
d__Bacteria	k__norank_ d__Bacteria	p__Myxococcota	c__Polyangia	o__Polyangiales	f__Polyangiaceae	g__Pajaroellobacter	s__unclassified_g__Pajaroellobacter	OTU1838
d__Bacteria	k__norank_ d__Bacteria	p__Proteobacteria	c__Alphaproteobacteria	o__Rhizobiales	f__Beijerinckiaceae	g__unclassified_f__Beijerinckiaceae	s__unclassified_f__Beijerinckiaceae	OTU2977
d__Bacteria	k__norank_ d__Bacteria	p__Acidobacteriota	c__Blastocatellia	o__Blastocatellales	f__Blastocatellaceae	g__Stenotrophobacter	s__unclassified_g__Stenotrophobacter	OTU2602
d__Bacteria	k__norank_ d__Bacteria	p__Bdellovibrionota	c__Bdellovibrionia	o__Bdellovibrionales	f__Bdellovibrionaceae	g__Bdellovibrio	s__unclassified_g__Bdellovibrio	OTU2399
d__Bacteria	k__norank_ d__Bacteria	p__Deinococcota	c__Deinococci	o__Deinococcales	f__Deinococcaceae	g__Deinococcus	s__unclassified_g__Deinococcus	OTU2967
d__Bacteria	k__norank_ d__Bacteria	p__Patescibacteria	c__Saccharimonadia	o__Saccharimonadales	f__norank_o__Saccharimonadales	g__norank_f__norank_o__Saccharimo nadales	s__uncultured_soil_bacterium_g__norank_f__norank_o__Saccharimonadales	OTU2226

d__Bacteria	k__norank_d__Bacteria	p__Chloroflexi	c__Chloroflexia	o__Chloroflexales	f__Herpetosiphonaceae	g__Herpetosiphon	s__Herpetosiphon_aurantiacus_DSM_785	OTU2864
d__Bacteria	k__norank_d__Bacteria	p__Proteobacteria	c__Alphaproteobacteria	o__Sphingomonadales	f__Sphingomonadaceae	g__Erythrobacter	s__unclassified_g__Erythrobacter	OTU494
d__Bacteria	k__norank_d__Bacteria	p__Myxococcota	c__Myxococcia	o__Myxococcales	f__Myxococcaceae	g__norank_f__Myxococcaceae	s__uncultured_bacterium_g__norank_f__Myxococcaceae	OTU2755
d__Bacteria	k__norank_d__Bacteria	p__unclassified_k__norank_d__Bacteria	c__unclassified_k__norank_d__Bacteria	o__unclassified_k__norank_d__Bacteria	f__unclassified_k__norank_d__Bacteria	g__unclassified_k__norank_d__Bacteria	s__unclassified_k__norank_d__Bacteria	OTU777
d__Bacteria	k__norank_d__Bacteria	p__Gemmatimonadota	c__Gemmatimonadetes	o__Gemmatimonadales	f__Gemmatimonadaceae	g__norank_f__Gemmatimonadaceae	s__unclassified_g__norank_f__Gemmatimonadaceae	OTU1445
d__Bacteria	k__norank_d__Bacteria	p__Bacteroidota	c__Bacteroidia	o__Chitinophagales	f__Chitinophagaceae	g__Sediminibacterium	s__unclassified_g__Sediminibacterium	OTU1590
d__Bacteria	k__norank_d__Bacteria	p__Chloroflexi	c__Ktedonobacteria	o__Ktedonobacterales	f__Ktedonobacteraceae	g__JG30a-KF-32	s__uncultured_Chloroflexi_bacterium_g__JG30a-KF-32	OTU2003
d__Bacteria	k__norank_d__Bacteria	p__Proteobacteria	c__Gammaproteobacteria	o__Burkholderiales	f__Comamonadaceae	g__Caenimonas	s__unclassified_g__Caenimonas	OTU2441
d__Bacteria	k__norank_d__Bacteria	p__Bacteroidota	c__Bacteroidia	o__Chitinophagales	f__Chitinophagaceae	g__norank_f__Chitinophagaceae	s__unclassified_g__norank_f__Chitinophagaceae	OTU2328
d__Bacteria	k__norank_d__Bacteria	p__Proteobacteria	c__Gammaproteobacteria	o__Gammaproteobacteria_Incertae_Sedis	f__unclassified_o__Gammaproteobacteria_Incertae_Sedis	g__Candidatus_Berkiella	s__uncultured_bacterium_g__Candidatus_Berkiella	OTU2781
d__Bacteria	k__norank_d__Bacteria	p__Proteobacteria	c__Gammaproteobacteria	o__Xanthomonadales	f__Xanthomonadaceae	g__Thermomonas	s__uncultured_bacterium_g__Thermomonas	OTU443
d__Bacteria	k__norank_d__Bacteria	p__Chloroflexi	c__Chloroflexia	o__Thermomicrobiales	f__JG30-KF-CM45	g__norank_f__JG30-KF-CM45	s__unclassified_g__norank_f__JG30-KF-CM45	OTU606
d__Bacteria	k__norank_d__Bacteria	p__Chloroflexi	c__Ktedonobacteria	o__B12-WMSP1	f__norank_o__B12-WMSP1	g__norank_f__norank_o__B12-WMSP1	s__uncultured_bacterium_g__norank_f__norank_o__B12-WMSP1	OTU1872
d__Bacteria	k__norank_d__Bacteria	p__Bacteroidota	c__Bacteroidia	o__Cytophagales	f__unclassified_o__Cytophagales	g__unclassified_o__Cytophagales	s__unclassified_o__Cytophagales	OTU1742
d__Bacteria	k__norank_d__Bacteria	p__Bacteroidota	c__Bacteroidia	o__Cytophagales	f__Spirosomaceae	g__Fibrella	s__Fibrella_sp._ES10-3-2-2	OTU2326
d__Bacteria	k__norank_d__Bacteria	p__Actinobacteriota	c__Thermoleophilia	o__Gaiellales	f__norank_o__Gaiellales	g__norank_f__norank_o__Gaiellales	s__uncultured_organism_g__norank_f__norank_o__Gaiellales	OTU2314

d__Bacteria	k__norank_ d__Bacteria	p__Acidobacteriota	c__Vicinamibacteria	o__Vicinamibacterales	f__norank_o__Vicinamibacterales	g__norank_f__norank_o__Vicinamiba cterales	s__uncultured_bacterium_g__norank_f__norank_o__Vicinamibacterales	OTU1204
d__Bacteria	k__norank_ d__Bacteria	p__Bacteroidota	c__Bacteroidia	o__Chitinophagales	f__Chitinophagaceae	g__Ferruginibacter	s__uncultured_bacterium_g__Ferruginibacter	OTU1264
d__Bacteria	k__norank_ d__Bacteria	p__Myxococcota	c__Myxococcia	o__Myxococcales	f__27F-1492R	g__norank_f__27F-1492R	s__uncultured_bacterium_g__norank_f__27F-1492R	OTU2666
d__Bacteria	k__norank_ d__Bacteria	p__Firmicutes	c__Bacilli	o__Lactobacillales	f__Carnobacteriaceae	g__Carnobacterium	s__Carnobacterium_inhibens_subsp._inhibens_DSM_13024	OTU2914
d__Bacteria	k__norank_ d__Bacteria	p__Actinobacteriota	c__Actinobacteria	o__Propionibacteriales	f__Nocardioidaceae	g__Marmoricola	s__uncultured_bacterium_g__Marmoricola	OTU89
d__Bacteria	k__norank_ d__Bacteria	p__Bdellovibrionota	c__Oligoflexia	o__0319-6G20	f__norank_o__0319-6G20	g__norank_f__norank_o__0319-6G20	s__unclassified_g__norank_f__norank_o__0319-6G20	OTU1082
d__Bacteria	k__norank_ d__Bacteria	p__Proteobacteria	c__Gammaproteobacteria	o__Burkholderiales	f__Comamonadaceae	g__unclassified_f__Comamonadaceae	s__unclassified_f__Comamonadaceae	OTU2454
d__Bacteria	k__norank_ d__Bacteria	p__Proteobacteria	c__Gammaproteobacteria	o__Gammaproteobacteria_I ncertae_Sedis	f__unclassified_o__Gammaproteobacteri a_Incertae_Sedis	g__Acidibacter	s__uncultured_bacterium_g__Acidibacter	OTU1371
d__Bacteria	k__norank_ d__Bacteria	p__Bacteroidota	c__Bacteroidia	o__Chitinophagales	f__Chitinophagaceae	g__Edaphobaculum	s__metagenome_g__Edaphobaculum	OTU2828
d__Bacteria	k__norank_ d__Bacteria	p__unclassified_k__no rank_d__Bacteria	c__unclassified_k__noran k_d__Bacteria	o__unclassified_k__norank_ d__Bacteria	f__unclassified_k__norank_d__Bacteria	g__unclassified_k__norank_d__Bacteri a	s__unclassified_k__norank_d__Bacteria	OTU1948
d__Bacteria	k__norank_ d__Bacteria	p__Patescibacteria	c__norank_p__Patescibact eria	o__norank_c__norank_p__P atescibacteria	f__norank_o__norank_c__norank_p__Pat escibacteria	g__norank_f__norank_o__norank_c__ norank_p__Patescibacteria	s__uncultured_bacterium_g__norank_f__norank_o__norank_c__norank_p__Patesci bacteria	OTU1498
d__Bacteria	k__norank_ d__Bacteria	p__Myxococcota	c__Polyangia	o__Polyangiales	f__Polyangiaceae	g__unclassified_f__Polyangiaceae	s__unclassified_f__Polyangiaceae	OTU2775
d__Bacteria	k__norank_ d__Bacteria	p__Myxococcota	c__Polyangia	o__Polyangiales	f__BIrii41	g__norank_f__BIrii41	s__metagenome_g__norank_f__BIrii41	OTU923
d__Bacteria	k__norank_ d__Bacteria	p__Proteobacteria	c__Gammaproteobacteria	o__Xanthomonadales	f__Xanthomonadaceae	g__Lysobacter	s__uncultured_gamma_proteobacterium_g__Lysobacter	OTU1274
d__Bacteria	k__norank_ d__Bacteria	p__unclassified_k__no rank_d__Bacteria	c__unclassified_k__noran k_d__Bacteria	o__unclassified_k__norank_ d__Bacteria	f__unclassified_k__norank_d__Bacteria	g__unclassified_k__norank_d__Bacteri a	s__unclassified_k__norank_d__Bacteria	OTU1943
d__Bacteria	k__norank_ d__Bacteria	p__Fibrobacterota	c__Fibrobacteria	o__Fibrobacterales	f__Fibrobacteraceae	g__possible_genus_04	s__uncultured_bacterium_g__possible_genus_04	OTU205

d__Bacteria	k__norank_d__Bacteria	p__Bacteroidota	c__Bacteroidia	o__Cytophagales	f__Spirosomaceae	g__Spirosoma	s__uncultured_bacterium_g__Spirosoma	OTU2329
d__Bacteria	k__norank_d__Bacteria	p__Myxococcota	c__Polyangia	o__Polyangiales	f__Sandaracinaceae	g__norank_f__Sandaracinaceae	s__uncultured_soil_bacterium_g__norank_f__Sandaracinaceae	OTU1331
d__Bacteria	k__norank_d__Bacteria	p__Proteobacteria	c__Gammaproteobacteria	o__Gammaproteobacteria_Incertae_Sedis	f__unclassified_o__Gammaproteobacteria_Incertae_Sedis	g__Candidatus_Ovatusbacter	s__uncultured_bacterium_g__Candidatus_Ovatusbacter	OTU868
d__Bacteria	k__norank_d__Bacteria	p__Myxococcota	c__Polyangia	o__Polyangiales	f__Polyangiaceae	g__Pajaroellobacter	s__unclassified_g__Pajaroellobacter	OTU2637
d__Bacteria	k__norank_d__Bacteria	p__Bacteroidota	c__Bacteroidia	o__Cytophagales	f__Hymenobacteraceae	g__Hymenobacter	s__uncultured_Hymenobacter_sp._g__Hymenobacter	OTU1291
d__Bacteria	k__norank_d__Bacteria	p__unclassified_k__norank_d__Bacteria	c__unclassified_k__norank_d__Bacteria	o__unclassified_k__norank_d__Bacteria	f__unclassified_k__norank_d__Bacteria	g__unclassified_k__norank_d__Bacteria	s__unclassified_k__norank_d__Bacteria	OTU1771
d__Bacteria	k__norank_d__Bacteria	p__Patescibacteria	c__Saccharimonadia	o__Saccharimonadales	f__LWQ8	g__norank_f__LWQ8	s__unclassified_g__norank_f__LWQ8	OTU170
d__Bacteria	k__norank_d__Bacteria	p__Chloroflexi	c__Ktedonobacteria	o__Ktedonobacterales	f__Ktedonobacteraceae	g__norank_f__Ktedonobacteraceae	s__unclassified_g__norank_f__Ktedonobacteraceae	OTU1902
d__Bacteria	k__norank_d__Bacteria	p__Proteobacteria	c__Alphaproteobacteria	o__Tistrellales	f__Geminicoccaceae	g__Candidatus_Alysiosphaera	s__uncultured_bacterium_g__Candidatus_Alysiosphaera	OTU2466
d__Bacteria	k__norank_d__Bacteria	p__Proteobacteria	c__Gammaproteobacteria	o__Xanthomonadales	f__Rhodanobacteraceae	g__Tahibacter	s__uncultured_bacterium_g__Tahibacter	OTU1620
d__Bacteria	k__norank_d__Bacteria	p__Myxococcota	c__Myxococcia	o__Myxococcales	f__Myxococcaceae	g__norank_f__Myxococcaceae	s__unclassified_g__norank_f__Myxococcaceae	OTU1329
d__Bacteria	k__norank_d__Bacteria	p__unclassified_k__norank_d__Bacteria	c__unclassified_k__norank_d__Bacteria	o__unclassified_k__norank_d__Bacteria	f__unclassified_k__norank_d__Bacteria	g__unclassified_k__norank_d__Bacteria	s__unclassified_k__norank_d__Bacteria	OTU988
d__Bacteria	k__norank_d__Bacteria	p__Deinococcota	c__Deinococci	o__Deinococcales	f__Deinococcaceae	g__Deinococcus	s__unclassified_g__Deinococcus	OTU119
d__Bacteria	k__norank_d__Bacteria	p__Proteobacteria	c__Gammaproteobacteria	o__Xanthomonadales	f__Xanthomonadaceae	g__Lysobacter	s__Lysobacter_sp._C1802	OTU453
d__Bacteria	k__norank_d__Bacteria	p__Bdellovibrionota	c__Bdellovibrionia	o__Bdellovibrionales	f__Bdellovibrionaceae	g__OM27_clade	s__metagenome_g__OM27_clade	OTU510
d__Bacteria	k__norank_d__Bacteria	p__Proteobacteria	c__Alphaproteobacteria	o__Rhizobiales	f__Xanthobacteraceae	g__Pseudolabrys	s__uncultured_bacterium_g__Pseudolabrys	OTU2241

d__Bacteria	k__norank_ d__Bacteria	p__Bdellovibrionota	c__Bdellovibrionia	o__Bacteriovoracales	f__Bacteriovoracaceae	g__Peredibacter	s__uncultured_bacterium_g__Peredibacter	OTU187
d__Bacteria	k__norank_ d__Bacteria	p__Actinobacteriota	c__Acidimicrobiia	o__Microtrichales	f__Ilumatobacteraceae	g__norank_f__Ilumatobacteraceae	s__uncultured_bacterium_g__norank_f__Ilumatobacteraceae	OTU2874
d__Bacteria	k__norank_ d__Bacteria	p__Actinobacteriota	c__Acidimicrobiia	o__IMCC26256	f__norank_o__IMCC26256	g__norank_f__norank_o__IMCC26256	s__uncultured_bacterium_g__norank_f__norank_o__IMCC26256	OTU2362
d__Bacteria	k__norank_ d__Bacteria	p__Myxococcota	c__Myxococcia	o__Myxococcales	f__Anaeromyxobacteraceae	g__Anaeromyxobacter	s__unclassified_g__Anaeromyxobacter	OTU81
d__Bacteria	k__norank_ d__Bacteria	p__Chloroflexi	c__Ktedonobacteria	o__Ktedonobacterales	f__Ktedonobacteraceae	g__unclassified_f__Ktedonobacteracea e	s__unclassified_f__Ktedonobacteraceae	OTU2032
d__Bacteria	k__norank_ d__Bacteria	p__Proteobacteria	c__Alphaproteobacteria	o__Elsterales	f__norank_o__Elsterales	g__norank_f__norank_o__Elsterales	s__uncultured_Alphaproteobacteria_bacterium_g__norank_f__norank_o__Elsterales	OTU1880
d__Bacteria	k__norank_ d__Bacteria	p__Acidobacteriota	c__Holophagae	o__Subgroup_7	f__norank_o__Subgroup_7	g__norank_f__norank_o__Subgroup_7	s__uncultured_bacterium_g__norank_f__norank_o__Subgroup_7	OTU1676
d__Bacteria	k__norank_ d__Bacteria	p__Proteobacteria	c__Alphaproteobacteria	o__Rhizobiales	f__KF-JG30-B3	g__norank_f__KF-JG30-B3	s__unclassified_g__norank_f__KF-JG30-B3	OTU2161
d__Bacteria	k__norank_ d__Bacteria	p__WPS-2	c__norank_p__WPS-2	o__norank_c__norank_p__ WPS-2	f__norank_o__norank_c__norank_p__W PS-2	g__norank_f__norank_o__norank_c__ norank_p__WPS-2	s__metagenome_g__norank_f__norank_o__norank_c__norank_p__WPS-2	OTU260
d__Bacteria	k__norank_ d__Bacteria	p__Bacteroidota	c__Bacteroidia	o__Chitinophagales	f__Chitinophagaceae	g__Segetibacter	s__unclassified_g__Segetibacter	OTU2201
d__Bacteria	k__norank_ d__Bacteria	p__Actinobacteriota	c__Acidimicrobiia	o__IMCC26256	f__norank_o__IMCC26256	g__norank_f__norank_o__IMCC26256	s__uncultured_Ferrimicrobium_sp._g__norank	OTU2574
d__Bacteria	k__norank_ d__Bacteria	p__Gemmatimonadota	c__Gemmatimonadetes	o__Gemmatimonadales	f__Gemmatimonadaceae	g__Roseisolibacter	s__Roseisolibacter_agri	OTU1594
d__Bacteria	k__norank_ d__Bacteria	p__Actinobacteriota	c__Acidimicrobiia	o__IMCC26256	f__norank_o__IMCC26256	g__norank_f__norank_o__IMCC26256	s__uncultured_Aciditerrimonas_sp._g__norank_f__norank	OTU1767
d__Bacteria	k__norank_ d__Bacteria	p__Chloroflexi	c__Anaerolineae	o__SBR1031	f__A4b	g__norank_f__A4b	s__uncultured_bacterium_g__norank_f__A4b	OTU2191
d__Bacteria	k__norank_ d__Bacteria	p__Bdellovibrionota	c__Bdellovibrionia	o__Bdellovibrionales	f__Bdellovibrionaceae	g__Bdellovibrio	s__uncultured_bacterium_g__Bdellovibrio	OTU150
d__Bacteria	k__norank_ d__Bacteria	p__Bacteroidota	c__Bacteroidia	o__Sphingobacteriales	f__env.OPS_17	g__norank_f__env.OPS_17	s__metagenome_g__norank_f__env.OPS_17	OTU1125

d__Bacteria	k__norank_d__Bacteria	p__unclassified_k__norank_d__Bacteria	c__unclassified_k__norank_d__Bacteria	o__unclassified_k__norank_d__Bacteria	f__unclassified_k__norank_d__Bacteria	g__unclassified_k__norank_d__Bacteria	s__unclassified_k__norank_d__Bacteria	OTU1175
d__Bacteria	k__norank_d__Bacteria	p__Bacteroidota	c__Bacteroidia	o__Chitinophagales	f__Chitinophagaceae	g__Segetibacter	s__unclassified_g__Segetibacter	OTU2020
d__Bacteria	k__norank_d__Bacteria	p__unclassified_k__norank_d__Bacteria	c__unclassified_k__norank_d__Bacteria	o__unclassified_k__norank_d__Bacteria	f__unclassified_k__norank_d__Bacteria	g__unclassified_k__norank_d__Bacteria	s__unclassified_k__norank_d__Bacteria	OTU1511
d__Bacteria	k__norank_d__Bacteria	p__Abditibacteriota	c__Abditibacteria	o__Abditibacteriales	f__Abditibacteriaceae	g__Abditibacterium	s__uncultured_bacterium_g__Abditibacterium	OTU2856
d__Bacteria	k__norank_d__Bacteria	p__Patescibacteria	c__Saccharimonadia	o__Saccharimonadales	f__LWQ8	g__norank_f__LWQ8	s__uncultured_bacterium_g__norank_f__LWQ8	OTU1912
d__Bacteria	k__norank_d__Bacteria	p__Actinobacteriota	c__Acidimicrobiia	o__Microtrichales	f__Iamiaceae	g__Iamia	s__metagenome_g__Iamia	OTU2879
d__Bacteria	k__norank_d__Bacteria	p__Actinobacteriota	c__Thermoleophilia	o__Solirubrobacterales	f__Solirubrobacteraceae	g__Conexibacter	s__Solirubrobacterales_bacterium_g__Conexibacter	OTU2789
d__Bacteria	k__norank_d__Bacteria	p__Proteobacteria	c__Gammaproteobacteria	o__Burkholderiales	f__Oxalobacteraceae	g__Noviherbaspirillum	s__uncultured_bacterium_g__Noviherbaspirillum	OTU918
d__Bacteria	k__norank_d__Bacteria	p__Proteobacteria	c__Gammaproteobacteria	o__Burkholderiales	f__unclassified_o__Burkholderiales	g__unclassified_o__Burkholderiales	s__unclassified_o__Burkholderiales	OTU2528
d__Bacteria	k__norank_d__Bacteria	p__Acidobacteriota	c__Vicinamibacteria	o__Vicinamibacterales	f__norank_o__Vicinamibacterales	g__norank_f__norank_o__Vicinamibacterales	s__unclassified_g__norank_f__norank_o__Vicinamibacterales	OTU2361
d__Bacteria	k__norank_d__Bacteria	p__Bacteroidota	c__Bacteroidia	o__Cytophagales	f__Hymenobacteraceae	g__Hymenobacter	s__uncultured_bacterium_g__Hymenobacter	OTU1938
d__Bacteria	k__norank_d__Bacteria	p__Bacteroidota	c__Bacteroidia	o__Chitinophagales	f__Chitinophagaceae	g__Edaphobaculum	s__unclassified_g__Edaphobaculum	OTU2504
d__Bacteria	k__norank_d__Bacteria	p__Chloroflexi	c__Anaerolineae	o__SBR1031	f__A4b	g__OLB13	s__uncultured_bacterium_g__OLB13	OTU2324
d__Bacteria	k__norank_d__Bacteria	p__Bacteroidota	c__Kapabacteria	o__Kapabacteriales	f__norank_o__Kapabacteriales	g__norank_f__norank_o__Kapabacteriales	s__uncultured_bacterium_KF-JG30-B11	OTU1631
d__Bacteria	k__norank_d__Bacteria	p__Chloroflexi	c__Ktedonobacteria	o__Ktedonobacterales	f__JG30-KF-AS9	g__norank_f__JG30-KF-AS9	s__uncultured_bacterium_g__norank_f__JG30-KF-AS9	OTU2026
d__Bacteria	k__norank_d__Bacteria	p__Proteobacteria	c__Gammaproteobacteria	o__Xanthomonadales	f__Rhodanobacteraceae	g__unclassified_f__Rhodanobacteraceae	s__unclassified_f__Rhodanobacteraceae	OTU258

d__Bacteria	k__norank_d__Bacteria	p__Proteobacteria	c__Alphaproteobacteria	o__Rhizobiales	f__Devosiaceae	g__Devosia	s__unclassified_g__Devosia	OTU2258
d__Bacteria	k__norank_d__Bacteria	p__Chloroflexi	c__Anaerolineae	o__SBR1031	f__norank_o__SBR1031	g__norank_f__norank_o__SBR1031	s__unclassified_g__norank_f__norank_o__SBR1031	OTU975
d__Bacteria	k__norank_d__Bacteria	p__Proteobacteria	c__Alphaproteobacteria	o__Rhizobiales	f__norank_o__Rhizobiales	g__norank_f__norank_o__Rhizobiales	s__uncultured_Alphaproteobacteria_bacterium_g__norank_f__norank_o__Rhizobiales	OTU2598
d__Bacteria	k__norank_d__Bacteria	p__Sumerlaeota	c__Sumerlaeia	o__Sumerlaeales	f__Sumerlaeaceae	g__Sumerlaea	s__unclassified_g__Sumerlaea	OTU893
d__Bacteria	k__norank_d__Bacteria	p__Proteobacteria	c__Gammaproteobacteria	o__Burkholderiales	f__Nitrosomonadaceae	g__norank_f__Nitrosomonadaceae	s__metagenome_g__norank_f__Nitrosomonadaceae	OTU1081
d__Bacteria	k__norank_d__Bacteria	p__Proteobacteria	c__Gammaproteobacteria	o__Enterobacterales	f__Erwiniaceae	g__unclassified_f__Erwiniaceae	s__unclassified_f__Erwiniaceae	OTU11
d__Bacteria	k__norank_d__Bacteria	p__Proteobacteria	c__Alphaproteobacteria	o__Rhodospirillales	f__norank_o__Rhodospirillales	g__norank_f__norank_o__Rhodospirillales	s__unclassified_g__norank_f__norank_o__Rhodospirillales	OTU579
d__Bacteria	k__norank_d__Bacteria	p__unclassified_k__norank_d__Bacteria	c__unclassified_k__norank_d__Bacteria	o__unclassified_k__norank_d__Bacteria	f__unclassified_k__norank_d__Bacteria	g__unclassified_k__norank_d__Bacteria	s__unclassified_k__norank_d__Bacteria	OTU1390
d__Bacteria	k__norank_d__Bacteria	p__Verrucomicrobiota	c__Chlamydiae	o__Chlamydiales	f__Simkaniaceae	g__norank_f__Simkaniaceae	s__metagenome_g__norank_f__Simkaniaceae	OTU817
d__Bacteria	k__norank_d__Bacteria	p__Chloroflexi	c__Ktedonobacteria	o__C0119	f__norank_o__C0119	g__norank_f__norank_o__C0119	s__uncultured_bacterium_g__norank_f__norank_o__C0119	OTU1286
d__Bacteria	k__norank_d__Bacteria	p__Firmicutes	c__Bacilli	o__Paenibacillales	f__Paenibacillaceae	g__Paenibacillus	s__unclassified_g__Paenibacillus	OTU2456
d__Bacteria	k__norank_d__Bacteria	p__unclassified_k__norank_d__Bacteria	c__unclassified_k__norank_d__Bacteria	o__unclassified_k__norank_d__Bacteria	f__unclassified_k__norank_d__Bacteria	g__unclassified_k__norank_d__Bacteria	s__unclassified_k__norank_d__Bacteria	OTU1004
d__Bacteria	k__norank_d__Bacteria	p__Gemmatimonadota	c__Gemmatimonadetes	o__Gemmatimonadales	f__Gemmatimonadaceae	g__Roseisolibacter	s__uncultured_forest_soil_bacterium_g__Roseisolibacter	OTU1837
d__Bacteria	k__norank_d__Bacteria	p__Cyanobacteria	c__Cyanobacteriia	o__Chloroplast	f__norank_o__Chloroplast	g__norank_f__norank_o__Chloroplast	s__Myrmecia_israeliensis	OTU2885
d__Bacteria	k__norank_d__Bacteria	p__Abditibacteriota	c__Abditibacteria	o__Abditibacteriales	f__Abditibacteriaceae	g__Abditibacterium	s__bacterium_g__Abditibacterium	OTU2848
d__Bacteria	k__norank_d__Bacteria	p__Acidobacteriota	c__Acidobacteriae	o__Subgroup_12	f__norank_o__Subgroup_12	g__norank_f__norank_o__Subgroup_12	s__uncultured_bacterium_g__norank_f__norank_o__Subgroup_12	OTU1127

d__Bacteria	k__norank_ d__Bacteria	p__Proteobacteria	c__Alphaproteobacteria	o__Rhizobiales	f__Hyphomicrobiaceae	g__Hyphomicrobium	s__metagenome_g__Hyphomicrobium	OTU144
d__Bacteria	k__norank_ d__Bacteria	p__Patescibacteria	c__Saccharimonadia	o__Saccharimonadales	f__norank_o__Saccharimonadales	g__norank_f__norank_o__Saccharimo nadales	s__unclassified_g__norank_f__norank_o__Saccharimonadales	OTU2584
d__Bacteria	k__norank_ d__Bacteria	p__Bacteroidota	c__Bacteroidia	o__Chitinophagales	f__Chitinophagaceae	g__norank_f__Chitinophagaceae	s__unclassified_g__norank_f__Chitinophagaceae	OTU1613
d__Bacteria	k__norank_ d__Bacteria	p__Actinobacteriota	c__Actinobacteria	o__Propionibacteriales	f__Propionibacteriaceae	g__Aestuariimicrobium	s__uncultured_bacterium_g__Aestuariimicrobium	OTU2115
d__Bacteria	k__norank_ d__Bacteria	p__Cyanobacteria	c__Cyanobacteriia	o__Chloroplast	f__norank_o__Chloroplast	g__norank_f__norank_o__Chloroplast	s__uncultured_bacterium_g__norank_f__norank_o__Chloroplast	OTU1681
d__Bacteria	k__norank_ d__Bacteria	p__Bacteroidota	c__Kapabacteria	o__Kapabacteriales	f__norank_o__Kapabacteriales	g__norank_f__norank_o__Kapabacteri ales	s__uncultured_bacterium_KF-JG30-B11	OTU894
d__Bacteria	k__norank_ d__Bacteria	p__Acidobacteriota	c__Vicinamibacteria	o__Subgroup_17	f__norank_o__Subgroup_17	g__norank_f__norank_o__Subgroup_1 7	s__uncultured_Acidobacteria_bacterium_g__norank_f__norank_o__Subgroup_17	OTU2961
d__Bacteria	k__norank_ d__Bacteria	p__Proteobacteria	c__Alphaproteobacteria	o__Rickettsiales	f__Rickettsiaceae	g__Rickettsia	s__uncultured_bacterium_g__Rickettsia	OTU1792
d__Bacteria	k__norank_ d__Bacteria	p__Actinobacteriota	c__Thermoleophilia	o__Gaiellales	f__Gaiellaceae	g__Gaiella	s__uncultured_actinobacterium_g__Gaiella	OTU2569
d__Bacteria	k__norank_ d__Bacteria	p__Actinobacteriota	c__Acidimicrobiia	o__Microtrichales	f__Ilumatobacteraceae	g__norank_f__Ilumatobacteraceae	s__unclassified_g__norank_f__Ilumatobacteraceae	OTU134
d__Bacteria	k__norank_ d__Bacteria	p__Chloroflexi	c__OLB14	o__norank_c__OLB14	f__norank_o__norank_c__OLB14	g__norank_f__norank_o__norank_c__ OLB14	s__unclassified_g__norank_f__norank_o__norank_c__OLB14	OTU2177
d__Bacteria	k__norank_ d__Bacteria	p__Armatimonadota	c__Armatimonadia	o__Armatimonadales	f__norank_o__Armatimonadales	g__norank_f__norank_o__Armatimona dales	s__uncultured_eubacterium_WD294	OTU1172
d__Bacteria	k__norank_ d__Bacteria	p__Proteobacteria	c__Alphaproteobacteria	o__norank_c__Alphaproteo bacteria	f__norank_o__norank_c__Alphaproteoba cteria	g__norank_f__norank_o__norank_c__ Alphaproteobacteria	s__uncultured_bacterium_g__norank_f__norank_o__norank_c__Alphaproteobacteri a	OTU623
d__Bacteria	k__norank_ d__Bacteria	p__Proteobacteria	c__Gammaproteobacteria	o__Cellvibrionales	f__Cellvibrionaceae	g__Cellvibrio	s__unclassified_g__Cellvibrio	OTU2390
d__Bacteria	k__norank_ d__Bacteria	p__WPS-2	c__norank_p__WPS-2	o__norank_c__norank_p__ WPS-2	f__norank_o__norank_c__norank_p__W PS-2	g__norank_f__norank_o__norank_c__ norank_p__WPS-2	s__metagenome_g__norank_f__norank_o__norank_c__norank_p__WPS-2	OTU2774
d__Bacteria	k__norank_ d__Bacteria	p__Planctomycetota	c__Planctomycetes	o__Gemmatales	f__Gemmataceae	g__norank_f__Gemmataceae	s__uncultured_planctomycete_g__norank_f__Gemmataceae	OTU1624

d__Bacteria	k__norank_ d__Bacteria	p__Bacteroidota	c__Bacteroidia	o__Cytophagales	f__Spirosomaceae	g__norank_f__Spirosomaceae	s__metagenome_g__norank_f__Spirosomaceae	OTU726
d__Bacteria	k__norank_ d__Bacteria	p__Chloroflexi	c__Anaerolineae	o__unclassified_c__Anaerol ineae	f__unclassified_c__Anaerolineae	g__unclassified_c__Anaerolineae	s__unclassified_c__Anaerolineae	OTU1768
d__Bacteria	k__norank_ d__Bacteria	p__Chloroflexi	c__Chloroflexia	o__Elev-1554	f__norank_o__Elev-1554	g__norank_f__norank_o__Elev-1554	s__uncultured_bacterium_g__norank_f__norank_o__Elev-1554	OTU1236
d__Bacteria	k__norank_ d__Bacteria	p__Bacteroidota	c__Bacteroidia	o__Cytophagales	f__Spirosomaceae	g__Emticicia	s__uncultured_bacterium_g__Emticicia	OTU1365
d__Bacteria	k__norank_ d__Bacteria	p__Chloroflexi	c__KD4-96	o__norank_c__KD4-96	f__norank_o__norank_c__KD4-96	g__norank_f__norank_o__norank_c__ KD4-96	s__uncultured_Chloroflexi_bacterium_g__norank_f__norank_o__norank_c__KD4-9 6	OTU1914
d__Bacteria	k__norank_ d__Bacteria	p__Acidobacteriota	c__Acidobacteriae	o__Acidobacteriales	f__unclassified_o__Acidobacteriales	g__unclassified_o__Acidobacteriales	s__unclassified_o__Acidobacteriales	OTU1184
d__Bacteria	k__norank_ d__Bacteria	p__Myxococcota	c__Polyangia	o__Polyangiales	f__BIrii41	g__norank_f__BIrii41	s__unclassified_g__norank_f__BIrii41	OTU2229
d__Bacteria	k__norank_ d__Bacteria	p__Proteobacteria	c__Gammaproteobacteria	o__Burkholderiales	f__Nitrosomonadaceae	g__Ellin6067	s__uncultured_bacterium_g__Ellin6067	OTU815
d__Bacteria	k__norank_ d__Bacteria	p__Chloroflexi	c__KD4-96	o__norank_c__KD4-96	f__norank_o__norank_c__KD4-96	g__norank_f__norank_o__norank_c__ KD4-96	s__uncultured_bacterium_g__norank_f__norank_o__norank_c__KD4-96	OTU442
d__Bacteria	k__norank_ d__Bacteria	p__Actinobacteriota	c__Actinobacteria	o__Propionibacteriales	f__Propionibacteriaceae	g__Friedmanniella	s__uncultured_bacterium_g__Friedmanniella	OTU151
d__Bacteria	k__norank_ d__Bacteria	p__Bacteroidota	c__Bacteroidia	o__Chitinophagales	f__Chitinophagaceae	g__norank_f__Chitinophagaceae	s__unclassified_g__norank_f__Chitinophagaceae	OTU434
d__Bacteria	k__norank_ d__Bacteria	p__Myxococcota	c__Polyangia	o__Polyangiales	f__Sandaracinaceae	g__norank_f__Sandaracinaceae	s__uncultured_Polyangiaceae_bacterium_g__norank_f__Sandaracinaceae	OTU2110
d__Bacteria	k__norank_ d__Bacteria	p__Proteobacteria	c__Alphaproteobacteria	o__Rhizobiales	f__Devosiaceae	g__Devosia	s__metagenome_g__Devosia	OTU420
d__Bacteria	k__norank_ d__Bacteria	p__Proteobacteria	c__Gammaproteobacteria	o__Steroidobacterales	f__Steroidobacteraceae	g__Steroidobacter	s__unclassified_g__Steroidobacter	OTU2543
d__Bacteria	k__norank_ d__Bacteria	p__Bdellovibrionota	c__Oligoflexia	o__0319-6G20	f__norank_o__0319-6G20	g__norank_f__norank_o__0319-6G20	s__unclassified_g__norank_f__norank_o__0319-6G20	OTU966
d__Bacteria	k__norank_ d__Bacteria	p__Gemmatimonadota	c__Gemmatimonadetes	o__Gemmatimonadales	f__Gemmatimonadaceae	g__unclassified_f__Gemmatimonadace ae	s__unclassified_f__Gemmatimonadaceae	OTU248

d__Bacteria	k__norank_ d__Bacteria	p__Gemmatimonadota	c__Gemmatimonadetes	o__Gemmatimonadales	f__Gemmatimonadaceae	g__Gemmatimonas	s__unclassified_g__Gemmatimonas	OTU1824
d__Bacteria	k__norank_ d__Bacteria	p__Bdellovibrionota	c__Oligoflexia	o__0319-6G20	f__norank_o__0319-6G20	g__norank_f__norank_o__0319-6G20	s__marine_metagenome_g__norank_f__norank_o__0319-6G20	OTU941
d__Bacteria	k__norank_ d__Bacteria	p__Bdellovibrionota	c__Oligoflexia	o__0319-6G20	f__norank_o__0319-6G20	g__norank_f__norank_o__0319-6G20	s__metagenome_g__norank_f__norank_o__0319-6G20	OTU1246
d__Bacteria	k__norank_ d__Bacteria	p__Actinobacteriota	c__Thermoleophilia	o__Solirubrobacterales	f__67-14	g__norank_f__67-14	s__uncultured_bacterium_g__norank_f__67-14	OTU435
d__Bacteria	k__norank_ d__Bacteria	p__Gemmatimonadota	c__Gemmatimonadetes	o__Gemmatimonadales	f__Gemmatimonadaceae	g__Gemmatimonas	s__uncultured_bacterium_g__Gemmatimonas	OTU2009
d__Bacteria	k__norank_ d__Bacteria	p__Chloroflexi	c__Ktedonobacteria	o__Ktedonobacterales	f__Ktedonobacteraceae	g__1921-2	s__uncultured_bacterium_g__1921-2	OTU1744
d__Bacteria	k__norank_ d__Bacteria	p__Acidobacteriota	c__Blastocatellia	o__DS-100	f__norank_o__DS-100	g__norank_f__norank_o__DS-100	s__unclassified_g__norank_f__norank_o__DS-100	OTU387
d__Bacteria	k__norank_ d__Bacteria	p__Myxococcota	c__Polyangia	o__Haliangiales	f__Haliangiaceae	g__Haliangium	s__unclassified_g__Haliangium	OTU2686
d__Bacteria	k__norank_ d__Bacteria	p__Patescibacteria	c__Saccharimonadia	o__Saccharimonadales	f__LWQ8	g__norank_f__LWQ8	s__metagenome_g__norank_f__LWQ8	OTU953
d__Bacteria	k__norank_ d__Bacteria	p__Actinobacteriota	c__Acidimicrobiia	o__Microtrichales	f__Microtrichaceae	g__norank_f__Microtrichaceae	s__metagenome_g__norank_f__Microtrichaceae	OTU2165
d__Bacteria	k__norank_ d__Bacteria	p__Gemmatimonadota	c__Longimicrobia	o__Longimicrobiales	f__Longimicrobiaceae	g__norank_f__Longimicrobiaceae	s__uncultured_bacterium_g__norank_f__Longimicrobiaceae	OTU2832
d__Bacteria	k__norank_ d__Bacteria	p__Proteobacteria	c__Gammaproteobacteria	o__Gammaproteobacteria_I ncertae_Sedis	f__unclassified_o__Gammaproteobacteri a_Incertae_Sedis	g__Acidibacter	s__unclassified_g__Acidibacter	OTU1322
d__Bacteria	k__norank_ d__Bacteria	p__Myxococcota	c__Polyangia	o__Polyangiales	f__Phaselicystidaceae	g__Phaselicystis	s__metagenome_g__Phaselicystis	OTU1623
d__Bacteria	k__norank_ d__Bacteria	p__Planctomycetota	c__Planctomycetes	o__Gemmatales	f__Gemmataceae	g__norank_f__Gemmataceae	s__unclassified_g__norank_f__Gemmataceae	OTU2496
d__Bacteria	k__norank_ d__Bacteria	p__Actinobacteriota	c__Actinobacteria	o__unclassified_c__Actinob acteria	f__unclassified_c__Actinobacteria	g__unclassified_c__Actinobacteria	s__unclassified_c__Actinobacteria	OTU1491
d__Bacteria	k__norank_ d__Bacteria	p__Chloroflexi	c__unclassified_p__Chlor oflexi	o__unclassified_p__Chlorofl exi	f__unclassified_p__Chloroflexi	g__unclassified_p__Chloroflexi	s__unclassified_p__Chloroflexi	OTU1604

d__Bacteria	k__norank_ d__Bacteria	p__Proteobacteria	c__Alphaproteobacteria	o__Acetobacterales	f__Acetobacteraceae	g__unclassified_f__Acetobacteraceae	s__unclassified_f__Acetobacteraceae	OTU2858
d__Bacteria	k__norank_ d__Bacteria	p__unclassified_k__no rank_d__Bacteria	c__unclassified_k__noran k_d__Bacteria	o__unclassified_k__norank_ d__Bacteria	f__unclassified_k__norank_d__Bacteria	g__unclassified_k__norank_d__Bacteri a	s__unclassified_k__norank_d__Bacteria	OTU2844
d__Bacteria	k__norank_ d__Bacteria	p__Proteobacteria	c__Alphaproteobacteria	o__Caulobacterales	f__Caulobacteraceae	g__PMMR1	s__uncultured_bacterium_g__PMMR1	OTU2949
d__Bacteria	k__norank_ d__Bacteria	p__Gemmatimonadota	c__Gemmatimonadetes	o__Gemmatimonadales	f__Gemmatimonadaceae	g__Gemmatimonas	s__unclassified_g__Gemmatimonas	OTU938
d__Bacteria	k__norank_ d__Bacteria	p__Dependentiae	c__Babeliae	o__Babeliales	f__norank_o__Babeliales	g__norank_f__norank_o__Babeliales	s__uncultured_Candidatus_Dependentiae_bacterium_g__norank_f__norank	OTU1633
d__Bacteria	k__norank_ d__Bacteria	p__Bacteroidota	c__Bacteroidia	o__Chitinophagales	f__Chitinophagaceae	g__Cnuella	s__uncultured_bacterium_g__Cnuella	OTU228
d__Bacteria	k__norank_ d__Bacteria	p__Bacteroidota	c__Bacteroidia	o__Chitinophagales	f__Chitinophagaceae	g__Ferruginibacter	s__uncultured_bacterium_g__Ferruginibacter	OTU1048
d__Bacteria	k__norank_ d__Bacteria	p__Gemmatimonadota	c__Gemmatimonadetes	o__Gemmatimonadales	f__Gemmatimonadaceae	g__unclassified_f__Gemmatimonadace ae	s__unclassified_f__Gemmatimonadaceae	OTU2526
d__Bacteria	k__norank_ d__Bacteria	p__Proteobacteria	c__Gammaproteobacteria	o__Xanthomonadales	f__Rhodanobacteraceae	g__Ahniella	s__uncultured_bacterium_g__Ahniella	OTU2469
d__Bacteria	k__norank_ d__Bacteria	p__Acidobacteriota	c__Acidobacteriae	o__Acidobacteriales	f__Acidobacteriaceae_Subgroup_1	g__Occallatibacter	s__unclassified_g__Occallatibacter	OTU1130
d__Bacteria	k__norank_ d__Bacteria	p__Bdellovibrionota	c__Bdellovibrionia	o__Bdellovibrionales	f__Bdellovibrionaceae	g__Bdellovibrio	s__uncultured_bacterium_g__Bdellovibrio	OTU1665
d__Bacteria	k__norank_ d__Bacteria	p__Proteobacteria	c__Alphaproteobacteria	o__Acetobacterales	f__Acetobacteraceae	g__norank_f__Acetobacteraceae	s__metagenome_g__norank_f__Acetobacteraceae	OTU1850
d__Bacteria	k__norank_ d__Bacteria	p__Proteobacteria	c__Gammaproteobacteria	o__Salinisphaerales	f__Solimonadaceae	g__Polycyclovorans	s__uncultured_bacterium_g__Polycyclovorans	OTU788
d__Bacteria	k__norank_ d__Bacteria	p__Bdellovibrionota	c__Oligoflexia	o__0319-6G20	f__norank_o__0319-6G20	g__norank_f__norank_o__0319-6G20	s__uncultured_bacterium_g__norank_f__norank_o__0319-6G20	OTU2652
d__Bacteria	k__norank_ d__Bacteria	p__Acidobacteriota	c__Acidobacteriae	o__Subgroup_2	f__norank_o__Subgroup_2	g__norank_f__norank_o__Subgroup_2	s__uncultured_Acidobacteria_bacterium_g__norank_f__norank_o__Subgroup_2	OTU1548
d__Bacteria	k__norank_ d__Bacteria	p__Chloroflexi	c__Chloroflexia	o__Chloroflexales	f__Chloroflexaceae	g__FFCH7168	s__uncultured_bacterium_g__FFCH7168	OTU2254

d__Bacteria	k__norank_ d__Bacteria	p__Actinobacteriota	c__Thermoleophilia	o__Solirubrobacterales	f__Solirubrobacteraceae	g__Patulibacter	s__Patulibacter_americanus	OTU251
d__Bacteria	k__norank_ d__Bacteria	p__Actinobacteriota	c__Acidimicrobiia	o__Microtrichales	f__Iamiaceae	g__Iamia	s__uncultured_bacterium_g__Iamia	OTU1420
d__Bacteria	k__norank_ d__Bacteria	p__Cyanobacteria	c__Cyanobacteriia	o__Chloroplast	f__norank_o__Chloroplast	g__norank_f__norank_o__Chloroplast	s__unclassified_g__norank_f__norank_o__Chloroplast	OTU2978
d__Bacteria	k__norank_ d__Bacteria	p__Verrucomicrobiota	c__Verrucomicrobiae	o__Pedosphaerales	f__Pedosphaeraceae	g__unclassified_f__Pedosphaeraceae	s__unclassified_f__Pedosphaeraceae	OTU1209
d__Bacteria	k__norank_ d__Bacteria	p__Proteobacteria	c__Gammaproteobacteria	o__Burkholderiales	f__Sutterellaceae	g__AAP99	s__metagenome_g__AAP99	OTU2237
d__Bacteria	k__norank_ d__Bacteria	p__Actinobacteriota	c__Actinobacteria	o__Micrococcales	f__Microbacteriaceae	g__Microterricola	s__Microterricola_viridarii	OTU1691
d__Bacteria	k__norank_ d__Bacteria	p__Verrucomicrobiota	c__Verrucomicrobiae	o__Chthoniobacterales	f__Chthoniobacteraceae	g__Candidatus_Udaeobacter	s__unclassified_g__Candidatus_Udaeobacter	OTU1046
d__Bacteria	k__norank_ d__Bacteria	p__Proteobacteria	c__Gammaproteobacteria	o__Burkholderiales	f__SC-I-84	g__norank_f__SC-I-84	s__unclassified_g__norank_f__SC-I-84	OTU2540
d__Bacteria	k__norank_ d__Bacteria	p__Proteobacteria	c__Alphaproteobacteria	o__Rhizobiales	f__Xanthobacteraceae	g__Rhodoplanes	s__unclassified_g__Rhodoplanes	OTU2144
d__Bacteria	k__norank_ d__Bacteria	p__Actinobacteriota	c__Actinobacteria	o__Propionibacteriales	f__Nocardioidaceae	g__Aeromicrobium	s__uncultured_bacterium_g__Aeromicrobium	OTU219
d__Bacteria	k__norank_ d__Bacteria	p__Chloroflexi	c__Ktedonobacteria	o__Ktedonobacterales	f__Ktedonobacteraceae	g__FCPS473	s__uncultured_bacterium_g__FCPS473	OTU1397
d__Bacteria	k__norank_ d__Bacteria	p__Proteobacteria	c__Gammaproteobacteria	o__Xanthomonadales	f__Rhodanobacteraceae	g__Ahniella	s__uncultured_bacterium_g__Ahniella	OTU386
d__Bacteria	k__norank_ d__Bacteria	p__Myxococcota	c__Polyangia	o__Haliangiales	f__Haliangiaceae	g__Haliangium	s__unclassified_g__Haliangium	OTU1932
d__Bacteria	k__norank_ d__Bacteria	p__Planctomycetota	c__Planctomycetes	o__Gemmatales	f__Gemmataceae	g__norank_f__Gemmataceae	s__unclassified_g__norank_f__Gemmataceae	OTU1599
d__Bacteria	k__norank_ d__Bacteria	p__Proteobacteria	c__Gammaproteobacteria	o__Pseudomonadales	f__Pseudomonadaceae	g__Pseudomonas	s__Pseudomonas_umsongensis	OTU566
d__Bacteria	k__norank_ d__Bacteria	p__Actinobacteriota	c__Actinobacteria	o__Streptomycetales	f__Streptomycetaceae	g__unclassified_f__Streptomycetaceae	s__unclassified_f__Streptomycetaceae	OTU1038

d__Bacteria	k__norank_d__Bacteria	p__Acidobacteriota	c__Vicinamibacteria	o__Vicinamibacterales	f__norank_o__Vicinamibacterales	g__norank_f__norank_o__Vicinamibacterales	s__uncultured_Acidobacteriaceae_bacterium_g__norank_f__norank_o__Vicinamibacterales	OTU1402
d__Bacteria	k__norank_d__Bacteria	p__Verrucomicrobiota	c__Verrucomicrobiae	o__Chthoniobacterales	f__Chthoniobacteraceae	g__Candidatus_Udaeobacter	s__uncultured_Verrucomicrobia_bacterium_g__Candidatus_Udaeobacter	OTU1942
d__Bacteria	k__norank_d__Bacteria	p__Bacteroidota	c__Bacteroidia	o__Cytophagales	f__Microscillaceae	g__norank_f__Microscillaceae	s__unclassified_g__norank_f__Microscillaceae	OTU2630
d__Bacteria	k__norank_d__Bacteria	p__Acidobacteriota	c__Acidobacteriae	o__Bryobacterales	f__Bryobacteraceae	g__Bryobacter	s__unclassified_g__Bryobacter	OTU1750
d__Bacteria	k__norank_d__Bacteria	p__Firmicutes	c__Bacilli	o__Lactobacillales	f__Lactobacillaceae	g__Pediococcus	s__Pediococcus_pentosaceus_g__Pediococcus	OTU192
d__Bacteria	k__norank_d__Bacteria	p__Proteobacteria	c__Alphaproteobacteria	o__Reyranellales	f__Reyranellaceae	g__Reyranella	s__unclassified_g__Reyranella	OTU841
d__Bacteria	k__norank_d__Bacteria	p__Actinobacteriota	c__Actinobacteria	o__Propionibacteriales	f__Propionibacteriaceae	g__Propioniciclava	s__uncultured_bacterium_g__Propioniciclava	OTU2119
d__Bacteria	k__norank_d__Bacteria	p__Acidobacteriota	c__Acidobacteriae	o__Elev-16S-1166	f__norank_o__Elev-16S-1166	g__norank_f__norank_o__Elev-16S-1166	s__uncultured_bacterium_g__norank_f__norank_o__Elev-16S-1166	OTU1058
d__Bacteria	k__norank_d__Bacteria	p__Proteobacteria	c__Gammaproteobacteria	o__Legionellales	f__Legionellaceae	g__Legionella	s__uncultured_bacterium_g__Legionella	OTU344
d__Bacteria	k__norank_d__Bacteria	p__Proteobacteria	c__Gammaproteobacteria	o__Legionellales	f__Legionellaceae	g__Legionella	s__uncultured_bacterium_g__Legionella	OTU342
d__Bacteria	k__norank_d__Bacteria	p__Bacteroidota	c__Bacteroidia	o__Cytophagales	f__norank_o__Cytophagales	g__norank_f__norank_o__Cytophagales	s__unclassified_g__norank_f__norank_o__Cytophagales	OTU1135
d__Bacteria	k__norank_d__Bacteria	p__Cyanobacteria	c__Sericytochromatia	o__norank_c__Sericytochromatia	f__norank_o__norank_c__Sericytochromatia	g__norank_f__norank_o__norank_c__Sericytochromatia	s__unclassified_g__norank_f__norank_o__norank_c__Sericytochromatia	OTU2306
d__Bacteria	k__norank_d__Bacteria	p__Bacteroidota	c__Bacteroidia	o__Cytophagales	f__Hymenobacteraceae	g__Adhaeribacter	s__unclassified_g__Adhaeribacter	OTU1382
d__Bacteria	k__norank_d__Bacteria	p__Bacteroidota	c__Bacteroidia	o__Flavobacteriales	f__NS9_marine_group	g__norank_f__NS9_marine_group	s__metagenome_g__norank_f__NS9_marine_group	OTU400
d__Bacteria	k__norank_d__Bacteria	p__Proteobacteria	c__Gammaproteobacteria	o__Burkholderiales	f__TRA3-20	g__norank_f__TRA3-20	s__unclassified_g__norank_f__TRA3-20	OTU1609
d__Bacteria	k__norank_d__Bacteria	p__Myxococcota	c__Polyangia	o__Polyangiales	f__Polyangiaceae	g__Pajaroellobacter	s__unclassified_g__Pajaroellobacter	OTU1827

d__Bacteria	k__norank_ d__Bacteria	p__Proteobacteria	c__Gammaproteobacteria	o__Pseudomonadales	f__Pseudomonadaceae	g__Pseudomonas	s__unclassified_g__Pseudomonas	OTU480
d__Bacteria	k__norank_ d__Bacteria	p__Acidobacteriota	c__Acidobacteriae	o__Bryobacterales	f__Bryobacteraceae	g__Bryobacter	s__Bryobacter_aggregatus	OTU1144
d__Bacteria	k__norank_ d__Bacteria	p__Cyanobacteria	c__Cyanobacteriia	o__Chloroplast	f__norank_o__Chloroplast	g__norank_f__norank_o__Chloroplast	s__Tetradesmus_obliquus	OTU1561
d__Bacteria	k__norank_ d__Bacteria	p__Actinobacteriota	c__Acidimicrobiia	o__norank_c__Acidimicrobi ia	f__norank_o__norank_c__Acidimicrobiia	g__norank_f__norank_o__norank_c__ Acidimicrobiia	s__uncultured_bacterium_g__norank_f__norank_o__norank_c__Acidimicrobiia	OTU356
d__Bacteria	k__norank_ d__Bacteria	p__Bdellovibrionota	c__Bdellovibrionia	o__Bdellovibrionales	f__Bdellovibrionaceae	g__Bdellovibrio	s__unclassified_g__Bdellovibrio	OTU2817
d__Bacteria	k__norank_ d__Bacteria	p__Actinobacteriota	c__Actinobacteria	o__Corynebacteriales	f__Mycobacteriaceae	g__Mycobacterium	s__unclassified_g__Mycobacterium	OTU1534
d__Bacteria	k__norank_ d__Bacteria	p__Actinobacteriota	c__Thermoleophilia	o__Solirubrobacterales	f__Solirubrobacteraceae	g__norank_f__Solirubrobacteraceae	s__metagenome_g__norank_f__Solirubrobacteraceae	OTU2423
d__Bacteria	k__norank_ d__Bacteria	p__Abditibacteriota	c__Abditibacteria	o__Abditibacteriales	f__Abditibacteriaceae	g__Abditibacterium	s__metagenome_g__Abditibacterium	OTU2839
d__Bacteria	k__norank_ d__Bacteria	p__Acidobacteriota	c__Holophagae	o__Subgroup_7	f__norank_o__Subgroup_7	g__norank_f__norank_o__Subgroup_7	s__uncultured_Acidobacteria_bacterium_g__norank_f__norank_o__Subgroup_7	OTU1629
d__Bacteria	k__norank_ d__Bacteria	p__Acidobacteriota	c__Acidobacteriae	o__Bryobacterales	f__Bryobacteraceae	g__Bryobacter	s__unclassified_g__Bryobacter	OTU1779
d__Bacteria	k__norank_ d__Bacteria	p__Proteobacteria	c__Alphaproteobacteria	o__Acetobacterales	f__Acetobacteraceae	g__Rhodovastum	s__uncultured_bacterium_g__Rhodovastum	OTU130
d__Bacteria	k__norank_ d__Bacteria	p__Chloroflexi	c__Chloroflexia	o__Chloroflexales	f__Roseiflexaceae	g__norank_f__Roseiflexaceae	s__uncultured_bacterium_g__norank_f__Roseiflexaceae	OTU1639
d__Bacteria	k__norank_ d__Bacteria	p__Proteobacteria	c__Gammaproteobacteria	o__Burkholderiales	f__Methylophilaceae	g__norank_f__Methylophilaceae	s__metagenome_g__norank_f__Methylophilaceae	OTU2061
d__Bacteria	k__norank_ d__Bacteria	p__Proteobacteria	c__Alphaproteobacteria	o__Rhizobiales	f__Hyphomicrobiaceae	g__Pedomicrobium	s__metagenome_g__Pedomicrobium	OTU2583
d__Bacteria	k__norank_ d__Bacteria	p__Bacteroidota	c__Bacteroidia	o__Cytophagales	f__Microscillaceae	g__norank_f__Microscillaceae	s__Bacteroidetes_bacterium_CHC2	OTU2112
d__Bacteria	k__norank_ d__Bacteria	p__Actinobacteriota	c__Actinobacteria	o__Propionibacteriales	f__Propionibacteriaceae	g__Cutibacterium	s__Cutibacterium_acnes_g__Cutibacterium	OTU2853

d__Bacteria	k__norank_ d__Bacteria	p__Cyanobacteria	c__Cyanobacteriia	o__Chloroplast	f__norank_o__Chloroplast	g__norank_f__norank_o__Chloroplast	s__unclassified_g__norank_f__norank_o__Chloroplast	OTU2081
d__Bacteria	k__norank_ d__Bacteria	p__Acidobacteriota	c__Blastocatellia	o__Blastocatellales	f__Blastocatellaceae	g__Blastocatella	s__uncultured_soil_bacterium_g__Blastocatella	OTU2265
d__Bacteria	k__norank_ d__Bacteria	p__Acidobacteriota	c__Vicinamibacteria	o__Vicinamibacterales	f__Vicinamibacteraceae	g__Luteitalea	s__uncultured_bacterium_g__Luteitalea	OTU2926
d__Bacteria	k__norank_ d__Bacteria	p__Bdellovibrionota	c__Bdellovibrionia	o__Bdellovibrionales	f__Bdellovibrionaceae	g__Bdellovibrio	s__unclassified_g__Bdellovibrio	OTU55
d__Bacteria	k__norank_ d__Bacteria	p__Planctomycetota	c__Planctomycetes	o__Gemmatales	f__Gemmataceae	g__norank_f__Gemmataceae	s__uncultured_bacterium_g__norank_f__Gemmataceae	OTU1499
d__Bacteria	k__norank_ d__Bacteria	p__Actinobacteriota	c__Acidimicrobiia	o__norank_c__Acidimicrobi ia	f__norank_o__norank_c__Acidimicrobiia	g__norank_f__norank_o__norank_c__ Acidimicrobiia	s__unclassified_g__norank_f__norank_o__norank_c__Acidimicrobiia	OTU1843
d__Bacteria	k__norank_ d__Bacteria	p__Chloroflexi	c__Anaerolineae	o__Caldilineales	f__Caldilineaceae	g__norank_f__Caldilineaceae	s__uncultured_bacterium_g__norank_f__Caldilineaceae	OTU2431
d__Bacteria	k__norank_ d__Bacteria	p__Bdellovibrionota	c__Oligoflexia	o__0319-6G20	f__norank_o__0319-6G20	g__norank_f__norank_o__0319-6G20	s__unclassified_g__norank_f__norank_o__0319-6G20	OTU992
d__Bacteria	k__norank_ d__Bacteria	p__Chloroflexi	c__Chloroflexia	o__Chloroflexales	f__Roseiflexaceae	g__norank_f__Roseiflexaceae	s__uncultured_soil_bacterium_g__norank_f__Roseiflexaceae	OTU1309
d__Bacteria	k__norank_ d__Bacteria	p__Bacteroidota	c__Bacteroidia	o__Sphingobacteriales	f__Sphingobacteriaceae	g__Mucilaginibacter	s__unclassified_g__Mucilaginibacter	OTU1995
d__Bacteria	k__norank_ d__Bacteria	p__Patescibacteria	c__Saccharimonadia	o__Saccharimonadales	f__norank_o__Saccharimonadales	g__norank_f__norank_o__Saccharimo nadales	s__unclassified_g__norank_f__norank_o__Saccharimonadales	OTU2048
d__Bacteria	k__norank_ d__Bacteria	p__Gemmatimonadota	c__Gemmatimonadetes	o__Gemmatimonadales	f__Gemmatimonadaceae	g__Gemmatimonas	s__unclassified_g__Gemmatimonas	OTU1482
d__Bacteria	k__norank_ d__Bacteria	p__Bacteroidota	c__Rhodothermia	o__Rhodothermales	f__Rhodothermaceae	g__norank_f__Rhodothermaceae	s__uncultured_bacterium_g__norank_f__Rhodothermaceae	OTU2175
d__Bacteria	k__norank_ d__Bacteria	p__Actinobacteriota	c__Actinobacteria	o__Micrococcales	f__Micrococcaceae	g__Arthrobacter	s__Arthrobacter_agilis_g__Arthrobacter	OTU2079
d__Bacteria	k__norank_ d__Bacteria	p__Planctomycetota	c__Planctomycetes	o__Isosphaerales	f__Isosphaeraceae	g__norank_f__Isosphaeraceae	s__uncultured_bacterium_g__norank_f__Isosphaeraceae	OTU2854
d__Bacteria	k__norank_ d__Bacteria	p__Actinobacteriota	c__Actinobacteria	o__Micrococcales	f__Dermatophilaceae	g__Austwickia	s__Austwickia_chelonae	OTU2363

d__Bacteria	k__norank_ d__Bacteria	p__Proteobacteria	c__Alphaproteobacteria	o__Rhizobiales	f__Pleomorphomonadaceae	g__unclassified_f__Pleomorphomonad aceae	s__unclassified_f__Pleomorphomonadaceae	OTU2600
d__Bacteria	k__norank_ d__Bacteria	p__Chloroflexi	c__Ktedonobacteria	o__C0119	f__norank_o__C0119	g__norank_f__norank_o__C0119	s__uncultured_Chloroflexi_bacterium_g__norank_f__norank_o__C0119	OTU1806
d__Bacteria	k__norank_ d__Bacteria	p__Actinobacteriota	c__Actinobacteria	o__Micrococcales	f__Micrococcaceae	g__Rothia	s__Rothia_mucilaginosa	OTU571
d__Bacteria	k__norank_ d__Bacteria	p__Proteobacteria	c__Alphaproteobacteria	o__Rhizobiales	f__Hyphomicrobiaceae	g__Pedomicrobium	s__uncultured_bacterium_g__Pedomicrobium	OTU227
d__Bacteria	k__norank_ d__Bacteria	p__Chloroflexi	c__JG30-KF-CM66	o__norank_c__JG30-KF-C M66	f__norank_o__norank_c__JG30-KF-CM 66	g__norank_f__norank_o__norank_c__ JG30-KF-CM66	s__uncultured_Caldilinea_sp._g__norank	OTU1727
d__Bacteria	k__norank_ d__Bacteria	p__Planctomycetota	c__Planctomycetes	o__Isosphaerales	f__Isosphaeraceae	g__norank_f__Isosphaeraceae	s__unclassified_g__norank_f__Isosphaeraceae	OTU2975
d__Bacteria	k__norank_ d__Bacteria	p__Myxococcota	c__Polyangia	o__Polyangiales	f__Sandaracinaceae	g__norank_f__Sandaracinaceae	s__uncultured_soil_bacterium_g__norank_f__Sandaracinaceae	OTU2489
d__Bacteria	k__norank_ d__Bacteria	p__Bacteroidota	c__Bacteroidia	o__Sphingobacteriales	f__Sphingobacteriaceae	g__norank_f__Sphingobacteriaceae	s__unclassified_g__norank_f__Sphingobacteriaceae	OTU1602
d__Bacteria	k__norank_ d__Bacteria	p__Proteobacteria	c__Gammaproteobacteria	o__Burkholderiales	f__Comamonadaceae	g__Rhizobacter	s__uncultured_bacterium_g__Rhizobacter	OTU2397
d__Bacteria	k__norank_ d__Bacteria	p__Acidobacteriota	c__Acidobacteriae	o__Acidobacteriales	f__unclassified_o__Acidobacteriales	g__unclassified_o__Acidobacteriales	s__unclassified_o__Acidobacteriales	OTU1047
d__Bacteria	k__norank_ d__Bacteria	p__Gemmatimonadota	c__Gemmatimonadetes	o__Gemmatimonadales	f__Gemmatimonadaceae	g__norank_f__Gemmatimonadaceae	s__Gemmatimonadetes_bacterium_SCN_70-22	OTU2633
d__Bacteria	k__norank_ d__Bacteria	p__Proteobacteria	c__Gammaproteobacteria	o__Burkholderiales	f__Burkholderiaceae	g__Limnobacter	s__unclassified_g__Limnobacter	OTU2493
d__Bacteria	k__norank_ d__Bacteria	p__Acidobacteriota	c__Blastocatellia	o__11-24	f__norank_o__11-24	g__norank_f__norank_o__11-24	s__uncultured_Acidobacteria_bacterium_g__norank_f__norank_o__11-24	OTU1649
d__Bacteria	k__norank_ d__Bacteria	p__Bacteroidota	c__Bacteroidia	o__Chitinophagales	f__Saprospiraceae	g__Haliscomenobacter	s__unclassified_g__Haliscomenobacter	OTU513
d__Bacteria	k__norank_ d__Bacteria	p__Planctomycetota	c__Planctomycetes	o__Gemmatales	f__Gemmataceae	g__norank_f__Gemmataceae	s__uncultured_bacterium_g__norank_f__Gemmataceae	OTU768
d__Bacteria	k__norank_ d__Bacteria	p__Bdellovibrionota	c__Oligoflexia	o__Oligoflexales	f__norank_o__Oligoflexales	g__Oligoflexus	s__metagenome_g__Oligoflexus	OTU2173

d__Bacteria	k__norank_d__Bacteria	p__Actinobacteriota	c__Thermoleophilia	o__Solirubrobacterales	f__Solirubrobacteraceae	g__Conexibacter	s__unclassified_g__Conexibacter	OTU2442
d__Bacteria	k__norank_d__Bacteria	p__Chloroflexi	c__Chloroflexia	o__Kallotenuales	f__AKIW781	g__norank_f__AKIW781	s__uncultured_bacterium_g__norank_f__AKIW781	OTU1986
d__Bacteria	k__norank_d__Bacteria	p__Gemmatimonadota	c__Gemmatimonadetes	o__Gemmatimonadales	f__Gemmatimonadaceae	g__unclassified_f__Gemmatimonadaceae	s__unclassified_f__Gemmatimonadaceae	OTU2525
d__Bacteria	k__norank_d__Bacteria	p__Actinobacteriota	c__Thermoleophilia	o__Solirubrobacterales	f__Solirubrobacteraceae	g__Conexibacter	s__metagenome_g__Conexibacter	OTU1328
d__Bacteria	k__norank_d__Bacteria	p__unclassified_k__norank_d__Bacteria	c__unclassified_k__norank_d__Bacteria	o__unclassified_k__norank_d__Bacteria	f__unclassified_k__norank_d__Bacteria	g__unclassified_k__norank_d__Bacteria	s__unclassified_k__norank_d__Bacteria	OTU998
d__Bacteria	k__norank_d__Bacteria	p__Acidobacteriota	c__Vicinamibacteria	o__Vicinamibacterales	f__norank_o__Vicinamibacterales	g__norank_f__norank_o__Vicinamibacterales	s__unclassified_g__norank_f__norank_o__Vicinamibacterales	OTU1581
d__Bacteria	k__norank_d__Bacteria	p__Bdellovibrionota	c__Bdellovibrionia	o__Bacteriovoracales	f__Bacteriovoracaceae	g__Peredibacter	s__microbial_mat_metagenome_g__Peredibacter	OTU181
d__Bacteria	k__norank_d__Bacteria	p__Methylomirabilota	c__Methylomirabilia	o__Rokubacteriales	f__norank_o__Rokubacteriales	g__norank_f__norank_o__Rokubacteriales	s__uncultured_bacterium_g__norank_f__norank_o__Rokubacteriales	OTU2868
d__Bacteria	k__norank_d__Bacteria	p__Myxococcota	c__Myxococcia	o__Myxococcales	f__Myxococcaceae	g__P3OB-42	s__uncultured_bacterium_g__P3OB-42	OTU47
d__Bacteria	k__norank_d__Bacteria	p__Actinobacteriota	c__Actinobacteria	o__Propionibacteriales	f__Nocardioidaceae	g__Nocardioides	s__Nocardioides_sp._g__Nocardioides	OTU2153
d__Bacteria	k__norank_d__Bacteria	p__Chloroflexi	c__Ktedonobacteria	o__C0119	f__norank_o__C0119	g__norank_f__norank_o__C0119	s__uncultured_Chloroflexi_bacterium_g__norank_f__norank_o__C0119	OTU1345
d__Bacteria	k__norank_d__Bacteria	p__Bacteroidota	c__Kryptonia	o__Kryptoniales	f__BSV26	g__norank_f__BSV26	s__uncultured_bacterium_g__norank_f__BSV26	OTU2148
d__Bacteria	k__norank_d__Bacteria	p__Proteobacteria	c__Gammaproteobacteria	o__Legionellales	f__Legionellaceae	g__Legionella	s__uncultured_bacterium_g__Legionella	OTU751
d__Bacteria	k__norank_d__Bacteria	p__Actinobacteriota	c__Actinobacteria	o__Corynebacteriales	f__Mycobacteriaceae	g__Mycobacterium	s__metagenome_g__Mycobacterium	OTU2451
d__Bacteria	k__norank_d__Bacteria	p__Chloroflexi	c__Anaerolineae	o__Caldilineales	f__Caldilineaceae	g__norank_f__Caldilineaceae	s__uncultured_Chloroflexia_bacterium_g__norank	OTU2872
d__Bacteria	k__norank_d__Bacteria	p__Acidobacteriota	c__Vicinamibacteria	o__Vicinamibacterales	f__norank_o__Vicinamibacterales	g__norank_f__norank_o__Vicinamibacterales	s__unclassified_g__norank_f__norank_o__Vicinamibacterales	OTU2579

d__Bacteria	k__norank_d__Bacteria	p__Cyanobacteria	c__Cyanobacteriia	o__Chloroplast	f__norank_o__Chloroplast	g__norank_f__norank_o__Chloroplast	s__Bryum_argenteum_var._argenteum	OTU945
d__Bacteria	k__norank_d__Bacteria	p__Proteobacteria	c__Alphaproteobacteria	o__Rhizobiales	f__Beijerinckiaceae	g__Bosea	s__unclassified_g__Bosea	OTU2615
d__Bacteria	k__norank_d__Bacteria	p__Proteobacteria	c__Alphaproteobacteria	o__Rhizobiales	f__Xanthobacteraceae	g__Tardiphaga	s__unclassified_g__Tardiphaga	OTU1149
d__Bacteria	k__norank_d__Bacteria	p__Acidobacteriota	c__Blastocatellia	o__Blastocatellales	f__Blastocatellaceae	g__Blastocatella	s__unclassified_g__Blastocatella	OTU1207
d__Bacteria	k__norank_d__Bacteria	p__Gemmatimonadota	c__Gemmatimonadetes	o__Gemmatimonadales	f__Gemmatimonadaceae	g__Gemmatimonas	s__unclassified_g__Gemmatimonas	OTU358
d__Bacteria	k__norank_d__Bacteria	p__Proteobacteria	c__Alphaproteobacteria	o__Rhizobiales	f__Rhizobiales_Incertae_Sedis	g__Phreatobacter	s__uncultured_bacterium_g__Phreatobacter	OTU578
d__Bacteria	k__norank_d__Bacteria	p__Actinobacteriota	c__Thermoleophilia	o__Gaiellales	f__norank_o__Gaiellales	g__norank_f__norank_o__Gaiellales	s__uncultured_bacterium_g__norank_f__norank_o__Gaiellales	OTU756
d__Bacteria	k__norank_d__Bacteria	p__Proteobacteria	c__Gammaproteobacteria	o__Diplorickettsiales	f__Diplorickettsiaceae	g__Aquicella	s__unclassified_g__Aquicella	OTU88
d__Bacteria	k__norank_d__Bacteria	p__Proteobacteria	c__Alphaproteobacteria	o__Caulobacterales	f__Caulobacteraceae	g__norank_f__Caulobacteraceae	s__uncultured_bacterium_g__norank_f__Caulobacteraceae	OTU810
d__Bacteria	k__norank_d__Bacteria	p__Acidobacteriota	c__Thermoanaerobaculia	o__Thermoanaerobaculales	f__Thermoanaerobaculaceae	g__Subgroup_10	s__unclassified_g__Subgroup_10	OTU2477
d__Bacteria	k__norank_d__Bacteria	p__Chloroflexi	c__Chloroflexia	o__Chloroflexales	f__Herpetosiphonaceae	g__Herpetosiphon	s__uncultured_bacterium_g__Herpetosiphon	OTU2277
d__Bacteria	k__norank_d__Bacteria	p__Actinobacteriota	c__Thermoleophilia	o__Solirubrobacterales	f__67-14	g__norank_f__67-14	s__metagenome_g__norank_f__67-14	OTU2080
d__Bacteria	k__norank_d__Bacteria	p__Gemmatimonadota	c__Gemmatimonadetes	o__Gemmatimonadales	f__Gemmatimonadaceae	g__Gemmatimonas	s__unclassified_g__Gemmatimonas	OTU807
d__Bacteria	k__norank_d__Bacteria	p__Bacteroidota	c__Bacteroidia	o__Chitinophagales	f__Chitinophagaceae	g__Segetibacter	s__uncultured_bacterium_g__Segetibacter	OTU1614
d__Bacteria	k__norank_d__Bacteria	p__Chloroflexi	c__Anaerolineae	o__SBR1031	f__norank_o__SBR1031	g__norank_f__norank_o__SBR1031	s__uncultured_Gemmatimonadetes_bacterium_g__norank_f__norank	OTU1424
d__Bacteria	k__norank_d__Bacteria	p__Chloroflexi	c__AD3	o__norank_c__AD3	f__norank_o__norank_c__AD3	g__norank_f__norank_o__norank_c__AD3	s__uncultured_soil_bacterium_g__norank_f__norank_o__norank_c__AD3	OTU952

d__Bacteria	k__norank_d__Bacteria	p__Planctomycetota	c__Planctomycetes	o__Isosphaerales	f__Isosphaeraceae	g__unclassified_f__Isosphaeraceae	s__unclassified_f__Isosphaeraceae	OTU2089
d__Bacteria	k__norank_d__Bacteria	p__Proteobacteria	c__Gammaproteobacteria	o__Burkholderiales	f__Nitrosomonadaceae	g__MND1	s__unclassified_g__MND1	OTU2565
d__Bacteria	k__norank_d__Bacteria	p__unclassified_k__norank_d__Bacteria	c__unclassified_k__norank_d__Bacteria	o__unclassified_k__norank_d__Bacteria	f__unclassified_k__norank_d__Bacteria	g__unclassified_k__norank_d__Bacteria	s__unclassified_k__norank_d__Bacteria	OTU1248
d__Bacteria	k__norank_d__Bacteria	p__Chloroflexi	c__Ktedonobacteria	o__C0119	f__norank_o__C0119	g__norank_f__norank_o__C0119	s__uncultured_bacterium_g__norank_f__norank_o__C0119	OTU1363
d__Bacteria	k__norank_d__Bacteria	p__Chloroflexi	c__AD3	o__norank_c__AD3	f__norank_o__norank_c__AD3	g__norank_f__norank_o__norank_c__AD3	s__unclassified_g__norank_f__norank_o__norank_c__AD3	OTU725
d__Bacteria	k__norank_d__Bacteria	p__Proteobacteria	c__Gammaproteobacteria	o__Xanthomonadales	f__Xanthomonadaceae	g__Arenimonas	s__uncultured_bacterium_g__Arenimonas	OTU2443
d__Bacteria	k__norank_d__Bacteria	p__Planctomycetota	c__Planctomycetes	o__Isosphaerales	f__Isosphaeraceae	g__unclassified_f__Isosphaeraceae	s__unclassified_f__Isosphaeraceae	OTU2172
d__Bacteria	k__norank_d__Bacteria	p__Patescibacteria	c__Saccharimonadia	o__Saccharimonadales	f__norank_o__Saccharimonadales	g__norank_f__norank_o__Saccharimonadales	s__unclassified_g__norank_f__norank_o__Saccharimonadales	OTU1961
d__Bacteria	k__norank_d__Bacteria	p__Proteobacteria	c__Alphaproteobacteria	o__Rhizobiales	f__Beijerinckiaceae	g__norank_f__Beijerinckiaceae	s__unclassified_g__norank_f__Beijerinckiaceae	OTU2976
d__Bacteria	k__norank_d__Bacteria	p__Acidobacteriota	c__Acidobacteriae	o__Subgroup_15	f__norank_o__Subgroup_15	g__norank_f__norank_o__Subgroup_15	s__unclassified_g__norank_f__norank_o__Subgroup_15	OTU437
d__Bacteria	k__norank_d__Bacteria	p__Abditibacteriota	c__Abditibacteria	o__Abditibacteriales	f__Abditibacteriaceae	g__Abditibacterium	s__unclassified_g__Abditibacterium	OTU208
d__Bacteria	k__norank_d__Bacteria	p__Proteobacteria	c__Alphaproteobacteria	o__Rickettsiales	f__Mitochondria	g__norank_f__Mitochondria	s__unclassified_g__norank_f__Mitochondria	OTU749
d__Bacteria	k__norank_d__Bacteria	p__Proteobacteria	c__Alphaproteobacteria	o__Rhizobiales	f__Rhizobiales_Incertae_Sedis	g__Bauldia	s__uncultured_bacterium_g__Bauldia	OTU2290
d__Bacteria	k__norank_d__Bacteria	p__Chloroflexi	c__Anaerolineae	o__SBR1031	f__norank_o__SBR1031	g__norank_f__norank_o__SBR1031	s__uncultured_Gemmatimonadetes_bacterium_g__norank_f__norank	OTU1731
d__Bacteria	k__norank_d__Bacteria	p__Proteobacteria	c__Gammaproteobacteria	o__Burkholderiales	f__Comamonadaceae	g__Simplicispira	s__unclassified_g__Simplicispira	OTU2794
d__Bacteria	k__norank_d__Bacteria	p__Proteobacteria	c__Alphaproteobacteria	o__Rhizobiales	f__Xanthobacteraceae	g__norank_f__Xanthobacteraceae	s__unclassified_g__norank_f__Xanthobacteraceae	OTU2863

d__Bacteria	k__norank_d__Bacteria	p__Patescibacteria	c__Saccharimonadia	o__Saccharimonadales	f__LWQ8	g__norank_f__LWQ8	s__uncultured_bacterium_g__norank_f__LWQ8	OTU1785
d__Bacteria	k__norank_d__Bacteria	p__Myxococcota	c__Polyangia	o__unclassified_c__Polyangia	f__unclassified_c__Polyangia	g__unclassified_c__Polyangia	s__unclassified_c__Polyangia	OTU2420
d__Bacteria	k__norank_d__Bacteria	p__Actinobacteriota	c__Thermoleophilia	o__Solirubrobacterales	f__67-14	g__norank_f__67-14	s__unclassified_g__norank_f__67-14	OTU2702
d__Bacteria	k__norank_d__Bacteria	p__Bdellovibrionota	c__Oligoflexia	o__Oligoflexales	f__norank_o__Oligoflexales	g__Oligoflexus	s__unclassified_g__Oligoflexus	OTU2957
d__Bacteria	k__norank_d__Bacteria	p__Bacteroidota	c__Bacteroidia	o__Flavobacteriales	f__Flavobacteriaceae	g__Flavobacterium	s__unclassified_g__Flavobacterium	OTU236
d__Bacteria	k__norank_d__Bacteria	p__Patescibacteria	c__Saccharimonadia	o__Saccharimonadales	f__norank_o__Saccharimonadales	g__norank_f__norank_o__Saccharimonadales	s__uncultured_bacterium_g__norank_f__norank_o__Saccharimonadales	OTU1487
d__Bacteria	k__norank_d__Bacteria	p__Chloroflexi	c__Anaerolineae	o__SBR1031	f__A4b	g__norank_f__A4b	s__metagenome_g__norank_f__A4b	OTU2109
d__Bacteria	k__norank_d__Bacteria	p__Bacteroidota	c__Bacteroidia	o__Chitinophagales	f__Chitinophagaceae	g__Aurantisolimonas	s__unclassified_g__Aurantisolimonas	OTU1492
d__Bacteria	k__norank_d__Bacteria	p__Cyanobacteria	c__Cyanobacteriia	o__Chloroplast	f__norank_o__Chloroplast	g__norank_f__norank_o__Chloroplast	s__uncultured_Chlorophyta_g__norank	OTU1584
d__Bacteria	k__norank_d__Bacteria	p__Cyanobacteria	c__Cyanobacteriia	o__Chloroplast	f__norank_o__Chloroplast	g__norank_f__norank_o__Chloroplast	s__Pseudomuriella_schumacherensis	OTU2773
d__Bacteria	k__norank_d__Bacteria	p__Bacteroidota	c__Bacteroidia	o__Sphingobacteriales	f__unclassified_o__Sphingobacteriales	g__unclassified_o__Sphingobacteriales	s__unclassified_o__Sphingobacteriales	OTU1282
d__Bacteria	k__norank_d__Bacteria	p__Cyanobacteria	c__Cyanobacteriia	o__Cyanobacteriales	f__Chroococcidiopsaceae	g__Aliterella	s__uncultured_bacterium_g__Aliterella	OTU2930
d__Bacteria	k__norank_d__Bacteria	p__Myxococcota	c__Polyangia	o__Polyangiales	f__Sandaracinaceae	g__norank_f__Sandaracinaceae	s__uncultured_bacterium_g__norank_f__Sandaracinaceae	OTU2825
d__Bacteria	k__norank_d__Bacteria	p__Abditibacteriota	c__Abditibacteria	o__Abditibacteriales	f__Abditibacteriaceae	g__Abditibacterium	s__uncultured_soil_bacterium_g__Abditibacterium	OTU1730
d__Bacteria	k__norank_d__Bacteria	p__Bdellovibrionota	c__Bdellovibrionia	o__Bacteriovoracales	f__Bacteriovoracaceae	g__Peredibacter	s__metagenome_g__Peredibacter	OTU2821
d__Bacteria	k__norank_d__Bacteria	p__Proteobacteria	c__Gammaproteobacteria	o__Diplorickettsiales	f__Diplorickettsiaceae	g__norank_f__Diplorickettsiaceae	s__metagenome_g__norank_f__Diplorickettsiaceae	OTU1427

d__Bacteria	k__norank_ d__Bacteria	p__Armatimonadota	c__Chthonomonadetes	o__Chthonomonadales	f__Chthonomonadaceae	g__Chthonomonas	s__uncultured_bacterium_g__Chthonomonas	OTU730
d__Bacteria	k__norank_ d__Bacteria	p__Bacteroidota	c__Bacteroidia	o__Chitinophagales	f__Chitinophagaceae	g__Terrimonas	s__uncultured_bacterium_g__Terrimonas	OTU441
d__Bacteria	k__norank_ d__Bacteria	p__Bacteroidota	c__Bacteroidia	o__Sphingobacteriales	f__env.OPS_17	g__norank_f__env.OPS_17	s__unclassified_g__norank_f__env.OPS_17	OTU707
d__Bacteria	k__norank_ d__Bacteria	p__unclassified_k__no rank_d__Bacteria	c__unclassified_k__noran k_d__Bacteria	o__unclassified_k__norank_ d__Bacteria	f__unclassified_k__norank_d__Bacteria	g__unclassified_k__norank_d__Bacteri a	s__unclassified_k__norank_d__Bacteria	OTU1496
d__Bacteria	k__norank_ d__Bacteria	p__Myxococcota	c__Polyangia	o__Polyangiales	f__Polyangiaceae	g__unclassified_f__Polyangiaceae	s__unclassified_f__Polyangiaceae	OTU2389
d__Bacteria	k__norank_ d__Bacteria	p__Acidobacteriota	c__Vicinamibacteria	o__Vicinamibacterales	f__norank_o__Vicinamibacterales	g__norank_f__norank_o__Vicinamiba cterales	s__uncultured_bacterium_259	OTU1423
d__Bacteria	k__norank_ d__Bacteria	p__Bdellovibrionota	c__Oligoflexia	o__0319-6G20	f__norank_o__0319-6G20	g__norank_f__norank_o__0319-6G20	s__uncultured_bacterium_g__norank_f__norank_o__0319-6G20	OTU318
d__Bacteria	k__norank_ d__Bacteria	p__Bdellovibrionota	c__Bdellovibrionia	o__Bdellovibrionales	f__Bdellovibrionaceae	g__Bdellovibrio	s__uncultured_bacterium_g__Bdellovibrio	OTU2936
d__Bacteria	k__norank_ d__Bacteria	p__Proteobacteria	c__Gammaproteobacteria	o__Burkholderiales	f__unclassified_o__Burkholderiales	g__unclassified_o__Burkholderiales	s__unclassified_o__Burkholderiales	OTU808
d__Bacteria	k__norank_ d__Bacteria	p__Acidobacteriota	c__Acidobacteriae	o__Subgroup_2	f__norank_o__Subgroup_2	g__norank_f__norank_o__Subgroup_2	s__uncultured_Acidobacteria_bacterium_g__norank_f__norank_o__Subgroup_2	OTU2605
d__Bacteria	k__norank_ d__Bacteria	p__Bacteroidota	c__Bacteroidia	o__Cytophagales	f__Hymenobacteraceae	g__Hymenobacter	s__unclassified_g__Hymenobacter	OTU2288
d__Bacteria	k__norank_ d__Bacteria	p__Verrucomicrobiota	c__Verrucomicrobiae	o__Verrucomicrobiales	f__Rubritaleaceae	g__Luteolibacter	s__uncultured_bacterium_g__Luteolibacter	OTU2093
d__Bacteria	k__norank_ d__Bacteria	p__Proteobacteria	c__Alphaproteobacteria	o__Rhodospirillales	f__norank_o__Rhodospirillales	g__norank_f__norank_o__Rhodospirill ales	s__uncultured_Rhodospirillaceae_bacterium_g__norank_f__norank_o__Rhodospiril lales	OTU2842
d__Bacteria	k__norank_ d__Bacteria	p__Firmicutes	c__Bacilli	o__Paenibacillales	f__Paenibacillaceae	g__Ammoniphilus	s__uncultured_bacterium_g__Ammoniphilus	OTU2035
d__Bacteria	k__norank_ d__Bacteria	p__Gemmatimonadota	c__Gemmatimonadetes	o__Gemmatimonadales	f__Gemmatimonadaceae	g__unclassified_f__Gemmatimonadace ae	s__unclassified_f__Gemmatimonadaceae	OTU914
d__Bacteria	k__norank_ d__Bacteria	p__Proteobacteria	c__Gammaproteobacteria	o__Burkholderiales	f__Chromobacteriaceae	g__Vogesella	s__uncultured_beta_proteobacterium_g__Vogesella	OTU858

d__Bacteria	k__norank_ d__Bacteria	p__Bacteroidota	c__Bacteroidia	o__Chitinophagales	f__Chitinophagaceae	g__Parasegetibacter	s__unclassified_g__Parasegetibacter	OTU128
d__Bacteria	k__norank_ d__Bacteria	p__Actinobacteriota	c__Actinobacteria	o__Micrococcales	f__Intrasporangiaceae	g__Oryzihumus	s__uncultured_bacterium_g__Oryzihumus	OTU15
d__Bacteria	k__norank_ d__Bacteria	p__Proteobacteria	c__Alphaproteobacteria	o__Rhizobiales	f__Beijerinckiaceae	g__unclassified_f__Beijerinckiaceae	s__unclassified_f__Beijerinckiaceae	OTU2882
d__Bacteria	k__norank_ d__Bacteria	p__Proteobacteria	c__Gammaproteobacteria	o__Cellvibrionales	f__Cellvibrionaceae	g__Cellvibrio	s__uncultured_gamma_proteobacterium_g__Cellvibrio	OTU2298
d__Bacteria	k__norank_ d__Bacteria	p__Patescibacteria	c__Saccharimonadia	o__Saccharimonadales	f__unclassified_o__Saccharimonadales	g__unclassified_o__Saccharimonadale s	s__unclassified_o__Saccharimonadales	OTU950
d__Bacteria	k__norank_ d__Bacteria	p__Proteobacteria	c__Alphaproteobacteria	o__Rhizobiales	f__Rhizobiales_Incertae_Sedis	g__norank_f__Rhizobiales_Incertae_S edis	s__uncultured_bacterium_g__norank_f__Rhizobiales_Incertae_Sedis	OTU477
d__Bacteria	k__norank_ d__Bacteria	p__Proteobacteria	c__Alphaproteobacteria	o__Tistrellales	f__Geminicoccaceae	g__Candidatus_Alysiosphaera	s__unclassified_g__Candidatus_Alysiosphaera	OTU1406
d__Bacteria	k__norank_ d__Bacteria	p__Acidobacteriota	c__Acidobacteriae	o__Bryobacterales	f__Bryobacteraceae	g__Bryobacter	s__uncultured_prokaryote_g__Bryobacter	OTU105
d__Bacteria	k__norank_ d__Bacteria	p__Bacteroidota	c__Bacteroidia	o__Sphingobacteriales	f__Sphingobacteriaceae	g__Mucilaginibacter	s__unclassified_g__Mucilaginibacter	OTU1045
d__Bacteria	k__norank_ d__Bacteria	p__Actinobacteriota	c__Actinobacteria	o__Propionibacteriales	f__Propionibacteriaceae	g__unclassified_f__Propionibacteriace ae	s__unclassified_f__Propionibacteriaceae	OTU2764
d__Bacteria	k__norank_ d__Bacteria	p__unclassified_k__no rank_d__Bacteria	c__unclassified_k__noran k_d__Bacteria	o__unclassified_k__norank_ d__Bacteria	f__unclassified_k__norank_d__Bacteria	g__unclassified_k__norank_d__Bacteri a	s__unclassified_k__norank_d__Bacteria	OTU1644
d__Bacteria	k__norank_ d__Bacteria	p__Cyanobacteria	c__Cyanobacteriia	o__Chloroplast	f__norank_o__Chloroplast	g__norank_f__norank_o__Chloroplast	s__unclassified_g__norank_f__norank_o__Chloroplast	OTU2986
d__Bacteria	k__norank_ d__Bacteria	p__Myxococcota	c__Polyangia	o__mle1-27	f__norank_o__mle1-27	g__norank_f__norank_o__mle1-27	s__uncultured_delta_proteobacterium_g__norank_f__norank_o__mle1-27	OTU1907
d__Bacteria	k__norank_ d__Bacteria	p__Acidobacteriota	c__Acidobacteriae	o__Bryobacterales	f__Bryobacteraceae	g__Bryobacter	s__uncultured_bacterium_g__Bryobacter	OTU2588
d__Bacteria	k__norank_ d__Bacteria	p__Myxococcota	c__Polyangia	o__Polyangiales	f__Polyangiaceae	g__Pajaroellobacter	s__unclassified_g__Pajaroellobacter	OTU2030
d__Bacteria	k__norank_ d__Bacteria	p__Patescibacteria	c__Saccharimonadia	o__Saccharimonadales	f__unclassified_o__Saccharimonadales	g__unclassified_o__Saccharimonadale s	s__unclassified_o__Saccharimonadales	OTU2768

d__Bacteria	k__norank_ d__Bacteria	p__Bacteroidota	c__Bacteroidia	o__Chitinophagales	f__Chitinophagaceae	g__norank_f__Chitinophagaceae	s__uncultured_Chitinophagaceae_bacterium_g__norank_f__Chitinophagaceae	OTU1923
d__Bacteria	k__norank_ d__Bacteria	p__Acidobacteriota	c__Blastocatellia	o__Pyrinomonadales	f__Pyrinomonadaceae	g__RB41	s__uncultured_Acidobacteria_bacterium_g__RB41	OTU2883
d__Bacteria	k__norank_ d__Bacteria	p__Actinobacteriota	c__Acidimicrobiia	o__norank_c__Acidimicrobi ia	f__norank_o__norank_c__Acidimicrobiia	g__norank_f__norank_o__norank_c__ Acidimicrobiia	s__uncultured_Iamia_sp._g__norank	OTU591
d__Bacteria	k__norank_ d__Bacteria	p__Elusimicrobiota	c__Lineage_IIa	o__norank_c__Lineage_IIa	f__norank_o__norank_c__Lineage_IIa	g__norank_f__norank_o__norank_c__ Lineage_IIa	s__unclassified_g__norank_f__norank_o__norank_c__Lineage_IIa	OTU838
d__Bacteria	k__norank_ d__Bacteria	p__Chloroflexi	c__Ktedonobacteria	o__Ktedonobacterales	f__Ktedonobacteraceae	g__HSB_OF53-F07	s__uncultured_Ktedonobacter_sp._g__HSB_OF53-F07	OTU1555
d__Bacteria	k__norank_ d__Bacteria	p__Actinobacteriota	c__Thermoleophilia	o__Solirubrobacterales	f__Solirubrobacteraceae	g__Solirubrobacter	s__unclassified_g__Solirubrobacter	OTU2517
d__Bacteria	k__norank_ d__Bacteria	p__Bdellovibrionota	c__Oligoflexia	o__0319-6G20	f__norank_o__0319-6G20	g__norank_f__norank_o__0319-6G20	s__unclassified_g__norank_f__norank_o__0319-6G20	OTU742
d__Bacteria	k__norank_ d__Bacteria	p__Chloroflexi	c__KD4-96	o__norank_c__KD4-96	f__norank_o__norank_c__KD4-96	g__norank_f__norank_o__norank_c__ KD4-96	s__uncultured_bacterium_g__norank_f__norank_o__norank_c__KD4-96	OTU1790
d__Bacteria	k__norank_ d__Bacteria	p__Bacteroidota	c__SJA-28	o__norank_c__SJA-28	f__norank_o__norank_c__SJA-28	g__norank_f__norank_o__norank_c__ SJA-28	s__uncultured_Chlorobi_bacterium_g__norank_f__norank_o__norank	OTU1479
d__Bacteria	k__norank_ d__Bacteria	p__Myxococcota	c__Myxococcia	o__Myxococcales	f__Anaeromyxobacteraceae	g__Anaeromyxobacter	s__uncultured_delta_proteobacterium_g__Anaeromyxobacter	OTU870
d__Bacteria	k__norank_ d__Bacteria	p__Firmicutes	c__Bacilli	o__Entomoplasmatales	f__type_III	g__norank_f__type_III	s__uncultured_Mollicutes_bacterium_g__norank	OTU2337
d__Bacteria	k__norank_ d__Bacteria	p__Proteobacteria	c__Alphaproteobacteria	o__Sphingomonadales	f__Sphingomonadaceae	g__Sphingopyxis	s__Sphingopyxis_chilensis	OTU2355
d__Bacteria	k__norank_ d__Bacteria	p__Gemmatimonadota	c__Longimicrobia	o__Longimicrobiales	f__Longimicrobiaceae	g__norank_f__Longimicrobiaceae	s__metagenome_g__norank_f__Longimicrobiaceae	OTU1560
d__Bacteria	k__norank_ d__Bacteria	p__Chloroflexi	c__Chloroflexia	o__Chloroflexales	f__Herpetosiphonaceae	g__Herpetosiphon	s__uncultured_bacterium_g__Herpetosiphon	OTU216
d__Bacteria	k__norank_ d__Bacteria	p__Actinobacteriota	c__Actinobacteria	o__Propionibacteriales	f__Nocardioidaceae	g__Nocardioides	s__unclassified_g__Nocardioides	OTU210
d__Bacteria	k__norank_ d__Bacteria	p__Chloroflexi	c__Anaerolineae	o__SBR1031	f__norank_o__SBR1031	g__norank_f__norank_o__SBR1031	s__unclassified_g__norank_f__norank_o__SBR1031	OTU2683

d__Bacteria	k__norank_d__Bacteria	p__Patescibacteria	c__Saccharimonadia	o__Saccharimonadales	f__norank_o__Saccharimonadales	g__norank_f__norank_o__Saccharimonadales	s__uncultured_bacterium_g__norank_f__norank_o__Saccharimonadales	OTU428
d__Bacteria	k__norank_d__Bacteria	p__Actinobacteriota	c__Actinobacteria	o__Micromonosporales	f__Micromonosporaceae	g__Longispora	s__uncultured_bacterium_g__Longispora	OTU2983
d__Bacteria	k__norank_d__Bacteria	p__Planctomycetota	c__Planctomycetes	o__Gemmatales	f__Gemmataceae	g__norank_f__Gemmataceae	s__metagenome_g__norank_f__Gemmataceae	OTU156
d__Bacteria	k__norank_d__Bacteria	p__Chloroflexi	c__Chloroflexia	o__Chloroflexales	f__Chloroflexaceae	g__unclassified_f__Chloroflexaceae	s__unclassified_f__Chloroflexaceae	OTU2263
d__Bacteria	k__norank_d__Bacteria	p__Chloroflexi	c__Chloroflexia	o__Kallotenuales	f__AKIW781	g__norank_f__AKIW781	s__uncultured_bacterium_g__norank_f__AKIW781	OTU1182
d__Bacteria	k__norank_d__Bacteria	p__Elusimicrobiota	c__Lineage_IIa	o__norank_c__Lineage_IIa	f__norank_o__norank_c__Lineage_IIa	g__norank_f__norank_o__norank_c__Lineage_IIa	s__uncultured_bacterium_g__norank_f__norank_o__norank_c__Lineage_IIa	OTU1658
d__Bacteria	k__norank_d__Bacteria	p__Proteobacteria	c__Gammaproteobacteria	o__Legionellales	f__Legionellaceae	g__Legionella	s__unclassified_g__Legionella	OTU351
d__Bacteria	k__norank_d__Bacteria	p__unclassified_k__norank_d__Bacteria	c__unclassified_k__norank_d__Bacteria	o__unclassified_k__norank_d__Bacteria	f__unclassified_k__norank_d__Bacteria	g__unclassified_k__norank_d__Bacteria	s__unclassified_k__norank_d__Bacteria	OTU72
d__Bacteria	k__norank_d__Bacteria	p__Bacteroidota	c__Bacteroidia	o__Sphingobacteriales	f__env.OPS_17	g__norank_f__env.OPS_17	s__uncultured_bacterium_g__norank_f__env.OPS_17	OTU1607
d__Bacteria	k__norank_d__Bacteria	p__Armatimonadota	c__Chthonomonadetes	o__Chthonomonadales	f__Chthonomonadaceae	g__Chthonomonas	s__uncultured_bacterium_g__Chthonomonas	OTU1972
d__Bacteria	k__norank_d__Bacteria	p__Bacteroidota	c__Bacteroidia	o__Chitinophagales	f__Saprospiraceae	g__norank_f__Saprospiraceae	s__uncultured_soil_bacterium_g__norank_f__Saprospiraceae	OTU2671
d__Bacteria	k__norank_d__Bacteria	p__Chloroflexi	c__unclassified_p__Chloroflexi	o__unclassified_p__Chloroflexi	f__unclassified_p__Chloroflexi	g__unclassified_p__Chloroflexi	s__unclassified_p__Chloroflexi	OTU2649
d__Bacteria	k__norank_d__Bacteria	p__Dependentiae	c__Babeliae	o__Babeliales	f__Vermiphilaceae	g__norank_f__Vermiphilaceae	s__uncultured_bacterium_g__norank_f__Vermiphilaceae	OTU2052
d__Bacteria	k__norank_d__Bacteria	p__Proteobacteria	c__Gammaproteobacteria	o__Diplorickettsiales	f__Diplorickettsiaceae	g__Rickettsiella	s__unclassified_g__Rickettsiella	OTU1647
d__Bacteria	k__norank_d__Bacteria	p__Chloroflexi	c__Ktedonobacteria	o__B12-WMSP1	f__norank_o__B12-WMSP1	g__norank_f__norank_o__B12-WMSP1	s__uncultured_bacterium_g__norank_f__norank_o__B12-WMSP1	OTU972
d__Bacteria	k__norank_d__Bacteria	p__Actinobacteriota	c__Actinobacteria	o__Propionibacteriales	f__Nocardioidaceae	g__Nocardioides	s__unclassified_g__Nocardioides	OTU2870

d__Bacteria	k__norank_d__Bacteria	p__Myxococcota	c__Polyangia	o__Blfdi19	f__norank_o__Blfdi19	g__norank_f__norank_o__Blfdi19	s__unclassified_g__norank_f__norank_o__Blfdi19	OTU1084
d__Bacteria	k__norank_d__Bacteria	p__Proteobacteria	c__Alphaproteobacteria	o__Caulobacterales	f__Caulobacteraceae	g__norank_f__Caulobacteraceae	s__uncultured_Caulobacteraceae_bacterium_g__norank	OTU871
d__Bacteria	k__norank_d__Bacteria	p__Actinobacteriota	c__Actinobacteria	o__Propionibacteriales	f__Nocardioidaceae	g__Nocardioides	s__Nocardioides_sp._g__Nocardioides	OTU2372
d__Bacteria	k__norank_d__Bacteria	p__Myxococcota	c__Polyangia	o__Polyangiales	f__Polyangiaceae	g__Polyangium	s__unclassified_g__Polyangium	OTU573
d__Bacteria	k__norank_d__Bacteria	p__Chloroflexi	c__AD3	o__norank_c__AD3	f__norank_o__norank_c__AD3	g__norank_f__norank_o__norank_c__AD3	s__unclassified_g__norank_f__norank_o__norank_c__AD3	OTU1413
d__Bacteria	k__norank_d__Bacteria	p__Chloroflexi	c__AD3	o__norank_c__AD3	f__norank_o__norank_c__AD3	g__norank_f__norank_o__norank_c__AD3	s__uncultured_Chloroflexi_bacterium_g__norank_f__norank_o__norank_c__AD3	OTU672
d__Bacteria	k__norank_d__Bacteria	p__Bacteroidota	c__Bacteroidia	o__Flavobacteriales	f__NS9_marine_group	g__norank_f__NS9_marine_group	s__metagenome_g__norank_f__NS9_marine_group	OTU1634
d__Bacteria	k__norank_d__Bacteria	p__Gemmatimonadota	c__Gemmatimonadetes	o__Gemmatimonadales	f__Gemmatimonadaceae	g__Gemmatimonas	s__uncultured_soil_bacterium_g__Gemmatimonas	OTU1818
d__Bacteria	k__norank_d__Bacteria	p__Dependentiae	c__Babeliae	o__Babeliales	f__Babeliaceae	g__norank_f__Babeliaceae	s__uncultured_bacterium_g__norank_f__Babeliaceae	OTU1164
d__Bacteria	k__norank_d__Bacteria	p__Bacteroidota	c__Kapabacteria	o__Kapabacteriales	f__norank_o__Kapabacteriales	g__norank_f__norank_o__Kapabacteriales	s__uncultured_bacterium_0319-6E22	OTU2174
d__Bacteria	k__norank_d__Bacteria	p__Actinobacteriota	c__Actinobacteria	o__Streptomycetales	f__Streptomycetaceae	g__E1B-B3-114	s__uncultured_actinobacterium_g__E1B-B3-114	OTU2966
d__Bacteria	k__norank_d__Bacteria	p__Latescibacterota	c__norank_p__Latescibacterota	o__norank_c__norank_p__Latescibacterota	f__norank_o__norank_c__norank_p__Latescibacterota	g__norank_f__norank_o__norank_c__norank_p__Latescibacterota	s__unclassified_g__norank_f__norank_o__norank_c__norank_p__Latescibacterota	OTU2596
d__Bacteria	k__norank_d__Bacteria	p__Patescibacteria	c__Saccharimonadia	o__Saccharimonadales	f__norank_o__Saccharimonadales	g__norank_f__norank_o__Saccharimonadales	s__unclassified_g__norank_f__norank_o__Saccharimonadales	OTU2728
d__Bacteria	k__norank_d__Bacteria	p__Acidobacteriota	c__Vicinamibacteria	o__Vicinamibacterales	f__norank_o__Vicinamibacterales	g__norank_f__norank_o__Vicinamibacterales	s__uncultured_Acidobacteriaceae_bacterium_g__norank_f__norank_o__Vicinamibacterales	OTU1411
d__Bacteria	k__norank_d__Bacteria	p__Myxococcota	c__Polyangia	o__Haliangiales	f__Haliangiaceae	g__Haliangium	s__unclassified_g__Haliangium	OTU766
d__Bacteria	k__norank_d__Bacteria	p__Elusimicrobiota	c__Elusimicrobia	o__Lineage_IV	f__norank_o__Lineage_IV	g__norank_f__norank_o__Lineage_IV	s__uncultured_bacterium_g__norank_f__norank_o__Lineage_IV	OTU118

d__Bacteria	k__norank_ d__Bacteria	p__Myxococcota	c__Polyangia	o__Haliangiales	f__Haliangiaceae	g__Haliangium	s__unclassified_g__Haliangium	OTU762
d__Bacteria	k__norank_ d__Bacteria	p__Actinobacteriota	c__Thermoleophilia	o__Solirubrobacterales	f__unclassified_o__Solirubrobacterales	g__unclassified_o__Solirubrobacterale s	s__unclassified_o__Solirubrobacterales	OTU867
d__Bacteria	k__norank_ d__Bacteria	p__Acidobacteriota	c__Acidobacteriae	o__Solibacterales	f__Solibacteraceae	g__Candidatus_Solibacter	s__uncultured_bacterium_g__Candidatus_Solibacter	OTU1226
d__Bacteria	k__norank_ d__Bacteria	p__Chloroflexi	c__JG30-KF-CM66	o__norank_c__JG30-KF-C M66	f__norank_o__norank_c__JG30-KF-CM 66	g__norank_f__norank_o__norank_c__ JG30-KF-CM66	s__uncultured_bacterium_g__norank_f__norank_o__norank_c__JG30-KF-CM66	OTU636
d__Bacteria	k__norank_ d__Bacteria	p__Actinobacteriota	c__Thermoleophilia	o__Solirubrobacterales	f__Solirubrobacteraceae	g__unclassified_f__Solirubrobacterace ae	s__unclassified_f__Solirubrobacteraceae	OTU2023
d__Bacteria	k__norank_ d__Bacteria	p__Proteobacteria	c__Gammaproteobacteria	o__Salinisphaerales	f__Solimonadaceae	g__Alkanibacter	s__unclassified_g__Alkanibacter	OTU729
d__Bacteria	k__norank_ d__Bacteria	p__Firmicutes	c__Clostridia	o__Clostridiales	f__Clostridiaceae	g__Clostridium_sensu_stricto_5	s__unclassified_g__Clostridium_sensu_stricto_5	OTU2482
d__Bacteria	k__norank_ d__Bacteria	p__Chloroflexi	c__Chloroflexia	o__Kallotenuales	f__AKIW781	g__norank_f__AKIW781	s__uncultured_bacterium_g__norank_f__AKIW781	OTU600
d__Bacteria	k__norank_ d__Bacteria	p__Bacteroidota	c__Bacteroidia	o__Cytophagales	f__Microscillaceae	g__norank_f__Microscillaceae	s__unclassified_g__norank_f__Microscillaceae	OTU2620
d__Bacteria	k__norank_ d__Bacteria	p__Patescibacteria	c__Parcubacteria	o__Candidatus_Kaiserbacter ia	f__norank_o__Candidatus_Kaiserbacteria	g__norank_f__norank_o__Candidatus_ Kaiserbacteria	s__unclassified_g__norank_f__norank_o__Candidatus_Kaiserbacteria	OTU740
d__Bacteria	k__norank_ d__Bacteria	p__Actinobacteriota	c__Acidimicrobiia	o__Microtrichales	f__Ilumatobacteraceae	g__Ilumatobacter	s__unclassified_g__Ilumatobacter	OTU2250
d__Bacteria	k__norank_ d__Bacteria	p__Actinobacteriota	c__Thermoleophilia	o__Solirubrobacterales	f__67-14	g__norank_f__67-14	s__unclassified_g__norank_f__67-14	OTU412
d__Bacteria	k__norank_ d__Bacteria	p__Proteobacteria	c__Gammaproteobacteria	o__Burkholderiales	f__TRA3-20	g__norank_f__TRA3-20	s__uncultured_beta_proteobacterium_g__norank_f__TRA3-20	OTU2801
d__Bacteria	k__norank_ d__Bacteria	p__Chloroflexi	c__Ktedonobacteria	o__B12-WMSP1	f__norank_o__B12-WMSP1	g__norank_f__norank_o__B12-WMSP 1	s__uncultured_Chloroflexi_bacterium_g__norank_f__norank_o__B12-WMSP1	OTU1820
d__Bacteria	k__norank_ d__Bacteria	p__Bdellovibrionota	c__Oligoflexia	o__0319-6G20	f__norank_o__0319-6G20	g__norank_f__norank_o__0319-6G20	s__metagenome_g__norank_f__norank_o__0319-6G20	OTU146
d__Bacteria	k__norank_ d__Bacteria	p__Gemmatimonadota	c__S0134_terrestrial_grou p	o__norank_c__S0134_terres trial_group	f__norank_o__norank_c__S0134_terrestr ial_group	g__norank_f__norank_o__norank_c__ S0134_terrestrial_group	s__metagenome_g__norank_f__norank_o__norank_c__S0134_terrestrial_group	OTU610

d__Bacteria	k__norank_ d__Bacteria	p__Chloroflexi	c__Ktedonobacteria	o__B12-WMSP1	f__norank_o__B12-WMSP1	g__norank_f__norank_o__B12-WMSP 1	s__uncultured_bacterium_g__norank_f__norank_o__B12-WMSP1	OTU1897
d__Bacteria	k__norank_ d__Bacteria	p__Proteobacteria	c__Gammaproteobacteria	o__Xanthomonadales	f__Xanthomonadaceae	g__Arenimonas	s__metagenome_g__Arenimonas	OTU508
d__Bacteria	k__norank_ d__Bacteria	p__Firmicutes	c__Bacilli	o__Lactobacillales	f__Leuconostocaceae	g__Weissella	s__Weissella_cibaria	OTU94
d__Bacteria	k__norank_ d__Bacteria	p__Bacteroidota	c__Bacteroidia	o__Cytophagales	f__Microscillaceae	g__unclassified_f__Microscillaceae	s__unclassified_f__Microscillaceae	OTU1622
d__Bacteria	k__norank_ d__Bacteria	p__Myxococcota	c__Polyangia	o__Polyangiales	f__Polyangiaceae	g__Pajaroellobacter	s__unclassified_g__Pajaroellobacter	OTU99
d__Bacteria	k__norank_ d__Bacteria	p__Dependentiae	c__Babeliae	o__Babeliales	f__Vermiphilaceae	g__norank_f__Vermiphilaceae	s__uncultured_bacterium_g__norank_f__Vermiphilaceae	OTU689
d__Bacteria	k__norank_ d__Bacteria	p__Armatimonadota	c__Chthonomonadetes	o__Chthonomonadales	f__Chthonomonadaceae	g__Chthonomonas	s__uncultured_bacterium_g__Chthonomonas	OTU1212
d__Bacteria	k__norank_ d__Bacteria	p__Chloroflexi	c__Ktedonobacteria	o__B12-WMSP1	f__norank_o__B12-WMSP1	g__norank_f__norank_o__B12-WMSP 1	s__uncultured_bacterium_g__norank_f__norank_o__B12-WMSP1	OTU1714
d__Bacteria	k__norank_ d__Bacteria	p__Bacteroidota	c__Bacteroidia	o__Flavobacteriales	f__Flavobacteriaceae	g__Flavobacterium	s__unclassified_g__Flavobacterium	OTU220
d__Bacteria	k__norank_ d__Bacteria	p__Chloroflexi	c__TK10	o__norank_c__TK10	f__norank_o__norank_c__TK10	g__norank_f__norank_o__norank_c__ TK10	s__uncultured_soil_bacterium_g__norank_f__norank_o__norank_c__TK10	OTU1863
d__Bacteria	k__norank_ d__Bacteria	p__Chloroflexi	c__Anaerolineae	o__Anaerolineales	f__Anaerolineaceae	g__norank_f__Anaerolineaceae	s__unclassified_g__norank_f__Anaerolineaceae	OTU595
d__Bacteria	k__norank_ d__Bacteria	p__Firmicutes	c__Bacilli	o__Exiguobacterales	f__Exiguobacteraceae	g__Exiguobacterium	s__Exiguobacterium_sibiricum_255-15	OTU2273
d__Bacteria	k__norank_ d__Bacteria	p__Acidobacteriota	c__Vicinamibacteria	o__Vicinamibacterales	f__Vicinamibacteraceae	g__norank_f__Vicinamibacteraceae	s__unclassified_g__norank_f__Vicinamibacteraceae	OTU1407
d__Bacteria	k__norank_ d__Bacteria	p__Bdellovibrionota	c__Bdellovibrionia	o__Bdellovibrionales	f__Bdellovibrionaceae	g__OM27_clade	s__uncultured_soil_bacterium_g__OM27_clade	OTU336
d__Bacteria	k__norank_ d__Bacteria	p__Acidobacteriota	c__Vicinamibacteria	o__Vicinamibacterales	f__Vicinamibacteraceae	g__norank_f__Vicinamibacteraceae	s__unclassified_g__norank_f__Vicinamibacteraceae	OTU1405
d__Bacteria	k__norank_ d__Bacteria	p__Actinobacteriota	c__Thermoleophilia	o__Solirubrobacterales	f__67-14	g__norank_f__67-14	s__metagenome_g__norank_f__67-14	OTU2748

d__Bacteria	k__norank_ d__Bacteria	p__Proteobacteria	c__Alphaproteobacteria	o__Rhizobiales	f__Xanthobacteraceae	g__norank_f__Xanthobacteraceae	s__unclassified_g__norank_f__Xanthobacteraceae	OTU1980
d__Bacteria	k__norank_ d__Bacteria	p__Bacteroidota	c__Bacteroidia	o__Cytophagales	f__Spirosomaceae	g__Spirosoma	s__Spirosoma_luteum_DSM_19990	OTU2321
d__Bacteria	k__norank_ d__Bacteria	p__Actinobacteriota	c__Thermoleophilia	o__Solirubrobacterales	f__Solirubrobacteraceae	g__Solirubrobacter	s__uncultured_bacterium_g__Solirubrobacter	OTU2404
d__Bacteria	k__norank_ d__Bacteria	p__Planctomycetota	c__OM190	o__norank_c__OM190	f__norank_o__norank_c__OM190	g__norank_f__norank_o__norank_c__ OM190	s__uncultured_bacterium_g__norank_f__norank_o__norank_c__OM190	OTU2055
d__Bacteria	k__norank_ d__Bacteria	p__Chloroflexi	c__Ktedonobacteria	o__C0119	f__norank_o__C0119	g__norank_f__norank_o__C0119	s__uncultured_bacterium_g__norank_f__norank_o__C0119	OTU1936
d__Bacteria	k__norank_ d__Bacteria	p__Gemmatimonadota	c__Gemmatimonadetes	o__Gemmatimonadales	f__Gemmatimonadaceae	g__Gemmatimonas	s__unclassified_g__Gemmatimonas	OTU253
d__Bacteria	k__norank_ d__Bacteria	p__Actinobacteriota	c__Actinobacteria	o__Corynebacteriales	f__Dietziaceae	g__Dietzia	s__unclassified_g__Dietzia	OTU162
d__Bacteria	k__norank_ d__Bacteria	p__Elusimicrobiota	c__Lineage_IIa	o__norank_c__Lineage_IIa	f__norank_o__norank_c__Lineage_IIa	g__norank_f__norank_o__norank_c__ Lineage_IIa	s__uncultured_bacterium_g__norank_f__norank_o__norank_c__Lineage_IIa	OTU1128
d__Bacteria	k__norank_ d__Bacteria	p__Dependentiae	c__Babeliae	o__Babeliales	f__Vermiphilaceae	g__norank_f__Vermiphilaceae	s__uncultured_bacterium_g__norank_f__Vermiphilaceae	OTU977
d__Bacteria	k__norank_ d__Bacteria	p__Acidobacteriota	c__Acidobacteriae	o__Acidobacteriales	f__Acidobacteriaceae_Subgroup_1	g__norank_f__Acidobacteriaceae_Sub group_1	s__uncultured_bacterium_g__norank_f__Acidobacteriaceae_Subgroup_1	OTU1973
d__Bacteria	k__norank_ d__Bacteria	p__Cyanobacteria	c__Sericytochromatia	o__norank_c__Sericytochro matia	f__norank_o__norank_c__Sericytochrom atia	g__norank_f__norank_o__norank_c__ Sericytochromatia	s__uncultured_bacterium_g__norank_f__norank_o__norank_c__Sericytochromatia	OTU2147
d__Bacteria	k__norank_ d__Bacteria	p__Bdellovibrionota	c__Oligoflexia	o__0319-6G20	f__norank_o__0319-6G20	g__norank_f__norank_o__0319-6G20	s__unclassified_g__norank_f__norank_o__0319-6G20	OTU839
d__Bacteria	k__norank_ d__Bacteria	p__Firmicutes	c__Clostridia	o__Peptostreptococcales-Tis sierellales	f__norank_o__Peptostreptococcales-Tissi erellales	g__Parvimonas	s__Parvimonas_micra	OTU554
d__Bacteria	k__norank_ d__Bacteria	p__Acidobacteriota	c__Acidobacteriae	o__Acidobacteriales	f__norank_o__Acidobacteriales	g__norank_f__norank_o__Acidobacter iales	s__uncultured_bacterium_g__norank_f__norank_o__Acidobacteriales	OTU1126
d__Bacteria	k__norank_ d__Bacteria	p__Acidobacteriota	c__Acidobacteriae	o__Bryobacterales	f__Bryobacteraceae	g__Bryobacter	s__uncultured_Acidobacteria_bacterium_g__Bryobacter	OTU1603
d__Bacteria	k__norank_ d__Bacteria	p__Acidobacteriota	c__Thermoanaerobaculia	o__Thermoanaerobaculales	f__Thermoanaerobaculaceae	g__Subgroup_10	s__uncultured_Holophaga_sp._g__Subgroup_10	OTU2436

d__Bacteria	k__norank_ d__Bacteria	p__Bdellovibrionota	c__Oligoflexia	o__0319-6G20	f__norank_o__0319-6G20	g__norank_f__norank_o__0319-6G20	s__unclassified_g__norank_f__norank_o__0319-6G20	OTU548
d__Bacteria	k__norank_ d__Bacteria	p__Myxococcota	c__Myxococcia	o__Myxococcales	f__Anaeromyxobacteraceae	g__Anaeromyxobacter	s__uncultured_bacterium_g__Anaeromyxobacter	OTU887
d__Bacteria	k__norank_ d__Bacteria	p__Firmicutes	c__Bacilli	o__Alicyclobacillales	f__Alicyclobacillaceae	g__Tumebacillus	s__uncultured_bacterium_g__Tumebacillus	OTU1417
d__Bacteria	k__norank_ d__Bacteria	p__Abditibacteriota	c__Abditibacteria	o__Abditibacteriales	f__Abditibacteriaceae	g__Abditibacterium	s__uncultured_bacterium_g__Abditibacterium	OTU2056
d__Bacteria	k__norank_ d__Bacteria	p__Chloroflexi	c__Chloroflexia	o__Thermomicrobiales	f__JG30-KF-CM45	g__norank_f__JG30-KF-CM45	s__unclassified_g__norank_f__JG30-KF-CM45	OTU368
d__Bacteria	k__norank_ d__Bacteria	p__Verrucomicrobiota	c__Verrucomicrobiae	o__Chthoniobacterales	f__Chthoniobacteraceae	g__Candidatus_Udaeobacter	s__unclassified_g__Candidatus_Udaeobacter	OTU2761
d__Bacteria	k__norank_ d__Bacteria	p__Proteobacteria	c__Alphaproteobacteria	o__Caulobacterales	f__Caulobacteraceae	g__PMMR1	s__unclassified_g__PMMR1	OTU2928
d__Bacteria	k__norank_ d__Bacteria	p__Chloroflexi	c__JG30-KF-CM66	o__norank_c__JG30-KF-C M66	f__norank_o__norank_c__JG30-KF-CM 66	g__norank_f__norank_o__norank_c__ JG30-KF-CM66	s__uncultured_soil_bacterium_g__norank_f__norank_o__norank_c__JG30-KF-CM 66	OTU1956
d__Bacteria	k__norank_ d__Bacteria	p__Actinobacteriota	c__Thermoleophilia	o__Gaiellales	f__norank_o__Gaiellales	g__norank_f__norank_o__Gaiellales	s__uncultured_Rubrobacterales_bacterium_g__norank_f__norank	OTU1360
d__Bacteria	k__norank_ d__Bacteria	p__Actinobacteriota	c__Actinobacteria	o__Propionibacteriales	f__Nocardioidaceae	g__Nocardioides	s__unclassified_g__Nocardioides	OTU2739
d__Bacteria	k__norank_ d__Bacteria	p__Acidobacteriota	c__Vicinamibacteria	o__Vicinamibacterales	f__norank_o__Vicinamibacterales	g__norank_f__norank_o__Vicinamiba cterales	s__unclassified_g__norank_f__norank_o__Vicinamibacterales	OTU1414
d__Bacteria	k__norank_ d__Bacteria	p__Bacteroidota	c__Kapabacteria	o__Kapabacteriales	f__norank_o__Kapabacteriales	g__norank_f__norank_o__Kapabacteri ales	s__unclassified_g__norank_f__norank_o__Kapabacteriales	OTU2668
d__Bacteria	k__norank_ d__Bacteria	p__Acidobacteriota	c__Vicinamibacteria	o__Subgroup_17	f__norank_o__Subgroup_17	g__norank_f__norank_o__Subgroup_1 7	s__metagenome_g__norank_f__norank_o__Subgroup_17	OTU429
d__Bacteria	k__norank_ d__Bacteria	p__Proteobacteria	c__Alphaproteobacteria	o__Elsterales	f__norank_o__Elsterales	g__norank_f__norank_o__Elsterales	s__unclassified_g__norank_f__norank_o__Elsterales	OTU1946
d__Bacteria	k__norank_ d__Bacteria	p__Actinobacteriota	c__Actinobacteria	o__Propionibacteriales	f__Propionibacteriaceae	g__unclassified_f__Propionibacteriace ae	s__unclassified_f__Propionibacteriaceae	OTU584
d__Bacteria	k__norank_ d__Bacteria	p__Chloroflexi	c__Anaerolineae	o__SBR1031	f__norank_o__SBR1031	g__norank_f__norank_o__SBR1031	s__uncultured_Gemmatimonadetes_bacterium_g__norank_f__norank	OTU2004

d__Bacteria	k__norank_ d__Bacteria	p__Patescibacteria	c__Parcubacteria	o__norank_c__Parcubacteria	f__norank_o__norank_c__Parcubacteria	g__norank_f__norank_o__norank_c__ Parcubacteria	s__uncultured_bacterium_g__norank_f__norank_o__norank_c__Parcubacteria	OTU1611
d__Bacteria	k__norank_ d__Bacteria	p__Patescibacteria	c__Saccharimonadia	o__Saccharimonadales	f__LWQ8	g__norank_f__LWQ8	s__unclassified_g__norank_f__LWQ8	OTU2537
d__Bacteria	k__norank_ d__Bacteria	p__Chloroflexi	c__Anaerolineae	o__SBR1031	f__norank_o__SBR1031	g__norank_f__norank_o__SBR1031	s__unclassified_g__norank_f__norank_o__SBR1031	OTU2046
d__Bacteria	k__norank_ d__Bacteria	p__Chloroflexi	c__Ktedonobacteria	o__Ktedonobacterales	f__Ktedonobacteraceae	g__JG30a-KF-32	s__uncultured_Ktedobacteria_bacterium_g__JG30a-KF-32	OTU1708
d__Bacteria	k__norank_ d__Bacteria	p__Patescibacteria	c__Saccharimonadia	o__Saccharimonadales	f__norank_o__Saccharimonadales	g__norank_f__norank_o__Saccharimo nadales	s__unclassified_g__norank_f__norank_o__Saccharimonadales	OTU483
d__Bacteria	k__norank_ d__Bacteria	p__Cyanobacteria	c__Cyanobacteriia	o__Chloroplast	f__norank_o__Chloroplast	g__norank_f__norank_o__Chloroplast	s__unclassified_g__norank_f__norank_o__Chloroplast	OTU2762
d__Bacteria	k__norank_ d__Bacteria	p__Latescibacterota	c__norank_p__Latescibact erota	o__norank_c__norank_p__L atescibacterota	f__norank_o__norank_c__norank_p__La tescibacterota	g__norank_f__norank_o__norank_c__ norank_p__Latescibacterota	s__unclassified_g__norank_f__norank_o__norank_c__norank_p__Latescibacterota	OTU986
d__Bacteria	k__norank_ d__Bacteria	p__Cyanobacteria	c__Cyanobacteriia	o__Chloroplast	f__norank_o__Chloroplast	g__norank_f__norank_o__Chloroplast	s__unclassified_g__norank_f__norank_o__Chloroplast	OTU83
d__Bacteria	k__norank_ d__Bacteria	p__Patescibacteria	c__Saccharimonadia	o__Saccharimonadales	f__norank_o__Saccharimonadales	g__norank_f__norank_o__Saccharimo nadales	s__uncultured_bacterium_g__norank_f__norank_o__Saccharimonadales	OTU324
d__Bacteria	k__norank_ d__Bacteria	p__Chloroflexi	c__Chloroflexia	o__Kallotenuales	f__AKIW781	g__norank_f__AKIW781	s__unclassified_g__norank_f__AKIW781	OTU2965
d__Bacteria	k__norank_ d__Bacteria	p__Bacteroidota	c__Bacteroidia	o__Sphingobacteriales	f__Sphingobacteriaceae	g__Mucilaginibacter	s__uncultured_bacterium_g__Mucilaginibacter	OTU1685
d__Bacteria	k__norank_ d__Bacteria	p__Proteobacteria	c__Gammaproteobacteria	o__Burkholderiales	f__Leeiaceae	g__Leeia	s__uncultured_bacterium_g__Leeia	OTU1686
d__Bacteria	k__norank_ d__Bacteria	p__Chloroflexi	c__JG30-KF-CM66	o__norank_c__JG30-KF-C M66	f__norank_o__norank_c__JG30-KF-CM 66	g__norank_f__norank_o__norank_c__ JG30-KF-CM66	s__unclassified_g__norank_f__norank_o__norank_c__JG30-KF-CM66	OTU2031
d__Bacteria	k__norank_ d__Bacteria	p__Actinobacteriota	c__Actinobacteria	o__Propionibacteriales	f__Nocardioidaceae	g__Nocardioides	s__Nocardioides_dilutus	OTU158
d__Bacteria	k__norank_ d__Bacteria	p__Chloroflexi	c__Anaerolineae	o__SBR1031	f__A4b	g__norank_f__A4b	s__uncultured_soil_bacterium_g__norank_f__A4b	OTU2108
d__Bacteria	k__norank_ d__Bacteria	p__Chloroflexi	c__Chloroflexia	o__Thermomicrobiales	f__JG30-KF-CM45	g__norank_f__JG30-KF-CM45	s__uncultured_Chloroflexi_bacterium_g__norank_f__JG30-KF-CM45	OTU685

d__Bacteria	k__norank_d__Bacteria	p__Proteobacteria	c__Gammaproteobacteria	o__Legionellales	f__Legionellaceae	g__Legionella	s__uncultured_bacterium_g__Legionella	OTU2691
d__Bacteria	k__norank_d__Bacteria	p__Chloroflexi	c__Chloroflexia	o__Thermomicrobiales	f__JG30-KF-CM45	g__norank_f__JG30-KF-CM45	s__unclassified_g__norank_f__JG30-KF-CM45	OTU409
d__Bacteria	k__norank_d__Bacteria	p__Chloroflexi	c__Anaerolineae	o__SBR1031	f__A4b	g__norank_f__A4b	s__uncultured_soil_bacterium_g__norank_f__A4b	OTU2102
d__Bacteria	k__norank_d__Bacteria	p__Actinobacteriota	c__Actinobacteria	o__Pseudonocardiales	f__Pseudonocardiaceae	g__Pseudonocardia	s__uncultured_bacterium_g__Pseudonocardia	OTU1589
d__Bacteria	k__norank_d__Bacteria	p__Proteobacteria	c__Gammaproteobacteria	o__Vibrionales	f__Vibrionaceae	g__Photobacterium	s__Photobacterium_damselae	OTU77
d__Bacteria	k__norank_d__Bacteria	p__Chloroflexi	c__Anaerolineae	o__Anaerolineales	f__Anaerolineaceae	g__norank_f__Anaerolineaceae	s__uncultured_sludge_bacterium_H39	OTU367
d__Bacteria	k__norank_d__Bacteria	p__Verrucomicrobiota	c__Chlamydiae	o__Chlamydiales	f__Parachlamydiaceae	g__Neochlamydia	s__metagenome_g__Neochlamydia	OTU1028
d__Bacteria	k__norank_d__Bacteria	p__Actinobacteriota	c__Acidimicrobiia	o__IMCC26256	f__norank_o__IMCC26256	g__norank_f__norank_o__IMCC26256	s__bacterium_enrichment_culture_clone_auto73_4W	OTU1770
d__Bacteria	k__norank_d__Bacteria	p__Chloroflexi	c__SHA-26	o__norank_c__SHA-26	f__norank_o__norank_c__SHA-26	g__norank_f__norank_o__norank_c__SHA-26	s__uncultured_bacterium_g__norank_f__norank_o__norank_c__SHA-26	OTU1027
d__Bacteria	k__norank_d__Bacteria	p__unclassified_k__norank_d__Bacteria	c__unclassified_k__norank_d__Bacteria	o__unclassified_k__norank_d__Bacteria	f__unclassified_k__norank_d__Bacteria	g__unclassified_k__norank_d__Bacteria	s__unclassified_k__norank_d__Bacteria	OTU1152
d__Bacteria	k__norank_d__Bacteria	p__Proteobacteria	c__Gammaproteobacteria	o__Burkholderiales	f__Comamonadaceae	g__Polaromonas	s__unclassified_g__Polaromonas	OTU2807
d__Bacteria	k__norank_d__Bacteria	p__Gemmatimonadota	c__Gemmatimonadetes	o__Gemmatimonadales	f__Gemmatimonadaceae	g__norank_f__Gemmatimonadaceae	s__unclassified_g__norank_f__Gemmatimonadaceae	OTU1528
d__Bacteria	k__norank_d__Bacteria	p__Planctomycetota	c__Planctomycetes	o__Gemmatales	f__Gemmataceae	g__norank_f__Gemmataceae	s__unclassified_g__norank_f__Gemmataceae	OTU1817
d__Bacteria	k__norank_d__Bacteria	p__Patescibacteria	c__Saccharimonadia	o__Saccharimonadales	f__Saccharimonadaceae	g__TM7a	s__uncultured_bacterium_g__TM7a	OTU2980
d__Bacteria	k__norank_d__Bacteria	p__Gemmatimonadota	c__Gemmatimonadetes	o__Gemmatimonadales	f__Gemmatimonadaceae	g__Gemmatimonas	s__uncultured_bacterium_g__Gemmatimonas	OTU1627
d__Bacteria	k__norank_d__Bacteria	p__Chloroflexi	c__Anaerolineae	o__RBG-13-54-9	f__norank_o__RBG-13-54-9	g__norank_f__norank_o__RBG-13-54-9	s__unclassified_g__norank_f__norank_o__RBG-13-54-9	OTU2881

d__Bacteria	k__norank_d__Bacteria	p__Firmicutes	c__Bacilli	o__Paenibacillales	f__Paenibacillaceae	g__Paenibacillus	s__unclassified_g__Paenibacillus	OTU1547
d__Bacteria	k__norank_d__Bacteria	p__Acidobacteriota	c__Holophagae	o__Subgroup_7	f__norank_o__Subgroup_7	g__norank_f__norank_o__Subgroup_7	s__unclassified_g__norank_f__norank_o__Subgroup_7	OTU2139
d__Bacteria	k__norank_d__Bacteria	p__Bacteroidota	c__Bacteroidia	o__Flavobacteriales	f__Flavobacteriaceae	g__Flavobacterium	s__unclassified_g__Flavobacterium	OTU2736
d__Bacteria	k__norank_d__Bacteria	p__unclassified_k__norank_d__Bacteria	c__unclassified_k__norank_d__Bacteria	o__unclassified_k__norank_d__Bacteria	f__unclassified_k__norank_d__Bacteria	g__unclassified_k__norank_d__Bacteria	s__unclassified_k__norank_d__Bacteria	OTU831
d__Bacteria	k__norank_d__Bacteria	p__Myxococcota	c__Polyangia	o__Polyangiales	f__Sandaracinaceae	g__norank_f__Sandaracinaceae	s__unclassified_g__norank_f__Sandaracinaceae	OTU2419
d__Bacteria	k__norank_d__Bacteria	p__Acidobacteriota	c__Vicinamibacteria	o__Vicinamibacterales	f__Vicinamibacteraceae	g__norank_f__Vicinamibacteraceae	s__uncultured_Acidobacteria_bacterium_g__norank_f__Vicinamibacteraceae	OTU2176
d__Bacteria	k__norank_d__Bacteria	p__Proteobacteria	c__Alphaproteobacteria	o__Rickettsiales	f__Mitochondria	g__norank_f__Mitochondria	s__unclassified_g__norank_f__Mitochondria	OTU2909
d__Bacteria	k__norank_d__Bacteria	p__Chloroflexi	c__Ktedonobacteria	o__C0119	f__norank_o__C0119	g__norank_f__norank_o__C0119	s__uncultured_bacterium_g__norank_f__norank_o__C0119	OTU1553
d__Bacteria	k__norank_d__Bacteria	p__Verrucomicrobiota	c__Verrucomicrobiae	o__Opitutales	f__Opitutaceae	g__Opitutus	s__metagenome_g__Opitutus	OTU2311
d__Bacteria	k__norank_d__Bacteria	p__Bdellovibrionota	c__Oligoflexia	o__Oligoflexales	f__norank_o__Oligoflexales	g__norank_f__norank_o__Oligoflexales	s__unclassified_g__norank_f__norank_o__Oligoflexales	OTU2712
d__Bacteria	k__norank_d__Bacteria	p__Myxococcota	c__Polyangia	o__Polyangiales	f__BIrii41	g__norank_f__BIrii41	s__uncultured_Sorangiineae_bacterium_g__norank	OTU1315
d__Bacteria	k__norank_d__Bacteria	p__Dependentiae	c__Babeliae	o__Babeliales	f__Vermiphilaceae	g__norank_f__Vermiphilaceae	s__uncultured_bacterium_g__norank_f__Vermiphilaceae	OTU1100
d__Bacteria	k__norank_d__Bacteria	p__Chloroflexi	c__Chloroflexia	o__Chloroflexales	f__Herpetosiphonaceae	g__Herpetosiphon	s__uncultured_bacterium_g__Herpetosiphon	OTU2198
d__Bacteria	k__norank_d__Bacteria	p__Patescibacteria	c__Saccharimonadia	o__Saccharimonadales	f__LWQ8	g__norank_f__LWQ8	s__unclassified_g__norank_f__LWQ8	OTU2459
d__Bacteria	k__norank_d__Bacteria	p__Bacteroidota	c__Bacteroidia	o__Cytophagales	f__Spirosomaceae	g__Fibrella	s__uncultured_bacterium_g__Fibrella	OTU43
d__Bacteria	k__norank_d__Bacteria	p__Myxococcota	c__Polyangia	o__Haliangiales	f__Haliangiaceae	g__Haliangium	s__metagenome_g__Haliangium	OTU2090

d__Bacteria	k__norank_ d__Bacteria	p__Cyanobacteria	c__Cyanobacteriia	o__Chloroplast	f__norank_o__Chloroplast	g__norank_f__norank_o__Chloroplast	s__uncultured_diatom	OTU2197
d__Bacteria	k__norank_ d__Bacteria	p__Gemmatimonadota	c__Gemmatimonadetes	o__Gemmatimonadales	f__Gemmatimonadaceae	g__unclassified_f__Gemmatimonadace ae	s__unclassified_f__Gemmatimonadaceae	OTU1601
d__Bacteria	k__norank_ d__Bacteria	p__Patescibacteria	c__Saccharimonadia	o__Saccharimonadales	f__Saccharimonadaceae	g__TM7a	s__uncultured_bacterium_g__TM7a	OTU898
d__Bacteria	k__norank_ d__Bacteria	p__Proteobacteria	c__Alphaproteobacteria	o__Rhodobacterales	f__Rhodobacteraceae	g__Rubellimicrobium	s__uncultured_bacterium_g__Rubellimicrobium	OTU2964
d__Bacteria	k__norank_ d__Bacteria	p__Bacteroidota	c__Bacteroidia	o__Flavobacteriales	f__Flavobacteriaceae	g__Flavobacterium	s__unclassified_g__Flavobacterium	OTU1643
d__Bacteria	k__norank_ d__Bacteria	p__Armatimonadota	c__Chthonomonadetes	o__Chthonomonadales	f__Chthonomonadaceae	g__Chthonomonas	s__uncultured_bacterium_g__Chthonomonas	OTU8
d__Bacteria	k__norank_ d__Bacteria	p__Bacteroidota	c__Bacteroidia	o__Chitinophagales	f__Chitinophagaceae	g__norank_f__Chitinophagaceae	s__uncultured_bacterium_g__norank_f__Chitinophagaceae	OTU1451
d__Bacteria	k__norank_ d__Bacteria	p__Proteobacteria	c__Alphaproteobacteria	o__Rhizobiales	f__D05-2	g__norank_f__D05-2	s__uncultured_bacterium_g__norank_f__D05-2	OTU2908
d__Bacteria	k__norank_ d__Bacteria	p__Bacteroidota	c__Bacteroidia	o__Sphingobacteriales	f__Sphingobacteriaceae	g__Mucilaginibacter	s__unclassified_g__Mucilaginibacter	OTU1720
d__Bacteria	k__norank_ d__Bacteria	p__Proteobacteria	c__Alphaproteobacteria	o__Reyranellales	f__Reyranellaceae	g__Reyranella	s__metagenome_g__Reyranella	OTU1960
d__Bacteria	k__norank_ d__Bacteria	p__Myxococcota	c__Polyangia	o__Haliangiales	f__Haliangiaceae	g__Haliangium	s__uncultured_bacterium_g__Haliangium	OTU897
d__Bacteria	k__norank_ d__Bacteria	p__unclassified_k__no rank_d__Bacteria	c__unclassified_k__noran k_d__Bacteria	o__unclassified_k__norank_ d__Bacteria	f__unclassified_k__norank_d__Bacteria	g__unclassified_k__norank_d__Bacteri a	s__unclassified_k__norank_d__Bacteria	OTU828
d__Bacteria	k__norank_ d__Bacteria	p__Bacteroidota	c__Bacteroidia	o__Bacteroidales	f__Marinifilaceae	g__Marinifilum	s__Marinifilum_fragile_CECT_7942	OTU482
d__Bacteria	k__norank_ d__Bacteria	p__Proteobacteria	c__Gammaproteobacteria	o__Burkholderiales	f__SC-I-84	g__norank_f__SC-I-84	s__uncultured_bacterium_g__norank_f__SC-I-84	OTU695
d__Bacteria	k__norank_ d__Bacteria	p__Proteobacteria	c__Gammaproteobacteria	o__Burkholderiales	f__A21b	g__norank_f__A21b	s__unclassified_g__norank_f__A21b	OTU2485
d__Bacteria	k__norank_ d__Bacteria	p__Actinobacteriota	c__Actinobacteria	o__Corynebacteriales	f__Mycobacteriaceae	g__Mycobacterium	s__unclassified_g__Mycobacterium	OTU2462

d__Bacteria	k__norank_d__Bacteria	p__Chloroflexi	c__Chloroflexia	o__Chloroflexales	f__Herpetosiphonaceae	g__Herpetosiphon	s__uncultured_Chloroflexi_bacterium_g__Herpetosiphon	OTU296
d__Bacteria	k__norank_d__Bacteria	p__Bdellovibrionota	c__Bdellovibrionia	o__Bdellovibrionales	f__Bdellovibrionaceae	g__Bdellovibrio	s__unclassified_g__Bdellovibrio	OTU490
d__Bacteria	k__norank_d__Bacteria	p__Actinobacteriota	c__Actinobacteria	o__Corynebacteriales	f__Nocardiaceae	g__Rhodococcus	s__Rhodococcus_fascians	OTU2449
d__Bacteria	k__norank_d__Bacteria	p__Patescibacteria	c__Saccharimonadia	o__Saccharimonadales	f__norank_o__Saccharimonadales	g__norank_f__norank_o__Saccharimonadales	s__unclassified_g__norank_f__norank_o__Saccharimonadales	OTU2857
d__Bacteria	k__norank_d__Bacteria	p__Planctomycetota	c__Planctomycetes	o__Gemmatales	f__Gemmataceae	g__norank_f__Gemmataceae	s__unclassified_g__norank_f__Gemmataceae	OTU771
d__Bacteria	k__norank_d__Bacteria	p__Bacteroidota	c__Bacteroidia	o__Sphingobacteriales	f__KD3-93	g__norank_f__KD3-93	s__unclassified_g__norank_f__KD3-93	OTU269
d__Bacteria	k__norank_d__Bacteria	p__Chloroflexi	c__JG30-KF-CM66	o__norank_c__JG30-KF-CM66	f__norank_o__norank_c__JG30-KF-CM66	g__norank_f__norank_o__norank_c__JG30-KF-CM66	s__uncultured_Caldilinea_sp._g__norank	OTU1951
d__Bacteria	k__norank_d__Bacteria	p__Proteobacteria	c__Gammaproteobacteria	o__Xanthomonadales	f__Xanthomonadaceae	g__norank_f__Xanthomonadaceae	s__unclassified_g__norank_f__Xanthomonadaceae	OTU1739
d__Bacteria	k__norank_d__Bacteria	p__Proteobacteria	c__Alphaproteobacteria	o__Caulobacterales	f__Caulobacteraceae	g__Brevundimonas	s__unclassified_g__Brevundimonas	OTU2713
d__Bacteria	k__norank_d__Bacteria	p__Gemmatimonadota	c__Gemmatimonadetes	o__Gemmatimonadales	f__Gemmatimonadaceae	g__Roseisolibacter	s__unclassified_g__Roseisolibacter	OTU1650
d__Bacteria	k__norank_d__Bacteria	p__Proteobacteria	c__Alphaproteobacteria	o__Rhizobiales	f__Beijerinckiaceae	g__1174-901-12	s__uncultured_bacterium_g__1174-901-12	OTU53
d__Bacteria	k__norank_d__Bacteria	p__WPS-2	c__norank_p__WPS-2	o__norank_c__norank_p__WPS-2	f__norank_o__norank_c__norank_p__WPS-2	g__norank_f__norank_o__norank_c__norank_p__WPS-2	s__metagenome_g__norank_f__norank_o__norank_c__norank_p__WPS-2	OTU908
d__Bacteria	k__norank_d__Bacteria	p__Acidobacteriota	c__Holophagae	o__Subgroup_7	f__norank_o__Subgroup_7	g__norank_f__norank_o__Subgroup_7	s__unclassified_g__norank_f__norank_o__Subgroup_7	OTU2457
d__Bacteria	k__norank_d__Bacteria	p__Abditibacteriota	c__Abditibacteria	o__Abditibacteriales	f__Abditibacteriaceae	g__Abditibacterium	s__uncultured_bacterium_g__Abditibacterium	OTU399
d__Bacteria	k__norank_d__Bacteria	p__Proteobacteria	c__Alphaproteobacteria	o__Rhodobacterales	f__Rhodobacteraceae	g__Rubellimicrobium	s__uncultured_bacterium_g__Rubellimicrobium	OTU2841
d__Bacteria	k__norank_d__Bacteria	p__Bacteroidota	c__Bacteroidia	o__Cytophagales	f__Cytophagaceae	g__Rhodocytophaga	s__uncultured_bacterium_g__Rhodocytophaga	OTU2464

d__Bacteria	k__norank_ d__Bacteria	p__Bacteroidota	c__Bacteroidia	o__Sphingobacteriales	f__NS11-12_marine_group	g__norank_f__NS11-12_marine_group	s__metagenome_g__norank_f__NS11-12_marine_group	OTU844
d__Bacteria	k__norank_ d__Bacteria	p__Actinobacteriota	c__Acidimicrobiia	o__IMCC26256	f__norank_o__IMCC26256	g__norank_f__norank_o__IMCC26256	s__unclassified_g__norank_f__norank_o__IMCC26256	OTU599
d__Bacteria	k__norank_ d__Bacteria	p__Actinobacteriota	c__Actinobacteria	o__Bifidobacteriales	f__Bifidobacteriaceae	g__Bifidobacterium	s__Bifidobacterium_pseudocatenulatum_DSM_20438__JCM_1200__LMG_10505	OTU2970
d__Bacteria	k__norank_ d__Bacteria	p__Bdellovibrionota	c__Oligoflexia	o__Oligoflexales	f__norank_o__Oligoflexales	g__Oligoflexus	s__uncultured_bacterium_g__Oligoflexus	OTU2962
d__Bacteria	k__norank_ d__Bacteria	p__Bdellovibrionota	c__Oligoflexia	o__0319-6G20	f__norank_o__0319-6G20	g__norank_f__norank_o__0319-6G20	s__unclassified_g__norank_f__norank_o__0319-6G20	OTU1747
d__Bacteria	k__norank_ d__Bacteria	p__Abditibacteriota	c__Abditibacteria	o__Abditibacteriales	f__Abditibacteriaceae	g__Abditibacterium	s__uncultured_soil_bacterium_g__Abditibacterium	OTU851
d__Bacteria	k__norank_ d__Bacteria	p__Bacteroidota	c__Bacteroidia	o__Sphingobacteriales	f__env.OPS_17	g__norank_f__env.OPS_17	s__metagenome_g__norank_f__env.OPS_17	OTU1077
d__Bacteria	k__norank_ d__Bacteria	p__Bacteroidota	c__Bacteroidia	o__Chitinophagales	f__Chitinophagaceae	g__Edaphobaculum	s__uncultured_bacterium_g__Edaphobaculum	OTU1304
d__Bacteria	k__norank_ d__Bacteria	p__Gemmatimonadota	c__Gemmatimonadetes	o__Gemmatimonadales	f__Gemmatimonadaceae	g__norank_f__Gemmatimonadaceae	s__unclassified_g__norank_f__Gemmatimonadaceae	OTU1008
d__Bacteria	k__norank_ d__Bacteria	p__Verrucomicrobiota	c__Chlamydiae	o__Chlamydiales	f__Parachlamydiaceae	g__norank_f__Parachlamydiaceae	s__metagenome_g__norank_f__Parachlamydiaceae	OTU736
d__Bacteria	k__norank_ d__Bacteria	p__Bacteroidota	c__Bacteroidia	o__Cytophagales	f__Hymenobacteraceae	g__Adhaeribacter	s__uncultured_bacterium_g__Adhaeribacter	OTU2301
d__Bacteria	k__norank_ d__Bacteria	p__Myxococcota	c__Myxococcia	o__Myxococcales	f__Myxococcaceae	g__Archangium	s__Archangium_gephyra	OTU463
d__Bacteria	k__norank_ d__Bacteria	p__Bacteroidota	c__Bacteroidia	o__Cytophagales	f__Spirosomaceae	g__unclassified_f__Spirosomaceae	s__unclassified_f__Spirosomaceae	OTU189
d__Bacteria	k__norank_ d__Bacteria	p__Deinococcota	c__Deinococci	o__Deinococcales	f__Deinococcaceae	g__Deinococcus	s__unclassified_g__Deinococcus	OTU171
d__Bacteria	k__norank_ d__Bacteria	p__Bdellovibrionota	c__Bdellovibrionia	o__Bdellovibrionales	f__Bdellovibrionaceae	g__Bdellovibrio	s__metagenome_g__Bdellovibrio	OTU270
d__Bacteria	k__norank_ d__Bacteria	p__Acidobacteriota	c__Vicinamibacteria	o__Vicinamibacterales	f__Vicinamibacteraceae	g__norank_f__Vicinamibacteraceae	s__unclassified_g__norank_f__Vicinamibacteraceae	OTU2230

d__Bacteria	k__norank_ d__Bacteria	p__Proteobacteria	c__Gammaproteobacteria	o__Enterobacterales	f__Yersiniaceae	g__unclassified_f__Yersiniaceae	s__unclassified_f__Yersiniaceae	OTU114
d__Bacteria	k__norank_ d__Bacteria	p__Acidobacteriota	c__Acidobacteriae	o__Acidobacteriales	f__norank_o__Acidobacteriales	g__norank_f__norank_o__Acidobacteriales	s__uncultured_Acidobacteria_bacterium_g__norank_f__norank_o__Acidobacteriales	OTU1964
d__Bacteria	k__norank_ d__Bacteria	p__Proteobacteria	c__Gammaproteobacteria	o__Burkholderiales	f__Comamonadaceae	g__unclassified_f__Comamonadaceae	s__unclassified_f__Comamonadaceae	OTU1310
d__Bacteria	k__norank_ d__Bacteria	p__Patescibacteria	c__Saccharimonadia	o__Saccharimonadales	f__unclassified_o__Saccharimonadales	g__unclassified_o__Saccharimonadales	s__unclassified_o__Saccharimonadales	OTU570
d__Bacteria	k__norank_ d__Bacteria	p__Patescibacteria	c__Saccharimonadia	o__Saccharimonadales	f__norank_o__Saccharimonadales	g__norank_f__norank_o__Saccharimonadales	s__metal-contaminated_soil_clone_K20-12	OTU1368
d__Bacteria	k__norank_ d__Bacteria	p__Actinobacteriota	c__Acidimicrobiia	o__Microtrichales	f__norank_o__Microtrichales	g__norank_f__norank_o__Microtrichales	s__unclassified_g__norank_f__norank_o__Microtrichales	OTU2769
d__Bacteria	k__norank_ d__Bacteria	p__Proteobacteria	c__Gammaproteobacteria	o__Xanthomonadales	f__Rhodanobacteraceae	g__norank_f__Rhodanobacteraceae	s__unclassified_g__norank_f__Rhodanobacteraceae	OTU2098
d__Bacteria	k__norank_ d__Bacteria	p__Bacteroidota	c__Bacteroidia	o__Cytophagales	f__Spirosomaceae	g__Spirosoma	s__unclassified_g__Spirosoma	OTU2968
d__Bacteria	k__norank_ d__Bacteria	p__Proteobacteria	c__Gammaproteobacteria	o__Steroidobacterales	f__Steroidobacteraceae	g__norank_f__Steroidobacteraceae	s__metagenome_g__norank_f__Steroidobacteraceae	OTU108
d__Bacteria	k__norank_ d__Bacteria	p__Chloroflexi	c__Ktedonobacteria	o__Ktedonobacterales	f__Ktedonobacteraceae	g__JG30a-KF-32	s__unclassified_g__JG30a-KF-32	OTU1697
d__Bacteria	k__norank_ d__Bacteria	p__Myxococcota	c__Polyangia	o__Haliangiales	f__Haliangiaceae	g__Haliangium	s__uncultured_Myxococcales_bacterium_g__Haliangium	OTU1544
d__Bacteria	k__norank_ d__Bacteria	p__RCP2-54	c__norank_p__RCP2-54	o__norank_c__norank_p__RCP2-54	f__norank_o__norank_c__norank_p__RCP2-54	g__norank_f__norank_o__norank_c__norank_p__RCP2-54	s__unclassified_g__norank_f__norank_o__norank_c__norank_p__RCP2-54	OTU955
d__Bacteria	k__norank_ d__Bacteria	p__Proteobacteria	c__Alphaproteobacteria	o__Rhodobacterales	f__Rhodobacteraceae	g__Rubellimicrobium	s__uncultured_bacterium_g__Rubellimicrobium	OTU328
d__Bacteria	k__norank_ d__Bacteria	p__Gemmatimonadota	c__Gemmatimonadetes	o__Gemmatimonadales	f__Gemmatimonadaceae	g__Roseisolibacter	s__uncultured_bacterium_g__Roseisolibacter	OTU1442
d__Bacteria	k__norank_ d__Bacteria	p__Acidobacteriota	c__Vicinamibacteria	o__Vicinamibacterales	f__Vicinamibacteraceae	g__norank_f__Vicinamibacteraceae	s__uncultured_Acidobacteria_bacterium_g__norank_f__Vicinamibacteraceae	OTU1410
d__Bacteria	k__norank_ d__Bacteria	p__Bacteroidota	c__Bacteroidia	o__Chitinophagales	f__Saprospiraceae	g__norank_f__Saprospiraceae	s__uncultured_Bacteroidetes_bacterium_g__norank_f__Saprospiraceae	OTU495

d__Bacteria	k__norank_d__Bacteria	p__Chloroflexi	c__Chloroflexia	o__Thermomicrobiales	f__JG30-KF-CM45	g__norank_f__JG30-KF-CM45	s__unclassified_g__norank_f__JG30-KF-CM45	OTU2845
d__Bacteria	k__norank_d__Bacteria	p__Proteobacteria	c__Alphaproteobacteria	o__Rhizobiales	f__Amb-16S-1323	g__norank_f__Amb-16S-1323	s__uncultured_bacterium_g__norank_f__Amb-16S-1323	OTU1351
d__Bacteria	k__norank_d__Bacteria	p__Actinobacteriota	c__Actinobacteria	o__Corynebacteriales	f__Nocardiaceae	g__Nocardia	s__uncultured_bacterium_g__Nocardia	OTU80
d__Bacteria	k__norank_d__Bacteria	p__Patescibacteria	c__Saccharimonadia	o__Saccharimonadales	f__norank_o__Saccharimonadales	g__norank_f__norank_o__Saccharimonadales	s__wastewater_metagenome_g__norank_f__norank_o__Saccharimonadales	OTU1387
d__Bacteria	k__norank_d__Bacteria	p__Bacteroidota	c__Bacteroidia	o__Cytophagales	f__Microscillaceae	g__norank_f__Microscillaceae	s__uncultured_Ohtaekwangia_sp.	OTU2255
d__Bacteria	k__norank_d__Bacteria	p__Proteobacteria	c__Gammaproteobacteria	o__Diplorickettsiales	f__Diplorickettsiaceae	g__Aquicella	s__unclassified_g__Aquicella	OTU976
d__Bacteria	k__norank_d__Bacteria	p__Myxococcota	c__Myxococcia	o__Myxococcales	f__Anaeromyxobacteraceae	g__Anaeromyxobacter	s__uncultured_bacterium_g__Anaeromyxobacter	OTU2503
d__Bacteria	k__norank_d__Bacteria	p__Proteobacteria	c__Gammaproteobacteria	o__Cellvibrionales	f__Cellvibrionaceae	g__norank_f__Cellvibrionaceae	s__uncultured_bacterium_g__norank_f__Cellvibrionaceae	OTU2053
d__Bacteria	k__norank_d__Bacteria	p__Dependentiae	c__Babeliae	o__Babeliales	f__Vermiphilaceae	g__norank_f__Vermiphilaceae	s__uncultured_bacterium_g__norank_f__Vermiphilaceae	OTU2778
d__Bacteria	k__norank_d__Bacteria	p__Firmicutes	c__Clostridia	o__Peptostreptococcales-Tissierellales	f__norank_o__Peptostreptococcales-Tissierellales	g__Fenollaria	s__uncultured_bacterium_g__Fenollaria	OTU1188
d__Bacteria	k__norank_d__Bacteria	p__Acidobacteriota	c__Blastocatellia	o__Blastocatellales	f__Blastocatellaceae	g__Blastocatella	s__unclassified_g__Blastocatella	OTU2907
d__Bacteria	k__norank_d__Bacteria	p__Gemmatimonadota	c__Gemmatimonadetes	o__Gemmatimonadales	f__Gemmatimonadaceae	g__Gemmatimonas	s__uncultured_bacterium_g__Gemmatimonas	OTU250
d__Bacteria	k__norank_d__Bacteria	p__Proteobacteria	c__Alphaproteobacteria	o__Rhizobiales	f__Hyphomicrobiaceae	g__Hyphomicrobium	s__unclassified_g__Hyphomicrobium	OTU2910
d__Bacteria	k__norank_d__Bacteria	p__Acidobacteriota	c__Blastocatellia	o__Pyrinomonadales	f__Pyrinomonadaceae	g__RB41	s__metagenome_g__RB41	OTU1273
d__Bacteria	k__norank_d__Bacteria	p__Chloroflexi	c__Chloroflexia	o__Chloroflexales	f__Roseiflexaceae	g__Kouleothrix	s__uncultured_bacterium_g__Kouleothrix	OTU2851
d__Bacteria	k__norank_d__Bacteria	p__Fibrobacterota	c__Fibrobacteria	o__Fibrobacterales	f__Fibrobacteraceae	g__possible_genus_04	s__uncultured_bacterium_g__possible_genus_04	OTU1218

d__Bacteria	k__norank_d__Bacteria	p__WPS-2	c__norank_p__WPS-2	o__norank_c__norank_p__WPS-2	f__norank_o__norank_c__norank_p__WPS-2	g__norank_f__norank_o__norank_c__norank_p__WPS-2	s__metagenome_g__norank_f__norank_o__norank_c__norank_p__WPS-2	OTU467
d__Bacteria	k__norank_d__Bacteria	p__Proteobacteria	c__Alphaproteobacteria	o__Rickettsiales	f__Mitochondria	g__norank_f__Mitochondria	s__unclassified_g__norank_f__Mitochondria	OTU4
d__Bacteria	k__norank_d__Bacteria	p__Acidobacteriota	c__Acidobacteriae	o__Acidobacteriales	f__Acidobacteriaceae_Subgroup_1	g__norank_f__Acidobacteriaceae_Subgroup_1	s__uncultured_Acidobacteria_bacterium_g__norank_f__Acidobacteriaceae_Subgroup_1	OTU1066
d__Bacteria	k__norank_d__Bacteria	p__Gemmatimonadota	c__Gemmatimonadetes	o__Gemmatimonadales	f__Gemmatimonadaceae	g__Roseisolibacter	s__uncultured_soil_bacterium_g__Roseisolibacter	OTU2884
d__Bacteria	k__norank_d__Bacteria	p__Myxococcota	c__Polyangia	o__Polyangiales	f__BIrii41	g__norank_f__BIrii41	s__uncultured_bacterium_g__norank_f__BIrii41	OTU2643
d__Bacteria	k__norank_d__Bacteria	p__Proteobacteria	c__Alphaproteobacteria	o__Rhizobiales	f__Beijerinckiaceae	g__alphaI_cluster	s__uncultured_bacterium_g__alphaI_cluster	OTU1533
d__Bacteria	k__norank_d__Bacteria	p__Proteobacteria	c__Gammaproteobacteria	o__Burkholderiales	f__Burkholderiaceae	g__unclassified_f__Burkholderiaceae	s__unclassified_f__Burkholderiaceae	OTU1660
d__Bacteria	k__norank_d__Bacteria	p__Gemmatimonadota	c__S0134_terrestrial_group	o__norank_c__S0134_terrestrial_group	f__norank_o__norank_c__S0134_terrestrial_group	g__norank_f__norank_o__norank_c__S0134_terrestrial_group	s__uncultured_bacterium_g__norank_f__norank_o__norank_c__S0134_terrestrial_group	OTU1562
d__Bacteria	k__norank_d__Bacteria	p__Proteobacteria	c__Gammaproteobacteria	o__Burkholderiales	f__Comamonadaceae	g__unclassified_f__Comamonadaceae	s__unclassified_f__Comamonadaceae	OTU2065
d__Bacteria	k__norank_d__Bacteria	p__Proteobacteria	c__Gammaproteobacteria	o__Burkholderiales	f__Burkholderiaceae	g__Lautropia	s__unclassified_g__Lautropia	OTU2439
d__Bacteria	k__norank_d__Bacteria	p__Armatimonadota	c__Chthonomonadetes	o__Chthonomonadales	f__Chthonomonadaceae	g__Chthonomonas	s__uncultured_bacterium_g__Chthonomonas	OTU1962
d__Bacteria	k__norank_d__Bacteria	p__Bdellovibrionota	c__Oligoflexia	o__0319-6G20	f__norank_o__0319-6G20	g__norank_f__norank_o__0319-6G20	s__unclassified_g__norank_f__norank_o__0319-6G20	OTU617
d__Bacteria	k__norank_d__Bacteria	p__Actinobacteriota	c__Actinobacteria	o__Propionibacteriales	f__Nocardioidaceae	g__Nocardioides	s__unclassified_g__Nocardioides	OTU2846
d__Bacteria	k__norank_d__Bacteria	p__Chloroflexi	c__Chloroflexia	o__Chloroflexales	f__Chloroflexaceae	g__Oscillochloris	s__uncultured_Chloroflexi_bacterium_g__Oscillochloris	OTU2548
d__Bacteria	k__norank_d__Bacteria	p__Patescibacteria	c__norank_p__Patescibacteria	o__norank_c__norank_p__Patescibacteria	f__norank_o__norank_c__norank_p__Patescibacteria	g__norank_f__norank_o__norank_c__norank_p__Patescibacteria	s__uncultured_bacterium_g__norank_f__norank_o__norank_c__norank_p__Patescibacteria	OTU2472
d__Bacteria	k__norank_d__Bacteria	p__Acidobacteriota	c__Acidobacteriae	o__Bryobacterales	f__Bryobacteraceae	g__Bryobacter	s__unclassified_g__Bryobacter	OTU444

d__Bacteria	k__norank_ d__Bacteria	p__Proteobacteria	c__Gammaproteobacteria	o__Enterobacterales	f__unclassified_o__Enterobacterales	g__unclassified_o__Enterobacterales	s__unclassified_o__Enterobacterales	OTU9
d__Bacteria	k__norank_ d__Bacteria	p__Actinobacteriota	c__Acidimicrobiia	o__Microtrichales	f__Ilumatobacteraceae	g__norank_f__Ilumatobacteraceae	s__metagenome_g__norank_f__Ilumatobacteraceae	OTU2770
d__Bacteria	k__norank_ d__Bacteria	p__Armatimonadota	c__Armatimonadia	o__Armatimonadales	f__norank_o__Armatimonadales	g__norank_f__norank_o__Armatimona dales	s__unclassified_g__norank_f__norank_o__Armatimonadales	OTU924
d__Bacteria	k__norank_ d__Bacteria	p__Proteobacteria	c__Gammaproteobacteria	o__Burkholderiales	f__Nitrosomonadaceae	g__MND1	s__unclassified_g__MND1	OTU491
d__Bacteria	k__norank_ d__Bacteria	p__Armatimonadota	c__Fimbriimonadia	o__Fimbriimonadales	f__Fimbriimonadaceae	g__Fimbriimonas	s__unclassified_g__Fimbriimonas	OTU2479
d__Bacteria	k__norank_ d__Bacteria	p__Myxococcota	c__Myxococcia	o__Myxococcales	f__unclassified_o__Myxococcales	g__unclassified_o__Myxococcales	s__unclassified_o__Myxococcales	OTU322
d__Bacteria	k__norank_ d__Bacteria	p__Patescibacteria	c__Saccharimonadia	o__Saccharimonadales	f__LWQ8	g__norank_f__LWQ8	s__uncultured_bacterium_g__norank_f__LWQ8	OTU2777
d__Bacteria	k__norank_ d__Bacteria	p__Bacteroidota	c__Bacteroidia	o__Cytophagales	f__Microscillaceae	g__Ohtaekwangia	s__metagenome_g__Ohtaekwangia	OTU2660
d__Bacteria	k__norank_ d__Bacteria	p__Patescibacteria	c__Saccharimonadia	o__Saccharimonadales	f__norank_o__Saccharimonadales	g__norank_f__norank_o__Saccharimo nadales	s__unclassified_g__norank_f__norank_o__Saccharimonadales	OTU581
d__Bacteria	k__norank_ d__Bacteria	p__Patescibacteria	c__Saccharimonadia	o__Saccharimonadales	f__norank_o__Saccharimonadales	g__norank_f__norank_o__Saccharimo nadales	s__unclassified_g__norank_f__norank_o__Saccharimonadales	OTU583
d__Bacteria	k__norank_ d__Bacteria	p__Gemmatimonadota	c__Gemmatimonadetes	o__Gemmatimonadales	f__Gemmatimonadaceae	g__unclassified_f__Gemmatimonadace ae	s__unclassified_f__Gemmatimonadaceae	OTU2720
d__Bacteria	k__norank_ d__Bacteria	p__Proteobacteria	c__Gammaproteobacteria	o__Burkholderiales	f__Oxalobacteraceae	g__Massilia	s__uncultured_bacterium_g__Massilia	OTU2403
d__Bacteria	k__norank_ d__Bacteria	p__Proteobacteria	c__Gammaproteobacteria	o__Burkholderiales	f__Nitrosomonadaceae	g__Ellin6067	s__uncultured_bacterium_g__Ellin6067	OTU2375
d__Bacteria	k__norank_ d__Bacteria	p__Patescibacteria	c__Berkelbacteria	o__norank_c__Berkelbacteri a	f__norank_o__norank_c__Berkelbacteria	g__norank_f__norank_o__norank_c__ Berkelbacteria	s__unclassified_g__norank_f__norank_o__norank_c__Berkelbacteria	OTU266
d__Bacteria	k__norank_ d__Bacteria	p__Actinobacteriota	c__Acidimicrobiia	o__Microtrichales	f__Ilumatobacteraceae	g__norank_f__Ilumatobacteraceae	s__unclassified_g__norank_f__Ilumatobacteraceae	OTU1467
d__Bacteria	k__norank_ d__Bacteria	p__Actinobacteriota	c__Actinobacteria	o__Propionibacteriales	f__Propionibacteriaceae	g__Friedmanniella	s__uncultured_bacterium_g__Friedmanniella	OTU2380

d__Bacteria	k__norank_ d__Bacteria	p__Chloroflexi	c__Chloroflexia	o__Chloroflexales	f__Roseiflexaceae	g__norank_f__Roseiflexaceae	s__unclassified_g__norank_f__Roseiflexaceae	OTU2227
d__Bacteria	k__norank_ d__Bacteria	p__Patescibacteria	c__Saccharimonadia	o__Saccharimonadales	f__WWH38	g__norank_f__WWH38	s__uncultured_soil_bacterium_g__norank_f__WWH38	OTU2550
d__Bacteria	k__norank_ d__Bacteria	p__Proteobacteria	c__Gammaproteobacteria	o__Diplorickettsiales	f__Diplorickettsiaceae	g__norank_f__Diplorickettsiaceae	s__unclassified_g__norank_f__Diplorickettsiaceae	OTU1741
d__Bacteria	k__norank_ d__Bacteria	p__Patescibacteria	c__Saccharimonadia	o__Saccharimonadales	f__norank_o__Saccharimonadales	g__norank_f__norank_o__Saccharimo nadales	s__unclassified_g__norank_f__norank_o__Saccharimonadales	OTU459
d__Bacteria	k__norank_ d__Bacteria	p__Chloroflexi	c__Chloroflexia	o__Thermomicrobiales	f__AKYG1722	g__norank_f__AKYG1722	s__uncultured_bacterium_g__norank_f__AKYG1722	OTU1573
d__Bacteria	k__norank_ d__Bacteria	p__Planctomycetota	c__Planctomycetes	o__Isosphaerales	f__Isosphaeraceae	g__unclassified_f__Isosphaeraceae	s__unclassified_f__Isosphaeraceae	OTU68
d__Bacteria	k__norank_ d__Bacteria	p__Proteobacteria	c__Alphaproteobacteria	o__Rhodobacterales	f__Rhodobacteraceae	g__Rubellimicrobium	s__uncultured_bacterium_g__Rubellimicrobium	OTU2293
d__Bacteria	k__norank_ d__Bacteria	p__Nitrospirota	c__Nitrospiria	o__Nitrospirales	f__Nitrospiraceae	g__Nitrospira	s__unclassified_g__Nitrospira	OTU1529
d__Bacteria	k__norank_ d__Bacteria	p__Proteobacteria	c__Gammaproteobacteria	o__Burkholderiales	f__Comamonadaceae	g__Polaromonas	s__unclassified_g__Polaromonas	OTU461
d__Bacteria	k__norank_ d__Bacteria	p__Deinococcota	c__Deinococci	o__Deinococcales	f__Deinococcaceae	g__Deinococcus	s__Deinococcus_sp.	OTU140
d__Bacteria	k__norank_ d__Bacteria	p__Acidobacteriota	c__Blastocatellia	o__DS-100	f__norank_o__DS-100	g__norank_f__norank_o__DS-100	s__unclassified_g__norank_f__norank_o__DS-100	OTU1298
d__Bacteria	k__norank_ d__Bacteria	p__Proteobacteria	c__Alphaproteobacteria	o__Caulobacterales	f__Caulobacteraceae	g__norank_f__Caulobacteraceae	s__uncultured_Caulobacter_sp._g__norank	OTU2225
d__Bacteria	k__norank_ d__Bacteria	p__Proteobacteria	c__Alphaproteobacteria	o__Rhizobiales	f__Beijerinckiaceae	g__norank_f__Beijerinckiaceae	s__uncultured_bacterium_g__norank_f__Beijerinckiaceae	OTU2170
d__Bacteria	k__norank_ d__Bacteria	p__Actinobacteriota	c__MB-A2-108	o__norank_c__MB-A2-108	f__norank_o__norank_c__MB-A2-108	g__norank_f__norank_o__norank_c__ MB-A2-108	s__uncultured_Catenulispora_sp.	OTU1462
d__Bacteria	k__norank_ d__Bacteria	p__Chloroflexi	c__Ktedonobacteria	o__Ktedonobacterales	f__Ktedonobacteraceae	g__G12-WMSP1	s__uncultured_Chloroflexi_bacterium_g__G12-WMSP1	OTU1749
d__Bacteria	k__norank_ d__Bacteria	p__Gemmatimonadota	c__Gemmatimonadetes	o__Gemmatimonadales	f__Gemmatimonadaceae	g__Roseisolibacter	s__Roseisolibacter_agri	OTU2157

d__Bacteria	k__norank_ d__Bacteria	p__Bacteroidota	c__Bacteroidia	o__Flavobacteriales	f__Weeksellaceae	g__Chryseobacterium	s__unclassified_g__Chryseobacterium	OTU2826
d__Bacteria	k__norank_ d__Bacteria	p__Actinobacteriota	c__Actinobacteria	o__Frankiales	f__Geodermatophilaceae	g__Antricoccus	s__uncultured_bacterium_g__Antricoccus	OTU2180
d__Bacteria	k__norank_ d__Bacteria	p__Desulfobacterota	c__Desulfuromonadia	o__Geobacterales	f__Geobacteraceae	g__Geobacter	s__uncultured_delta_proteobacterium_g__Geobacter	OTU2041
d__Bacteria	k__norank_ d__Bacteria	p__Bdellovibrionota	c__Oligoflexia	o__Oligoflexales	f__norank_o__Oligoflexales	g__norank_f__norank_o__Oligoflexale s	s__unclassified_g__norank_f__norank_o__Oligoflexales	OTU30
d__Bacteria	k__norank_ d__Bacteria	p__Chloroflexi	c__Dehalococcoidia	o__S085	f__norank_o__S085	g__norank_f__norank_o__S085	s__metagenome_g__norank_f__norank_o__S085	OTU2124
d__Bacteria	k__norank_ d__Bacteria	p__Actinobacteriota	c__Acidimicrobiia	o__Microtrichales	f__Ilumatobacteraceae	g__norank_f__Ilumatobacteraceae	s__uncultured_bacterium_g__norank_f__Ilumatobacteraceae	OTU365
d__Bacteria	k__norank_ d__Bacteria	p__Proteobacteria	c__Alphaproteobacteria	o__Rhizobiales	f__Rhizobiales_Incertae_Sedis	g__Phreatobacter	s__unclassified_g__Phreatobacter	OTU2923
d__Bacteria	k__norank_ d__Bacteria	p__Proteobacteria	c__Alphaproteobacteria	o__Rhizobiales	f__Methylopilaceae	g__Hansschlegelia	s__Hansschlegelia_plantiphila	OTU2382
d__Bacteria	k__norank_ d__Bacteria	p__Acidobacteriota	c__Vicinamibacteria	o__Vicinamibacterales	f__norank_o__Vicinamibacterales	g__norank_f__norank_o__Vicinamiba cterales	s__metagenome_g__norank_f__norank_o__Vicinamibacterales	OTU180
d__Bacteria	k__norank_ d__Bacteria	p__Planctomycetota	c__Planctomycetes	o__Isosphaerales	f__Isosphaeraceae	g__norank_f__Isosphaeraceae	s__unclassified_g__norank_f__Isosphaeraceae	OTU65
d__Bacteria	k__norank_ d__Bacteria	p__Planctomycetota	c__Planctomycetes	o__Isosphaerales	f__Isosphaeraceae	g__norank_f__Isosphaeraceae	s__unclassified_g__norank_f__Isosphaeraceae	OTU62
d__Bacteria	k__norank_ d__Bacteria	p__Bacteroidota	c__Bacteroidia	o__Cytophagales	f__Spirosomaceae	g__norank_f__Spirosomaceae	s__metagenome_g__norank_f__Spirosomaceae	OTU1419
d__Bacteria	k__norank_ d__Bacteria	p__Bacteroidota	c__Bacteroidia	o__Chitinophagales	f__Chitinophagaceae	g__Ferruginibacter	s__uncultured_bacterium_g__Ferruginibacter	OTU476
d__Bacteria	k__norank_ d__Bacteria	p__Chloroflexi	c__unclassified_p__Chlor oflexi	o__unclassified_p__Chlorofl exi	f__unclassified_p__Chloroflexi	g__unclassified_p__Chloroflexi	s__unclassified_p__Chloroflexi	OTU1240
d__Bacteria	k__norank_ d__Bacteria	p__Chloroflexi	c__Ktedonobacteria	o__Ktedonobacterales	f__Ktedonobacteraceae	g__FCPS473	s__uncultured_bacterium_g__FCPS473	OTU2897
d__Bacteria	k__norank_ d__Bacteria	p__Proteobacteria	c__Alphaproteobacteria	o__Zavarziniales	f__norank_o__Zavarziniales	g__norank_f__norank_o__Zavarziniale s	s__metagenome_g__norank_f__norank_o__Zavarziniales	OTU2338

d__Bacteria	k__norank_ d__Bacteria	p__Proteobacteria	c__Gammaproteobacteria	o__Burkholderiales	f__Oxalobacteraceae	g__unclassified_f__Oxalobacteraceae	s__unclassified_f__Oxalobacteraceae	OTU384
d__Bacteria	k__norank_ d__Bacteria	p__Proteobacteria	c__Alphaproteobacteria	o__Elsterales	f__norank_o__Elsterales	g__norank_f__norank_o__Elsterales	s__unclassified_g__norank_f__norank_o__Elsterales	OTU1908
d__Bacteria	k__norank_ d__Bacteria	p__Acidobacteriota	c__Vicinamibacteria	o__Vicinamibacterales	f__norank_o__Vicinamibacterales	g__norank_f__norank_o__Vicinamiba cterales	s__unclassified_g__norank_f__norank_o__Vicinamibacterales	OTU1392
d__Bacteria	k__norank_ d__Bacteria	p__Proteobacteria	c__Gammaproteobacteria	o__Burkholderiales	f__Nitrosomonadaceae	g__Nitrosospira	s__uncultured_bacterium_g__Nitrosospira	OTU1341
d__Bacteria	k__norank_ d__Bacteria	p__Proteobacteria	c__Gammaproteobacteria	o__Xanthomonadales	f__Xanthomonadaceae	g__Vulcaniibacterium	s__uncultured_bacterium_g__Vulcaniibacterium	OTU109
d__Bacteria	k__norank_ d__Bacteria	p__Acidobacteriota	c__Vicinamibacteria	o__Vicinamibacterales	f__norank_o__Vicinamibacterales	g__norank_f__norank_o__Vicinamiba cterales	s__unclassified_g__norank_f__norank_o__Vicinamibacterales	OTU1395
d__Bacteria	k__norank_ d__Bacteria	p__Armatimonadota	c__Chthonomonadetes	o__Chthonomonadales	f__Chthonomonadaceae	g__Chthonomonas	s__uncultured_bacterium_g__Chthonomonas	OTU710
d__Bacteria	k__norank_ d__Bacteria	p__Proteobacteria	c__Alphaproteobacteria	o__Acetobacterales	f__Acetobacteraceae	g__norank_f__Acetobacteraceae	s__uncultured_proteobacterium_g__norank_f__Acetobacteraceae	OTU1061
d__Bacteria	k__norank_ d__Bacteria	p__Actinobacteriota	c__Thermoleophilia	o__Solirubrobacterales	f__Solirubrobacteraceae	g__Conexibacter	s__uncultured_bacterium_g__Conexibacter	OTU2402
d__Bacteria	k__norank_ d__Bacteria	p__Acidobacteriota	c__Holophagae	o__Subgroup_7	f__norank_o__Subgroup_7	g__norank_f__norank_o__Subgroup_7	s__unclassified_g__norank_f__norank_o__Subgroup_7	OTU2385
d__Bacteria	k__norank_ d__Bacteria	p__Proteobacteria	c__Alphaproteobacteria	o__Rhodobacterales	f__Rhodobacteraceae	g__Pseudorhodobacter	s__unclassified_g__Pseudorhodobacter	OTU2707
d__Bacteria	k__norank_ d__Bacteria	p__Sumerlaeota	c__Sumerlaeia	o__Sumerlaeales	f__Sumerlaeaceae	g__Sumerlaea	s__unclassified_g__Sumerlaea	OTU2806
d__Bacteria	k__norank_ d__Bacteria	p__Acidobacteriota	c__Acidobacteriae	o__Acidobacteriales	f__Acidobacteriaceae_Subgroup_1	g__Acidicapsa	s__unclassified_g__Acidicapsa	OTU1737
d__Bacteria	k__norank_ d__Bacteria	p__Myxococcota	c__Myxococcia	o__Myxococcales	f__Myxococcaceae	g__unclassified_f__Myxococcaceae	s__unclassified_f__Myxococcaceae	OTU1815
d__Bacteria	k__norank_ d__Bacteria	p__Proteobacteria	c__Alphaproteobacteria	o__Sphingomonadales	f__Sphingomonadaceae	g__Sphingomonas	s__Sphingomonas_faeni	OTU1895
d__Bacteria	k__norank_ d__Bacteria	p__Patescibacteria	c__Saccharimonadia	o__Saccharimonadales	f__unclassified_o__Saccharimonadales	g__unclassified_o__Saccharimonadale s	s__unclassified_o__Saccharimonadales	OTU168

d__Bacteria	k__norank_d__Bacteria	p__Actinobacteriota	c__Acidimicrobiia	o__IMCC26256	f__norank_o__IMCC26256	g__norank_f__norank_o__IMCC26256	s__unclassified_g__norank_f__norank_o__IMCC26256	OTU1266
d__Bacteria	k__norank_d__Bacteria	p__Bacteroidota	c__Bacteroidia	o__Cytophagales	f__Microscillaceae	g__norank_f__Microscillaceae	s__uncultured_soil_bacterium_g__norank_f__Microscillaceae	OTU2059
d__Bacteria	k__norank_d__Bacteria	p__Bacteroidota	c__Bacteroidia	o__Sphingobacteriales	f__KD3-93	g__norank_f__KD3-93	s__uncultured_bacterium_g__norank_f__KD3-93	OTU2866
d__Bacteria	k__norank_d__Bacteria	p__Chloroflexi	c__Anaerolineae	o__SBR1031	f__norank_o__SBR1031	g__norank_f__norank_o__SBR1031	s__unclassified_g__norank_f__norank_o__SBR1031	OTU1494
d__Bacteria	k__norank_d__Bacteria	p__Armatimonadota	c__norank_p__Armatimonadota	o__norank_c__norank_p__Armatimonadota	f__norank_o__norank_c__norank_p__Armatimonadota	g__norank_f__norank_o__norank_c__norank_p__Armatimonadota	s__uncultured_bacterium_g__norank_f__norank_o__norank_c__norank_p__Armatimonadota	OTU1930
d__Bacteria	k__norank_d__Bacteria	p__Bdellovibrionota	c__Bdellovibrionia	o__Bdellovibrionales	f__Bdellovibrionaceae	g__OM27_clade	s__uncultured_bacterium_g__OM27_clade	OTU2111
d__Bacteria	k__norank_d__Bacteria	p__Bacteroidota	c__Bacteroidia	o__Chitinophagales	f__Saprospiraceae	g__norank_f__Saprospiraceae	s__unclassified_g__norank_f__Saprospiraceae	OTU628
d__Bacteria	k__norank_d__Bacteria	p__Gemmatimonadota	c__Gemmatimonadetes	o__Gemmatimonadales	f__Gemmatimonadaceae	g__norank_f__Gemmatimonadaceae	s__Gemmatimonadetes_bacterium_LX87	OTU2421
d__Bacteria	k__norank_d__Bacteria	p__Deinococcota	c__Deinococci	o__Deinococcales	f__Deinococcaceae	g__Deinococcus	s__uncultured_bacterium_g__Deinococcus	OTU2767
d__Bacteria	k__norank_d__Bacteria	p__Bacteroidota	c__Bacteroidia	o__Cytophagales	f__Hymenobacteraceae	g__Hymenobacter	s__unclassified_g__Hymenobacter	OTU2014
d__Bacteria	k__norank_d__Bacteria	p__Chloroflexi	c__Chloroflexia	o__unclassified_c__Chloroflexia	f__unclassified_c__Chloroflexia	g__unclassified_c__Chloroflexia	s__unclassified_c__Chloroflexia	OTU781
d__Bacteria	k__norank_d__Bacteria	p__Bacteroidota	c__Bacteroidia	o__Cytophagales	f__Microscillaceae	g__norank_f__Microscillaceae	s__uncultured_Sphingobacteriales_bacterium_g__norank_f__Microscillaceae	OTU2358
d__Bacteria	k__norank_d__Bacteria	p__Patescibacteria	c__Saccharimonadia	o__Saccharimonadales	f__norank_o__Saccharimonadales	g__norank_f__norank_o__Saccharimonadales	s__uncultured_bacterium_g__norank_f__norank_o__Saccharimonadales	OTU1719
d__Bacteria	k__norank_d__Bacteria	p__Proteobacteria	c__Gammaproteobacteria	o__Gammaproteobacteria_Incertae_Sedis	f__unclassified_o__Gammaproteobacteria_Incertae_Sedis	g__Acidibacter	s__unclassified_g__Acidibacter	OTU1294
d__Bacteria	k__norank_d__Bacteria	p__Proteobacteria	c__Gammaproteobacteria	o__Burkholderiales	f__A21b	g__norank_f__A21b	s__uncultured_bacterium_g__norank_f__A21b	OTU2407
d__Bacteria	k__norank_d__Bacteria	p__Planctomycetota	c__Planctomycetes	o__Isosphaerales	f__Isosphaeraceae	g__norank_f__Isosphaeraceae	s__uncultured_bacterium_g__norank_f__Isosphaeraceae	OTU1025

d__Bacteria	k__norank_ d__Bacteria	p__Chloroflexi	c__Anaerolineae	o__Caldilineales	f__Caldilineaceae	g__norank_f__Caldilineaceae	s__uncultured_bacterium_g__norank_f__Caldilineaceae	OTU2071
d__Bacteria	k__norank_ d__Bacteria	p__Acidobacteriota	c__Subgroup_22	o__norank_c__Subgroup_22	f__norank_o__norank_c__Subgroup_22	g__norank_f__norank_o__norank_c__ Subgroup_22	s__unclassified_g__norank_f__norank_o__norank_c__Subgroup_22	OTU1384
d__Bacteria	k__norank_ d__Bacteria	p__Chloroflexi	c__Ktedonobacteria	o__Ktedonobacterales	f__Ktedonobacteraceae	g__JG30a-KF-32	s__uncultured_bacterium_g__JG30a-KF-32	OTU2692
d__Bacteria	k__norank_ d__Bacteria	p__Proteobacteria	c__Alphaproteobacteria	o__Rickettsiales	f__Mitochondria	g__norank_f__Mitochondria	s__unclassified_g__norank_f__Mitochondria	OTU1698
d__Bacteria	k__norank_ d__Bacteria	p__Bacteroidota	c__Bacteroidia	o__Cytophagales	f__Spirosomaceae	g__Spirosoma	s__Spirosoma_rigui	OTU338
d__Bacteria	k__norank_ d__Bacteria	p__unclassified_k__no rank_d__Bacteria	c__unclassified_k__noran k_d__Bacteria	o__unclassified_k__norank_ d__Bacteria	f__unclassified_k__norank_d__Bacteria	g__unclassified_k__norank_d__Bacteri a	s__unclassified_k__norank_d__Bacteria	OTU535
d__Bacteria	k__norank_ d__Bacteria	p__Proteobacteria	c__Gammaproteobacteria	o__Diplorickettsiales	f__Diplorickettsiaceae	g__norank_f__Diplorickettsiaceae	s__metagenome_g__norank_f__Diplorickettsiaceae	OTU433
d__Bacteria	k__norank_ d__Bacteria	p__unclassified_k__no rank_d__Bacteria	c__unclassified_k__noran k_d__Bacteria	o__unclassified_k__norank_ d__Bacteria	f__unclassified_k__norank_d__Bacteria	g__unclassified_k__norank_d__Bacteri a	s__unclassified_k__norank_d__Bacteria	OTU536
d__Bacteria	k__norank_ d__Bacteria	p__Actinobacteriota	c__Actinobacteria	o__Frankiales	f__unclassified_o__Frankiales	g__unclassified_o__Frankiales	s__unclassified_o__Frankiales	OTU2669
d__Bacteria	k__norank_ d__Bacteria	p__Actinobacteriota	c__Actinobacteria	o__Frankiales	f__Geodermatophilaceae	g__unclassified_f__Geodermatophilace ae	s__unclassified_f__Geodermatophilaceae	OTU2757
d__Bacteria	k__norank_ d__Bacteria	p__Acidobacteriota	c__Thermoanaerobaculia	o__Thermoanaerobaculales	f__Thermoanaerobaculaceae	g__Subgroup_10	s__unclassified_g__Subgroup_10	OTU383
d__Bacteria	k__norank_ d__Bacteria	p__Myxococcota	c__Myxococcia	o__Myxococcales	f__27F-1492R	g__norank_f__27F-1492R	s__uncultured_bacterium_g__norank_f__27F-1492R	OTU1161
d__Bacteria	k__norank_ d__Bacteria	p__Sumerlaeota	c__Sumerlaeia	o__Sumerlaeales	f__Sumerlaeaceae	g__Sumerlaea	s__uncultured_Acidobacteria_bacterium_g__Sumerlaea	OTU1430
d__Bacteria	k__norank_ d__Bacteria	p__Proteobacteria	c__Alphaproteobacteria	o__Rickettsiales	f__Mitochondria	g__norank_f__Mitochondria	s__unclassified_g__norank_f__Mitochondria	OTU90
d__Bacteria	k__norank_ d__Bacteria	p__Actinobacteriota	c__Actinobacteria	o__Propionibacteriales	f__Nocardioidaceae	g__Nocardioides	s__uncultured_bacterium_g__Nocardioides	OTU1598
d__Bacteria	k__norank_ d__Bacteria	p__Bacteroidota	c__Bacteroidia	o__Chitinophagales	f__Chitinophagaceae	g__norank_f__Chitinophagaceae	s__metagenome_g__norank_f__Chitinophagaceae	OTU2045

d__Bacteria	k__norank_d__Bacteria	p__Proteobacteria	c__Gammaproteobacteria	o__Burkholderiales	f__Rhodocyclaceae	g__norank_f__Rhodocyclaceae	s__uncultured_bacterium_g__norank_f__Rhodocyclaceae	OTU1754
d__Bacteria	k__norank_d__Bacteria	p__Proteobacteria	c__Alphaproteobacteria	o__Rhizobiales	f__Rhizobiaceae	g__Aliihoeflea	s__uncultured_Aliihoeflea_sp.	OTU2276
d__Bacteria	k__norank_d__Bacteria	p__Actinobacteriota	c__Actinobacteria	o__Micromonosporales	f__Micromonosporaceae	g__unclassified_f__Micromonosporaceae	s__unclassified_f__Micromonosporaceae	OTU2364
d__Bacteria	k__norank_d__Bacteria	p__Chloroflexi	c__JG30-KF-CM66	o__norank_c__JG30-KF-CM66	f__norank_o__norank_c__JG30-KF-CM66	g__norank_f__norank_o__norank_c__JG30-KF-CM66	s__uncultured_Caldilinea_sp._g__norank	OTU14
d__Bacteria	k__norank_d__Bacteria	p__Chloroflexi	c__Ktedonobacteria	o__Ktedonobacterales	f__Ktedonobacteraceae	g__1921-2	s__unclassified_g__1921-2	OTU1661
d__Bacteria	k__norank_d__Bacteria	p__Proteobacteria	c__Alphaproteobacteria	o__Rhizobiales	f__Hyphomicrobiaceae	g__Hyphomicrobium	s__unclassified_g__Hyphomicrobium	OTU377
d__Bacteria	k__norank_d__Bacteria	p__Actinobacteriota	c__Thermoleophilia	o__Gaiellales	f__norank_o__Gaiellales	g__norank_f__norank_o__Gaiellales	s__unclassified_g__norank_f__norank_o__Gaiellales	OTU1636
d__Bacteria	k__norank_d__Bacteria	p__Acidobacteriota	c__Vicinamibacteria	o__Vicinamibacterales	f__norank_o__Vicinamibacterales	g__norank_f__norank_o__Vicinamibacterales	s__unclassified_g__norank_f__norank_o__Vicinamibacterales	OTU821
d__Bacteria	k__norank_d__Bacteria	p__WS2	c__norank_p__WS2	o__norank_c__norank_p__WS2	f__norank_o__norank_c__norank_p__WS2	g__norank_f__norank_o__norank_c__norank_p__WS2	s__uncultured_soil_bacterium_g__norank_f__norank_o__norank_c__norank_p__WS2	OTU1303
d__Bacteria	k__norank_d__Bacteria	p__Proteobacteria	c__Gammaproteobacteria	o__Burkholderiales	f__Nitrosomonadaceae	g__Ellin6067	s__unclassified_g__Ellin6067	OTU2186
d__Bacteria	k__norank_d__Bacteria	p__Gemmatimonadota	c__Gemmatimonadetes	o__Gemmatimonadales	f__Gemmatimonadaceae	g__Gemmatimonas	s__unclassified_g__Gemmatimonas	OTU2589
d__Bacteria	k__norank_d__Bacteria	p__Proteobacteria	c__Gammaproteobacteria	o__Burkholderiales	f__TRA3-20	g__norank_f__TRA3-20	s__unclassified_g__norank_f__TRA3-20	OTU2460
d__Bacteria	k__norank_d__Bacteria	p__Acidobacteriota	c__Vicinamibacteria	o__Vicinamibacterales	f__Vicinamibacteraceae	g__norank_f__Vicinamibacteraceae	s__uncultured_Acidobacteria_bacterium_g__norank_f__Vicinamibacteraceae	OTU2309
d__Bacteria	k__norank_d__Bacteria	p__Proteobacteria	c__Alphaproteobacteria	o__Rhizobiales	f__Beijerinckiaceae	g__1174-901-12	s__uncultured_bacterium_g__1174-901-12	OTU1150
d__Bacteria	k__norank_d__Bacteria	p__Proteobacteria	c__Gammaproteobacteria	o__Burkholderiales	f__Comamonadaceae	g__Acidovorax	s__unclassified_g__Acidovorax	OTU222
d__Bacteria	k__norank_d__Bacteria	p__Chloroflexi	c__Anaerolineae	o__RBG-13-54-9	f__norank_o__RBG-13-54-9	g__norank_f__norank_o__RBG-13-54-9	s__unclassified_g__norank_f__norank_o__RBG-13-54-9	OTU1504

d__Bacteria	k__norank_ d__Bacteria	p__Myxococcota	c__bacteriap25	o__norank_c__bacteriap25	f__norank_o__norank_c__bacteriap25	g__norank_f__norank_o__norank_c__ bacteriap25	s__uncultured_Syntrophobacterales_bacterium_g__norank_f__norank	OTU2376
d__Bacteria	k__norank_ d__Bacteria	p__Proteobacteria	c__Alphaproteobacteria	o__Sneathiellales	f__Sneathiellaceae	g__norank_f__Sneathiellaceae	s__uncultured_bacterium_g__norank_f__Sneathiellaceae	OTU2585
d__Bacteria	k__norank_ d__Bacteria	p__Patescibacteria	c__Parcubacteria	o__norank_c__Parcubacteria	f__norank_o__norank_c__Parcubacteria	g__norank_f__norank_o__norank_c__ Parcubacteria	s__uncultured_bacterium_g__norank_f__norank_o__norank_c__Parcubacteria	OTU1269
d__Bacteria	k__norank_ d__Bacteria	p__Actinobacteriota	c__Acidimicrobiia	o__Microtrichales	f__Ilumatobacteraceae	g__unclassified_f__Ilumatobacteraceae	s__unclassified_f__Ilumatobacteraceae	OTU691
d__Bacteria	k__norank_ d__Bacteria	p__Verrucomicrobiota	c__Verrucomicrobiae	o__Opitutales	f__Opitutaceae	g__Lacunisphaera	s__unclassified_g__Lacunisphaera	OTU2315
d__Bacteria	k__norank_ d__Bacteria	p__Acidobacteriota	c__Vicinamibacteria	o__Vicinamibacterales	f__Vicinamibacteraceae	g__norank_f__Vicinamibacteraceae	s__unclassified_g__norank_f__Vicinamibacteraceae	OTU2058
d__Bacteria	k__norank_ d__Bacteria	p__Chloroflexi	c__Chloroflexia	o__Chloroflexales	f__Chloroflexaceae	g__unclassified_f__Chloroflexaceae	s__unclassified_f__Chloroflexaceae	OTU2248
d__Bacteria	k__norank_ d__Bacteria	p__Proteobacteria	c__Alphaproteobacteria	o__Reyranellales	f__Reyranellaceae	g__norank_f__Reyranellaceae	s__uncultured_Alphaproteobacteria_bacterium_g__norank_f__Reyranellaceae	OTU438
d__Bacteria	k__norank_ d__Bacteria	p__Deinococcota	c__Deinococci	o__Deinococcales	f__Deinococcaceae	g__Deinococcus	s__uncultured_bacterium_g__Deinococcus	OTU2563
d__Bacteria	k__norank_ d__Bacteria	p__Armatimonadota	c__norank_p__Armatimon adota	o__norank_c__norank_p__ Armatimonadota	f__norank_o__norank_c__norank_p__Ar matimonadota	g__norank_f__norank_o__norank_c__ norank_p__Armatimonadota	s__uncultured_bacterium_g__norank_f__norank_o__norank_c__norank_p__Armati monadota	OTU1237
d__Bacteria	k__norank_ d__Bacteria	p__Acidobacteriota	c__Acidobacteriae	o__Acidobacteriales	f__Acidobacteriaceae_Subgroup_1	g__Bryocella	s__uncultured_bacterium_g__Bryocella	OTU61
d__Bacteria	k__norank_ d__Bacteria	p__Proteobacteria	c__Alphaproteobacteria	o__Acetobacterales	f__Acetobacteraceae	g__norank_f__Acetobacteraceae	s__uncultured_bacterium_g__norank_f__Acetobacteraceae	OTU1198
d__Bacteria	k__norank_ d__Bacteria	p__Chloroflexi	c__Dehalococcoidia	o__S085	f__norank_o__S085	g__norank_f__norank_o__S085	s__metagenome_g__norank_f__norank_o__S085	OTU1375
d__Bacteria	k__norank_ d__Bacteria	p__Actinobacteriota	c__Thermoleophilia	o__Solirubrobacterales	f__Solirubrobacteraceae	g__unclassified_f__Solirubrobacterace ae	s__unclassified_f__Solirubrobacteraceae	OTU533
d__Bacteria	k__norank_ d__Bacteria	p__Bacteroidota	c__Bacteroidia	o__Chitinophagales	f__unclassified_o__Chitinophagales	g__unclassified_o__Chitinophagales	s__unclassified_o__Chitinophagales	OTU223
d__Bacteria	k__norank_ d__Bacteria	p__Proteobacteria	c__Alphaproteobacteria	o__Rhodobacterales	f__Rhodobacteraceae	g__norank_f__Rhodobacteraceae	s__unclassified_g__norank_f__Rhodobacteraceae	OTU2904

d__Bacteria	k__norank_ d__Bacteria	p__Chloroflexi	c__Anaerolineae	o__SBR1031	f__A4b	g__norank_f__A4b	s__unclassified_g__norank_f__A4b	OTU2042
d__Bacteria	k__norank_ d__Bacteria	p__Proteobacteria	c__Gammaproteobacteria	o__Diplorickettsiales	f__Diplorickettsiaceae	g__Aquicella	s__uncultured_bacterium_g__Aquicella	OTU1495
d__Bacteria	k__norank_ d__Bacteria	p__Proteobacteria	c__Gammaproteobacteria	o__Diplorickettsiales	f__Diplorickettsiaceae	g__norank_f__Diplorickettsiaceae	s__uncultured_bacterium_g__norank_f__Diplorickettsiaceae	OTU2776
d__Bacteria	k__norank_ d__Bacteria	p__Cyanobacteria	c__Cyanobacteriia	o__Chloroplast	f__norank_o__Chloroplast	g__norank_f__norank_o__Chloroplast	s__Stichococcus_bacillaris	OTU2116
d__Bacteria	k__norank_ d__Bacteria	p__Proteobacteria	c__Gammaproteobacteria	o__Diplorickettsiales	f__Diplorickettsiaceae	g__norank_f__Diplorickettsiaceae	s__unclassified_g__norank_f__Diplorickettsiaceae	OTU2383
d__Bacteria	k__norank_ d__Bacteria	p__Proteobacteria	c__Alphaproteobacteria	o__Micropepsales	f__Micropepsaceae	g__norank_f__Micropepsaceae	s__uncultured_bacterium_g__norank_f__Micropepsaceae	OTU1969
d__Bacteria	k__norank_ d__Bacteria	p__Bdellovibrionota	c__Oligoflexia	o__0319-6G20	f__norank_o__0319-6G20	g__norank_f__norank_o__0319-6G20	s__unclassified_g__norank_f__norank_o__0319-6G20	OTU1625
d__Bacteria	k__norank_ d__Bacteria	p__Actinobacteriota	c__Acidimicrobiia	o__Microtrichales	f__Ilumatobacteraceae	g__CL500-29_marine_group	s__metagenome_g__CL500-29_marine_group	OTU2765
d__Bacteria	k__norank_ d__Bacteria	p__Bdellovibrionota	c__Bdellovibrionia	o__Bdellovibrionales	f__Bdellovibrionaceae	g__Bdellovibrio	s__Bdellovibrio_sp._oral_clone_CA006	OTU190
d__Bacteria	k__norank_ d__Bacteria	p__Acidobacteriota	c__Vicinamibacteria	o__Vicinamibacterales	f__Vicinamibacteraceae	g__Luteitalea	s__uncultured_bacterium_gp6_g__Luteitalea	OTU2146
d__Bacteria	k__norank_ d__Bacteria	p__Gemmatimonadota	c__Longimicrobia	o__Longimicrobiales	f__Longimicrobiaceae	g__YC-ZSS-LKJ147	s__unclassified_g__YC-ZSS-LKJ147	OTU2682
d__Bacteria	k__norank_ d__Bacteria	p__Patescibacteria	c__Saccharimonadia	o__Saccharimonadales	f__LWQ8	g__norank_f__LWQ8	s__uncultured_bacterium_g__norank_f__LWQ8	OTU2763
d__Bacteria	k__norank_ d__Bacteria	p__Chloroflexi	c__Chloroflexia	o__Thermomicrobiales	f__JG30-KF-CM45	g__norank_f__JG30-KF-CM45	s__unclassified_g__norank_f__JG30-KF-CM45	OTU2542
d__Bacteria	k__norank_ d__Bacteria	p__Myxococcota	c__Polyangia	o__Blfdi19	f__norank_o__Blfdi19	g__norank_f__norank_o__Blfdi19	s__unclassified_g__norank_f__norank_o__Blfdi19	OTU1713
d__Bacteria	k__norank_ d__Bacteria	p__Actinobacteriota	c__Acidimicrobiia	o__IMCC26256	f__norank_o__IMCC26256	g__norank_f__norank_o__IMCC26256	s__uncultured_Acidimicrobiia_bacterium_g__norank_f__norank	OTU982
d__Bacteria	k__norank_ d__Bacteria	p__Myxococcota	c__Polyangia	o__Blfdi19	f__norank_o__Blfdi19	g__norank_f__norank_o__Blfdi19	s__unclassified_g__norank_f__norank_o__Blfdi19	OTU293

d__Bacteria	k__norank_d__Bacteria	p__WPS-2	c__norank_p__WPS-2	o__norank_c__norank_p__WPS-2	f__norank_o__norank_c__norank_p__WPS-2	g__norank_f__norank_o__norank_c__norank_p__WPS-2	s__unclassified_g__norank_f__norank_o__norank_c__norank_p__WPS-2	OTU537
d__Bacteria	k__norank_d__Bacteria	p__unclassified_k__norank_d__Bacteria	c__unclassified_k__norank_d__Bacteria	o__unclassified_k__norank_d__Bacteria	f__unclassified_k__norank_d__Bacteria	g__unclassified_k__norank_d__Bacteria	s__unclassified_k__norank_d__Bacteria	OTU1608
d__Bacteria	k__norank_d__Bacteria	p__Myxococcota	c__Myxococcia	o__Myxococcales	f__Myxococcaceae	g__P3OB-42	s__uncultured_Archangiaceae_bacterium_g__P3OB-42	OTU2634
d__Bacteria	k__norank_d__Bacteria	p__Chloroflexi	c__Ktedonobacteria	o__Ktedonobacterales	f__Ktedonobacteraceae	g__unclassified_f__Ktedonobacteraceae	s__unclassified_f__Ktedonobacteraceae	OTU2006
d__Bacteria	k__norank_d__Bacteria	p__Actinobacteriota	c__Actinobacteria	o__Micrococcales	f__Microbacteriaceae	g__Amnibacterium	s__unclassified_g__Amnibacterium	OTU1735
d__Bacteria	k__norank_d__Bacteria	p__Proteobacteria	c__Alphaproteobacteria	o__Sphingomonadales	f__Sphingomonadaceae	g__Polymorphobacter	s__Polymorphobacter_sp.	OTU2650
d__Bacteria	k__norank_d__Bacteria	p__Proteobacteria	c__Gammaproteobacteria	o__Legionellales	f__Legionellaceae	g__Legionella	s__uncultured_bacterium_g__Legionella	OTU481
d__Bacteria	k__norank_d__Bacteria	p__Gemmatimonadota	c__Gemmatimonadetes	o__Gemmatimonadales	f__Gemmatimonadaceae	g__unclassified_f__Gemmatimonadaceae	s__unclassified_f__Gemmatimonadaceae	OTU2507
d__Bacteria	k__norank_d__Bacteria	p__Patescibacteria	c__Saccharimonadia	o__Saccharimonadales	f__norank_o__Saccharimonadales	g__norank_f__norank_o__Saccharimonadales	s__unclassified_g__norank_f__norank_o__Saccharimonadales	OTU2155
d__Bacteria	k__norank_d__Bacteria	p__Chloroflexi	c__Chloroflexia	o__Kallotenuales	f__AKIW781	g__norank_f__AKIW781	s__uncultured_bacterium_g__norank_f__AKIW781	OTU1141
d__Bacteria	k__norank_d__Bacteria	p__Armatimonadota	c__Armatimonadia	o__Armatimonadales	f__unclassified_o__Armatimonadales	g__unclassified_o__Armatimonadales	s__unclassified_o__Armatimonadales	OTU178
d__Bacteria	k__norank_d__Bacteria	p__Myxococcota	c__Polyangia	o__Polyangiales	f__Phaselicystidaceae	g__Phaselicystis	s__unclassified_g__Phaselicystis	OTU385
d__Bacteria	k__norank_d__Bacteria	p__Proteobacteria	c__Alphaproteobacteria	o__Acetobacterales	f__Acetobacteraceae	g__Endobacter	s__Acetobacteraceae_bacterium_PAMC_26568	OTU1949
d__Bacteria	k__norank_d__Bacteria	p__Bdellovibrionota	c__Oligoflexia	o__Oligoflexales	f__norank_o__Oligoflexales	g__norank_f__norank_o__Oligoflexales	s__unclassified_g__norank_f__norank_o__Oligoflexales	OTU2814
d__Bacteria	k__norank_d__Bacteria	p__Chloroflexi	c__OLB14	o__norank_c__OLB14	f__norank_o__norank_c__OLB14	g__norank_f__norank_o__norank_c__OLB14	s__unclassified_g__norank_f__norank_o__norank_c__OLB14	OTU2057
d__Bacteria	k__norank_d__Bacteria	p__Proteobacteria	c__Gammaproteobacteria	o__Legionellales	f__Legionellaceae	g__Legionella	s__uncultured_bacterium_g__Legionella	OTU557

d__Bacteria	k__norank_d__Bacteria	p__WPS-2	c__norank_p__WPS-2	o__norank_c__norank_p__WPS-2	f__norank_o__norank_c__norank_p__WPS-2	g__norank_f__norank_o__norank_c__norank_p__WPS-2	s__unclassified_g__norank_f__norank_o__norank_c__norank_p__WPS-2	OTU997
d__Bacteria	k__norank_d__Bacteria	p__Bacteroidota	c__Bacteroidia	o__Chitinophagales	f__Chitinophagaceae	g__Aurantisolimonas	s__uncultured_bacterium_g__Aurantisolimonas	OTU559
d__Bacteria	k__norank_d__Bacteria	p__Actinobacteriota	c__Actinobacteria	o__Micromonosporales	f__Micromonosporaceae	g__Virgisporangium	s__Virgisporangium_ochraceum	OTU2684
d__Bacteria	k__norank_d__Bacteria	p__Gemmatimonadota	c__Gemmatimonadetes	o__Gemmatimonadales	f__Gemmatimonadaceae	g__norank_f__Gemmatimonadaceae	s__metagenome_g__norank_f__Gemmatimonadaceae	OTU521
d__Bacteria	k__norank_d__Bacteria	p__Proteobacteria	c__Alphaproteobacteria	o__Acetobacterales	f__Acetobacteraceae	g__Roseomonas	s__metagenome_g__Roseomonas	OTU2200
d__Bacteria	k__norank_d__Bacteria	p__Proteobacteria	c__Gammaproteobacteria	o__Legionellales	f__Legionellaceae	g__Legionella	s__uncultured_Legionella_sp._g__Legionella	OTU2498
d__Bacteria	k__norank_d__Bacteria	p__Proteobacteria	c__Alphaproteobacteria	o__Acetobacterales	f__Acetobacteraceae	g__Acidiphilium	s__unclassified_g__Acidiphilium	OTU1177
d__Bacteria	k__norank_d__Bacteria	p__Verrucomicrobiota	c__Verrucomicrobiae	o__Chthoniobacterales	f__Chthoniobacteraceae	g__Chthoniobacter	s__uncultured_bacterium_g__Chthoniobacter	OTU2233
d__Bacteria	k__norank_d__Bacteria	p__Bdellovibrionota	c__Bdellovibrionia	o__Bdellovibrionales	f__Bdellovibrionaceae	g__Bdellovibrio	s__unclassified_g__Bdellovibrio	OTU2587
d__Bacteria	k__norank_d__Bacteria	p__Actinobacteriota	c__Actinobacteria	o__Frankiales	f__Sporichthyaceae	g__Longivirga	s__uncultured_bacterium_g__Longivirga	OTU2371
d__Bacteria	k__norank_d__Bacteria	p__Proteobacteria	c__Alphaproteobacteria	o__Rhizobiales	f__Rhizobiaceae	g__Pseudochrobactrum	s__uncultured_bacterium_g__Pseudochrobactrum	OTU2903
d__Bacteria	k__norank_d__Bacteria	p__Chloroflexi	c__Chloroflexia	o__Elev-1554	f__norank_o__Elev-1554	g__norank_f__norank_o__Elev-1554	s__uncultured_bacterium_g__norank_f__norank_o__Elev-1554	OTU1009
d__Bacteria	k__norank_d__Bacteria	p__Actinobacteriota	c__Actinobacteria	o__Propionibacteriales	f__Propionibacteriaceae	g__Raineyella	s__uncultured_bacterium_g__Raineyella	OTU2704
d__Bacteria	k__norank_d__Bacteria	p__Proteobacteria	c__Gammaproteobacteria	o__Diplorickettsiales	f__Diplorickettsiaceae	g__norank_f__Diplorickettsiaceae	s__unclassified_g__norank_f__Diplorickettsiaceae	OTU5
d__Bacteria	k__norank_d__Bacteria	p__Proteobacteria	c__Alphaproteobacteria	o__Rhizobiales	f__Rhizobiales_Incertae_Sedis	g__Bauldia	s__uncultured_bacterium_g__Bauldia	OTU2920
d__Bacteria	k__norank_d__Bacteria	p__Elusimicrobiota	c__Elusimicrobia	o__Lineage_IV	f__norank_o__Lineage_IV	g__norank_f__norank_o__Lineage_IV	s__uncultured_bacterium_g__norank_f__norank_o__Lineage_IV	OTU680

d__Bacteria	k__norank_d__Bacteria	p__Chloroflexi	c__Chloroflexia	o__Thermomicrobiales	f__JG30-KF-CM45	g__norank_f__JG30-KF-CM45	s__bacterium_Ellin6537	OTU1094
d__Bacteria	k__norank_d__Bacteria	p__Chloroflexi	c__AD3	o__norank_c__AD3	f__norank_o__norank_c__AD3	g__norank_f__norank_o__norank_c__AD3	s__unclassified_g__norank_f__norank_o__norank_c__AD3	OTU1891
d__Bacteria	k__norank_d__Bacteria	p__Bdellovibrionota	c__Oligoflexia	o__0319-6G20	f__norank_o__0319-6G20	g__norank_f__norank_o__0319-6G20	s__unclassified_g__norank_f__norank_o__0319-6G20	OTU2766
d__Bacteria	k__norank_d__Bacteria	p__Chloroflexi	c__Anaerolineae	o__SBR1031	f__A4b	g__norank_f__A4b	s__uncultured_bacterium_g__norank_f__A4b	OTU460
d__Bacteria	k__norank_d__Bacteria	p__Cyanobacteria	c__Cyanobacteriia	o__Chloroplast	f__norank_o__Chloroplast	g__norank_f__norank_o__Chloroplast	s__unclassified_g__norank_f__norank_o__Chloroplast	OTU2202
d__Bacteria	k__norank_d__Bacteria	p__Bacteroidota	c__Bacteroidia	o__Chitinophagales	f__Chitinophagaceae	g__Niastella	s__uncultured_bacterium_g__Niastella	OTU1585
d__Bacteria	k__norank_d__Bacteria	p__Actinobacteriota	c__Actinobacteria	o__Actinomycetales	f__Actinomycetaceae	g__Actinomyces	s__Schaalia_odontolytica	OTU640
d__Bacteria	k__norank_d__Bacteria	p__Proteobacteria	c__Gammaproteobacteria	o__Burkholderiales	f__A21b	g__norank_f__A21b	s__uncultured_bacterium_g__norank_f__A21b	OTU27
d__Bacteria	k__norank_d__Bacteria	p__Myxococcota	c__Polyangia	o__unclassified_c__Polyangia	f__unclassified_c__Polyangia	g__unclassified_c__Polyangia	s__unclassified_c__Polyangia	OTU1167
d__Bacteria	k__norank_d__Bacteria	p__Bdellovibrionota	c__Oligoflexia	o__0319-6G20	f__norank_o__0319-6G20	g__norank_f__norank_o__0319-6G20	s__unclassified_g__norank_f__norank_o__0319-6G20	OTU1889
d__Bacteria	k__norank_d__Bacteria	p__Actinobacteriota	c__Acidimicrobiia	o__Microtrichales	f__Iamiaceae	g__Iamia	s__unclassified_g__Iamia	OTU2651
d__Bacteria	k__norank_d__Bacteria	p__WPS-2	c__norank_p__WPS-2	o__norank_c__norank_p__WPS-2	f__norank_o__norank_c__norank_p__WPS-2	g__norank_f__norank_o__norank_c__norank_p__WPS-2	s__unclassified_g__norank_f__norank_o__norank_c__norank_p__WPS-2	OTU2919
d__Bacteria	k__norank_d__Bacteria	p__Bacteroidota	c__Bacteroidia	o__Cytophagales	f__Spirosomaceae	g__Spirosoma	s__uncultured_bacterium_g__Spirosoma	OTU1705
d__Bacteria	k__norank_d__Bacteria	p__Myxococcota	c__Myxococcia	o__Myxococcales	f__Anaeromyxobacteraceae	g__Anaeromyxobacter	s__uncultured_bacterium_g__Anaeromyxobacter	OTU2508
d__Bacteria	k__norank_d__Bacteria	p__Myxococcota	c__Polyangia	o__Nannocystales	f__Nannocystaceae	g__Nannocystis	s__metagenome_g__Nannocystis	OTU2179
d__Bacteria	k__norank_d__Bacteria	p__Myxococcota	c__Myxococcia	o__Myxococcales	f__Myxococcaceae	g__P3OB-42	s__uncultured_bacterium_g__P3OB-42	OTU69

d__Bacteria	k__norank_d__Bacteria	p__Proteobacteria	c__Gammaproteobacteria	o__Burkholderiales	f__Nitrosomonadaceae	g__Nitrosomonas	s__unclassified_g__Nitrosomonas	OTU2422
d__Bacteria	k__norank_d__Bacteria	p__Gemmatimonadota	c__Gemmatimonadetes	o__Gemmatimonadales	f__Gemmatimonadaceae	g__Gemmatimonas	s__unclassified_g__Gemmatimonas	OTU1597
d__Bacteria	k__norank_d__Bacteria	p__Bdellovibrionota	c__Oligoflexia	o__0319-6G20	f__norank_o__0319-6G20	g__norank_f__norank_o__0319-6G20	s__unclassified_g__norank_f__norank_o__0319-6G20	OTU991
d__Bacteria	k__norank_d__Bacteria	p__Patescibacteria	c__Saccharimonadia	o__Saccharimonadales	f__norank_o__Saccharimonadales	g__norank_f__norank_o__Saccharimonadales	s__unclassified_g__norank_f__norank_o__Saccharimonadales	OTU295
d__Bacteria	k__norank_d__Bacteria	p__Gemmatimonadota	c__BD2-11_terrestrial_group	o__norank_c__BD2-11_terrestrial_group	f__norank_o__norank_c__BD2-11_terrestrial_group	g__norank_f__norank_o__norank_c__BD2-11_terrestrial_group	s__metagenome_g__norank_f__norank_o__norank_c__BD2-11_terrestrial_group	OTU471
d__Bacteria	k__norank_d__Bacteria	p__Actinobacteriota	c__Actinobacteria	o__Frankiales	f__Sporichthyaceae	g__Sporichthya	s__metagenome_g__Sporichthya	OTU2710
d__Bacteria	k__norank_d__Bacteria	p__Acidobacteriota	c__Blastocatellia	o__11-24	f__norank_o__11-24	g__norank_f__norank_o__11-24	s__uncultured_Acidobacteria_bacterium_g__norank_f__norank_o__11-24	OTU1532
d__Bacteria	k__norank_d__Bacteria	p__Actinobacteriota	c__Thermoleophilia	o__Solirubrobacterales	f__Solirubrobacteraceae	g__Conexibacter	s__metagenome_g__Conexibacter	OTU2804
d__Bacteria	k__norank_d__Bacteria	p__Chloroflexi	c__unclassified_p__Chloroflexi	o__unclassified_p__Chloroflexi	f__unclassified_p__Chloroflexi	g__unclassified_p__Chloroflexi	s__unclassified_p__Chloroflexi	OTU2784
d__Bacteria	k__norank_d__Bacteria	p__Proteobacteria	c__Alphaproteobacteria	o__Rhizobiales	f__Beijerinckiaceae	g__Methylobacterium-Methylorubrum	s__Methylobacterium_brachiatum	OTU22
d__Bacteria	k__norank_d__Bacteria	p__Myxococcota	c__Polyangia	o__Polyangiales	f__Polyangiaceae	g__Minicystis	s__unclassified_g__Minicystis	OTU1814
d__Bacteria	k__norank_d__Bacteria	p__Cyanobacteria	c__Cyanobacteriia	o__Leptolyngbyales	f__Leptolyngbyaceae	g__norank_f__Leptolyngbyaceae	s__uncultured_bacterium_g__norank_f__Leptolyngbyaceae	OTU1489
d__Bacteria	k__norank_d__Bacteria	p__Gemmatimonadota	c__Gemmatimonadetes	o__Gemmatimonadales	f__Gemmatimonadaceae	g__Roseisolibacter	s__unclassified_g__Roseisolibacter	OTU1466
d__Bacteria	k__norank_d__Bacteria	p__Bdellovibrionota	c__Oligoflexia	o__0319-6G20	f__norank_o__0319-6G20	g__norank_f__norank_o__0319-6G20	s__unclassified_g__norank_f__norank_o__0319-6G20	OTU1885
d__Bacteria	k__norank_d__Bacteria	p__Patescibacteria	c__Saccharimonadia	o__Saccharimonadales	f__norank_o__Saccharimonadales	g__norank_f__norank_o__Saccharimonadales	s__unclassified_g__norank_f__norank_o__Saccharimonadales	OTU1020
d__Bacteria	k__norank_d__Bacteria	p__Bdellovibrionota	c__Oligoflexia	o__Oligoflexales	f__norank_o__Oligoflexales	g__norank_f__norank_o__Oligoflexales	s__unclassified_g__norank_f__norank_o__Oligoflexales	OTU2140

d__Bacteria	k__norank_ d__Bacteria	p__Planctomycetota	c__Planctomycetes	o__Gemmatales	f__Gemmataceae	g__norank_f__Gemmataceae	s__unclassified_g__norank_f__Gemmataceae	OTU234
d__Bacteria	k__norank_ d__Bacteria	p__Gemmatimonadota	c__Gemmatimonadetes	o__Gemmatimonadales	f__Gemmatimonadaceae	g__norank_f__Gemmatimonadaceae	s__uncultured_Gemmatimonadales_bacterium_g__norank_f__Gemmatimonadaceae	OTU2663
d__Bacteria	k__norank_ d__Bacteria	p__Chloroflexi	c__Anaerolineae	o__Caldilineales	f__Caldilineaceae	g__norank_f__Caldilineaceae	s__uncultured_bacterium_g__norank_f__Caldilineaceae	OTU2487
d__Bacteria	k__norank_ d__Bacteria	p__Bacteroidota	c__Bacteroidia	o__Sphingobacteriales	f__NS11-12_marine_group	g__norank_f__NS11-12_marine_group	s__metagenome_g__norank_f__NS11-12_marine_group	OTU1254
d__Bacteria	k__norank_ d__Bacteria	p__Bacteroidota	c__Bacteroidia	o__Chitinophagales	f__Chitinophagaceae	g__norank_f__Chitinophagaceae	s__unclassified_g__norank_f__Chitinophagaceae	OTU2645
d__Bacteria	k__norank_ d__Bacteria	p__unclassified_k__no rank_d__Bacteria	c__unclassified_k__noran k_d__Bacteria	o__unclassified_k__norank_ d__Bacteria	f__unclassified_k__norank_d__Bacteria	g__unclassified_k__norank_d__Bacteri a	s__unclassified_k__norank_d__Bacteria	OTU1669
d__Bacteria	k__norank_ d__Bacteria	p__Chloroflexi	c__TK10	o__norank_c__TK10	f__norank_o__norank_c__TK10	g__norank_f__norank_o__norank_c__ TK10	s__metagenome_g__norank_f__norank_o__norank_c__TK10	OTU2333
d__Bacteria	k__norank_ d__Bacteria	p__Chloroflexi	c__AD3	o__norank_c__AD3	f__norank_o__norank_c__AD3	g__norank_f__norank_o__norank_c__ AD3	s__unclassified_g__norank_f__norank_o__norank_c__AD3	OTU1437
d__Bacteria	k__norank_ d__Bacteria	p__Bacteroidota	c__Bacteroidia	o__Cytophagales	f__Microscillaceae	g__norank_f__Microscillaceae	s__uncultured_soil_bacterium_g__norank_f__Microscillaceae	OTU2064
d__Bacteria	k__norank_ d__Bacteria	p__Planctomycetota	c__Planctomycetes	o__Isosphaerales	f__Isosphaeraceae	g__Singulisphaera	s__uncultured_bacterium_g__Singulisphaera	OTU1208
d__Bacteria	k__norank_ d__Bacteria	p__Planctomycetota	c__Planctomycetes	o__Gemmatales	f__Gemmataceae	g__norank_f__Gemmataceae	s__uncultured_bacterium_g__norank_f__Gemmataceae	OTU2497
d__Bacteria	k__norank_ d__Bacteria	p__Planctomycetota	c__Planctomycetes	o__Gemmatales	f__Gemmataceae	g__norank_f__Gemmataceae	s__uncultured_bacterium_g__norank_f__Gemmataceae	OTU1102
d__Bacteria	k__norank_ d__Bacteria	p__Acidobacteriota	c__Vicinamibacteria	o__Vicinamibacterales	f__norank_o__Vicinamibacterales	g__norank_f__norank_o__Vicinamiba cterales	s__unclassified_g__norank_f__norank_o__Vicinamibacterales	OTU1380
d__Bacteria	k__norank_ d__Bacteria	p__Chloroflexi	c__Chloroflexia	o__Thermomicrobiales	f__JG30-KF-CM45	g__norank_f__JG30-KF-CM45	s__unclassified_g__norank_f__JG30-KF-CM45	OTU2991
d__Bacteria	k__norank_ d__Bacteria	p__Proteobacteria	c__Gammaproteobacteria	o__Diplorickettsiales	f__Diplorickettsiaceae	g__norank_f__Diplorickettsiaceae	s__unclassified_g__norank_f__Diplorickettsiaceae	OTU464
d__Bacteria	k__norank_ d__Bacteria	p__Actinobacteriota	c__Acidimicrobiia	o__norank_c__Acidimicrobi ia	f__norank_o__norank_c__Acidimicrobiia	g__norank_f__norank_o__norank_c__ Acidimicrobiia	s__metagenome_g__norank_f__norank_o__norank_c__Acidimicrobiia	OTU2698

d__Bacteria	k__norank_ d__Bacteria	p__Chloroflexi	c__Anaerolineae	o__norank_c__Anaerolineae	f__norank_o__norank_c__Anaerolineae	g__norank_f__norank_o__norank_c__ Anaerolineae	s__uncultured_Bellilinea_sp._g__norank_f__norank	OTU1113
d__Bacteria	k__norank_ d__Bacteria	p__Proteobacteria	c__Gammaproteobacteria	o__Xanthomonadales	f__Rhodanobacteraceae	g__unclassified_f__Rhodanobacteracea e	s__unclassified_f__Rhodanobacteraceae	OTU1369
d__Bacteria	k__norank_ d__Bacteria	p__Gemmatimonadota	c__Gemmatimonadetes	o__Gemmatimonadales	f__Gemmatimonadaceae	g__norank_f__Gemmatimonadaceae	s__uncultured_soil_bacterium_g__norank_f__Gemmatimonadaceae	OTU431
d__Bacteria	k__norank_ d__Bacteria	p__Bacteroidota	c__Bacteroidia	o__Chitinophagales	f__Chitinophagaceae	g__Flavisolibacter	s__uncultured_Flavisolibacter_sp._g__Flavisolibacter	OTU1388
d__Bacteria	k__norank_ d__Bacteria	p__Myxococcota	c__Polyangia	o__Polyangiales	f__Sandaracinaceae	g__norank_f__Sandaracinaceae	s__metagenome_g__norank_f__Sandaracinaceae	OTU764
d__Bacteria	k__norank_ d__Bacteria	p__Myxococcota	c__Polyangia	o__Polyangiales	f__Polyangiaceae	g__Pajaroellobacter	s__uncultured_bacterium_g__Pajaroellobacter	OTU2511
d__Bacteria	k__norank_ d__Bacteria	p__Actinobacteriota	c__Actinobacteria	o__Propionibacteriales	f__Propionibacteriaceae	g__Aestuariimicrobium	s__Mycobacterium_abscessus_g__Aestuariimicrobium	OTU2703
d__Bacteria	k__norank_ d__Bacteria	p__Chloroflexi	c__Chloroflexia	o__Chloroflexales	f__Roseiflexaceae	g__norank_f__Roseiflexaceae	s__uncultured_Chloroflexaceae_bacterium_g__norank_f__Roseiflexaceae	OTU2264
d__Bacteria	k__norank_ d__Bacteria	p__Actinobacteriota	c__Thermoleophilia	o__Solirubrobacterales	f__Solirubrobacteraceae	g__Conexibacter	s__unclassified_g__Conexibacter	OTU1684
d__Bacteria	k__norank_ d__Bacteria	p__Proteobacteria	c__Alphaproteobacteria	o__Micavibrionales	f__unclassified_o__Micavibrionales	g__unclassified_o__Micavibrionales	s__unclassified_o__Micavibrionales	OTU2843
d__Bacteria	k__norank_ d__Bacteria	p__Proteobacteria	c__Alphaproteobacteria	o__Acetobacterales	f__Acetobacteraceae	g__Roseomonas	s__unclassified_g__Roseomonas	OTU669
d__Bacteria	k__norank_ d__Bacteria	p__Patescibacteria	c__Parcubacteria	o__norank_c__Parcubacteria	f__norank_o__norank_c__Parcubacteria	g__norank_f__norank_o__norank_c__ Parcubacteria	s__uncultured_bacterium_g__norank_f__norank_o__norank_c__Parcubacteria	OTU879
d__Bacteria	k__norank_ d__Bacteria	p__Chloroflexi	c__TK10	o__norank_c__TK10	f__norank_o__norank_c__TK10	g__norank_f__norank_o__norank_c__ TK10	s__unclassified_g__norank_f__norank_o__norank_c__TK10	OTU1133
d__Bacteria	k__norank_ d__Bacteria	p__Cyanobacteria	c__Cyanobacteriia	o__Chloroplast	f__norank_o__Chloroplast	g__norank_f__norank_o__Chloroplast	s__Castanea_mollissima_Chinese_chestnut_g__norank	OTU50
d__Bacteria	k__norank_ d__Bacteria	p__Proteobacteria	c__Gammaproteobacteria	o__Burkholderiales	f__Comamonadaceae	g__unclassified_f__Comamonadaceae	s__unclassified_f__Comamonadaceae	OTU2107
d__Bacteria	k__norank_ d__Bacteria	p__Proteobacteria	c__Alphaproteobacteria	o__Caulobacterales	f__Caulobacteraceae	g__PMMR1	s__uncultured_bacterium_g__PMMR1	OTU2834

d__Bacteria	k__norank_ d__Bacteria	p__Elusimicrobiota	c__Lineage_IIa	o__norank_c__Lineage_IIa	f__norank_o__norank_c__Lineage_IIa	g__norank_f__norank_o__norank_c__ Lineage_IIa	s__uncultured_bacterium_g__norank_f__norank_o__norank_c__Lineage_IIa	OTU1185
d__Bacteria	k__norank_ d__Bacteria	p__Proteobacteria	c__Alphaproteobacteria	o__Rhizobiales	f__Hyphomicrobiaceae	g__Hyphomicrobium	s__unclassified_g__Hyphomicrobium	OTU418
d__Bacteria	k__norank_ d__Bacteria	p__Bacteroidota	c__Bacteroidia	o__Chitinophagales	f__Chitinophagaceae	g__Edaphobaculum	s__unclassified_g__Edaphobaculum	OTU233
d__Bacteria	k__norank_ d__Bacteria	p__Planctomycetota	c__Planctomycetes	o__Gemmatales	f__Gemmataceae	g__norank_f__Gemmataceae	s__unclassified_g__norank_f__Gemmataceae	OTU2738
d__Bacteria	k__norank_ d__Bacteria	p__Bdellovibrionota	c__Bdellovibrionia	o__Bdellovibrionales	f__Bdellovibrionaceae	g__Bdellovibrio	s__uncultured_bacterium_g__Bdellovibrio	OTU2719
d__Bacteria	k__norank_ d__Bacteria	p__Chloroflexi	c__Anaerolineae	o__SBR1031	f__A4b	g__norank_f__A4b	s__uncultured_soil_bacterium_g__norank_f__A4b	OTU2760
d__Bacteria	k__norank_ d__Bacteria	p__Proteobacteria	c__Alphaproteobacteria	o__Rhizobiales	f__Xanthobacteraceae	g__Variibacter	s__unclassified_g__Variibacter	OTU2952
d__Bacteria	k__norank_ d__Bacteria	p__Chloroflexi	c__Ktedonobacteria	o__Ktedonobacterales	f__Ktedonobacteraceae	g__1921-3	s__uncultured_bacterium_g__1921-3	OTU1774
d__Bacteria	k__norank_ d__Bacteria	p__Chloroflexi	c__AD3	o__norank_c__AD3	f__norank_o__norank_c__AD3	g__norank_f__norank_o__norank_c__ AD3	s__uncultured_bacterium_g__norank_f__norank_o__norank_c__AD3	OTU1846
d__Bacteria	k__norank_ d__Bacteria	p__Acidobacteriota	c__Thermoanaerobaculia	o__Thermoanaerobaculales	f__Thermoanaerobaculaceae	g__Subgroup_10	s__unclassified_g__Subgroup_10	OTU2085
d__Bacteria	k__norank_ d__Bacteria	p__Patescibacteria	c__Saccharimonadia	o__Saccharimonadales	f__Saccharimonadaceae	g__TM7a	s__uncultured_bacterium_g__TM7a	OTU2685
d__Bacteria	k__norank_ d__Bacteria	p__Myxococcota	c__Myxococcia	o__Myxococcales	f__Myxococcaceae	g__Archangium	s__Archangium_gephyra	OTU1671
d__Bacteria	k__norank_ d__Bacteria	p__Actinobacteriota	c__Thermoleophilia	o__Solirubrobacterales	f__Solirubrobacteraceae	g__Solirubrobacter	s__uncultured_bacterium_g__Solirubrobacter	OTU2798
d__Bacteria	k__norank_ d__Bacteria	p__Patescibacteria	c__Saccharimonadia	o__Saccharimonadales	f__norank_o__Saccharimonadales	g__norank_f__norank_o__Saccharimo nadales	s__unclassified_g__norank_f__norank_o__Saccharimonadales	OTU1156
d__Bacteria	k__norank_ d__Bacteria	p__Acidobacteriota	c__Acidobacteriae	o__Bryobacterales	f__Bryobacteraceae	g__Bryobacter	s__metagenome_g__Bryobacter	OTU2513
d__Bacteria	k__norank_ d__Bacteria	p__Myxococcota	c__Polyangia	o__Haliangiales	f__Haliangiaceae	g__Haliangium	s__uncultured_prokaryote_g__Haliangium	OTU310

d__Bacteria	k__norank_d__Bacteria	p__Proteobacteria	c__Alphaproteobacteria	o__Tistrellales	f__Geminicoccaceae	g__Candidatus_Alysiosphaera	s__metagenome_g__Candidatus_Alysiosphaera	OTU413
d__Bacteria	k__norank_d__Bacteria	p__Actinobacteriota	c__Actinobacteria	o__Micromonosporales	f__Micromonosporaceae	g__unclassified_f__Micromonosporaceae	s__unclassified_f__Micromonosporaceae	OTU2392
d__Bacteria	k__norank_d__Bacteria	p__Actinobacteriota	c__Thermoleophilia	o__Solirubrobacterales	f__Solirubrobacteraceae	g__Conexibacter	s__unclassified_g__Conexibacter	OTU2430
d__Bacteria	k__norank_d__Bacteria	p__Cyanobacteria	c__Cyanobacteriia	o__Chloroplast	f__norank_o__Chloroplast	g__norank_f__norank_o__Chloroplast	s__unclassified_g__norank_f__norank_o__Chloroplast	OTU2837
d__Bacteria	k__norank_d__Bacteria	p__Planctomycetota	c__Planctomycetes	o__Gemmatales	f__Gemmataceae	g__norank_f__Gemmataceae	s__unclassified_g__norank_f__Gemmataceae	OTU1168
d__Bacteria	k__norank_d__Bacteria	p__WPS-2	c__norank_p__WPS-2	o__norank_c__norank_p__WPS-2	f__norank_o__norank_c__norank_p__WPS-2	g__norank_f__norank_o__norank_c__norank_p__WPS-2	s__uncultured_bacterium_g__norank_f__norank_o__norank_c__norank_p__WPS-2	OTU1869
d__Bacteria	k__norank_d__Bacteria	p__Proteobacteria	c__Alphaproteobacteria	o__Rhodobacterales	f__Rhodobacteraceae	g__Amaricoccus	s__uncultured_Amaricoccus_sp._g__Amaricoccus	OTU516
d__Bacteria	k__norank_d__Bacteria	p__Chloroflexi	c__JG30-KF-CM66	o__norank_c__JG30-KF-CM66	f__norank_o__norank_c__JG30-KF-CM66	g__norank_f__norank_o__norank_c__JG30-KF-CM66	s__unclassified_g__norank_f__norank_o__norank_c__JG30-KF-CM66	OTU1931
d__Bacteria	k__norank_d__Bacteria	p__Proteobacteria	c__Gammaproteobacteria	o__Burkholderiales	f__Oxalobacteraceae	g__norank_f__Oxalobacteraceae	s__unclassified_g__norank_f__Oxalobacteraceae	OTU2665
d__Bacteria	k__norank_d__Bacteria	p__WPS-2	c__norank_p__WPS-2	o__norank_c__norank_p__WPS-2	f__norank_o__norank_c__norank_p__WPS-2	g__norank_f__norank_o__norank_c__norank_p__WPS-2	s__uncultured_bacterium_g__norank_f__norank_o__norank_c__norank_p__WPS-2	OTU1867
d__Bacteria	k__norank_d__Bacteria	p__Acidobacteriota	c__Vicinamibacteria	o__Vicinamibacterales	f__norank_o__Vicinamibacterales	g__norank_f__norank_o__Vicinamibacterales	s__unclassified_g__norank_f__norank_o__Vicinamibacterales	OTU857
d__Bacteria	k__norank_d__Bacteria	p__Myxococcota	c__Polyangia	o__Polyangiales	f__Phaselicystidaceae	g__Phaselicystis	s__uncultured_Polyangiaceae_bacterium_g__Phaselicystis	OTU2260
d__Bacteria	k__norank_d__Bacteria	p__Myxococcota	c__Polyangia	o__mle1-27	f__norank_o__mle1-27	g__norank_f__norank_o__mle1-27	s__unclassified_g__norank_f__norank_o__mle1-27	OTU2808
d__Bacteria	k__norank_d__Bacteria	p__Proteobacteria	c__Gammaproteobacteria	o__Burkholderiales	f__Nitrosomonadaceae	g__Ellin6067	s__Betaproteobacteria_bacterium_GR16-43	OTU2674
d__Bacteria	k__norank_d__Bacteria	p__Actinobacteriota	c__Acidimicrobiia	o__Microtrichales	f__norank_o__Microtrichales	g__norank_f__norank_o__Microtrichales	s__uncultured_Actinomycetales_bacterium_g__norank_f__norank_o__Microtrichales	OTU331
d__Bacteria	k__norank_d__Bacteria	p__Planctomycetota	c__Planctomycetes	o__Gemmatales	f__Gemmataceae	g__norank_f__Gemmataceae	s__unclassified_g__norank_f__Gemmataceae	OTU1543

d__Bacteria	k__norank_d__Bacteria	p__Bacteroidota	c__Bacteroidia	o__Chitinophagales	f__Chitinophagaceae	g__Terrimonas	s__metagenome_g__Terrimonas	OTU665
d__Bacteria	k__norank_d__Bacteria	p__Proteobacteria	c__Alphaproteobacteria	o__Rhodobacterales	f__Rhodobacteraceae	g__Cereibacter	s__Cereibacter_changlensis	OTU1219
d__Bacteria	k__norank_d__Bacteria	p__Chloroflexi	c__Anaerolineae	o__SBR1031	f__norank_o__SBR1031	g__norank_f__norank_o__SBR1031	s__unclassified_g__norank_f__norank_o__SBR1031	OTU589
d__Bacteria	k__norank_d__Bacteria	p__Proteobacteria	c__Gammaproteobacteria	o__Xanthomonadales	f__Xanthomonadaceae	g__Lysobacter	s__unclassified_g__Lysobacter	OTU2204
d__Bacteria	k__norank_d__Bacteria	p__Acidobacteriota	c__Blastocatellia	o__Pyrinomonadales	f__Pyrinomonadaceae	g__RB41	s__uncultured_bacterium_g__RB41	OTU1243
d__Bacteria	k__norank_d__Bacteria	p__Actinobacteriota	c__Thermoleophilia	o__Gaiellales	f__Gaiellaceae	g__Gaiella	s__unclassified_g__Gaiella	OTU694
d__Bacteria	k__norank_d__Bacteria	p__Chloroflexi	c__Ktedonobacteria	o__C0119	f__norank_o__C0119	g__norank_f__norank_o__C0119	s__uncultured_soil_bacterium_g__norank_f__norank_o__C0119	OTU826
d__Bacteria	k__norank_d__Bacteria	p__Acidobacteriota	c__Acidobacteriae	o__Acidobacteriales	f__norank_o__Acidobacteriales	g__norank_f__norank_o__Acidobacteriales	s__uncultured_Acidobacteria_bacterium_g__norank_f__norank_o__Acidobacteriales	OTU1870
d__Bacteria	k__norank_d__Bacteria	p__Chloroflexi	c__OLB14	o__norank_c__OLB14	f__norank_o__norank_c__OLB14	g__norank_f__norank_o__norank_c__OLB14	s__uncultured_bacterium_g__norank_f__norank_o__norank_c__OLB14	OTU1249
d__Bacteria	k__norank_d__Bacteria	p__Acidobacteriota	c__Blastocatellia	o__11-24	f__norank_o__11-24	g__norank_f__norank_o__11-24	s__uncultured_bacterium_g__norank_f__norank_o__11-24	OTU984
d__Bacteria	k__norank_d__Bacteria	p__Proteobacteria	c__Gammaproteobacteria	o__Burkholderiales	f__Nitrosomonadaceae	g__MND1	s__unclassified_g__MND1	OTU2938
d__Bacteria	k__norank_d__Bacteria	p__Myxococcota	c__Polyangia	o__Blfdi19	f__norank_o__Blfdi19	g__norank_f__norank_o__Blfdi19	s__unclassified_g__norank_f__norank_o__Blfdi19	OTU2534
d__Bacteria	k__norank_d__Bacteria	p__Cyanobacteria	c__Cyanobacteriia	o__Chloroplast	f__norank_o__Chloroplast	g__norank_f__norank_o__Chloroplast	s__Vischeria_sp._CAUP_Q_202	OTU1539
d__Bacteria	k__norank_d__Bacteria	p__Actinobacteriota	c__Acidimicrobiia	o__norank_c__Acidimicrobiia	f__norank_o__norank_c__Acidimicrobiia	g__norank_f__norank_o__norank_c__Acidimicrobiia	s__uncultured_bacterium_g__norank_f__norank_o__norank_c__Acidimicrobiia	OTU245
d__Bacteria	k__norank_d__Bacteria	p__Proteobacteria	c__Gammaproteobacteria	o__Burkholderiales	f__Comamonadaceae	g__unclassified_f__Comamonadaceae	s__unclassified_f__Comamonadaceae	OTU110
d__Bacteria	k__norank_d__Bacteria	p__Chloroflexi	c__Ktedonobacteria	o__C0119	f__norank_o__C0119	g__norank_f__norank_o__C0119	s__uncultured_bacterium_g__norank_f__norank_o__C0119	OTU1763

d__Bacteria	k__norank_ d__Bacteria	p__Acidobacteriota	c__Blastocatellia	o__DS-100	f__norank_o__DS-100	g__norank_f__norank_o__DS-100	s__uncultured_Acidobacteriales_bacterium_g__norank_f__norank	OTU1305
d__Bacteria	k__norank_ d__Bacteria	p__Actinobacteriota	c__Acidimicrobiia	o__IMCC26256	f__norank_o__IMCC26256	g__norank_f__norank_o__IMCC26256	s__uncultured_Acidimicrobiia_bacterium_g__norank_f__norank	OTU2568
d__Bacteria	k__norank_ d__Bacteria	p__Bacteroidota	c__Bacteroidia	o__Sphingobacteriales	f__AKYH767	g__norank_f__AKYH767	s__unclassified_g__norank_f__AKYH767	OTU2238
d__Bacteria	k__norank_ d__Bacteria	p__Verrucomicrobiota	c__Chlamydiae	o__Chlamydiales	f__Simkaniaceae	g__norank_f__Simkaniaceae	s__metagenome_g__norank_f__Simkaniaceae	OTU527
d__Bacteria	k__norank_ d__Bacteria	p__Bdellovibrionota	c__Oligoflexia	o__0319-6G20	f__norank_o__0319-6G20	g__norank_f__norank_o__0319-6G20	s__metagenome_g__norank_f__norank_o__0319-6G20	OTU1211
d__Bacteria	k__norank_ d__Bacteria	p__Actinobacteriota	c__Actinobacteria	o__Frankiales	f__norank_o__Frankiales	g__norank_f__norank_o__Frankiales	s__uncultured_bacterium_g__norank_f__norank_o__Frankiales	OTU106
d__Bacteria	k__norank_ d__Bacteria	p__Chloroflexi	c__TK10	o__norank_c__TK10	f__norank_o__norank_c__TK10	g__norank_f__norank_o__norank_c__ TK10	s__uncultured_bacterium_g__norank_f__norank_o__norank_c__TK10	OTU2641
d__Bacteria	k__norank_ d__Bacteria	p__Bacteroidota	c__SJA-28	o__norank_c__SJA-28	f__norank_o__norank_c__SJA-28	g__norank_f__norank_o__norank_c__ SJA-28	s__uncultured_bacterium_g__norank_f__norank_o__norank_c__SJA-28	OTU2121
d__Bacteria	k__norank_ d__Bacteria	p__Planctomycetota	c__Planctomycetes	o__norank_c__Planctomycet es	f__norank_o__norank_c__Planctomycete s	g__norank_f__norank_o__norank_c__ Planctomycetes	s__uncultured_bacterium_g__norank_f__norank_o__norank_c__Planctomycetes	OTU2074
d__Bacteria	k__norank_ d__Bacteria	p__Chloroflexi	c__Ktedonobacteria	o__Ktedonobacterales	f__Ktedonobacteraceae	g__1921-2	s__uncultured_bacterium_g__1921-2	OTU1793
d__Bacteria	k__norank_ d__Bacteria	p__Proteobacteria	c__Gammaproteobacteria	o__Xanthomonadales	f__Rhodanobacteraceae	g__Dokdonella	s__uncultured_bacterium_g__Dokdonella	OTU2050
d__Bacteria	k__norank_ d__Bacteria	p__Chloroflexi	c__TK10	o__norank_c__TK10	f__norank_o__norank_c__TK10	g__norank_f__norank_o__norank_c__ TK10	s__unclassified_g__norank_f__norank_o__norank_c__TK10	OTU1913
d__Bacteria	k__norank_ d__Bacteria	p__Bdellovibrionota	c__Oligoflexia	o__Silvanigrellales	f__Silvanigrellaceae	g__norank_f__Silvanigrellaceae	s__uncultured_bacterium_g__norank_f__Silvanigrellaceae	OTU2810
